# Boron Enabled Directed [2+2]- and Dearomative [4+2]-Cycloadditions Initiated by Energy Transfer

**DOI:** 10.1002/anie.202416215

**Published:** 2024-11-07

**Authors:** Souvik Adak, Partha Sarathi Hazra, Carter B. Fox, M. Kevin Brown

**Affiliations:** Department of Chemistry, Indiana University, 800 E. Kirkwood Ave. Bloomington, IN 47401, US

**Keywords:** Cycloaddition, Photochemistry, Cyclobutanes, Directed Reaction

## Abstract

A strategy for the photosensitized [2+2]-cycloaddition between styrenyl dihaloboranes and unactivated allylamines to access cyclobutylboronates with control of stereochemistry and regiochemistry is presented. The success of the reaction relies on the temporary coordination between in situ generated dihaloboranes and amines under mild reaction conditions. In addition, cyclobutanes with varying substitution patterns have been prepared using *N*-heterocycles as directing group. Manipulation of the C–B bond allows for the synthesis of a diverse class of cyclobutanes from simple precursors. Moreover, these reactions lead to the synthesis of complex amines and heteroaromatic compounds, which have significant utility in medicinal chemistry. Finally, a dearomative [4+2]-cycloaddition of naphthalenes using a boron-enabled temporary tethering strategy has also been uncovered to synthesize complex 3-dimensional borylated building blocks.

## Graphical Abstract

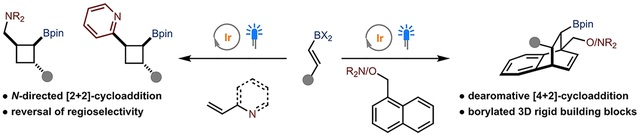

Two methods are presented for the directed cycloaddition of alkenylboronates. In one method, [2+2]-cycloaddition of allylic amines and heterocycles to arrive at diverse cyclobutanes is presented. In a second process, dearomative [4+2]-cycloaddition allows for the synthesis of rigid building blocks from naphthalenes and alkenylboronate. Both processes allow for the construction of complex molecules with high regio and stereocontrol from simple reaction components.

## Introduction

Cyclobutanes are an important motif found in many natural products and biologically relevant molecules.^[1]^ Photochemical [2+2]-cycloadditions are one of the premier methods for the synthesis of cyclobutanes.^[2]^ A common feature of intermolecular variants is the use of activated alkenes.^[3]^ This is, in part, due to the triplet state energy of the photo-activated alkene being too low to engage an unactivated alkene as well as rapid relaxation by bond rotation for acyclic substrates. However, notable exceptions are reactions of cyclic substrates where bond rotation is limited.^[4]^ Utilization of unactivated alkenes for photochemical [2+2] cycloadditions typically rely on the design of substrates that can engage in an intramolecular reaction.^[5]^ The synthesis of intramolecular substrates often require multiple synthetic steps therefore limits the scope of the reaction. Additionally, modification of the products can be challenging, which limits utility.^[6]^

**Scheme 1.**
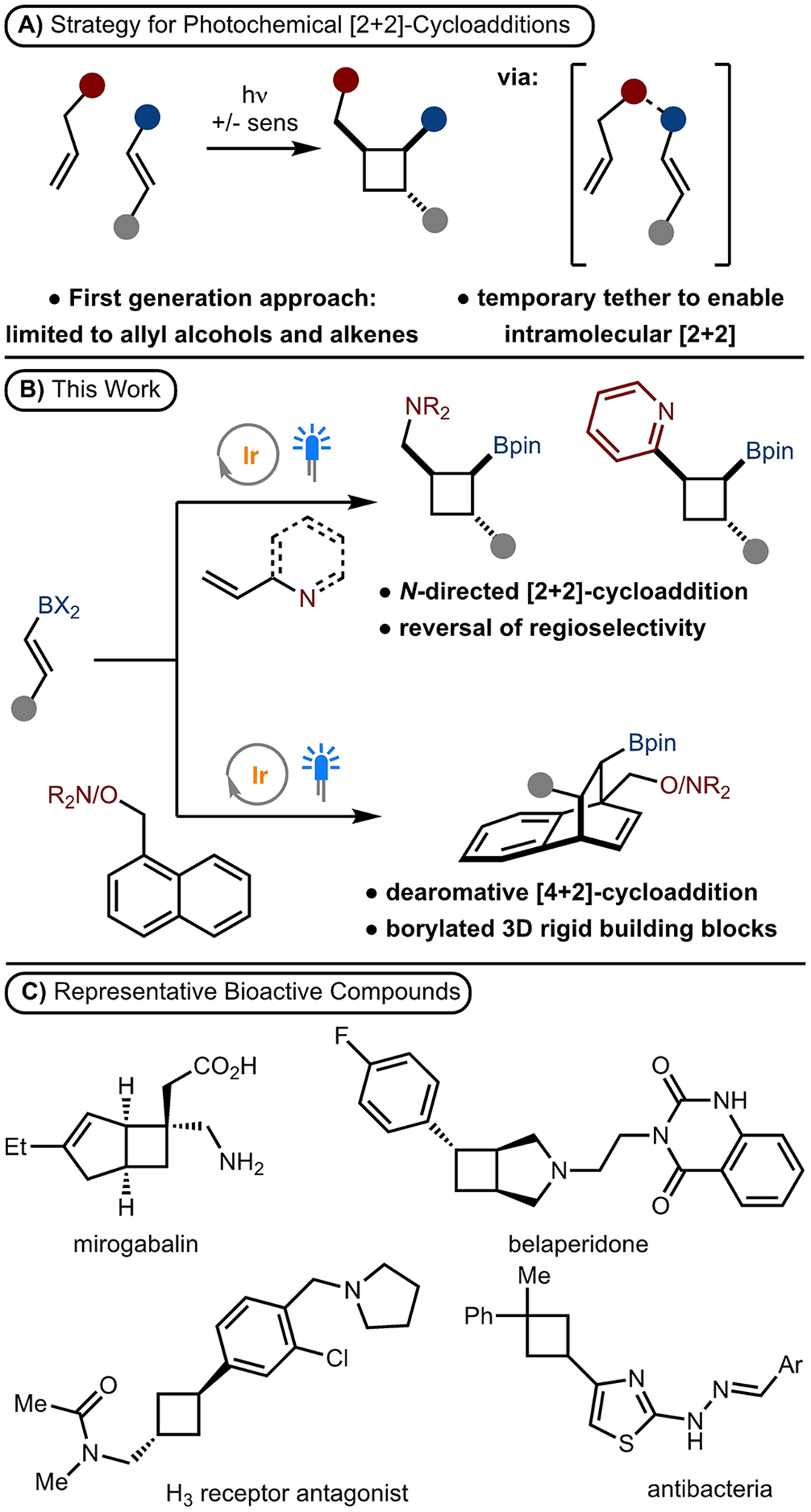
Boron Enabled Cycloaddition.

To address these challenges, we developed a strategy in which a temporary coordination allows for a formal intramolecular cycloaddition to occur, yet results in products that are more similar to that from an intermolecular cycloaddition (Scheme 1A). In our first-generation approach, we developed a photosensitized [2+2]-cycloaddition of allylic alcohols and alkenyl boronic esters.^[7]^ Upon reaction workup, the temporary tether is cleaved to release the product. Despite the utility of this method, especially in natural product synthesis, an allyl alcohol was a necessary component. Engagement of other directing groups such as amines and *N*-heterocycles were unsuccessful. This is a significant objective because amines and *N*-heterocycles are one of the most important motifs present in biologically and pharmaceutically active compounds because they often improve physiological properties such as potency, metabolic stability, membrane permeability as well as protein binding.^[8]^ The challenge with extension to other directing groups, such as amines and N-heterocycles, is due to the unfavorable equilibrium with pinacol boronic esters.

Herein, we describe the successful development of this strategy to allow for cycloaddition of allylic amines (Scheme 1B). In addition, this approach was generalized to heteroaromatic compounds to allow for the synthesis of complex polyarylated cyclobutanes (Scheme 1B). During the course of these investigations, we discovered a dearomative [4+2]-cycloaddition for the synthesis of novel borylated bicycles from simple precursors (Scheme 1B). These reactions are valuable as allylic amines/alkenyl-heteroarenes are widely available, thus allowing for synthesis of diverse products that incorporate a boronic ester functional group.^[9]^ In addition, these products are significant due to the prevalence of amines and heteroaromatic motifs in pharmaceutical agents as shown in Scheme 1C.^[10]^

## Results and Discussion

**Scheme 2.**
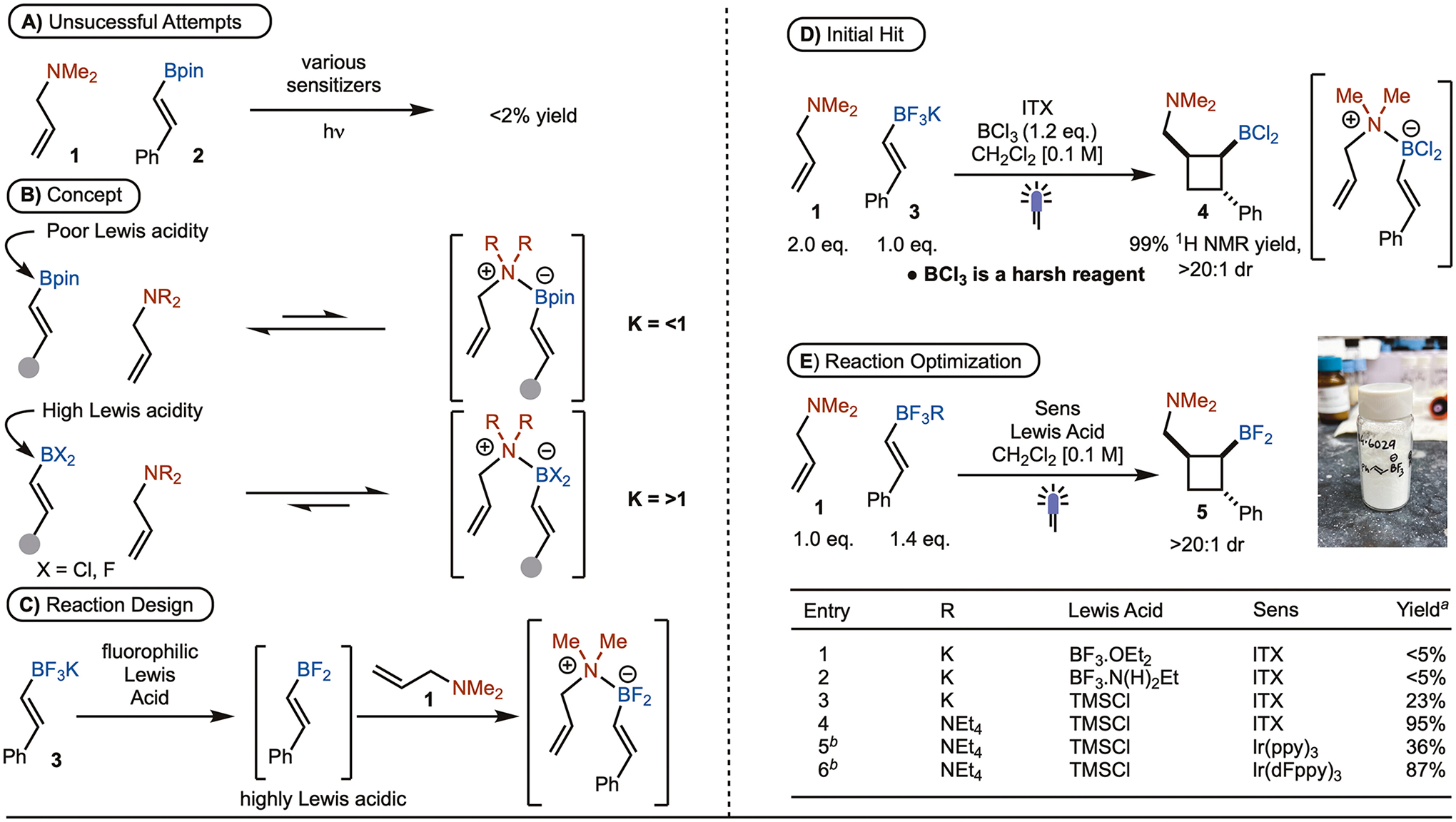
Initial Studies. Reactions run on 0.2 mmol scale with 1.4 eq. BF_3_NEt_4_ salt, 1.0 eq. of amine, 1.6 eq. of Lewis Acid and 10 mol % ITX in CH_2_Cl_2_ [0.1 M] under 395 nm LEDs. (a) ^1^H NMR yield were determined using CH_2_Br_2_ as internal standard. (b) 1 mol % Ir-sensitizer was used under blue LEDs (450 nm).

Under conditions previously reported for alcohol directed [2+2]-cycloadditions, the reaction of allylic amine was not successful (Scheme 2A). It was reasoned that coordination of the amine to the Bpin unit was not strong enough to generate the boronate complex in situ due to electron donation from the oxygen lone pairs (Scheme 2B). To address this challenge, we elected to explore the use of dihaloboranes. Here the increased Lewis acidity should allow for coordination of an amine. However, discrete synthesis of dihaloboranes is challenging. Therefore, we targeted conditions that would generate the dihaloborane in situ by treatment of an alkenyl BF_3_K with a fluorophilic Lewis acid (Scheme 2C).^[11]^

Initial reaction optimization was conducted with *E-*styrenyl BF_3_K **3** and *N*,*N*-dimethylallylamine (**1**) in the presence of BCl_3_ and ITX (*i*-Pr-thioxanthone) as photosensitizer. We were delighted to observe that the desired cyclobutyl boronate product **4** was formed in quantitative yield upon irradiation with 395 nm LEDs (Scheme 2D). However, the use of BCl_3_ would likely limit functional group tolerance as it is a harsh reagent. Therefore, additional fluorophilic Lewis acids were evaluated, which ultimately led to the finding that Me_3_SiCl allowed for formation of **5** in 23% yield (Scheme 2E, entry 3). One of the major issues working with BF_3_K salts is the poor solubility in common organic solvents. To address this issue, reaction with an alkenyl BF_3_NEt_4_ was explored as it should be more soluble.^[12]^ As shown in Scheme 2D, entry 4, the reaction worked in 95% yield. Use of other photosensitzers were also explored (Scheme 2E, entries 5–6). While Ir(ppy)_3_ worked, the use of Ir(dFppy)_3_ was comparable to ITX yet 450 nm LEDs could be used. ITX was selected for substrate evaluation due to its low cost.

While satisfactory yields were observed for the [2+2] cycloadduct, isolation of the difluoroborane (**5**) was challenging by column chromatography. Conversion to the Bpin could be carried out by treatment with pinacol. However, refluxing conditions were required and the corresponding Bpin suffers from instability during purification by silica gel column chromatography. It was ultimately found that addition of Morken’s mac-diol (**6**) rapidly formed the Bmac at ambient temperature and was straightforward to isolate.^[13]^

**Scheme 3.**
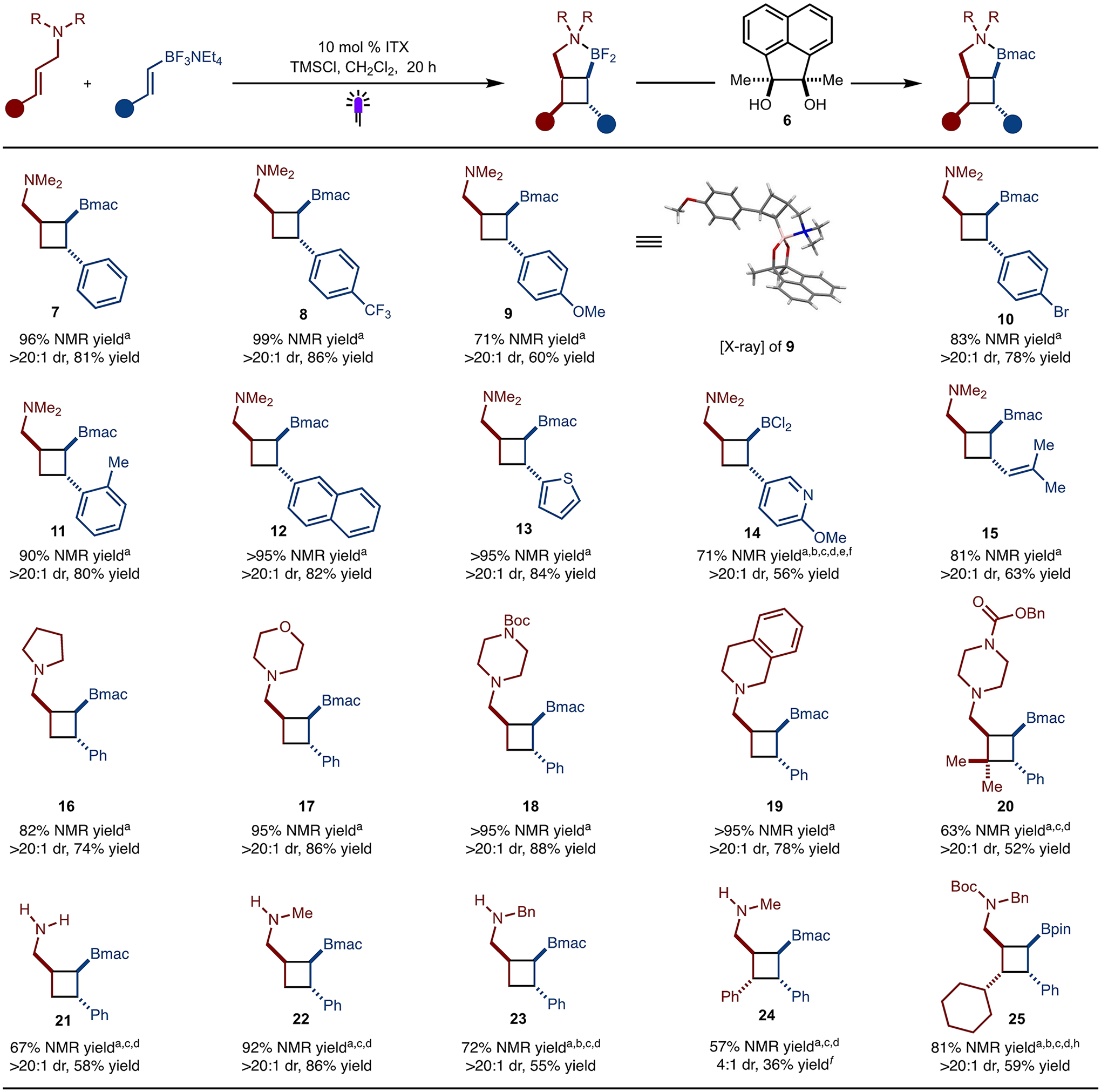
Substrate Scope for Amine Directed [2+2]-Cycloaddition Reactions have been performed in 0.2 mmol scale. ^a^NMR yield refers to yield determined by ^1^H NMR analysis of the unpurified reaction mixture of the product before protection of the BF_2_ unit using CH_2_Br_2_ as internal standard. Diastereomeric ratio (dr) determined of the unpurified reaction mixture by ^1^H NMR analysis. Yield is of isolated, purified product. ^b^BCl_3_ condition was used instead of TMSCl. ^c^Penn PhD Photoreactor M2 was used. ^d^1.0 mol % fac-Ir(dFppy)_3_ was used as photosensitizer under 450 nm LEDs. ^e^BF_3_K salt was used instead of BF_3_NEt_4_ salt. ^f^Isolated yield of major diastereomer. ^g^Isolated as BCl_2_ adduct. ^h^Isolated as Bpin after BOC protection of the amine.

Under the modified workup conditions, several styrenyl BF_3_ salts with different electronic and steric properties were tested (Scheme 3). Electron-withdrawing (product **8**) as well as electron-donating (product **9**) substituents were well tolerated under the standard reaction condition. Sterically demanding ortho-methyl group is also compatible in the reaction to provide product **11** in good yield. Bromide substitution (product **10**), extended conjugation (product **12**), as well as heterocycles like thiophene (product **13**) and pyridine (product **14**) all worked well under the reactions conditions. However, for pyridine substrate, the BF_3_K salt was used instead of BF_3_NEt_4_ salt due to purification issues after ion exchange. In addition to styrenyl-BF_3_NEt_4_ salts, dienyl-BF_3_NEt_4_ salts worked well in the reaction (product **15**).

On the other hand, amines of different ring sizes are also well tolerated in the reaction. For example, pyrolidine (product **16**), morpholine (product **17**), piperazine (product **18**) as well as tetrahydro-isoquinoline (product **19**) derived allyl amines provided the corresponding cycloadducts in excellent yield and diastereoselectivity. However, unsubstituted allylamines (both primary and secondary) failed to show any desired reactivity under the standard reaction conditions. We believe that under the Lewis acidic conditions, ITX undergoes condensation to form an imine with primary and secondary allylamines, resulting in sensitizer poisoning. Based on this hypothesis, when *fac-*Ir(dFppy)_3_ was implemented as photosensitizer, under blue LEDs irradiation, the formation of the corresponding cyclobutane product was observed. However, control experiments revealed that condenstation did not occur with ITX, and therefore the reason for the failure of these reactions is not clear at this time (See the Supporting Information for details). Regardless, primary as well as secondary allylamines are well tolerated under the reaction conditions to provide the products in good yield (product **21, 22**). 1,2 disubstituted substrates such as aryl substituted allyl-amines worked well under the reaction conditions, although the diastereoselectivity was poor (product **24**). However, having a sterically bulkier cyclohexyl group improved the diastereoselectivity (product **25**). Trisubstituted allylamine also worked smoothly to provide product **20**.

**Scheme 4.**
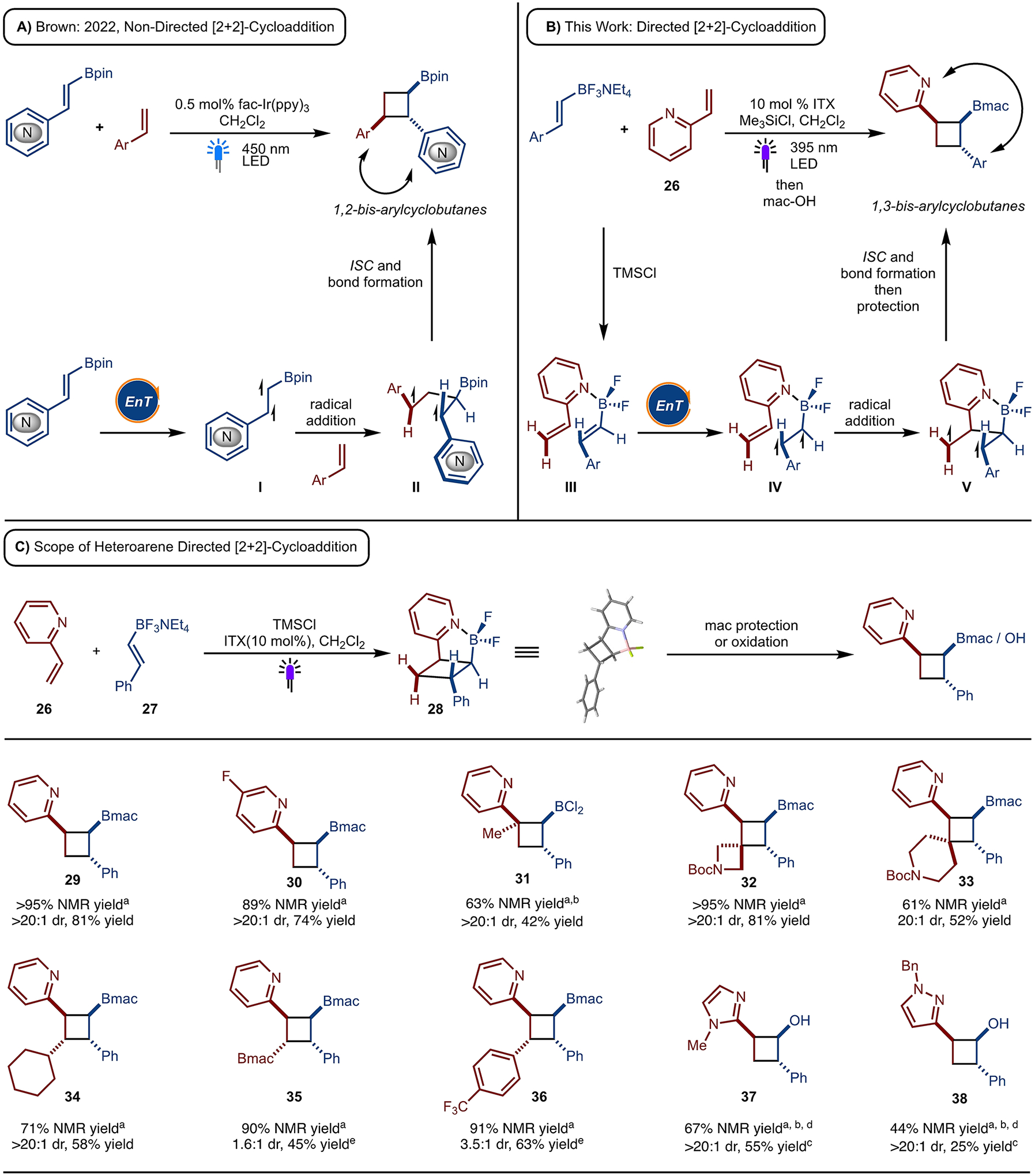
Divergent Reactivity and Substrate Scope. Reactions run on 0.2 mmol scale with 10 mol % ITX in CH_2_Cl_2_ [0.1 M] under 395 nm LEDs. ^a^ NMR yields were determined of unpurified reaction mixture after the cycloadditions reaction using CH_2_Br_2_ as an internal standard. ^b^ Reactions run with BCl_3_ instead of TMSCl ^c^ Product isolated after oxidation of dihaloborane. ^d^ Penn Photoreactor M2 (IPR) was used instead of LEDs. ^e^ Isolated yield for major diastereomer.

The strategy presented herein is of much broader significance beyond aliphatic amines because other Lewis basic functionality can be utilized. Along these lines, reaction of alkenyl-heteroarenes as a directing group was explored. Prior work from our lab demonstrated the cycloaddition of alkenyl boronates and styrenes to generate 1,2-bis-arylcyclobutylboronates as the exclusive product by a non-directed cycloaddition pathway (Scheme 4A).^[3e]^ This is due to the developing formation of highly stable 1,4-bis-benzylic diradical **II** in the transition state. However, directed cycloaddition of an alkenyl-heteroarenes, such as 2-vinyl pyridine, underwent cycloaddition to afford the 1,3-diaryl cyclobutylboronates (Scheme 4B). Thus, the directed approach can change the regioselectivity of the intermolecular cycloaddition. While in the coordinated complex **III** the reaction may proceed by sensitization of the alkenylboronate, it is also possible that the alkenyl-heteroarene may be sensitized to deliver the same product.

A range of 2-vinylpyridines have been examined with different substitution patterns and functional groups (Scheme 4C). Fluorine containing vinyl-pyridine worked well under the reaction condition (product **30**). Di- as well as trisubstituted vinylpyridines have been tested and allowed for synthesis of products **31–36**. Cyclic trisubstituted vinylpyridines provided the corresponding spirocyclic products (product **32** and **33**) with good yield and excellent selectivity. Both alkyl and aryl substituted 1,2-vinyl-pyridines provided the corresponding cyclobutylboronates in good yield, however, poor diastereoselectivity was observed (product **35** and **36**). Bulkier substituents, such as cyclohexyl, results in the formation of a single diastereomer of product **34**. 1,1-Disubstituted vinyl-pyridine was also tolerated, however, BCl_3_ was required for this reaction (product **31**). It is likely that generation of a more Lewis acidic alkenyl BCl_2_ (relative to alkenyl BF_2_) is necessary for the more sterically demanding substrate.^[14]^ Other *N*-heterocycles such as imidazole as well as pyrazole can also act as a directing group to provide the corresponding cyclobutane products in good yield and diastereoselectivity (products **37** and **38)**.

**Scheme 5.**
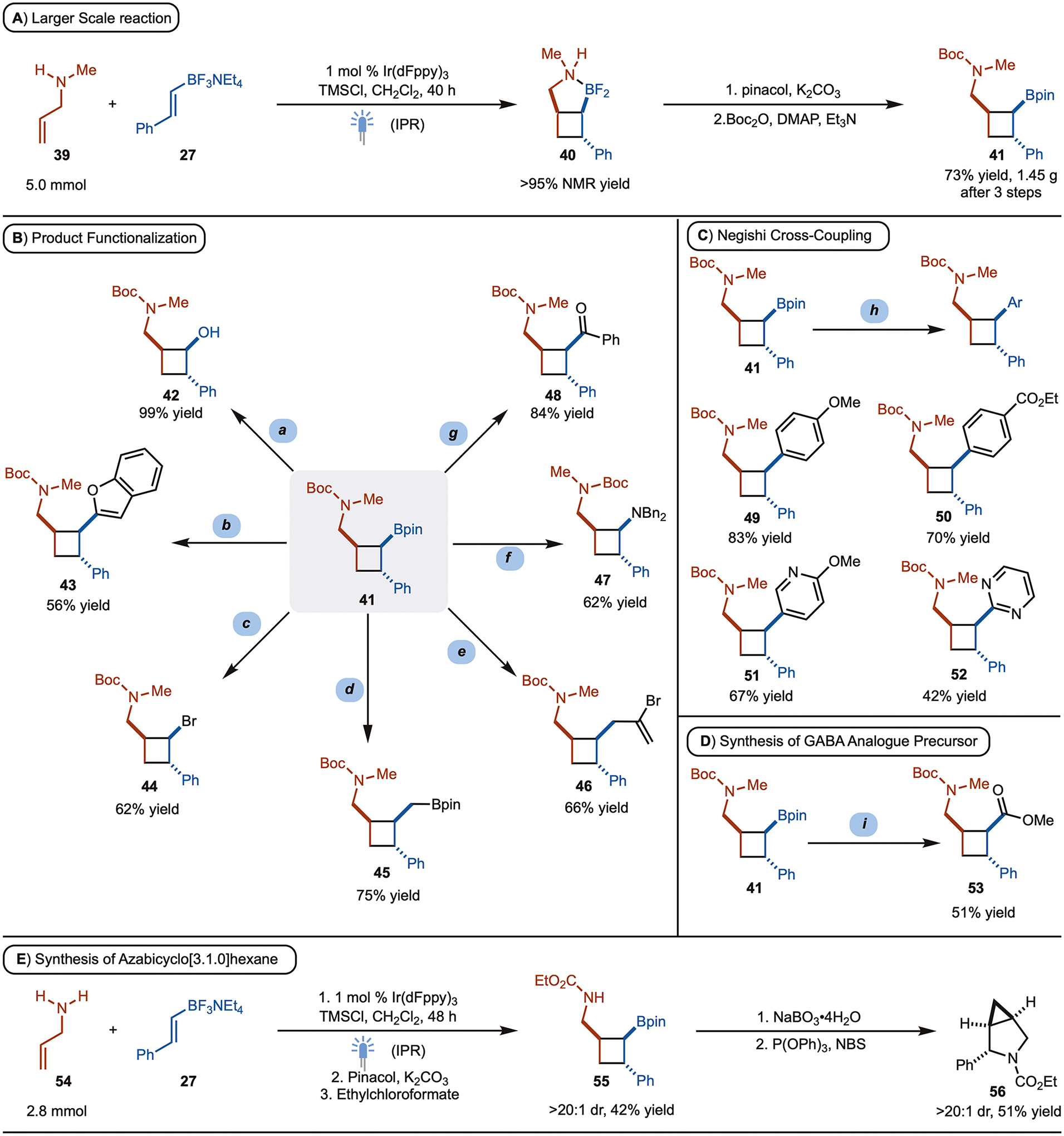
Larger Scale [2+2]-Cycloaddition and Product Diversification. Reaction conditions: (**a**) NaBO_3_•4H_2_O, THF-H_2_O. (**b**) benzofuran, *n*-BuLi, NBS. (**c**) 3,5-Bis-trifuoromethylbromobenzene, *n*-BuLi, NBS. (**d**) *n*-BuLi, CH_2_Br_2_. (**e**) *t*-BuLi, CuCN (cat), 2,3-dibromopropene, styrene.(**f**) *t*-BuLi, CuCN (cat), Bn_2_NOBz, styrene. (**g**) *t*-BuLi, CuCN (cat), BzCl, styrene. (**h**) *t*-BuLi, then Zn(OAc)_2_, then CPhos-PdG3 (cat), ArBr or ArCl, LiCl. (**i**) *t-*BuLi, CuCN (cat), methylchloroformate, styrene.

At this stage, we turned our attention towards the synthetic utility of the products (Scheme 5A). First a larger-scale reaction (5.0 mmol) was performed with *N*-methyl allylamine **39** and the corresponding cycloadduct **40** was generated in quantitative yield by NMR analysis. The difluoro borane cycloadduct was subsequently treated with pinacol and Boc_2_O to provide **41** on gram scale (1.45 g) in 73% isolated yield (Scheme 5A). With **41** in hand, we targeted manipulation of C–B bond to access a diverse class of cyclobutanes (Scheme 5B). First, perborate oxidation of the Bpin unit was performed to access the γ-amino alcohol **42** in 99% yield. Metal-free cross coupling with lithiated benzofuran provided the product **43** in 56% yield.^[15]^ Bromination with NBS from the activated boronate complex enabled the transformation of the C–B bond into a C–Br bond in 62% yield (product **44**).^[16]^ Next, Matteson homologation of the C–B bond was performed to generate the homologated product **45** in 75% yield.^[17]^ The cyclobutyl boronic ester was also converted to a ketone (product **48**), amine (product **47**), and allyl group (product **46**) by Cu-catalyzed coupling.^[18]^

Secondary alkyl boronic esters are challenging substrates for Pd-catalyzed cross coupling reactions. For the products of the [2+2]-cycloaddition, traditional Suzuki–Miyaura reaction conditions failed to provide the desired product. However, Negishi cross-coupling of an in situ generated alkyl zinc was found to be effective towards Csp^3^-Csp^2^ bond formation between cyclobutylboronates **41** and aryl halides.^[19]^ Several examples for the reaction are illustrated in Scheme 5C (product **49–52**). All the reactions proceed smoothly to provide the cross-coupling product with complete retention of stereochemistry. Finally, Cu-catalyzed acylation of cycloadduct **41** also provided access to phenyl substituted γ-aminobutyric acid (GABA) analogue precursor **53** (Scheme 5D).^[20]^

The [2+2] cycloaddition of allylic amine **54** was also carried out in larger scale to provide **55** after sequential treatment with pinacol and ethylchloroformate (Scheme 5E). Perborate oxidation afforded the corresponding alcohol. At this stage, it was desired to carry out a series of substitutions, first with bromide then the pendant carbamate to access an azabicyclo[2.2.0]hexane. However, upon treatment with NBS and P(OPh)_3_, azabicyclo[3.1.0]hexane **56** was generated as a single observable diastereomer. The mechanism for the formation of **56** is not clear at this time.^[21]^

**Scheme 6.**
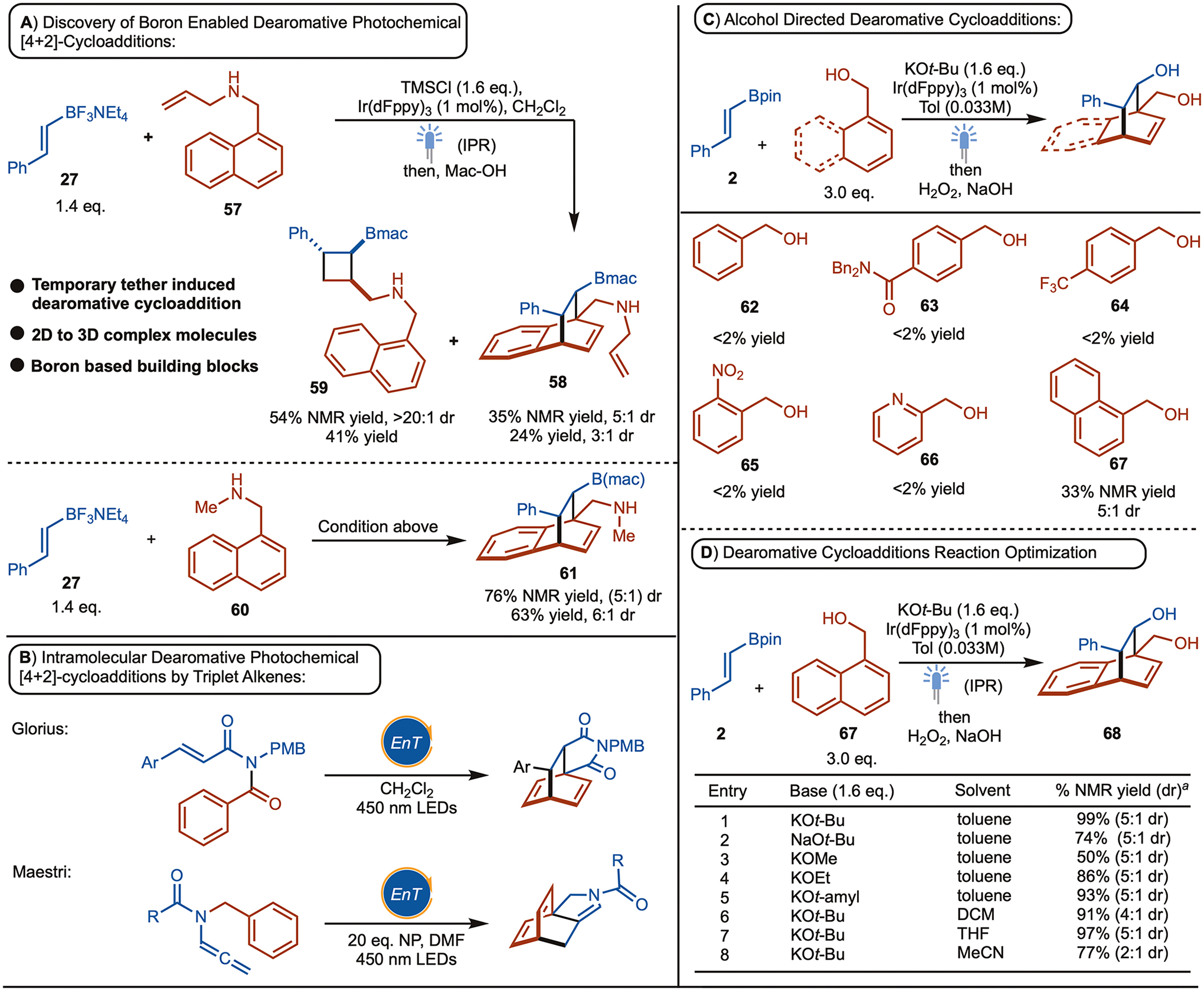
Initial Studies on Boron Enabled Dearomative [4+2]-Cycloaddition. Reactions were performed on 0.2 mmol scale with toluene [0.033 M] under 450 nm LEDs in IPR, ^a 1^H NMR yield were determined using CH_2_Br_2_ as internal standard. 1 mol % Ir-sensitizer was used as photosensitizer.

During evaluation of the substrate scope for the amine directed [2+2]-cycloadditions reactions, allylamine substrate **57** was also tested under standard reaction condition. In this case the expected [2+2]-cycloaddition product **59** was observed along with formation of dearomative [4+2]-cycloaddition product **58** (Scheme 6A). Based on this observation, when substrate **60** was tested, the dearomative [4+2]-cycloaddition product **61** was observed in 76% NMR yield, with 5:1 dr. Dearomative cycloaddition reactions are efficient in terms of building molecular complexity from widely available arenes and alkenes.^[22,23,24]^ This opens the opportunity for accessing new chemical space, which is of high value in medicinal chemistry.^[25,26,27]^ With rare exception, photochemical dearomative [4+2]-cycloaddition involves substrates capable of intramolecular reaction. For example, in 2019, inspired by Kishikawa’s work,^[28]^ Glorius reported an intramolecular reaction where the alkene and arene components are tethered together through amide linker.^[29]^ In addition, in 2022, Maestri reported an intramolecular *para* cycloaddition reaction of (hetero) aromatics (Scheme 6B).^[30]^ On the other hand, in 2021, our group along with the Houk and Glorious group reported the intermolecular para-cycloaddition reactions between quinoline and alkenes.^[31]^ However, these reactions likely proceed by sensitization of the arene followed by stepwise cycloaddition (which is different form that shown in Scheme 6A–B). Subsequently, this reactivity was extended to electron deficient naphthalenes by independent reports from our group and Maji’s lab.^[32,33]^ Recently an enantioselective variant was also reported by You & co-workers.^[34]^

Inspired by our previous boron enabled alkoxide directed [2+2] cycloadditions reactions, we explored reactions of benzyl alcohols with *E*-styrenylBpin **2** (Scheme 6C). Unfortunately, <2% dearomative cycloadduct was observed with benzyl alcohol or substituted variants (**62–65**). Pyridine-1-methanol (**66**) was also tested but remained unsuccessful. However, when naphthyl-1-methanol (**67**) was used, the desired product **68** was generated in 33% NMR yield, and 5:1 dr. Using blue LED strips, we found that even after 24 hours incomplete conversion of the starting material was observed. Therefore, we elected to use an Integrated Photo-Reactor (IPR), which supplies significantly higher flux and observed a significant increase in yield (Scheme 6D, entry 1).^[35]^ Other alkoxide bases were investigated with KO*t*-Bu being optimal (Scheme 6D, entries 2–5). In addition, while other solvents are competent, toluene was found to be ideal (Scheme 6D, entries 6–8). While amines and alcohols can both be used to direct the cycloaddition, we elected to use naphthyl methanol because it is commercial available, whereas the corresponding amines need to be prepared from naphthaldehyde by reductive amination.

**Scheme 7.**
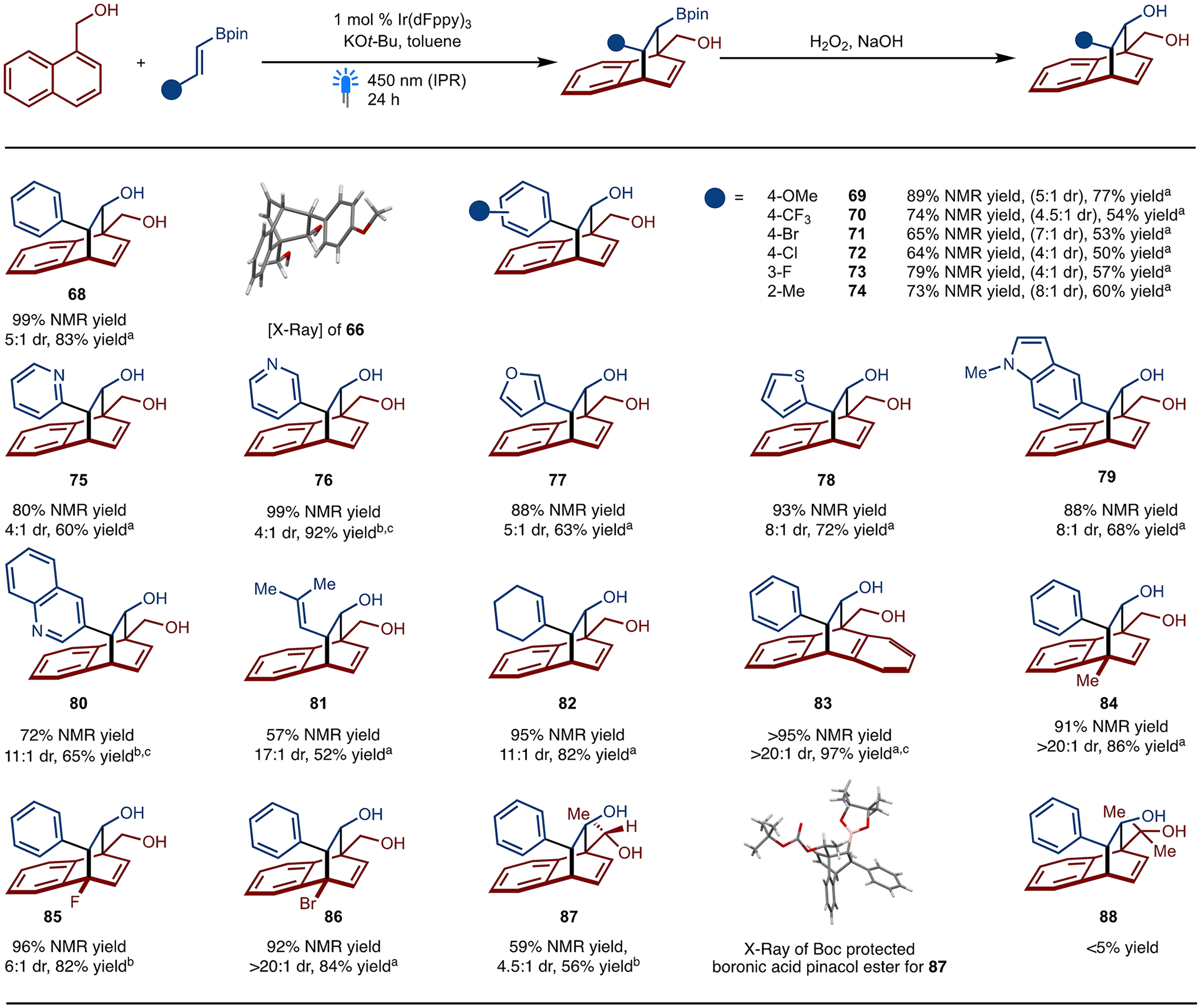
Substrate Scope of Dearomative [4+2]-Cycloaddition. NMR yield refers to yield determined by ^1^H NMR analysis of the unpurified reaction mixture of the product after oxidation of the Bpin unit. Diastereomeric ratio (dr) determined of the unpurified reaction mixture by ^1^H NMR analysis. (a) isolated Yield of major diastereomer. (b) Isolated yield of mixture of diastereomers. (c) THF was used as solvent.

Under the optimal conditions, a series of substituted styrenyl-Bpin substrates with different electronic and steric properties were tested (Scheme 7). Substrates with electron donating methoxy substitution at the *para* position (product **69**) as well as an electron withdrawing trifluoromethyl group at the *para* position (product **70**) worked well in the reaction. Halogen substitution, such as 4-bromo (product **71**), 4-chloro (product **72**) and 3-fluoro (product **73**) are also well tolerated under the standard reaction condition. Sterically demanding *ortho*-methyl (product **74**) substituted styrenyl-Bpins also provided the *para*-cycloadduct with good yield and diastereoselectivity. Heterocyclic compounds like 2-pyridine (product **75**), 3-pyridine (product **76**), furan (product **77**), thiophene (product **78**), quinoline (product **80**) as well as indole (product **79**) all worked well. In addition to Bpin substituted alkenylarenes, Bpin substituted dienes (product **81** and **82**) functioned well. After examining the scope for alkenyl-Bpin substrates, 4-Substitued naphthyl-methanol such as methyl (product **84**), fluoro (product **85**), and bromo (product **86**) groups were also tolerated.

While a secondary alcohol was able to provide the cycloadduct in moderate yield and diastereoselectivity (product **87**), the reaction does not tolerate tertiary alcohols (product **88**). Bulkier substituents (bromide and methyl) generally provided high diastereoselectivity for the cycloadducts. Anthracene methanol (product **83**) also works in the reaction with excellent yield and diastereoselectivity.

**Scheme 8.**
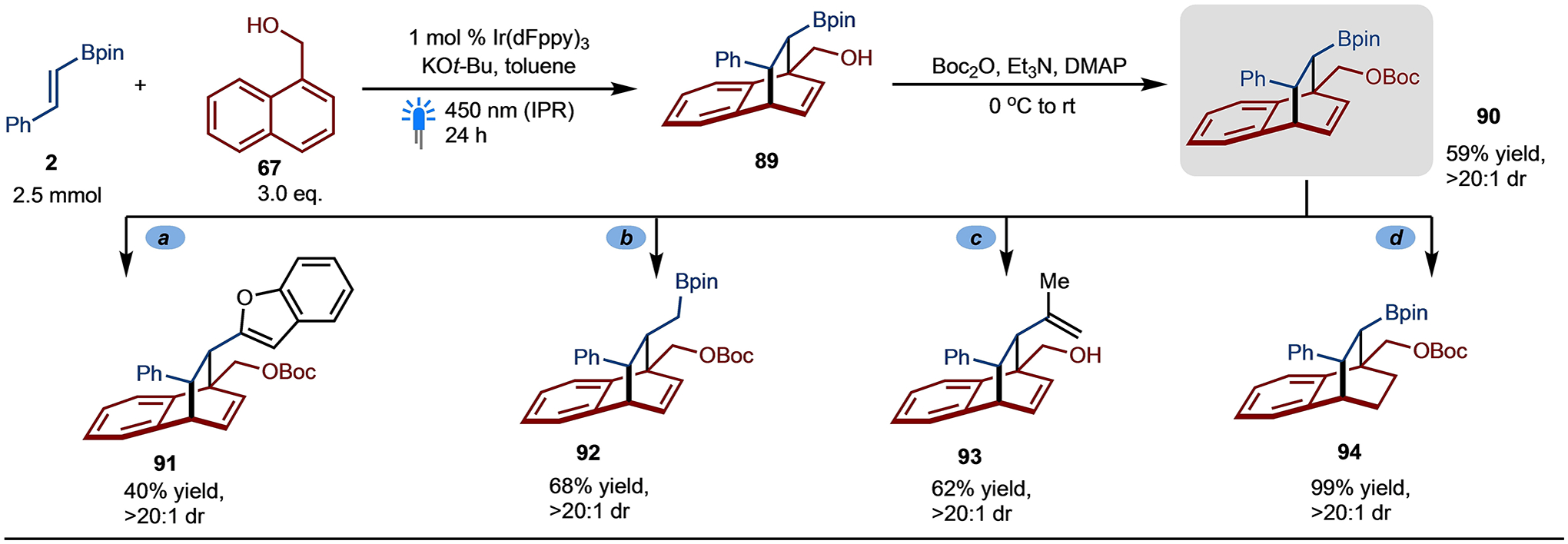
Larger scale [4+2]-Cycloaddition and Product Diversifications. Reaction conditions: (**a**) benzofuran, *n*-BuLi, NBS. (**b**) *n*-BuLi, CH_2_Br_2_. (**c**) 2-bromopropene, *t*-BuLi, I_2_. (**d**) H_2_, Pd–C, MeOH.

The dearomative [4+2]-cycloaddition reaction has been also carried out on larger scale and the major diasteromer of the cycloadduct **90** has been isolated in 59% yield after Boc protection (Scheme 8). Taking advantage of the C–B bond, several modifications of the borylated cycloadduct have been performed.^[36]^ Metal-free cross-coupling to introduce benzofuran proceeded in moderate yield to provide the corresponding product **91**.^[15]^ Matteson homologation (product **92**) as well as Zweifel olefination^[37]^ (product **93**) also worked well. Reduction of the olefin provided **94** in quantitative yield.

## Conclusion

In conclusion, a protocol for photochemical [2+2] cycloadditions between styrenyl dihaloboranes and unactivated allylamines has been developed to synthesize substituted cyclobutylboronates with high regio and diastereocontrol. Optimization of counter cation for the alkenyl BF_3_ was as a key factor towards the success of the reaction under mild conditions. Additionally, cyclobutanes with alternative substitution patterns have been realized by using *N*-heterocycles as a directing group. These cyclobutane products are valuable because amines and *N*-heterocycles are common in small molecule therapeutics. The cycloaddition reaction has been performed on gram scale and functionalization of the C–B bond allows for diverse product formation. Finally, a dearomative [4+2]-cycloaddition between alkenyl boronic esters and naphthyl methanols to synthesize complex 3D borylated bicyclic motifs with high selectivity was demonstrated.

## Supplementary Material

Supporting Info

## Data Availability

The data that support the findings of this study are available in the supplementary material of this article.

## References

[1] a) For selected reviews on cyclobutanes: Erin JM Wahl M. K. Brown, Nat. Prod. Rep 2019, 36, 1383–1393; 30855044 10.1039/c8np00083b PMC6739199

[2] For selected reviews see: a) Poplata S, Tröster A, Zou Y-Q, Bach T, Chem. Rev 2016, 116, 9748–9815; 27018601 10.1021/acs.chemrev.5b00723 PMC5025837

[3] For examples see: a) Murray PRD, Bussink WMM, Davies GHM, van der Mei FW, Antropow AH, Edwards JT, D’Agostino LA, Ellis JM, Hamann LG, Romanov-Michailidis F, Knowles RR, J. Am. Chem. Soc 2021, 143, 4055–4063; 33666086 10.1021/jacs.1c01173

[4] For example, see: a) Poplata S, Bach T, J. Am. Chem. Soc 2018, 140, 3228–3231; 29458250 10.1021/jacs.8b01011 PMC5849358

[5] For example, see: a) Bach T, Synthesis 1998, 1998, 683–703;

[6] a) Großkopf J, Kratz T, Rigotti T, Bach T, Chem. Rev 2022, 122, 1626–1653; 34227803 10.1021/acs.chemrev.1c00272

[7] Liu Y, Ni D, Brown MK, J. Am. Chem. Soc 2022, 144, 18790–18796. 36200833 10.1021/jacs.2c08777 PMC9832331

[8] a) Walsh CT, Tetrahedron Lett 2015, 56, 3075–3081;

[9] a) van der Kolk MR, Janssen MACH, Rutjes FPJT, Blanco-Ania D, ChemMedChem 2022, 17, e202200020; 35263505 10.1002/cmdc.202200020 PMC9314592

[10] a) Vitaku E, Smith DT, Njardarson JT, J. Med. Chem 2014, 57, 10257–10274; 25255204 10.1021/jm501100b

[11] a) Vedejs E, Chapman RW, Fields SC, Lin S, Schrimpf MR, J. Org. Chem 1995, 60, 3020–3027;

[12] a) Batey RA, Quach TD, Tetrahedron Lett 2001, 42, 9099–9103;

[13] Zhang C, Hu W, Lovinger GJ, Jin J, Chen J, Morken JP, J. Am. Chem. Soc 2021, 143, 14189–14195. 34425672 10.1021/jacs.1c05274 PMC8859859

[14] Jones V, Frenking G, Reetz MT, J. Am. Chem. Soc 1994, 116, 8741–8753.

[15] Bonet A, Odachowski M, Leonori D, Essafi S, Aggarwal VK, Nat. Chem 2014, 6, 584–589. 24950327 10.1038/nchem.1971

[16] Larouche-Gauthier R, Elford TG, Aggarwal VK, J. Am. Chem. Soc 2011, 133, 16794–16797. 21939203 10.1021/ja2077813

[17] Matteson DS, Mah RWH, J. Am. Chem. Soc 1963, 85, 2599–2603.

[18] Xu N, Liang H, Morken JP, J. Am. Chem. Soc 2022, 144, 11546–11552. 35735669 10.1021/jacs.2c04037 PMC10436227

[19] Liang H, Morken JP, J. Am. Chem. Soc 2023, 145, 9976–9981. 37126565 10.1021/jacs.3c01677 PMC10407644

[20] a) Baxendale IR, Ernst M, Krahnert W-R, Ley SV, Synlett 2002, 2002, 1641–1644;

[21] For possible mechanisms for formation of **56**, see the SI.

[22] Huck CJ, Sarlah D, Chem 2020, 6, 1589–1603. 32715154 10.1016/j.chempr.2020.06.015 PMC7380651

[23] Okumura M, Sarlah D, Eur. J. Org. Chem 2020, 2020, 1259–1273. 10.1002/ejoc.201901229 PMC7250370 32457562

[24] Cheng Y-Z, Feng Z, Zhang X, You S-L, Chem. Soc. Rev 2022, 51, 2145–2170. 35212320 10.1039/c9cs00311h

[25] Wei W, Cherukupalli S, Jing L, Liu X, Zhan P, Drug Discovery Today 2020, 25, 1839–1845. 32712310 10.1016/j.drudis.2020.07.017

[26] Lovering F, MedChemComm 2013, 4, 515–519.

[27] Lovering F, Bikker J, Humblet C, J. Med. Chem 2009, 52, 6752–6756. 19827778 10.1021/jm901241e

[28] Kishikawa K, Akimoto S, Kohmoto S, Yamamoto M, Yamada K, J. Chem. Soc. Perkin Trans 1 1997, 77–84.

[29] Ma J, Strieth-Kalthoff F, Dalton T, Freitag M, Schwarz JL, Bergander K, Daniliuc C, Glorius F, Chem 2019, 5, 2854–2864.

[30] Chiminelli M, Serafino A, Ruggeri D, Marchiò L, Bigi F, Maggi R, Malacria M, Maestri G, Angew. Chem. Int. Ed 2023, 62, e202216817. 10.1002/anie.202216817 36705630

[31] a) Ma J, Chen S, Bellotti P, Guo R, Schäfer F, Heusler A, Zhang X, Daniliuc C, Brown MK, Houk KN, Glorius F, Science 2021, 371, 1338–1345. 33766881 10.1126/science.abg0720 PMC7610643

[32] Wang W, Cai Y, Guo R, Brown MK, Chem. Sci 2022, 13, 13582–13587. 36507189 10.1039/d2sc04789f PMC9682912

[33] Rai P, Maji K, Jana SK, Maji B, Chem. Sci 2022, 13, 12503–12510. 36349268 10.1039/d2sc04005k PMC9628934

[34] Li M, Huang X-L, Zhang Z-Y, Wang Z, Wu Z, Yang H, Shen W-J, Cheng Y-Z, You S-L, J. Am. Chem. Soc 2024, 146, 16982–16989. 38870424 10.1021/jacs.4c05288

[35] Le CC, Wismer MK, Shi Z-C, Zhang R, Conway DV, Li G, Vachal P, Davies IW, MacMillan DWC, ACS Cent. Sci 2017, 3, 647–653. 28691077 10.1021/acscentsci.7b00159 PMC5492256

[36] Sandford C, Aggarwal VK, Chem. Commun 2017, 53, 5481–5494. 10.1039/c7cc01254c 28397919

[37] Zweifel G, Arzoumanian H, Whitney CC, J. Am. Chem. Soc 1967, 89, 3652–3653.

