# Supplementary material for: Boron Enabled Directed [2+2]- and Dearomative [4+2]-Cycloadditions Initiated by Energy Transfer
Source: Angew Chem Int Ed Engl. Author manuscript; Available in PMC 2026 Jan 21. (PMC11753935; doi:10.1002/anie.202416215)
Supplement: Supporting Info [file NIHMS2046750-supplement-Supporting_Info.pdf]

Angewandte Chemie
Eine Zeitschrift der Gesellschaft Deutscher Chemiker

Supporting Information

Boron Enabled Directed [2+2]- and Dearomative [4+2]-Cycloadditions Initiated by Energy Transfer

*S. Adak, P. S. Hazra, C. B. Fox, M. K. Brown**

Boron Enabled Directed [2+2] and Dearomative [4+2] Cycloadditions initiated by Energy Transfer

Souvik Adak, Partha S. Hazra, Carter Fox and M. Kevin Brown*

Indiana University, Department of Chemistry
800 E. Kirkwood Avenue, Bloomington, IN 47405

Supplementary Information – Table of Contents

General……………………………………………………………………S02

Reagents and Catalysts ……………………………………………………S03

Reaction monitor progress for unsuccessful reaction………………………S06

Optimization Study…………………………………………………………S07

Experimental Procedures……………………………………………………S11

X-Ray Data…………………………………………………………………S74

Spectra………………………………………………………………………S85

References…………………………………………………………………S190

1. General:

NMR: ^{1}H NMR spectra were recorded at room temperature on a Varian I400 (400 MHz), Varian VXR400 (400 MHz), Varian I500 (500 MHz), Bruker 500 (500 MHz) or a Varian I600 (600 MHz) spectrometer. Chemical shifts are reported in ppm from tetramethylsilane with the residual solvent resonance as the internal standard [$CDCl_3$: 7.26 ppm, CD_3CN: 1.94 ppm, quin (*J* = 2.5 Hz), 2.13 ppm, s, CD_3OD: 3.31 ppm, quin (*J* = 1.7 Hz), 4.87 ppm, s, DMSO-d^6: 2.50 ppm, quin, (*J* = 1.9 Hz), s, 3.33 ppm] Data are reported as follows: chemical shift, multiplicity (s = singlet, d = doublet, t = triplet, q = quartet, quin = quintet, br = broad, m = multiplet), coupling constants (Hz), and integration. ^{13}C NMR spectra were recorded on a Varian I400 (101 MHz), Varian I500 (126 MHz) or Bruker 500 (126 MHz) spectrometer with complete proton decoupling. Chemical shifts are reported in ppm from tetramethylsilane with the solvent resonance as the internal standard ($CDCl_3$: 77.16 ppm, CD_3CN: 118.3, 1.3, CD_3OD: 49.0, DMSO-d^6: 39.5). Note: In case of boron containing compounds, carbon attached directly to boron is not observed in ^{13}C NMR due to quadrupolar relaxation.

IR: Infrared (IR) spectra were recorded on a Bruker Tensor II FT-IR Spectrometer, ν_{max} in cm^{-1}. Bands are characterized as broad (br), strong (s), medium (m), and weak (w).

HRMS: High Resolution Mass Spectrometry (HRMS) analysis was obtained using Electrospray Ionization (ESI) and reported as m/z (relative intensity). ESI was acquired using a Waters/Micromass LCT Classic (ESI-TOF).

Solvents: Dichloromethane/ CH_2Cl_2 (DCM), Tetrahydrofuran (THF), Diethyl ether (Et_2O), dioxane and Dimethylformamide (DMF) were purified under a positive pressure of dry argon by passage through two columns of activated alumina. Toluene (PhMe) was purified under a positive pressure of dry argon by passage through columns of activated alumina and Q5 (Grubbs apparatus).

Reactions: Unless otherwise noted, all reactions have been carried out with distilled and degassed solvents under an atmosphere of dry N_2 in oven- (150 °C) and flame-dried glassware with standard vacuum-line techniques. All work-up and purification procedures were carried out with reagent grade solvents in air. Standard flash column chromatography (FCC) techniques using ZEOprep 60/40-63 µm silica gel were used for purification

Light source: Double-density sapphire LEDs strip was purchased from Creative Lighting Solutions. [1]The strip was wrapped around a 125 × 65 mm crystallization dish 4-5 times. Reactions was generally placed ~1 cm away from the lights (the walls of the dish), by placing them in a vial holder. For setup, see the picture from previous literature.[2] Penn Photoreactor M2 was used in some cases as light source.[3]

2. Reagents and Catalysts:

E-Phenylethynylboronic acid was purchased from Combi Blocks and used as received.

KHF_2 was purchased from Oakwood Chemicals and used as received.

Tetraethylammonium hydroxide (35% in water) was purchased from Oakwood Chemicals and used as received.

2-Isopropylthioxanthone (ITX) was purchased from Combi Blocks and used as received.

Allyl Bromide was purchased from Oakwood Chemicals and used as received.

2-Vinylpyridine was purchased from TCI and used as received.

Trimethylaluminium was purchased from Aldrich and used as received.

Acenapthalene-1, 2,-dione was purchased from Ambeed and used as received.

Mac-OH was prepared according to literature procedure.[4]

BCl_3 (1M in DCM) was purchased from Sigma and used as received.

TMSCl was purchased from oakwood and distilled over CaH_2 prior to use.

Pinacol was purchased from oakwood and used as received.

***n*-BuLi** (2.5 M in hexane) was purchased from sigma and titrated prior to use.

***t*-BuLi** (1.7 M in pentane) was purchased from sigma and titrated prior to use.

1-bromo-3-methylbut-2-ene was purchased from TCI and used as received.

benzyl piperazine-1-carboxylate was purchased from TCI and used as received.

1-naphthaldehyde was purchased from Sigma Aldrich and used as received.

prop-2-en-1-amine was purchased from sigma and distilled over CaH_2 prior to use.

Sodium cyanoborohydride ($NaBH_3CN$) was purchased from oakwood and used as received.

$MeNH_2$ (1M in MeOH) was purchased from Sigma Aldrich and used as received.

(Bromomethyl)triphenylphosphonium bromide was purchased from Ambeed and used as received.

Phosphonium, triphenyl(2-pyridinylmethyl)-, chloride (1:1) was prepared according to literature procedure. [5]

1-(pyridin-2-yl)ethan-1-one was purchased from Ambeed and used as received.

1-methyl-1H-imidazole-2-carbaldehyde purchased from AK Scientific and used as received.

4-(trifluoromethyl)benzaldehyde was purchased from oakwood and used as received.

5-fluoropicolinaldehyde was purchased from Combi-blocks and used as received.

tert-butyl 3-oxoazetidine-1-carboxylate was purchased from Ambeed and used as received.

tert-butyl 4-oxopiperidine-1-carboxylate was purchased from Ambeed and used as received.

Isobutyl Chloroformate was purchased from oakwood and used as received.
N, N-diisopropyl ethylamine (DIPEA) was purchased from sigma and distilled over CaH_2 prior to use.
Imidazole was purchased from oakwood and used as received.
K_2CO_3 (anhyd.) Was purchased from Fischer Scientific and used as received.
Pyrrolidine was purchased from oakwood and used as received.
Morpholine was purchased from oakwood and used as received.
1-Boc piperazine was purchased from oakwood and used as received.
1,2,3,4-tetrahydroisoquinoline was purchased from Sigma Aldrich and used as received.
methyl (prop-2-en-1-yl) amine was purchased from oakwood and distilled over CaH_2 prior to use.
Cinnamyl alcohol was purchased from Sigma Aldrich and used as received.
Benzylamine was purchased from Sigma Aldrich and used as received.
Di-tertbuty-dicarbonate (Boc_2O) was was purchased from Oakwood and used as received.
Sodium perborate tetrahydrate was purchased from Sigma Aldrich and used as received.
Benzofuran was purchased from Combiblocks and used as received.
NBS was was purchased from oakwood and recrystallized prior to use.
3, 5-bis(trifluromethyl)bromobenzene was was purchased from oakwood and used as received.
2,3-Dibromopropene was purchased from Sigma Aldrich and used as received.
Copper(I) cyanide was purchased from Sigma Aldrich and used as received.
Styrene was purchased from Sigma Aldrich and purified by a small silica plug before use.
Cesium Fluride was purchased from Sigma Aldrich and used as received.
Benzoyl Chloride was purchased from Sigma Aldrich and purified by distillation before use.
$Zn(OAc)_2$ was purchased from Sigma Aldrich and used as received.
CPhos was purchased from Strem and used as received.
$[PdG_3]_2$ Was prepared according to the literature procedure.[6]
LiCl was purchased from Sigma Aldrich and used as received.
4-bromoanisole was purchased from TCI and used as received.
Ethyl 4-bromobenzoate was purchased from Oakwood and used as received.
5-Bromo-2methoxypyridine was purchased from Oakwood and used as received.
2-Chloropyrimidine was purchased from Sigma Aldrich and used as received.
Methyl chloroformate was purchased from Sigma Aldrich and purified by distillation before use.
DMAP was purchased from Oakwood and used as received.
2-bromopropene was purchased from Oakwood and used as received.
I_2 was purchased from Alfa Aesar and used as received.
10 % Pd-C was purchased from Sigma Aldrich and used as recieved.
Potassium *tert*-butoxide was purchased from Strem and used as received.

Sodium borohydride ($NaBH_4$) was purchased from Sigma Aldrich and used as received**.**

Naphthalen-1-ylmethanol was purchased from Ambeed and used as received.

Ir(dFppy)$_3$ was prepared according to the literature procedure.[7]

1-(naphthalen-1-yl)ethan-1-ol was purchased from Ambeed and used as received.

(4-methylnaphthalen-1-yl)methanol was purchased from Ambeed and used as received.

4-fluoro-1-naphthaldehyde was purchased from Combi Blocks and used as received**.**

4-bromo-1-naphthoic acid was purchased from Ambeed and used as received.

Anthracen-9-ylmethanol was purchased from Sigma Aldrich and used as received.

3. Reaction progress monitoring of unsuccessful [2+2] reaction between BF3 salt 27 and prop-2-en-1-amine in presence of ITX:

3a. NMR experiment:

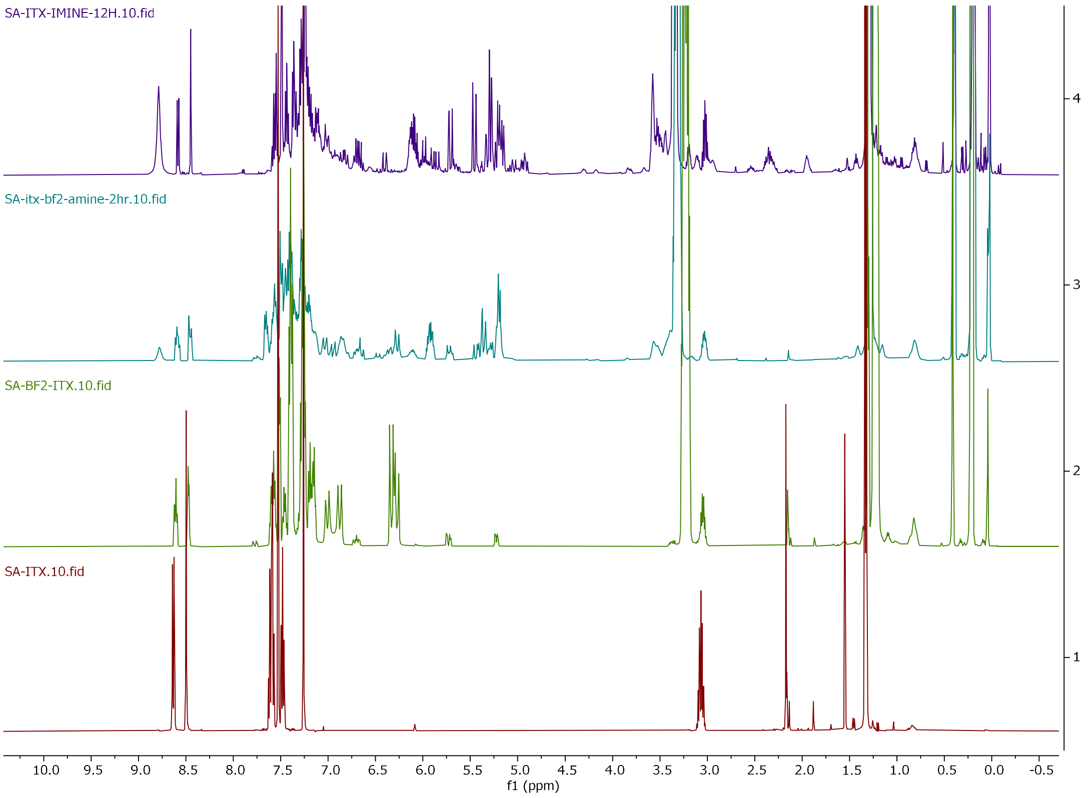

1. --- ITX only
2. --- ITX (50 mol %) + Styrenyl BF_3NEt_4 27 (1.4 eq.) + TMSCl (1.6 eq.)
3. --- ITX (50 mol %) + Styrenyl BF_3NEt_4 27 (1.4 eq.) + TMSCl (1.6 eq.) + prop-2-en-1-amine (1.0 eq.) [stir together for 2h at rt]
4. --- ITX (50 mol %) + Styrenyl BF_3NEt_4 27 (1.4 eq.) + TMSCl (1.6 eq.) + prop-2-en-1-amine (1.0 eq.) [stir together for 12h at rt under 395 nm LED strips]

Comments:

Based on the crude NMR analysis, ITX was still observed in the reaction mixture even after the LED irradiation. This data suggests complete poisoning of ITX due to the condensation with prop-2-en-1-amine is probably not the major reason behind the failure of the directed [2+2] cycloadditions reaction with free amines.

3b. UV-Vis experiment:

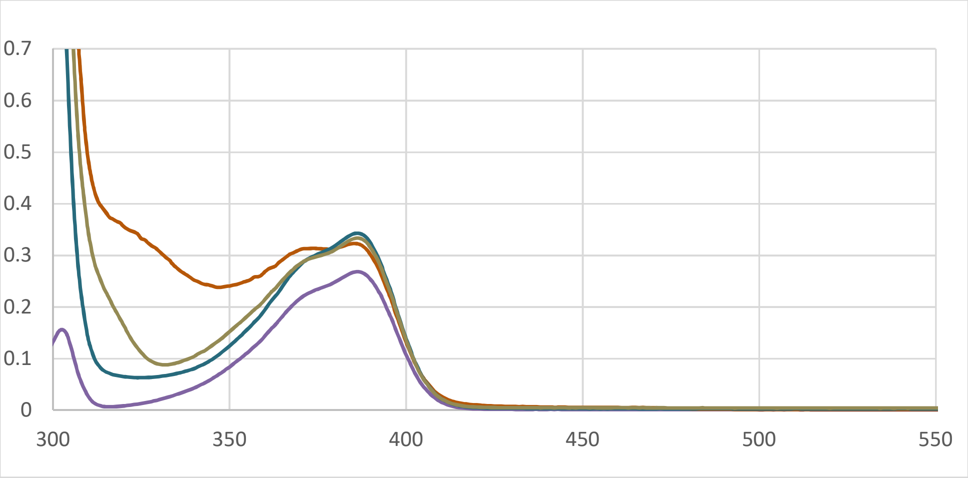

1. ----- ITX only
2. ----- ITX (10 mol %) + Styrenyl BF_3NEt_4 27 (1.4 eq.) + TMSCl (1.6 eq.)
3. ----- ITX (10 mol %) + Styrenyl BF_3NEt_4 27 (1.4 eq.) + TMSCl (1.6 eq.) + N, N-dimethyl prop-2-en-1-amine (1.0 eq.) [stir together for 1h at rt]
4. ----- ITX (10 mol %) + Styrenyl BF_3NEt_4 27 (1.4 eq.) + TMSCl (1.6 eq.) + prop-2-en-1-amine (1.0 eq.) [stir together for 1h at rt]

Comments:

No significant changes observed in the UV-Vis absorption spectra under the reaction condition in prescence of N, N-dimethylprop-2-en-1-amine compared to that in presence of prop-2-en-1-amine.

4. Optimization of the Reaction Condition:

4.1 Optimization of Photochemical [2+2] Cycloadditions Reactions:

4.1a Reaction with BCl_3:

Procedure:

In a 2 dram vial with a stir bar inside was charged with ITX (5.5 mg, 0.020 mmol, 0.10 equiv.) and *E*-StyrenylBF_3K salt **3** (42.2 mg, 0.200 mmol, 1.00 equiv.). The reaction vial was capped with a rubber septum and degasses and backfilled with N_2 (X 3). 2 mL of anhydrous DCM was added to it via a syringe under N_2 atmosphere. N,N-dimethylallylamine **1** (47 μL, 0.40 mmol, 2.0 equiv.) was added slowly to the reaction mixture via a syringe. After that a solution of BCl_3 (1M solution in DCM) was added to the reaction mixture dropwise under inert atmosphere. The rubber septum was quickly replaced by a plastic cap and the reaction vial was sealed with a Teflon tape. The reaction vial was allowed to stir under 395 nm LEDs irradiation at rt for 16 h. After the reaction period, all the volatiles were removed in vacuo and the crude mixture was taken for ^{1}H-NMR analysis using CH_2Br_2 as internal standard.

Note: For inverse addition, ITX (5.5 mg, 0.02 mmol, 0.10 equiv.) and *E*-StyrenylBF_3K salt (42.2 mg, 0.200 mmol, 1.00 equiv.) were taken in a dram vial with a stir bar inside. After degassing and backfilling with N_2 (X 3), under N_2 atmosphere, 2 mL of anhydrous DCM was added. After that a solution of of BCl_3 (1M solution in DCM) was added to

the reaction mixture dropwise under inert atmosphere and the mixture was allowed to stir for 10 minutes at rt. After that N,N-dimethylallylamine (47 μL, 0.40 mmol, 2.0 equiv.) was added slowly to the reaction mixture via a syringe. The rubber septum was quickly replaced by a plastic cap and the reaction vial was sealed with a Teflon tape. The reaction vial was allowed to stir under 395 nm LEDs irradiation at rt for 16 h. After the reaction time period, all the volatiles were removed in vacuo and the crude mixture was taken for ^{1}H-NMR analysis using CH_2Br_2 as internal standard.

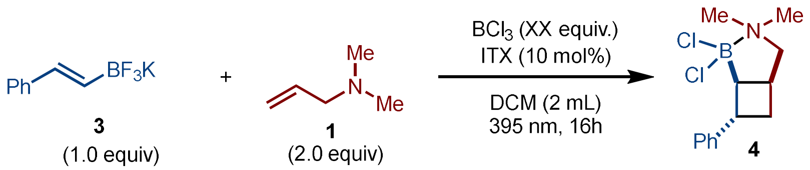

Entry	BCl_3(equiv)	NMR yield of XX
1.	0.15 equiv.	n.d.
2.	0.15 equiv.	14%[a]
3.	1.20 equiv.	>95%[a]

reaction were performed in 0.2 mmol scale, NMR yield were determined using CH_2Br_2 as internal standard. a-inverse addition of amine was followed.

Table 3a: reaction condition evaluation with BCl_3

4.1b Reaction of BF_3K salt with Other Lewis Acids:

Procedure:

In a 2 dram vial with a stir bar inside was charged with ITX (5.50 mg, 0.0200 mmol, 0.100 equiv.) and *E*-StyrenylBF_3K salt **3** (42.2 mg, 0.200 mmol, 1.00 equiv.). After degassing and backfilling with N_2 (X 3), under N2 atmosphere, 2 mL of anhydrous DCM was added. After that Lewis acid (3.3 equiv.) was added to the reaction mixture dropwise under inert atmosphere at the mixture was allowed to stir for 10 minutes at rt. [In case of BF_3.N(H)Et_2, it was added as a solution in 0.5 mL anhydrous DCM under inert atmosphere]. After that N,N-dimethylallylamine **1** (47 μL, 0.40 mmol, 2.0 equiv.) was added to the reaction mixture via a syringe dropwise. The rubber septum was quickly replaced by a plastic cap and the reaction vial was sealed with a Teflon tape. The reaction vial was allowed to stir under 395 nm LEDs irradiation at rt for 16 h. After the reaction time period, all the volatiles were removed in vacuo and the crude mixture was taken for ^{1}H-NMR analysis using CH_2Br_2 as internal standard.

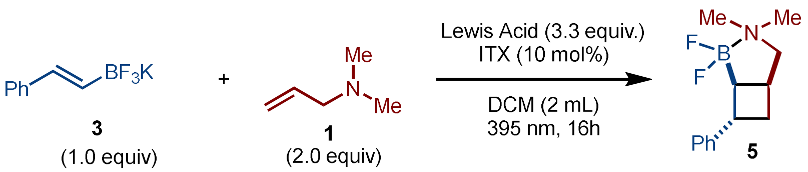

Entry	Lewis Acid	NMR yield of XX
1.	$BF_3{\cdot}OEt_2$	trace
2.	TMSCl	n.d.
3.	$BF_3.N(H)Et_2$	n.d.

reaction were performed in 0.2 mmol scale, NMR yield were determined using CH_2Br_2 as internal standard.

Table 3b: Reaction of BF_3K salt with other Lewis acids

4.1c Screening of amount of BF_3NEt_4 salt:

Procedure:

In a 2 dram vial with a stir bar inside was charged with ITX (5.5 mg, 0.020 mmol, 0.10 equiv.) and *E*-StyrenylBF_3NEt_4 salt **27**. After degassing and backfilling with N_2 (X 3), under N_2 atmosphere, 2 mL of anhydrous DCM was added. After that freshly distilled TMSCl (37 µL, 0.30 mmol, 1.5 equiv.) was added to the reaction mixture dropwise under inert atmosphere and the mixture was allowed to stir for 10 minutes at rt. After that N,N-dimethylallylamine **1** (24 µL, 0.20 mmol, 1.0 equiv.) was added to the reaction mixture via a syringe dropwise. The rubber septum was quickly replaced by a plastic cap and the reaction vial was sealed with a Teflon tape. The reaction vial was allowed to stir under 395 nm LEDs irradiation at rt for 16 h. After the reaction time period, all the volatiles were removed in vacuo and the crude mixture was taken for ^{1}H-NMR analysis using CH_2Br_2 as internal standard.

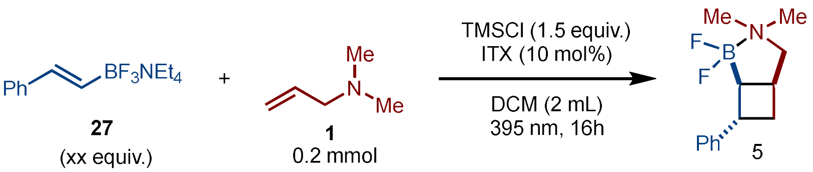

Entry	BF_3 salt	NMR yield of XX
1.	1.2 equiv.	65%
2.	1.3 equiv.	81%
3.	1.4 equiv.	84%
4.	1.5 equiv.	85%

Table 3c: Screening of amount of BF_3NEt_4 salt

4. 1d. Screening of amount of TMSCl with 1.3 equiv. of BF_3NEt_4 salt:

Procedure:

In a 2 dram vial with a stir bar inside was charged with ITX (5.5 mg, 0.020 mmol, 0.10 equiv.) and *E*-StyrenylBF_3NEt_4 salt **27** (78.2 mg, 0.260 mmol, 1.30 equiv.). After degassing and backfilling with N_2 (X 3), under N_2 atmosphere, 2 mL of DCM was added.

After that freshly distilled TMSCl was added to the reaction mixture dropwise under inert atmosphere at the mixture was allowed to stir for 10 minutes at rt. After that N,N-dimethylallylamine **1** (24 µL, 0.20 mmol, 1.0 equiv.) was added to the reaction mixture via a syringe. The rubber septum was quickly replaced by a plastic cap and the reaction vial was sealed with a Teflon tape. The reaction vial was allowed to stir under 395 nm LEDs irradiation at rt for 16 h. After the reaction time period, all the volatiles were removed in vacuo and the crude mixture was taken for ^{1}H-NMR analysis using CH_2Br_2 as internal standard.

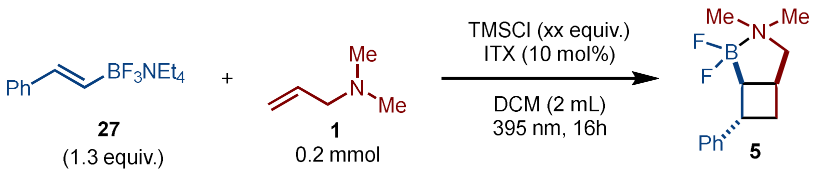

Entry	TMSCl	NMR yield of XX
1.	1.3 equiv.	65%
2.	1.5 equiv.	84%
3.	2.0 equiv.	59%
4.	2.5 equiv.	41%

Table 3d. Screening of amount of TMSCl with 1.3 equiv. BF_3NEt_4 salt

4.1e Screening of amount of TMSCl with 1.4 equiv. of BF_3NEt_4 salt:

Procedure:

In a 2 dram vial with a stir bar inside was charged with ITX (5.5 mg, 0.020 mmol, 0.10 equiv.) and *E*-StyrenylBF_3NEt_4 salt **27** (84.3 mg, 0.280 mmol, 1.40 equiv.). After degassing and backfilling with N_2 (X 3), under N_2 atmosphere, 2 mL of anhydrous DCM was added. After that freshly distilled TMSCl was added to the reaction mixture dropwise under inert atmosphere at the mixture was allowed to stir for 10 minutes at rt. After that N,N-dimethylallylamine **1** (24 µL, 0.20 mmol, 1.0 equiv.) was added to the reaction mixture via a syringe dropwise. The rubber septum was quickly replaced by a plastic cap and the reaction vial was sealed with a Teflon tape. The reaction vial was allowed to stir under 395 nm LEDs irradiation at rt for 16 h. After the reaction time period, all the volatiles were removed in vacuo and the crude mixture was taken for ^{1}H-NMR analysis using CH_2Br_2 as internal standard.

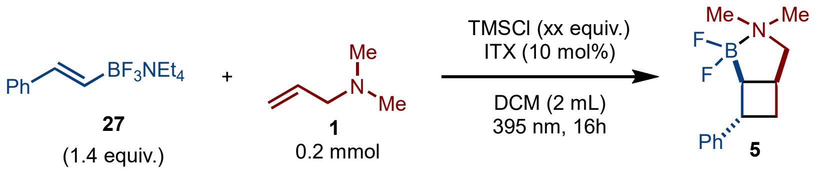

Entry	TMSCl	NMR yield of XX
1.	1.5 equiv.	83%
2.	1.6 equiv.	95%
3.	1.7 equiv.	81%

Table 3e. Screening of amount of TMSCl with 1.4 equiv. BF_3NEt_4 salt

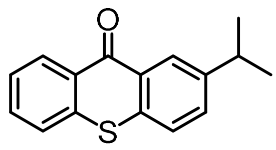

ITX

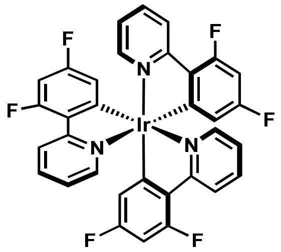

$Ir(dFppy)_3$

4.2 Optimization of Photochemical [4+2] Cycloadditions Reactions:

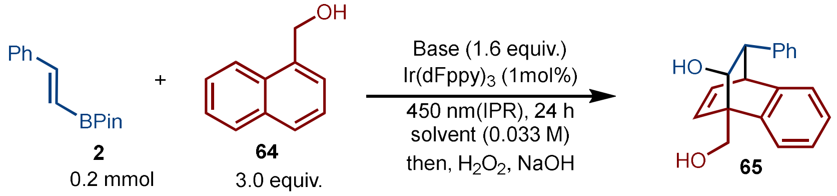

4.2a General procedure for optimization studies:

In a 2-dram vial with a stir bar inside it, $Ir(dFppy)_3$ (1.5 mg, 2.0 μmol, 1 mol %), Styrenyl-Bpin **2** (46 mg, 0.20 mmol, 1.0 equiv) was charged, along with the napthalene-methanol **64** (95 mg, 0.60 mmol, 3.0 equiv). then was taken inside the glovebox and base(0.32 mmol, 1.6 equiv) was added to it and capped and taken outside the glovebox. Then the reaction vial cap was quickly replaced with a rubber septum and the reaction mixture was degassed and filled with N_2 (X 3) followed by addition of the 6 mL of solvent. Then the rubber septum was quickly replaced by plastic cap and sealed with teflon. The vial was placed under the irradiation of visible light for 24 h in IPR. After that, the reaction mixture was transferred to a 25 mL round bottom flask containing 2 ml of 4N NaOH, washing with additional 2 mL of THF. Then 2 mL of 30% aqueous H_2O_2 was added to the solution and stirred at room temperature for additional 3h. Then the reaction mixture was extracted with EtOAc (3 X 5 mL), concentrated and taken for the ^{1}H-NMR analysis.

Entry	Base (1.6 equiv.)	Solvent	% NMR yield (dr)[a]
1.	KO*t*-Bu	Toluene	>95% (5:1 dr)
2	NaO*t*-Bu	Toluene	74% (5:1 dr)
3.	KOEt	Toluene	86% (5:1 dr)
4.	KO*t*-amyl	Toluene	93% (5:1 dr)
5.	KOMe	Toluene	50% (5:1 dr)
6.	KO*t*-Bu	DCM	91% (4:1 dr)
7.	KO*t*-Bu	THF	>95% (5:1 dr)
8.	KO*t*-Bu	MeCN	77% (2:1 dr)

experiments were carried out in 0.2 mmol scale, (*a*) yields and dr were calculated by crude NMR using CH_2Br_2 as internal standard.

Table 4.1: Optimization of [4+2] Cycloadditions Reaction Condition

5. Experimental Procedures:
5.1 Substrate Synthesis:
5.1a Preparation of alkenyl-Bpin:

Table 4.2: Starting material alkenyl-Bpins

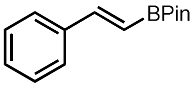

2

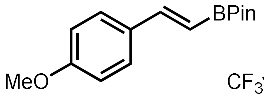

S1

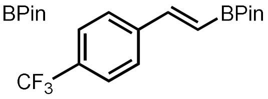

S2

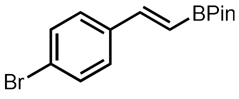

S3

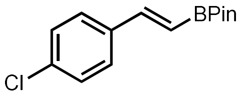

S4

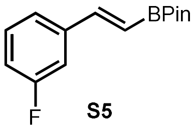

S5

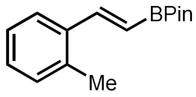

S6

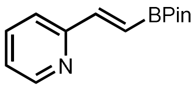

S7

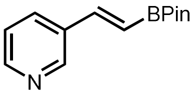

S8

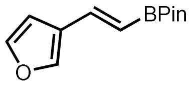

S9

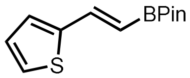

S10

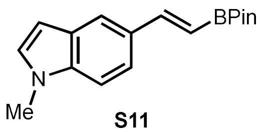

S11

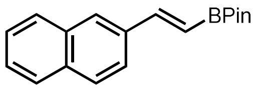

S12

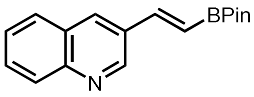

S13

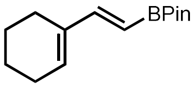

S14

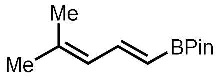

S15

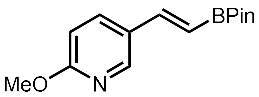

S16

All the alkenyl-Bpin substrates (**SI 2.1-2.18**) were prepared according to the literature procedure, and the spectral data matches with the literature report. [8, 9]

5.1b Preparation of Styrenyl-BF_3 salts:

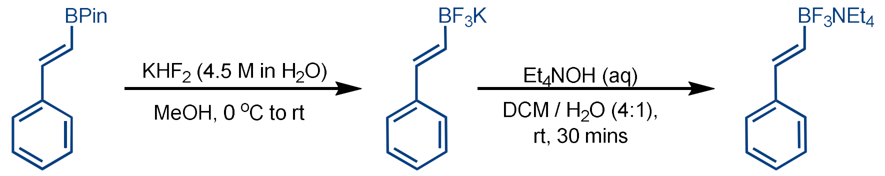

General Procedure 1:

The trifluoroborate tetraethylammonium salts are prepared with a slight modification of the literature procedure.[10] To a solution of the boronic acid pinacol ester or boronic acid (1.0 equiv) in MeOH (0.5 M) was added 4.5 M aq. KHF_2 (3.0 equiv) at 0 °C, the resulting mixture was stirred at the same temperature for 1h then at rt for 1h. Solvent was removed in rotary evaporator; residue was dissolved in hot acetone and filtered. The filtrate was concentrated and dissolved in minimum amount of acetone, preAcipitated by adding MTBE. The precipitate was collected by filtration to get products (BF_3K salts) as white solids.

A 35% by volume solution of tetraethylammonium hydroxide in water (1.2 equiv.) was added to a suspension of potassium trifluoroborate in a 4:1 mixture of DCM and water (0.2 M). The biphasic mixture was stirred for 30 minutes, by this time all of the starting material had dissolved. The organic layer was separated and the aqueous layer washed with DCM (3 x 15 mL). The combined organic fractions were dried over $MgSO_4$ and the solvent evaporated to give tetraethylammonium styrenyltrifluoroborate salts.

Table 4.3: Starting material BF_3 salts

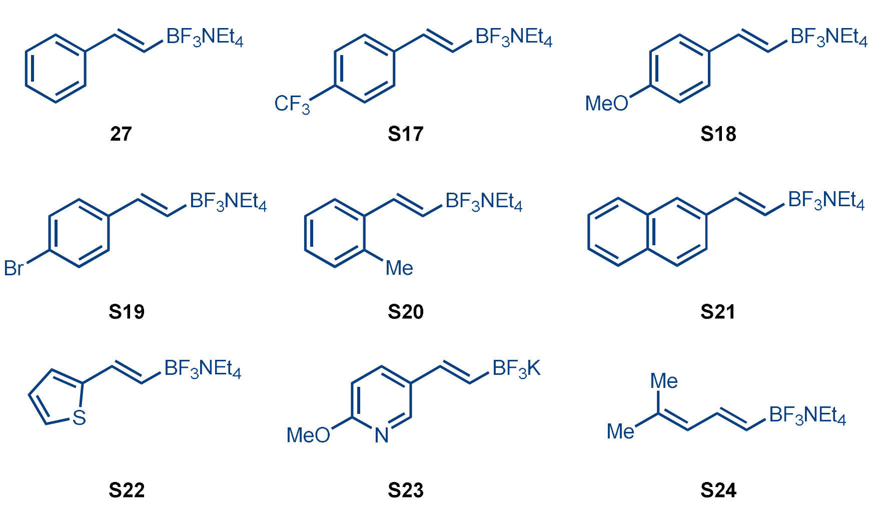

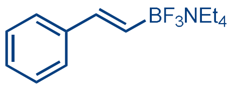

27

(E)-trifluoro(styryl)borate tetraethylammonium (27): The title compound was made according to the general procedure **1** starting from E-Phenylethynylboronic acid (5.00 g, 33.8 mmol, 1.00 equiv). The compound was obtained as a white solid (8.10 g, 27 mmol, 80% yield).

^{1}H NMR (500 MHz, $CDCl_3$): δ 7.35 (d, *J* = 7.4 Hz, 2H), 7.23 (t, *J* = 7.6 Hz, 2H), 7.12 – 7.07 (m, 1H), 6.82 – 6.48 (m, 1H), 6.42 – 6.33 (m, 1H), 3.14 (q, *J* = 7.3 Hz, 8H), 1.17 (tt, *J* = 7.3, 1.9 Hz, 12H).

^{13}C NMR (126 MHz, $CDCl_3$): δ = 140.4, 134.7(q, *J* = 5.0 Hz), 128.3, 126.2, 125.8, 52.4 (t, *J* = 3.8 Hz), 7.4.

^{19}F NMR (376 MHz, CD_3CN): δ = -140.89.

^{11}B NMR (160 MHz, $CDCl_3$): δ = 2.69.

HRMS (ESI, m/z): Calculated for $C_8H_7BF_3$ $[M\text{-}NEt_4]^-$: 171.0598. Observed: 171.0595.

IR (neat): 1489, 1233, 1063, 967, 832 cm^{-1}.

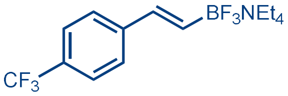

S-17

(E)-trifluoro(4-(trifluoromethyl)styryl)borate tetraethylammonium (S17): The title compound was made according to the general procedure **1** starting from the corresponding (E)-4,4,5,5-tetramethyl-2-(4-(trifluoromethyl)styryl)-1,3,2-dioxaborolane **S2** (597 mg, 2.00 mmol, 1.00 equiv). The compound was obtained as a white solid (520 mg, 1.41 mmol, 70%).

^{1}H NMR (500 MHz, $CDCl_3$): δ 7.48 (m, 4H), 6.94 – 6.73 (m, 1H), 6.65 – 6.49 (m, 1H), 3.59 – 2.88 (m, 8H), 1.31 (tt, *J* = 4.9, 2.0 Hz, 12H).

^{13}C NMR (126 MHz, $CDCl_3$): δ = 144.1, 133.7, 127.7 (d, *J* = 32.8 Hz), 126.0, 125.1 (q, J = 3.8 Hz), 123.5, 52.6 (t, J = 2.5 Hz), 7.5.

^{19}F NMR (376 MHz, $CDCl_3$): δ = -62.17, -141.70.

HRMS (ESI, m/z): Calculated for $C_9H_6BF_6$ $[M\text{-}NEt_4]^-$: 239.0472. Observed: 239.0468.

IR (neat): 3000, 1489, 1333, 1234, 1163, 1108, 861 cm^{-1}.

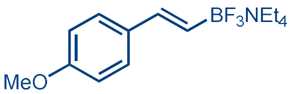

S18

(E)-trifluoro(4-methoxystyryl)borate tetraethylammonium (S18): The title compound was made according to the general procedure **1** starting from the corresponding (E)-4,4,5,5-tetramethyl-2-(4-methoxystyryl)-1,3,2-dioxaborolane **S1** (520 mg, 2.00 mmol, 1.00 equiv). The compound was obtained as a white solid (335 mg, 1.00 mmol, 50%).

^{1}H NMR (500 MHz, CDCl$_3$): δ 7.35 – 7.28 (m, 2H), 6.82 – 6.76 (m, 2H), 6.76 – 6.63 (m, 1H), 6.33 – 6.17 (m, 1H), 3.77 (s, 3H), 3.23 (q, *J* = 7.3 Hz, 8H), 1.25 (tt, *J* = 7.4, 1.9 Hz, 12H).
^{13}C NMR (126 MHz, CDCl$_3$): δ = 158.1, 134.2, 133.6, 126.9, 113.6, 55.3, 52.5 (t, *J* = 3.8 Hz), 7.5.
HRMS (ESI, m/z): Calculated for $C_9H_9OBF_3$ [M-NEt$_4$]$^-$: 201.0704. Observed: 201.0702.
IR (neat): 2987, 1604, 1508, 1249, 1061, 976, 852 cm^{-1}.

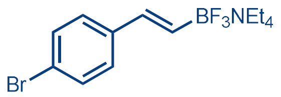

S19

(E)-(4-bromostyryl)trifluoroborate tetraethylammonium (S19): The title compound was made according to the general procedure **1** starting from the corresponding (E)-4,4,5,5-tetramethyl-2-(4-bromostyryl)-1,3,2-dioxaborolane **S3** (616 mg, 2.00 mmol, 1.00 equiv). The compound was obtained as a white solid (490 mg, 1.30 mmol, 65%).
^{1}H NMR (500 MHz, CDCl$_3$): δ 7.41 – 7.30 (m, 2H), 7.25 (d, *J* = 8.6 Hz, 2H), 6.87 – 6.54 (m, 1H), 6.53 – 6.07 (m, 1H), 3.22 (q, *J* = 7.3 Hz, 8H), 1.24 (tt, *J* = 7.3, 1.8 Hz, 12H).
^{13}C NMR (126 MHz, CDCl$_3$): δ = 139.5, 133.6(q, *J* = 3.8 Hz), 131.3, 127.5, 119.6, 52.5(t, *J* = 2.5 Hz), 7.5.
HRMS (ESI, m/z): Calculated for $C_8H_6BBrF_3$ [M-NEt$_4$]$^-$: 248.9704. Observed: 248.9702.
IR (neat): 2988, 1484, 1283, 1057, 977, 855, 793 cm^{-1}.

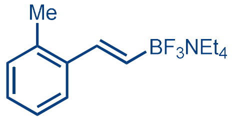

S20

(E)-trifluoro(2-methylstyryl)borate tetraethylammonium S20: The title compound was made according to the general procedure **1** starting from the corresponding (E)-4,4,5,5-tetramethyl-2-(4-bromostyryl)-1,3,2-dioxaborolane **S6** (732 mg, 3.00 mmol, 1.0 equiv). The compound was obtained as a white solid (570 mg, 1.80 mmol, 60%).
^{1}H NMR (500 MHz, CDCl$_3$): δ 7.55 (dd, *J* = 7.8, 1.4 Hz, 1H), 7.13 – 6.99 (m, 4H), 6.46 – 6.18 (m, 1H), 3.26 (qd, *J* = 7.4, 1.5 Hz, 8H), 2.35 (s, 3H), 1.29 (tt, *J* = 7.3, 1.8 Hz, 12H).
^{13}C NMR (126 MHz, CDCl$_3$): δ 139.6, 134.7, 132.3, 129.9, 125.9, 125.7, 125.1, 52.5 (t, *J* = 3.8 Hz), 20.0, 7.5.
HRMS (ESI, m/z): Calculated for $C_9H_9BF_3$ [M-NEt$_4$]$^-$: 185.0755. Observed: 185.0754.
IR (neat): 2948, 1481, 1231, 1066, 958, 787, 753 cm^{-1}.

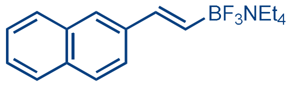

S21

(E)-trifluoro(2-(naphthalen-2-yl)vinyl)borate tetraethylammonium (S21): The title compound was made according to the general procedure **1** starting from the corresponding (E)-4,4,5,5-tetramethyl-2-(2-(naphthalen-2-yl)vinyl)-1,3,2-dioxaborolane **S12** (920 mg, 3.30 mmol, 1.00 equiv). The compound was obtained as a white solid (830 mg, 2.40 mmol, 72%).

^{1}H NMR (500 MHz, $CDCl_3$): δ 7.75 (ddd, *J* = 7.7, 5.8, 1.4 Hz, 2H), 7.71 – 7.65 (m, 3H), 7.43 – 7.30 (m, 2H), 7.09 – 6.84 (m, 1H), 6.72 – 6.25 (m, 1H), 3.18 (q, *J* = 7.3 Hz, 8H), 1.21 (tt, *J* = 7.3, 1.8 Hz, 12H).

^{13}C NMR (126 MHz, $CDCl_3$): δ = 138.2, 134.9 (m), 133.9, 132.5, 127.8, 127.64, 127.56, 125.8, 125.0, 125.0, 124.0, 52.7 (t,*J*= 2.5 Hz) 7.4.

HRMS (ESI, m/z): Calculated for $C_{12}H_9BF_3$ $[M\text{-}NEt_4]^-$: 221.0755. Observed: 221.0753.

IR (neat): 2988, 1622, 1483, 1060, 993, 789 cm^{-1}.

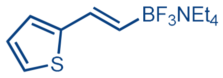

S22

(E)-trifluoro(2-(thiophen-2-yl)vinyl)borate tetraethylammonium (S22): The title compound was made according to the general procedure **1** starting from the corresponding (E)-4,4,5,5-tetramethyl-2-(2-(thiophen-2-yl)vinyl)-1,3,2-dioxaborolane **S10** (690 mg, 2.90 mmol, 1.00 equiv). The compound was obtained as a white solid (620 mg, 2.00 mmol, 69%).

^{1}H NMR (500 MHz, $CDCl_3$): δ 7.00 (d, *J* = 5.1 Hz, 1H), 6.94 – 6.83 (m, 2H), 6.81 (d, *J* = 3.5 Hz, 1H), 6.33 – 5.89 (m, 1H), 3.27 – 3.19 (m, 8H), 1.30 – 1.23 (m, 12H).

^{13}C NMR (126 MHz, $CDCl_3$): δ = 147.6, 127.9(q, *J* = 5.0 Hz), 127.2, 123.0, 122.3, 52.4(t, *J* = 3.8 Hz), 7.4.

HRMS (ESI, m/z): Calculated for $C_6H_5BF_3S$ $[M\text{-}NEt_4]^-$: 177.0163. Observed: 177.0163.

IR (neat): 2992, 1614, 1396, 1185, 1059, 980 cm^{-1}.

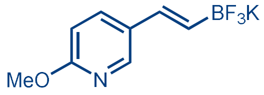

S23

(E)-2-methoxy-5-(2-(trifluoro-l4-boraneyl)vinyl)pyridine, potassium salt (S23): The title compound was made according to a slight modification of general procedure **1**. (E)-2-methoxy-5-(2-(4,4,5,5-tetramethyl-1,3,2-dioxaborolan-2-yl)vinyl)pyridine **S16** (300 mg, 1.10 mmol) was dissolved in acetonitrile (6 mL) and saturated aqueous KHF_2 (4.5 M, 1.3 mL, 5.1 equiv) was added. The reaction mixture was stirred at room temperature for 3 hours, concentrated, azeotroped with methanol, and placed on the vacuum for 3 hours. The crude product was dissolved in hot acetone, filtered, and then

concentrated. To the resulting crude product, Et_2O (10 mL) was added, and the mixture was sonicated for 15 min and filtered to give the desired product XX as a white solid (85 mg, 0.4 mmol, 31%).

^{1}H NMR (500 MHz, DMSO): δ 8.01 (d, *J* = 2.5 Hz, 1H), 7.72 (dd, *J* = 8.6, 2.4 Hz, 1H), 6.71 (d, *J* = 8.6 Hz, 1H), 6.48 – 6.28 (m, 1H), 6.15 – 6.05 (m, 1H), 3.82 (s, 3H).

^{13}C NMR (126 MHz, DMSO): δ = 162.5, 144.6, 135.6, 130.1, 129.3 (m), 110.7, 53.4.

HRMS (ESI, m/z): Calculated for $C_8H_8ONBF_3$ $[M-K]^-$: 171.0598. Observed: 171.0595.

IR (neat): 2988, 1600, 1493, 1378, 1085, 991 cm^{-1}.

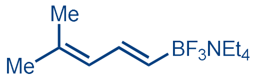

S24

(E)-trifluoro(4-methylpenta-1,3-dien-1-yl)-l4-borane tetraethylammonium (S24): The title compound was made according to the general procedure **1** starting from the corresponding (E)-4,4,5,5-tetramethyl-2-(4-methylpenta-1,3-dien-1-yl)-1,3,2-dioxaborolane **S15** (416 mg, 2.00 mmol, 1.00 equiv). The compound was obtained as a white solid (290 mg, 1.07 mmol, 53%).

^{1}H NMR (500 MHz, $CDCl_3$): δ 6.64 (dd, *J* = 17.4, 10.6 Hz, 1H), 5.80 (d, *J* = 10.6 Hz, 1H), 5.62 (dq, *J* = 17.4, 4.2 Hz, 1H), 3.29 (q, *J* = 7.3 Hz, 9H), 1.74 – 1.68 (m, 6H), 1.30 (tt, *J* = 7.3, 1.8 Hz, 12H).

^{13}C NMR (126 MHz, $CDCl_3$): δ = 132.1 (d, J = 5.04 Hz), 130.4, 129.6, 52.5 (t, *J* = 3.75 Hz), 25.9, 18.3, 7.5.

HRMS (ESI, m/z): Calculated for $C_9H_9OBF_3$ $[M-NEt_4]^-$: 165.0704. Observed: 165.0702.

IR (neat): 2981, 1715, 1174, 1049, 999 cm^{-1}.

5.1c Synthesis of Starting Materials: Allylamines:

Table 4.4: Starting material allylamines

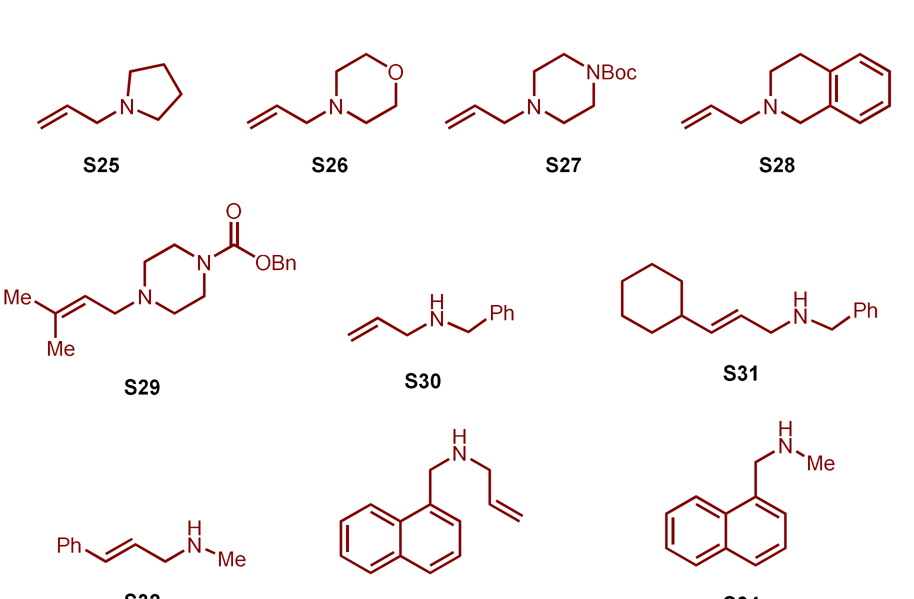

S25 **S26** **S27** **S28** **S29** **S30** **S31** **S32** **S33** **S34**

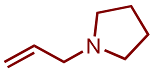

1-allylpyrrolidine (S25): was prepared according to the literature procedure. All characterization was in accordance with literature reports.[11]

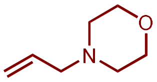

4-allylmorpholine (S26): was prepared according to the literature procedure. All characterization was in accordance with literature reports.[12]

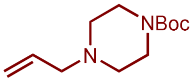

tert-butyl 4-allylpiperazine-1-carboxylate (S27): was prepared according to the literature procedure. All characterization was in accordance with literature reports.[13]

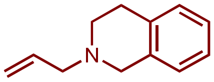

2-allyl-1,2,3,4-tetrahydroisoquinoline (S28): was prepared according to the literature procedure. All characterization was in accordance with literature reports [14]

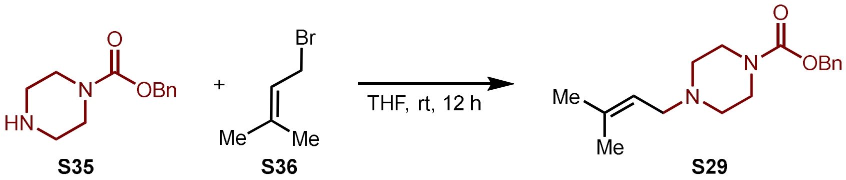

benzyl 4-(3-methylbut-2-en-1-yl)piperazine-1-carboxylate (S29): was prepared with a slight modification of literature procedure. A solution of the 1-bromo-3-methylbut-2-ene **(S36)** (5.00 mmol) in THF (10 mL) was added dropwise to a solution of the secondary amine **(S35)** (2.73 g, 12.5 mmol, 2.50 equiv) in THF (5 mL) via a syringe at rt. After addition was complete, the reaction was stirred for 12 h. 1 M aq NaOH (10 mL) was then added in one portion, and the reaction stirred for a further 10 minutes. The reaction was diluted with Et_2O (25 mL), the layers separated and the aqueous layer extracted with Et_2O (2 x 10 mL). The combined organics were washed with brine (equal volume), dried over $MgSO_4$, filtered, and concentrated in vacuo. The residue was triturated with Et_2O, any solids removed by filtration, and the solution was concentrated in vacuo to afford the tertiary allylic amines that was purified further by flash chromatography on silica gel (DCM/MeOH = 49:1). Colorless oil [1.05 g, 3.64 mmol, 73%].

^{1}H NMR (500 MHz, $CDCl_3$): δ 7.40 – 7.28 (m, 5H), 5.23 (tp, *J* = 6.5, 1.4 Hz, 1H), 5.13 (s, 2H), 3.52 (t, *J* = 5.1 Hz, 4H), 2.96 (d, *J* = 7.0 Hz, 2H), 2.40 (s, 4H), 1.74 (d, *J* = 1.6 Hz, 3H), 1.64 (d, *J* = 1.3 Hz, 3H).

^{13}C NMR (126 MHz, $CDCl_3$): δ = 155.4, 136.9, 136.1, 128.6, 128.1, 128.0, 120.6, 67.2, 56.2, 52.9, 44.0, 26.1, 18.2.

HRMS (ESI, m/z): Calculated for $C_{17}H_{24}O_2N_2Na$ [M+Na]$^+$: 311.1730, Observed: 311.1731.
IR (neat): 2914, 1700, 1428, 1237, 697 cm^{-1}.

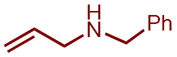

N-benzylprop-2-en-1-amine (S30): was prepared according to the literature procedure. All characterization was in accordance with literature reports.[15]

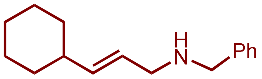

(E)-N-benzyl-3-cyclohexylprop-2-en-1-amine (S31): was prepared according to the literature procedure. All characterization was in accordance with literature reports.[16]

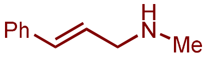

(E)-N-methyl-3-phenylprop-2-en-1-amine (S32): was prepared according to the literature procedure. [17] The crude material was further purified by FCC (EtOAc: hexanes = 1:1) to afford the product a pale-yellow oil. All characterization was in accordance with literature reports.[18]

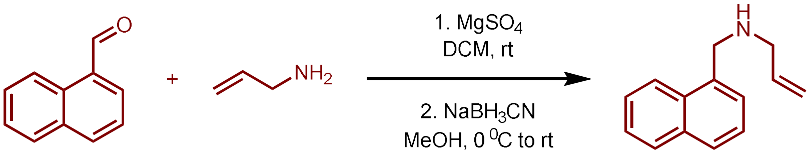

S37 **S38** **S33**

N-(naphthalen-1-ylmethyl)prop-2-en-1-amine (S33) was prepared from 1-naphthaldehyde **(S37)** using following procedure. In a 100 mL round bottom flask, with a stir bar inside, 1-naphthaldehyde **(S37)** (1.6 g, 10 mmol, 1.0 equiv) was dissolved in DCM (15 mL) at room temperature. $MgSO_4$ (1.8 g, 15 mmol, 1.5 equiv) and allylamine **(S38)** (0.7 mL, 10 mmol) were added and the solution was stirred at room temperature for 3h. At the end of the reaction, the mixture was filtered over celite pad and the solvent was removed under reduced pressure. The resulting yellow oil was dissolved in MeOH (30 mL) at 0 °C, $NaBH_3CN$ (0.82 g, 13.0 mmol, 1.30 equiv) was added and the reaction was stirred at room temperature overnight. The solution was then diluted with EtOAc (20 mL), washed with water (40 mL) and the aqueous phase was extracted with EtOAc (3 x 20 mL). The combined organic layers were washed with brine (2 x 30 mL), dried over anhydrous Na2SO4, and then concentrated in vacuo. The crude material was purified by FCC (EtOAc / hexanes) to afford the title compound as a yellow oil (1.3 g, 6.6 mmol, 66% yield).
^{1}H NMR (500 MHz, $CDCl_3$): δ 8.16 – 8.11 (m, 1H), 7.90 – 7.84 (m, 1H), 7.78 (d, J = 8.1 Hz, 1H), 7.57 – 7.40 (m, 4H), 6.00 (ddt, J = 17.1, 10.2, 6.0 Hz, 1H), 5.32 – 5.21 (m, 1H), 5.15 (dq, J = 10.2, 1.4 Hz, 1H), 4.25 (s, 2H), 3.40 (dt, J = 6.0, 1.5 Hz, 2H), 1.50 – 1.25 (m, 1H).
^{13}C NMR (126 MHz, $CDCl_3$): δ = 137.0, 136.0, 134.0, 132.0, 128.8, 127.9, 126.22, 126.19, 125.7, 125.5, 123.8, 116.3, 52.5, 51.0.
IR (neat): 3046, 2812, 1596, 1218, 916, 790 cm^{-1}.
HRMS (ESI, m/z): Calculated for C14H16N [M+H]$^+$: 198.1277, Observed: 198.1276.

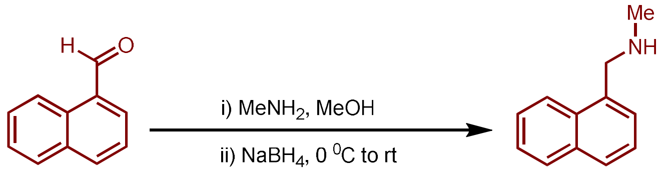

S37 **S34**

N-methyl-1-(naphthalen-1-yl)methanamine (S34) was prepared according to the literature procedure. To a round bottomed flask charged with 1-naphthaldehyde **(S37)** (782 mg, 5.00 mmol, 1.00 equiv) was added MeOH (25 mL) and methylamine (1.50 equiv., 3.75 mL) (2M solution in methanol). After stirring for 2 hours, $NaBH_4$ (379 mg, 10.0 mmol, 2.00 equiv) was added slowlyn at 0 ^{0}C, and the resultant mixture was stirred until bubbling ceased. The reaction mixture was quenched with 1 M NaOH (aq.) and extracted with EtOAc (3 X 10 mL). The combined organic layers were washed with 1 M HCl (aq.), and the resulting aqueous layer was brought to pH 14 with 2 M NaOH (aq.) and extracted with EtOAc (3 X 10 mL). The combined organic layers were dried over $MgSO_4$ filtered and concentrated under reduced pressure. The crude product was purified by FCC (5% MeOH in DCM). Yellow oil, 680 mg, 3.97 mmol, 79%.

^{1}H NMR (500 MHz, $CDCl_3$): δ 8.15 – 8.09 (m, 1H), 7.87 (dd, *J* = 8.2, 1.5 Hz, 1H), 7.78 (d, *J* = 8.0 Hz, 1H), 7.58 – 7.40 (m, 4H), 4.21 (s, 2H), 2.56 (s, 3H)

^{13}C NMR (126 MHz, $CDCl_3$): δ = 135.6, 134.0, 132.0, 128.9, 128.0, 126.4, 126.3, 125.8, 125.5, 123.7, 53.8, 36.6.

IR (neat): 2935, 1596, 1264, 791, 776 cm^{-1}.

HRMS (ESI, m/z): Calculated for $C_{12}H_{14}N$ $[M+H]^+$: 172.1121, Observed: 172.1121.

5.1d Synthesis of Starting Materials 2-vinylpyridines:

Table 4.5: Starting material 2-vinylpyridines

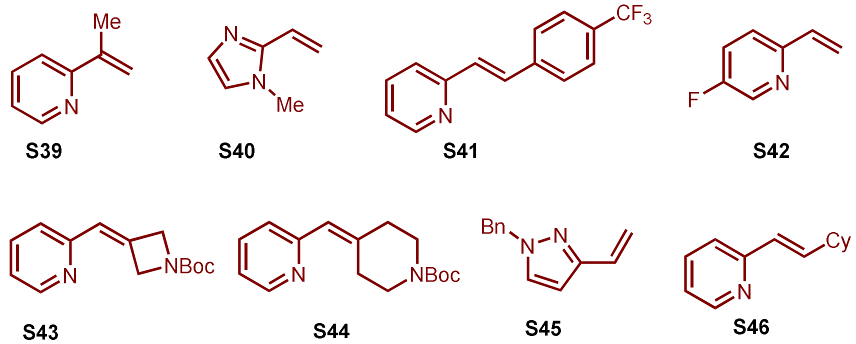

General procedure for Wittig reaction:

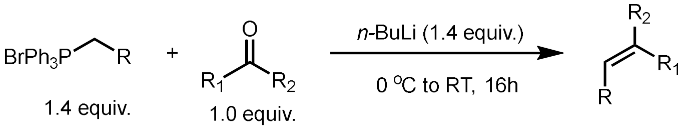

Wittig reaction procedure was modified from the existing literature procedure by Miller

& co-workers[1]. To an oven-dried 100 mL flask equipped with a stir bar was added phosphonium salt (1.4 equiv., 7.0 mmol). The flask was subsequently capped with a rubber septum and put under a nitrogen atmosphere. Anhydrous THF (25 mL) from a solvent system was then added via syringe and the resulting white slurry was cooled to 0 ^oC in an ice bath under a nitrogen atmosphere. Next, *n*-butylithium (7.0 mmol, 1.4 equiv, 2.8 mL of a 2.5 M solution in hexanes) was added dropwise. After complete addition of n-butyllithium, the bright orange solution was allowed to stir at 0 ^oC for 3h. After stirring for 3h, freshly distilled aldehyde/ketone (1.0 equiv, 5.0 mmol) was added to the orange solution dropwise. The reaction mixture was allowed to stir for 16 hours, warming to room temperature. After 16 hours, the reaction mixture was cooled to 0 ^oC in an ice bath. Under a nitrogen atmosphere, 10 mL of saturated aqueous ammonium chloride solution was added dropwise and allowed to stir for five minutes. The contents of the flask were then transferred to a separatory funnel containing 100 mL of 1:1 ethyl acetate: saturated aqueous ammonium chloride solution. The organic layer was then separated and dried over Na_2SO_4, filtered, and concentrated via rotary evaporation. Next, the crude product was purified via silica gel column chromatography, eluting with hexanes and ethyl acetate to obtain the corresponding alkene.

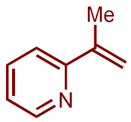

S39

2-(prop-1-en-2-yl) pyridine (S39): The title compound was prepared according to the general procedure for Wittig reaction using 1-(pyridin-2-yl)ethan-1-one (606 mg, 5.00 mmol, 1.00 equiv) and Methyltriphenylphosphonium bromide (2.5 g, 7.0 mmol, 1.4 equiv.). Yield: 0.43 g, 3.6 mmol, 72%. Spectral data matches with existing literature report. [19]

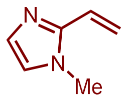

S40

1-methyl-2-vinyl-1H-imidazole (S40): The title compound was prepared according to the general procedure for Wittig reaction using 1-methyl-1H-imidazole-2-carbaldehyde (0.55 g, 5.0 mmol, 1.00 equiv) and Methyltriphenylphosphonium bromide (2.5 g, 7.0 mmol, 1.4 equiv.). Yield: 240 mg, 2.20 mmol, 44%. For separation we used *Kugelrohr* distillation technique as the product and triphenylphosphine oxide have same polarity. Spectral data matches with existing literature report [20].

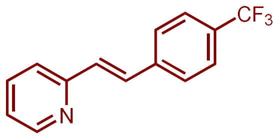

S41

(E)-2-(4-(trifluoromethyl)styryl)pyridine (S41): The title compound was prepared according to the general procedure for Wittig reaction using 4-(trifluoromethyl)benzaldehyde (0.87 g, 5.0 mmol, 1.0 equiv) and Phosphonium, triphenyl(2-pyridinylmethyl)-, chloride (1:1) (2.7g, 7.0 mmol, 1.4 equiv.). Yield: 1.1 g, 4.3 mmol, 85% (E/Z 7:1). Spectral data matches with existing literature report[21].

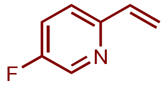

S42

5-fluoro-2-vinylpyridine (S42): The title compound was prepared according to the general procedure for Wittig reaction using 5-fluoropicolinaldehyde (0.63 g, 5.0 mmol, 1.0 equiv) and Methyltriphenylphosphonium bromide (2.5 g, 7.0 mmol, 1.4 equiv.). Yield: 0.55 g, 4.5 mmol, 89%. Spectral data matches with existing literature report [22].

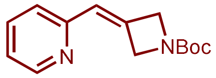

S43

tert-butyl 3-(pyridin-2-ylmethylene)azetidine-1-carboxylate (S43): The title compound was prepared according to the general procedure for Wittig reaction using tert-butyl 3-oxoazetidine-1-carboxylate (0.86 g, 5.0 mmol, 1.0 equiv) and Phosphonium, triphenyl(2-pyridinylmethyl)-, chloride (1:1) (2.7 g, 7.0 mmol, 1.4 equiv.). Yield: 1.0 g, 4.0 mmol, 81%. Spectral data matches with existing literature report [23].

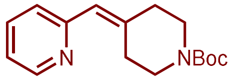

S44

tert-butyl 4-(pyridin-2-ylmethylene)piperidine-1-carboxylate (S44): The title compound was prepared according to the general procedure for Wittig reaction using tert-butyl 4-oxopiperidine-1-carboxylate (0.99 g, 5.0 mmol, 1.0 equiv) and phosphonium, triphenyl(2-pyridinylmethyl)-, chloride (1:1) (2.7 g, 7.0 mmol, 1.4 equiv.). Yield: 830 mg, 3.40 mmol, 68%. Spectral data matches with existing literature report [24].

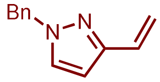

S45

1-benzyl-3-vinyl-1H-pyrazole (S45): The title compound was prepared according to the general procedure for Wittig reaction using 1-benzyl-1H-pyrazole-3-carbaldehyde (0.94 g, 5.0 mmol, 1.0 equiv) and Methyl triphenylphosphonium bromide (2.5 g, 7.0 mmol, 1.4 equiv.). Yield: 0.35 g, 1.9 mmol, 38%. Spectral data matches with existing literature report.[25]

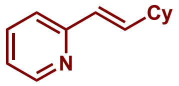

S46

(E)-2-(2-cyclohexylvinyl)pyridine (S46): was prepared according to the literature procedure.[26]

5.1e Preparation of naphthyl-methanols:

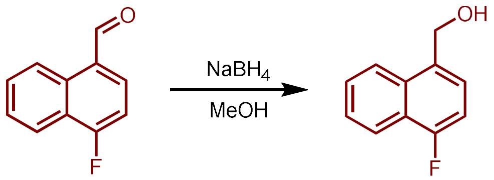

S47 **S48**

(4-fluoronaphthalen-1-yl)methanol (S48) was prepared from the borohydride reduction of 4-fluoro-1-naphthaldehyde. To a flask with stir bar was added aldehyde **(S47)** (697 mg, 4.00 mmol, 1.00 equiv.) and methanol (20 mL) and cooled to 0 °C. Sodium borohydride (303 mg, 8.00 mmol, 2.00 equiv.) was added portion-wise and let warm to room temperature and stir for 2 h. Reaction solution was then diluted with NH_4Cl (10 mL) and Ethyl Acetate (50 mL), the layers separated, and the organic phase rinsed with H_2O, brine, dried with anhydrous $MgSO_4$, filtered, and concentrated under reduced pressure. If necessary, the resulting residue was purified by column chromatography. The product was isolated as a white solid (680 mg, 3.86 mmol, 97%). The spectral data matched with the literature. [27]

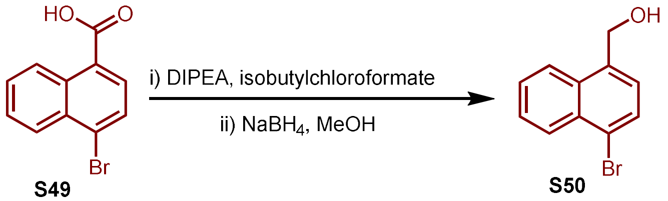

S49 **S50**

(4-bromonaphthalen-1-yl)methanol (S50) was prepared according to the literature procedure from 4-bromo-1-naphthoic acid.[28] A 50 mL flask with stir bar was flame-dried under vacuum. Once cooled, the flask was refilled with N_2. Then the flask was briefly opened and charged with 4-bromo-1-naphthoic acid **S49** (1.0 g, 4.0 mmol, 1.0 equiv.), then evacuated and backfilled 3 x with nitrogen. THF (25 mL) was added and the reaction was cooled to -10°C via NaCl ice bath. N,N-diisopropylethylamine (0.76 mL, 4.4 mmol, 1.1 equiv.) was added, followed by dropwise addition of isobutyl chloroformate (0.573 mL, 4.40 mmol, 1.10 equiv.), and let stir for 0.5 h. The solution was then filtered through a plug of celite into a flask containing sodium borohydride (605.3 mg, 16.00 mmol, 4.000 equiv.) dissolved in a minimal amount of H_2O. The reaction solution was stirred until gas formation ceased, then quenched by dropwise addition of 1N HCl (5 mL), partitioned between Ethyl Acetate/H_2O, rinsed with $NaHCO_3$, brine, dried with $MgSO_4$, filtered, and condensed to give a white solid which was used purified by FCC to give the product (640 mg, 2.70 mmol, 68%). The spectral data matches with the literature.[29]

5.2 Photochemical [2+2] cycloadditions:

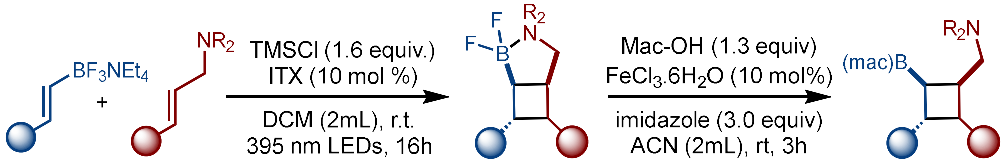

General Procedure 2:

In a 2-dram vial with a stir bar inside was charged with BF_3 salt (0.28 mmol, 1.4 eq), ITX (5.1 mg, 0.02 mmol, 0.10 equiv) and degassed and backfilled with N_2 (X 3). Then 2ml of DCM was added under N_2 atmosphere followed by the addition of TMSCl (40 μL, 0.32 mmol, 1.6 equiv) dropwise at room temperature and stirred it for 10 mins at rt. Then with a syringe, allyl amine (0.2 mmol, 1.0 equiv) was slowly added to the reaction vial under N_2 atmosphere, and the septum was quickly replaced with plastic cap and sealed with teflon tape. The reaction was allowed to be irradiated under 395 nm for 16 h. After that the volatiles were removed in vacuo and taken for crude NMR analysis using CH_2Br_2 as internal standard.

After that the Mac-OH (1.30 equiv, 55.6 mg, 0.26 mmol), Imidazole (3.00 equiv, 40.8 mg, 0.600 mmol), $FeCl_3.6H_2O$ (5.5 mg, 0.020 mmol, 0.10 equiv.) was added to the crude reaction mixture followed by addition of 2 mL acetonitrile. The reaction was allowed to stir in open air at room temperature for 3 h. After that, the reaction mixture was passed through a small celite plug. The mixture was then concentrated and purified with flash column chromatography with pre-basified silica gel with Et_3N.

Note: Ir(dFppy)$_3$ (1.53 mg, 2.00 μmol, 0.0100 equiv) has been used as sensitizer under 450 nm LEDs instead of ITX for the cycloadditions of free amines.

General Procedure 3:

In a 2-dram vial with a stir bar inside was charged with BF_3 salt (0.20 mmol, 1.00 equiv), ITX (5.1 mg, 0.020 mmol, 0.10 equiv) and degassed and backfilled with N_2 (x3). Then 2 ml of DCM was added under N_2 atmosphere followed by the addition of a solution of BCl_3 (1M in DCM) (0.24 mL, 0.24 mmol, 1.20 equiv) dropwise at room temperature and stirred it for 10 mins at rt. Then with a syringe, allyl amine (0.4 mmol, 2.0 equiv) was slowly added to the reaction vial under N_2 atmosphere, and the septum was quickly replaced with plastic cap and sealed with teflon tape. The reaction was allowed to be irradiated under 395 nm for 16 h. After that the volatiles were removed in vacuo and taken for crude NMR analysis using CH_2Br_2 as internal standard.

After that the Mac-OH (1.30 equiv, 55.6 mg, 0.260 mmol), Imidazole (3.00 equiv, 40.8 mg, 0.600 mmol), $FeCl_3.6H_2O$ (5.5 mg, 0.020 mmol, 0.10 equiv.) was added to the crude reaction mixture follwed by addition of 2 mL acetonitrile. The reaction was allowed to stir in open air at room temperature for 3 h. After that, the reaction mixture

was passed through a small celite plug. The mixture was then concentrated and purified with flash column chromatography with pre-basified silica gel with Et_3N.

Note: Ir(dFppy)$_3$ (1.53 mg, 2.00 µmol, 0.0100 equiv) has been used as sensitizer under 450 nm LEDs instead of ITX for the cycloadditions of free amines.

5.3 [2+2] Cycloadditions Products Characterization:

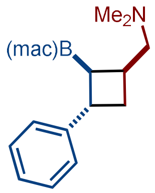

1-(2-(6b,9a-dimethyl-6b,9a-dihydroacenaphtho[1,2-d][1,3,2]dioxaborol-8-yl)-3-phenylcyclobutyl)-N,N-dimethylmethanamine (7): The title compound was prepared according to the general procedure **2** from styrenyl trifluoroborate salt **27** (84.3 mg, 0.280 mmol, 1.40 equiv) and *N,N*-dimethylallylamine **1** (24 µL, 0.20 mmol, 1.0 equiv.) The crude product was purified with pre basified (2% Et_3N in EtOAc) silica gel column chromatography (EtOAc) to afford the compound as a foamy pale yellow solid (68 mg, 0.17 mmol, 82% yield).
^{1}H NMR (500 MHz, CDCl$_3$): δ 7.76 – 7.67 (m, 2H), 7.57 (qd, *J* = 6.9, 1.5 Hz, 3H), 7.54 – 7.48 (m, 1H), 7.28 (t, *J* = 7.6 Hz, 2H), 7.21 (d, *J* = 7.5 Hz, 2H), 7.18 – 7.11 (m, 1H), 3.84 (q, *J* = 8.3 Hz, 1H), 2.73 (t, *J* = 9.9 Hz, 1H), 2.51 – 2.32 (m, 3H), 2.08 (ddd, *J* = 11.6, 8.6, 2.4 Hz, 1H), 1.95 (t, *J* = 8.6 Hz, 1H), 1.70 - 1.65 (m, 6H), 1.56 (s, 6H).
^{13}C NMR (126 MHz, CDCl$_3$): δ 149.1, 148.9, 147.8, 135.2, 131.1, 128.39, 128.35, 128.0, 126.5, 124.9, 124.2, 124.1, 119.0, 118.7, 89.93, 89.85, 65.0, 43.4, 37.3, 31.7, 29.0, 24.4, 23.1.
^{11}B NMR (160 MHz, CDCl$_3$): δ = 15.90.
HRMS: ESI (m/z): Calculated for $C_{27}H_{31}O_2NB$ $[M+H]^+$: 412.2442, found: 412.2452.
IR (neat): 2927, 1471, 1118, 1090, 782 cm^{-1}.

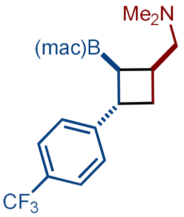

1-(2-(6b,9a-dimethyl-6b,9a-dihydroacenaphtho[1,2-d][1,3,2]dioxaborol-8-yl)-3-(4-(trifluoromethyl)phenyl)cyclobutyl)-N,N-dimethylmethanamine (8): The title compound was prepared according to the general procedure **2** from corresponding styrenyl trifluoroborate salt **S17** (104 mg, 0.280 mmol, 1.40 equiv) and *N,N*-dimethylallylamine **1** (24.0 µL, 0.20 mmol, 1.00 equiv.) The crude product was purified with pre basified (2% Et_3N in EtOAc) silica gel column chromatography (EtOAc) to afford the compound as a white solid (83 mg, 0.17 mmol, 86% yield).
^{1}H NMR (500 MHz, CDCl$_3$): δ 7.85 – 7.62 (m, 2H), 7.59 – 7.50 (m, 3H), 7.51 – 7.43 (m, 3H), 7.19 (d, *J* = 8.0 Hz, 2H), 3.83 (q, *J* = 8.4 Hz, 1H), 2.92 – 2.57 (m, 1H), 2.45

(td, J = 7.7, 5.2 Hz, 2H), 2.33 (dt, J = 11.9, 8.1 Hz, 1H), 2.09 (ddd, J = 10.8, 8.3, 2.1 Hz, 1H), 1.92 – 1.83 (m, 1H), 1.69 – 1.60 (m, 12H).
^{13}C NMR (126 MHz, $CDCl_3$): δ = 153.0, 148.9, 147.7, 135.1, 131.1, 128.4, 128.3, 127.1 (q, J= 32.8 Hz), 126.6, 124.9 (q, J = 3.8 Hz), 124.6 (q, J = 272.2 Hz), 124.2, 124.1, 118.9, 118.7, 89.9, 89.8, 64.9, 43.5, 37.3, 31.4, 28.9, 24.3, 23.2.
^{19}F NMR (471 MHz, $CDCl_3$): δ -62.04.
HRMS: ESI (m/z): Calculated for $C_{28}H_{30}O_2NBF_3$ $[M+H]^+$: 480.2316, found- 480.2322.
IR (neat): 2930, 1616, 1324, 1117, 781 cm^{-1}.

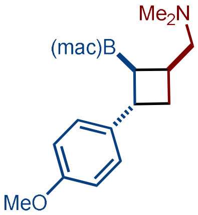

1-(2-(6b,9a-dimethyl-6b,9a-dihydroacenaphtho[1,2-d][1,3,2]dioxaborol-8-yl)-3-(4-methoxyphenyl)cyclobutyl)-N,N-dimethylmethanamine (9): The title compound was prepared according to the general procedure **2** from corresponding styrenyl trifluoroborate salt **S18** (93.1 mg, 0.280 mmol, 1.40 equiv) and *N,N*-dimethylallylamine **1** (24 µL, 0.20 mmol, 1.0 equiv.) The crude product was purified with pre basified (2% Et_3N in EtOAc) silica gel column chromatography (EtOAc) to afford the compound as a foamy pale yellow solid (53 mg, 0.17 mmol, 60% yield).
^{1}H NMR (500 MHz, $CDCl_3$): δ 7.70 (dd, J = 7.4, 1.9 Hz, 2H), 7.61 – 7.52 (m, 3H), 7.50 (d, J = 6.7 Hz, 1H), 7.11 (d, J = 8.3 Hz, 2H), 6.86 – 6.79 (m, 2H), 3.80 (s, 3H), 3.75 (t, J = 8.3 Hz, 1H), 2.71 (t, J = 9.6 Hz, 1H), 2.41 (dq, J = 14.5, 7.9 Hz, 2H), 2.30 (dt, J = 11.7, 8.3 Hz, 1H), 2.04 (dd, J = 11.6, 8.8 Hz, 1H), 1.88 (t, J = 8.7 Hz, 1H), 1.69 – 1.61 (m, 6H), 1.54 (s, 6H).
^{13}C NMR (126 MHz, $CDCl_3$): δ = 157.2, 149.1, 147.8, 141.1, 135.1, 131.1, 128.4, 128.3, 127.4, 124.2, 124.1, 119.0, 118.7, 113.4, 89.9, 89.8, 65.0, 55.3, 43.4, 36.7, 31.9, 28.9, 24.3, 23.0.
HRMS: ESI (m/z): Calculated for $C_{28}H_{33}O_3NB$ $[M+H]^+$: 442.2548, found: 442.2555.
IR (neat): 2928, 1609, 1510, 1087, 783 cm^{-1}.

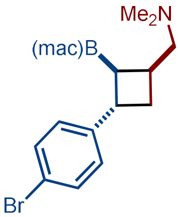

1-(3-(4-bromophenyl)-2-(6b,9a-dimethyl-6b,9a-dihydroacenaphtho[1,2-d][1,3,2]dioxaborol-8-yl)cyclobutyl)-N,N-dimethylmethanamine (10): The title compound was prepared according to the general procedure **2** from corresponding styrenyl trifluoroborate salt **S19** (107 mg, 0.280 mmol, 1.40 eq) and *N,N*-dimethylallylamine **1** (24 µL, 0.20 mmol, 1.0 equiv.) The crude product was purified with pre basified (2% Et_3N in EtOAc) silica gel column chromatography (EtOAc) to afford the compound as a white solid (77 mg, 0.16 mmol, 78% yield).

^{1}H NMR (500 MHz, CDCl$_3$): δ 7.70 (dd, *J* = 8.4, 2.8 Hz, 2H), 7.61 – 7.46 (m, 4H), 7.34 (d, *J* = 8.4 Hz, 2H), 6.99 (d, *J* = 8.3 Hz, 2H), 3.73 (q, *J* = 8.3 Hz, 1H), 2.77 – 2.68 (m, 1H), 2.43 (qd, *J* = 7.2, 4.3 Hz, 2H), 2.28 (dt, *J* = 11.9, 8.2 Hz, 1H), 2.09 – 2.01 (m, 1H), 1.87 – 1.80 (m, 1H), 1.66 (s, 3H), 1.63 (s, 3H), 1.59 (s, 6H).
^{13}C NMR (126 MHz, CDCl$_3$): δ 148.8, 147.8, 147.6, 135.0, 131.1, 130.9, 128.39, 128.36, 128.2, 124.3, 124.2, 122.0, 119.0, 118.7, 118.5, 89.9, 89.8, 65.0, 43.5, 36.9, 31.5, 28.8, 24.3, 23.1.
HRMS: ESI (m/z): Calculated for $C_{27}H_{30}O_2NBBr$ [M+H]$^+$: 490.1547, found: 490.1554.
IR (neat): 2927, 1486, 1118, 1086, 781 cm^{-1}.

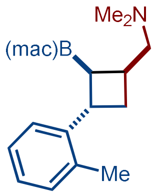

1-(2-(6b,9a-dimethyl-6b,9a-dihydroacenaphtho[1,2-d][1,3,2]dioxaborol-8-yl)-3-(o-tolyl)cyclobutyl)-N,N-dimethylmethanamine (11): The title compound was prepared according to the general procedure **2** from corresponding styrenyl trifluoroborate salt **S20** (88.6 mg, 0.280 mmol, 1.40 equiv) and *N,N*-dimethylallylamine (24 µL, 0.20 mmol, 1.0 equiv.) The crude product was purified with pre basified (2% Et_3N in EtOAc) silica gel column chromatography (EtOAc) to afford the compound as a white solid (68 mg, 0.16 mmol, 80% yield).
^{1}H NMR (500 MHz, CDCl$_3$): δ 7.75 – 7.67 (m, 2H), 7.61 – 7.49 (m, 4H), 7.34 (d, *J* = 7.7 Hz, 1H), 7.19 (td, *J* = 7.3, 2.0 Hz, 1H), 7.13 – 7.05 (m, 2H), 3.88 (q, *J* = 8.0 Hz, 1H), 2.73 (t, *J* = 10.0 Hz, 1H), 2.51 – 2.36 (m, 2H), 2.29 – 2.15 (m, 5H), 2.10 (t, *J* = 8.7 Hz, 1H), 1.65 (d, *J* = 9.0 Hz, 6H), 1.55 (s, 6H).
^{13}C NMR (126 MHz, CDCl$_3$): δ = 149.1, 147.8, 146.1, 136.0, 135.2, 131.1, 129.7, 128.38, 128.35, 125.6, 125.5, 125.1, 124.3, 124.1, 119.0, 118.7, 90.0, 89.9, 65.1, 43.5, 35.2, 32.5, 28.7, 24.4, 23.0, 20.0.
HRMS: ESI (m/z): Calculated for $C_{28}H_{33}O_2NB$ [M+H]$^+$: 426.2599, found: 426.2608.
IR (neat): 2924, 1460, 1117, 782, 749 cm^{-1}.

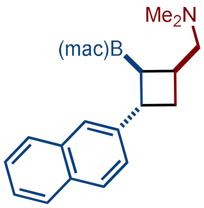

1-(2-(6b,9a-dimethyl-6b,9a-dihydroacenaphtho[1,2-d][1,3,2]dioxaborol-8-yl)-3-(naphthalen-2-yl)cyclobutyl)-N,N-dimethylmethanamine (12): The title compound was prepared according to the general procedure **2** from corresponding styrenyl trifluoroborate salt **S21** (98.8 mg, 0.280 mmol, 1.40 equiv) and *N,N*-dimethylallylamine (24 µL, 0.20 mmol, 1.0 equiv.) The crude product was purified with pre basified (2% Et_3N in EtOAc) silica gel column chromatography (EtOAc) to afford the compound as a white solid (76 mg, 0.16 mmol, 82% yield).

^{1}H NMR (500 MHz, $CDCl_3$): δ 7.82 – 7.73 (m, 3H), 7.70 (td, *J* = 7.6, 1.5 Hz, 2H), 7.61 – 7.48 (m, 5H), 7.46 – 7.36 (m, 2H), 7.34 (dd, *J* = 8.4, 1.8 Hz, 1H), 3.97 (q, *J* = 8.1 Hz, 1H), 2.78 (t, *J* = 9.6 Hz, 1H), 2.55 – 2.40 (m, 3H), 2.15 (dd, *J* = 11.1, 8.6 Hz, 1H), 2.04 (t, *J* = 8.4 Hz, 1H), 1.70 (s, 3H), 1.66 (s, 3H), 1.59 (s, 6H).
^{13}C NMR (126 MHz, $CDCl_3$): δ = 149.1, 147.8, 146.3, 135.1, 133.6, 131.7, 131.1, 128.4, 128.3, 127.6, 127.5, 127.5, 126.0, 125.6, 124.7, 124.2, 124.1, 123.9, 119.0, 118.7, 89.9, 89.8, 65.0, 43.5, 37.5, 31.5, 29.0, 24.4, 23.1.
HRMS: ESI (m/z): Calculated for $C_{31}H_{33}O_2NB$ $[M+H]^+$: 462.2599, found: 462.2606.
IR (neat): 2927, 1471, 1119, 1088, 783 cm^{-1}.

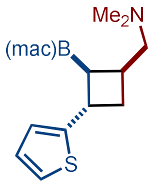

1-(2-(6b,9a-dimethyl-6b,9a-dihydroacenaphtho[1,2-d][1,3,2]dioxaborol-8-yl)-3-(thiophen-2-yl)cyclobutyl)-N,N-dimethylmethanamine (13): The title compound was prepared according to the general procedure **2** from corresponding styrenyl trifluoroborate salt **S22** (86.4 mg, 0.280 mmol, 1.40 equiv) and *N,N*-dimethylallylamine (24 µL, 0.20 mmol, 1.0 equiv.) The crude product was purified with pre basified (2% Et_3N in EtOAc) silica gel column chromatography (EtOAc) to afford the compound as a pale yellow solid (70 mg, 0.17 mmol, 84% yield).
^{1}H NMR (500 MHz, $CDCl_3$): δ 7.70 (d, *J* = 7.7 Hz, 2H), 7.61 – 7.47 (m, 4H), 7.11 (d, *J* = 5.1 Hz, 1H), 6.94 – 6.89 (m, 1H), 6.77 (d, *J* = 3.4 Hz, 1H), 3.99 (q, *J* = 8.1 Hz, 1H), 2.68 (t, *J* = 10.4 Hz, 1H), 2.48 (q, *J* = 8.3 Hz, 1H), 2.40 (dd, *J* = 11.3, 7.4 Hz, 2H), 2.13 (ddd, *J* = 11.6, 8.3, 2.6 Hz, 1H), 1.98 (d, *J* = 9.1 Hz, 1H), 1.70 (s, 3H), 1.62 (s, 3H), 1.52 (s, 6H).
^{13}C NMR (126 MHz, $CDCl_3$): δ = 154.1, 149.1, 147.8, 135.1, 131.1, 128.4, 128.3, 126.5, 124.2, 124.1, 122.2, 121.7, 119.0, 118.7, 90.0, 89.9, 64.8, 43.4, 33.6, 33.4, 28.8, 24.3, 23.3.
HRMS: ESI (m/z): Calculated for $C_{25}H_{29}O_2NBS$ $[M+H]^+$: 418.2007, found: 418.2015.
IR (neat): 2928, 1471, 1088, 1119, 783 cm^{-1}.

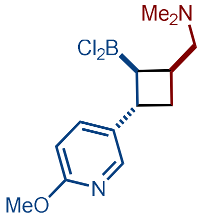

1-((1S,2R,3S)-2-(dichloroboraneyl)-3-(6-methoxypyridin-3-yl)cyclobutyl)-N,N-dimethylmethanamine (14): The title compound was prepared according to the general procedure **3** from corresponding potassium styrenyl trifluoroborate salt **S23** (48.2 mg, 0.200 mmol, 1.00 equiv.) using Ir(dFppy)$_3$ (1.53 mg, 2.00 µmol, 0.0100 equiv) as sensitizer under 450 nm LEDs (IPR, 24 h) instead of ITX. After the cycloadditions reaction, the crude reaction mixture was concentrated and the dichloroborane product was purified directly without additional Mac protection. The product was purified by

silica gel column chromatography (Et_3N: MeOH: DCM = 1:1:98) to afford the product as white solid (34 mg, 0.11 mmol, 56% yield).
^{1}H NMR (500 MHz, $CDCl_3$): δ 8.08 (d, *J* = 2.5 Hz, 1H), 7.54 (dd, *J* = 8.5, 2.5 Hz, 1H), 6.71 (d, *J* = 8.5 Hz, 1H), 3.92 (s, 3H), 3.77 (dt, *J* = 9.0, 6.0 Hz, 1H), 3.39 (dd, *J* = 11.4, 8.5 Hz, 1H), 3.10 (dd, *J* = 11.4, 7.9 Hz, 1H), 2.91 (dt, *J* = 8.8, 4.4 Hz, 1H), 2.88 (s, 3H), 2.60 (s, 3H), 2.43 – 2.19 (m, 3H).
^{13}C NMR (126 MHz, $CDCl_3$): δ 162.5, 144.6, 137.5, 135.7, 110.5, 68.1, 53.3, 48.0, 46.1, 35.7, 31.8, 29.8.
HRMS: (m/z): Calculated for $C_{13}H_{20}ON_2BCl_2$ [M+H]$^+$: 301.1040, found: 301.1041.
IR (neat): 2942, 1605, 1491, 1283, 1023, 773 cm^{-1}.

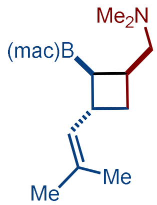

1-((1S,2R,3S)-2-(6b,9a-dimethyl-6b,9a-dihydroacenaphtho[1,2-d][1,3,2]dioxaborol-8-yl)-3-(2-methylprop-1-en-1-yl)cyclobutyl)-N,N-dimethylmethanamine (15): The title compound was prepared according to the general procedure **2** from corresponding styrenyl trifluoroborate salt **S24** (78.5 mg, 0.280 mmol, 1.40 equiv) and *N,N*-dimethylallylamine (24 μL, 0.20 mmol, 1.0 equiv.) The crude product was purified with pre basified (2% Et_3N in EtOAc) silica gel column chromatography (Et_3N: hexanes: EtOAc = 1: 50: 50) to afford the compound as a pale yellow solid (49 mg, 0.13 mmol, 63% yield).
^{1}H NMR (500 MHz, $CDCl_3$): δ 7.79 – 7.65 (m, 2H), 7.62 – 7.51 (m, 3H), 7.49 (d, *J* = 6.7 Hz, 1H), 5.35 – 5.28 (m, 1H), 3.33 (p, *J* = 7.8 Hz, 1H), 2.55 (t, *J* = 10.3 Hz, 1H), 2.35 (pd, *J* = 8.1, 3.6 Hz, 1H), 2.26 (dd, *J* = 11.0, 7.3 Hz, 1H), 1.93 (dt, *J* = 12.1, 7.9 Hz, 1H), 1.84 (ddd, *J* = 11.7, 8.3, 2.9 Hz, 1H), 1.68 (d, *J* = 4.6 Hz, 6H), 1.64 (s, 3H), 1.61 (s, 3H), 1.57 (dd, *J* = 9.4, 7.1 Hz, 1H), 1.44 (s, 6H).
^{13}C NMR (126 MHz, $CDCl_3$): δ = 149.2, 147.9, 135.3, 133.5, 131.2, 128.9, 128.5, 128.4, 124.3, 124.2, 119.1, 118.8, 90.00, 89.97, 65.3, 43.5, 32.4, 31.4, 29.1, 25.9, 24.4, 23.0, 18.5.
HRMS: ESI (m/z): Calculated for $C_{25}H_{33}O_2NB$ [M+H]$^+$: 390.2599, found: 390.2605.
IR (neat): 2924, 1471, 1118, 1084, 781 cm^{-1}.

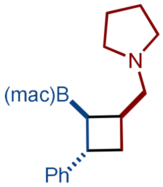

1-((2-(6b,9a-dimethyl-6b,9a-dihydroacenaphtho[1,2-d][1,3,2]dioxaborol-8-yl)-3-phenylcyclobutyl)methyl)pyrrolidine (16): The title compound was prepared according to the general procedure **2** from styrenyl trifluoroborate salt **27** (84.3 mg, 0.280 mmol, 1.40 equiv) and 1-allylpyrrolidine **S25** (22.2 mg, 0.200 mmol, 1.00 equiv.)

The crude product was purified with pre basified (2% Et_3N in EtOAc) silica gel column chromatography (3% Et_3N in EtOAc) to afford the compound as a pale yellow solid (65 mg, 0.15 mmol, 74% yield).

^{1}H NMR (500 MHz, CDCl$_3$): δ 7.78 – 7.73 (m, 2H), 7.64 – 7.54 (m, 3H), 7.53 – 7.47 (m, 1H), 7.23 (t, *J* = 7.5 Hz, 2H), 7.11 (t, *J* = 7.0 Hz, 3H), 3.58 (q, *J* = 7.8 Hz, 1H), 2.75 (d, *J* = 8.8 Hz, 2H), 2.70 – 2.55 (m, 3H), 2.41 (s, 2H), 2.33 (dt, *J* = 12.0, 8.0 Hz, 1H), 2.21 (ddd, *J* = 12.0, 8.7, 4.7 Hz, 1H), 1.99 (t, *J* = 8.5 Hz, 1H), 1.72 (s, 3H), 1.68 (s, 3H), 1.61 (d, *J* = 7.9 Hz, 2H), 1.46 (s, 2H).

^{13}C NMR (126 MHz, CDCl$_3$): δ 146.7, 146.5, 146.3, 134.5, 131.3, 128.7, 128.5, 128.2, 126.3, 125.5, 124.9, 124.7, 119.3, 119.2, 90.89 (2*C*, overlap), 60.7, 53.5, 37.7, 31.8, 29.5, 23.4, 23.0, 22.7.

HRMS: ESI: (m/z): Calculated for $C_{29}H_{33}O_2NB$ [M+H]$^+$: 438.2599, found: 438.2607.

IR (neat): 2927, 1639, 1376, 1063, 783 cm^{-1}.

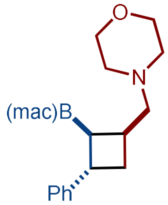

4-((2-(6b,9a-dimethyl-6b,9a-dihydroacenaphtho[1,2-d][1,3,2]dioxaborol-8-yl)-3-phenylcyclobutyl)methyl)morpholine (18): The title compound was prepared according to the general procedure **2** from styrenyl trifluoroborate salt **27** (84.3 mg, 0.280 mmol, 1.40 equiv) and 1-allylmorpholine **S26** (25.4 mg, 0.200 mmol, 1.00 equiv.) The crude product was purified with pre basified (2% Et_3N in EtOAc) silica gel column chromatography (3% Et_3N in EtOAc) to afford the compound as a pale yellow solid (78 mg, 0.17 mmol, 86% yield).

^{1}H NMR (500 MHz, CDCl$_3$): δ 7.78 (td, *J* = 7.4, 1.3 Hz, 2H), 7.61 – 7.59 (m, 1H), 7.58-7.56 (m, 3H), 7.27 – 7.21 (m, 2H), 7.20 – 7.16 (m, 2H), 7.15 – 7.11 (m, 1H), 3.69 (q, *J* = 8.3 Hz, 1H), 3.13 (ddd, *J* = 11.4, 6.6, 2.9 Hz, 2H), 2.90 (t, *J* = 9.1 Hz, 2H), 2.58 (dtd, *J* = 14.0, 7.9, 4.4 Hz, 1H), 2.43 – 2.27 (m, 2H), 2.18 – 1.99 (m, 5H), 1.79 (s, 3H), 1.76 (d, *J* = 8.0 Hz, 5H).

^{13}C NMR (126 MHz, CDCl$_3$): δ 146.9, 145.39, 145.35, 134.8, 131.5, 128.6, 128.4, 128.2, 126.3, 125.5, 125.23, 125.15, 119.5, 119.4, 91.71, 91.67, 66.0, 61.7, 53.4, 53.0, 38.1, 32.1, 29.6, 22.6.

HRMS: ESI (m/z): Calculated for $C_{29}H_{33}O_3NB$ [M+H]$^+$: 454.2548, found: 454.2557.

IR (neat): 2958, 1372, 1116, 1077, 780 cm^{-1}.

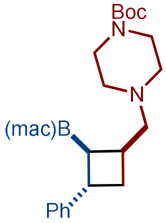

Tert-butyl 4-((2-(6b,9a-dimethyl-6b,9a-dihydroacenaphtho[1,2-d][1,3,2]dioxaborol-8-yl)-3-phenylcyclobutyl)methyl)piperazine-1-carboxylate (18): The title compound was prepared according to the general procedure **2** from

styrenyl trifluoroborate salt **27** (84.3 mg, 0.280 mmol, 1.40 equiv) and tert-butyl 4-allylpiperazine-1-carboxylate **S27** (45.3 mg, 0.200 mmol, 1.00 equiv.) The crude product was purified with pre basified (EtOAc: hexane: Et_3N = 50:50:3) silica gel column chromatography (3% Et_3N in EtOAc) to afford the compound as a pale yellow solid (97 mg, 0.18 mmol, 88% yield).
^{1}H NMR (500 MHz, $CDCl_3$): δ 7.78 (t, *J* = 8.1 Hz, 2H), 7.63 – 7.48 (m, 4H), 7.23 (d, *J* = 7.5 Hz, 2H), 7.18 – 7.10 (m, 3H), 3.67 (q, *J* = 8.0 Hz, 1H), 2.87 (s, 2H), 2.70 – 2.53 (m, 3H), 2.42 – 2.27 (m, 2H), 2.16 (dd, *J* = 11.9, 6.9 Hz, 1H), 1.96 – 2.10 (m, 4H), 1.81 – 1.69 (m, 8H), 1.46 (d, *J* = 3.1 Hz, 1H), 1.44 (s, 9H).
^{13}C NMR (126 MHz, $CDCl_3$): δ 154.6, 146.8, 145.3, 144.5, 134.8, 131.4, 128.49, 128.47, 128.4, 128.2, 126.3, 125.5, 125.2, 119.41, 119.35, 91.68, 91.65, 79.3, 61.0, 52.2, 38.0, 32.2, 29.9, 28.5, 22.6, 22.5, 22.1.
HRMS: ESI (m/z): Calculated for $C_{34}H_{42}O_4N_2B$ $[M+H]^+$: 553.3232, found: 553.3241.
IR (neat): 2930, 1689, 1171, 1116, 1004, 779 cm^{-1}.

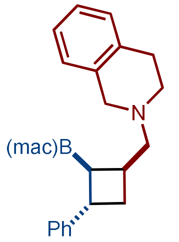

2-((2-(6b,9a-dimethyl-6b,9a-dihydroacenaphtho[1,2-d][1,3,2]dioxaborol-8-yl)-3-phenylcyclobutyl)methyl)-1,2,3,4-tetrahydroisoquinoline (19): The title compound was prepared according to the general procedure **2** from styrenyl trifluoroborate salt **27** (84.3 mg, 0.280 mmol, 1.40 equiv) and 2-allyl-1,2,3,4-tetrahydroisoquinoline **S28** (34.7 mg, 0.200 mmol, 1.00 equiv.) The crude product was purified with pre basified (EtOAc: hexane: Et_3N = 50:50:3) silica gel column chromatography (3% Et_3N in EtOAc) to afford the compound as a pale yellow solid (78 mg, 0.16 mmol, 78% yield).
^{1}H NMR (500 MHz, $CDCl_3$): δ 7.71 (dp, *J* = 5.9, 1.8 Hz, 1H), 7.65 (dt, *J* = 6.8, 1.6 Hz, 1H), 7.61 – 7.55 (m, 2H), 7.51 – 7.44 (m, 2H), 7.26 – 7.22 (m, 2H), 7.19 – 7.10 (m, 3H), 7.09 – 7.04 (m, 2H), 6.94 (d, *J* = 7.2 Hz, 1H), 6.73 (d, *J* = 7.2 Hz, 1H), 3.82 (q, *J* = 8.3 Hz, 1H), 3.42 – 3.29 (m, 1H), 3.03 (d, *J* = 16.0 Hz, 1H), 2.74 (q, *J* = 6.2 Hz, 1H), 2.65 – 2.32 (m, 6H), 2.13 – 2.03 (m, 1H), 1.98 (t, *J* = 8.6 Hz, 2H), 1.69 (dd, *J* = 2.9, 1.4 Hz, 6H).
^{13}C NMR (126 MHz, $CDCl_3$): δ 148.4, 147.7, 147.2, 134.9, 132.6, 132.2, 131.2, 128.5, 128.3, 128.0, 127.1, 126.59, 126.57, 126.4, 126.2, 125.1, 124.5, 124.4, 119.1, 118.8, 90.41, 90.40, 56.4, 52.5, 47.0, 37.6, 31.8, 29.1, 24.7, 23.6, 23.0.
HRMS: ESI (m/z): Calculated for $C_{34}H_{35}O_2NB$ $[M+H]^+$: 500.2755, found: 500.2763.
IR (neat): 2929, 1602, 1463, 1114, 1082, 827 cm^{-1}.

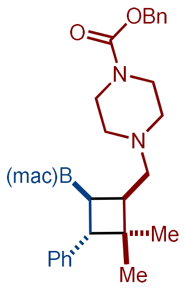

benzyl 4-((-4-(6b,9a-dimethyl-6b,9a-dihydroacenaphtho[1,2-d][1,3,2]dioxaborol-8-yl)-2,2-dimethyl-3-phenylcyclobutyl)methyl)piperazine-1-carboxylate (20): The title compound was prepared according to the general procedure **2** from styrenyl trifluoroborate salt **27** (84.3 mg, 0.280 mmol, 1.40 equiv) and benzyl 4-(3-methylbut-2-en-1-yl)piperazine-1-carboxylate,**S29** (58 mg, 0.20 mmol, 1.0 equiv.) with Ir(dFppy)$_3$ (1.53 mg, 2.00 µmol, 0.0100 equiv) as sensitizer under 450 nm LEDs (IPR) for 24 h. The crude product was purified with silica gel column chromatography (hexane: EtOAc = 5:2) to afford the compound as a white solid (64 mg, 0.10 mmol, 52% yield).

^{1}H NMR (500 MHz, CDCl$_3$): δ 7.72 (dd, *J* = 8.0, 1.0 Hz, 1H), 7.64 (dd, *J* = 6.9, 2.0 Hz, 1H), 7.57 (dd, *J* = 8.0, 6.9 Hz, 1H), 7.54 – 7.46 (m, 3H), 7.44 – 7.38 (m, 2H), 7.35 (dt, *J* = 5.7, 1.4 Hz, 3H), 7.30 – 7.24 (m, 2H), 7.20 – 7.13 (m, 1H), 7.10 – 7.05 (m, 2H), 5.11 (d, *J* = 12.5 Hz, 1H), 5.05 (d, *J* = 12.5 Hz, 1H), 3.46 (d, *J* = 10.1 Hz, 1H), 2.73 (ddd, *J* = 13.3, 7.1, 3.1 Hz, 2H), 2.50 – 2.40 (m, 2H), 2.33 (s, 1H), 2.21 (t, *J* = 10.1 Hz, 1H), 2.14 – 1.89 (m, 4H), 1.72 (s, 3H), 1.65 (s, 5H), 1.09 (s, 3H), 0.69 (s, 3H).

^{13}C NMR (126 MHz, CDCl$_3$): δ 155.1, 145.5, 145.4, 142.7, 137.1, 135.0, 131.5, 128.6, 128.6, 128.5, 128.1, 128.98, 127.95, 127.4, 125.7, 125.33, 125.25, 119.5, 119.5, 91.73, 91.71, 66.9, 56.9, 52.1, 48.3, 42.5, 40.4, 40.3, 25.6, 24.6, 22.9, 22.8.

HRMS: ESI (m/z): Calculated for $C_{39}H_{44}O_4N_2B$ [M+H]$^+$: 615.3389, found: 615.3386.

IR (neat): 2952, 1701, 1377, 1240, 1117, 825 cm^{-1}.

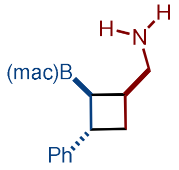

(2-(6b,9a-dimethyl-6b,9a-dihydroacenaphtho[1,2-d][1,3,2]dioxaborol-8-yl)-3-phenylcyclobutyl)methanamine (21): The title compound was prepared according to the general procedure **2** using Ir(dFppy)$_3$ (1.53 mg, 2.00 µmol, 0.0100 equiv) as sensitizer instead of ITX, under blue LED irradiation (IPR, 14h) from styrenyl trifluoroborate salt **27** (84.3 mg, 0.280 mmol, 1.400 equiv) and prop-2-en-1-amine (11.5 mg, 0.200 mmol, 1.00 equiv.) The crude product was purified with pre-basified silica gel first with (hexane: EtOAc: Et_3N = 50:50:3) then with MeOH: DCM: Et_3N (5:95:3). Colorless solid (45 mg, 0.12 mmol, 58% yield).

^{1}H NMR (500 MHz, CDCl$_3$): δ 7.76 – 7.70 (m, 2H), 7.60 – 7.52 (m, 4H), 7.29 – 7.20 (m, 4H), 7.16 – 7.09 (m, 1H), 3.92 (b, 2H), 3.47 (q, *J* = 7.6 Hz, 1H), 2.72 (dd, *J* = 11.7, 5.9 Hz, 1H), 2.60 (d, *J* = 13.7 Hz, 2H), 2.26 (dt, *J* = 12.7, 8.0 Hz, 1H), 2.00 – 1.90 (m, 1H), 1.82 (t, *J* = 7.9 Hz, 1H), 1.67 (d, *J* = 13.8 Hz, 6H).

^{13}C NMR (126 MHz, CDCl$_3$): δ 148.1, 147.4, 147.1, 134.7, 131.3, 128.7 (2*C*, overlap), 128.1, 126.4, 125.3, 124.7, 124.6, 119.3, 119.0, 90.1, 89.7, 45.3, 37.2, 32.6, 31.7, 23.1, 22.6.
HRMS: ESI (m/z): Calculated for $C_{25}H_{27}O_2NB$ [M+H]$^+$: 384.2129, found: 384.2133.
IR (neat): 2928, 1374, 1116, 1086, 781 cm^{-1}.

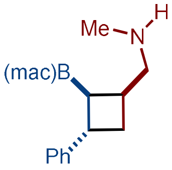

1-(2-(6b,9a-dimethyl-6b,9a-dihydroacenaphtho[1,2-d][1,3,2]dioxaborol-8-yl)-3-phenylcyclobutyl)-N-methylmethanamine (22): The title compound was prepared according to the general procedure **2** using Ir(dFppy)$_3$ (1.53 mg, 2.00 μmol, 0.0100 equiv) as sensitizer instead of ITX, under blue LED irradiation (IPR, 14h) from styrenyl trifluoroborate salt **27** (84.3 mg, 0.280 mmol, 1.40 equiv) and N-methylprop-2-en-1-amine **39** (14.3 mg, 0.200 mmol, 1.00 equiv.) The crude product was purified with pre-basified silica gel first with (hexane: EtOAc: Et$_3$N = 50:50:3) then with MeOH: DCM: Et$_3$N (5:95:3). (69 mg, 0.17 mmol, 86% yield).
^{1}H NMR (500 MHz, CDCl$_3$): δ 7.76 – 7.69 (m, 2H), 7.56 (td, *J* = 7.3, 5.6 Hz, 3H), 7.53 – 7.47 (m, 1H), 7.29 (d, *J* = 7.7 Hz, 2H), 7.21 (d, *J* = 7.5 Hz, 2H), 7.17 – 7.10 (m, 1H), 3.64 (q, *J* = 7.9 Hz, 1H), 2.73 (dd, *J* = 11.0, 5.5 Hz, 1H), 2.65 – 2.54 (m, 2H), 2.39 (dt, *J* = 12.4, 8.1 Hz, 1H), 2.04 (ddd, *J* = 12.1, 8.9, 3.2 Hz, 1H), 1.78 (t, *J* = 8.1 Hz, 1H), 1.67 (s, 6H), 1.51 (s, 3H).
^{13}C NMR (126 MHz, CDCl$_3$): δ 149.0, 148.4, 148.2, 134.8, 131.2, 128.51, 128.48, 128.0, 126.4, 125.0, 124.3, 124.2, 118.9, 118.8, 89.6, 89.2, 55.8, 37.1, 32.9, 31.7, 30.3, 23.5, 23.4.
HRMS: ESI (m/z): Calculated for $C_{26}H_{29}O_2NB$ [M+H]$^+$: 398.2286, found: 398.2290.
IR (neat): 2926, 1494, 1116, 1082, 781 cm^{-1}.

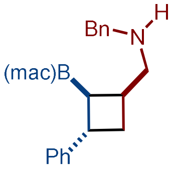

N-benzyl-1-(2-(6b,9a-dimethyl-6b,9a-dihydroacenaphtho[1,2-d][1,3,2]dioxaborol-8-yl)-3-phenylcyclobutyl)methanamine [SI-XX]: The title compound was prepared according to the general procedure **3** [BCl$_3$ condition] using Ir(dFppy)$_3$ (1.53 mg, 2.00 μmol, 0.01 equiv) as sensitizer instead of ITX, under blue LED (450 nm) irradiation (IPR, 14h) from styrenyl trifluoroborate salt **27** (60.2 mg, 0.200 mmol, 1.00 equiv) and N-benzylprop-2-en-1-amine **S30** (59 mg, 0.40 mmol, 2.0 equiv.) The crude product was purified with pre-basified silica gel first with (first with MeOH: Acetone: DCM= 2:2:96, to get a mixture of mac-OH and the pdt, then another column with 50% EtOAc in hexanes). Colorless solid (52 mg, 0.11 mmol, 55% yield).
^{1}H NMR (500 MHz, CDCl$_3$): δ 7.78 (td, *J* = 6.3, 3.5 Hz, 2H), 7.65 – 7.55 (m, 4H), 7.30 (t, *J* = 7.5 Hz, 2H), 7.25 (d, *J* = 8.5 Hz, 2H), 7.20 – 7.10 (m, 4H), 6.35 (d, *J* = 7.1 Hz,

2H), 3.77 (q, J = 8.1 Hz, 1H), 2.89 (d, J = 14.1 Hz, 1H), 2.77 (d, J = 14.1 Hz, 1H), 2.53 (dt, J = 12.5, 6.1 Hz, 3H), 2.38 (dt, J = 12.5, 8.0 Hz, 1H), 2.02 (ddd, J = 11.6, 8.6, 2.5 Hz, 1H), 1.91 (t, J = 8.0 Hz, 1H), 1.72 (d, J = 11.9 Hz, 6H).
^{13}C NMR (126 MHz, $CDCl_3$): δ = 148.8, 148.4, 147.9, 135.8, 134.9, 131.4, 128.9, 128.8, 128.7, 128.5, 128.0, 127.8, 126.4, 125.0, 124.5, 124.4, 119.2, 119.1, 89.7, 89.3, 50.0, 49.5, 37.1, 31.7, 30.0, 23.5, 23.1.
HRMS: ESI (m/z): Calculated for $C_{32}H_{33}O_2NB$ $[M+H]^+$: 474.2599, found: 474.2606.
IR (neat): 2926, 1601, 1494, 1115, 1085, 781 cm^{-1}.

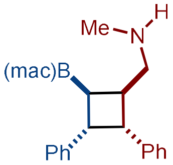

1-(2-(6b,9a-dimethyl-6b,9a-dihydroacenaphtho[1,2-d][1,3,2]dioxaborol-8-yl)-3,4-diphenylcyclobutyl)-N-methylmethanamine (24): The title compound was prepared according to the general procedure **2** using 1mol % [Ir(dFppy)$_3$] as photosensitizer instead of ITX, under blue LED irradiation (IPR, 14h) from styrenyl trifluoroborate salt **27** (84.3 mg, 0.280 mmol, 1.40 equiv) and (E)-N-methyl-3-phenylprop-2-en-1-amine **S32** (29.4 mg, 0.200 mmol, 1.00 equiv.). ^{1}H NMR analysis of the crude reaction mixture shows 57% NMR yield, 4:1 dr. The crude product was purified with pre-basified silica gel first with (hexane: EtOAc: Et_3N = 50:50:3) then with MeOH: DCM: Et_3N (5:95:3). White solid (36 mg, 72 μmol, 36% yield, >20:1 dr, major diastereomer).
^{1}H NMR (500 MHz, $CDCl_3$): δ 7.78 – 7.70 (m, 2H), 7.59 (dd, J = 4.9, 3.4 Hz, 2H), 7.57 – 7.49 (m, 2H), 7.09 – 6.91 (m, 8H), 6.89 – 6.84 (m, 2H), 4.06 (dd, J = 9.5, 5.5 Hz, 1H), 3.63 (dd, J = 9.5, 6.7 Hz, 1H), 3.04 (ddt, J = 16.7, 11.5, 6.6 Hz, 1H), 2.78 (dd, J = 11.9, 4.7 Hz, 1H), 2.68 (dd, J = 11.8, 6.5 Hz, 1H), 2.12 – 1.95 (m, 1H), 1.68 (d, J = 7.7 Hz, 6H), 1.54 (s, 3H).
^{13}C NMR (126 MHz, $CDCl_3$): δ = 148.2, 147.9, 143.6, 141.4, 134.9, 131.2, 128.53, 128.51, 128.3, 128.0, 127.6, 127.4, 125.4, 124.9, 124.4, 124.3, 119.0, 118.9, 89.9, 89.6, 55.1, 48.0, 43.0, 37.9, 33.4, 23.4, 23.3.
HRMS: (m/z): Calculated for $C_{32}H_{33}O_2NB$ $[M+H]^+$: 474.2599, found: 474.2606.
IR (neat): 2927, 1601, 1118, 1081, 782 cm^{-1}.

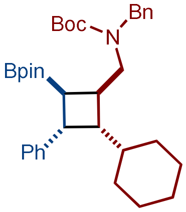

tert-butyl benzyl((2-cyclohexyl-3-phenyl-4-(4,4,5,5-tetramethyl-1,3,2-dioxaborolan-2-yl)cyclobutyl)methyl)carbamate (25): In a 2-dram vial with a stir bar inside was charged styrenyl trifluoroborate salt **27** (60.2 mg, 0.200 mmol, 1.00 equiv)and [Ir(dFppy)$_3$] (1.53 mg, 2.00 μmol, 0.0100 equiv). The vial was degassed and backfilled with N_2 (X 3). Then 2 mL of DCM was added under N_2 atmosphere followed

by the addition of a solution of BCl_3 (1M in CH_2Cl_2) (0.24 mL, 0.24 mmol, 1.2 equiv) dropwise at room temperature under N_2 atmosphere and stirred it for 10 min at rt. Then with a syringe, allyl amine **S31** (91 mg, 0.40 mmol, 2.0 equiv) was added to the reaction vial dropwise under N_2 atmosphere, and the septum was quickly replaced with plastic cap and sealed with teflon tape. The reaction was allowed to be irradiated under 450 nm LEDs in IPR for 18h. After that, all the volatiles were removed in the rotovap and the crude reaction mixture was analyzed by ^{1}H-NMR. Crude NMR analysis shows 81% NMR yield of the cycloadduct, >20:1 dr.

After evaporating all the volatiles, to the crude mixture, with a stir bar inside, pinacol (35 mg, 0.30 mmol, 1.5 equiv) and anhyd. K_2CO_3 (41 mg, 0.30 mmol, 1.5 equiv) were added, and 2.0 mL anhyd. THF was added to the reaction mixture and the vial was capped properly with a plastic cap and sealed with Teflon tape. The reaction mixture was heated to reflux overnight (14 h). After that, the crude reaction mixture was passed through a celite pad to remove the solid. The filtrate was concentrated and in vacuo.

To the reaction mixture was added Boc_2O (0.22 g, 1.0 mmol, 5.0 equiv.) and DMAP (12 mg, 0.10 mmol, 0.50 equiv.) and the reaction was capped with a septum. Then under N_2 atmosphere 2 mL anhyd. DCM was added to the mixture through a syringe. Then at 0 ^{0}C (ice bath) slowly added DIPEA (0.17 mL, 1.0 mmol, 5.0 equiv.) with a syringe. After half an hour the ice bath was removed and the reaction was allowed to stir at rt for overnight.

After the reaction time period the reaction was quenched with slow addition of sat. aq. $NaHCO_3$ solution (2 mL) at 0 ^{0}C (ice bath). The organic layer was separated, and the aqueous layer was extracted 3 times with ethyl acetate. The combined organic layer was washed with brine and dried over MgSO4 followed by concentrated and purified with FCC (3% EtOAc in hexanes). Pale yellow oil (66 mg, 0.12 mmol, 59% yield.)

^{1}H NMR (500 MHz, $CDCl_3$ @ 50 ^{0}C): δ 7.34 (t, *J* = 7.6 Hz, 2H), 7.26 (ddd, *J* = 13.4, 8.9, 6.3 Hz, 7H), 7.21 – 7.15 (m, 1H), 4.56 (s, 1H), 4.44 (d, *J* = 15.8 Hz, 1H), 3.71 (dd, *J* = 14.1, 7.8 Hz, 1H), 3.62 (dd, *J* = 9.4, 6.1 Hz, 1H), 3.46 – 3.23 (m, 1H), 2.85 – 2.74 (m, 1H), 2.30 (q, *J* = 9.1 Hz, 1H), 2.11 (dd, *J* = 10.4, 6.0 Hz, 1H), 1.64 – 1.55 (m, 3H), 1.49 (s, 10H), 1.32 – 1.23 (m, 1H), 1.20 (d, *J* = 4.0 Hz, 12H), 1.11 – 1.00 (m, 3H), 0.78 (td, *J* = 14.0, 10.6 Hz, 2H), 0.65 – 0.53 (m, 1H).

^{13}C NMR (126 MHz, $CDCl_3$, @ 50 ^{0}C): δ = 156.2, 143.6, 128.8, 128.5, 128.2, 128.0, 127.4, 127.0, 125.9, 83.4, 79.6, 51.2, 49.5, 41.8, 38.5, 31.4, 29.9, 28.7, 26.8, 26.4, 26.0, 25.12, 25.05.

^{11}B NMR (160 MHz, $CDCl_3$): δ = 22.40.

HRMS: ESI (m/z): Calculated for $C_{35}H_{50}O_4NBNa$ $[M+Na]^+$: 582.3725, found: 582.3722.

IR (neat): 2923, 1690, 1160, 1142, 734, 698 cm^{-1}.

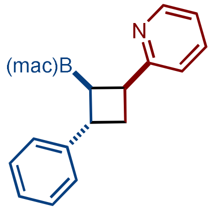

2-(2-(6b,9a-dimethyl-6b,9a-dihydroacenaphtho[1,2-d][1,3,2]dioxaborol-8-yl)-3-phenylcyclobutyl)pyridine (29): The title compound was prepared according to the general procedure **2** from styrenyl trifluoroborate salt **27** (84.3 mg, 0.280 mmol, 1.40 equiv) and 2-vinylpyridine **26** (21 mg, 0.20 mmol, 1.0 equiv) The crude product was purified with pre basified silica gel (with 3% Et_3N in hexane) column chromatography using (EtOAc: hexane: Et_3N = 30:70:3) as eluent to afford the compound as a pale yellow solid (78 mg, 0.16 mmol, 78% yield).

^{1}H NMR (500 MHz, $CDCl_3$): δ 7.82 – 7.71 (m, 3H), 7.64 – 7.56 (m, 2H), 7.53 (dd, *J* = 8.1, 6.9 Hz, 1H), 7.46 (dd, *J* = 7.0, 1.4 Hz, 1H), 7.33 (d, *J* = 7.9 Hz, 1H), 7.24 (t, *J* = 7.6 Hz, 2H), 7.16 – 7.08 (m, 4H), 6.96 (t, *J* = 6.6 Hz, 1H), 3.76 – 3.68 (m, 1H), 3.47 (q, *J* = 8.4 Hz, 1H), 2.82 (ddd, *J* = 11.9, 10.1, 8.8 Hz, 1H), 2.22 (ddd, *J* = 11.6, 8.4, 3.0 Hz, 1H), 2.01 (t, *J* = 7.9 Hz, 1H), 1.77 (s, 3H), 1.75 (s, 3H).

^{13}C NMR (126 MHz, $CDCl_3$): δ 165.7, 148.9, 148.4, 148.0, 141.4, 141.0, 135.0, 131.6, 128.7, 128.6, 128.1, 126.5, 125.1, 124.3, 124.2, 123.3, 123.1, 119.1, 119.1, 90.0, 89.6, 38.7, 37.0, 34.4, 24.2, 23.2.

^{11}B NMR (160 MHz, $CDCl_3$): δ = 16.32.

HRMS: ESI (m/z): Calculated for $C_{29}H_{27}BNO_2$ $[M+H]^+$: 432.2129, found: 432.2137.

IR (neat): 2926, 1616, 1482, 1197, 1087 cm^{-1}.

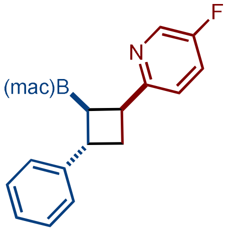

2-((1R,2S,3R)-2-(6b,9a-dimethyl-6b,9a-dihydroacenaphtho[1,2-d] [1,3,2]dioxaborol-8-yl)-3-phenylcyclobutyl)-5-fluoropyridine (30):

The title compound was prepared according to the general procedure **2** from styrenyl trifluoroborate salt **27** (84.3 mg, 0.280 mmol, 1.40 equiv) and 5-fluoro-2-vinylpyridine **S42** (24.6 mg, 0.200 mmol, 1.00 equiv.) The crude product was purified with pre basified (2% Et_3N in EtOAc) silica gel column chromatography (EtOAc) to afford the compound as a foamy white solid (67 mg, 0.15 mmol, 74% yield).

^{1}H NMR (500 MHz, $CDCl_3$): δ 7.81 (dd, *J* = 10.3, 8.1 Hz, 2H), 7.66 – 7.51 (m, 3H), 7.43 (d, *J* = 6.8 Hz, 1H), 7.37 – 7.31 (m, 1H), 7.28 (t, *J* = 7.7 Hz, 2H), 7.23 – 7.17 (m, 3H), 7.15 (d, *J* = 7.3 Hz, 1H), 6.57 (t, *J* = 2.3 Hz, 1H), 3.70 (td, *J* = 9.3, 2.9 Hz, 1H), 3.58 (q, *J* = 8.5 Hz, 1H), 2.79 (dt, *J* = 11.6, 9.4 Hz, 1H), 2.24 (ddd, *J* = 11.7, 8.5, 3.1 Hz, 1H), 2.17 (t, *J* = 8.4 Hz, 1H), 1.75 (s, 3H), 1.73 (s, 3H).

^{13}C NMR (126 MHz, $CDCl_3$): δ = 161.0, 158.5 (d, *J* = 254.5 Hz), 147.9, 147.5 (d, *J* = 3.8 Hz), 134.9, 131.6, 131.4, 131.1, 128.7, 128.6, 128.2, 127.4, 127.2, 126.5, 125.4, 124.8, 124.6, 123.7 (d, *J* = 5.0 Hz), 119.3 (d, *J* = 3.8 Hz), 90.6, 90.2, 38.5, 37.0, 34.5, 23.7, 22.8.

^{19}F NMR (471 MHz, CD_3CN): δ – 127.11.

HRMS: (ESI, m/z): Calculated for $C_{29}H_{26}BFNO_2$ $[M+H]^+$: 450.2035, found: 450.2041.

IR: 2928.61, 1736.54, 1491.98, 1373.06, 1112.89, 745.20 cm^{-1}.

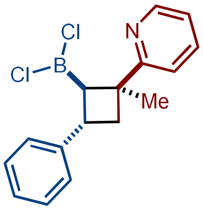

2-(dichloroboraneyl)-1-methyl-3-phenylcyclobutyl) pyridine (31):

The title compound was prepared according to the general procedure **3** [BCl_3 condition] from styrenyl trifluoroborate salt **27** (60.2 mg, 0.200 mmol, 1.00 equiv) and 2-(prop-1-en-2-yl) pyridine **S39** (47.6 mg, 0.400 mmol, 2.00 equiv.). After the cycloadditions reaction, the product was purified without additional protection step. The crude product was purified with pre basified (2% Et_3N in EtOAc) silica gel column chromatography (EtOAc) to afford the compound as a foamy pale-white solid (25 mg, 42% yield).

^{1}H NMR (400MHz, $CDCl_3$): δ 9.15 (ddd, *J* = 6.1, 1.7, 0.7 Hz, 1H), 8.09 (td, *J* = 7.8, 1.7 Hz, 1H), 7.66 (ddd, *J* = 7.5, 6.0, 1.3 Hz, 1H), 7.51 (d, *J* = 8.3 Hz, 1H), 7.44 – 7.32 (m, 4H), 7.31 – 7.27 (m, 1H), 3.53 (d, *J* = 4.8 Hz, 1H), 2.62 – 2.52 (m, 1H), 2.44 (d, *J* = 7.9 Hz, 1H), 2.21 (dd, *J* = 9.7, 4.9 Hz, 1H), 1.03 (s, 3H).

^{13}C NMR (126 MHz, $CDCl_3$): δ 168.2, 147.8, 143.0, 140.1, 128.9, 128.6, 126.9, 123.8, 120.7, 53.9, 46.7, 37.0, 19.7.

HRMS: (ESI, m/z): Calculated for $C_{16}H_{16}BCl_2NNa$ $[M+Na]^+$: 326.0646, found: 326.0645.

IR: 2926.08, 1734.59, 1620.37, 1487.25, 1086.78, 750.35 cm^{-1}.

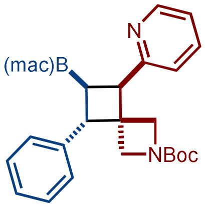

tert-butyl-6-(6b,9a-dimethyl-6b,9a-dihydroacenaphtho[1,2-d][1,3,2]dioxaborol-8-yl)-5-phenyl-7-(pyridin-2-yl)-2-azaspiro[3.3]heptane-2-carboxylate (32):

The title compound was prepared according to the general procedure **2** from styrenyl trifluoroborate salt **27** (84.3 mg, 0.280 mmol, 1.40 equiv) and tert-butyl 3-(pyridin-2-ylmethylene)azetidine-1-carboxylate **S43** (49.2 mg, 0.200 mmol, 1.00 equiv.) The crude product was purified with pre basified (2% Et_3N in EtOAc) silica gel column chromatography (EtOAc) to afford the compound as a foamy pale-yellow solid (93.0 mg, 0.17 mmol, 81% yield).

^{1}H NMR (400 MHz, $CDCl_3$) δ 7.83 (t, *J* = 7.6 Hz, 1H), 7.74 (dd, *J* = 15.0, 8.2 Hz, 2H), 7.57 (dd, *J* = 8.1, 6.8 Hz, 1H), 7.53 – 7.41 (m, 3H), 7.38 – 7.25 (m,2H,1H), 7.20 (t, *J* = 7.2 Hz, 1H), 7.14 (d, *J* = 5.7 Hz, 1H), 7.03 (t, *J* = 6.6 Hz, 3H), 3.75 – 3.64 (m, 3H), 3.57 – 3.47 (m, 2H), 3.37 (d, *J* = 8.5 Hz, 1H), 2.04 – 1.94 (m, 1H), 1.69 (s, 3H), 1.63 (s, 3H), 1.36 (s, 9H).

^{13}C NMR (126 MHz, $CDCl_3$): δ = 160.2, 156.6, 148.4, 148.0, 142.4, 142.0, 140.8, 134.8, 131.4, 128.5, 128.4, 128.2, 127.6, 126.0, 124.7, 124.3, 124.1, 123.7, 119.0, 118.9,

90.0, 89.4, 79.5, 48.5, 45.7, 44.5, 30.3, 28.3, 24.0, 22.7.
HRMS: ESI (m/z): Calculated for $C_{36}H_{38}O_4N_2B$ [M+H]+: 573.2919, found: 573.2926.
IR (neat): 2930.52, 1697.83, 1392.42, 1087.65, 745.21 cm^{-1}.

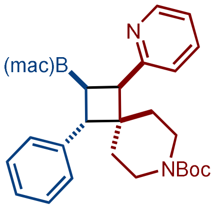

tert-butyl-2-(6b,9a-dimethyl-6b,9a-dihydroacenaphtho[1,2-d][1,3,2]dioxaborol-8-yl)-1-phenyl-3-(pyridin-2-yl)-7-azaspiro[3.5]nonane-7-carboxylate (33):
The title compound was prepared according to the general procedure **2** from styrenyl trifluoroborate salt **27** (84.3 mg, 0.28 mmol, 1.40 equiv) and tert-butyl 4-(pyridin-2-ylmethylene)piperidine-1-carboxylate **S44** (54.8 mg, 0.200 mmol, 1.00 equiv.) The crude product was purified with pre basified (2% Et_3N in EtOAc) silica gel column chromatography (EtOAc) to afford the compound as a foamy pale-yellow solid (62 mg, 0.11 mmol, 52% yield).
^{1}H NMR (500 MHz, $CDCl_3$): δ 7.77 (td, *J* = 7.7, 1.5 Hz, 1H), 7.73 (d, *J* = 8.1 Hz, 1H), 7.67 (d, *J* = 8.2 Hz, 1H), 7.56 (dd, *J* = 8.2, 6.8 Hz, 1H), 7.50 (d, *J* = 6.7 Hz, 1H), 7.38 (dd, *J* = 8.2, 6.8 Hz, 1H), 7.32 (d, *J* = 8.0 Hz, 1H), 7.28 (d, *J* = 7.0 Hz, 2H), 7.21 (dd, *J* = 8.0, 6.4 Hz, 2H), 7.19 – 7.13 (m, 1H), 7.09 – 6.99 (m, 3H), 3.40 (d, *J* = 8.5 Hz, 1H), 3.37 – 3.27 (m, 1H), 3.26 – 3.17 (m, 2H), 3.12 (ddd, *J* = 12.2, 7.6, 3.6 Hz, 1H), 2.92 – 2.83 (m, 1H), 2.05 (t, *J* = 8.6 Hz, 1H), 1.66 (s, 3H), 1.58 (s, 3H), 1.46 (t, *J* = 4.3 Hz, 1H), 1.37 (s, 9H), 1.34 – 1.24 (m, 2H), 1.21 – 1.09 (m, 1H).
^{13}C NMR (126 MHz, $CDCl_3$): δ = 161.8, 154.9, 148.6, 148.1, 142.5, 142.1, 140.5, 134.9, 131.4, 128.7 (2C overlap), 128.5, 127.9, 125.8, 124.9, 124.3, 124.0, 123.4, 119.0, 118.9, 89.9, 89.3, 79.4, 48.8, 47.8, 45.0, 35.0, 33.8, 28.5, 24.3, 22.6.
HRMS: ESI (m/z): Calculated for $C_{38}H_{42}O_4N_2B$ [M+H]+: 601.3232, found: 601.3249.
IR (neat): 2928.64, 1736.48, 1686.25, 1365.38, 1240.29, 753.59 cm^{-1}.

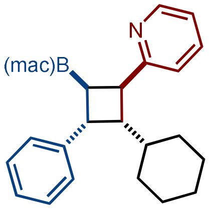

2-cyclohexyl-4-(6b,9a-dimethyl-6b,9a-dihydroacenaphtho[1,2-d] [1,3,2] dioxaborol-8-yl)-3-phenylcyclobutyl) pyridine: (34):
The title compound was prepared according to the general procedure **2** from styrenyl trifluoroborate salt **27** (84.3 mg, 0.280 mmol, 1.40 equiv) and (E)-2-(2-cyclohexylvinyl)pyridine **S46** (37.4 mg, 0.200 mmol, 1.00 equiv.) ^{1}H-NMR analysis of the crude reaction mixture shows 71% NMR yield of the cycloadduct, >20:1 dr. The crude product was purified with pre basified (2% Et_3N in EtOAc) silica gel column chromatography (EtOAc) to afford the compound as a foamy white solid (59 mg, 0.12 mmol, 58% yield).

^{1}H NMR (500 MHz, CDCl$_3$): δ 7.76 – 7.71 (m, 2H), 7.64 (td, *J* = 7.6, 1.6 Hz, 1H), 7.59 – 7.53 (m, 3H), 7.52 – 7.48 (m, 1H), 7.34 (dd, *J* = 8.1, 1.5 Hz, 2H), 7.29 (t, *J* = 7.6 Hz, 2H), 7.22 (d, *J* = 7.9 Hz, 1H), 7.20 – 7.16 (m, 1H), 7.00 (t, *J* = 4.8 Hz, 1H), 6.84 (ddd, *J* = 7.1, 5.7, 1.2 Hz, 1H), 3.67 (dd, *J* = 9.0, 7.1 Hz, 1H), 3.60 (dd, *J* = 9.0, 3.9 Hz, 1H), 2.19 – 2.10 (m, 1H), 1.87 (dd, *J* = 9.1, 3.8 Hz, 1H), 1.84 – 1.77 (m, 1H), 1.74 (s, 3H), 1.64 (s, 3H), 1.63 – 1.57 (m, 1H), 1.48 (dt, *J* = 13.0, 3.5 Hz, 1H), 1.38 – 1.32 (m, 1H), 1.30 – 1.20 (m, 3H), 1.10 (qt, *J* = 12.7, 3.5 Hz, 1H), 1.02 – 0.87 (m, 1H), 0.75 – 0.60 (m, 2H), 0.51 – 0.40 (m, 1H).
^{13}C NMR (126 MHz, CDCl$_3$): δ = 165.7, 149.1, 148.3, 145.6, 142.1, 140.8, 135.0, 131.6, 129.3, 128.6, 128.5, 127.7, 125.4, 124.3, 124.2, 122.63, 122.62, 119.06, 119.05, 90.0, 89.8, 52.4, 45.7, 40.8, 38.5, 30.5, 30.1, 26.5, 26.1, 25.6, 24.2, 22.7.
HRMS: ESI (m/z): Calculated for $C_{35}H_{37}O_2NB$ [M+H]+: 514.2912, found: 514.2920.
IR (neat): 2922.87, 1737.37, 1572.86, 1481.87, 1087.82, 781.91 cm^{-1}.

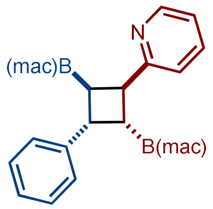

2-((2,4-bis(6b,9a-dimethyl-6b,9a-dihydroacenaphtho[1,2-d] [1,3,2] dioxaborol-8-yl)-3-phenylcyclobutyl) pyridine (35): The title compound was prepared according to the general procedure **2** from styrenyl trifluoroborate salt **27** (84.3 mg, 0.280 mmol, 1.40 equiv) and (E)-2-(2-(4,4,5,5-tetramethyl-1,3,2-dioxaborolan-2-yl)vinyl)pyridine **S7** (46.2 mg, 0.200 mmol, 1.00 equiv.) ^{1}H-NMR analysis of the crude reactiuon mixture shows formation of cycloadduct in 90% yield, 1.6:1 dr. After the mac-OH protection, the crude product was purified with pre basified (2% Et_3N in EtOAc) silica gel column chromatography (EtOAc) to afford the compound as a foamy white solid (59.0 mg, 0.09 mmol, 45% yield, major diastereomer).
^{1}H NMR (500 MHz, CDCl$_3$): δ 7.79 – 7.72 (m, 3H), 7.72 – 7.68 (m, 1H), 7.62 (td, *J* = 7.6, 1.5 Hz, 1H), 7.59 – 7.49 (m, 4H), 7.46 (dd, *J* = 8.1, 6.9 Hz, 1H), 7.42 (dd, *J* = 6.9, 2.5 Hz, 2H), 7.39 (d, *J* = 6.8 Hz, 1H), 7.19 – 7.14 (m, 1H), 7.13 – 7.07 (m, 2H), 7.03 – 6.96 (m, 3H), 6.96 – 6.92 (m, 1H), 6.86 (t, *J* = 6.7 Hz, 1H), 3.75 (dd, *J* = 8.4, 4.7 Hz, 1H), 3.65 (dd, *J* = 10.3, 7.3 Hz, 1H), 2.32 – 2.23 (m, 1H), 2.11 (ddd, *J* = 10.3, 4.7, 1.1 Hz, 1H), 1.67 (d, *J* = 10.7 Hz, 3H, 3H), 1.45 (s, 3H), 1.34 (s, 3H).
^{13}C NMR (126 MHz, CDCl$_3$): δ = 166.3, 149.0, 148.3, 146.3, 145.1, 145.0, 141.3, 140.8, 134.9, 134.8, 131.5, 131.4, 128.6, 128.54, 128.51 (2C, overlap), 127.6, 127.4, 125.3, 125.2, 125.1, 124.3, 124.1, 123.2, 122.8, 119.5, 119.4, 119.1, 119.0, 92.04, 91.95, 90.0, 89.4, 39.7, 39.0, 24.3, 22.8, 22.0, 21.7.
HRMS: ESI (m/z): Calculated for $C_{43}H_{38}O_4NB_2$ [M+H]+: 654.2981, found: 654.3001.
IR (neat): 2926.96, 1735.32, 1617.55, 1448.99, 1372.68, 780.19 cm^{-1}.

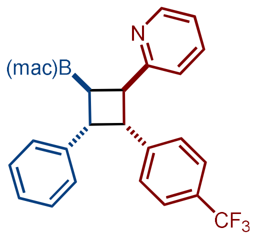

2-(2-(6b,9a-dimethyl-6b,9a-dihydroacenaphtho[1,2-d][1,3,2]dioxaborol-8-yl)-3-phenyl-4-(4-(trifluoromethyl)phenyl)cyclobutyl)pyridine (36):

The title compound was prepared according to the general procedure **2** from styrenyl trifluoroborate salt **27** (84.3 mg, 0.280 mmol, 1.40 equiv) and (E)-2-(4-(trifluoromethyl)styryl) pyridine **S41** (50 mg, 0.20 mmol, 1.0 equiv). ^{1}H-NMR analysis of the crude reaction mixture shows formation of cycloadduct in 91% NMR yield, 3.5:1 dr. The crude product was purified with pre-basified (2% Et_3N in EtOAc) silica gel column chromatography (EtOAc) to afford the compound as a foamy white solid (73 mg, 0.13 mmol, 63% yield).

^{1}H NMR (500 MHz, $CDCl_3$): δ 7.83 – 7.75 (m, 2H), 7.72 (td, *J* = 7.6, 1.6 Hz, 1H), 7.64 – 7.51 (m, 3H), 7.48 (d, *J* = 6.8 Hz, 1H), 7.32 (d, *J* = 8.1 Hz, 2H), 7.25 (s, 1H), 7.12 – 7.02 (m, 5H), 7.02 – 6.93 (m, 2H), 6.86 (d, *J* = 7.2 Hz, 2H), 4.03 (td, *J* = 9.9, 5.9 Hz, 2H), 3.81 (dd, *J* = 9.3, 5.8 Hz, 1H), 2.31 (dd, *J* = 9.0, 6.0 Hz, 1H), 1.75 (d, *J* = 5.7 Hz, 6H).

^{13}C NMR (126 MHz, $CDCl_3$): δ = 163.7, 148.7, 148.2, 145.4, 142.6, 142.4, 141.1, 135.0, 131.6, 128.7, 128.6, 128.4, 128.3, 128.1 (q, *J* = 31.5 Hz), 127.8, 125.4, 124.8 (q, *J* = 3.8 Hz), 124.5, 124.4 (q, *J* = 272.2 Hz), 124.3, 123.5, 122.7, 119.2, 119.1, 90.2, 90.0, 51.4, 45.8, 42.8, 24.1, 23.1.

^{19}F NMR (471 MHz, CD_3CN) δ – 62.34.

HRMS: ESI (m/z): Calculated for $C_{36}H_{30}O_2NBF_3$ [M+H]+: 576.2316, found: 576.2326.

IR: 2929.23, 1735.65, 16161.29, 1325.03, 1120.42, 782.51 cm^{-1}.

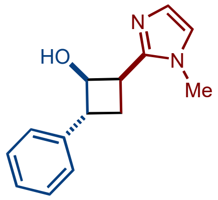

(1R,2R,4R)-2-(1-methyl-1H-imidazol-2-yl)-4-phenylcyclobutan-1-ol (37):

The title compound was prepared according to the general procedure 3 from styrenyl trifluoroborate salt **27** (60.2 mg, 0.200 mmol, 1.00 equiv) and 1-methyl-2-vinyl-1H-imidazole **S40** (43.3 mg, 0.400 mmol, 2.00 equiv.) using 395 nm LEDs in IPR for 24 h. After the cycloadditions reaction, all volatiles were removed in vacuo and taken for crude NMR analysis. After that the crude material was concentrated in a 25 mL round bottom flask. A stir bar was added, and 5 mL of anhyd. THF was added to it, followed by addition of 2 mL of 4M aqueous NaOH solution dropwise. To the stirring reaction mixture 2 mL of 30% aqueous H_2O_2 was added dropwise and stir the reaction mixture for 4h at room temperature. After that, the organic layer was separated and the aqueous layer was extracted with EtOAc (5 mL X 3 times). The combined organic layer

was dried with anhyd. MgSO4 and filtered and concentrated. The crude product was purified with pre basified (1% Et_3N in DCM) silica gel column chromatography (MeOH: Et_3N: DCM = 2: 1: 97) to afford the compound as a colorless oil (25 mg, 0.11 mmol, 55% yield). Use CAM as stain.

^{1}H NMR (500 MHz, $CDCl_3$): δ 7.25 – 7.17 (m, 4H), 7.13 (ddd, *J* = 8.7, 5.5, 2.3 Hz, 1H), 6.86 (d, *J* = 1.3 Hz, 1H), 6.65 (d, *J* = 1.3 Hz, 1H), 4.54 (s, 1H), 4.26 (q, *J* = 7.9 Hz, 1H), 3.72 (t, *J* = 8.6 Hz, 1H), 3.27 (s, 3H), 2.94 (td, *J* = 9.6, 7.8 Hz, 1H), 2.70 (dt, *J* = 11.0, 7.5 Hz, 1H), 2.46 – 2.37 (m, 1H).

^{13}C NMR (126 MHz, $CDCl_3$): δ = 149.8, 141.4, 128.6, 127.1, 127.0, 126.8, 120.9, 69.9, 58.3, 36.0, 32.7, 30.2.

HRMS: ESI (m/z): Calculated for $C_{14}H_{17}ON_2$ [M+H]+: 229.1335, found: 229.1334.

IR (neat): 2924.30, 1732.84, 1669.76, 1494.96, 699.39 cm^{-1}.

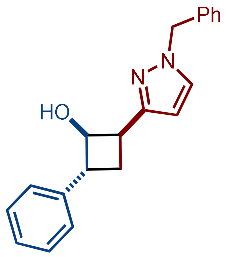

2-(1-benzyl-1H-pyrazol-3-yl)-4-phenylcyclobutan-1-ol (38):

The title compound was prepared according to the general procedure 3 from styrenyl trifluoroborate salt **27** (60.2 mg, 0.200 mmol, 1.00 equiv) and 1-benzyl-3-vinyl-1H-pyrazole **S45** (73.6 mg, 0.400 mmol, 2.00 equiv.) using 395 nm LEDs in IPR for 24 h. After that the volatiles were removed in vacuo and taken for crude NMR analysis. After that the crude material was concentrated in a 25 mL round bottom flask. A stir bar was added, and 5 mL of anhyd. THF was added to it, followed by addition of 2 mL of 4M aqueous NaOH solution dropwise. To the stirring reaction mixture 2 mL of 30% aqueous H_2O_2 was added dropwise and stir the reaction mixture for 4h at room temperature. After that, the organic layer was separated and the aqueous layer was extracted with EtOAc (5 mL X 3 times). The combined organic layer was dried with anhyd. $MgSO_4$ and filtered and concentrated. The crude product was purified with pre-basified (2% Et_3N in EtOAc) silica gel column chromatography (EtOAc) to afford the compound as a white solid (15 mg, 0.050 mmol, 25% yield).

^{1}H NMR (400 MHz, $CDCl_3$): δ 7.35 – 7.31 (m, 2H), 7.31 – 7.28 (m, 1H), 7.28 – 7.25 (m, 3H), 7.22 (s, 3H), 7.17 (ddd, *J* = 9.3, 5.8, 2.0 Hz, 3H), 6.15 (d, *J* = 2.3 Hz, 1H), 5.28 (s, 2H), 4.56 (d, *J* = 10.6 Hz, 1H), 4.43 – 4.32 (m, 1H), 3.77 – 3.66 (m, 1H), 2.19 (td, *J* = 10.6, 2.1 Hz, 1H), 2.07 (td, *J* = 10.8, 8.7 Hz, 1H).

^{13}C NMR (126 MHz, $CDCl_3$): δ 153.6, 143.4, 136.7, 130.4, 129.0, 128.4, 128.2, 127.8, 126.7, 126.3, 106.0, 74.6, 56.1, 50.7, 37.4, 26.5.

HRMS: ESI (m/z): Calculated for $C_{20}H_{21}ON_2$ [M+H]+: 305.1648, found: 305.1649.

IR: 2928.59, 1734.37, 1496.91, 1454.76, 1123.40, 699.08 cm^{-1}.

5.4 Characterization of Pyridine-BF_2 Cycloadduct:

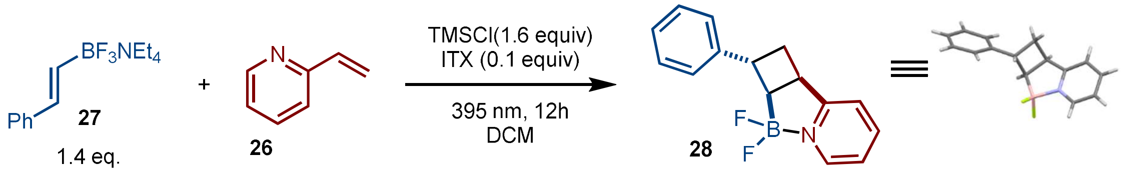

Following general procedure **2** - In a 2-dram vial with a stir bar inside was charged with BF_3 salt **27** (84.3 mg, 0.28 mmol, 1.40 equiv), ITX (5.1 mg, 0.020 mmol, 0.10 equiv) and degassed and backfilled with N_2 (X 3). Then 2 mL of anhyd. DCM was added under N_2 atmosphere followed by the addition of TMSCl (40 µL, 0.32 mmol, 1.6 equiv) dropwise at room temperature and stirred it for 10 mins at rt. Then with a syringe, 2-vinylpyridine **26** (21 µL, 0.20 mmol, 1.0 equiv) was slowly added to the reaction vial under N_2 atmosphere, and the septum was quickly replaced with plastic cap and sealed with Teflon tape. The reaction was allowed to be irradiated under 395 nm for 12h. After the time period, all the volatiles were removed and the crude mixture was purified by FCC (MeOH: DCM = 1:20) to obtain the product **28** as pale yellow solid. (31 mg, 0.12 mmol, 60% yield.)

^{1}H NMR (400 MHz, $CDCl_3$): δ 8.58 (d, J = 5.6 Hz, 1H), 8.12 (t, J = 7.7 Hz, 1H), 7.61 (d, J = 7.5 Hz, 2H), 7.36 – 7.30 (m, 4H), 7.23 – 7.16 (m, 1H), 3.86 (d, J = 9.4 Hz, 1H), 3.48 (q, J = 8.2 Hz, 1H), 2.90 (q, J = 10.1 Hz, 1H), 2.43 – 2.23 (m, 2H).

^{13}C NMR (126 MHz, $CDCl_3$): δ = 165.9, 147.0, 142.7, 140.8, 128.4, 126.6, 125.7, 124.0, 123.8, 38.7 (d, J = 3.8 Hz), 36.58 (t, J = 5.0 Hz), 35.16.

HRMS (EI, m/z): Calculated for $C_{15}H_{14}BF_2N$ $[M]^+$: 257.1185, Observed: 257.1178.

IR (neat): 2932, 1620, 1485, 1198, 1090, 1045, 751 cm^{-1}.

5.5 Large Scale [2+2] Cycloadditions Reaction and Product Diversifications:

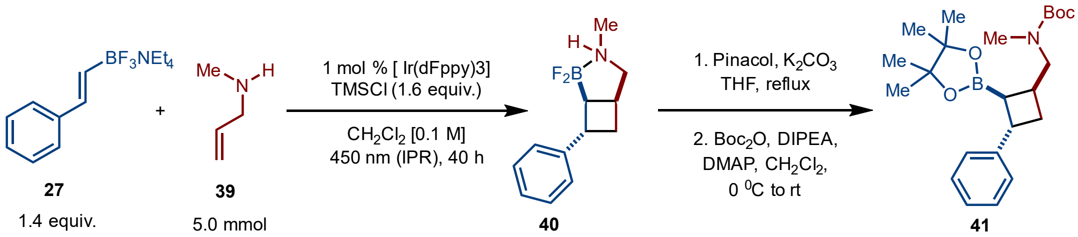

5.5a Procedure for Large Scale Reaction:

In an 150 mL glass reaction vial with a stir bar inside was charged with BF_3 salt **27** (2.11 g, 7.00 mmol, 1.40 equiv.), $Ir(dFppy)_3$ (38.1 mg, 0.0500 mmol, 0.0100 equiv) and degassed and backfilled with N_2 (X 3). Then 50 mL of CH_2Cl_2 was added under N_2 atmosphere followed by the addition of TMSCl (0.99 ml, 8.0 mmol, 1.6 equiv) dropwise at rt and stirred it for 30 mins at rt . Then with a syringe, methyl (prop-2-en-1-yl) amine **39** (0.50 mL, 5.0 mmol, 1.0 equiv) was slowly added to the reaction vial under N_2 atmosphere, and the septum was properly sealed with electric tape. The reaction was

allowed to be irradiated under 450 nm for 40h. After that 1 mL from reaction mixture was taken out by a syringe, and ^{1}H-NMR analysis was done after removal of all the volatiles. Crude NMR analysis shows >95% NMR yield, >20:1 dr. The crude reaction mixture was transferred to a 100 mL round bottom flask washing with CH_2Cl_2 several times. After that all the volatiles were removed in vacuo. With a stir bar inside, pinacol (0.89 g, 7.5 mmol, 1.5 equiv) and anhyd. K_2CO_3 (1.04g, 7.50 mmol, 1.50 equiv) were added, and a refluxing condenser was placed properly with the round bottom flask. 50 mL of anhyd. THF was added to the reaction mixture and the reaction was heated to reflux overnight (14 h). After that, the crude reaction mixture was passed through a celite pad to remove the solid. The filtrate was concentrated and in vacuo.

To the reaction mixture was added Boc_2O (3.28 g, 15.0 mmol, 3.00 equiv.) and DMAP (0.18 g, 1.5 mmol, 0.30 equiv.) and the reaction was capped with a septum. Then under N_2 atmosphere 50 mL anhyd. CH_2Cl_2 was added to the mixture through a syringe. Then at 0 ^{0}C (ice bath) slowly added DIPEA (4.3 ml, 25 mmol, 5.0 equiv.) with a syringe. After half an hour the ice bath was removed and the reaction was allowed to stir at rt for overnight.

After the reaction time period the reaction was quenched with slow addition of sat. aq. $NaHCO_3$ solution (25 mL) at 0 ^{0}C (ice bath). The organic layer was separated, and the aqueous layer was extracted 3 times with ethyl acetate (30 mL X 3). The combined organic layer was washed with brine and dried over MgSO4 followed by concentrated and purified with FCC (15% EtOAc in hexanes). The product **41** was isolated as a white solid (1.45 g, 3.62 mmol, 73% yield).

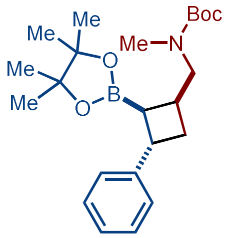

tert-butyl methyl(((1S,2R,3S)-3-phenyl-2-(4,4,5,5-tetramethyl-1,3,2-dioxaborolan-2-yl)cyclobutyl)methyl)carbamate (41):
^{1}H NMR (500 MHz, $CDCl_3$, *mixture of rotamers*): δ 7.29 (t, *J* = 7.6 Hz, 2H), 7.25 – 7.21 (m, 2H), 7.16 (t, *J* = 7.3 Hz, 1H), 4.10 – 3.84 (m, 1H), 3.83 – 3.55 (m, 1H), 3.13 (dd, *J* = 13.8, 4.4 Hz, 1H), 2.83 (s, 3H), 2.70 (tq, *J* = 11.0, 5.6 Hz, 1H), 2.28 (dd, *J* = 8.6, 6.0 Hz, 2H), 2.15 (t, *J* = 9.5 Hz, 1H), 1.48 (s, 9H), 1.27 (d, *J* = 3.5 Hz, 12H).
^{13}C NMR (126 MHz, $CDCl_3$, *mixture of rotamers*): δ = 156.3, 146.6, 128.3, 126.4, 125.7, 83.5, 79.4, 51.5, 38.1, 34.0, 31.7, 28.7, 28.0, 25.2, 25.0.
IR (neat): 2931, 1692, 1378, 1139, 854 cm^{-1}.
HRMS (ESI, m/z): Calculated for $C_{23}H_{36}O_4NBNa$ $[M+Na]^+$: 424.2630, observed: 424.2636.

5.5b Oxidation:

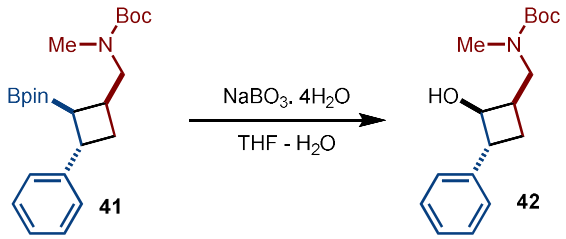

tert-butyl (((1R,2R,3R)-2-hydroxy-3-phenylcyclobutyl)methyl)(methyl)carbamate (42): The title compound was prepared according to literature procedure.[30] The starting cyclobutylboronate **41** (80.2 mg, 0.200 mmol) was dissolved in 4 mL (THF/H_2O 1:1) and was cooled to 0 ˚C using an ice/water bath. Solid sodium perborate (62 mg, 0.40 mmol, 2.0 equiv.) was added directly. Reaction was allowed to warm to room temperature and stir vigorously for 12 hours. Reaction was quenched with 2 mL of water. Aqueous layer was extracted three times with ethyl acetate. The collected organic layers were washed with brine, dried with $MgSO_4$, filtered, and concentrated under vacuum. Crude mixture was purified using silica gel column chromatography (20% EtOAc in hexanes) to afford the product as a colorless liquid (57 mg, 0.2 mmol, 99% yield).

^{1}H NMR (500 MHz, $CDCl_3$, *mixture of rotamers*): δ 7.29 – 7.23 (m, 4H), 7.21 – 7.15 (m, 1H), 4.92 (s, 1H), 4.08 (d, *J* = 12.2 Hz, 2H), 3.46 (q, *J* = 9.1 Hz, 1H), 3.07 – 3.00 (m, 1H), 2.90 (s, 3H), 2.76 (m, 1H), 1.81 (m 2H), 1.48 (s, 9H).

^{13}C NMR (126 MHz, $CDCl_3$, *mixture of rotamers*): δ 157.46, 143.04, 128.31, 126.53, 126.15, 80.67, 73.39, 49.61, 48.78, 37.55, 34.76, 28.45, 22.78.

IR (neat): 3386 (br), 2973, 2929, 1662, 1152, 698 cm^{-1}.

HRMS: (ESI, m/z): Calculated for $C_{17}H_{25}O_3NNa$ $[M+Na]^+$: 314.1727, observed: 314.1731.

5.5c Metal-free Cross Coupling:

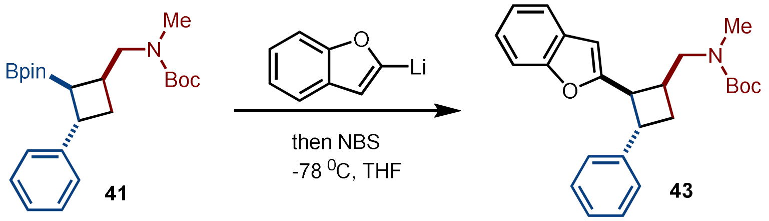

tert-butyl (((1S,2R,3S)-2-(benzofuran-2-yl)-3-phenylcyclobutyl)methyl)(methyl)carbamate (43): The title compound was prepared according to literature procedure.[31] A reaction tube equipped with a stir bar was flame-dried under vacuum. Once cooled, then the flask was backfilled with N_2. The tube was evacuated and backfilled with N_2 (X 3 times). Under N_2 atmosphere was charged with benzofuran (17.0 μL, 0.150 mmol, 1.50 equiv.) and THF (0.5 mL) sequentially via syringe. The mixture was cooled to -78 ^{o}C in a dry ice/acetone bath, and then n-butyllithium (61.5 uL, 2.44 M in hexanes, 0.150 mmol, 1.50 equiv) was

added dropwise via syringe. The mixture was warmed to room temperature and stirred for 1 h, before being cooled to -78 °C. A solution of cyclobutylborane **41** (41 mg, 0.10 mmol, 1.0 equiv.) in THF (0.5 mL) was then added dropwise via syringe to the solution of lithiated benzofuran at -78 °C, and stirred for 1 h at same temperature. The reaction mixture was then added dropwise via syringe to a solution of NBS (36 mg, 0.20 mmol, 2.0 equiv.) in THF (0.5 mL) at -78 °C, and stirred for 1 h at same temperature. After 1 h, the reaction was quenched with 20% aq. $Na_2S_2O_3$ (3 mL) and warmed to room temperature. The layers were separated, and the aqueous layer was extracted with ethyl acetate (3 X 3 mL), the combined organic layers were dried over $MgSO_4$, filtered, and concentrated in vacuo. The crude material was purified by FCC (EtOAc:hexanes = 1:9) to afford the product as a colorless oil (22 mg, 56 µmol, 56% yield).

^{1}H NMR (500 MHz, CDCl$_3$, *mixture of rotamers*): δ 7.73 – 7.35 (m, 2H), 7.35 – 7.14 (m, 7H), 6.56 (s, 1H), 4.36 – 3.76 (m, 3H), 2.92 (td, *J* = 17.3, 7.5 Hz, 2H), 2.84 – 2.70 (m, 3H), 2.39 – 2.25 (m, 2H), 1.61 – 1.24 (m, 9H).

^{13}C NMR (126 MHz, CDCl$_3$, *mixture of rotamers*): δ = 157.83, 156.28, 155.94, 154.92, 143.57, 129.51, 128.45, 126.33, 123.60, 122.62, 120.51, 110.95, 103.63, 76.78, 49.38, 42.05, 40.99, 34.17, 33.93, 28.84, 28.44.

IR (neat): 2931, 1688, 1453, 1163, 750 cm^{-1}.

HRMS (ESI, m/z): Calculated for $C_{25}H_{29}O_3NNa$ [M+Na]$^+$: 414.2040, observed: 414.2043.

5.5d Bromination:

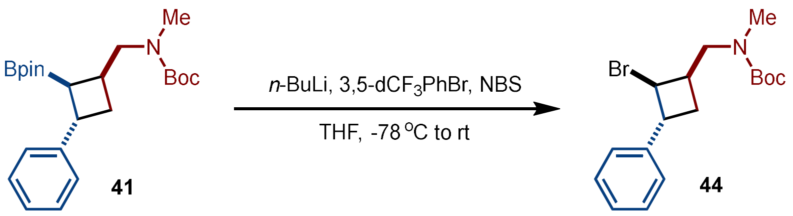

tert-butyl (((1R,2R,3R)-2-bromo-3-phenylcyclobutyl)methyl)(methyl)carbamate (44): Following the literature procedure.[32] A reaction tube was flamed-dried under vacuum. Once cool, the tube was refilled with N_2 and evacuated and backfilled with N_2 three times. Once under N_2, the tube was sealed with a septum. The septum was briefly removed and charged with the 1-bromo-3,5-bis(trifluoromethyl)benzene (58.6 mg, 0.200 mmol, 2.00 equiv) and dissolved in anhyd. THF (0.6 mL). The flask was cooled to –78 °C (dry ice/acetone) followed by dropwise addition of nBuLi (2.40 M in hexanes, 84.0 µL, 0.200 mmol, 2.00 equiv) and allowed to react for 1 h. A separate flame-dried 2 mL dram vial was charged with boronic ester **41** (40.1 mg, 0.100 mmol, 1.00 equiv.) and dissolved in anhyd. THF (0.6 mL). This solution was added dropwise to the former ArLi mixture and allowed to stir for 30 min at –78 °C. The reaction was warmed to ambient temperature followed by stirring for 30 min. A second flame-dried, dram vial was charged with NBS (35.6 mg, 0.200 mmol, 2.00 equiv) and THF (0.6 mL) that was subsequently added dropwise to the reaction mixture. After stirring for 1 h, the reaction was quenched with $Na_2S_2O_3$ (20 wt% in H_2O, 5 mL) slowly –78 °C and allowed to warm to rt. The mixture was extracted with EtOAc (15 mL X 3), dried over Na_2SO_4, and concentrated. The product was purified by flash column chromatography

(5% acetone in hexanes). Colorless oil (22 mg, 62 µmol, 62% yield).
^{1}H NMR (500 MHz, CDCl$_3$, *mixture of rotamers*): δ 7.38 (t, *J* = 7.6 Hz, 2H), 7.32 – 7.21 (m, 3H), 4.84 – 4.57 (m, 1H), 3.84 (s, 1H), 3.68 – 3.49 (m, 1H), 3.33 (dd, *J* = 14.3, 6.6 Hz, 1H), 3.06 (s, 1H), 2.93 – 2.87 (m, 3H), 2.61 (t, *J* = 11.4 Hz, 1H), 2.20 (dt, *J* = 11.8, 8.5 Hz, 1H), 1.50 – 1.46 (m, 9H).
^{13}C NMR (126 MHz, CDCl$_3$, ***mixture of rotamers***): δ = 156.4, 140.7, 128.4, 127.1, 126.4, 80.0, 51.5, 50.6, 45.0, 43.6, 35.0, 28.6, 27.8.
IR (neat): 2974, 1690, 1365, 1143, 698 cm^{-1}.
HRMS: (APCI, m/z): Calculated for $C_{17}H_{24}O_2NBrNa$ [M+Na]$^+$: 376.0883, observed: 376.0884.

5.5e Homologation:

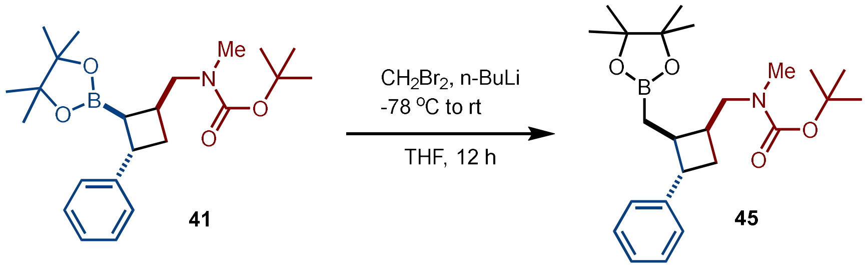

tert-butyl methyl(((1S,2R,3S)-3-phenyl-2-((4,4,5,5-tetramethyl-1,3,2-dioxaborolan-2-yl)methyl)cyclobutyl)methyl)carbamate (45): The title compound was prepared according to literature procedure.[33] A 2 mL dram vial equipped with a stir bar was flamed-dried under vacuum. Once cool, the tube was refilled with N_2 and evacuated and backfilled with N_2 three times. Once under N_2, the tube was sealed with a septum. The septum was briefly removed and was charged with cyclobutyl boronate **41** (40.1 mg, 0.100 mmol, 1.00 equiv.) and evacuated and backfilled with N_2 X 3. THF (1.00 mL) and dibromomethane (17.5 µL, 0.250 mmol, 2.50 equiv.) were added sequentially via syringe and the mixture was cooled to -78 °C in a dry ice/acetone bath. *n*-BuLi (90.0 µL, 2.44 M in hexanes, 0.220 mmol, 2.20 equiv.) was added dropwise via syringe over 10 minutes. The reaction was stirred at -78 °C for 5 minutes and then warmed to room temperature and stirred for 18 h. The reaction was quenched with H_2O (3 mL), the layers were separated, and the aqueous layer was extracted with ethyl acetate (3 X 3 mL). The combined organic layers were dried over $MgSO_4$, gravity filtered, and concentrated in vacuo. Purification via column chromatography (15% EtOAc in hexanes). Colorless oil (31 mg, 75 µmol, 75% yield).
^{1}H NMR (500 MHz, CDCl$_3$, , *mixture of rotamers*): δ 7.26 (p, *J* = 3.0 Hz, 4H), 7.20 – 7.13 (m, 1H), 4.02 – 3.78 (m, 1H), 3.14 (dd, *J* = 13.9, 5.0 Hz, 2H), 2.83 (s, 3H), 2.71 (p, *J* = 8.5 Hz, 1H), 2.59 (dddd, *J* = 13.6, 10.8, 8.2, 4.2 Hz, 1H), 2.16 – 1.98 (m, 2H), 1.48 (s, 9H), 1.11 (d, *J* = 15.9 Hz, 12H), 0.97 (dq, *J* = 13.5, 7.7 Hz, 2H).
^{13}C NMR (126 MHz, CDCl$_3$, *mixture of rotamers*): δ = 156.1, 144.4, 128.2, 127.0, 126.0, 83.0, 79.3, 48.6, 46.8, 40.8, 38.0, 33.7, 32.5, 29.5, 28.5, 24.7.
IR (neat): 2975, 2929, 1691, 1364, 1142, 697 cm^{-1}.
HRMS (ESI, m/z): Calculated for $C_{24}H_{38}O_4NBNa$ [M+Na]$^+$: 438.2786, observed: 438.2795.

5.5f Cu-catalyzed Alkenylation:

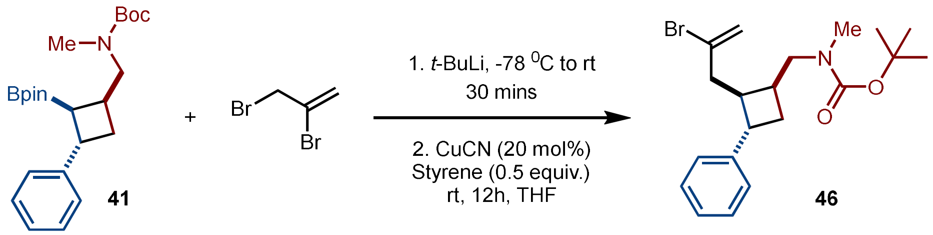

tert-butyl (((1S,2R,3S)-2-(2-bromoallyl)-3-phenylcyclobutyl)methyl)(methyl)carbamate (46): To an oven-dried reaction tube equipped with a magnetic stir bar was added alkyl boronic ester **41** (80.2 mg, 0.200 mmol, 1.00 equiv.). The tube was capped with a rubber septum and backfilled and refilled with N_2 (X 3). Under N_2 atmosphere anhydrous THF (0.5 mL) was added to the tube with a syringe. The reaction mixture was then cooled to -78 °C and *tert*-butyl lithium (0.15 mL, 1.3 M in pentane, 1.0 equiv.) was added dropwise by a syringe. The reaction mixture was then allowed to warm to room temperature and stirred for 30 minutes. The reaction vial was transferred into the glovebox, styrene (10.4 mg, 0.100 mmol, 0.500 equiv.), copper cyanide (3.6 mg, 0.040 mmol, 20 mol %) and electrophile 2,3-dibromo prop-1-ene (48.0 mg, 0.240 mmol, 1.20 equiv.) were added. The vial was sealed with a plastic cap and removed from the glovebox. The vial was stirred for 12 hours at 25 °C. The reaction mixture was diluted with diethyl ether and was passed through a silica gel plug with diethyl ether as eluent. The solvent was removed under reduced pressure. The crude product was purified by silica gel column chromatography (15% EtOAc in hexanes) to furnish the desired product. Colorless oil (52 mg, 0.13 mmol, 66% yield).

^{1}H NMR (500 MHz, $CDCl_3$, *mixture of rotamers*): δ 7.30 (d, J = 7.6 Hz, 2H), 7.26 – 7.23 (m, 2H), 7.23 – 7.16 (m, 1H), 5.53 (s, 1H), 5.37 (d, J = 7.6 Hz, 1H), 3.94 – 3.63 (m, 1H), 3.39 – 3.26 (m, 2H), 2.98 – 2.76 (m, 4H), 2.76 – 2.55 (m, 3H), 2.13 (h, J = 13.2 Hz, 2H), 1.48 (s, 9H).

^{13}C NMR (126 MHz, $CDCl_3$, ***mixture of rotamers***): δ 156.4, 144.1, 132.9, 128.5, 126.9, 126.5, 117.0, 79.6, 48.3, 43.6, 42.4, 41.8, 33.9, 31.6, 29.8, 28.6.

IR (neat): 2973, 2929, 1691, 1392, 1152, 698 cm^{-1}.

HRMS (ESI, m/z): Calculated for $C_{20}H_{28}O_2NBrNa$ [M+Na]$^+$: 416.1196, observed: 416.1199.

5.5g Cu-catalyzed Amination:

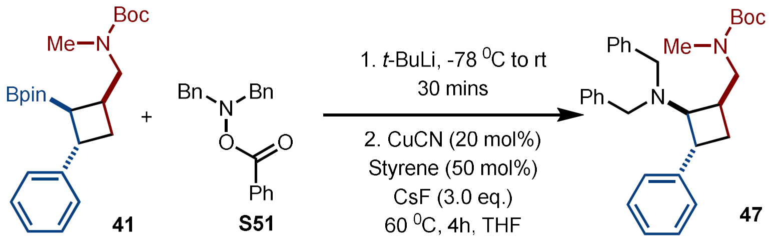

tert-butyl (((1R,2R,3R)-2-(dibenzylamino)-3-phenylcyclobutyl)methyl)(methyl)carbamate (47): The title compound was

prepared according to literature procedure.[34] In an oven-dried 2-dram vial equipped with a magnetic stir bar was added alkyl boronic ester **41** (40.1 mg, 0.100 mmol, 1.00 equiv). The vial was capped with a rubber septum and coated with Teflon. After evacuating and backfilled with N_2 (X 3), anhydrous THF (0.3 mL) was added to the reaction vial under N_2 atmosphere. The vial was cooled to -78 °C and *tert*-butyl lithium (71.4 μL, 1.40 M in pentane, 1.00 equiv) was added dropwise by a syringe. The vial was then allowed to warm to room temperature and further stirred for 30 minutes. The reaction vial was then transferred into the glove box, styrene (5.20 mg, 50.0 μmol, 0.500 equiv), copper cyanide (1.79 mg, 0.0200 mmol, 0.200 equiv), cesium fluoride (45.6 mg, 0.300 mmol, 3.00 equiv.) and aminating reagent **S51** (38.1 mg, 0.120 mmol, 1.20 equiv) were added. The vial was sealed with plastic cap and removed from the glove box. The vial was heated to 80 °C (oil bath) and stirred for 4 hours. The reaction mixture was subsequently diluted with diethyl ether and was passed through a silica gel plug using diethyl ether as eluent. The solvent was removed under reduced pressure. The crude product was purified by column chromatography. (15% EtOAc in hexanes). The product was isolated as colorless oil (29.0 mg, 62.0 μmol, 62% yield).
^{1}H NMR (500 MHz, $CDCl_3$, *mixture of rotamers*): δ 7.36 – 7.14 (m, 14H), 7.08 (d, *J* = 7.4 Hz, 1H), 4.44 – 3.99 (m, 1H), 3.83 – 3.32 (m, 6H), 3.28 – 2.94 (m, 1H), 2.90 – 2.66 (m, 3H), 2.58 (d, *J* = 10.4 Hz, 1H), 2.17 – 1.98 (m, 1H), 1.66 (td, *J* = 10.6, 7.9 Hz, 1H), 1.48 (s, 9H).
^{13}C NMR (126 MHz, $CDCl_3$, *mixture of rotamers*) δ 156.2, 143.5, 139.0, 128.9, 128.3, 128.1, 127.3, 126.8, 126.4, 79.4, 65.3, 55.5, 48.0, 44.3, 34.3, 34.2, 28.6, 27.8.
IR (neat): 2973, 2929, 1688, 1154, 749, 697 cm^{-1}.
HRMS (ESI, m/z): Calculated for $C_{31}H_{39}O_2N_2$ [M+H]$^+$: 471.3006, observed: 471.3008.

5.5h Cu-catalyzed Acylation:

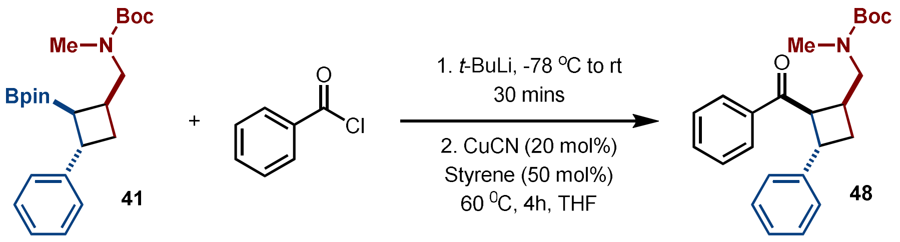

tert-butyl (((1S,2R,3S)-2-benzoyl-3-phenylcyclobutyl)methyl)(methyl)carbamate (48): The title compound was prepared according to literature procedure.[35] In an oven-dried 2-dram vial equipped with a magnetic stir bar was added alkyl boronic ester **41** (40.1 mg, 0.100 mmol, 1.00 equiv). The vial was capped with a rubber septum and coated with teflon. After evacuating and backfilled with N_2 (X 3), anhyd. THF (0.3 mL) was added to the reaction vial under N_2 atmosphere. The vial was cooled to -78 °C and *tert*-butyl lithium (71.4 μL, 1.40 M in pentane, 1.00 equiv) was added dropwise by a syringe. The vial was then allowed to warm to room temperature and further stirred for 30 minutes. The reaction vial was then transferred into the glove box, styrene (5.20 mg, 50.0 μmol, 0.500 equiv), copper cyanide (1.79 mg, 0.0200 mmol, 0.200 equiv)

and benzoyl chloride (16.9 mg, 0.120 mmol, 1.20 equiv) were added. The vial was sealed with plastic cap and removed from the glove box. The vial was heated to 60 °C (oil bath) and stirred for 4 h. The reaction mixture was subsequently diluted with diethyl ether and was passed through a silica gel plug using diethyl ether as eluent. The solvent was removed under reduced pressure. The crude product was purified by column chromatography. (15% EtOAC in hexanes). The product was isolated as colorless oil (32.0 mg, 84.0 µmol, 84% yield).

^{1}H NMR (500 MHz, CDCl$_3$, *mixture of rotamers*): δ 7.93 (d, *J* = 7.6 Hz, 2H), 7.55 (t, *J* = 7.5 Hz, 1H), 7.45 (t, *J* = 7.6 Hz, 2H), 7.31 – 7.23 (m, 4H), 7.18 (t, *J* = 7.2 Hz, 1H), 4.42 – 4.34 (m, 1H), 4.18 – 4.08 (m, 1H), 3.57 (dd, *J* = 14.0, 10.6 Hz, 1H), 3.41 – 3.10 (m, 1H), 2.98 (d, *J* = 13.4 Hz, 1H), 2.75 (m, 3H), 2.40 (td, *J* = 10.8, 8.4 Hz, 1H), 2.29 – 2.09 (m, 1H), 1.69 – 1.31 (m, 9H).

^{13}C NMR (126 MHz, CDCl$_3$, *mixture of rotamers*): δ = 198.4, 155.8, 143.8, 136.5, 133.4, 128.8, 128.4, 127.9, 126.6, 126.3, 79.7, 51.5, 49.0, 36.8, 35.7, 34.9, 28.4, 27.8.

IR (neat): 2974, 2931, 1675, 1365, 1162, 1001, 697cm^{-1}.

HRMS: (ESI, m/z): Calculated for $C_{24}H_{29}O_3NNa$ [M+Na]$^+$: 402.2040, observed: 402.2045.

5.5i General Procedure for Negishi Cross Coupling:

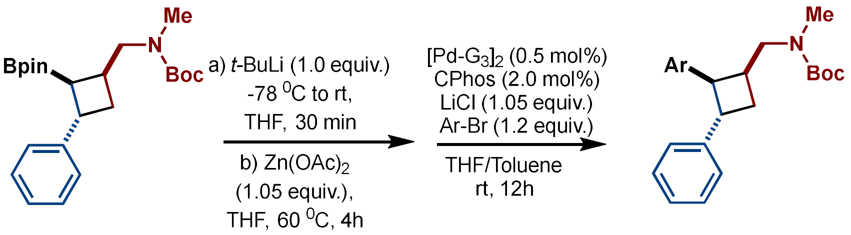

Following the literature procedure.[36] To an oven-dried reaction tube with a stir bar inside, was added alkylboronic pinacol ester **41** (80.2 mg, 0.200 mmol, 1.00 equiv.). The tube was capped with a rubber septum and backfilled and refilled with N_2 (X 3). Under N_2 atmosphere anhyd. THF (1.0 mL) was added to the tube with a syringe. The vial cooled to - 78 °C. Tert-butyl lithium (0.140 mL, 1.43 M, 1.00 equiv.) was added dropwise by a syringe and the reaction was allowed to warm to room temperature and further sitr for 30 minutes. The reaction vial was transferred to the glovebox and added zinc acetate (38.5 mg, 0.210 mmol, 1.05 equiv.) and was allowed to stir at 60 °C for 4 hours. Upon completion, [Pd-G$_3$]$_2$ (0.74 mg, 0.010 mmol, 0.0050 equiv.) and CPhos (0.04 mmol, 0.02 equiv.) was added as a THF solution (premixed for 10 min. in 0.2 mL THF), lithium chloride (8.90 mg, 0.210 mmol, 1.05 equiv.), Aryl halide (0.240 mmol, 1.20 equiv.) and toluene (1.0 mL). The vial was capped and sealed with Teflon tape, brought out of glovebox, and was allowed to stir at room temperature for 12 hours. Upon completion, the reaction mixture was filtered through a silica gel plug, rinsed with diethyl ether, and concentrated under reduced pressure. The resulting reaction mixture was purified by silica gel column chromatography to furnish the desired product.

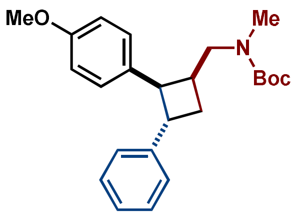

tert-butyl (((1S,2R,3S)-2-(4-methoxyphenyl)-3-phenylcyclobutyl)methyl)(methyl)carbamate (49): The title compound was prepared according to the general procedure for Negishi cross coupling using 1-bromo-4-methoxybenzene (45 mg, 0.24 mmol, 1.2 equiv.). The crude reaction mixture was purified by silica gel column chromatography to furnish the desired product (10% acetone in hexanes). Colorless oil (63 mg, 0.17 mmol, 83% yield).

^{1}H NMR (500 MHz, $CDCl_3$, *mixture of rotamers*): δ 7.31 – 7.23 (m, 4H), 7.21 – 7.13 (m, 3H), 6.87 (d, *J* = 7.5 Hz, 2H), 4.23 – 3.95 (m, 1H), 3.92 – 3.65 (m, 4H), 2.96 – 2.59 (m, 5H), 2.29 (dq, *J* = 17.4, 7.3 Hz, 1H), 2.17 (td, *J* = 10.5, 8.2 Hz, 1H), 1.48 (d, *J* = 17.3 Hz, 9H), 1.28 (d, *J* = 3.5 Hz, 1H).

^{13}C NMR (126 MHz, $CDCl_3$, *mixture of rotamers*): δ = 158.2, 156.1, 144.5, 131.9, 128.5, 126.6, 126.2, 120.4, 113.9, 79.4, 55.3, 49.6, 47.2, 41.0, 34.3, 29.0, 28.6, 24.7.

IR (neat): 2972, 2932, 1689, 1512, 1142, 698 cm^{-1}.

HRMS (ESI, m/z): Calculated for $C_{24}H_{31}O_3NNa$ $[M+Na]^+$: 404.2196, observed: 404.2197.

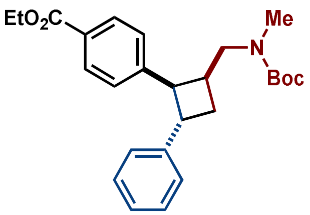

ethyl 4-((1R,2S,4S)-2-(((tert-butoxycarbonyl)(methyl)amino)methyl)-4-phenylcyclobutyl)benzoate (50): The title compound was prepared according to the general procedure for Negishi cross coupling using ethyl 4-bromobenzoate (55 mg, 0.24 mmol, 1.2 equiv.). The crude reaction mixture was purified by silica gel column chromatography to furnish the desired product (10% acetone in hexanes). Colorless oil (59 mg, 0.14 mmol, 70% yield).

^{1}H NMR (500 MHz, $CDCl_3$, *mixture of rotamers*): δ 8.05 (d, *J* = 7.7 Hz, 2H), 7.44 – 7.20 (m, 7H), 4.43 (q, *J* = 7.1 Hz, 2H), 4.33 – 4.10 (m, 1H), 4.01 (d, *J* = 9.3 Hz, 1H), 3.84 – 3.48 (m, 1H), 3.00 (d, *J* = 10.8 Hz, 1H), 2.84 (s, 1H), 2.79 (d, *J* = 10.9 Hz, 3H), 2.37 (d, *J* = 10.0 Hz, 1H), 2.28 (td, *J* = 10.5, 8.0 Hz, 1H), 1.61 – 1.48 (m, 9H), 1.44 (t, *J* = 7.1 Hz, 3H).

^{13}C NMR (126 MHz, $CDCl_3$, *mixture of rotamers*): δ = 166.5, 155.9, 145.2, 144.1, 129.7, 128.5, 127.6, 127.4, 126.5, 126.3, 79.4, 60.9, 49.3, 47.7, 40.7, 34.6, 34.3, 29.0, 28.5, 14.4.

IR (neat): 2976, 2933, 1688, 1273, 1166, 698 cm^{-1}.

HRMS (ESI, m/z): Calculated for $C_{26}H_{33}O_4NNa$ [M+Na] $^+$: 446.2302, observed: 446.2304.

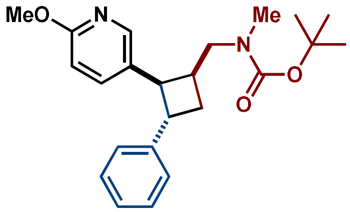

tert-butyl (((1S,2R,3S)-2-(6-methoxypyridin-3-yl)-3-phenylcyclobutyl)methyl)(methyl)carbamate (51): The title compound was prepared according to the general procedure for Negishi cross coupling using 5-bromo-2-methoxypyridine (51 mg, 0.24 mmol, 1.2 equiv.). The crude reaction mixture was purified by silica gel column chromatography to furnish the desired product (10% acetone in hexanes). Colorless oil (51 mg, 0.13 mmol, 67% yield).

^{1}H NMR (500 MHz, $CDCl_3$): δ 8.06 (d, *J* = 2.5 Hz, 1H), 7.62 – 7.35 (m, 1H), 7.33 – 7.27 (m, 2H), 7.25 – 7.15 (m, 3H), 6.74 – 6.67 (m, 1H), 4.20 – 3.97 (m, 1H), 3.91 (s, 3H), 3.80 (t, *J* = 9.7 Hz, 1H), 3.76 – 3.52 (m, 1H), 2.87 (d, *J* = 10.9 Hz, 1H), 2.82 – 2.68 (m, 4H), 2.34 – 2.14 (m, 2H), 1.60 – 1.36 (m, 9H).

^{13}C NMR (126 MHz, $CDCl_3$, *mixture of rotamers*): δ = 167.8, 163.0, 155.9, 150.6, 145.7, 143.7, 138.2, 128.4, 126.5, 110.65, 79.48, 54.12, 54.04, 53.39, 49.36, 45.12, 40.93, 34.18, 29.04, 28.47.

IR (neat): 2974, 2942, 1688, 1492, 1027, 731 cm^{-1}.

HRMS (ESI, m/z): Calculated for $C_{23}H_{31}O_3N_2$ [M+H] $^+$: 383.2329, observed: 383.2329.

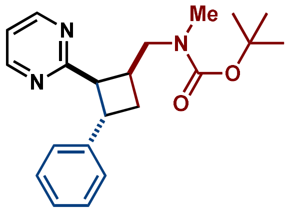

tert-butyl methyl(((1S,2R,3S)-3-phenyl-2-(pyrimidin-2-yl)cyclobutyl)methyl)carbamate (52): The title compound was prepared according to a slight modification of general procedure for Negishi cross coupling. To an oven-dried reaction tube with a stir bar inside, was added alkylboronic pinacol ester [XX] (40.1 mg, 0.100 mmol, 1.00 equiv.). The tube was capped with a rubber septum and backfilled and refilled with N_2 (X 3). Under N_2 atmosphere anhydrous THF (0.5 mL) was added to the tube with a syringe. The reaction vial was cooled to - 78 °C. *Tert*-butyl lithium (70.0 μL, 1.43 M, 1.00 equiv.) was added dropwise by a syringe and the reaction was allowed to warm to room temperature and further stir for 30 minutes. The reaction vial was transferred to the glovebox and added zinc acetate (19.0 mg, 0.105 mmol, 1.05 equiv.) and was allowed to stir at 60 ^{0}C for 4 hours. Upon completion, [Pd-G3]$_2$ (0.37 mg, 50 μmol, 0.0050 equiv.) and CPhos (0.87 mg, 0.020 mmol, 0.020 equiv.) was added as a THF solution (premixed for 10 min. in 0.2 mL THF), lithium chloride

(4.45 mg, 0.210 mmol, 1.05 equiv.), 2-chloropyrimidine (14.0 mg, 0.120 mmol, 1.20 equiv.) and toluene (0.5 mL). The vial was capped and sealed with Telfon tape, brought out of glovebox, and was allowed to stir at room temperature for 12 hours. Upon completion, the reaction mixture was filtered through a silica gel plug, rinsed with diethyl ether, and concentrated under reduced pressure. The resulting reaction mixture was purified by silica gel column chromatography (40% EtOAc in hexanes) to furnish the desired product. Colorless oil (15 mg, 42 μmol, 42% yield).

^{1}H NMR (500 MHz, CDCl$_3$*, mixture of rotamers*): δ 8.69 (d, *J* = 5.2 Hz, 2H), 7.29 (d, *J* = 2.9 Hz, 4H), 7.20 – 7.11 (m, 2H), 4.68 – 4.39 (m, 1H), 4.07 (t, *J* = 9.5 Hz, 1H), 3.74 (s, 1H), 3.08 (d, *J* = 13.1 Hz, 1H), 2.90 – 2.63 (m, 4H), 2.33 – 2.25 (m, 2H), 1.45 (d, *J* = 14.6 Hz, 9H).

^{13}C NMR (126 MHz, CDCl$_3$, *mixture of rotamers*): δ = 169.5, 157.0, 156.2, 144.6, 129.6, 128.4, 126.8, 126.2, 118.7, 79.5, 50.9, 49.1, 48.7, 39.6, 34.3, 34.0, 28.6.

IR (neat): 2972, 2932, 1688, 1425, 1138, 698 cm^{-1}.

HRMS (ESI, m/z): Calculated for $C_{21}H_{27}O_2N_3Na$ [M+Na] $^+$: 376.1995, observed: 376.2000.

4.5j Synthesis of GABA-analogue - 53:

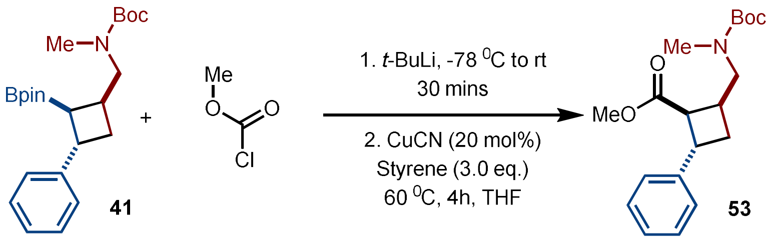

methyl (1R,2S,4S)-2-(((tert-butoxycarbonyl)(methyl)amino)methyl)-4-phenylcyclobutane-1-carboxylate (53): The title compound was prepared according to literature procedure. To an oven-dried reaction tube equipped with a magnetic stir bar was added alkyl boronic ester **41** (40.1 mg, 0.100 mmol, 1.00 equiv.). The tube was capped with a rubber septum and backfilled and refilled with N_2 (X 3). Under N_2 atmosphere anhydrous tetrahydrofuran (0.3 mL) was added to the tube with a syringe. The vial was cooled to -78 ^{o}C and *tert*-butyl lithium (65.4 μL, 1.53 M in pentane, 1.00 equiv.) was added dropwise by a syringe. The vial was then allowed to warm to room temperature and further stirred for 30 minutes. The reaction vial was then transferred into the glove box, styrene (31.2 mg, 0.300 mmol, 3.00 equiv), copper cyanide (1.80 mg, 0.020 mmol, 20 mol %) and methyl chlorocarbonate (11.3 mg, 0.120 mmol, 1.20 equiv.) were added. The vial was sealed with septum cap and removed from the glove box. The vial was heated to 60 ^{o}C (oil bath) and stirred for 4 hours. The reaction mixture was subsequently diluted with diethyl ether and was passed through a silica gel plug using diethyl ether as eluent. The solvent was removed under reduced pressure. The crude product was purified by column chromatography. (15% EtOAC in hexanes). Colorless oil (17 mg, 0.05 mmol, 51 % yield).

^{1}H NMR (500 MHz, CDCl$_3$, *mixture of rotamers*): δ 7.31 (t, *J* = 7.5 Hz, 2H), 7.25 – 7.18 (m, 3H), 4.03 (s, 1H), 3.82 – 3.74 (m, 1H), 3.69 (s, 3H), 3.32 (t, *J* = 9.4 Hz, 1H), 3.26 – 3.16 (m, 1H), 2.89-2.85 (m, 4H), 2.32 – 2.11 (m, 2H), 1.48 (s, 9H).

^{13}C NMR (126 MHz, CDCl$_3$, *mixture of rotamers*): δ = 172.9, 156.1, 143.4, 128.6, 126.8, 126.6, 79.8, 55.0, 51.7, 49.1, 46.6, 39.2, 34.3, 32.4, 28.6.
IR (neat): 2974, 1690, 1365, 1163, 751 cm^{-1}.
HRMS (ESI, m/z): Calculated for $C_{19}H_{27}O_4NNa$ [M+Na]$^+$: 356.1832, observed: 356.1834.

5.5k Synthesis of azabicyclo[3.1.0]hexanes-56:

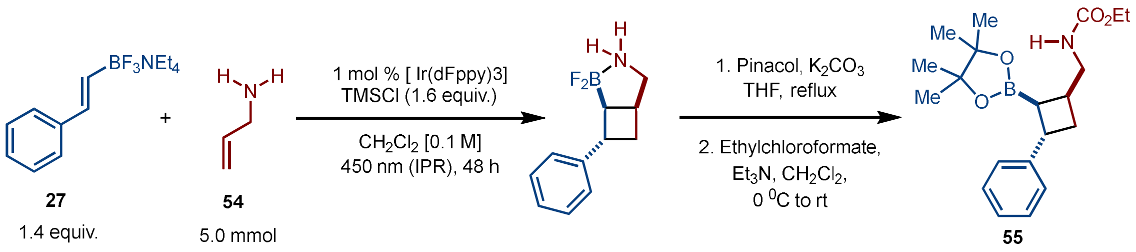

ethyl (((1S,2R,3S)-3-phenyl-2-(4,4,5,5-tetramethyl-1,3,2-dioxaborolan-2-yl)cyclobutyl)methyl)carbamate (55): In an 150 mL glass reaction vial with a stir bar inside was charged with BF$_3$ salt **27** (1.2 g, 3.9 mmol, 1.4 equiv.), Ir(dFppy)$_3$ (21 mg, 0.03 mmol, 0.01 equiv) and degassed and backfilled with N$_2$ (X 3). Then 30 mL of CH$_2$Cl$_2$ was added under N$_2$ atmosphere followed by the addition of TMSCl (0.55 ml, 4.4 mmol, 1.6 equiv) dropwise at rt and stirred it for 30 mins at rt . Then with a syringe, allyl amine **54** (0.16 g, 2.8 mmol, 1.0 equiv) was slowly added to the reaction vial under N$_2$ atmosphere, and the septum was properly sealed with electric tape. The reaction was allowed to be irradiated under 450 nm for 48h. The crude reaction mixture was transferred to a 100 mL round bottom flask washing with CH$_2$Cl$_2$ several times. After that all the volatiles were removed in vacuo. With a stir bar inside, pinacol (0.50 g, 4.2 mmol, 1.5 equiv) and anhyd. K$_2$CO$_3$ (0.58 g, 4.2 mmol, 1.5 equiv) were added, and a refluxing condenser was placed properly with the round bottom flask. 30 mL of anhyd. THF was added to the reaction mixture and the reaction was heated to reflux overnight (14 h). After that, the crude reaction mixture was passed through a celite pad to remove the solid. The filtrate was concentrated and in vacuo.

The round bottom flask with the reaction mixture was capped with a rubber septa and evacuated/back-filled three times with nitrogen. A nitrogen line was placed into it. Next under inert atmosphere CH$_2$Cl$_2$ (30 mL) was added to the reaction mixture. Next triethylamine (0.78 mL, 5.6 mmol, 2.0 equiv) was added and the reaction mixture was cooled to 0 °C for 5 mins. After that ethyl chloroformate (0.40 mL, 4.2 mmol, 1.5 equiv.) was added dropwise to the cold solution and the reaction mixture was stirred at that temperature for 5 more mins. Next, the flask was moved to room temperature and stirred for 18 hours. Upon completion, the reaction mixture was quenched with sat NH$_4$Cl solution and the aqueous layer was extracted with DCM (3 times, 20 ml each). The combined organic layers were washed with sat. NaHCO$_3$ solution and followed by brine. The organic layer was dried over Na$_2$SO$_4$ and filtered. The filtrate was evaporated to dryness and the crude mixture was purified by FCC (25% EtOAc in

hexanes) to afford the product as a colorless oil (410 mg, 1.17 mmol, 42% yield).

^{1}H NMR (500 MHz, $CDCl_3$*, mixture of rotamers*): δ 7.29 (dd, *J* = 8.6, 6.7 Hz, 2H), 7.25 – 7.20 (m, 2H), 7.19 – 7.13 (m, 1H), 5.52 – 5.18 (m, 1H), 4.12 (dq, *J* = 14.0, 6.8 Hz, 2H), 3.67 (q, *J* = 9.0 Hz, 1H), 3.58 – 3.45 (m, 1H), 3.36 (ddd, *J* = 13.5, 8.7, 4.8 Hz, 1H), 2.60 (q, *J* = 10.4 Hz, 1H), 2.40 (dt, *J* = 11.5, 8.9 Hz, 1H), 2.14 (qd, *J* = 9.1, 4.9 Hz, 2H), 1.27 (d, *J* = 3.5 Hz, 12H), 1.25 – 1.18 (m, 3H).
^{13}C NMR (126 MHz, $CDCl_3$, *mixture of rotamers*): δ = 156.9, 146.3, 128.2, 126.2, 125.7, 83.6, 60.6, 44.7, 38.1, 33.0, 31.7, 24.9, 24.6, 14.0.
IR (neat): 3361, 2977, 1701, 1240, 1142, 699 cm^{-1}.
HRMS (ESI, m/z): Calculated for $C_{20}H_{30}BNO_4Na$ $[M+Na]^+$: 382.2160, observed: 382.2169.

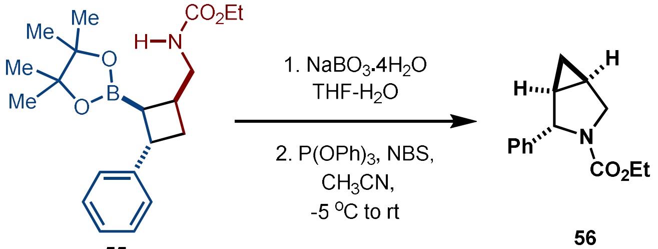

55

56

ethyl (1S,2S,5R)-2-phenyl-3-azabicyclo[3.1.0]hexane-3-carboxylate (56): The starting cyclobutylboronate (36 mg, 0.1 mmol) was dissolved in 2 mL (THF/H_2O 1:1) and was cooled to 0 ˚C using a ice/water bath. Solid sodium perborate (31 mg, 0.2 mmol, 2.0 equiv.) was added directly. Reaction was allowed to warm to room temperature and stir vigorously for 12 hours. Reaction was quenched with 2 mL of water. Aqueous layer was extracted three times with ethyl acetate. The collected organic layers were washed with brine, dried with magnesium sulfate, filtered, and concentrated under vacuum. Crude mixture was used in the next step without purification.
To a pre-cooled (-5°C to -10°C) solution of triphenyl phosphite (47 mg, 0.15 mmol, 1.5 eq.) in CH3CN (0.5 mL) in a glass reactor was added a solution of NBS (27 mg, 1.5 eq.) in CH3CN (0.5 mL) using an addition flask drop wise under a nitrogen atmosphere. It was ensured that the reaction mixture temperature was below 0°C during the addition. To the resulting solution was added a solution of the crude cyclobutyl alcohol (obtained previously after perborate oxidation) in CH3CN (1mL) using a syringe drop wise ensuring the reaction mixture temperature was below 0°C. The reaction was warmed to rt and stirred for 1 h. After the reaction was adjudged complete by TLC, all the volatiles were removed in vacuo, and the purified by silica gel column chromatography (15% Et_2O in hexanes) to obtain a colorless liquid which solidified after storing in a freezer overnight. 11 mg, 0.05 mmol, 51 % yield.
Note: The product has a similar polarity with triphenylphosphate, multiple column required to purify the product.

^{1}H NMR (500 MHz, $CDCl_3$, *mixture of rotamers*): δ 7.40 – 7.30 (m, 3H), 7.30 – 7.22 (m, 2H), 5.01 – 4.74 (m, 1H), 4.15 – 3.98 (m, 2H), 3.93 – 3.71 (m, 1H), 3.72 – 3.59 (m, 1H), 1.68 (dddt, *J* = 10.3, 6.2, 4.1, 1.9 Hz, 1H), 1.55 (ddd, *J* = 7.9, 6.7, 4.0 Hz, 1H), 1.26 – 1.02 (m, 3H), 0.85 – 0.77 (m, 1H), 0.36 (ddd, *J* = 9.1, 7.8, 4.3 Hz, 1H).
^{13}C NMR (126 MHz, $CDCl_3$, *mixture of rotamers*): δ = 155.6, 155.3, 143.2, 142.7, 128.5, 128.4, 127.2, 127.1, 126.7, 126.4, 62.93, 62.86, 61.98, 60.96, 48.7, 48.2, 23.5, 22.4, 16.1, 15.3, 14.7, 14.5, 9.7, 9.5.
IR (neat): 2917, 1696, 1413, 1111, 698 cm^{-1}.
HRMS (EI, m/z): Calculated for $C_{14}H_{17}NO_2$ $[M]^{+}$: 231.1254, observed: 231.1255.

Plausible mechanisms for the formation of 56:

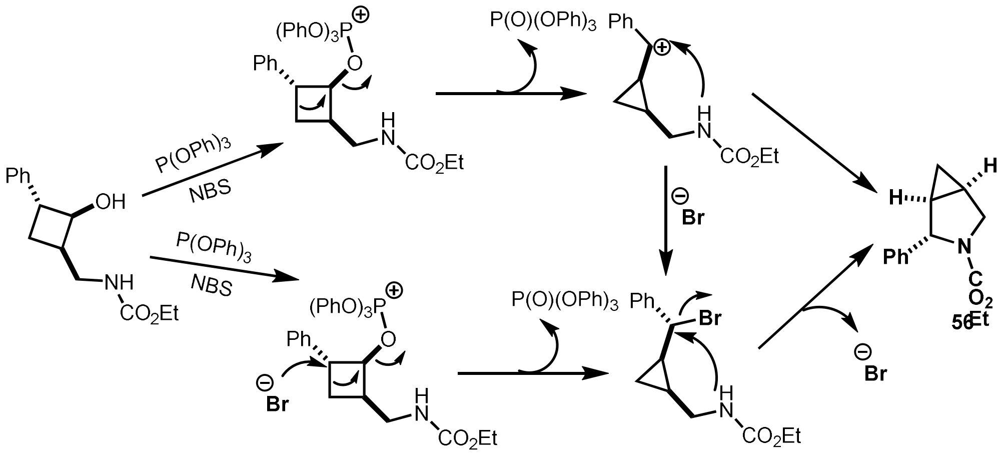

5.6 Discovery of Dearomative [4+2] Cycloadditions Reactions:

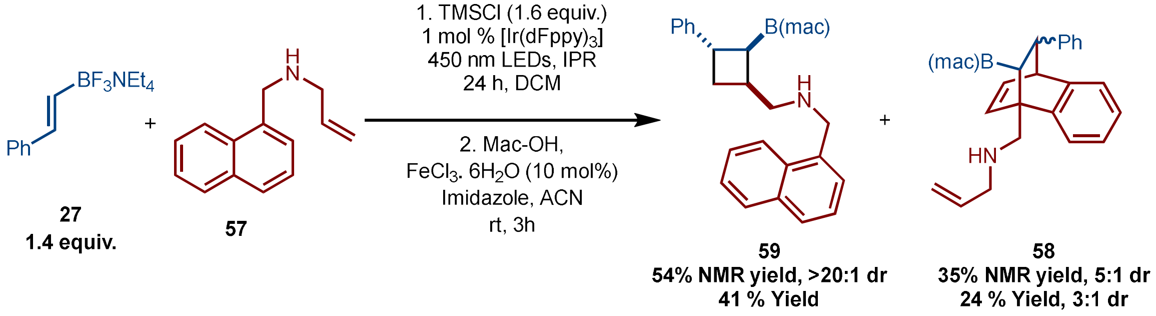

Procedure: Following the general procedure **2.** In a 2-dram vial with a stir bar inside was charged with Styrenyl-BF_3 salt **27** (84.3 mg, 0.280 mmol, 1.40 equiv), Ir(dFppy)$_3$ (1.53 mg, 2.00 µmol, 0.0100 equiv). The vial was capped with a rubber septum and degassed and backfilled with N_2 (X 3). Then 6 mL of DCM was added under N_2 atmosphere followed by the addition of TMSCl (40 µL, 0.32 mmol, 1.6 equiv) dropwise at room temperature and stirred it for 10 mins at rt. Then with a syringe, N-(naphthalen-1-ylmethyl)prop-2-en-1-amine **57** (39.4 mg, 0.200 mmol, 1.00 equiv) was slowly added

to the reaction vial under N_2 atmosphere, and the septum was quickly replaced with plastic cap and sealed with Teflon tape. The reaction was allowed to be irradiated under 450 nm LEDs in IPR for 24h. After that the volatiles were removed in vacuo and taken for crude NMR analysis using CH_2Br_2 as internal standard. Crude NMR analysis shows 54% NMR yield of [2+2] cycloadduct **59**, >20:1 dr, along with 35% NMR yield of [4+2] cycloadduct, **58**, 5:1 dr.

After removal of all the volatiles, Mac-OH (1.30 equiv, 55.6 mg, 0.260 mmol), Imidazole (3.00 equiv, 40.8 mg, 0.600 mmol), $FeCl_3{\cdot}6H_2O$ (5.5 mg, 0.020 mmol, 0.100 equiv.) was added to the crude reaction mixture follwed by addition of 2 mL acetonitrile. The reaction was allowed to stir in open air at room temperature for 3 h. After that, the reaction mixture was passed through a small celite plug. The mixture was then concentrated and purified with flash column chromatography with silica gel (2-10% Acetone in CH_2Cl_2).

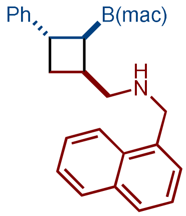

1-((1S,2R,3S)-2-((6bR,9aS)-6b,9a-dimethyl-6b,9a-dihydroacenaphtho[1,2-d][1,3,2]dioxaborol-8-yl)-3-phenylcyclobutyl)-N-(naphthalen-1-ylmethyl)methanamine (59): Isolated as a foamy white solid [43 mg, 82 μmol, 41 % yield].

^{1}H NMR (500 MHz, CDCl$_3$): δ 7.83 – 7.70 (m, 3H), 7.71 – 7.52 (m, 5H), 7.44 – 7.38 (m, 1H), 7.32 – 7.22 (m, 6H), 7.19 – 7.12 (m, 1H), 6.87 (d, *J* = 6.9 Hz, 1H), 6.41 (d, *J* = 8.4 Hz, 1H), 3.80 (q, *J* = 8.1 Hz, 1H), 3.45 (d, *J* = 14.7 Hz, 1H), 3.25 (d, *J* = 14.7 Hz, 1H), 2.62 – 2.47 (m, 2H), 2.43 – 2.30 (m, 2H), 1.96 (td, *J* = 10.1, 5.8 Hz, 2H), 1.75 (d, *J* = 16.3 Hz, 6H).

^{13}C NMR (126 MHz, CDCl$_3$): δ = 148.9, 148.4, 148.0, 135.0, 133.8, 131.5, 131.2, 131.1, 129.1, 128.94, 128.91, 128.2, 128.0, 127.0, 126.5, 126.0, 125.2, 125.1, 124.62, 124.58, 122.1, 119.4, 119.3, 90.1, 89.8, 50.8, 47.3, 37.4, 31.7, 30.2, 23.9, 23.5.

IR (neat): 2964, 2926, 1458, 1263, 1114, 889, 779 cm^{-1}.

HRMS (ESI, m/z): Calculated for $C_{36}H_{35}O_2NB$ $[M+H]^+$: 524.2755, Observed: 524.2776.

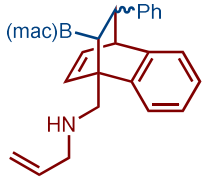

N-(((1R,4S,9R,10R)-10-(6b,9a-dimethyl-6b,9a-dihydroacenaphtho[1,2-d][1,3,2]dioxaborol-8-yl)-9-phenyl-1,4-ethanonaphthalen-1(4H)-yl)methyl)prop-

2-en-1-amine (58): The title compound was isolated as a foamy white solid, 26 mg, 50 µmol, 25% Yield, 3:1 dr.

^{1}H NMR (500 MHz, CDCl$_3$): δ 7.71 (dt, *J* = 8.4, 3.0 Hz, 2.6H), 7.54 (dt, *J* = 7.6, 4.1 Hz, 3.8H), 7.45 (dd, *J* = 6.8, 2.7 Hz, 1.3H), 7.33 – 7.27 (m, 0.8H) (min), 7.27 – 7.22 (m, 1.6H), 7.15 (td, *J* = 7.2, 1.4 Hz, 0.6H) (min), 7.08 (tdd, *J* = 6.9, 3.8, 1.9 Hz, 3.7H), 7.01 (d, *J* = 4.3 Hz, 2H), 6.93 (dd, *J* = 7.6, 6.2 Hz, 1H), 6.88 (d, *J* = 7.3 Hz, 1H), 6.76 (d, *J* = 7.2 Hz, 0.3H) (min), 6.69 – 6.63 (m, 2H), 6.54 (d, *J* = 7.6 Hz, 0.3H) (min), 6.42 (dd, *J* = 7.6, 5.8 Hz, 0.3H) (min), 6.07 (d, *J* = 7.6 Hz, 1H), 5.41 (ddt, *J* = 17.0, 10.1, 7.0 Hz, 1.3H), 4.95 (dd, *J* = 10.1, 1.5 Hz, 1.3H), 4.60 – 4.52 (m, 1.4H), 4.10 (dt, *J* = 6.0, 1.5 Hz, 0.3H) (min), 3.90 (dt, *J* = 6.4, 1.5 Hz, 1H), 3.75 (d, *J* = 12.0 Hz, 0.3H) (min), 3.50 (d, *J* = 11.2 Hz, 1H), 3.22 (dd, *J* = 7.6, 1.8 Hz, 1H), 3.03 (s, 0.3H) (min), 2.95 (d, *J* = 11.3 Hz, 1H), 2.71 – 2.51 (m, 1.3H), 2.34 (dd, *J* = 14.2, 6.3 Hz, 1.4H), 1.88 (dd, *J* = 14.5, 7.5 Hz, 1H), 1.81 – 1.71 (m, 0.3H) (min), 1.63 (s, 3H), 1.44 (s, 1H) (min), 1.36 (d, *J* = 3.6 Hz, 4H), 1.11 (d, *J* = 7.7 Hz, 0.3H) (min), 0.76 (d, *J* = 7.6 Hz, 1H).

^{13}C NMR (126 MHz, CDCl$_3$): δ = 149.1, 148.2, 147.6, 147.2(min), 146.9(min), 145.9, 142.3(min), 140.9, 140.6(min), 140.3, 134.91, 134.85(min), 133.8, 133.0(min), 132.3, 131.5(min), 131.32, 131.26(min), 128.63(min), 128.61, 128.55(min), 128.5, 128.21(min), 128.15, 127.94(min), 127.87, 126.2, 125.7(min), 125.6, 125.04(min), 124.99, 124.6(min), 124.5(min), 124.4, 124.3, 124.2, 122.3(min), 120.4, 120.0(min), 119.6(min), 119.3(min), 119.2, 119.0, 118.7(min), 116.3, 90.0, 89.5, 51.5, 51.4(min), 51.1(min), 50.9, 50.8, 50.0, 49.5(min), 48.4, 48.1(min), 24.1, 23.7(min), 22.6, 22.2(min).

IR (neat): 2976, 1468, 1264, 1153, 1114, 783, 684 cm^{-1}.

HRMS (ESI, m/z): Calculated for $C_{36}H_{35}O_2NB$ [M+H]$^+$: 524.2755, Observed: 524.2769.

5.7 Amine Directed Dearomative [4+2] Cycloadditions Reaction:

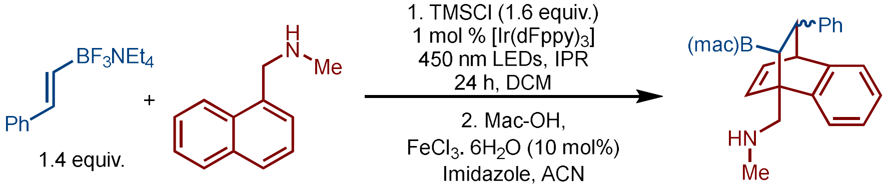

27 **60** **61**

1-((1R,4S,9R,10R)-10-((6bR,9aS)-6b,9a-dimethyl-6b,9a-dihydroacenaphtho[1,2-d][1,3,2]dioxaborol-8-yl)-9-phenyl-1,4-ethanonaphthalen-1(4H)-yl)-N-methylmethanamine (61): In a 2-dram vial with a stir bar inside was charged with StyrenylBF$_3$NEt$_4$ salt **27** (84 mg, 0.28 mmol, 1.4 equiv), Ir(dFppy)$_3$ (1.5 mg, 2.0 µmol, 0.010 equiv) and degassed and backfilled with N_2 (X 3). Then 6 mL of DCM was added under N_2 atmosphere followed by the addition of TMSCl (40 µL, 0.32 mmol, 1.6 equiv) dropwise at room temperature and stirred it for 10 mins at same temperature. Then with a syringe, a solution of N-methyl-1-(naphthalen-1-yl)methanamine **60** (34 mg, 0.20 mmol, 1.0 equiv) in 0.2 mL DCM was slowly added to the reaction vial under N_2 atmosphere, and the septum was quickly replaced with plastic cap and sealed with

teflon tape. The reaction was allowed to be irradiated under 450 nm in IPR for 18h. After that the volatiles were removed in vacuo and taken for crude NMR analysis using CH_2Br_2 as internal standard. Crude ^{1}H-NMR analysis shows 76% NMR yield of cycloadduct, 5:1 dr.

After that to the crude reaction mixture, Mac-OH (1.3 equiv, 56 mg, 0.26 mmol), Imidazole (3.0 equiv, 41 mg, 0.60 mmol), $FeCl_3.6H_2O$ (5.5 mg, 0.020 mmol, 0.10 equiv) was added to the crude reaction mixture followed by addition of 2 mL acetonitrile. The reaction was allowed to stir in open air at room temperature for 3 hours. After that, the reaction mixture was passed through a small silica plug. The mixture was then concentrated and purified with flash column chromatography with pre-basified silica gel with Et_3N. The title compound was isolated as a white foamy solid 60 mg, 0.13 mmol, 63%, 6:1 dr.

^{1}H NMR (500 MHz, $CDCl_3$): δ 7.73 – 7.65 (m, 2.3H), 7.57 – 7.47 (m, 3.3H), 7.46 (d, *J* = 6.7 Hz, 1.2H), 7.29 (t, *J* = 7.8 Hz, 0.5H) (min), 7.20 – 7.14 (m, 0.5H) (min), 7.12 – 7.04 (m, 4H), 7.01 (d, *J* = 3.5 Hz, 2H), 6.94 (dd, *J* = 7.6, 6.3 Hz, 1H), 6.85 (d, *J* = 7.3 Hz, 1H), 6.71 – 6.65 (m, 2H), 6.55 (dd, *J* = 7.5, 1.3 Hz, 0.15H)(min), 6.44 – 6.35 (m, 0.15H)(min), 6.07 (d, *J* = 7.6 Hz, 1H), 4.16 – 4.05 (m, 0.24H)(min), 3.90 (dd, *J* = 6.4, 1.6 Hz, 1H), 3.83 – 3.70 (m, 0.3H)(min), 3.43 (d, *J* = 10.7 Hz, 1H), 3.26 – 3.20 (m, 1H), 3.01 (dd, *J* = 17.6, 13.5 Hz, 1.3H), 2.65 (d, *J* = 12.1 Hz, 0.2H) (min), 2.07 (d, *J* = 18.0 Hz, 0.2H)(min), 1.62 (s, 3H), 1.41 (s, 3H), 1.37 (d, *J* = 7.7 Hz, 1H), 1.33 (s, 3.4H), 1.13 (d, *J* = 8.0 Hz, 0.2H)(min), 0.78 (d, *J* = 7.8 Hz, 1H).

^{13}C NMR (126 MHz, $CDCl_3$) (major diastereomer): δ 148.94, 147.99, 147.71, 145.79, 140.67, 134.84, 133.30, 131.09, 128.41, 128.36, 128.08, 128.03, 127.80, 127.76, 126.07, 125.49, 124.89, 124.16, 124.12, 118.98, 118.80, 116.06, 89.81, 89.31, 54.90, 50.73, 49.92, 48.32, 34.12, 23.95, 22.70.

IR (neat): 3388 (br), 2970, 1394, 1115, 1080 cm^{-1}.

HRMS (ESI, m/z): Calculated for $C_{34}H_{33}O_2NB$ [M+H]$^+$: 498.2599, found: 498.2606.

5.8 General Procedure 4: for Alcohol Directed Dearomative [4+2] Cycloadditions Reactions:

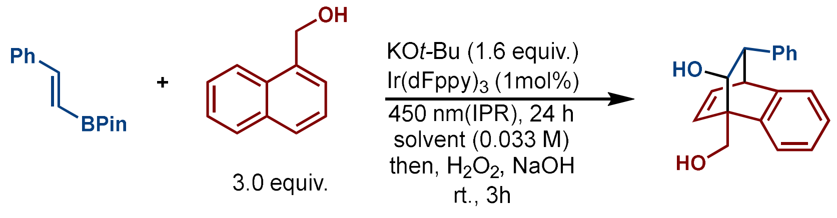

In a 2 dram vial with a stir bar inside it, Ir(dFppy)$_3$ (1.5 mg, 2.0 µmol, 1.0 mol %), styrenyl-Bpin (0.20 mmol, 1.0 equiv.) was charged, along with the naphthalene-methanol (0.60 mmol, 3.0 equiv.), then was taken inside the glovebox and KO*t*-Bu (36 mg, 0.32 mmol, 1.6 equiv.) was added to it and capped and taken outside the glovebox. Then the reaction vial cap was quickly replaced with a rubber septum and the reaction

mixture was degassed and filled with N_2 (X 3) followed by addition of the 6 mL of toluene. Then the rubber septum was quickly replaced by plastic cap and sealed with teflon. The vial was placed under the irradiation of visible light for 24 h in IPR. After that, the reaction mixture was transferred to a 25 mL round bottom flask containing 2 ml of 4N NaOH, washing with additional 2 mL of THF. Then 2 mL of 30% aqueous H_2O_2 was added to the solution and stirred at room temperature for additional 3h. Then the reaction mixture was extracted with EtOAc (3 X 5 mL), concentrated and taken for the ^{1}H-NMR analysis. The crude reaction mixture was further purified by FCC.

5.9 [4+2] Cycloadditions Products Characterization:

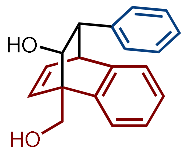

(1S,4S,9R,10R)-4-(hydroxymethyl)-10-phenyl-1,4-dihydro-1,4-ethanonaphthalen-9-ol (68): The title compound was prepared according to the general procedure **4** using styrenyl-Bpin **2** (46 mg, 0.20 mmol, 1.0 equiv.) and naphthalene-1-yl-methanol **67** (95 mg, 0.60 mmol, 3.0 equiv.). ^{1}H-NMR analysis of the crude reaction mixture shows 99% NMR yield, 5:1 dr. The crude reaction mixture was purified by silica gel FCC using hexane-EtOAc (1:1) as eluent. The product was isolated as a colorless solid (46 mg, 0.17 mmol, 83%, >20:1 dr).

^{1}H NMR (500 MHz, CDCl$_3$): δ 7.25 (d, *J* = 1.4 Hz, 1H), 7.19 (td, *J* = 7.6, 1.3 Hz, 1H), 7.15 – 7.01 (m, 4H), 6.92 (dd, *J* = 7.7, 6.2 Hz, 1H), 6.87 (dd, *J* = 7.3, 1.3 Hz, 1H), 6.46 (ddt, *J* = 16.4, 6.4, 1.6 Hz, 3H), 4.60 (d, *J* = 11.0 Hz, 1H), 4.49 (d, *J* = 11.0 Hz, 1H), 4.02 (dd, *J* = 3.5, 1.1 Hz, 1H), 3.71 (dt, *J* = 6.5, 1.7 Hz, 1H), 3.43 – 2.85 (b, 2H), 2.78 (t, *J* = 2.9 Hz, 1H).

^{13}C NMR (126 MHz, CDCl$_3$): δ = 142.4, 141.8, 141.3, 138.0, 132.4, 128.02, 127.96, 126.5, 125.9, 125.7, 125.6, 121.4, 81.5, 64.1, 57.6, 51.8, 48.3.

IR (neat): 3368 (br), 1600, 1473, 1010, 701 cm^{-1}.

HRMS (ESI, m/z): Calculated for $C_{19}H_{18}O_2Na$ [M+Na]$^+$: 301.1199, found: 301.1199.

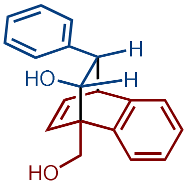

(1S,4S,9S,10R)-1-(hydroxymethyl)-9-phenyl-1,4-dihydro-1,4-ethanonaphthalen-10-ol (S52): The title compound was prepared according to the general procedure [**2.1**] using styrenyl-Bpin **2** (46 mg, 0.20 mmol, 1.0 equiv) and naphthalene-1-yl-methanol **67** (95 mg, 0.60 mmol, 3.0 equiv). The crude reaction mixture was purified by silica gel FCC using hexane-EtOAc (1:1) as eluent. The product was isolated as a colorless solid (8 mg, 0.03 mmol, 14%, >20:1 dr).

^{1}H NMR (500 MHz, CDCl$_3$): δ 7.52 (dd, *J* = 6.8, 1.7 Hz, 1H), 7.31 (d, *J* = 4.3 Hz, 4H), 7.29 – 7.27 (m, 1H), 7.25 – 7.18 (m, 3H), 6.63 (dd, *J* = 7.7, 5.8 Hz, 1H), 6.36 (dd, *J* =

7.8, 1.6 Hz, 1H), 4.63 (d, J = 11.0 Hz, 1H), 4.46 (d, J = 10.9 Hz, 1H), 4.23 (d, J = 3.7 Hz, 1H), 3.96 (dt, J = 5.9, 1.9 Hz, 1H), 2.58 (dd, J = 3.8, 2.1 Hz, 2H), 1.82 (s, 1H).
^{13}C NMR (126 MHz, $CDCl_3$): δ = 145.6, 143.6, 139.4, 135.7, 134.5, 128.4, 127.7, 126.6, 126.1, 125.7, 124.4, 122.8, 80.3, 63.8, 57.3, 52.9, 46.8.
IR (neat): 3366 (br), 1601, 1158, 1057, 729, 699 cm^{-1}.
HRMS (ESI, m/z): Calculated for $C_{19}H_{18}O_2Na$ [M+Na]$^+$: 301.1199, found: 301.1200.

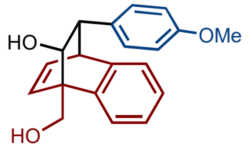

(1S,4S,9R,10R)-4-(hydroxymethyl)-10-(4-methoxyphenyl)-1,4-dihydro-1,4-ethanonaphthalen-9-ol (69): The title compound was prepared according to the general procedure **4** using styrenyl-Bpin **S1** (52 mg, 0.20 mmol, 1.0 equiv.) and naphthalene-1-yl-methanol **67** (95 mg, 0.60 mmol, 3.0 equiv.). ^{1}H-NMR analysis of the crude reaction mixture shows 89% NMR yield, 5:1 dr. The crude reaction mixture was purified by silica gel FCC using hexane-EtOAc (1:1) as eluent. The product was isolated as a colorless solid (47 mg, 0.15 mmol, 77%, >20:1dr).
^{1}H NMR (500 MHz, $CDCl_3$): δ 7.25 (d, J = 1.1 Hz, 1H), 7.19 (td, J = 7.5, 1.3 Hz, 1H), 7.06 (td, J = 7.3, 1.2 Hz, 1H), 6.93 (dd, J = 7.7, 6.2 Hz, 1H), 6.89 (dd, J = 7.3, 1.2 Hz, 1H), 6.66 – 6.59 (m, 2H), 6.49 (dt, J = 7.7, 1.2 Hz, 1H), 6.39 – 6.32 (m, 2H), 4.61 (d, J = 11.0 Hz, 1H), 4.50 (d, J = 11.0 Hz, 1H), 3.97 (d, J = 3.4 Hz, 1H), 3.71 (s, 3H), 3.69 (s, 1H), 3.27 (s, 1H), 2.88 (s, 1H), 2.74 (t, J = 2.9 Hz, 1H).
^{13}C NMR (126 MHz, $CDCl_3$): δ = 158.2, 141.9, 141.3, 138.0, 134.5, 132.3, 128.9, 125.9, 125.7, 125.6, 121.4, 113.4, 81.8, 64.1, 56.9, 55.2, 51.7, 48.5.
IR (neat): 3350 (br), 2972, 1512, 1455, 1017 cm^{-1}.
HRMS (ESI, m/z): Calculated for $C_{20}H_{20}O_3Na$ [M+Na]$^+$: 331.1305, found: 331.1306.

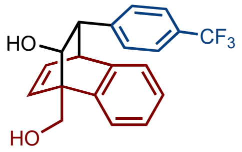

(1S,4S,9R,10R)-4-(hydroxymethyl)-10-(4-(trifluoromethyl)phenyl)-1,4-dihydro-1,4-ethanonaphthalen-9-ol (70): The title compound was prepared according to the general procedure **4** using styrenyl-Bpin **S2** (59.6 mg, 0.200 mmol, 1.00 equiv) and naphthalene-1-yl-methanol **67** (95 mg, 0.60 mmol, 3.0 equiv). ^{1}H-NMR analysis of the crude reaction mixture shows 74% NMR yield, 4.5:1 dr. The crude reaction mixture was purified by silica gel FCC using hexane-EtOAc (1:1) as eluent. The product was isolated as a colorless solid (38 mg, 0.11 mmol, 54%, >20:1 dr).
^{1}H NMR (500 MHz, $CDCl_3$): δ 7.34 (d, J = 8.1 Hz, 2H), 7.30 – 7.27 (m, 1H), 7.21 (td, J = 7.5, 1.3 Hz, 1H), 7.07 (td, J = 7.4, 1.2 Hz, 1H), 6.94 (dd, J = 7.7, 6.2 Hz, 1H), 6.87 (dd, J = 7.2, 1.3 Hz, 1H), 6.54 (d, J = 8.1 Hz, 2H), 6.50 (dt, J = 7.7, 1.3 Hz, 1H), 4.64

(d, J = 11.0 Hz, 1H), 4.54 (d, J = 11.0 Hz, 1H), 4.01 (d, J = 3.3 Hz, 1H), 3.72 (dt, J = 6.5, 1.7 Hz, 1H), 3.22 – 2.98 (m, 2H), 2.86 (t, J = 2.9 Hz, 1H).
^{13}C NMR (126 MHz, CDCl$_3$): δ = 146.5, 141.2, 141.1, 137.7, 132.7, 128.8 (q, J = 32.8, Hz), 128.2, 126.0, 125.9, 125.8, 124.9(q, J= 3.8 Hz), 124.2 (q, J= 272.2 Hz), 121.5, 81.2, 64.0, 57.3, 51.8, 47.9.
^{19}F NMR (471 MHz, CDCl$_3$): δ = -62.23.
IR (neat): 3369 (br), 2928, 1617, 1325, 1069 cm^{-1}.
HRMS (ESI, m/z): Calculated for $C_{20}H_{17}O_2F_3Na$ [M+Na]$^+$: 369.1073, found: 369.1073.

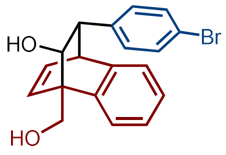

(1S,4S,9R,10R)-10-(4-bromophenyl)-4-(hydroxymethyl)-1,4-dihydro-1,4-ethanonaphthalen-9-ol (71): The title compound was prepared according to the general procedure **4** using styrenyl-Bpin **S3** (61.8 mg, 0.200 mmol, 1.00 equiv) and naphthalene-1-yl-methanol **67** (95 mg, 0.60 mmol, 3.0 equiv). ^{1}H-NMR analysis of the crude reaction mixture shows 65% NMR yield, 7:1 dr. The crude reaction mixture was purified by silica gel FCC using hexane-EtOAc (1:1) as eluent. The product was isolated as a colorless solid (38 mg, 0.11 mmol, 53%, >20:1 dr).
^{1}H NMR (500 MHz, CDCl$_3$): δ 7.25 (d, J = 1.2 Hz, 1H), 7.22 – 7.17 (m, 3H), 7.06 (td, J = 7.3, 1.3 Hz, 1H), 6.92 (dd, J = 7.7, 6.2 Hz, 1H), 6.86 (dd, J = 7.3, 1.2 Hz, 1H), 6.48 (dd, J = 7.7, 1.3 Hz, 1H), 6.29 (d, J = 8.4 Hz, 2H), 4.61 (d, J = 11.0 Hz, 1H), 4.51 (d, J = 11.0 Hz, 1H), 3.95 (d, J = 3.3 Hz, 1H), 3.79 – 3.57 (m, 1H), 3.19 (s, 1H), 3.04 (s, 1H), 2.74 (d, J = 2.9 Hz, 1H).
^{13}C NMR (126 MHz, CDCl$_3$): δ = 141.4, 141.3, 141.1, 137.7, 132.5, 131.1, 129.6, 125.9, 125.9, 125.7, 121.5, 120.4, 81.3, 64.0, 57.0, 51.8, 48.1.
IR (neat): 3340 (br), 1637, 1488, 1057, 731 cm^{-1}.
HRMS (ESI, m/z): Calculated for $C_{19}H_{17}O_2BrNa$ [M+Na]$^+$: 379.0304, found: 379.0303.

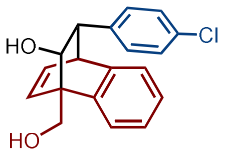

(1S,4S,9R,10R)-10-(4-chlorophenyl)-4-(hydroxymethyl)-1,4-dihydro-1,4-ethanonaphthalen-9-ol (72): The title compound was prepared according to the general procedure **4** using styrenyl-Bpin **S4** (52.9 mg, 0.200 mmol, 1.00 equiv) and naphthalene-1-yl-methanol **67** (95 mg, 0.60 mmol, 3.0 equiv). ^{1}H-NMR analysis of the crude reaction mixture shows 64% NMR yield, 4:1 dr. The crude reaction mixture was purified by silica gel FCC using hexane-EtOAc (1:1) as eluent. The product was isolated as a colorless solid (31 mg, 0.10 mmol, 50%, >20:1 dr).
^{1}H NMR (500 MHz, CDCl$_3$): δ 7.29 – 7.24 (m, 1H), 7.20 (td, J = 7.5, 1.3 Hz, 1H), 7.11 – 7.02 (m, 3H), 6.93 (dd, J = 7.7, 6.2 Hz, 1H), 6.86 (dd, J = 7.3, 1.2 Hz, 1H), 6.49 (dt,

J = 7.7, 1.2 Hz, 1H), 6.39 – 6.32 (m, 2H), 4.62 (d, *J* = 11.0 Hz, 1H), 4.52 (d, *J* = 11.0 Hz, 1H), 3.96 (d, *J* = 3.4 Hz, 1H), 3.68 (dt, *J* = 6.2, 1.7 Hz, 1H), 3.13 (s, 1H), 2.93 (s, 1H), 2.77 (t, *J* = 2.9 Hz, 1H).
^{13}C NMR (126 MHz, $CDCl_3$): δ = 141.4, 141.1, 140.9, 137.8, 132.5, 132.3, 129.2, 128.1, 125.93, 125.89, 125.7, 121.5, 81.4, 64.0, 57.0, 51.8, 48.1.
IR (neat): 3369 (br), 2958, 1599, 1492, 1013 cm^{-1}.
HRMS (ESI, m/z): Calculated for $C_{19}H_{17}O_2ClNa$ [M+Na]$^+$: 335.0809, found: 335.0808.

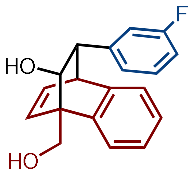

(1S,4S,9R,10R)-10-(3-fluorophenyl)-4-(hydroxymethyl)-1,4-dihydro-1,4-ethanonaphthalen-9-ol (73): The title compound was prepared according to the general procedure **4** using styrenyl-Bpin **S5** (49.6 mg, 0.200 mmol, 1.00 equiv) and naphthalene-1-yl-methanol **67** (95 mg, 0.60 mmol, 3.0 equiv). ^{1}H-NMR analysis of the crude reaction mixture shows 79% NMR yield, 4:1 dr. The crude reaction mixture was purified by silica gel FCC using hexane-EtOAc (1:1) as eluent. The product was isolated as a colorless solid (34 mg, 0.12 mmol, 57%, >20:1 dr).
^{1}H NMR (500 MHz, $CDCl_3$): δ 7.28 (s, 1H), 7.21 (td, *J* = 7.5, 1.2 Hz, 1H), 7.11 – 7.01 (m, 2H), 6.96 – 6.87 (m, 2H), 6.81 (td, *J* = 8.4, 2.6 Hz, 1H), 6.50 (d, *J* = 7.7 Hz, 1H), 6.32 (d, *J* = 7.7 Hz, 1H), 6.08 (dt, *J* = 10.5, 2.1 Hz, 1H), 4.64 (d, *J* = 11.0 Hz, 1H), 4.53 (d, *J* = 11.0 Hz, 1H), 3.98 (d, *J* = 3.8 Hz, 1H), 3.73 (dt, *J* = 6.4, 1.7 Hz, 1H), 3.15 (s, 1H), 3.01 – 2.91 (m, 1H), 2.79 (t, *J* = 2.9 Hz, 1H).
^{13}C NMR (126 MHz, $CDCl_3$): δ = 163.5, 161.5 145.1 (d, *J* = 3.8 Hz), 141.3 (d, *J* = 37.8 Hz), 137.8, 132.6, 129.4 (d, *J* = 8.8 Hz), 126.0, 125.9, 125.7, 123.7 (d, *J* = 2.5 Hz), 121.5, 114.7 (d, *J* = 21.4 Hz), 113.3 (d, *J* = 20.2 Hz), 81.4, 64.0, 57.3, 51.8, 47.9.
IR (neat): 3336 (br), 2970, 1638, 1248, 1045 cm^{-1}.
HRMS (ESI, m/z): Calculated for $C_{19}H_{17}O_2FNa$ [M+Na]$^+$: 319.1105, found: 319.1104.

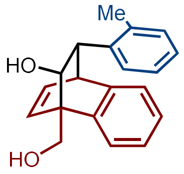

(1S,4S,9R,10R)-4-(hydroxymethyl)-10-(o-tolyl)-1,4-dihydro-1,4-ethanonaphthalen-9-ol (74): The title compound was prepared according to the general procedure **4** using styrenyl-Bpin **S6** (48.8 mg, 0.200 mmol, 1.00 equiv) and naphthalene-1-yl-methanol **67** (95 mg, 0.60 mmol, 3.0 equiv). ^{1}H-NMR analysis of the crude reaction mixture shows 73% NMR yield, 8:1 dr. The crude reaction mixture was purified by silica gel FCC using hexane-EtOAc (1:1) as eluent. The product was isolated as a colorless solid (35 mg, 0.12 mmol, 60%, >20:1 dr).

^{1}H NMR (500 MHz, CDCl$_3$): δ 7.27 (dd, *J* = 8.6, 1.8 Hz, 1H), 7.20 (td, *J* = 7.5, 1.3 Hz, 1H), 7.12 (d, *J* = 7.5 Hz, 1H), 7.06 – 6.94 (m, 3H), 6.75 (ddd, *J* = 16.7, 7.5, 1.4 Hz, 2H), 6.54 (dt, *J* = 7.8, 1.2 Hz, 1H), 5.45 (dd, *J* = 8.0, 1.3 Hz, 1H), 4.64 (d, *J* = 11.1 Hz, 1H), 4.53 (d, *J* = 11.1 Hz, 1H), 4.12 (s, 1H), 3.62 (dt, *J* = 6.4, 1.8 Hz, 1H), 3.21 (s, 1H), 3.10 (t, *J* = 2.8 Hz, 1H), 2.77 (s, 1H), 2.43 (s, 3H).
^{13}C NMR (126 MHz, CDCl$_3$): δ = 141.8, 141.1, 140.1, 138.0, 136.0, 132.4, 130.0, 126.4, 126.2, 125.9, 125.7, 125.6, 125.4, 121.3, 81.3, 64.3, 52.9, 51.8, 47.1, 20.1.
IR (neat): 3344 (br), 2956, 1637, 1471, 1050 cm^{-1}.
HRMS (ESI, m/z): Calculated for $C_{20}H_{20}O_2Na$ [M+Na]$^+$: 315.1356, found: 315.1356.

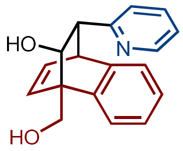

(1S,4S,9R,10R)-4-(hydroxymethyl)-10-(pyridin-2-yl)-1,4-dihydro-1,4-ethanonaphthalen-9-ol (75): The title compound was prepared according to the general procedure **4** using styrenyl-Bpin **S7** (46.2 mg, 0.200 mmol, 1.00 equiv) and naphthalene-1-yl-methanol **67** (95 mg, 0.60 mmol, 3.0 equiv). ^{1}H-NMR analysis of the crude reaction mixture shows 80% NMR yield, 4:1 dr. The crude reaction mixture was purified by silica gel FCC using EtOAc as eluent. The product was isolated as a colorless solid (34 mg, 0.12 mmol, 60%, >20:1 dr).
^{1}H NMR (500 MHz, CD$_3$OD_SPE): δ 8.36 (dd, *J* = 5.0, 1.7 Hz, 1H), 7.54 (d, *J* = 7.5 Hz, 1H), 7.42 (td, *J* = 7.8, 1.8 Hz, 1H), 7.20 – 7.11 (m, 2H), 6.97 (t, *J* = 7.4 Hz, 1H), 6.89 (dd, *J* = 7.8, 6.3 Hz, 1H), 6.73 (d, *J* = 7.3 Hz, 1H), 6.05 (d, *J* = 8.0 Hz, 1H), 4.54 (d, *J* = 11.1 Hz, 1H), 4.48 (d, *J* = 11.1 Hz, 1H), 4.09 (d, *J* = 3.3 Hz, 1H), 3.87 – 3.81 (m, 1H), 3.35 (s, 1H), 3.31 (d, *J* = 1.5 Hz, 1H), 3.13 (t, *J* = 3.0 Hz, 1H).
^{13}C NMR (126 MHz, CD$_3$OD_SPE): δ = 161.9, 147.7, 142.1, 141.6, 136.4, 136.1, 133.7, 125.2, 124.8, 124.7, 122.4, 121.6, 121.5, 76.6, 61.4, 58.8, 53.1, 47.2.
IR (neat): 3368 (br), 1593, 1473, 1060, 751 cm^{-1}.
HRMS (ESI, m/z): Calculated for $C_{18}H_{18}O_2N$ [M+H]$^+$: 280.1332, found: 280.1333.

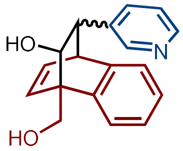

(1S,4S,9R,10R)-4-(hydroxymethyl)-10-(pyridin-3-yl)-1,4-dihydro-1,4-ethanonaphthalen-9-ol (76): The title compound was prepared according to the general procedure **4** using styrenyl-Bpin **S8** (46.2 mg, 0.200 mmol, 1.00 equiv) and naphthalene-1-yl-methanol **67** (95 mg, 0.60 mmol, 3.0 equiv) using THF (6.0 mL) as solvent. ^{1}H-NMR analysis of the crude reaction mixture shows 99% NMR yield, 4:1 dr. The crude reaction mixture was purified by silica gel FCC using MeOH-DCM (1:9) as eluent. The product was isolated as a mixture of diastereomers, colorless solid (52 mg, 0.19 mmol, 93% yield, and 3:1 dr).

^{1}H NMR (500 MHz, CD_3OD_SPE): δ 8.51 (s, 0.32H), 8.39 (d, *J* = 4.8 Hz, 0.34H), 8.24 (d, *J* = 5.1 Hz, 1H), 7.84 (dt, *J* = 8.1, 1.9 Hz, 0.39H), 7.80 (s, 1H), 7.57 (d, *J* = 7.5 Hz, 1H), 7.37 (dd, *J* = 7.9, 4.9 Hz, 0.36H), 7.30 (dd, *J* = 7.0, 1.7 Hz, 0.35H), 7.27 (dd, *J* = 6.8, 1.8 Hz, 0.36H), 7.21 (td, *J* = 7.5, 1.3 Hz, 1H), 7.15 (td, *J* = 6.9, 1.5 Hz, 0.63H), 7.10 (dd, *J* = 8.0, 4.9 Hz, 1H), 7.03 (td, *J* = 7.4, 1.2 Hz, 1H), 6.87 (dd, *J* = 7.8, 6.2 Hz, 1H), 6.81 (dd, *J* = 7.3, 1.2 Hz, 1H), 6.72 (dt, *J* = 8.1, 1.9 Hz, 1H), 6.63 (dd, *J* = 7.8, 1.6 Hz, 0.36H), 6.58 (dd, *J* = 7.8, 5.7 Hz, 0.35H), 6.39 (dt, *J* = 7.8, 1.3 Hz, 1H), 4.51 (d, *J* = 11.1 Hz, 1H), 4.45 (d, *J* = 10.8 Hz, 1.4H), 4.39 (d, *J* = 10.7 Hz, 0.36H), 4.10 (d, *J* = 4.0 Hz, 0.34H), 3.90 (dt, *J* = 5.7, 1.9 Hz, 0.33H), 3.85 (d, *J* = 3.3 Hz, 1H), 3.69 (dt, *J* = 6.5, 1.7 Hz, 1H), 3.61 – 3.53 (m, 0.24H), 3.35 (s, 1H), 2.90 (t, *J* = 2.9 Hz, 1H), 2.62 (dd, *J* = 4.1, 2.1 Hz, 0.34H).
^{13}C NMR (126 MHz, CD_3OD_SPE): δ 149.8, 149.7(min), 148.0(min), 147.8, 146.6(min), 143.6, 142.7, 142.3(min), 141.5(min), 141.0, 137.6, 137.4, 137.3(min), 135.5(min), 134.8, 126.8, 126.6(min), 126.49, 126.46 (min), 126.3, 125.0(min), 124.8(min), 124.5, 123.9, 123.6(min), 79.6, 78.8(min), 62.6, 61.8(min), 55.7, 54.83(min), 54.79(min), 54.5, 49.1, 47.7(min).
IR (neat): 3338 (br), 1637, 1470, 1047cm^{-1}.
HRMS (ESI, m/z): Calculated for $C_{18}H_{18}O_2N$ [M+H]$^+$: 280.1332, found: 280.1335.

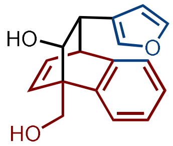

(1S,4S,9R,10R)-10-(furan-3-yl)-4-(hydroxymethyl)-1,4-dihydro-1,4-ethanonaphthalen-9-ol (77): The title compound was prepared according to the general procedure **4** using styrenyl-Bpin **S9** (44 mg, 0.20 mmol, 1.0 equiv) and naphthalene-1-yl-methanol **67** (95 mg, 0.60 mmol, 3.0 equiv) using Toluene (6.0 mL) as solvent. ^{1}H-NMR analysis of the crude reaction mixture shows 88% NMR yield, 5:1 dr. The crude reaction mixture was purified by silica gel FCC using hexane-EtOAc (1:1) as eluent. The product was isolated as a colorless solid (34 mg, 0.13 mmol, 63%, >20:1 dr).
^{1}H NMR (500 MHz, $CDCl_3$): δ 7.23 (d, *J* = 7.5 Hz, 1H), 7.20 – 7.13 (m, 2H), 7.08 (td, *J* = 7.3, 1.2 Hz, 1H), 7.03 (dd, *J* = 7.3, 1.3 Hz, 1H), 6.92 (dd, *J* = 7.7, 6.2 Hz, 1H), 6.88 (s, 1H), 6.49 (d, *J* = 7.7 Hz, 1H), 5.43 (d, *J* = 1.8 Hz, 1H), 4.63 (d, *J* = 11.0 Hz, 1H), 4.52 (dd, *J* = 11.4, 3.4 Hz, 1H), 3.84 (s, 1H), 3.75 (dt, *J* = 6.3, 1.8 Hz, 1H), 3.04 (s, 1H), 2.73 (d, *J* = 5.7 Hz, 1H), 2.68 (t, *J* = 2.9 Hz, 1H).
^{13}C NMR (126 MHz, $CDCl_3$): δ 142.5, 142.3, 141.0, 138.9, 137.7, 132.3, 126.65, 125.73, 125.62, 125.58, 121.4, 109.9, 81.8, 64.1, 51.6, 48.2, 47.3.
IR (neat): 3342 (br), 2960, 1637, 1477, 1278, 1018 cm^{-1}.
HRMS (ESI, m/z): Calculated for $C_{17}H_{16}O_3Na$ [M+Na]$^+$: 291.0992, found: 291.0992.

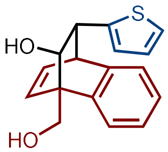

(1S,4S,9S,10R)-1-(hydroxymethyl)-9-(thiophen-2-yl)-1,4-dihydro-1,4-ethanonaphthalen-10-ol (78): The title compound was prepared according to the general procedure **4** using styrenyl-Bpin **S10** (47.2 mg, 0.200 mmol, 1.00 eq) and naphthalene-1-yl-methanol **67** (95 mg, 0.60 mmol, 3.0 equiv) using Toluene (6.0 mL) as solvent. ^{1}H-NMR analysis of the crude reaction mixture shows 93% NMR yield, 8:1 dr. The crude reaction mixture was purified by silica gel FCC using hexane-EtOAc (1:1) as eluent. The product was isolated as a colorless solid (41 mg, 0.15 mmol, 72%, >20:1 dr).

^{1}H NMR (500 MHz, CDCl$_3$): δ 7.26 – 7.16 (m, 2H), 7.13 – 7.03 (m, 2H), 7.00 (dd, *J* = 5.2, 1.2 Hz, 1H), 6.91 (dd, *J* = 7.7, 6.2 Hz, 1H), 6.78 (dd, *J* = 5.1, 3.5 Hz, 1H), 6.48 (dt, *J* = 7.8, 1.3 Hz, 1H), 6.33 (d, *J* = 3.5 Hz, 1H), 4.60 (d, *J* = 11.0 Hz, 1H), 4.52 – 4.46 (m, 1H), 4.00 (t, *J* = 3.5 Hz, 1H), 3.29 (s, 1H), 3.22 (d, *J* = 5.2 Hz, 2H), 3.08 (t, *J* = 2.9 Hz, 1H).

^{13}C NMR (126 MHz, CDCl$_3$): δ 146.1, 141.6, 141.2, 137.48, 132.47, 126.5, 126.3, 125.9, 125.6, 124.3, 123.5, 121.4, 83.0, 64.0, 52.6, 51.6, 48.2.

IR (neat): 3351 (br), 2988, 1637, 1394, 1057 cm^{-1}.

HRMS (ESI, m/z): Calculated for $C_{17}H_{16}O_2SNa$ [M+Na]$^+$: 307.0763, found: 307.0765.

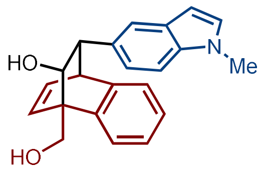

(1S,4S,9R,10R)-4-(hydroxymethyl)-10-(1-methyl-1H-indol-5-yl)-1,4-dihydro-1,4-ethanonaphthalen-9-ol (79): The title compound was prepared according to the general procedure **4** using alkenyl-Bpin **S11** (56.6 mg, 0.200 mmol, 1.00 equiv) and naphthalene-1-yl-methanol **67** (95 mg, 0.60 mmol, 3.0 equiv) using Toluene (6.0 mL) as solvent. ^{1}H-NMR analysis of the crude reaction mixture shows 88% NMR yield, 8:1 dr. The crude reaction mixture was purified by silica gel FCC using hexane-EtOAc (1:1) as eluent. The product was isolated as a colorless solid (45 mg, 0.14 mmol, 68%, >20:1 dr).

^{1}H NMR (500 MHz, CDCl$_3$): δ 7.26 – 7.15 (m, 2H), 7.09 – 7.00 (m, 2H), 6.99 – 6.92 (m, 2H), 6.87 (d, *J* = 7.3 Hz, 1H), 6.75 (d, *J* = 1.8 Hz, 1H), 6.51 (d, *J* = 7.7 Hz, 1H), 6.29 (d, *J* = 3.1 Hz, 1H), 6.25 (dd, *J* = 8.5, 1.8 Hz, 1H), 4.59 (d, *J* = 11.0 Hz, 1H), 4.49 (d, *J* = 11.0 Hz, 1H), 4.06 (d, *J* = 3.4 Hz, 1H), 3.79 – 3.72 (m, 1H), 3.69 (s, 3H), 3.31 (s, 1H), 2.89 – 2.84 (m, 1H), 2.81 (s, 1H).

^{13}C NMR (126 MHz, CDCl$_3$): δ 142.3, 141.5, 138.3, 135.6, 133.3, 132.1, 129.0, 128.2, 126.0, 125.6, 125.5, 121.9, 121.3, 120.1, 108.7, 100.7, 82.3, 64.3, 58.0, 51.7, 49.0, 32.8.

IR (neat): 3373 (br), 2924, 1722, 1490, 1056 cm^{-1}.

HRMS (ESI, m/z): Calculated for $C_{22}H_{21}O_2NNa$ [M+Na]$^+$: 354.1465, found: 354.1466.

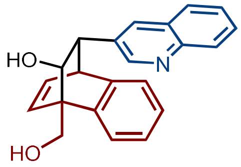

(1S,4S,9R,10R)-4-(hydroxymethyl)-10-(quinolin-3-yl)-1,4-dihydro-1,4-ethanonaphthalen-9-ol (80): The title compound was prepared according to the general procedure **4** using alkenyl-Bpin **S13** (56.3 mg, 0.200 mmol, 1.00 equiv) and naphthalene-1-yl-methanol **67** (95 mg, 0.60 mmol, 3.0 equiv) using THF (6.0 mL) as solvent. ^{1}H-NMR analysis of the crude reaction mixture shows 72% NMR yield, 11:1 dr. The crude reaction mixture was purified by silica gel FCC using MeOH-DCM (1:9) as eluent. The product was isolated as a colorless solid (43 mg, 0.13 mmol, 65%, >20:1 dr).

^{1}H NMR (500 MHz, CD_3OD_SPE): δ 8.07 (d, *J* = 2.3 Hz, 1H), 7.91 – 7.86 (m, 1H), 7.68 – 7.60 (m, 2H), 7.56 (dd, *J* = 8.4, 1.5 Hz, 1H), 7.48 (ddd, *J* = 8.1, 6.7, 1.2 Hz, 1H), 7.25 (td, *J* = 7.5, 1.3 Hz, 1H), 7.15 (d, *J* = 2.2 Hz, 1H), 7.01 (td, *J* = 7.4, 1.2 Hz, 1H), 6.90 (dd, *J* = 7.7, 6.2 Hz, 1H), 6.76 (dd, *J* = 7.3, 1.2 Hz, 1H), 6.43 (dt, *J* = 7.7, 1.2 Hz, 1H), 4.56 (d, *J* = 11.1 Hz, 1H), 4.49 (d, *J* = 11.1 Hz, 1H), 4.01 (d, *J* = 3.3 Hz, 1H), 3.76 (dt, *J* = 6.3, 2.0 Hz, 1H), 3.34 (s, 2H), 3.11 (t, *J* = 2.9 Hz, 1H).

^{13}C NMR (126 MHz, CD_3OD_SPE): δ 150.6, 145.9, 142.3, 141.5, 136.5, 136.1, 134.9, 133.5, 129.1, 127.8, 127.5, 127.1, 126.6, 125.5, 125.2, 125.0, 122.7, 78.3, 61.3, 54.5, 53.2, 47.9.

IR (neat): 2922, 1496, 1065, 752 cm^{-1}.

HRMS (ESI, m/z): Calculated for $C_{22}H_{20}O_2N$ $[M+H]^+$: 330.1489, found: 330.1491.

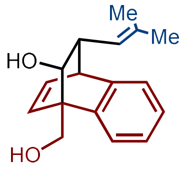

(1S,4S,9S,10R)-1-(hydroxymethyl)-9-(2-methylprop-1-en-1-yl)-1,4-dihydro-1,4-ethanonaphthalen-10-ol (81): The title compound was prepared according to the general procedure **4** using alkenyl-Bpin **S15** (41.6 mg, 0.200 mmol, 1.00 equiv) and naphthalene-1-yl-methanol **67** (95 mg, 0.60 mmol, 3.00 equiv) using Toluene (6.0 mL) as solvent. ^{1}H-NMR analysis of the crude reaction mixture shows 57% NMR yield, 17:1 dr. The crude reaction mixture was purified by silica gel FCC using EtOAc-hexane (1:1) as eluent. The product was isolated as a colorless solid (27 mg, 0.10 mmol, 52%, >20:1 dr).

^{1}H NMR (500 MHz, $CDCl_3$): δ 7.21 – 7.06 (m, 4H), 6.88 (dd, *J* = 7.8, 6.2 Hz, 1H), 6.47 (dt, *J* = 7.9, 1.3 Hz, 1H), 4.60 (d, *J* = 11.1 Hz, 1H), 4.50 (d, *J* = 11.1 Hz, 1H), 4.39 (dp, *J* = 9.7, 1.4 Hz, 1H), 3.57-3.55 (m, 2H), 2.78 (s, 1H), 2.39 (dt, *J* = 9.5, 2.7 Hz, 1H), 2.06 (s, 1H), 1.70 (d, *J* = 1.4 Hz, 3H), 1.59 (d, *J* = 1.5 Hz, 3H), 1.25 (d, *J* = 7.0 Hz, 1H).

^{13}C NMR (126 MHz, CDCl$_3$): δ 142.5, 140.9, 138.1, 133.0, 132.0, 126.4, 125.6, 125.4, 125.0, 121.3, 82.3, 64.4, 51.6, 50.7, 46.6, 25.7, 18.3.
IR (neat): 3326 (br), 1638, 1513, 1044, 731 cm^{-1}.
HRMS (ESI, m/z): Calculated for $C_{17}H_{20}O_2Na$ [M+Na]: 279.1356, found: 279.1356.

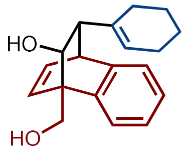

(1S,4S,9R,10R)-10-(cyclohex-1-en-1-yl)-4-(hydroxymethyl)-1,4-dihydro-1,4-ethanonaphthalen-9-ol (82): The title compound was prepared according to the general procedure **4** using alkenyl-Bpin **S14** (46.9 mg, 0.200 mmol, 1.00 equiv) and naphthalene-1-yl-methanol **67** (95 mg, 0.60 mmol, 3.0 equiv) using Toluene (6.0 mL) as solvent. ^{1}H-NMR analysis of the crude reaction mixture shows 95% NMR yield, 11:1 dr. The crude reaction mixture was purified by silica gel FCC using EtOAc-hexane (1:1) as eluent. The product was isolated as a colorless solid (47 mg, 0.16 mmol, 82%, >20:1 dr).
^{1}H NMR (500 MHz, CDCl$_3$): δ 7.18 (d, *J* = 7.3 Hz, 1H), 7.14 – 7.03 (m, 3H), 6.89 (dd, *J* = 7.7, 6.3 Hz, 1H), 6.43 (dt, *J* = 7.7, 1.3 Hz, 1H), 4.93 – 4.87 (m, 1H), 4.61 (d, *J* = 11.1 Hz, 1H), 4.50 (d, *J* = 11.1 Hz, 1H), 3.85 (d, *J* = 3.7 Hz, 1H), 3.75 (dt, *J* = 6.2, 1.7 Hz, 1H), 3.29 (s, 1H), 2.58 (s, 1H), 2.03 (t, *J* = 2.5 Hz, 1H), 1.85 – 1.59 (m, 4H), 1.53 – 1.40 (m, 4H).
^{13}C NMR (126 MHz, CDCl$_3$): δ 143.0, 141.2, 138.5, 137.2, 132.1, 125.4, 125.33, 125.26, 122.6, 121.1, 78.3, 64.2, 58.9, 51.6, 45.9, 28.4, 25.3, 23.1, 22.3.
IR (neat): 3368 (br), 2926, 1655, 1472, 1057 cm^{-1}.
HRMS (ESI, m/z): Calculated for $C_{19}H_{22}O_2Na$ $[M+Na]^+$: 305.1512, found: 305.1512.

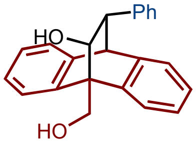

(9S,10S,11R,12R)-10-(hydroxymethyl)-12-phenyl-9,10-dihydro-9,10-ethanoanthracen-11-ol [**83**]: The title compound was prepared according to the general procedure **4** using styrenyl-Bpin **2** (46.0 mg, 0.200 mmol, 1.00 equiv) and anthracen-9-ylmethanol (130 mg, 0.600 mmol, 3.00 equiv), using THF (6 mL) as solvent. ^{1}H-NMR analysis of the crude reaction mixture shows 99% NMR yield, >20:1 dr. The crude reaction mixture was purified by silica gel FCC using hexane-EtOAc (1:1) as eluent. The product was isolated as a colorless solid (65 mg, 0.20 mmol, 97%, >20:1 dr).
^{1}H NMR (500 MHz, CDCl$_3$): δ 7.59 – 7.52 (m, 1H), 7.40 (d, *J* = 7.6 Hz, 1H), 7.38 – 7.32 (m, 1H), 7.24 – 7.18 (m, 3H), 7.18 – 7.13 (m, 3H), 7.09 (td, *J* = 7.5, 1.2 Hz, 1H), 7.02 (dd, *J* = 7.4, 1.4 Hz, 1H), 6.67 – 6.59 (m, 2H), 4.91 (dd, *J* = 11.2, 2.7 Hz, 1H),

4.85 (d, *J* = 11.1 Hz, 1H), 4.26 (t, *J* = 4.4 Hz, 1H), 4.13 (d, *J* = 2.3 Hz, 1H), 3.15 (s, 1H), 2.85 (dd, *J* = 3.8, 2.3 Hz, 1H), 2.51 (d, *J* = 6.4 Hz, 1H).
^{13}C NMR (126 MHz, CDCl$_3$): δ 144.9, 142.6, 141.4, 140.7, 138.7, 128.2, 128.0, 126.7, 126.5, 126.22, 126.21, 126.17, 126.1, 125.0, 123.4, 122.5, 80.0, 62.6, 57.4, 51.9, 51.5.
IR (neat): 3339 (br), 2936, 1634, 1455, 1042, 748 cm^{-1}.
HRMS (ESI, m/z): Calculated for $C_{23}H_{20}O_2Na$ [M+Na]$^+$: 351.1356, found: 351.1356.

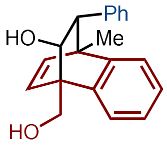

(1S,9S,10R)-1-(hydroxymethyl)-4-methyl-9-phenyl-1,4-dihydro-1,4-ethanonaphthalen-10-ol (84): The title compound was prepared according to the general procedure **4** using styrenyl-Bpin **2** (46.0 mg, 0.200 mmol, 1.00 equiv) and (4-methylnaphthalen-1-yl)methanol (103 mg, 0.600 mmol, 3.00 equiv). ^{1}H-NMR analysis of the crude reaction mixture shows 91% NMR yield, >20:1 dr. The crude reaction mixture was purified by silica gel FCC using hexane-EtOAc (1:1) as eluent. The product was isolated as colorless solid (50 mg, 0.17 mmol, 86%, >20:1 dr).
^{1}H NMR (500 MHz, CDCl$_3$): δ 7.28 (d, *J* = 1.5 Hz, 1H), 7.23 (td, *J* = 7.4, 1.2 Hz, 1H), 7.17 – 7.09 (m, 2H), 7.07 – 7.02 (m, 2H), 6.91 (dd, *J* = 7.5, 1.2 Hz, 1H), 6.57 (d, *J* = 7.6 Hz, 1H), 6.52 (dd, *J* = 7.7, 1.1 Hz, 1H), 6.24 (d, *J* = 7.6 Hz, 2H), 4.57 (d, *J* = 11.0 Hz, 1H), 4.47 (d, *J* = 9.8 Hz, 1H), 3.99 (s, 1H), 3.42 (s, 1H), 3.12 (d, *J* = 5.2 Hz, 1H), 2.54 (d, *J* = 3.4 Hz, 1H), 1.29 (s, 3H).
^{13}C NMR (126 MHz, CDCl$_3$): δ 144.5, 142.9, 141.9, 140.5, 133.0, 128.9, 127.7, 126.6, 125.6, 125.5, 123.2, 121.2, 83.9, 64.1, 63.3, 51.4, 46.3, 18.4.
IR (neat): 3365 (br), 2928, 1600, 1452, 1056 cm^{-1}.
HRMS (ESI, m/z): Calculated for $C_{20}H_{20}O_2Na$ [M+Na]$^+$: 315.1356, found: 315.1360.

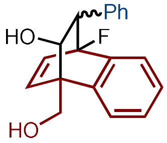

(1R,4S,9R,10R)-1-fluoro-4-(hydroxymethyl)-10-phenyl-1,4-dihydro-1,4-ethanonaphthalen-9-ol (85): The title compound was prepared according to the general procedure **4** using styrenyl-Bpin **2** (46.0 mg, 0.200 mmol, 1.00 equiv) and (4-fluoronaphthalen-1-yl)methanol **S48** (108 mg, 0.600 mmol, 3.00 equiv). ^{1}H-NMR analysis of the crude reaction mixture shows 96% NMR yield, 6:1 dr. The crude reaction mixture was purified by silica gel FCC using hexane-EtOAc (1:1) as eluent. The product was isolated as a mixture of diastereomers, colorless solid (50 mg, 0.16 mmol, 82%, 5:1 dr).
^{1}H NMR (500 MHz, CDCl$_3$): δ 7.54 (dd, *J* = 7.3, 1.4 Hz, 0.2H) (min), 7.38 (d, *J* = 7.3 Hz, 0.2H)(min), 7.34 – 7.26 (m, 2.72H), 7.17 (td, *J* = 7.7, 2.5 Hz, 2H), 7.12 – 7.05 (m, 3H), 6.89 (dd, *J* = 11.7, 8.3 Hz, 1H), 6.58 (t, *J* = 9.0 Hz, 0.2H)(min), 6.43 (ddd, *J* = 8.3, 4.5, 1.3 Hz, 1H), 6.39 – 6.30 (m, 2.2H), 4.55 (d, *J* = 10.9 Hz, 1.2H), 4.45 (d, *J* = 11.0

Hz, 1H), 4.35-4.30 (m, 0.4H)(min), 4.03 (t, *J* = 3.6 Hz, 1H), 3.29 (s, 2H), 3.10 (t, *J* = 3.4 Hz, 1H), 2.78 (t, *J* = 3.3 Hz, 0.2H)(min).

^{13}C NMR (126 MHz, CDCl$_3$)(major): δ 140.4 (d, *J* = 20.8 Hz), 138.0, 137.8, 137.5, 131.4 (d, *J* = 9.7 Hz), 129.2, 127.8, 127.2, 126.6, 126.0, 121.6 (d, *J* = 6.0 Hz), 121.3 (d, *J* = 3.2 Hz), 98.89, 97.31, 81.0 (d, *J* = 7.1 Hz), 63.5, 61.0 (d, *J* = 18.4 Hz), 51.1 (d, *J* = 2.7 Hz).

^{13}C NMR (126 MHz, CDCl$_3$)(minor): δ 143.8 (d, *J* = 20.1 Hz), 139.0, 137.0 (d, *J* = 7.0 Hz), 136.7 (d, *J* = 27.3 Hz), 132.0 (d, *J* = 10.5 Hz), 129.3, 128.3, 127.4, 126.3 (d, *J* = 8.6 Hz), 123.7 (d, *J* = 2.7 Hz), 118.5 (d, *J* = 6.9 Hz), 98.4, 96.8, 79.1 (d, *J* = 7.0 Hz), 62.5, 60.2 (d, *J* = 18.3 Hz), 52.2 (d, *J* = 2.5 Hz).

^{19}F NMR (471 MHz, CDCl$_3$): δ -184.90(major), -185.99 (minor).

IR (neat): 3375 (br), 2926, 1601, 1279, 1019, 757 cm^{-1}.

HRMS (ESI, m/z): Calculated for $C_{19}H_{17}O_2FNa$ [M+Na]$^+$: 319.1105, found: 319.1106.

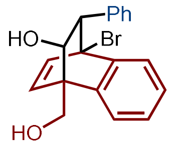

(4S,9R,10R)-1-bromo-4-(hydroxymethyl)-10-phenyl-1,4-dihydro-1,4-ethanonaphthalen-9-ol (86): The title compound was prepared according to the general procedure **4** using styrenyl-Bpin **2** (46.0 mg, 0.200 mmol, 1.00 equiv) and (4-bromonaphthalen-1-yl)methanol **S50** (142 mg, 0.600 mmol, 3.00 equiv). ^{1}H-NMR analysis of the crude reaction mixture shows 92% NMR yield, >20:1 dr. The crude reaction mixture was purified by silica gel FCC using hexane-EtOAc (1:1) as eluent. The product was isolated as a colorless solid (59 mg, 0.17 mmol, 84%, >20:1 dr).

^{1}H NMR (500 MHz, CDCl$_3$): δ 7.36 (dt, *J* = 7.5, 1.0 Hz, 1H), 7.33 – 7.28 (m, 2H), 7.24 – 7.19 (m, 1H), 7.19 – 7.14 (m, 1H), 7.13 – 7.04 (m, 3H), 6.53 (dd, *J* = 8.1, 1.4 Hz, 1H), 6.29 (d, *J* = 7.5 Hz, 2H), 4.65 (d, *J* = 11.0 Hz, 1H), 4.55 (d, *J* = 11.0 Hz, 1H), 4.16 – 4.09 (m, 1H), 3.12 (d, *J* = 3.2 Hz, 1H).

^{13}C NMR (126 MHz, CDCl$_3$): δ = 143.1, 140.7, 139.1, 138.7, 133.0, 129.5, 127.6, 127.3, 126.9, 126.4, 126.3, 121.1, 82.6, 67.0, 65.8, 63.7, 51.4.

IR (neat): 3367, 2924, 1454, 1057, 731 cm^{-1}.

HRMS (ESI, m/z): Calculated for $C_{19}H_{17}O_2BrNa$ [M+Na]$^+$: 379.0304, found: 379.0308.

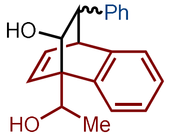

(1S,4S,9R,10R)-4-(1-hydroxyethyl)-10-phenyl-1,4-dihydro-1,4-ethanonaphthalen-9-ol (87): The title compound was prepared according to the general procedure **4** using styrenyl-Bpin **2** (46.0 mg, 0.200 mmol, 1.00 equiv) and 1-(naphthalen-1-yl)ethan-1-ol **S53** (103 mg, 0.600 mmol, 3.00 equiv). ^{1}H-NMR analysis of the crude reaction mixture shows 59% NMR yield, 4.5:1 dr. The crude reaction mixture was purified by silica gel FCC using hexane-EtOAc (1:1) as eluent. The product was isolated as mixture of diastereomers, colorless solid (33 mg, 0.11 mmol,

56% yield, and 3:1 dr).

^{1}H NMR (500 MHz, $CDCl_3$): δ 7.54 – 7.49 (m, 0.4H)(minor), 7.31 (d, *J* = 4.4 Hz, 1.4H), 7.25 - 7.16(m, 3.5H), 7.15 – 7.00 (m, 4.8H), 6.95 (t, *J* = 7.1 Hz, 1H), 6.92 – 6.87 (m, 0.3 H)(minor), 6.81 (d, *J* = 7.2 Hz, 1H), 6.63 (dd, *J* = 13.0, 7.8 Hz, 1.4H), 6.54 – 6.49 (m, 0.3H)(minor), 6.44 (dd, *J* = 7.9, 1.5 Hz, 0.4H)(minor), 6.30 (d, *J* = 7.3 Hz, 2H), 5.00 (q, *J* = 6.2 Hz, 1H), 4.92 (q, *J* = 6.4 Hz, 0.36H)(minor), 4.36 (d, *J* = 3.9 Hz, 0.5H)(minor), 4.03 (d, *J* = 3.2 Hz, 1H), 3.93 (dd, *J* = 5.6, 2.1 Hz, 0.6H)(minor), 3.73 – 3.66 (m, 1.3H), 2.83 – 2.74 (m, 1H), 2.55 (dd, *J* = 3.9, 2.1 Hz, 0.35H)(minor), 1.72 (d, *J* = 6.5 Hz, 0.47H)(minor), 1.57 (t, *J* = 6.8 Hz, 4H).

^{13}C NMR (126 MHz, $CDCl_3$): δ = 146.0 (minor), 143.9 (minor), 142.2, 142.1, 140.6, 139.8 (minor), 137.6 (minor), 137.1, 135.5 (minor), 133.5, 128.4 (minor), 127.92, 127.90, 127.8 (minor), 126.6 (minor), 126.4, 126.0, 125.9 (minor), 125.54, 125.50 (minor), 125.47, 125.3 (minor), 123.1, 122.7 (minor), 83.2 (minor), 78.6, 70.0, 66.5 (minor), 58.7, 56.9, 56.2 (minor), 54.5 (minor), 48.3, 46.9(minor), 20.4 (minor), 19.9.

IR (neat): 3306(br), 2925, 1492, 1452, 1154, 752 cm^{-1}.

HRMS (ESI, m/z): Calculated for $C_{20}H_{20}O_2Na$ $[M+Na]^+$: 315.1356, found: 315.1358.

5.10 Large Scale [4+2] Cycloadditions Reaction and Further Functionalization:

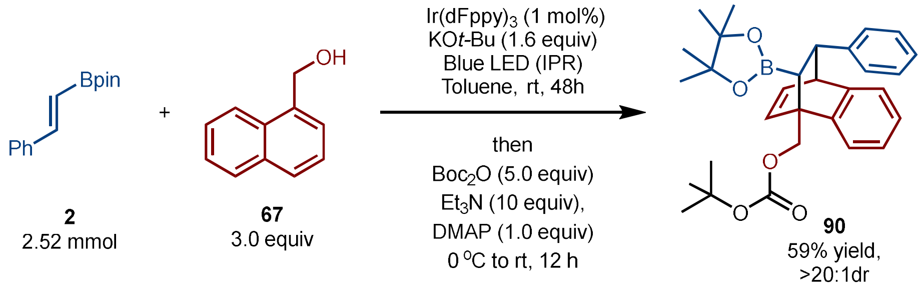

tert-butyl (((1R,4S,9R,10R)-9-phenyl-10-(4,4,5,5-tetramethyl-1,3,2-dioxaborolan-2-yl)-1,4-ethanonaphthalen-1(4H)-yl)methyl) carbonate (90): The title compound was made according to the following procedure. In a 120 mL glass photo-reaction bottle, with a stir bar inside it, $Ir(dFppy)_3$ (19.2 mg, 25.2 μmol, 1.00 mol%), *E*-styrenyl-Bpin **2** (580 mg, 2.50 mmol, 1.00 equiv.) was charged, along with the naphthalene-methanol **67** (1.2 g, 7.6 mmol, 3.0 equiv.). then the reaction flask was taken inside the glovebox and KO*t*-Bu (453 mg, 4.00 mmol, 1.60 equiv.) was added to it and capped with a rubber septum and taken outside the glovebox. Then the reaction mixture was degassed and backfilled with N_2 (X 3) and the solvent was added, capped and sealed with Teflon. Put under the irradiation of visible light inside IPR for 48 h. After that the reaction flask was cooled to 0 °C in an ice-water bath. Triethylamine (3.50 mL, 25.0 mmol, 10.0 equiv) was added via syringe under N_2 followed by dropwise addition of a solution of Boc_2O (2.80 g, 12.5 mmol, 5.00 equiv) in 10 mL of toluene, and a solution of DMAP (0.3 g, 2.5 mmol, 1.0 equiv), in 5 mL of Toluene under N_2 atmosphere. The reaction mixture was allowed to be stirred at room temperature for 12 hours. Then the

mixture was transferred to a separatory funnel and washed with H_2O (20 mL). The layers were separated, and aqueous phase was extracted with ethyl acetate (3 x 20 mL). The combined organic layers were washed with brine (25 mL), dried over anhydrous MgSO4, filtered and concentrated by rotary evaporator. The crude reaction mixture was purified by FCC (5% EtOAc in hexanes) to afford the product as a white solid (720 mg, 1.48 mmol, 59% yield, >20:1 dr). Stain with hanessian or PMA.

^{1}H NMR (500 MHz, $CDCl_3$): δ 7.32 (d, *J* = 7.6 Hz, 1H), 7.20 (t, *J* = 7.5 Hz, 1H), 7.09 – 7.00 (m, 4H), 6.87 (d, *J* = 7.2 Hz, 1H), 6.79 (dd, *J* = 7.6, 6.4 Hz, 1H), 6.47 (dd, *J* = 6.8, 2.9 Hz, 2H), 6.34 (d, *J* = 7.7 Hz, 1H), 4.96 (d, *J* = 11.1 Hz, 1H), 4.90 (d, *J* = 11.1 Hz, 1H), 3.81 – 3.76 (m, 1H), 3.27 (dd, *J* = 6.6, 2.3 Hz, 1H), 1.51 (s, 9H), 1.20 (d, *J* = 8.4 Hz, 12H).

^{13}C NMR (126 MHz, $CDCl_3$): δ 154.0, 146.1, 144.3, 140.9, 137.5, 134.5, 127.9, 127.8, 126.0, 125.7, 125.4, 124.6, 119.7, 83.4, 81.9, 68.0, 50.0, 48.9, 47.7, 27.9, 24.8, 24.7.

IR (neat): 2978, 1740, 1472, 1278, 1105, 849 cm^{-1}.

HRMS (ESI, m/z): Calculated for $C_{20}H_{37}O_5BNa$ $[M+Na]^+$: 511.2626, found: 511.2632.

4.10a Metal-free Cross Coupling:

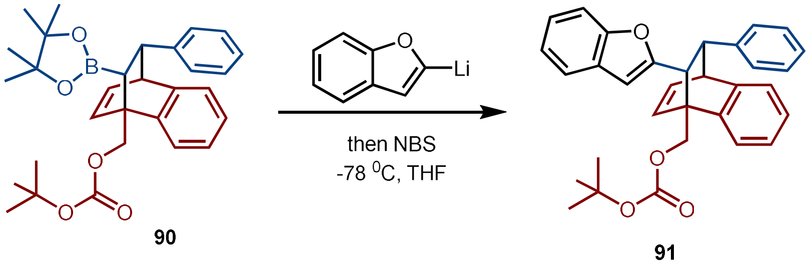

40% yield, >20:dr

((1S,4S,9S,10R)-10-(benzofuran-2-yl)-9-phenyl-1,4-ethanonaphthalen-1(4H)-yl)methyl tert-butyl carbonate (91): The title compound was made according to the following procedure. A reaction tube with a stir bar was flamed-dried under vacuum. Once cool, the tube was refilled with N_2 and evacuated and backfilled with N_2 three times. Once under N_2, the tube was sealed with a septum. The reaction tube was evacuated and backfilled with N_2 three times. Under N_2 atmosphere, the tube was charged with benzofuran (16.0 µL, 0.150 mmol, 1.50 equiv.) and THF (0.5 mL) sequentially via syringe. The mixture was cooled to -78 ^{o}C in a dry ice/acetone bath, and then *n*-butyllithium (65.0 µL, 2.30 M in hexanes, 0.150 mmol, 1.50 equiv.) was added dropwise via syringe. The mixture was warmed to room temperature and stirred for 1 h, before being cooled to -78 ^{o}C. A solution of borylated cycloadduct **90** (49 mg, 0.10 mmol, 1.0 equiv.) in THF (0.5 mL) was then added dropwise via syringe to the solution of lithiated benzofuran at -78 ^{o}C, and stirred for 1 h at this temperature. A solution of NBS (36.0 mg, 0.200 mmol, 2.00 equiv.) in THF (0.5 mL) to the reaction mixture at -78 ^{o}C, and stirred for 1 h at this temperature. After 1 h, the reaction was quenched with 20% aq. $Na_2S_2O_3$ (3 mL) and warmed to room temperature. The layers were separated, and the aqueous layer was extracted with ethyl acetate (3 X 3 mL), the combined organic layers were dried over $MgSO_4$, gravity filtered, and concentrated

in vacuo. The crude reaction mixture was purified by FCC (2% EtOAc in hexanes) to obtain the product as a colorless oil 19.0 mg, 0.04 mmol, 40% yield, >20:1 dr.

^{1}H NMR (600 MHz, $CDCl_3$): δ 7.45 – 7.39 (m, 3H), 7.28 (td, *J* = 7.6, 1.3 Hz, 1H), 7.23 – 7.19 (m, 1H), 7.18 – 7.12 (m, 2H), 7.11 – 7.03 (m, 4H), 7.02 (dd, *J* = 7.3, 1.2 Hz, 1H), 6.57 – 6.52 (m, 2H), 6.44 – 6.38 (m, 2H), 4.67 (d, *J* = 11.3 Hz, 1H), 4.57 (d, *J* = 11.3 Hz, 1H), 3.92 (dt, *J* = 6.5, 1.7 Hz, 1H), 3.47 (dd, *J* = 6.5, 2.1 Hz, 1H), 3.16 (d, *J* = 6.4 Hz, 1H), 1.44 (s, 9H).

^{13}C NMR (126 MHz, $CDCl_3$): δ = 158.1, 154.9, 153.7, 144.1, 142.5, 141.1, 138.2, 133.3, 128.5, 128.0, 127.8, 126.6, 126.0, 125.9, 125.4, 123.6, 122.6, 120.9, 120.4, 111.1, 104.6, 82.1, 66.8, 53.8, 49.9, 48.9, 48.5, 27.8.

IR (neat): 1634, 1279, 1160 cm^{-1}.

HRMS (ESI, m/z): Calculated for $C_{32}H_{30}O_4Na$ $[M+Na]^+$: 501.2036, found: 501.2039.

5.10b Homologation:

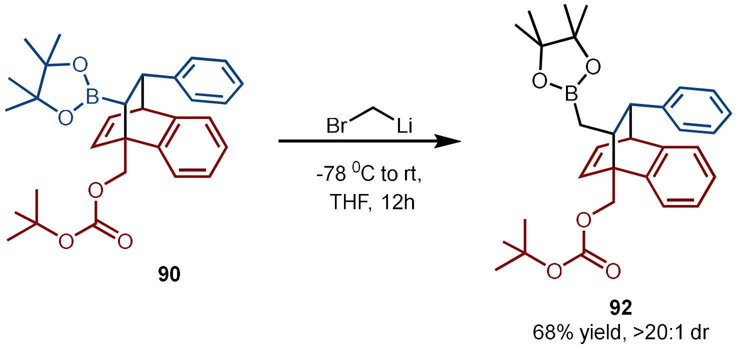

tert-butyl (((1S,4S,9R,10R)-9-phenyl-10-((4,4,5,5-tetramethyl-1,3,2-dioxaborolan-2-yl)methyl)-1,4-ethanonaphthalen-1(4H)-yl)methyl) carbonate (92): The title compound was made according to the following procedure. A reaction tube with a stir bar was flamed-dried under vacuum. Once cool, the tube was refilled with N_2 and evacuated and backfilled with N_2 three times. Once under N_2, the tube was sealed with a septum. The septum was briefly removed and was charged with borylated cycloadduct **90** (40 mg, 0.080 mmol, 1.00 equiv.) and evacuated and backfilled with N_2 X 3. THF (1.5 mL) and dibromomethane (14 μL, 0.20 mmol, 2.5 equiv.) were added sequentially via syringe and the mixture was cooled to -78 ^{o}C in a dry ice/acetone bath. *n*-butyllithium (78 μL, 2.3 M in hexanes, 0.18 mmol, 2.2 equiv.) was added dropwise via syringe over 10 minutes. The reaction was stirred at -78 ^{o}C for 5 minutes and then warmed to room temperature and stirred for 18 h. The reaction was quenched with H_2O (3 mL), the layers were separated, and the aqueous layer was extracted with ethyl acetate (3 X 3 mL). The combined organic layers were dried over MgSO4, gravity filtered, and concentrated in vacuo. The crude reaction mixture was purified by FCC (5% EtOAc in hexanes) to afford the product as a colorless oil 28 mg, 56 μmol, 68% yield, >20:1 dr.

^{1}H NMR (500 MHz, CDCl$_3$): δ 7.33 (d, *J* = 7.5 Hz, 1H), 7.19 (td, *J* = 7.5, 1.2 Hz, 1H), 7.07 – 7.01 (m, 3H), 6.99 (td, *J* = 7.4, 1.1 Hz, 1H), 6.79 – 6.69 (m, 2H), 6.47 – 6.42 (m, 2H), 6.25 (d, *J* = 7.7 Hz, 1H), 4.95 – 4.86 (m, 2H), 3.59 (dt, *J* = 6.3, 1.8 Hz, 1H), 2.70 (dd, *J* = 5.4, 2.3 Hz, 1H), 2.07 – 1.98 (m, 1H), 1.52 (s, 9H), 1.07 (dd, *J* = 14.0, 4.0 Hz, 1H), 1.02 (s, 6H), 0.81 (s, 6H), 0.68 (dd, *J* = 13.9, 10.8 Hz, 1H).
^{13}C NMR (126 MHz, CDCl$_3$): δ 154.2, 144.5, 143.6, 140.8, 136.9, 132.9, 128.5, 127.6, 126.0, 125.53, 125.45, 124.8, 120.8, 83.0, 82.1, 67.2, 55.4, 50.1, 49.6, 43.8, 27.9, 24.8, 24.5.
IR (neat): 2978, 1738, 1369, 1280, 1104 cm^{-1}.
HRMS (ESI, m/z): Calculated for $C_{31}H_{39}O_5BNa$ [M+Na]$^+$: 525.2783, found: 525.2792.
5.10c Zweifel Olefination:

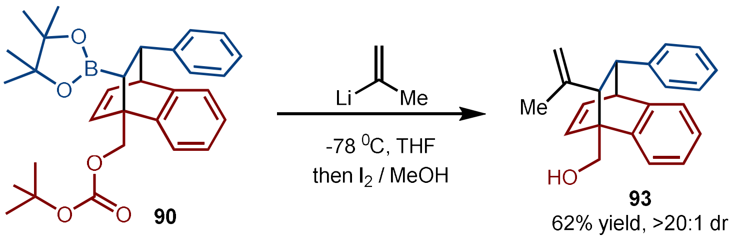

((1S,4S,9S,10S)-9-phenyl-10-(prop-1-en-2-yl)-1,4-ethanonaphthalen-1(4H)-yl)methanol (93): The title compound was made according to the following procedure. A reaction tube with a stir bar was flamed-dried under vacuum. Once cool, the tube was refilled with N_2 and evacuated and backfilled with N_2 three times. Once under N_2, the tube was sealed with a septum. The reaction tube was evacuated and backfilled with N_2 three times. Under N_2 atmosphere, the tube was charged with 2- bromoprop-1-ene (55.0 µL, 0.620 mmol, 4.10 equiv.). THF (1.5 mL) was added, and the solution cooled to -78 °C in a dry ice/acetone bath. *t*-BuLi (0.420 mL, 1.43 M in pentane, 0.600 mmol, 4.00 equiv.) was added dropwise and the solution stirred at -78 °C for 30 min. At this time, a solution of Borylated cycloadduct **90** (74 mg, 0.15 mmol, 1.0 equiv.) dissolved in THF (0.50 mL) was added dropwise at -78 °C and the mixture was allowed to stir at -78 °C for 1 hour. The reaction was then warmed to -40 °C by switching to a dry ice/acetonitrile bath and allowed to stir for 1 hour. The reaction was cooled back to -78 °C and a solution of I_2 (152 mg, 0.600 mmol, 4.00 equiv.) dissolved in MeOH (2.50 mL) was added dropwise down the side of the flask. Upon complete addition, the reaction was allowed to stir at -78 °C for 45 minutes then allowed to warm to room temperature and stir for an additional 1 hour. The reaction was quenched upon the addition of $Na_2S_2O_3$ (sat. aqueous solution, 3.0 mL) and stirred for another 1 h. The organic layer was separated, and the aqueous phase extracted with EtOAc (3 x 10 mL). The combined organic layers were dried over MgSO4, filtered, and concentrated.

Crude material was purified by silica gel column chromatography with 15% EtOAc in hexane as the eluent to give the alcohol as a colorless foamy solid 28 mg, 93 μmol, 62 % yield, >20:1 dr.

^{1}H NMR (500 MHz, CDCl$_3$): δ 7.52 (d, *J* = 7.5 Hz, 1H), 7.29 – 7.22 (m, 1H), 7.15 – 7.06 (m, 4H), 6.99 (dd, *J* = 7.3, 1.3 Hz, 1H), 6.87 (dd, *J* = 7.7, 6.4 Hz, 1H), 6.60 – 6.52 (m, 2H), 6.34 (d, *J* = 7.7 Hz, 1H), 4.78 (t, *J* = 1.9 Hz, 1H), 4.66 (d, *J* = 2.3 Hz, 1H), 4.40 – 4.31 (m, 2H), 3.80 (dt, *J* = 6.4, 1.7 Hz, 1H), 2.95 (dd, *J* = 6.8, 2.0 Hz, 1H), 2.46 (d, *J* = 6.8 Hz, 1H), 1.95 (s, 1H), 1.76 (d, *J* = 1.4 Hz, 3H).

^{13}C NMR (126 MHz, CDCl$_3$): δ = 146.0, 145.7, 143.6, 141.8, 137.7, 133.5, 128.0, 127.9, 126.3, 126.0, 125.6, 124.9, 120.3, 115.3, 63.6, 57.6, 51.6, 51.0, 48.6, 20.6.

IR (neat): 3359 (br), 2921, 1636, 1494, 1145, 801 cm^{-1}.

HRMS (ESI, m/z): Calculated for $C_{22}H_{22}ONa$ [M+Na]$^+$: 325.1563, found: 325.1561.

5.10d Hydrogenation:

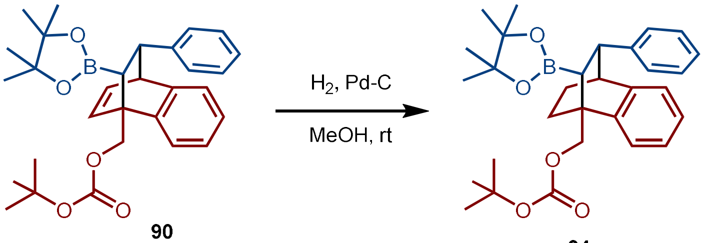

90

94
99% yield, >20:1 dr

tert-butyl (((1S,2R,3R,4S)-3-phenyl-2-(4,4,5,5-tetramethyl-1,3,2-dioxaborolan-2-yl)-3,4-dihydro-1,4-ethanonaphthalen-1(2H)-yl)methyl) carbonate (94): The title compound was made according to the following procedure. An oven-dried 2-dram vial, equipped with a stir bar was cooled under vacuum. Borylated cycloadduct **90** (49 mg, 0.10 mmol, 1.0 equiv.) and Pd/C 10% (20 mg) were added into the vial. After evacuated/backfilled with N_2 (X 3), dry MeOH (1 mL) was added. After degassing the solution with H_2 using a needle connected to H_2 balloon, the reaction mixture was stirred at room temperature for 24 h with H_2 balloon attached. After that, the reaction mixture was filtered throught a pad of celite. The filtrate was concentrated by rotary evaporation and then under high vac to obtain the product as a foamy solid 49 mg, 0.1 mmol, 99% yield, >20:1 dr.

^{1}H NMR (500 MHz, CDCl$_3$): δ 7.32 – 7.28 (m, 2H), 7.18 (dp, *J* = 8.3, 3.9 Hz, 1H), 7.07 (q, *J* = 3.4 Hz, 3H), 6.98 (d, *J* = 7.3 Hz, 1H), 6.61 (dd, *J* = 7.0, 2.6 Hz, 2H), 4.62 (d, *J* = 11.0 Hz, 1H), 4.53 (d, *J* = 11.1 Hz, 1H), 3.36 (dd, *J* = 7.4, 1.9 Hz, 1H), 3.01 (q, *J* = 2.6 Hz, 1H), 2.16 – 1.99 (m, 2H), 1.51 (s, 9H), 1.26 (d, *J* = 12.4 Hz, 12H).

^{13}C NMR (126 MHz, CDCl$_3$): δ 154.0, 147.0, 144.8, 141.1, 127.9, 127.6, 126.2, 126.0, 125.77, 125.75, 120.8, 83.3, 81.6, 70.2, 47.4, 42.2, 40.6, 27.9, 25.3, 24.94, 24.88.

IR (neat): 2977, 1739, 1346, 1254, 969 cm^{-1}.

HRMS (ESI, m/z): Calculated for $C_{30}H_{39}O_5BNa$ [M+Na]$^+$: 513.2783, found: 513.2790.

5.11 Determination of stereochemistry for 87:

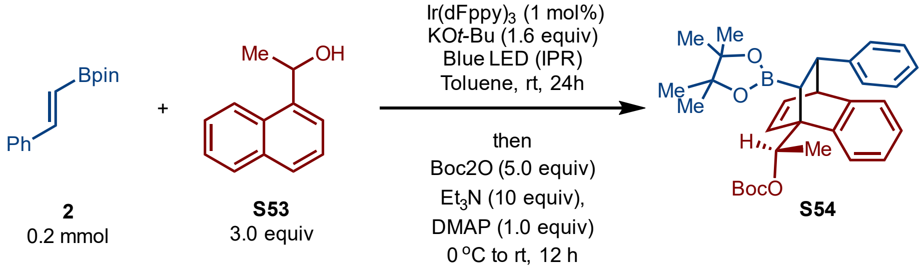

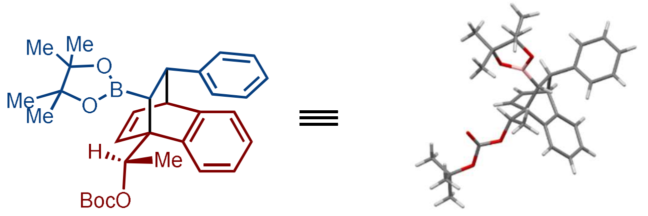

tert-butyl ((S)-1-((1R,4S,9R,10R)-9-phenyl-10-(4,4,5,5-tetramethyl-1,3,2-dioxaborolan-2-yl)-1,4-ethanonaphthalen-1(4H)-yl)ethyl) carbonate (S54): The title compound was prepared according to the following procedure. In a 2 dram vial with a stir bar inside it, Ir(dFppy)3 (1.5 mg, 2.0 μmol, 1.0 mol %), styrenyl-Bpin (46 mg, 0.20 mmol, 1.0 equiv.) was charged, along with the1-(naphthalen-1-yl)ethan-1-ol (103 mg, 0.600 mmol, 3.00 equiv.). then was taken inside the glovebox and KO*t*Bu(36 mg, 0.32 mmol, 1.60 equiv) was added to it and capped and taken outside the glovebox. Then the reaction vial cap was quickly replaced with a rubber septum and the reaction mixture was degassed and filled with N_2 (X 3) followed by addition of the 6 mL of Toluene. Then the rubber septum was quickly replaced by plastic cap and sealed with teflon. The vial was placed under the irradiation of visible light for 24 h in IPR. After that the reaction vial cap was switched to a rubber septum and cooled to 0 °C in an ice-water bath. Triethylamine (280 μL, 2.00 mmol, 10.0 equiv.) was added via syringe under N_2 followed by dropwise addition of Boc_2O (220 mg, 1.00 mmol, 5.00 equiv.) in 1 mL of toluene, and DMAP (24 mg, 0.20 mmol, 1.0 eq), in 0.5 mL of Toluene. The vial was capped and stirred at room temperature for 4 hours. The mixture was transferred to a separatory funnel and washed with H_2O (10 mL). The layers were separated, and aqueous phase was extracted with ethyl acetate (3 x 10 mL). The combined organic layers were washed with brine (15 mL), dried over anhydrous MgSO4, filtered and concentrated by rotary evaporator. Purified by FCC (2% EtOAC in hexanes) to obtain the product as a white solid (30 mg, 0.06 mmol, 30%, >20:1 dr). Stain with Hanessian or PMA. The stereochemistry was determined by single crystal X-Ray.

^{1}H NMR (500 MHz, $CDCl_3$): δ 7.19 (td, *J* = 7.6, 1.4 Hz, 1H), 7.11 – 7.04 (m, 3H), 7.03 (td, *J* = 7.4, 1.1 Hz, 1H), 6.88 (d, *J* = 7.3 Hz, 1H), 6.76 (dd, *J* = 7.8, 6.3 Hz, 1H), 6.54 (s, 2H), 6.41 (d, *J* = 7.5 Hz, 1H), 5.61 (q, *J* = 6.7 Hz, 1H), 3.77 (dt, *J* = 6.2, 1.8

Hz, 1H), 3.24 (dd, J = 6.7, 2.2 Hz, 1H), 1.77 (d, J = 6.7 Hz, 3H), 1.50 (s, 9H), 1.24 (d, J = 11.4 Hz, 12H).

^{13}C NMR (126 MHz, $CDCl_3$): δ 153.8, 144.6, 144.3, 141.8, 137.1, 127.9, 126.2, 126.1, 124.8, 124.4, 122.8, 83.5, 81.7, 76.2, 52.0, 50.5, 49.0, 27.9, 24.8, 24.6, 14.1.

IR (neat): 2978, 1733, 1368, 1279, 1094, 846 cm^{-1}.

HRMS (ESI, m/z): Calculated for $C_{31}H_{39}O_5BNa^+$ $[M+Na]^+$: 525.2783, found: 525.2789.

6. X-Ray data:

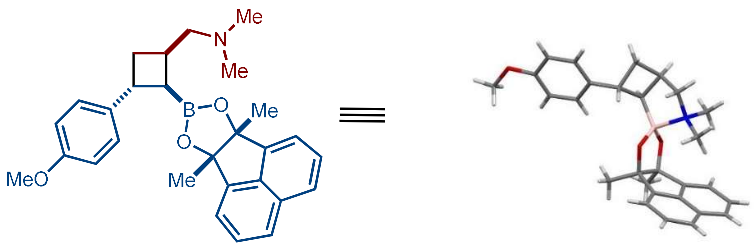

Single crystal suitable for X-Ray diffraction were grown by slow evaporation of a mixture of dichloromethane. A colorless plate crystal (approximate dimensions 0.373 × 0.319 × 0.086 mm^3) was placed onto the tip of a MiTeGen pin and mounted on a Bruker Venture D8 diffractometer equipped with a Photon III detector at 173(2) K.

Data collection

The data collection was carried out using Mo Kα radiation (graphite monochromator) with a frame time of 1 and 15 seconds and a detector distance of 4.0 cm. A collection strategy was calculated and complete data to a resolution of 0.84 Å with a redundancy of 10.24 were collected. Twelve major sections of frames were collected with 1.00º ϕ and ω scans. A total of 2296 frames were collected. The total exposure time was 9.73 hours. The frames were integrated with the Bruker SAINT Software package[37] using a narrow-frame algorithm. The integration of the data using an orthorhombic unit cell yielded a total of 172700 reflections to a maximum θ angle of 26.37° (0.80 Å resolution), of which 3188 were independent (average redundancy 54.172, completeness = 99.9%, Rint = 10.12%, Rsig = 2.79%) and 2820 (88.46%) were greater than 2σ(F2). The final cell constants of a = 7.3388(5) Å, b = 20.0011(13) Å, c = 21.2545(15) Å, volume = 3119.8(4) Å3, are based upon the refinement of the XYZ-centroids of 9901 reflections above 20 σ(I) with 4.500° < 2θ < 54.16°. Data were corrected for absorption effects using the Multi-Scan method (SADABS[38]). The ratio of minimum to maximum apparent transmission was 0.624. The calculated minimum and maximum transmission coefficients (based on crystal size) are 0.9670 and 0.9970. Please refer to Table 1 for additional crystal and refinement information.

Structure solution and refinement

The space group *P*-1 was determined based on intensity statistics and systematic absences. The structure was solved with intrinsic-methods and refined with full-matrix-least squares using the SHELX suite of programs in the Olex2. [39],[40],[41] All non-

hydrogen atoms were refined with anisotropic displacement parameters. The hydrogen atoms were placed in ideal positions and refined as riding atoms with relative isotropic displacement parameters. The final anisotropic full-matrix least-squares refinement on F^2 with 303 variables converged at R1 = 4.68%, for the observed data and wR2 = 13.29% for all data. The goodness-of-fit was 1.089. The largest peak in the final difference electron density synthesis was 0.373 e-/Å^3 and the largest hole was -0.209 e-/Å^3 with an RMS deviation of 0.054 e-/Å^3. On the basis of the final model, the calculated density was 1.242 g/cm^3 and F (000), 472 e$^-$.

Table 1. Crystal data and structure refinement for 23185.

Empirical formula	C28 H32 B N O3	
Formula weight	441.35	
Crystal color, shape, size	colourless plate, 0.373 × 0.319 × 0.086 mm^3	
Temperature	173(3) K	
Wavelength	0.71073 Å	
Crystal system, space group	Triclinic, *P*-1	
Unit cell dimensions	a = 7.903(2) Å	α = 114.490(6)°.
	b = 12.431(3) Å	β = 101.871(8)°.
	c = 13.943(4) Å	γ = 98.123(8)°.
Volume	1179.7(5) $Å^3$	
Z	2	
Density (calculated)	1.242 Mg/m^3	
Absorption coefficient	0.079 mm^{-1}	
F(000)	472	
Data collection		
Diffractometer	Venture D8, Bruker	
Source	Iμ3.0, Incoatec	
Detector	Photon III	
Theta range for data collection	2.723 to 25.024°.	
Index ranges	-9<=h<=9, -14<=k<=14, -16<=l<=16	
Reflections collected	42270	
Independent reflections	4110 [R_{int} = 0.0850]	
Observed Reflections	3710	
Completeness to theta = 25.024°	98.3 %	
Solution and Refinement		
Absorption correction	Multi-Scan	
Max. and min. transmission	0.7452 and 0.4639	
Solution	Intrinsic methods	
Refinement method	Full-matrix least-squares on F^2	
Weighting scheme	$w = [\sigma^2Fo^2 + AP^2 + BP]^{-1}$, with	
	$P = (Fo^2 + 2 Fc^2)/3$, A = 0.0613, B = 0.6043	
Data / restraints / parameters	4110 / 0 / 303	
Goodness-of-fit on F^2	1.089	
Final R indices [I>2σ(I)]	R1 = 0.0468, wR2 = 0.1274	
R indices (all data)	R1 = 0.0510, wR2 = 0.1329	
Extinction coefficient	n/a	
Largest diff. peak and hole	0.373 and -0.209 e.$Å^{-3}$	

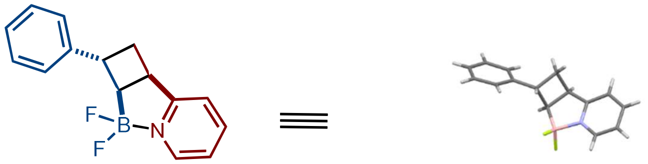

Single crystal suitable for X-Ray diffraction were grown by slow evaporation of a mixture of dichloromethane. A colorless crystal (approximate dimensions 0.168 × 0.103 × 0.034 mm3) was placed onto the tip of a MiTeGen loop and mounted on a Bruker Venture D8 diffractometer equipped with a PhotonIII detector at 173(2) K.

Data collection

The data collection was carried out using Mo Kα radiation (graphite monochromator) with a frame time of 2, 10, 60, and 100 seconds and a detector distance of 4.00 cm. A collection strategy was calculated and complete data to a resolution of 0.84 Å with a redundancy of 13.8 were collected. Six major sections of frames were collected with 1° ω and φ scans. A total of 1314 frames were collected. The total exposure time was 20.99 hours. The frames were integrated with the Bruker SAINT Software package[36] using a narrow-frame algorithm. The integration of the data using a monoclinic unit cell yielded a total of 31740 reflections to a maximum θ angle of 25.05° (0.84 Å resolution), of which 2261 were independent (average redundancy 14.038, completeness = 99.8%, Rint = 4.86%, Rsig= 2.34%) and 1762 (77.93%) were greater than 2σ(F2). The final cell constants of a = 6.6537(3) Å, b = 26.9637(14) Å, c = 7.6078(3) Å, β = 109.3771(17)°, volume = 1287.59(10) Å3, are based upon the refinement of the XYZ-centroids of 7583 reflections above 20 σ(I) with 5.874° < 2θ < 44.37°. Data were corrected for absorption effects using the Multi-Scan method (SADABS[37]). The ratio of minimum to maximum apparent transmission was 0.917. The calculated minimum and maximum transmission coefficients (based on crystal size) are 0.9840 and 0.9970. Please refer to Table 1 for additional crystal and refinement information.

Structure solution and refinement

The space group P21/n was determined based on intensity statistics and systematic absences. The structure was solved and refined using the SHELX suite of programs.[38, 39] An intrinsic-methods solution was calculated, which provided most non-hydrogen atoms from the E-map. Full-matrix least squares / difference Fourier cycles were performed, which located the remaining non-hydrogen atoms. All non-hydrogen atoms were refined with anisotropic displacement parameters. The hydrogen atoms were placed in ideal positions and refined as riding atoms with relative isotropic displacement parameters. Disorder was refined for a phenyl moiety. The final anisotropic full-matrix least-squares refinement on F2 with 188 variables converged at R1 = 6.68%, for the observed data and wR2 = 17.24% for all data. The goodness-of-fit was 1.043. The largest peak in the final difference electron density synthesis was 0.532 e-/Å3 and the largest hole was -0.211 e-/$Å^3$ with an RMS deviation of 0.047 e-/$Å^3$. On the basis of the final model, the calculated density was 1.326 g/cm3 and F (000), 536 e^-.

Table 1. Crystal data and structure refinement for 22197.

Empirical formula	C15 H14 B F2 N	
Formula weight	257.08	
Crystal color, shape, size	colorless plate, 0.168 × 0.103 × 0.034 mm^3	
Temperature	173(2) K	
Wavelength	0.71073 Å	
Crystal system, space group	Monoclinic, $P2_1/n$	
Unit cell dimensions	a = 6.6537(3) Å	α = 90°.
	b = 26.9637(14) Å	β = 109.3771(17)°.
	c = 7.6078(3) Å	γ = 90°.
Volume	1287.59(10) $Å^3$	
Z	4	
Density (calculated)	1.326 Mg/m^3	
Absorption coefficient	0.096 mm^{-1}	
F(000)	536	
Data collection		
Diffractometer	Venture D8, Bruker	
Source	Iμ3.0, Incoatec	
Detector	Photon III	
Theta range for data collection	2.937 to 25.050°.	
Index ranges	-7<=h<=7, -32<=k<=32, -9<=l<=9	
Reflections collected	31740	
Independent reflections	2261 [R_{int} = 0.0486]	
Observed Reflections	1762	
Completeness to theta = 25.051°	99.8 %	
Solution and Refinement		
Absorption correction	Semi-empirical from equivalents	
Max. and min. transmission	0.7452 and 0.6832	
Solution	Intrinsic methods	
Refinement method	Full-matrix least-squares on F^2	
Weighting scheme	$w = [\sigma^2Fo^2 + AP^2 + BP]^{-1}$, with	
	$P = (Fo^2 + 2 Fc^2)/3$, A = 0.0618, B = 1.5060	
Data / restraints / parameters	2261 / 218 / 188	
Goodness-of-fit on F^2	1.043	
Final R indices [I>2σ(I)]	R1 = 0.0668, wR2 = 0.1600	
R indices (all data)	R1 = 0.0845, wR2 = 0.1724	
Largest diff. peak and hole	0.532 and -0.211 e.$Å^{-3}$	

Goodness-of-fit = $[\Sigma[w(F_o^2 - F_c^2)^2]/N_{observns} - N_{params})]^{1/2}$, all data.

$R1 = \Sigma(|F_o| - |F_c|) / \Sigma |F_o|$. $wR2 = [\Sigma[w(F_o^2 - F_c^2)^2] / \Sigma [w(F_o^2)^2]]^{1/2}$.

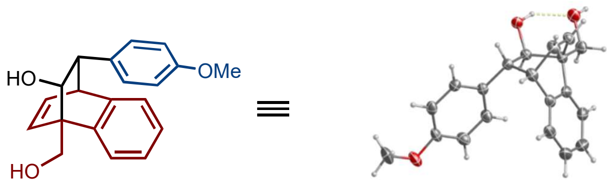

Single crystal suitable for X-Ray diffraction were grown by slow evaporation of a mixture of dichloromethane .A colorless crystal (approximate dimensions 0.184 × 0.177 × 0.038 mm3) was placed onto the tip of a MiTeGen loop and mounted on a Bruker Venture D8 diffractometer equipped with a PhotonIII detector at 153(2) K.

Data collection:

The data collection was carried out using Mo Kα radiation (graphite monochromator) with a frame time of 1 seconds and a detector distance of 4.00 cm. A collection strategy was calculated and complete data to a resolution of 0.77 Å with a redundancy of 13.5 were collected. Six major sections of frames were collected with 1° ω and φ scans. A total of 1816 frames were collected. The total exposure time was 5.30 hours. The frames were integrated with the Bruker SAINT Software package[36] using a narrow-frame algorithm. The integration of the data using a monoclinic unit cell yielded a total of 168820 reflections to a maximum θ angle of 26.42° (0.80 Å resolution), of which 12955 were independent (average redundancy 13.031, completeness = 99.7%, Rint = 8.93%, Rsig = 3.88%) and 8483 (65.48%) were greater than 2σ(F2). The final cell constants of a = 21.1997(6) Å, b = 10.1488(3) Å, c = 30.8598(10) Å, β = 108.0530(10) °, volume = 6312.7(3) $Å^3$, are based upon the refinement of the XYZ-centroids of 8994 reflections above 20 σ(I) with 4.514° < 2θ < 52.09°. Data were corrected for absorption effects using the MultiScan method (SADABS[37]). The ratio of minimum to maximum apparent transmission was 0.948. The calculated minimum and maximum transmission coefficients (based on crystal size) are 0.9840 and 0.9970. Please refer to Table 1 for additional crystal and refinement information.

Structure solution and refinement:

The space group P21/n was determined based on intensity statistics and systematic absences. The structure was solved and refined using the SHELX suite of programs.[38, 39] An intrinsic-methods solution was calculated, which provided most non-hydrogen atoms from the E-map. Full-matrix least squares / difference Fourier cycles were performed, which located the remaining non-hydrogen atoms. All non-hydrogen atoms were refined with anisotropic displacement parameters. The hydrogen atoms were placed in ideal positions and refined as riding atoms with relative isotropic displacement parameters. Four crystallographically independent molecules are in the asymmetric unit. Disorder was refined for molecule C and D. The final anisotropic full-matrix least-squares refinement on F2 with 867 variables converged at R1 = 4.90%, for the observed data and wR2 = 13.02% for all data. The goodness-of-fit was 0.990. The largest peak in the final difference electron density synthesis was 0.347 e- /$Å^3$ and the largest hole was - 0.363 e- /Å3 with an RMS deviation of 0.042 e- /$Å^3$. On the basis

of the final model, the calculated density was 1.298 g/cm3 and F (000), 2624 e- . Hydrogen bonding was found; details are listed in the tables.

Table 1. Crystal data and structure refinement for 22066.

Empirical formula	C20 H20 O3	
Formula weight	308.36	
Crystal color, shape, size	colorless plate, 0.184 × 0.177 × 0.038 mm^3	
Temperature	153(2) K	
Wavelength	0.71073 Å	
Crystal system, space group	Monoclinic, $P2_1/n$	
Unit cell dimensions	a = 21.1997(6) Å	α = 90°.
	b = 10.1488(3) Å	β = 108.0530(10)°.
	c = 30.8598(10) Å	γ = 90°.
Volume	6312.7(3) $Å^3$	
Z	16	
Density (calculated)	1.298 Mg/m^3	
Absorption coefficient	0.086 mm^{-1}	
F(000)	2624	
Data collection		
Diffractometer	Venture D8, Bruker	
Source	IμS 3.0, Incoatec	
Detector	Photon III	
Theta range for data collection	2.013 to 26.419°.	
Index ranges	-24<=h<=26, -12<=k<=12, -38<=l<=38	
Reflections collected	168820	
Independent reflections	12955 [R_{int} = 0.0893]	
Observed Reflections	8483	
Completeness to theta = 25.242°	100.0 %	

Solution and Refinement

Absorption correction	Semi-empirical from equivalents
Max. and min. transmission	0.7454 and 0.7068
Solution	Intrinsic methods
Refinement method	Full-matrix least-squares on F^2
Weighting scheme	$w = [\sigma^2Fo^2 + AP^2 + BP]^{-1}$, with
	$P = (Fo^2 + 2 Fc^2)/3$, A = 0.0484, B = 3.8221
Data / restraints / parameters	12955 / 1456 / 867
Goodness-of-fit on F^2	0.990
Final R indices [I>2σ(I)]	R1 = 0.0490, wR2 = 0.1093
R indices (all data)	R1 = 0.0876, wR2 = 0.1302
Largest diff. peak and hole	0.347 and -0.363 e.$Å^{-3}$

Goodness-of-fit = $[\Sigma[w(F_o^2 - F_c^2)^2]/N_{observns} - N_{params})]^{1/2}$, all data.

R1 = $\Sigma(|F_o| - |F_c|) / \Sigma |F_o|$. $wR2 = [\Sigma[w(F_o^2 - F_c^2)^2] / \Sigma [w(F_o^2)^2]]^{1/2}$.

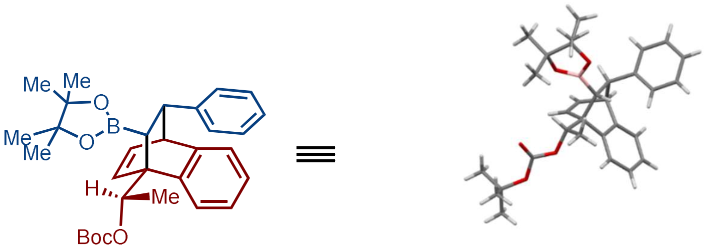

Single crystal suitable for X-Ray diffraction were grown by slow evaporation of a mixture of dichloromethane and pentane. The sample was submitted by Souvik Adak (research group of Kevin Brown, Department of Chemistry, Indiana University). A colorless block crystal (approximate dimensions 0.265 × 0.239 × 0.203 mm^3) was placed onto the tip of a MiTeGen pin and mounted on a Bruker Venture D8 diffractometer equipped with a Photon III detector at 213(2) K. Data collection The data collection was carried out using Mo Kα radiation (graphite monochromator) with a frame time of 1, 5, and 60 seconds and a detector distance of 5.0 cm. A collection strategy was calculated and complete data to a resolution of 0.84 Å with a redundancy of 11.75 were collected. Sixteen major sections of frames were collected with 1.00º ϕ and ω scans. A total of 2475 frames were collected. The total exposure time was 22.16 hours. The frames were integrated with the Bruker SAINT Software package[36] using a narrow-frame algorithm. The integration of the data using a triclinic unit cell yielded a total of 122079 reflections to a maximum θ angle of 25.03° (0.84 Å resolution), of which 10373 were independent (average redundancy 11.769, completeness = 99.8%, Rint = 7.36%, Rsig = 3.72%) and 8793 (84.77%) were greater than 2σ(F2). The final cell constants of a = 13.654(3) Å, b = 15.017(4) Å, c = 15.842(4) Å, α = 85.001(8)°, β = 75.075(7)°, γ = 69.697(8)°, volume = 2943.7(13) Å3 , are based upon the refinement of the XYZ-centroids of 8712 reflections above 20 σ(I) with 5.321° < 2θ < 50.02°. Data were corrected for absorption effects using the Multi-Scan method (SADABS[37]). The ratio of minimum to maximum apparent transmission was 0.710. The calculated minimum and maximum transmission coefficients (based on crystal size) are 0.4446 and 0.6258.

Structure solution and refinement

The space group P-1 was determined based on intensity statistics and systematic absences. The structure was solved with intrinsic-methods and refined with full-matrix-least squares using the SHELX suite of programs in the Olex2.[38, 39, 40] All non-hydrogen atoms were refined with anisotropic displacement parameters. The hydrogen atoms were placed in ideal positions and refined as riding atoms with relative isotropic displacement parameters. Disorder was found in the structure. Site occupancies and constraints/restraints were applied. The final anisotropic full-matrix least-squares refinement on F2 with 836 variables converged at R1 = 5.20%, for the observed data and wR2 = 14.64% for all data. The goodness-of-fit was 1.046. The largest peak in the final difference electron density synthesis was 0.309 e- /Å^3 and the largest hole was -0.265 e- /Å^3 with an RMS deviation of 0.039 e- /Å^3. On the basis of

the final model, the calculated density was 1.134 g/cm^3 and F (000), 1080 e$^-$.

Table 1. Crystal data and structure refinement for 24023.

Empirical formula	C31 H39 B O5	
Formula weight	502.43	
Crystal color, shape, size	colourless block, 0.265 × 0.239 × 0.203 mm^3	
Temperature	213(2) K	
Wavelength	0.71073 Å	
Crystal system, space group	Triclinic, *P*-1	
Unit cell dimensions	a = 13.654(3) Å	α = 85.001(8)°.
	b = 15.017(4) Å	β = 75.075(7)°.
	c = 15.842(4) Å	γ = 69.697(8)°.
Volume	2943.8(13) Å^3	
Z	4	
Density (calculated)	1.134 Mg/m^3	
Absorption coefficient	0.075 mm^{-1}	
F(000)	1080	
Data collection		
Diffractometer	Venture D8, Bruker	
Source	Iμ3.0, Incoatec	
Detector	Photon III	
Theta range for data collection	1.962 to 25.027°.	
Index ranges	-16<=h<=16, -17<=k<=17, -18<=l<=18	
Reflections collected	122079	
Independent reflections	10373 [R_{int} = 0.0736]	
Observed Reflections	8793	
Completeness to theta = 25.027°	99.8 %	
Solution and Refinement		
Absorption correction	Multi-Scan	
Max. and min. transmission	0.6258 and 0.4446	
Solution	Intrinsic methods	
Refinement method	Full-matrix least-squares on F^2	
Weighting scheme	w = [σ^2Fo^2+ AP^2+ BP]$^{-1}$, with	
	P = (Fo^2+ 2 Fc^2)/3, A = 0.0678, B = 1.0695	
Data / restraints / parameters	10373 / 1172 / 836	
Goodness-of-fit on F^2	1.046	
Final R indices [I>2σ(I)]	R1 = 0.0520, wR2 = 0.1361	
R indices (all data)	R1 = 0.0606, wR2 = 0.1464	
Extinction coefficient	n/a	
Largest diff. peak and hole	0.309 and -0.265 e.Å^{-3}	

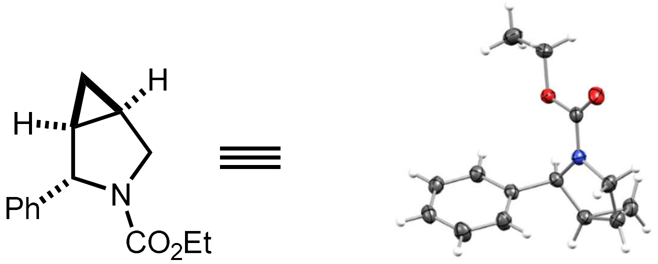

Single crystal suitable for X-Ray diffraction were grown by slow evaporation of hexanes. . A colorless plate crystal (approximate dimensions 0.486 × 0.150 × 0.061 mm3) was placed onto the tip of a MiTeGen pin and mounted on a Bruker Venture D8 diffractometer equipped with a Photon III detector at 173(2) K.

Data collection

The data collection was carried out using Mo Kα radiation (graphite monochromator) with a frame time of 2 and 15 seconds and a detector distance of 5.0 cm. A collection strategy was calculated and complete data to a resolution of 0.84 Å with a redundancy of 8.92 were collected. Five major sections of frames were collected with 1.00º ϕ and ω scans. A total of 1086 frames were collected. The total exposure time was 2.78 hours. The frames were integrated with the Bruker SAINT Software package using a narrow-frame algorithm. The integration of the data using a monoclinic unit cell yielded a total of 19430 reflections to a maximum θ angle of 25.09° (0.84 Å resolution), of which 2115 were independent (average redundancy 9.187, completeness = 99.8%, Rint = 6.93%, Rsig = 3.77%) and 1802 (85.20%) were greater than 2σ (F2). The final cell constants of a = 9.4874(7) Å, b = 9.1393(7) Å, c = 13.7153(11) Å, β = 90.563(3)°, volume = 1189.17(16) Å3, are based upon the refinement of the XYZ-centroids of 7954 reflections above 20 σ(I) with 5.245° < 2θ < 50.01°. Data were corrected for absorption effects using the Multi-Scan method (SADABS). The ratio of minimum to maximum apparent transmission was 0.631. The calculated minimum and maximum transmission coefficients (based on crystal size) are 0.9590 and 0.9950.

Structure solution and refinement

The space group *P*21/*n* was determined based on intensity statistics and systematic absences. The structure was solved with intrinsic-methods and refined with full-matrix-least squares using the SHELX suite of programs in the Olex2. [38, 39, 40] All non-hydrogen atoms were refined with anisotropic displacement parameters. The hydrogen atoms were placed in ideal positions and refined as riding atoms with relative isotropic displacement parameters. The final anisotropic full-matrix least-squares refinement on F2 with 155 variables converged at R1 = 4.23%, for the observed data and wR2 = 11.68% for all data. The goodness-of-fit was 1.070. The largest peak in the final difference electron density synthesis was 0.215 e-/Å3 and the largest hole was -0.256 e-/Å3 with an RMS deviation of 0.047 e-/Å3. On the basis of the final model, the calculated density was 1.292 g/cm3 and F(000), 496 e-.

Table 1. Crystal data and structure refinement for 24183.

Empirical formula	C14 H17 N O2	
Formula weight	231.28	
Crystal color, shape, size	colourless plate, 0.486 × 0.15 × 0.061 mm^3	
Temperature	173(2) K	
Wavelength	0.71073 Å	
Crystal system, space group	Monoclinic, $P2_1/n$	
Unit cell dimensions	a = 9.4874(7) Å	α = 90°.
	b = 9.1393(7) Å	β = 90.563(3)°.
	c = 13.7153(11) Å	γ = 90°.
Volume	1189.17(16) $Å^3$	
Z	4	
Density (calculated)	1.292 Mg/m^3	
Absorption coefficient	0.086 mm^{-1}	
F(000)	496	
Data collection		
Diffractometer	Venture D8, Bruker	
Source	Iμ3.0, Incoatec	
Detector	Photon III	
Theta range for data collection	2.599 to 25.088°.	
Index ranges	-11<=h<=11, -10<=k<=10, -16<=l<=16	
Reflections collected	19430	
Independent reflections	2115 [R_{int} = 0.0693]	
Observed Reflections	1802	
Completeness to theta = 25.088°	99.8 %	
Solution and Refinement		
Absorption correction	Multi-Scan	
Max. and min. transmission	0.7452 and 0.4700	
Solution	Intrinsic methods	
Refinement method	Full-matrix least-squares on F^2	
Weighting scheme	w = $[\sigma^2Fo^2 + AP^2 + BP]^{-1}$, with	
	P = $(Fo^2 + 2 Fc^2)/3$, A = 0.0539P, B = 0.5098	
Data / restraints / parameters	2115 / 0 / 155	
Goodness-of-fit on F^2	1.070	
Final R indices [I>2σ(I)]	R1 = 0.0423, wR2 = 0.1071	
R indices (all data)	R1 = 0.0508, wR2 = 0.1168	
Extinction coefficient	n/a	
Largest diff. peak and hole	0.215 and -0.256 e.$Å^{-3}$	

7. Spectra:

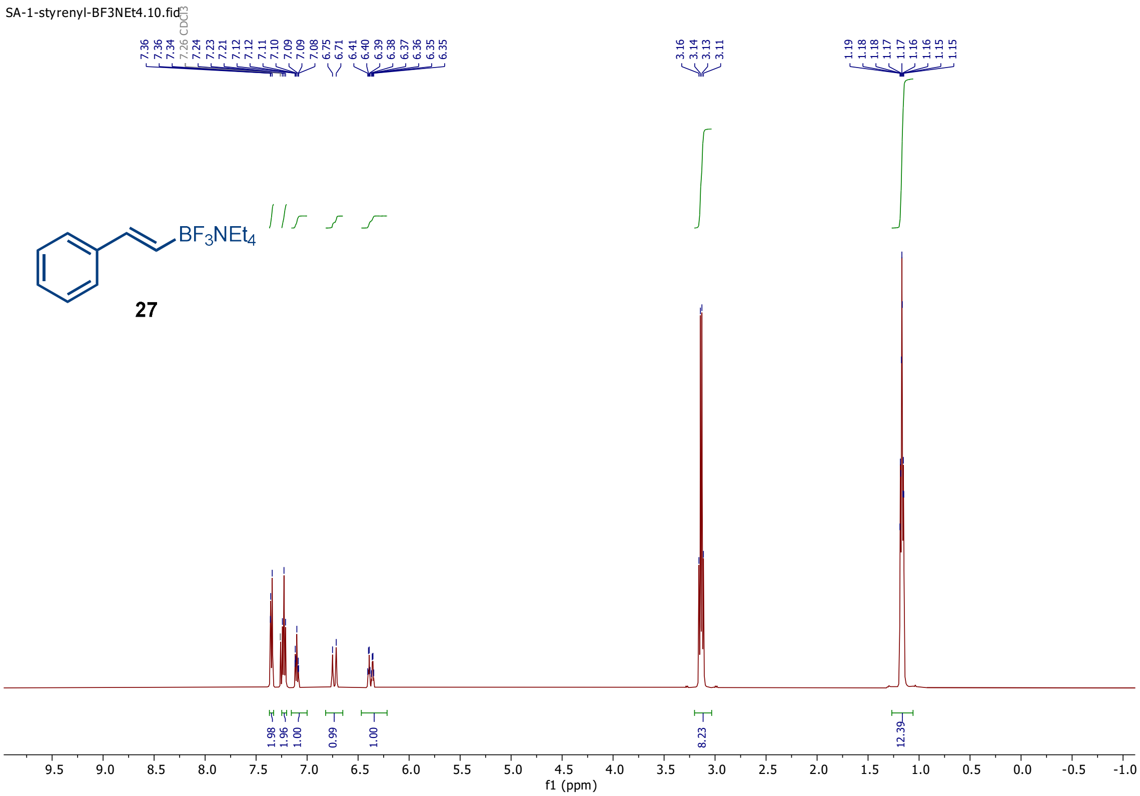

27

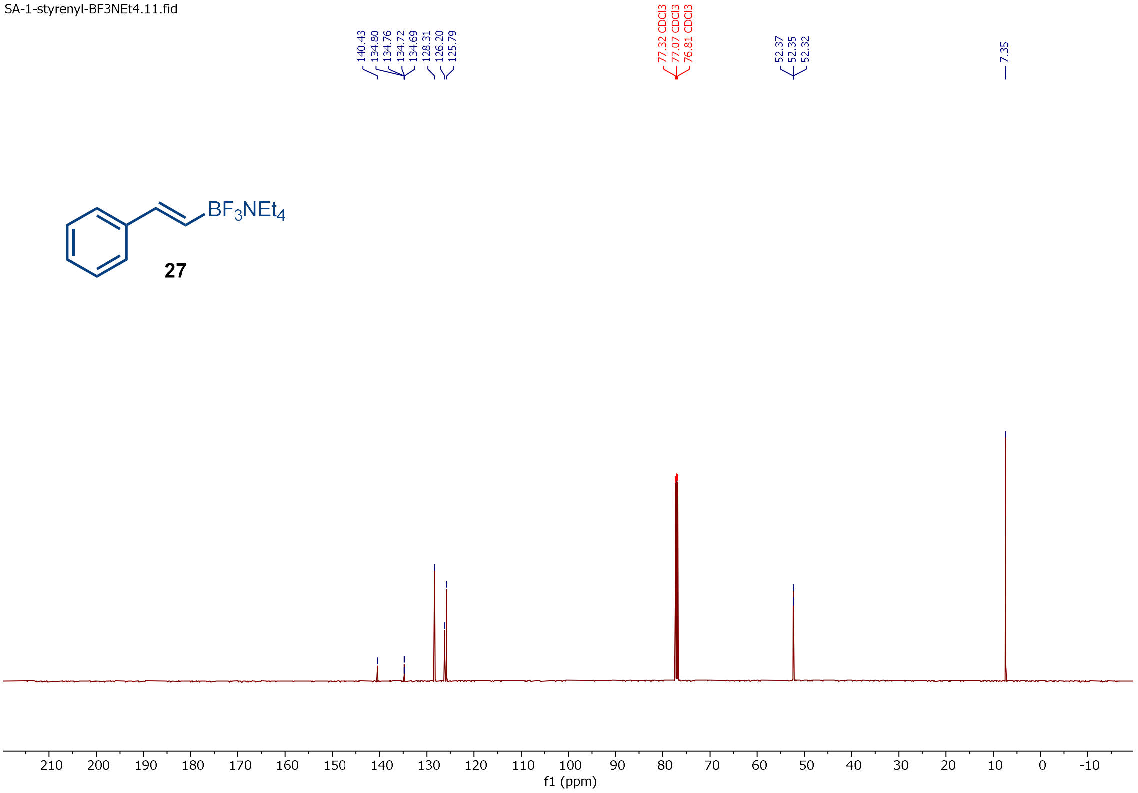

27

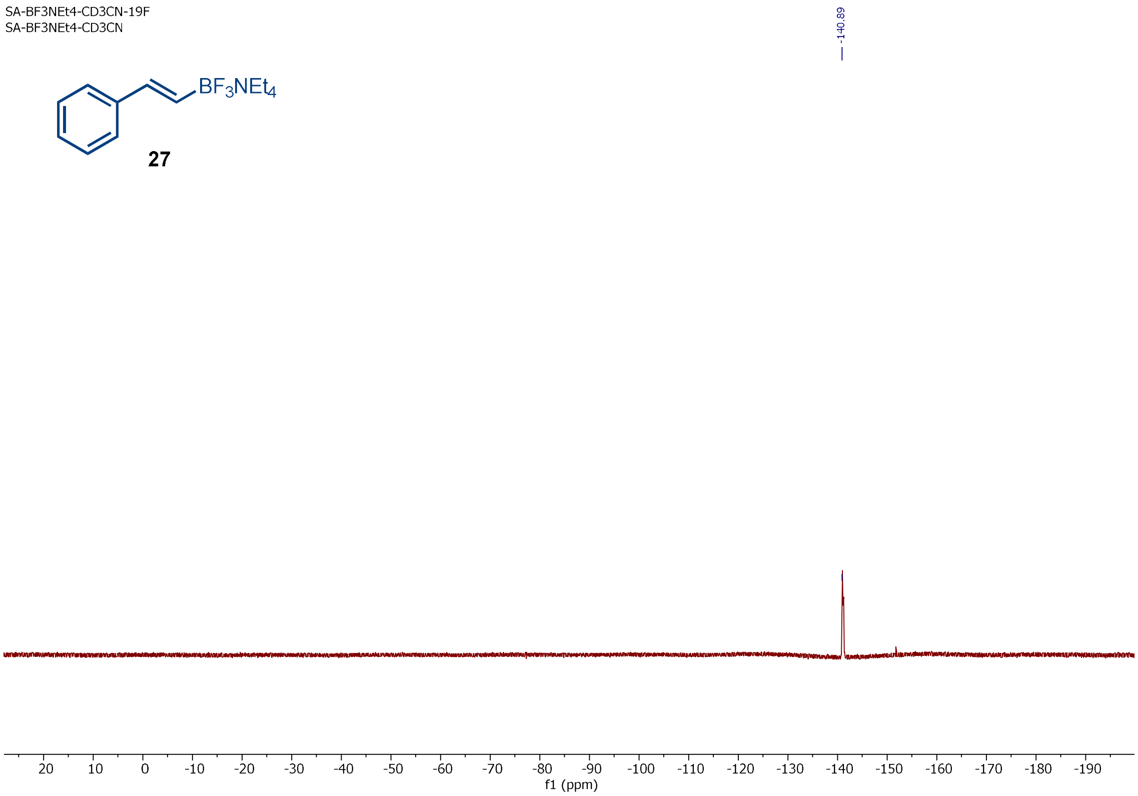
SA-BF3NEt4-CD3CN-19F
SA-BF3NEt4-CD3CN
-140.89
BF3NEt4
27
f1 (ppm)

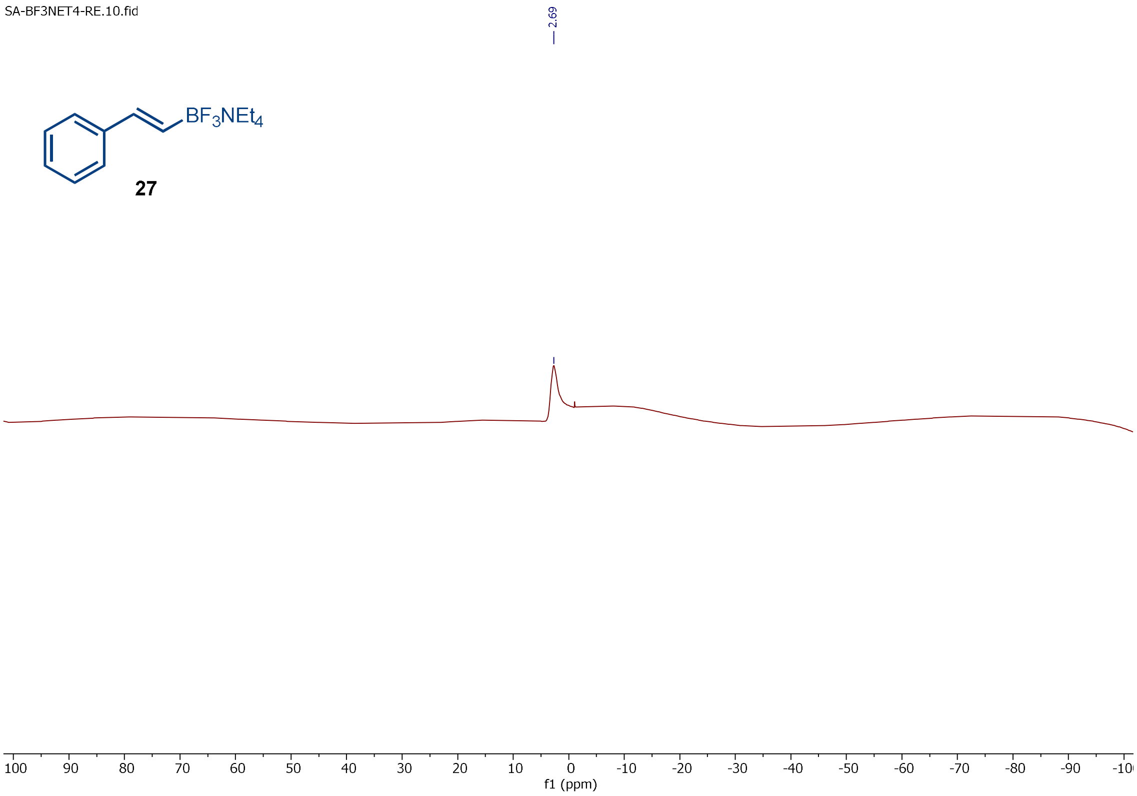
SA-BF3NET4-RE.10.fid
2.69
BF3NEt4
27
f1 (ppm)

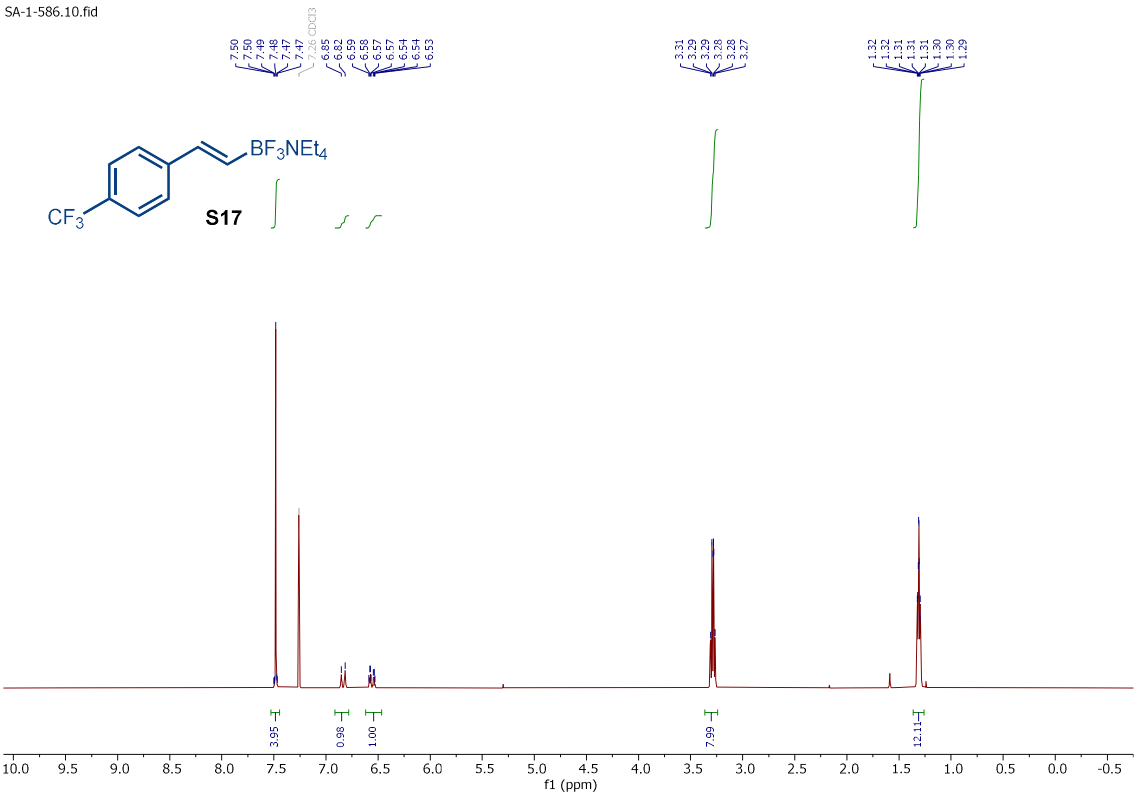
SA-1-586.10.fid
7.50
7.50
7.49
7.48
7.47
7.26 CDCl3
6.85
6.82
6.59
6.58
6.57
6.57
6.54
6.54
6.53
3.31
3.29
3.29
3.28
3.28
3.27
1.32
1.32
1.31
1.31
1.31
1.30
1.30
1.29
BF3NEt4
CF3
S17
3.95
0.98
1.00
7.99
12.11
f1 (ppm)

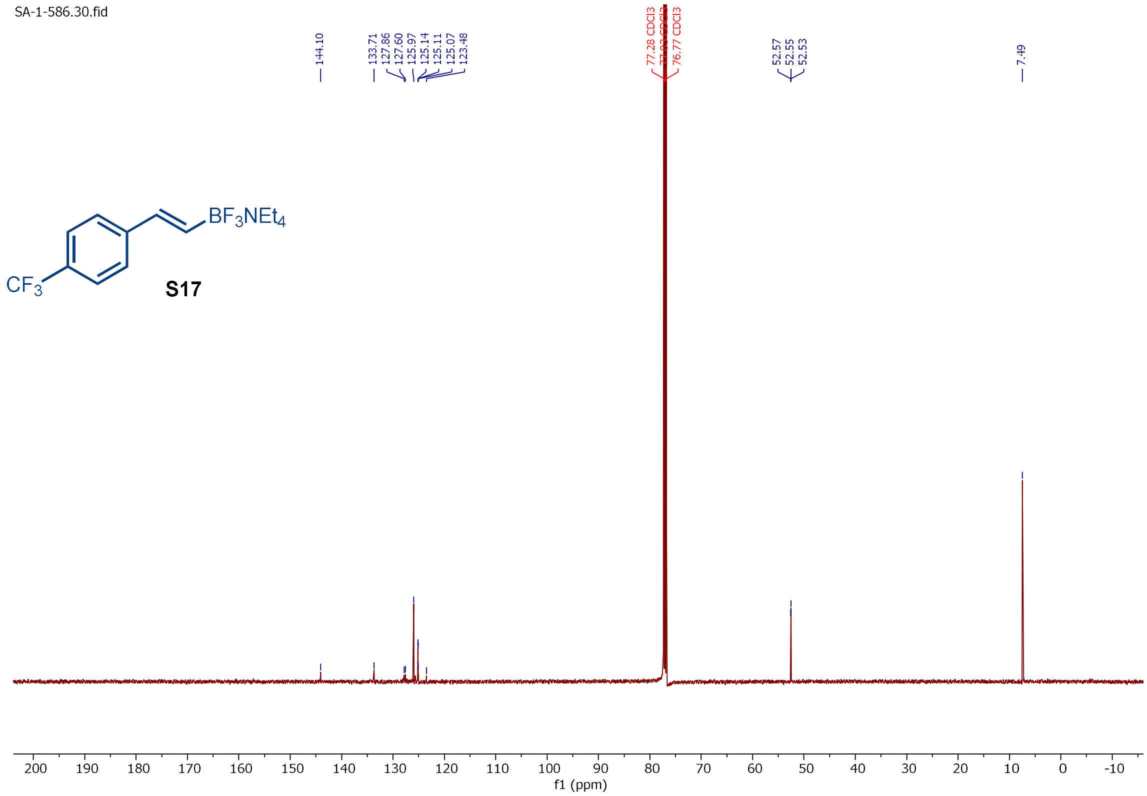
SA-1-586.30.fid
144.10
133.71
127.86
127.60
125.97
125.14
125.11
125.07
123.48
77.28 CDCl3
77.02 CDCl3
76.77 CDCl3
52.57
52.55
52.53
7.49
BF3NEt4
CF3
S17
f1 (ppm)

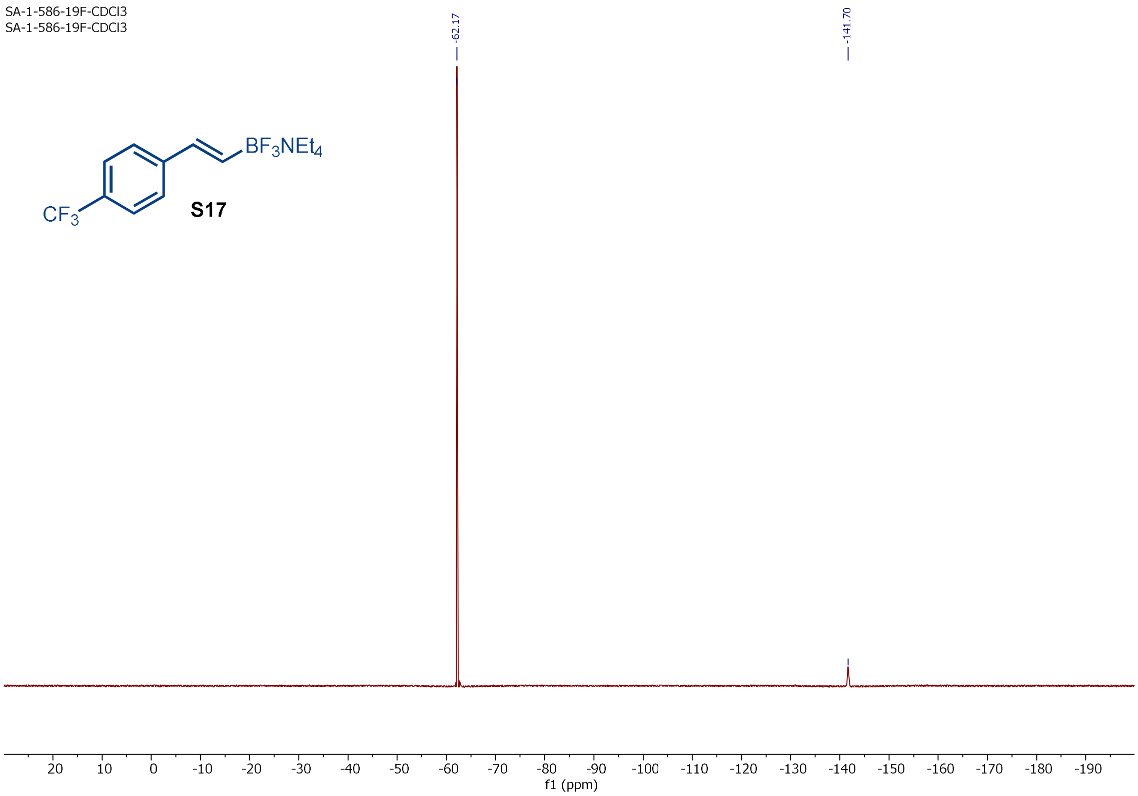
SA-1-586-19F-CDCl3
SA-1-586-19F-CDCl3
-62.17
-141.70
BF3NEt4
CF3
S17
20 10 0 -10 -20 -30 -40 -50 -60 -70 -80 -90 -100 -110 -120 -130 -140 -150 -160 -170 -180 -190
f1 (ppm)

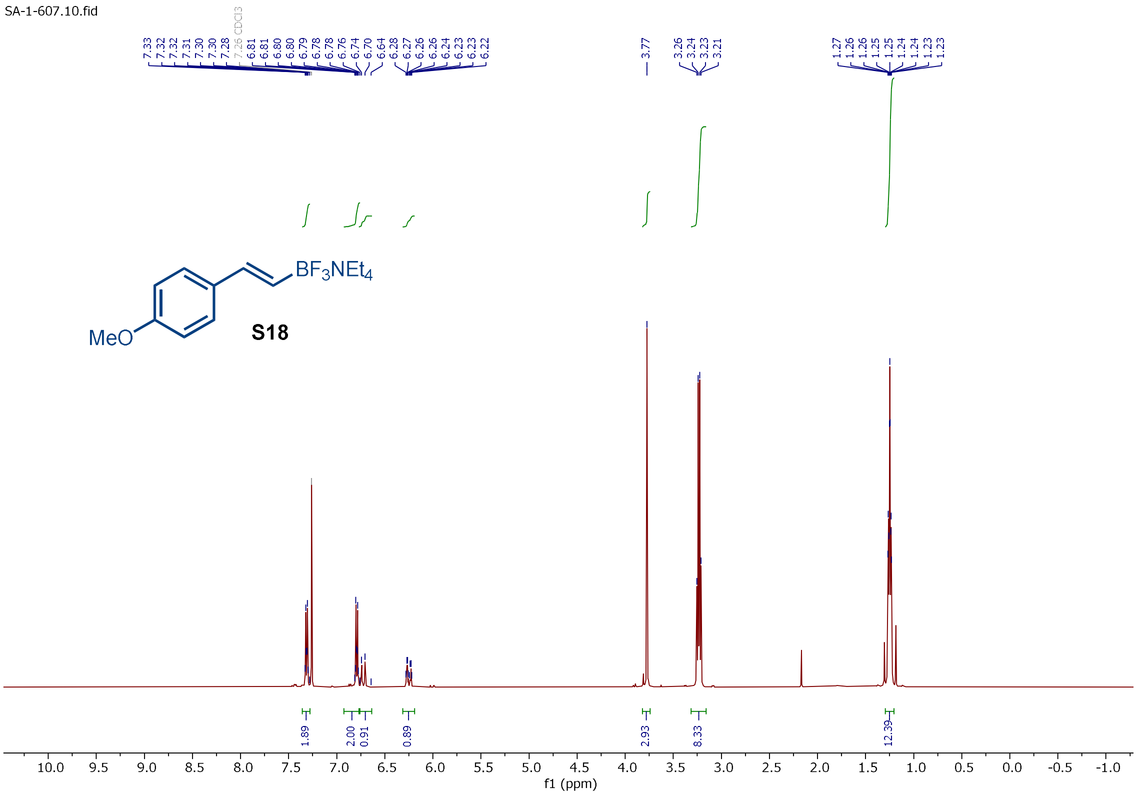
SA-1-607.10.fid
7.33 7.32 7.32 7.31 7.30 7.28 7.26 CDCl3 6.81 6.81 6.80 6.80 6.79 6.78 6.78 6.76 6.74 6.70 6.64 6.28 6.27 6.26 6.26 6.24 6.24 6.23 6.23 6.22
3.77
3.26 3.24 3.23 3.21
1.27 1.26 1.26 1.25 1.25 1.24 1.24 1.23 1.23
BF3NEt4
MeO
S18
1.89
2.00
0.91
0.89
2.93
8.33
12.39
10.0 9.5 9.0 8.5 8.0 7.5 7.0 6.5 6.0 5.5 5.0 4.5 4.0 3.5 3.0 2.5 2.0 1.5 1.0 0.5 0.0 -0.5 -1.0
f1 (ppm)

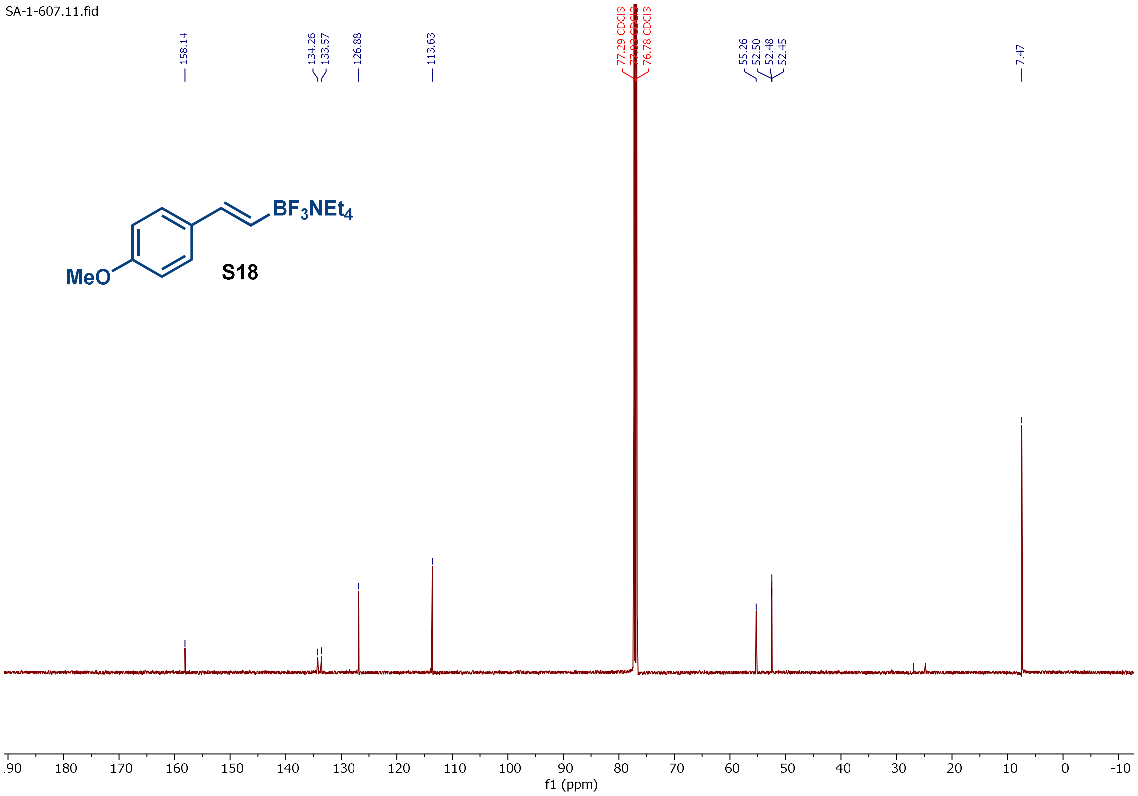
SA-1-607.11.fid
158.14
134.26
133.57
126.88
113.63
77.29 CDCl3
76.78 CDCl3
55.26
52.50
52.48
52.45
7.47
BF3NEt4
MeO
S18
f1 (ppm)

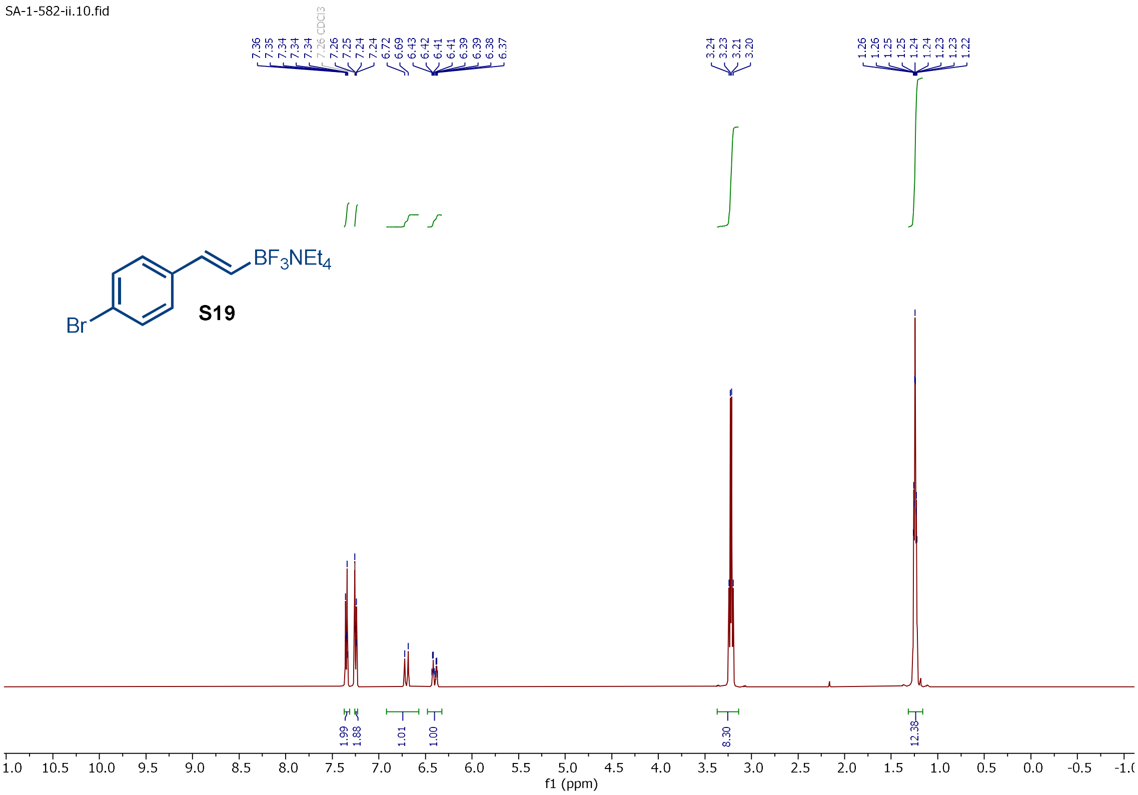
SA-1-582-ii.10.fid
7.36
7.35
7.34
7.34
7.34
7.26 CDCl3
7.26
7.25
7.24
7.24
6.72
6.69
6.43
6.42
6.41
6.41
6.39
6.39
6.38
6.37
3.24
3.23
3.21
3.20
1.26
1.26
1.25
1.25
1.24
1.24
1.23
1.23
1.22
BF3NEt4
Br
S19
1.99
1.88
1.01
1.00
8.30
12.38
f1 (ppm)

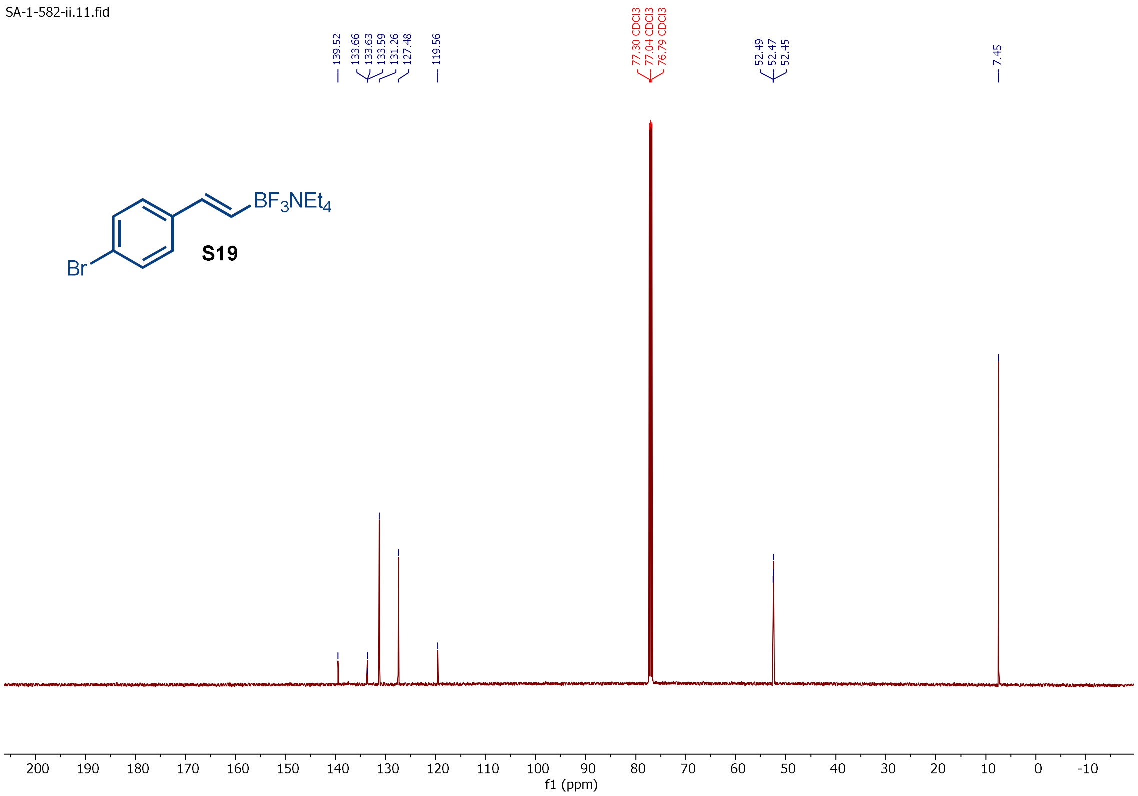

SA-1-582-ii.11.fid
139.52
133.66
133.63
133.59
131.26
127.48
119.56
77.30 CDCl3
77.04 CDCl3
76.79 CDCl3
52.49
52.47
52.45
7.45
BF3NEt4
Br
S19
f1 (ppm)

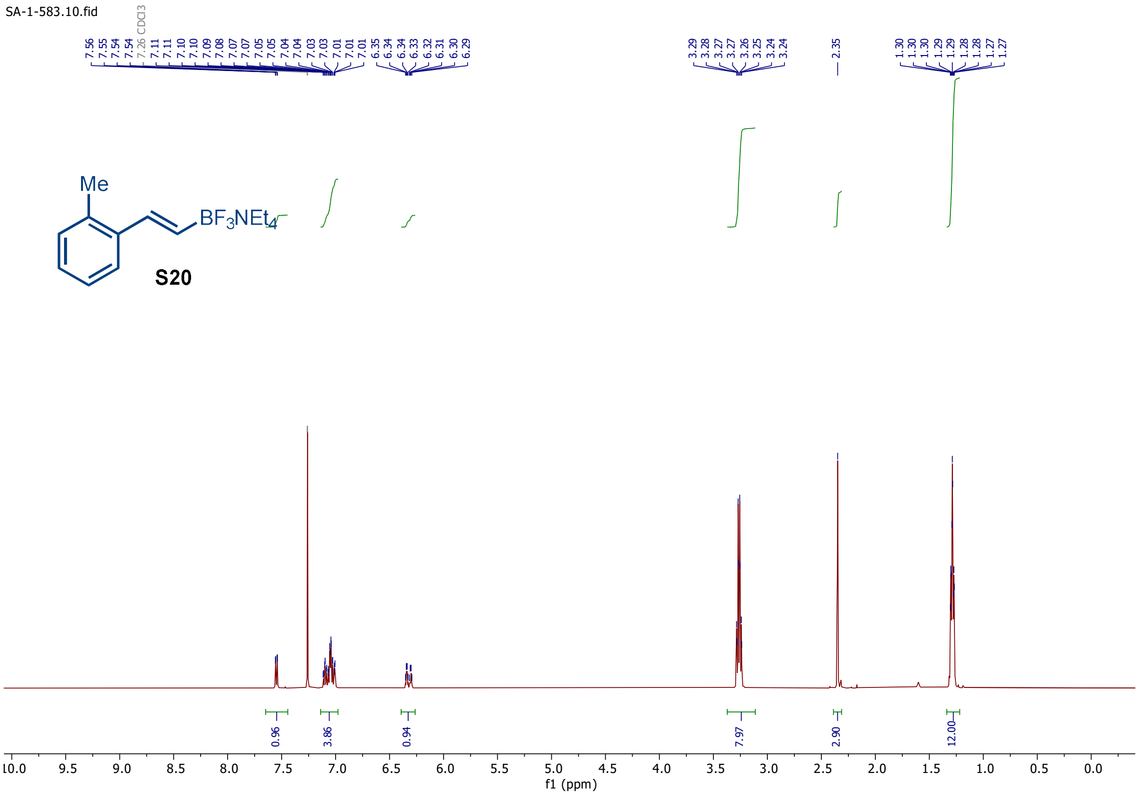

SA-1-583.10.fid
7.26 CDCl3
Me
BF3NEt4
S20
0.96
3.86
0.94
7.97
2.90
12.00
f1 (ppm)

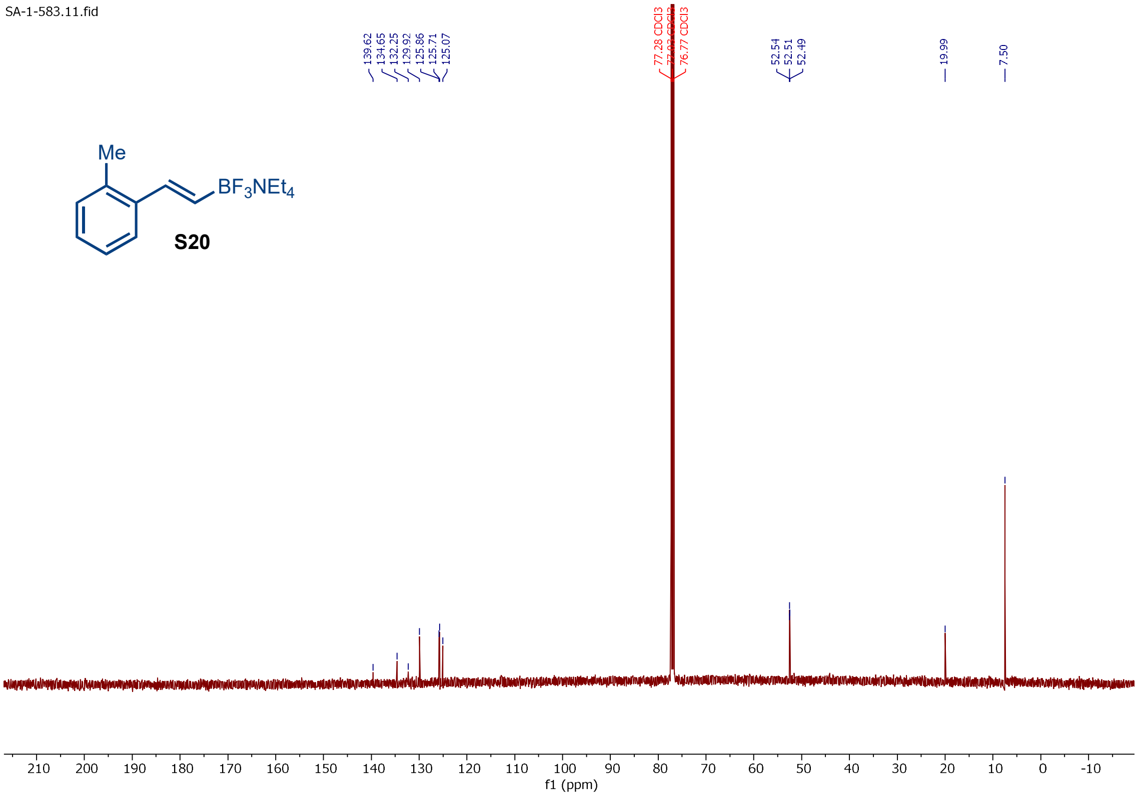
SA-1-583.11.fid
139.62
134.65
132.25
129.92
125.86
125.71
125.07
77.28 CDCl3
76.77 CDCl3
52.54
52.51
52.49
19.99
7.50
Me
BF3NEt4
S20
f1 (ppm)

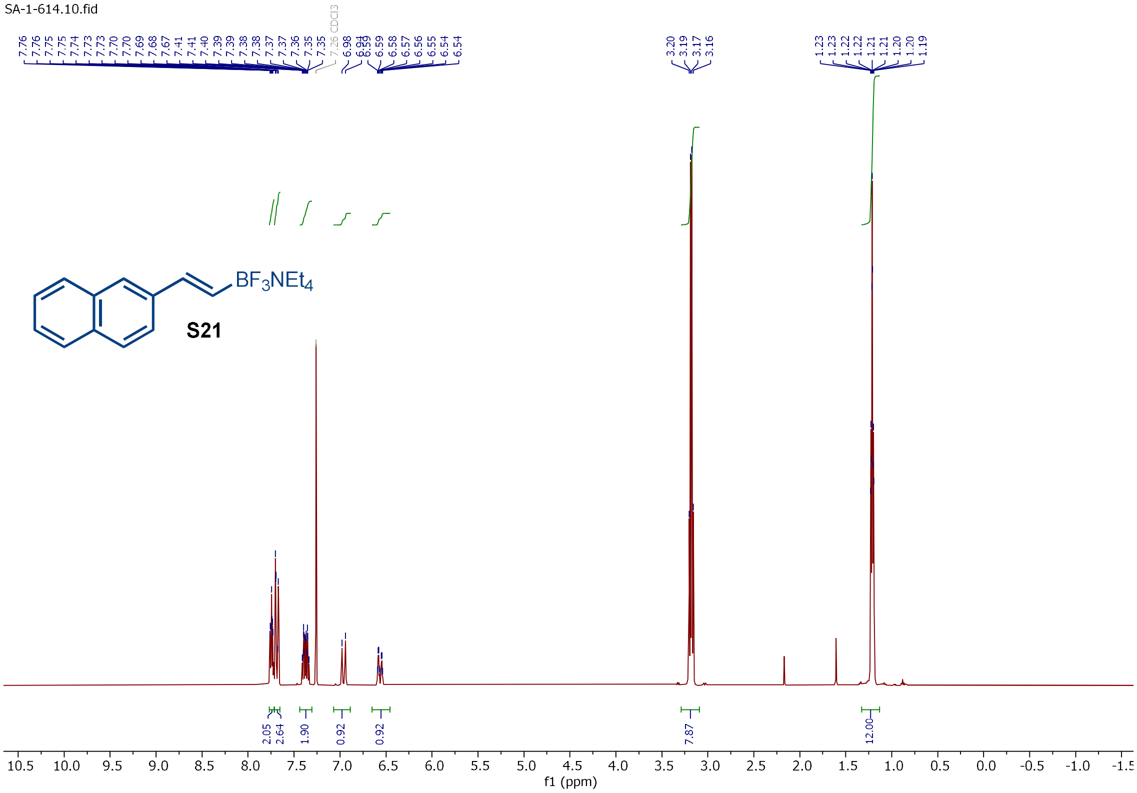
SA-1-614.10.fid
7.76
7.76
7.75
7.74
7.73
7.73
7.70
7.69
7.68
7.67
7.41
7.40
7.39
7.39
7.38
7.38
7.37
7.37
7.36
7.35
7.26 CDCl3
6.98
6.94
6.59
6.59
6.58
6.57
6.56
6.55
6.54
6.54
3.20
3.19
3.17
3.16
1.23
1.23
1.22
1.22
1.21
1.21
1.20
1.20
1.19
BF3NEt4
S21
2.05
2.64
1.90
0.92
0.92
7.87
12.00
f1 (ppm)

SA-1-614.11.fid

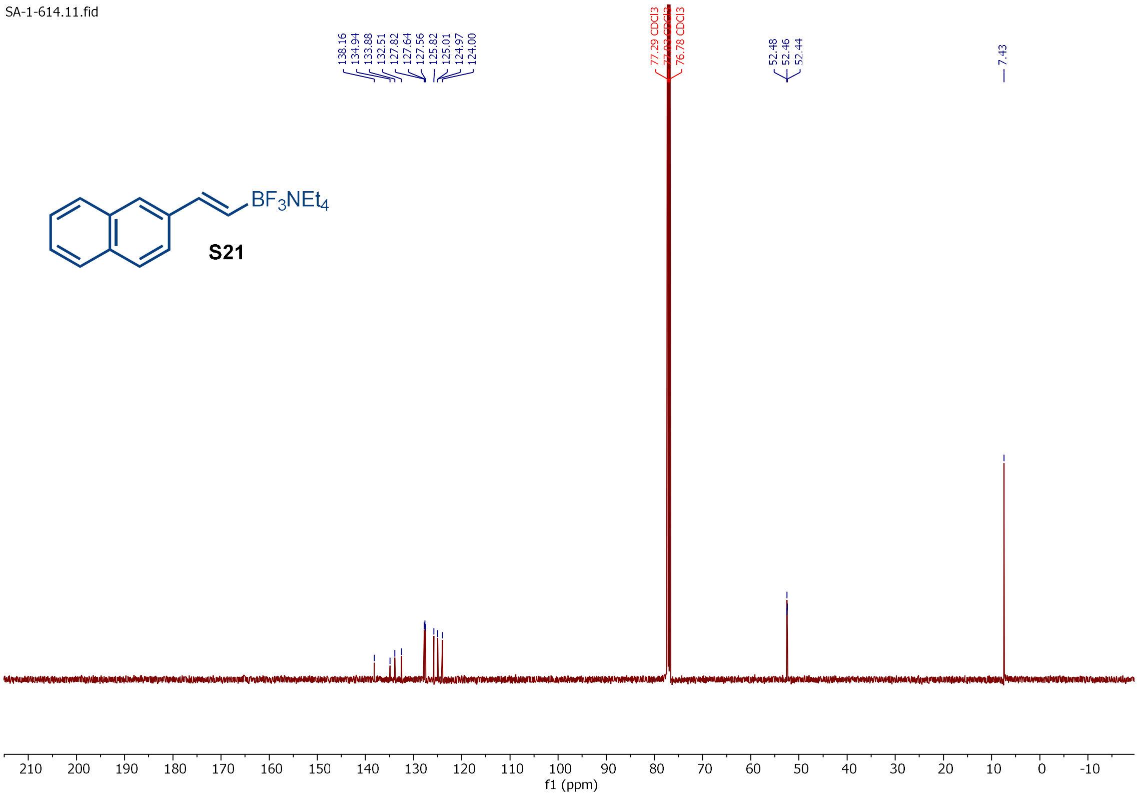

SA-1-615.10.fid

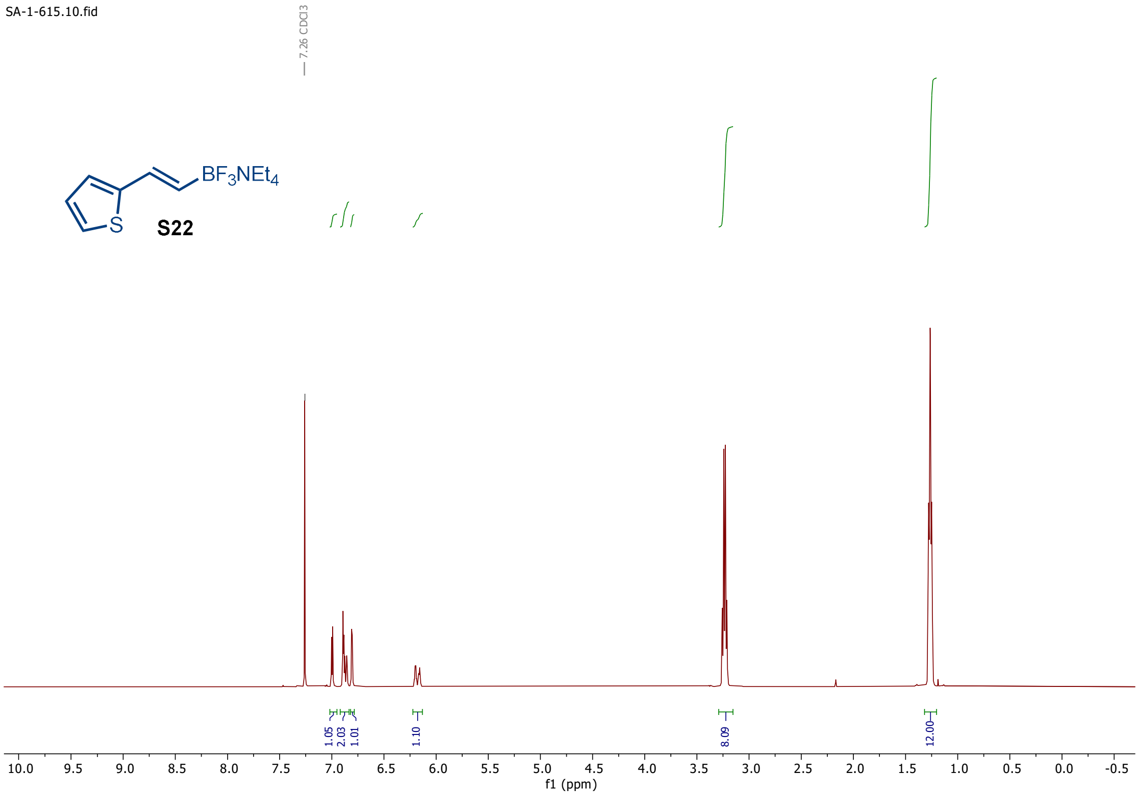

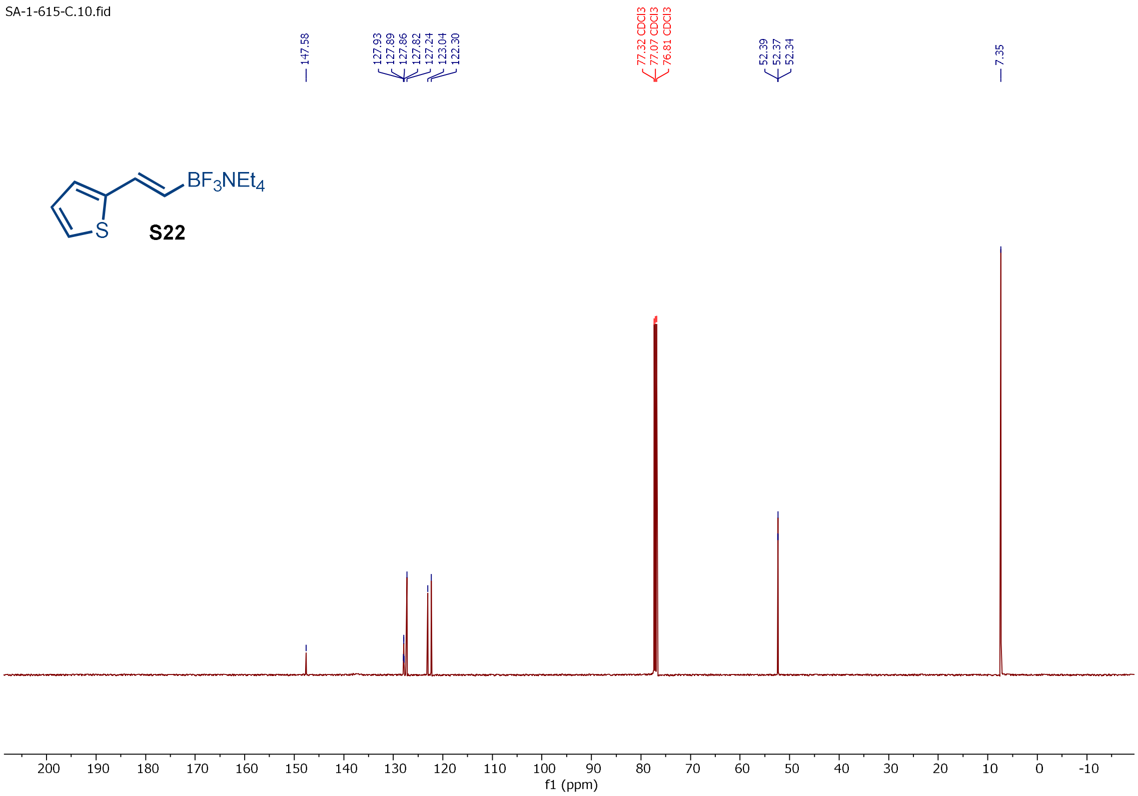
SA-1-615-C.10.fid
147.58
127.93
127.89
127.86
127.82
127.24
123.04
122.30
77.32 CDCl3
77.07 CDCl3
76.81 CDCl3
52.39
52.37
52.34
7.35
BF3NEt4
S
S22
f1 (ppm)

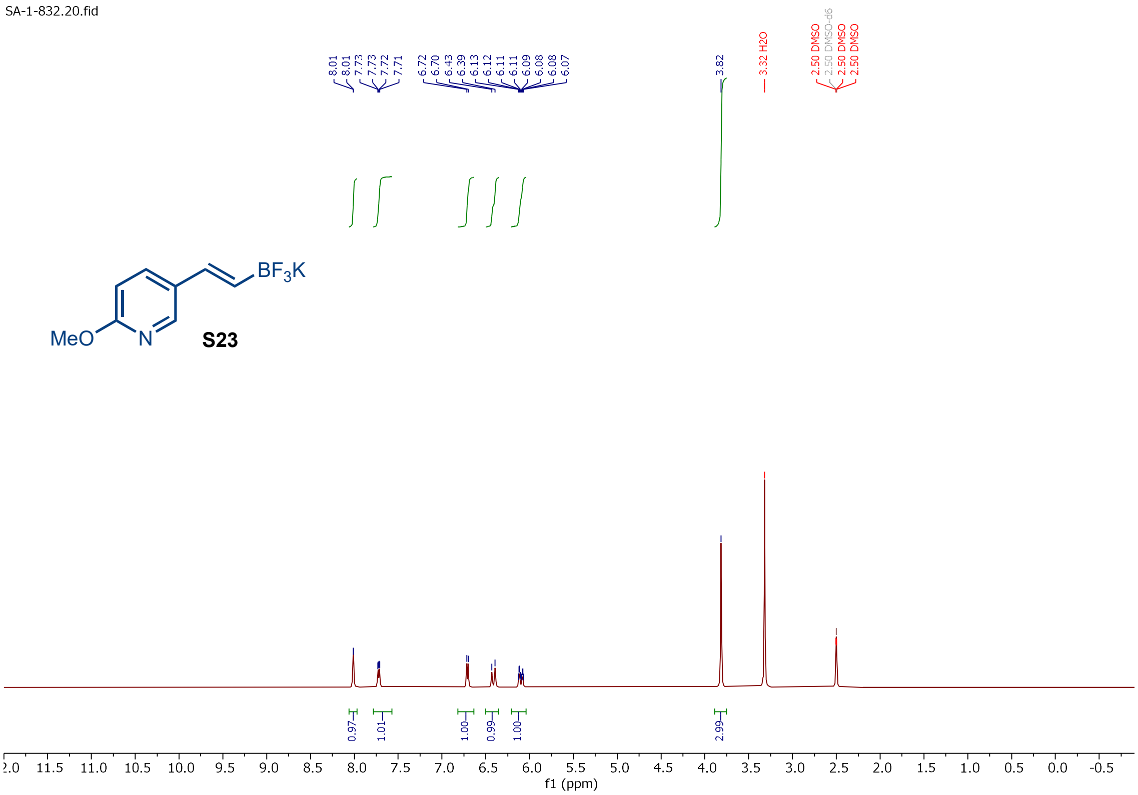
SA-1-832.20.fid
8.01
8.01
7.73
7.73
7.72
7.71
6.72
6.70
6.43
6.39
6.13
6.12
6.11
6.09
6.08
6.07
3.82
3.32 H2O
2.50 DMSO
2.50 DMSO-d6
2.50 DMSO
2.50 DMSO
BF3K
MeO
N
S23
0.97
1.01
1.00
0.99
1.00
2.99
f1 (ppm)

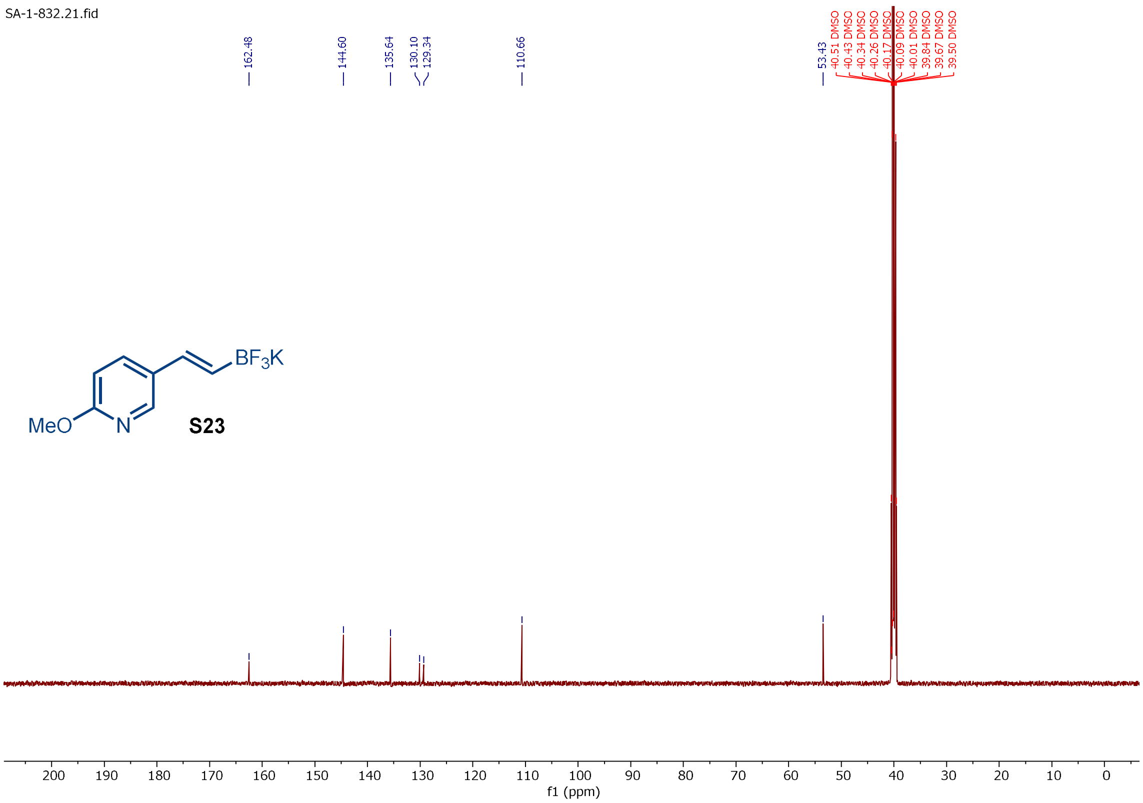
SA-1-832.21.fid
162.48
144.60
135.64
130.10
129.34
110.66
53.43
40.51 DMSO
40.43 DMSO
40.34 DMSO
40.26 DMSO
40.17 DMSO
40.09 DMSO
40.01 DMSO
39.84 DMSO
39.67 DMSO
39.50 DMSO
BF3K
MeO
N
S23
f1 (ppm)

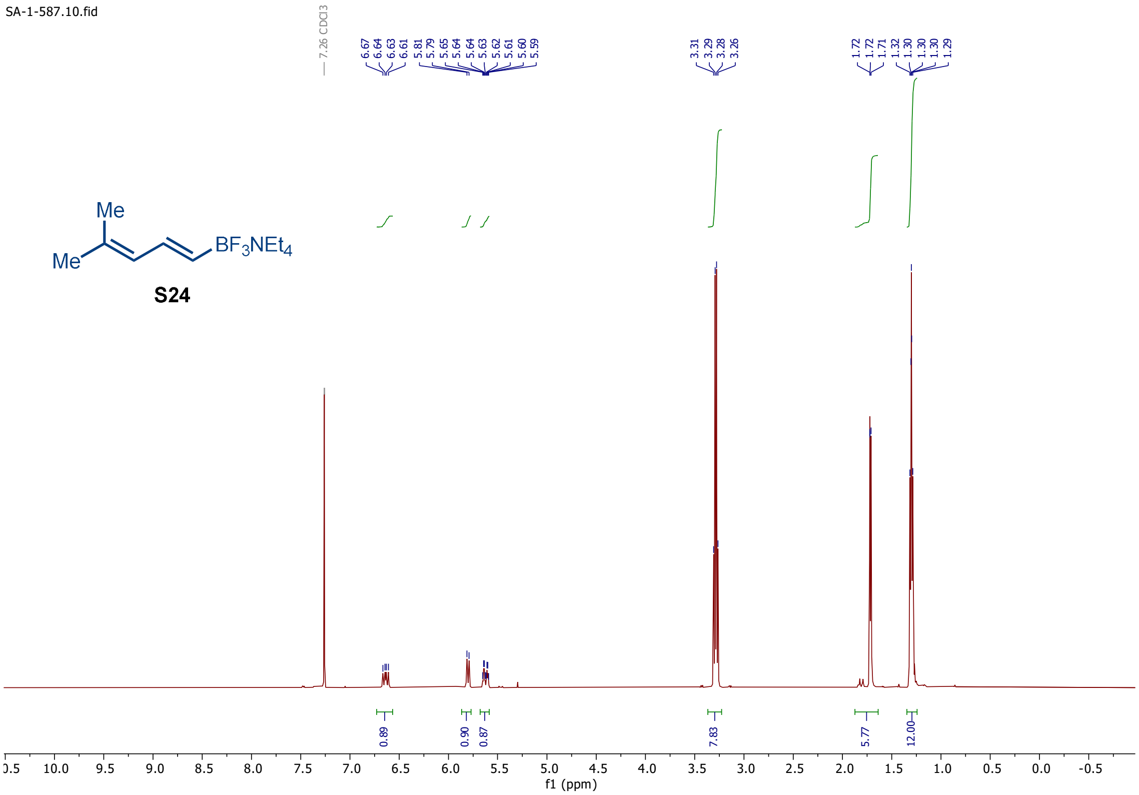
SA-1-587.10.fid
7.26 CDCl3
6.67
6.64
6.63
6.61
5.81
5.79
5.65
5.64
5.64
5.63
5.62
5.61
5.60
5.59
3.31
3.29
3.28
3.26
1.72
1.72
1.71
1.32
1.30
1.30
1.30
1.29
Me
Me
BF3NEt4
S24
0.89
0.90
0.87
7.83
5.77
12.00
f1 (ppm)

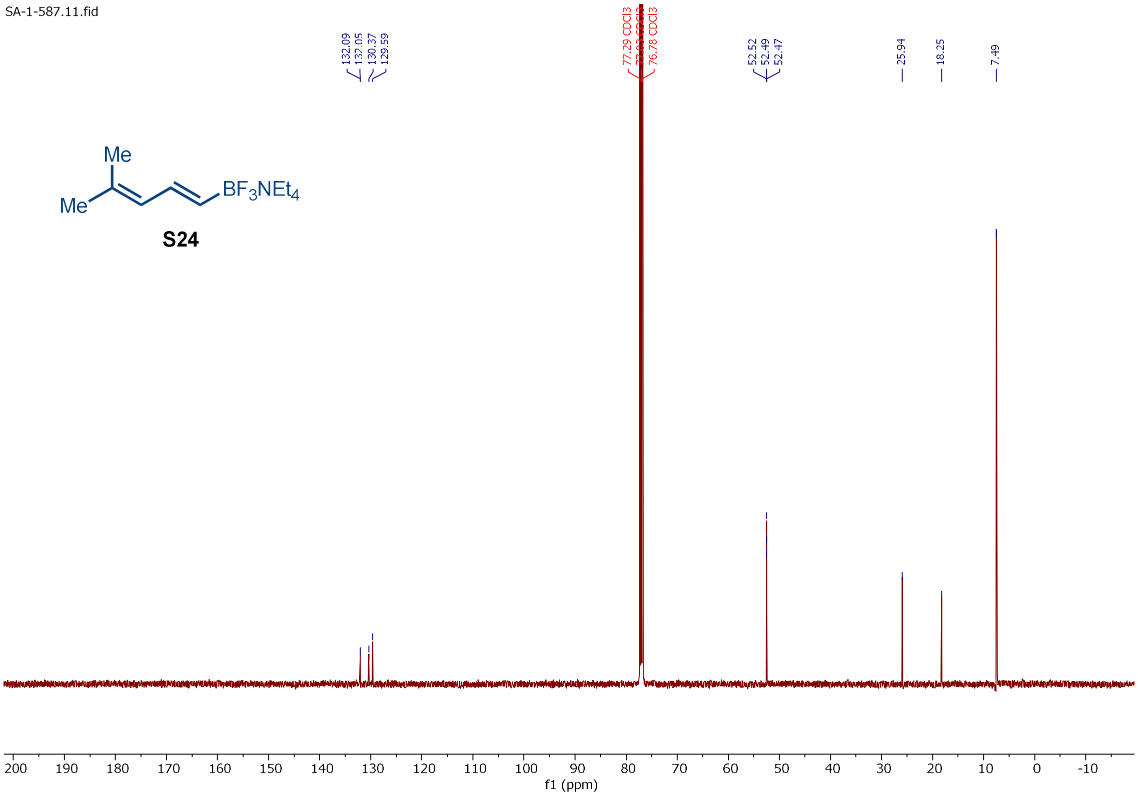
SA-1-587.11.fid
132.09
132.05
130.37
129.59
77.29 CDCl3
76.78 CDCl3
52.52
52.49
52.47
25.94
18.25
7.49
Me
Me
BF3NEt4
S24
200 190 180 170 160 150 140 130 120 110 100 90 80 70 60 50 40 30 20 10 0 -10
f1 (ppm)

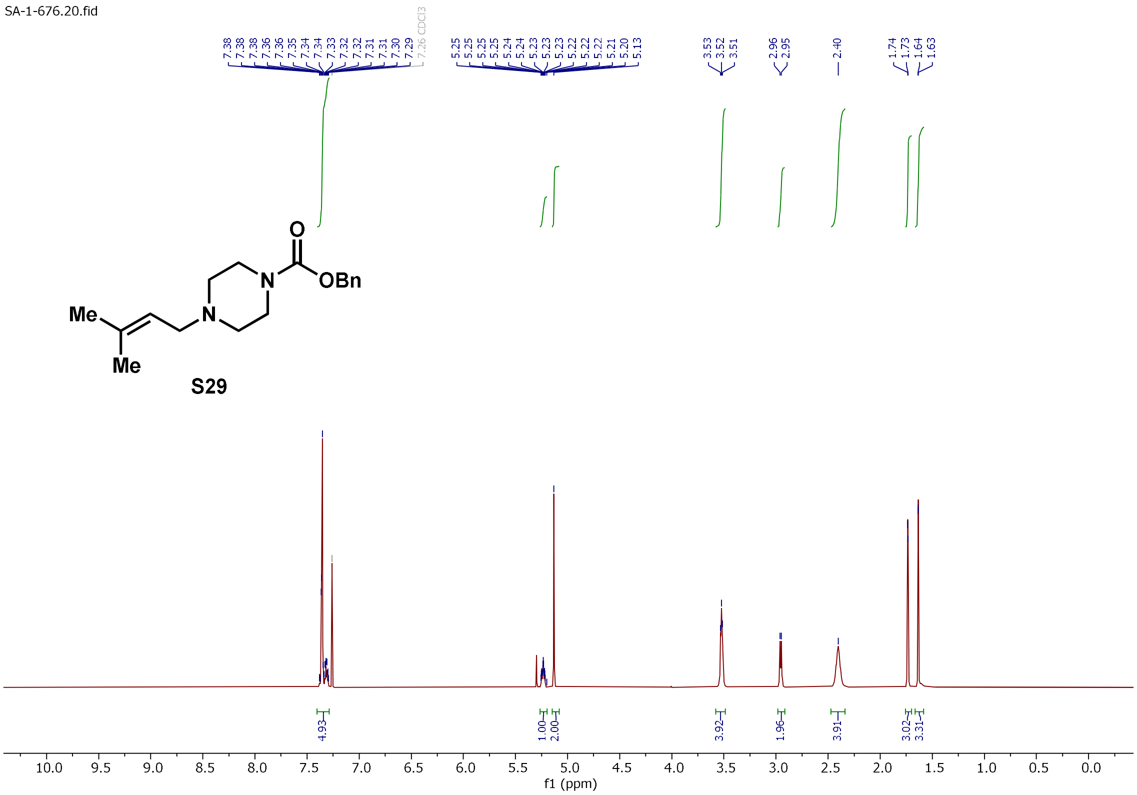
SA-1-676.20.fid
7.38
7.38
7.38
7.36
7.36
7.35
7.34
7.34
7.33
7.32
7.32
7.31
7.30
7.29
7.26 CDCl3
5.25
5.25
5.25
5.25
5.24
5.24
5.23
5.23
5.23
5.22
5.22
5.22
5.21
5.20
5.13
3.53
3.52
3.51
2.96
2.95
2.40
1.74
1.73
1.64
1.63
O
N
OBn
N
Me
Me
S29
4.93
1.00
2.00
3.92
1.96
3.91
3.02
3.31
10.0 9.5 9.0 8.5 8.0 7.5 7.0 6.5 6.0 5.5 5.0 4.5 4.0 3.5 3.0 2.5 2.0 1.5 1.0 0.5 0.0
f1 (ppm)

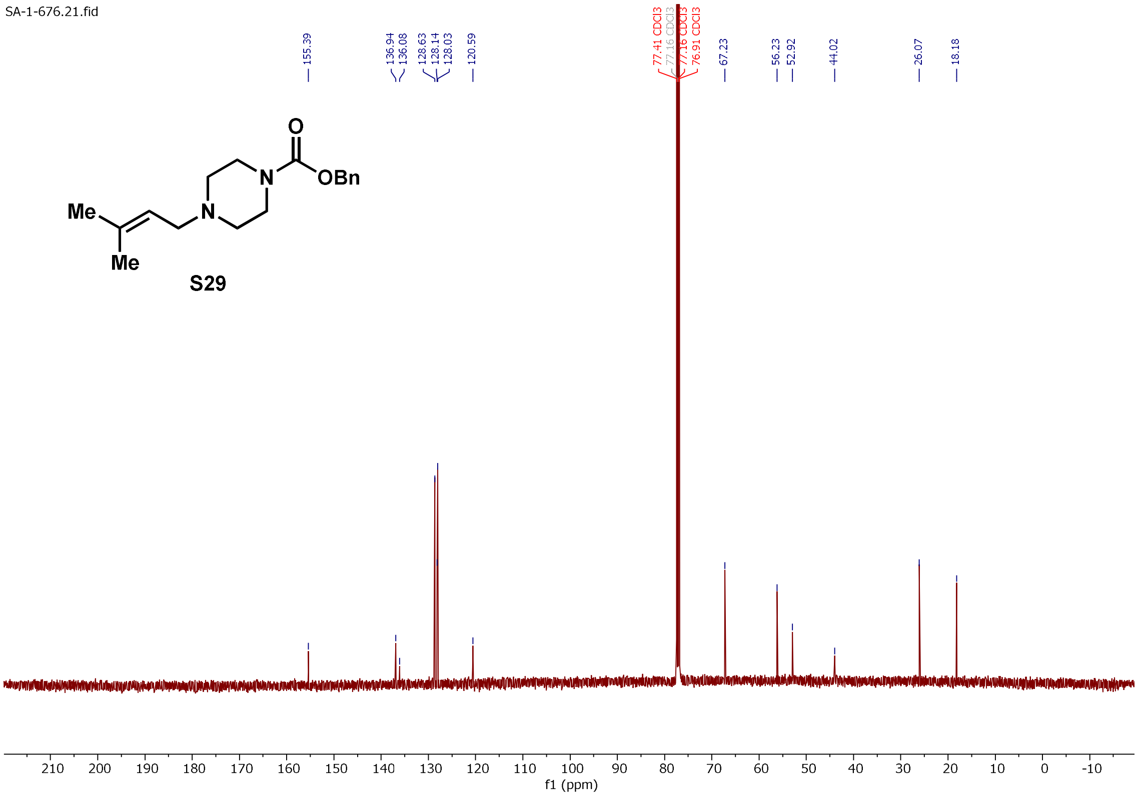
SA-1-676.21.fid
155.39
136.94
136.08
128.63
128.14
128.03
120.59
77.41 CDCl3
77.16 CDCl3
77.16 CDCl3
76.91 CDCl3
67.23
56.23
52.92
44.02
26.07
18.18
O
N
OBn
Me
N
Me
S29
210 200 190 180 170 160 150 140 130 120 110 100 90 80 70 60 50 40 30 20 10 0 -10
f1 (ppm)

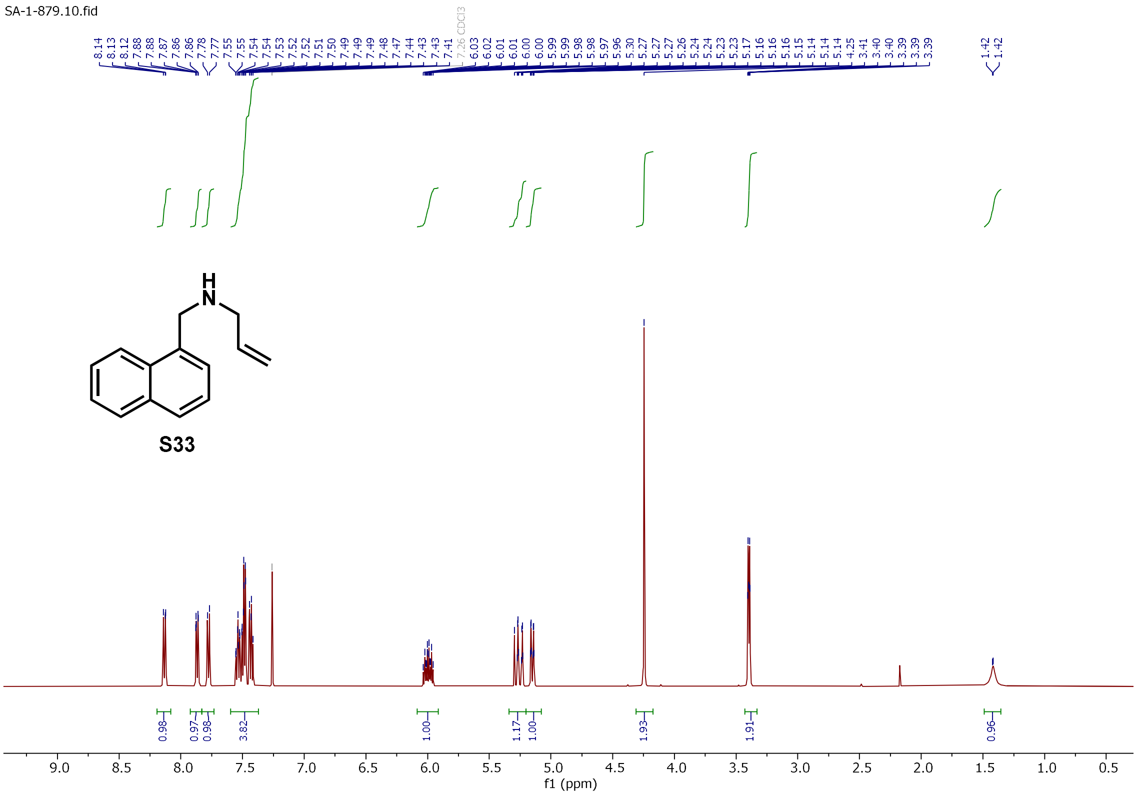
SA-1-879.10.fid
8.14 8.13 8.12 7.88 7.87 7.86 7.86 7.78 7.77 7.55 7.55 7.54 7.54 7.53 7.52 7.52 7.51 7.50 7.49 7.49 7.49 7.48 7.47 7.44 7.43 7.43 7.41
7.26 CDCl3
6.03 6.02 6.01 6.01 6.00 6.00 5.99 5.99 5.98 5.98 5.97 5.96 5.30 5.27 5.27 5.26 5.24 5.24 5.23 5.17 5.16 5.16 5.15 5.14 5.14 5.14 4.25 3.41 3.40 3.40 3.39 3.39 3.39
1.42 1.42
H
N
S33
0.98 0.97 0.98 3.82 1.00 1.17 1.00 1.93 1.91 0.96
9.0 8.5 8.0 7.5 7.0 6.5 6.0 5.5 5.0 4.5 4.0 3.5 3.0 2.5 2.0 1.5 1.0 0.5
f1 (ppm)

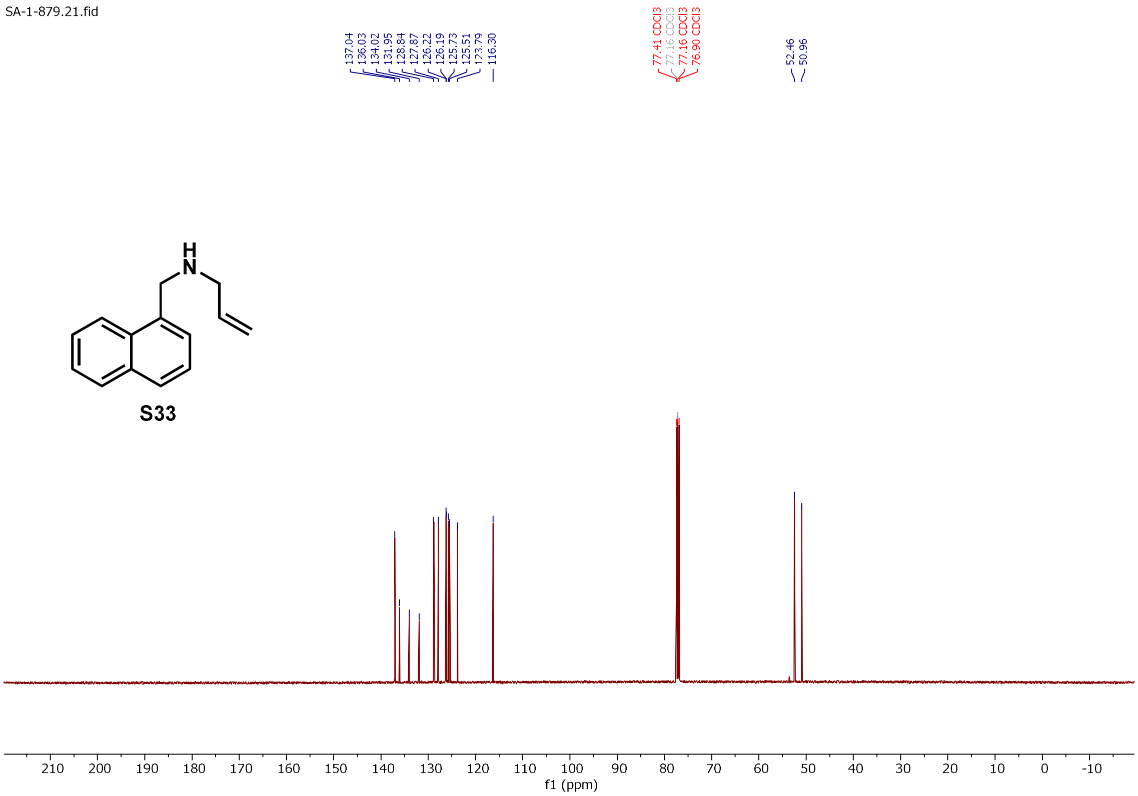
SA-1-879.21.fid
137.04
136.03
134.02
131.95
128.84
127.87
126.22
126.19
125.73
125.51
123.79
116.30
77.41 CDCl3
77.16 CDCl3
77.16 CDCl3
76.90 CDCl3
52.46
50.96
H
N
S33
210 200 190 180 170 160 150 140 130 120 110 100 90 80 70 60 50 40 30 20 10 0 -10
f1 (ppm)

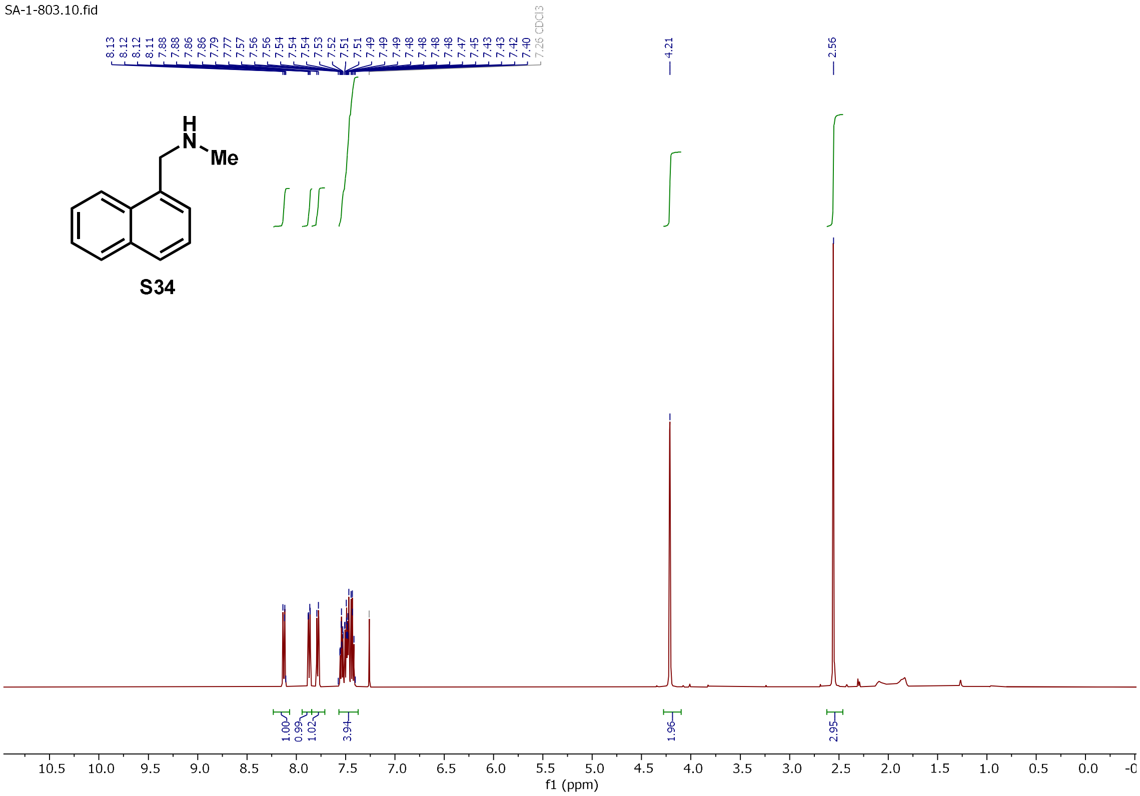
SA-1-803.10.fid
8.13
8.12
8.11
7.88
7.88
7.86
7.79
7.77
7.57
7.56
7.56
7.54
7.54
7.54
7.53
7.52
7.51
7.51
7.49
7.49
7.48
7.48
7.48
7.48
7.47
7.45
7.43
7.43
7.42
7.40
7.26 CDCl3
4.21
2.56
H
N
Me
S34
1.00
0.99
1.02
3.94
1.96
2.95
10.5 10.0 9.5 9.0 8.5 8.0 7.5 7.0 6.5 6.0 5.5 5.0 4.5 4.0 3.5 3.0 2.5 2.0 1.5 1.0 0.5 0.0
f1 (ppm)

SA-1-803.11.fid

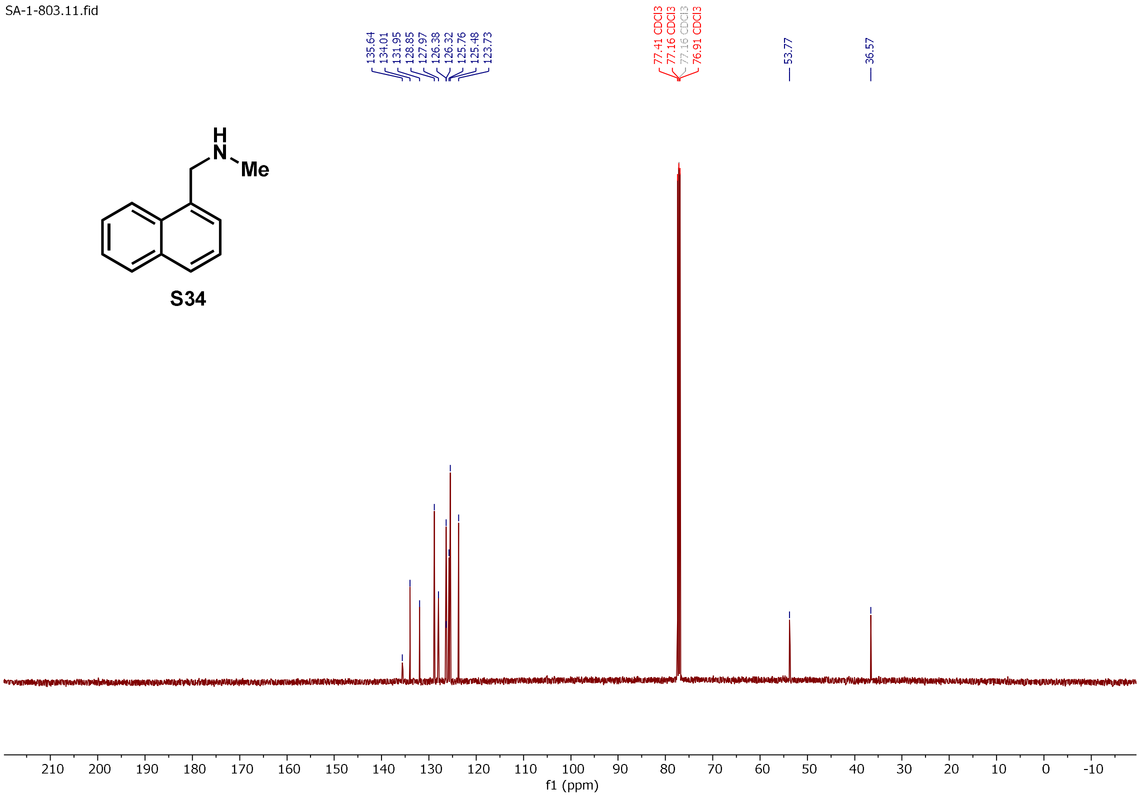

S34

SA-1-579-B-ii.10.fid

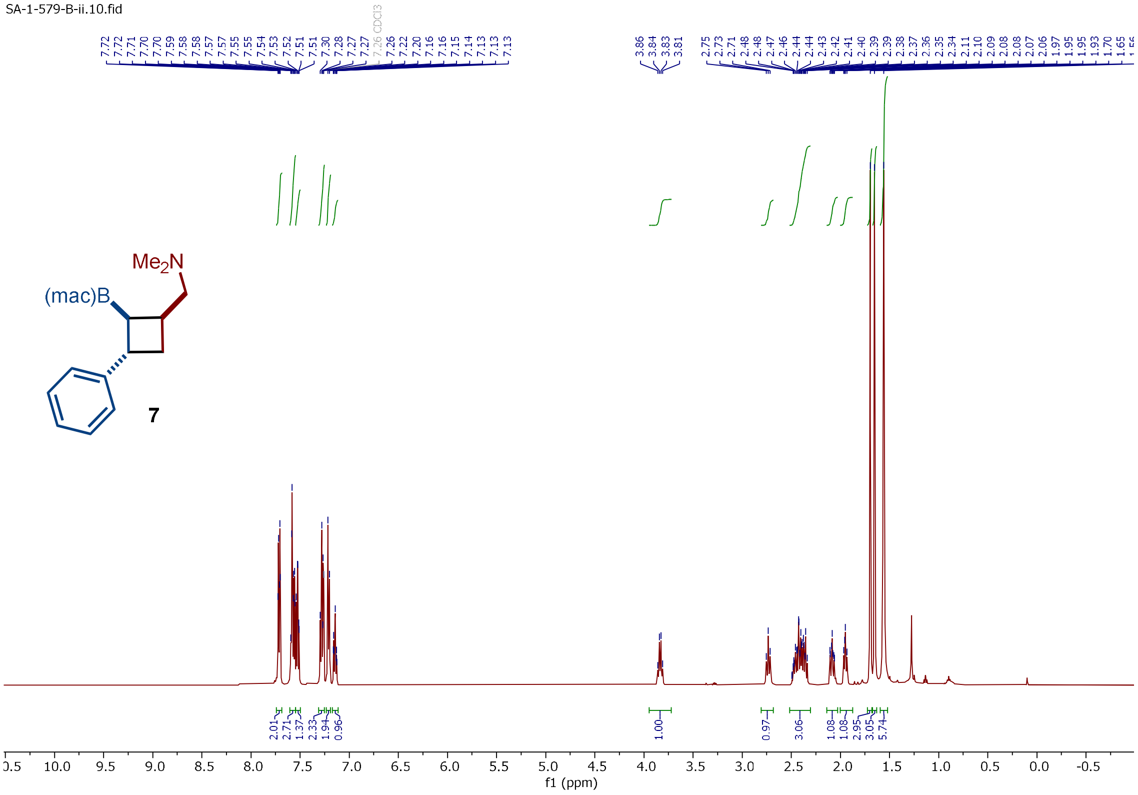

7

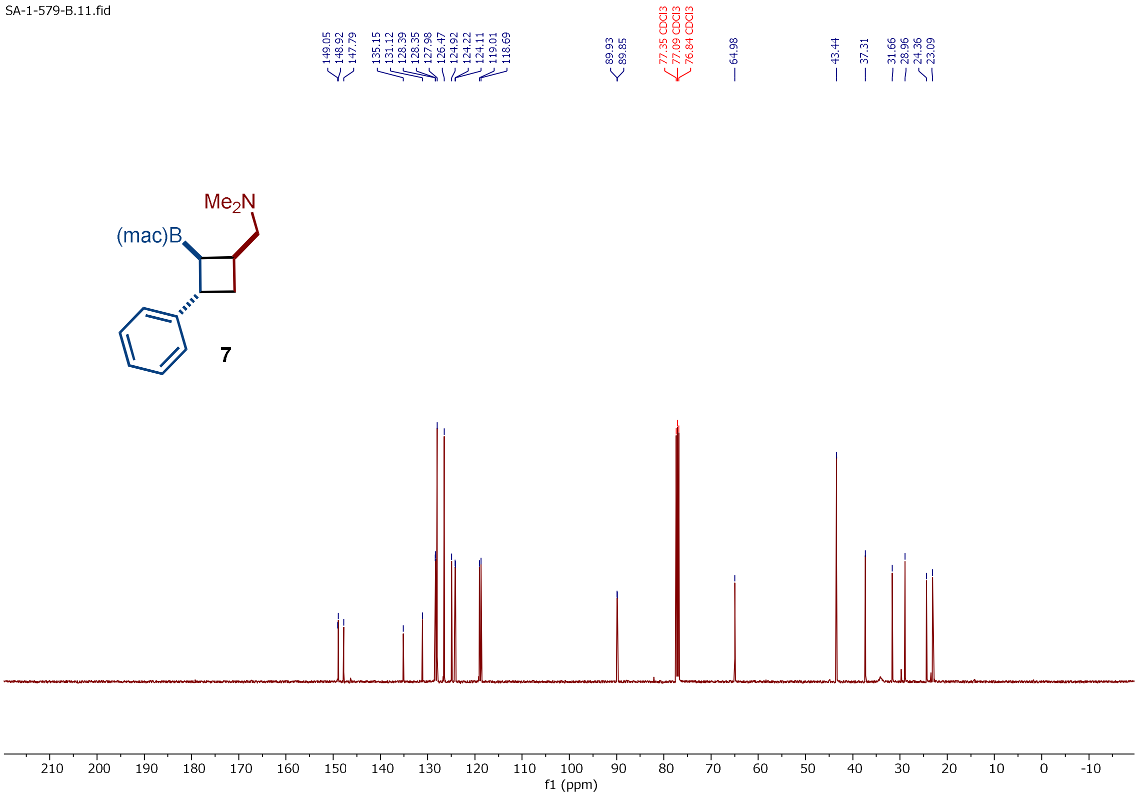

SA-1-579-B.11.fid
149.05
148.92
147.79
135.15
131.12
128.39
128.35
127.98
126.47
124.92
124.22
124.11
119.01
118.69
89.93
89.85
77.35 CDCl3
77.09 CDCl3
76.84 CDCl3
64.98
43.44
37.31
31.66
28.96
24.36
23.09
Me2N
(mac)B
7
210 200 190 180 170 160 150 140 130 120 110 100 90 80 70 60 50 40 30 20 10 0 -10
f1 (ppm)

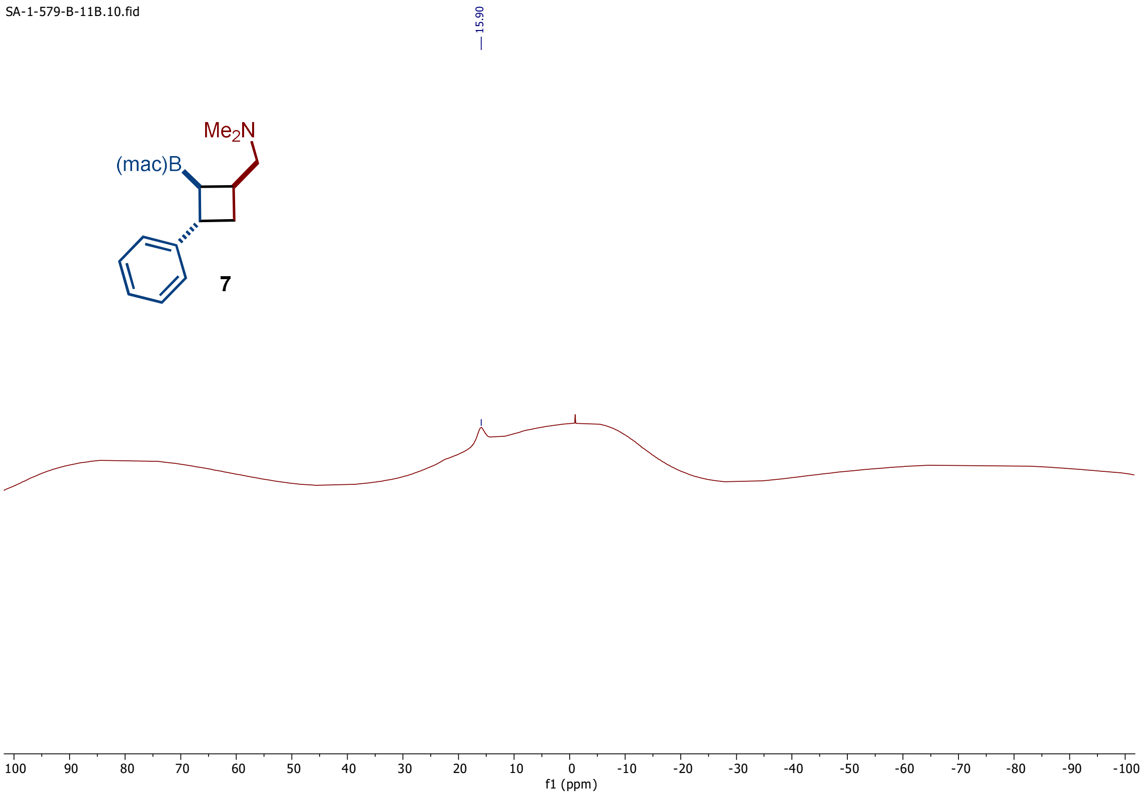

SA-1-579-B-11B.10.fid
15.90
Me2N
(mac)B
7
100 90 80 70 60 50 40 30 20 10 0 -10 -20 -30 -40 -50 -60 -70 -80 -90 -100
f1 (ppm)

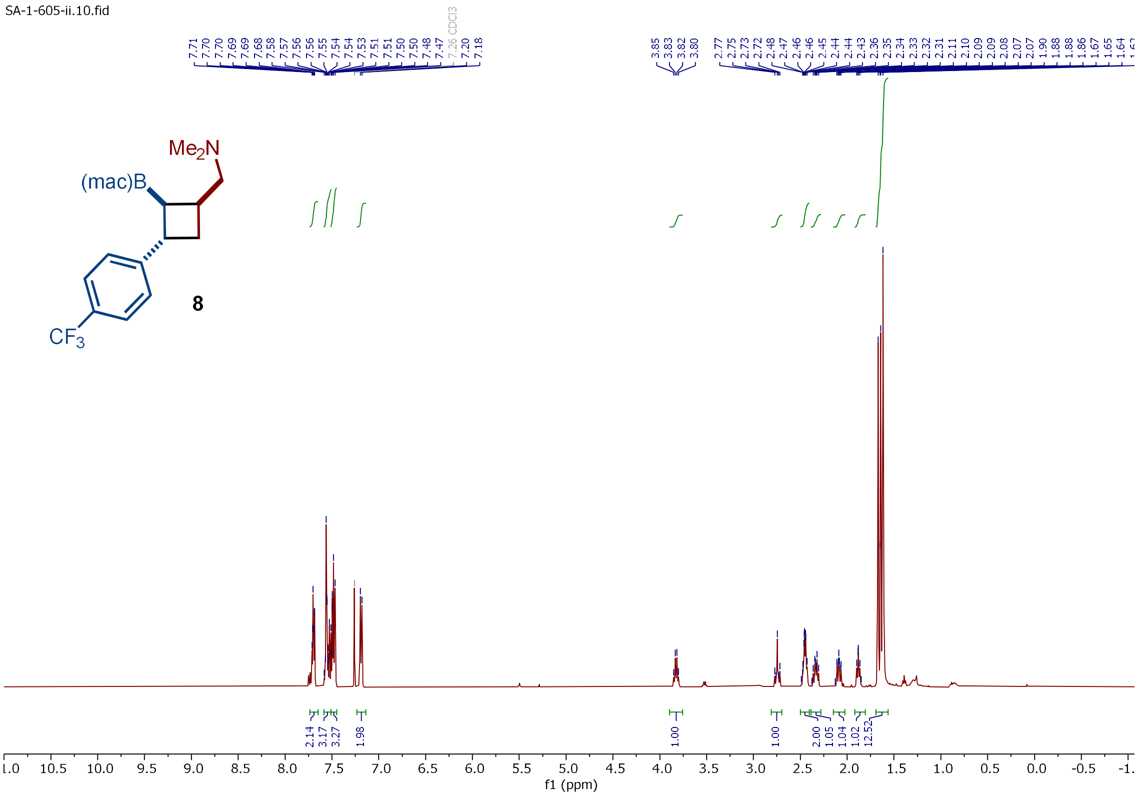
SA-1-605-ii.10.fid
7.71 7.70 7.70 7.69 7.69 7.68 7.58 7.57 7.56 7.56 7.55 7.54 7.53 7.51 7.51 7.50 7.48 7.47 7.26 CDCl3 7.20 7.18
3.85 3.83 3.82 3.80
2.77 2.75 2.73 2.72 2.48 2.47 2.46 2.46 2.45 2.44 2.44 2.43 2.36 2.35 2.34 2.33 2.32 2.31 2.11 2.10 2.09 2.09 2.08 2.07 2.07 1.90 1.88 1.88 1.86 1.67 1.65 1.64
Me2N
(mac)B
CF3
8
2.14 3.17 3.27 1.98 1.00 1.00 2.00 1.05 1.04 1.02 12.52
f1 (ppm)

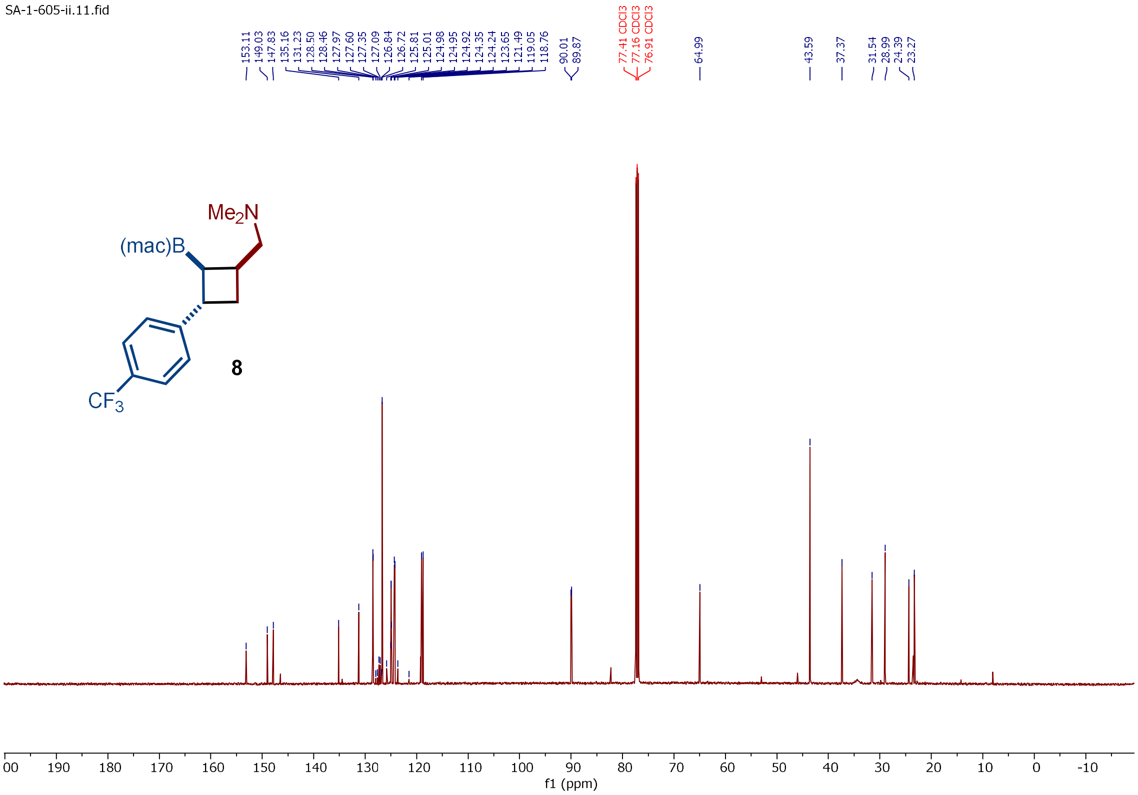
SA-1-605-ii.11.fid
153.11 149.03 147.83 135.16 131.23 128.50 128.46 127.97 127.60 127.35 127.09 126.84 126.72 125.81 125.01 124.98 124.95 124.92 124.35 124.24 123.65 121.49 119.05 118.76
90.01 89.87
77.41 CDCl3 77.16 CDCl3 76.91 CDCl3
64.99
43.59
37.37
31.54 28.99 24.39 23.27
Me2N
(mac)B
CF3
8
f1 (ppm)

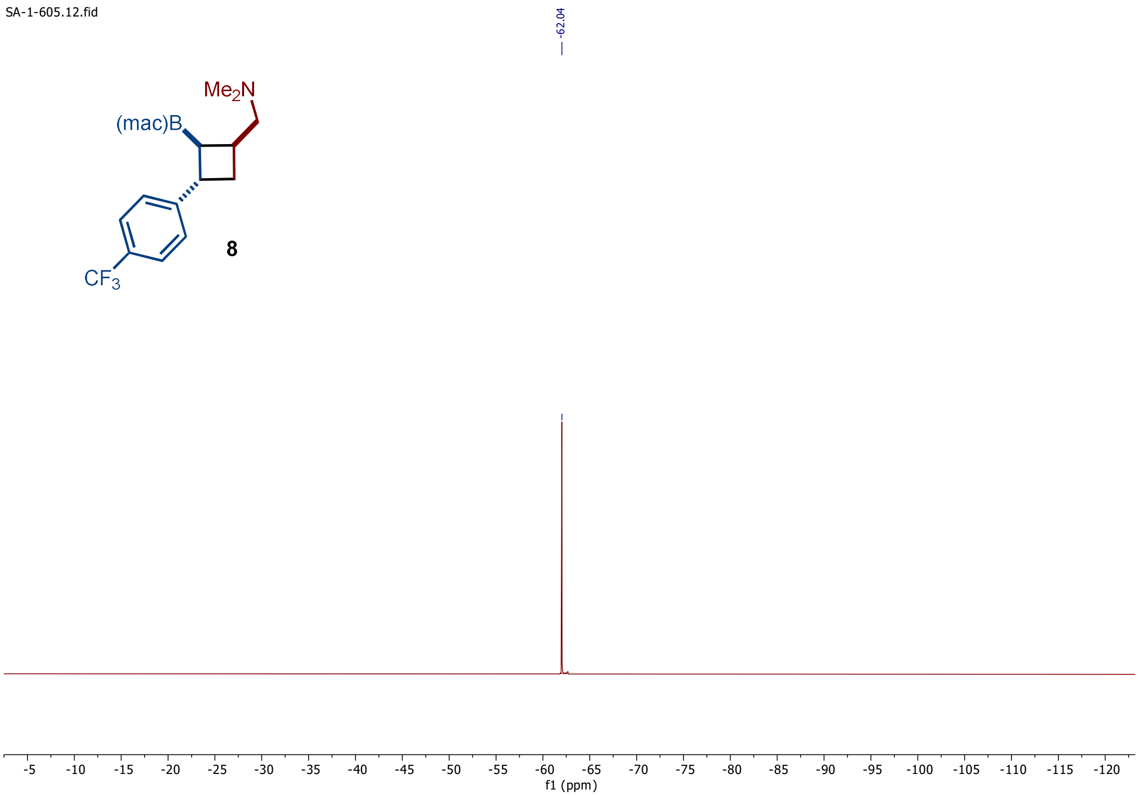
SA-1-605.12.fid
-62.04
Me_2N
(mac)B
CF_3
8
-5 -10 -15 -20 -25 -30 -35 -40 -45 -50 -55 -60 -65 -70 -75 -80 -85 -90 -95 -100 -105 -110 -115 -120
f1 (ppm)

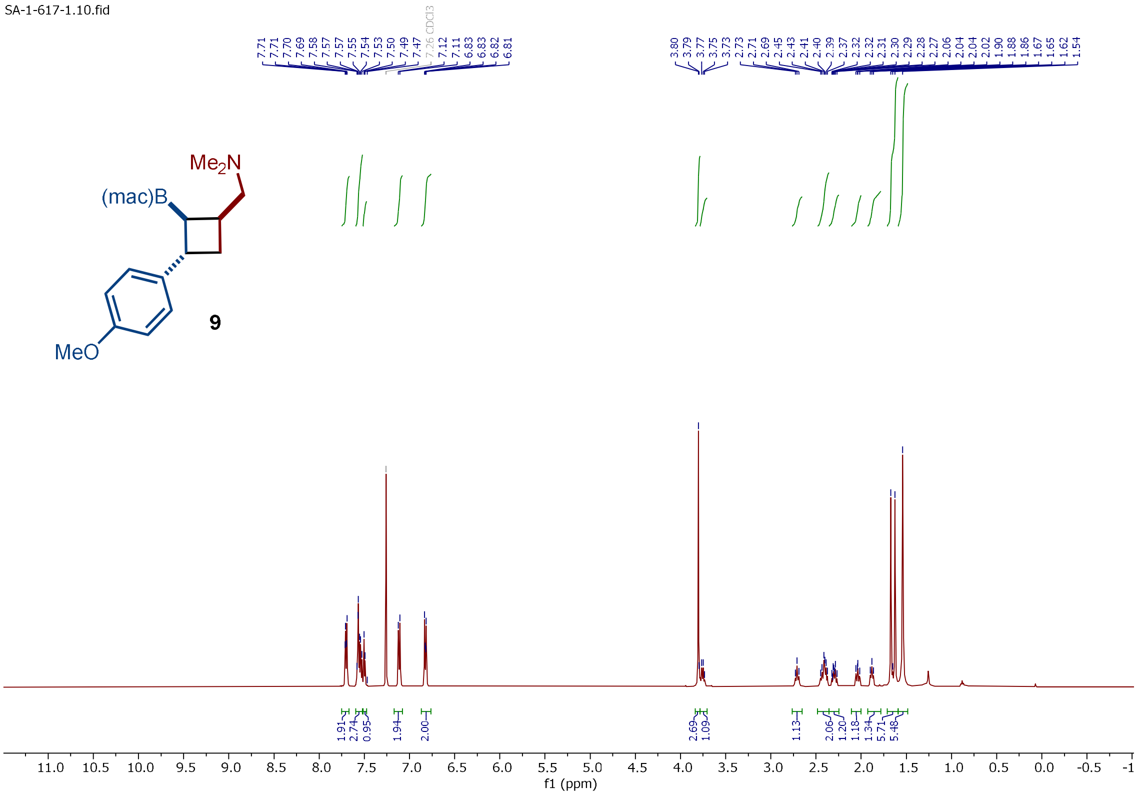
SA-1-617-1.10.fid
7.71 7.71 7.70 7.69 7.58 7.57 7.57 7.55 7.54 7.53 7.50 7.49 7.47 7.26 CDCl3 7.12 7.11 6.83 6.83 6.82 6.81
3.80 3.79 3.77 3.75 3.73 2.73 2.71 2.69 2.45 2.43 2.41 2.40 2.39 2.37 2.32 2.32 2.31 2.30 2.29 2.28 2.27 2.06 2.04 2.04 2.02 1.90 1.88 1.86 1.67 1.65 1.62 1.54
Me_2N
(mac)B
MeO
9
1.91 2.74 0.95 1.94 2.00
2.69 1.09
1.13 2.06 1.20 1.18 1.34 5.71 5.48
11.0 10.5 10.0 9.5 9.0 8.5 8.0 7.5 7.0 6.5 6.0 5.5 5.0 4.5 4.0 3.5 3.0 2.5 2.0 1.5 1.0 0.5 0.0 -0.5 -1
f1 (ppm)

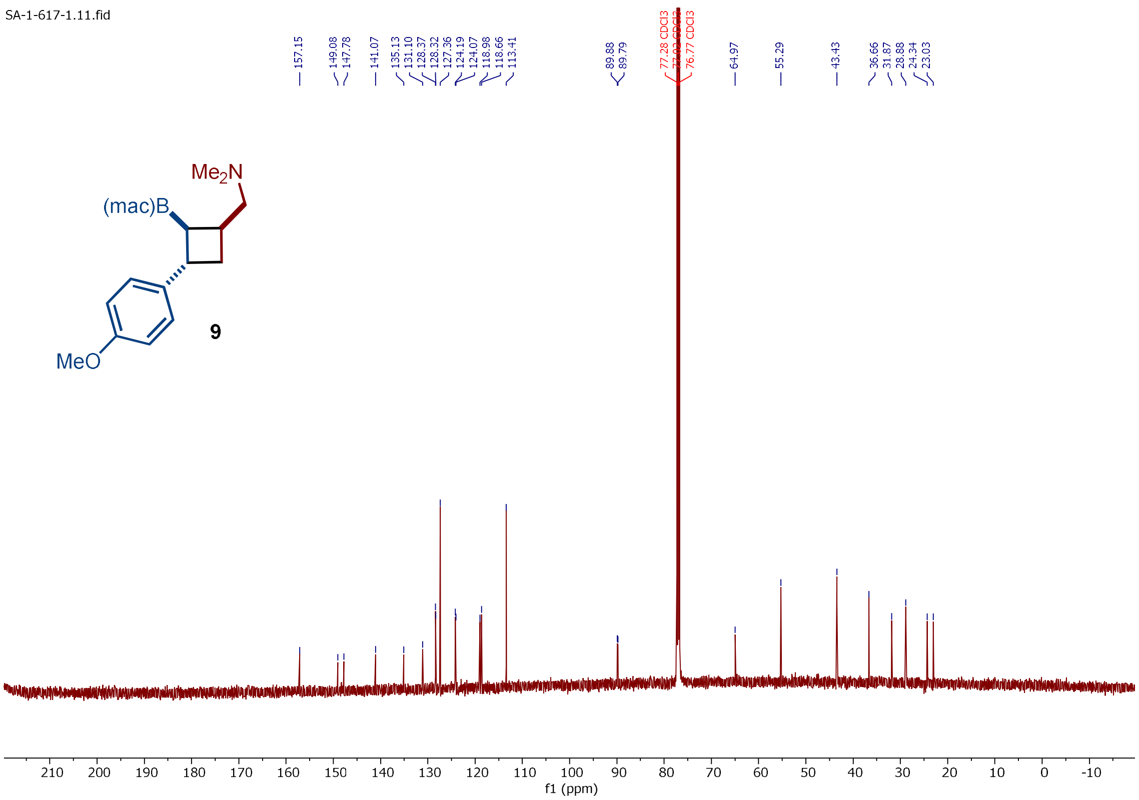
SA-1-617-1.11.fid
157.15
149.08
147.78
141.07
135.13
131.10
128.37
128.32
127.36
124.19
124.07
118.98
118.66
113.41
89.88
89.79
77.28 CDCl3
76.77 CDCl3
64.97
55.29
43.43
36.66
31.87
28.88
24.34
23.03
Me2N
(mac)B
MeO
9
f1 (ppm)

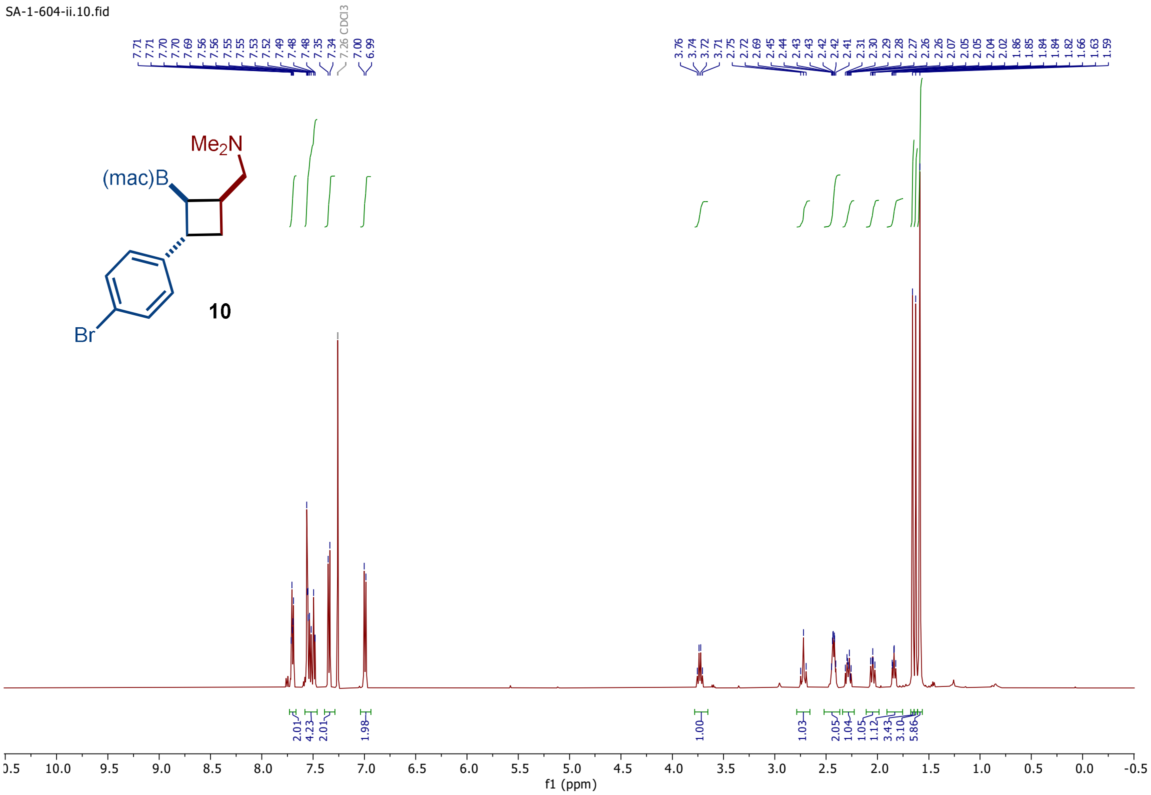
SA-1-604-ii.10.fid
7.71
7.71
7.70
7.70
7.69
7.56
7.56
7.55
7.55
7.53
7.52
7.49
7.48
7.35
7.34
7.26 CDCl3
7.00
6.99
3.76
3.74
3.72
3.71
2.75
2.72
2.69
2.45
2.44
2.43
2.43
2.42
2.42
2.41
2.31
2.30
2.29
2.28
2.27
2.26
2.26
2.07
2.05
2.05
2.04
2.02
1.86
1.85
1.84
1.84
1.82
1.66
1.63
1.59
Me2N
(mac)B
Br
10
2.01
4.23
2.01
1.98
1.00
1.03
2.05
1.04
1.05
1.12
3.43
3.10
5.86
f1 (ppm)

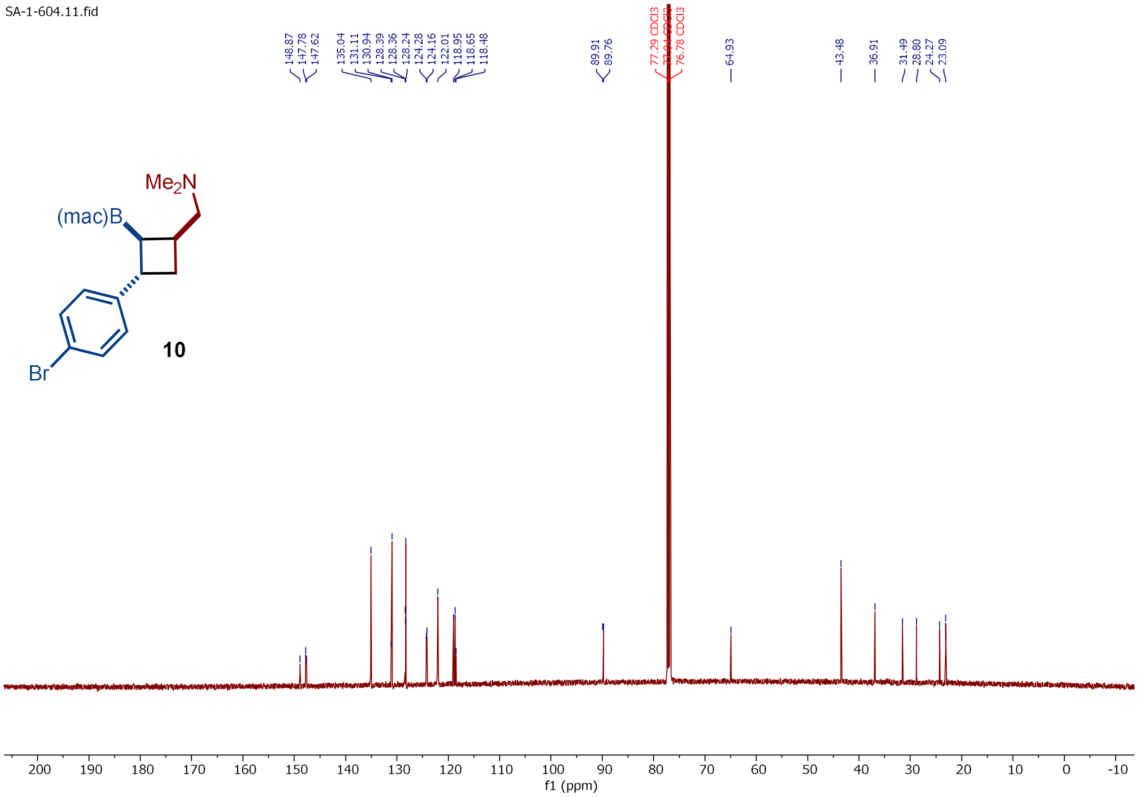
SA-1-604.11.fid
148.87
147.78
147.62
135.04
131.11
130.94
128.39
128.36
128.24
124.28
124.16
122.01
118.95
118.65
118.48
89.91
89.76
77.29 CDCl3
77.04 CDCl3
76.78 CDCl3
64.93
43.48
36.91
31.49
28.80
24.27
23.09
Me2N
(mac)B
Br
10
f1 (ppm)

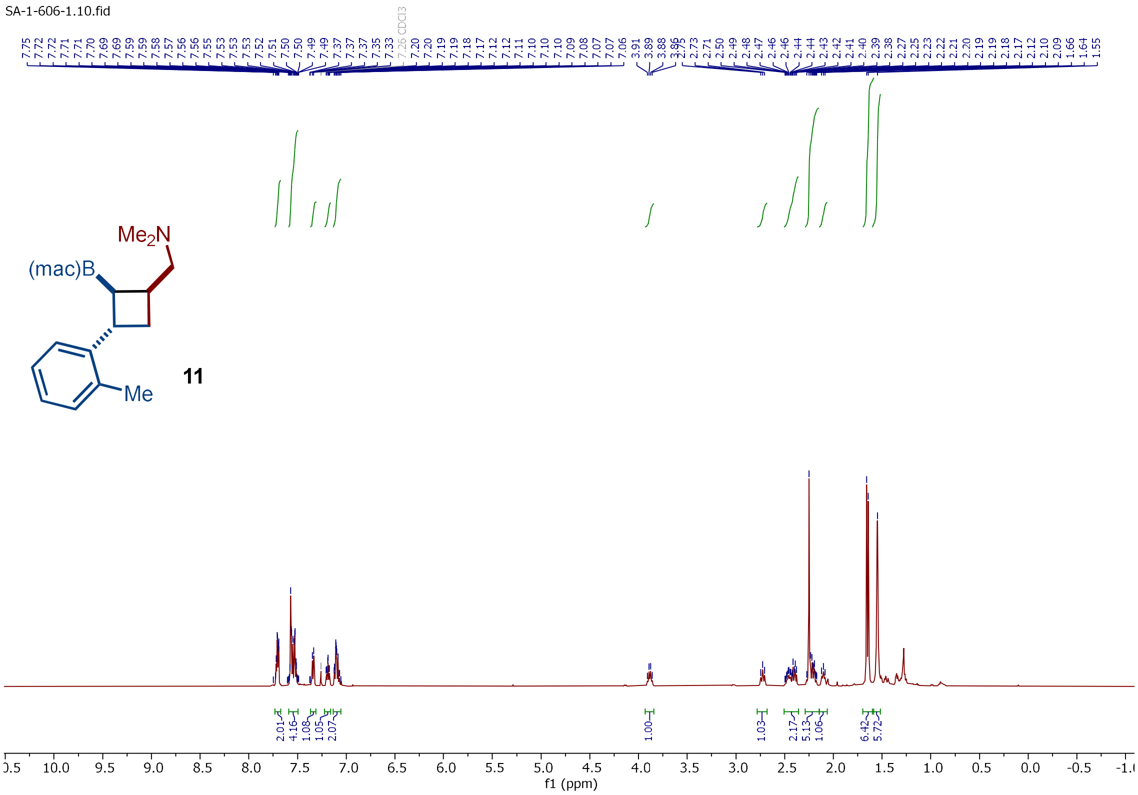
SA-1-606-1.10.fid
7.75 7.72 7.72 7.71 7.71 7.70 7.69 7.69 7.59 7.59 7.58 7.57 7.56 7.56 7.55 7.53 7.53 7.53 7.52 7.51 7.50 7.50 7.49 7.49 7.37 7.37 7.35 7.33
7.26 CDCl3
7.20 7.20 7.19 7.19 7.18 7.17 7.12 7.11 7.10 7.10 7.09 7.08 7.07 7.07 7.06
3.91 3.89 3.88 3.86
2.75 2.73 2.71 2.50 2.49 2.48 2.47 2.46 2.46 2.44 2.44 2.43 2.42 2.41 2.40 2.39 2.38 2.27 2.25 2.23 2.22 2.21 2.20 2.19 2.19 2.18 2.17 2.12 2.10 2.09 1.66 1.64 1.55
2.01 4.16 1.08 1.05 2.07 1.00 1.03 2.17 5.13 1.06 6.42 5.72
Me2N
(mac)B
Me
11
f1 (ppm)

SA-1-606-1.11.fid

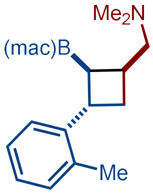

11

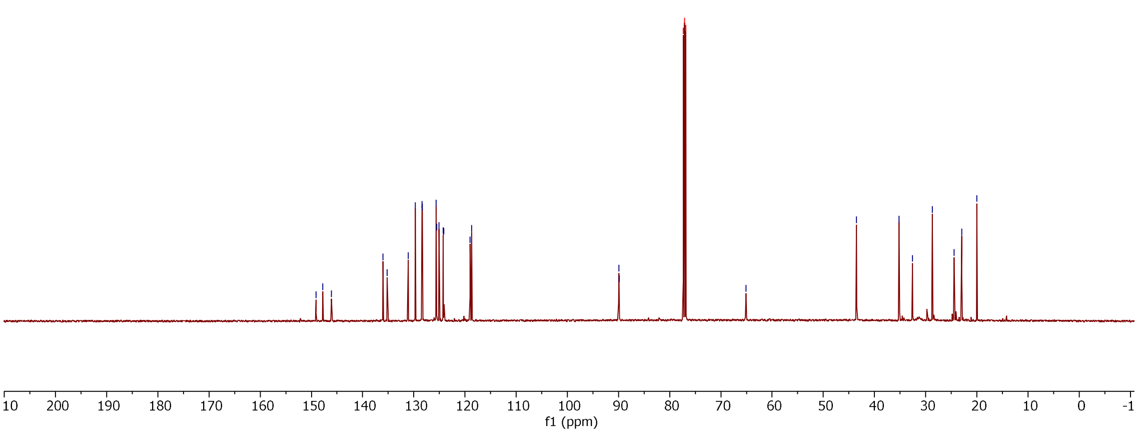

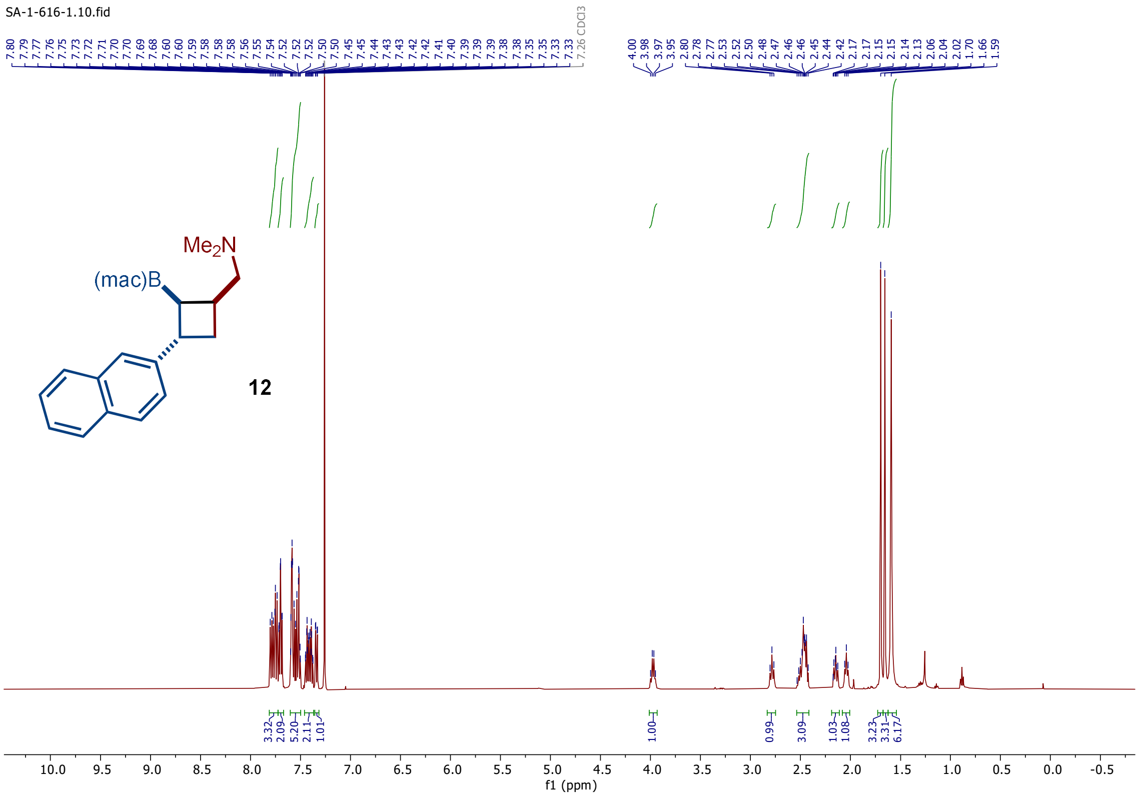
SA-1-616-1.10.fid
7.80 7.79 7.77 7.76 7.75 7.73 7.72 7.71 7.70 7.70 7.69 7.68 7.60 7.59 7.58 7.58 7.58 7.58 7.56 7.55 7.54 7.52 7.52 7.52 7.50 7.50 7.45 7.45 7.44 7.43 7.43 7.42 7.42 7.41 7.40 7.39 7.39 7.39 7.38 7.38 7.35 7.35 7.33 7.33
7.26 CDCl3
4.00 3.98 3.97 3.95 2.80 2.78 2.77 2.53 2.52 2.50 2.48 2.47 2.46 2.46 2.45 2.44 2.42 2.17 2.17 2.15 2.15 2.14 2.13 2.06 2.04 2.02 1.70 1.66 1.59
Me2N
(mac)B
12
3.32 2.09 5.20 2.11 1.01
1.00
0.99 3.09
1.03 1.08
3.23 3.31 6.17
f1 (ppm)

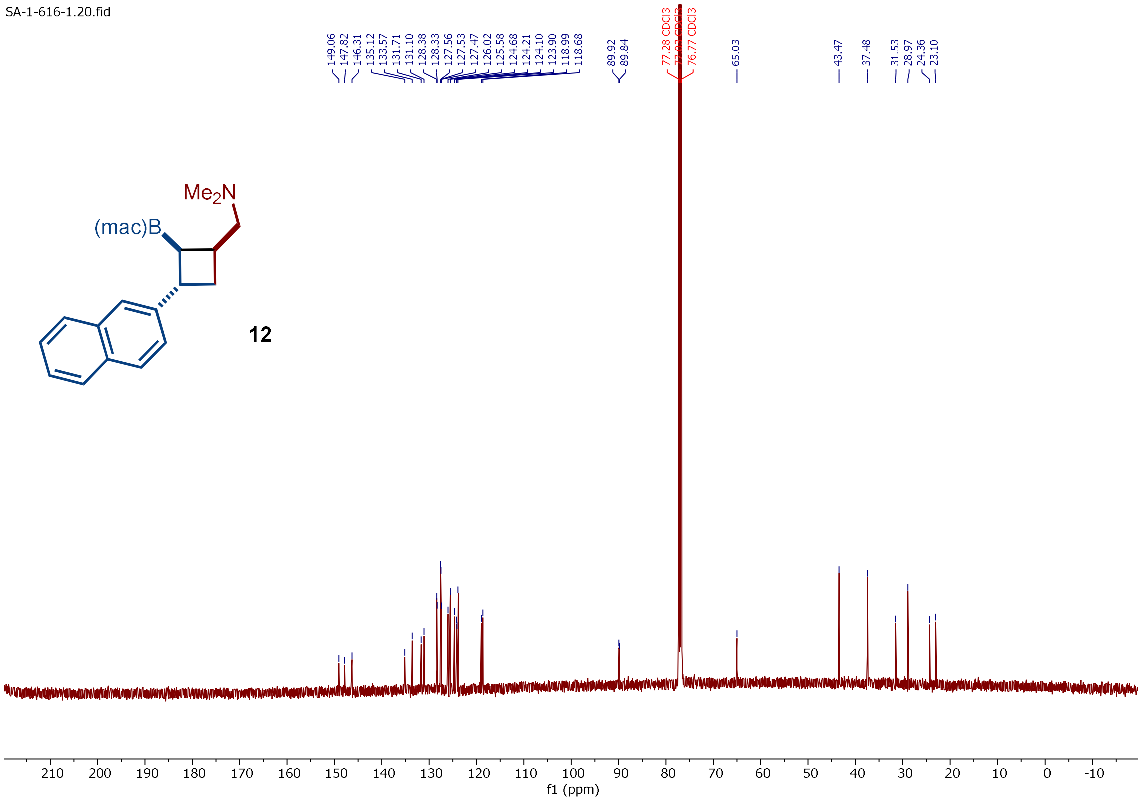
SA-1-616-1.20.fid
149.06 147.82 146.31 135.12 133.57 131.71 131.10 128.38 128.33 127.56 127.53 127.47 126.02 125.58 124.68 124.21 124.10 123.90 118.99 118.68
89.92 89.84
77.28 CDCl3
77.03 CDCl3
76.77 CDCl3
65.03
43.47 37.48 31.53 28.97 24.36 23.10
Me2N
(mac)B
12
f1 (ppm)

SA-1-618-1.10.fid

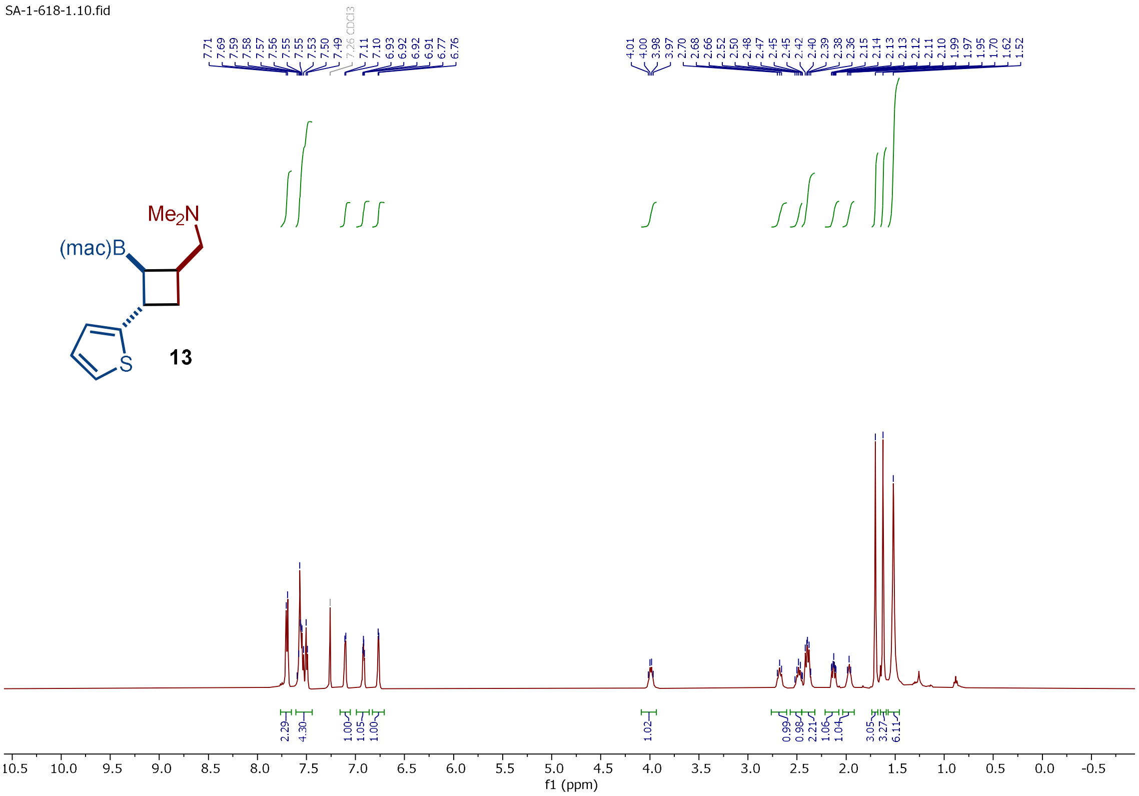

SA-1-618-1.11.fid

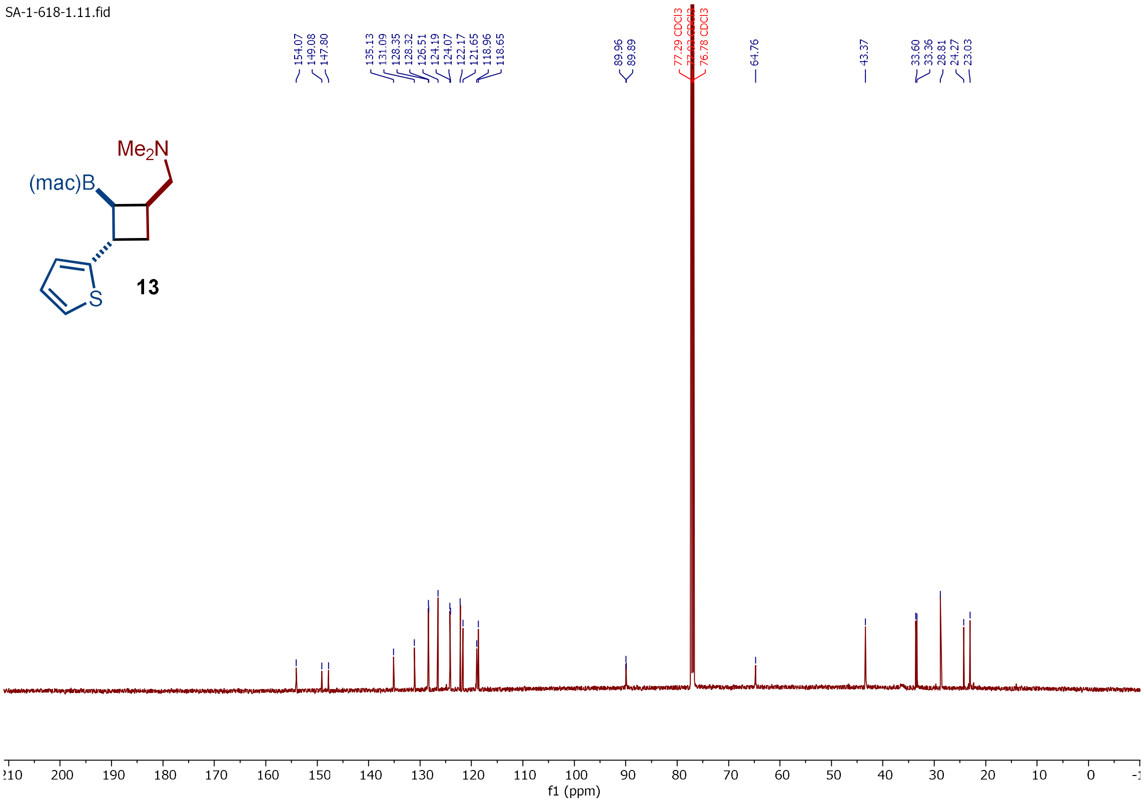

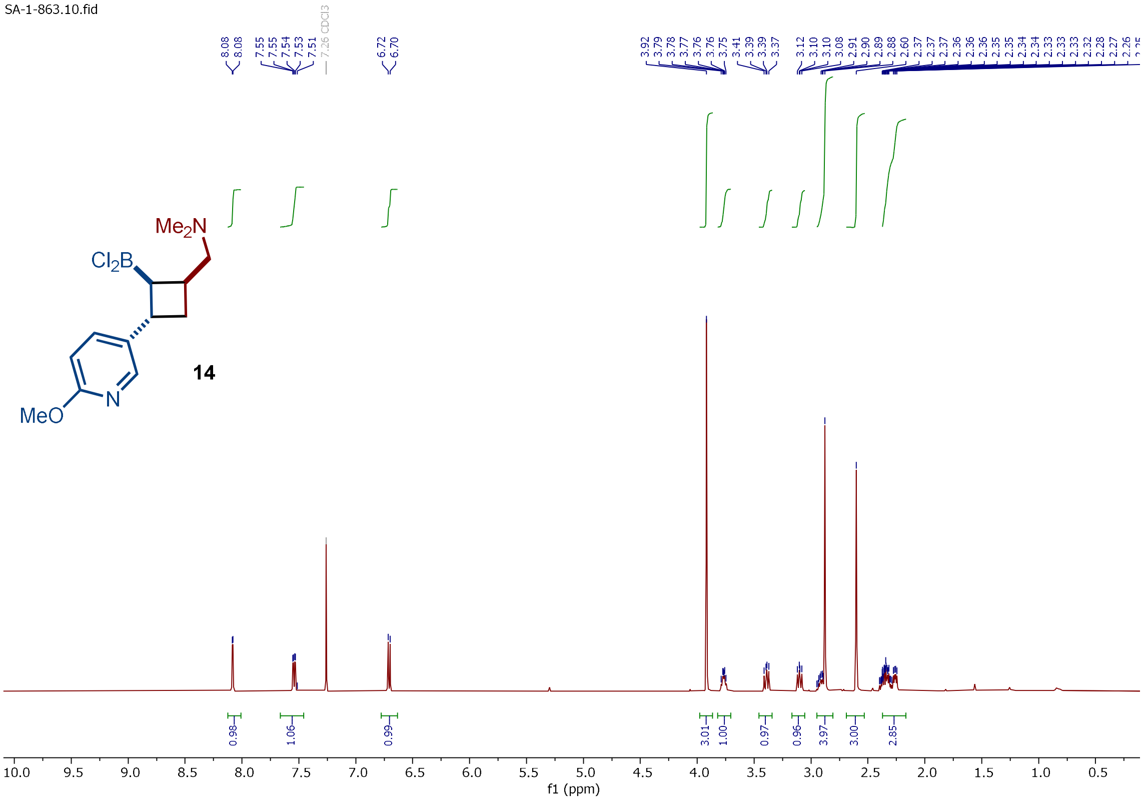
SA-1-863.10.fid
Me2N
Cl2B
MeO
N
14
f1 (ppm)

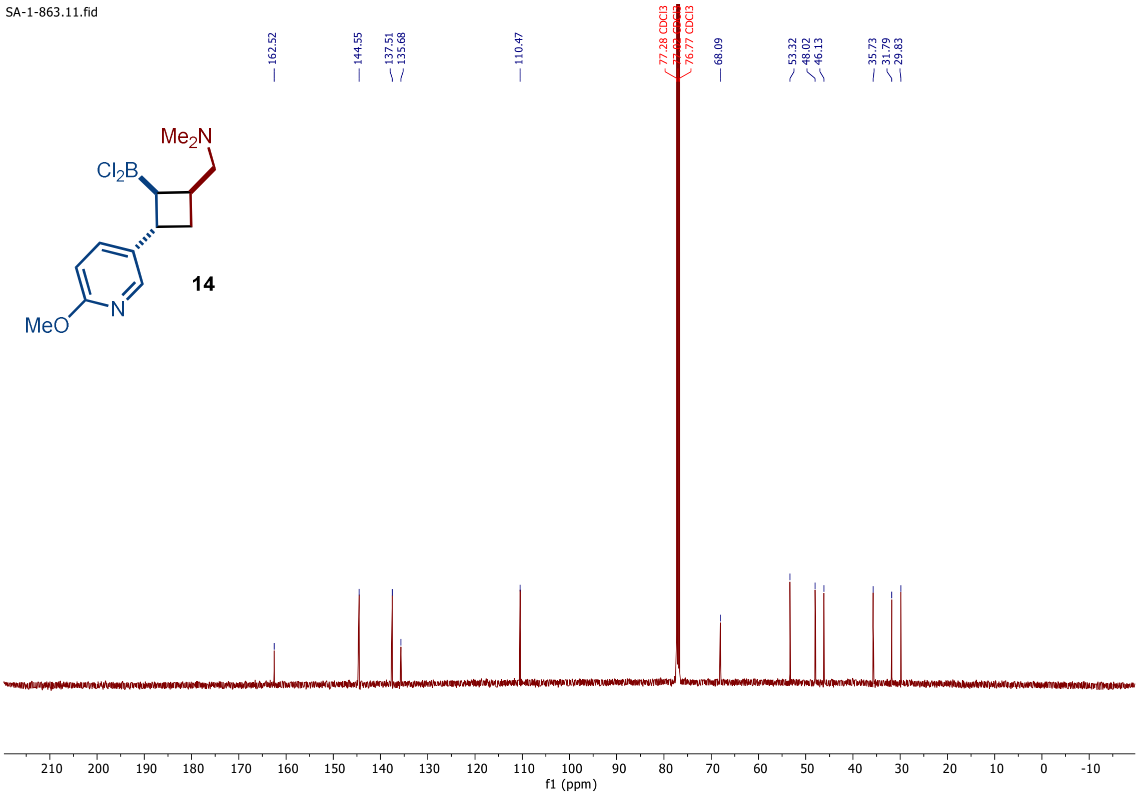
SA-1-863.11.fid
162.52
144.55
137.51
135.68
110.47
77.28 CDCl3
76.77 CDCl3
68.09
53.32
48.02
46.13
35.73
31.79
29.83
Me2N
Cl2B
MeO
N
14
f1 (ppm)

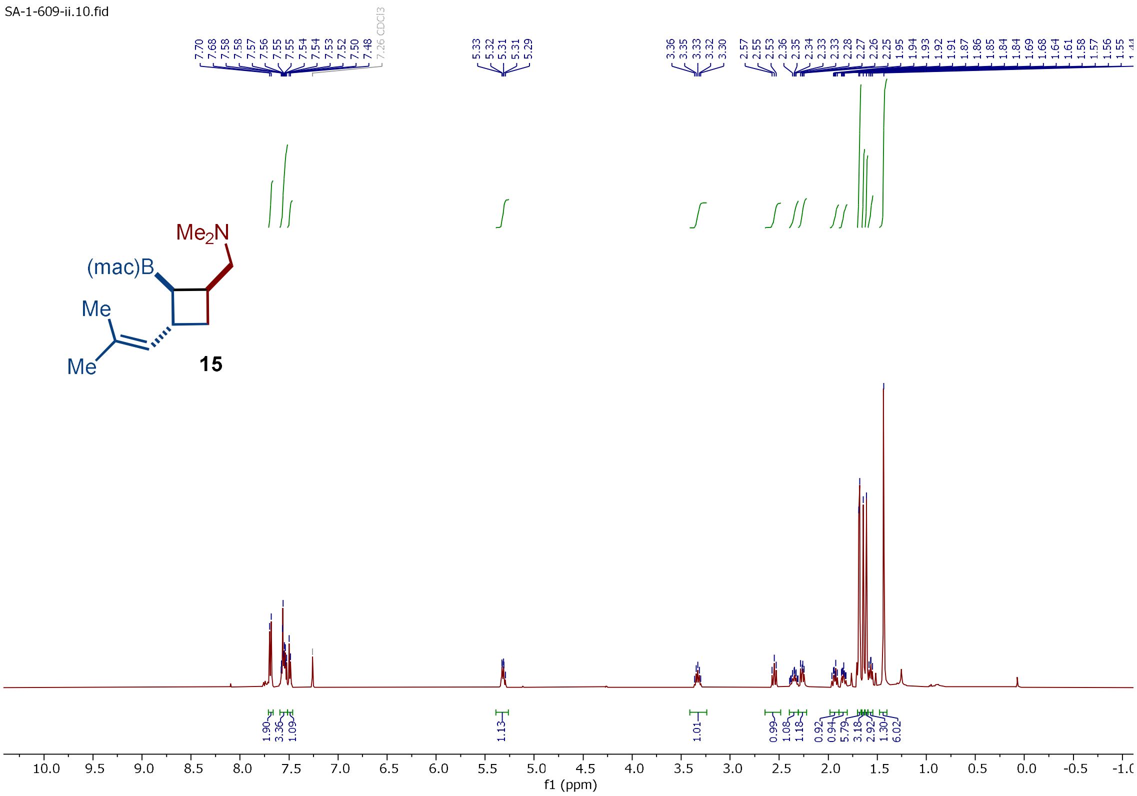
SA-1-609-ii.10.fid
Me2N
(mac)B
Me
Me
15
f1 (ppm)

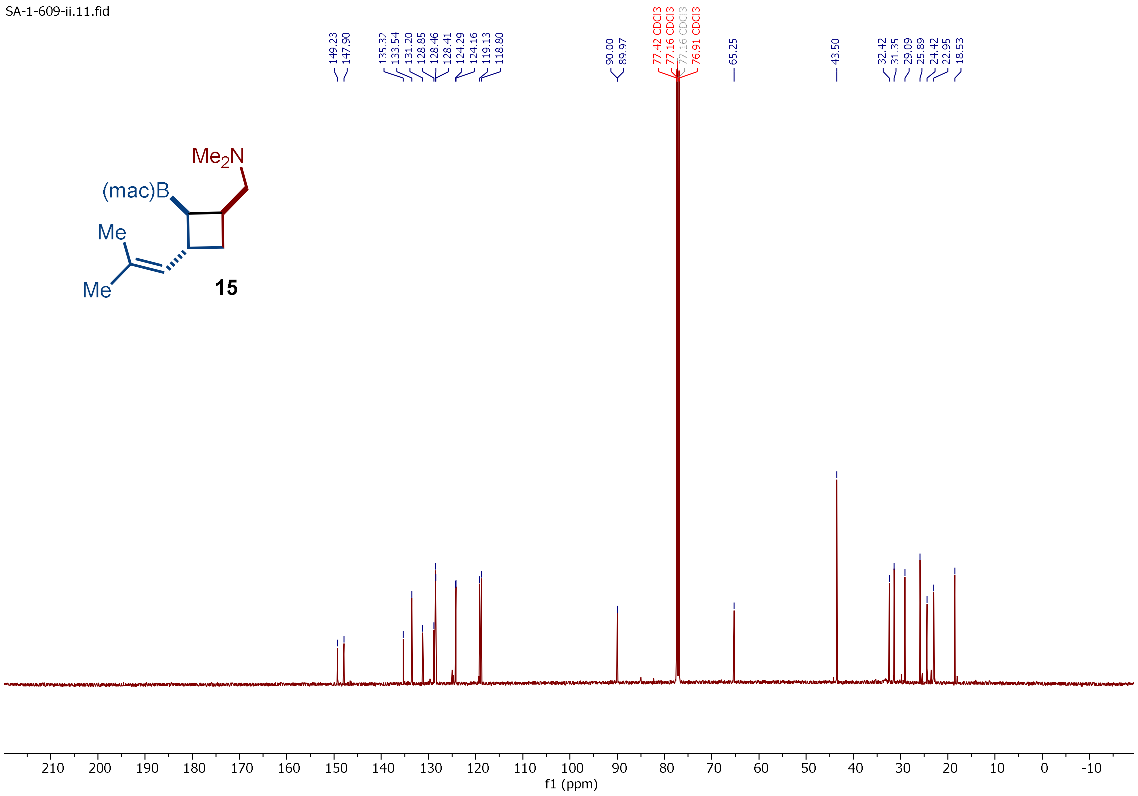
SA-1-609-ii.11.fid
Me2N
(mac)B
Me
Me
15
f1 (ppm)

SA-1-632-1.10.fid

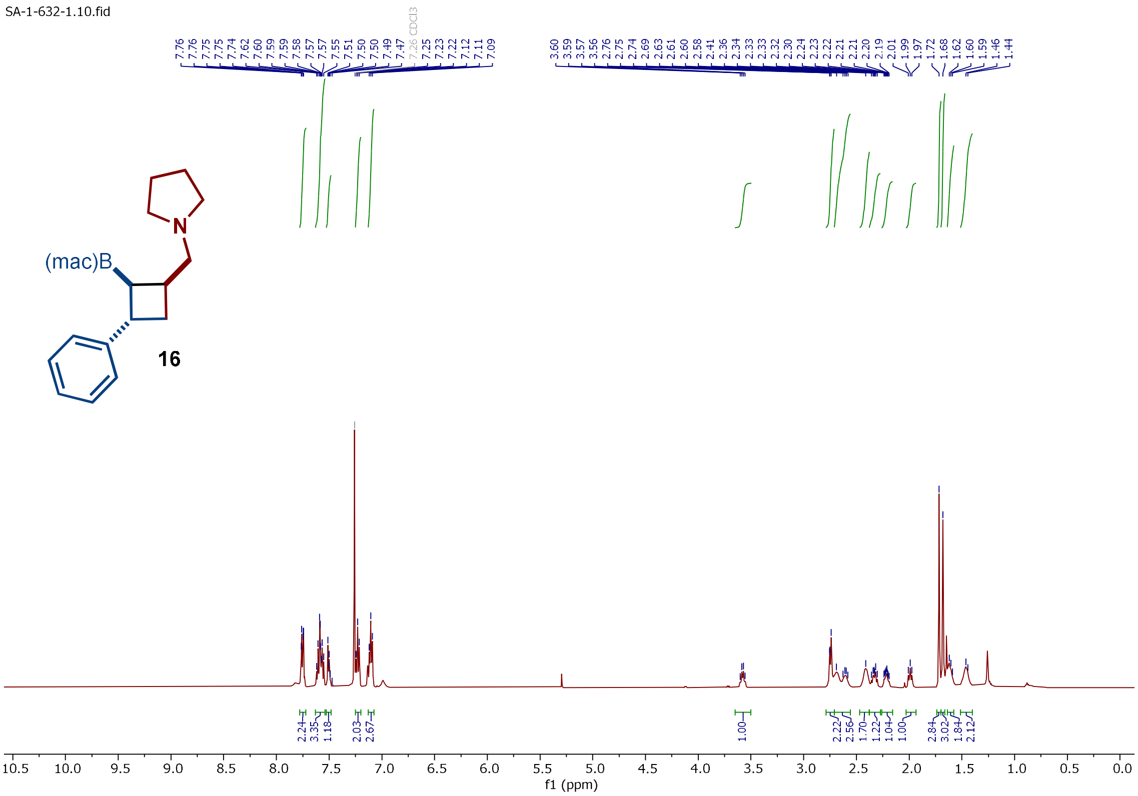

16

SA-1-632-1.20.fid

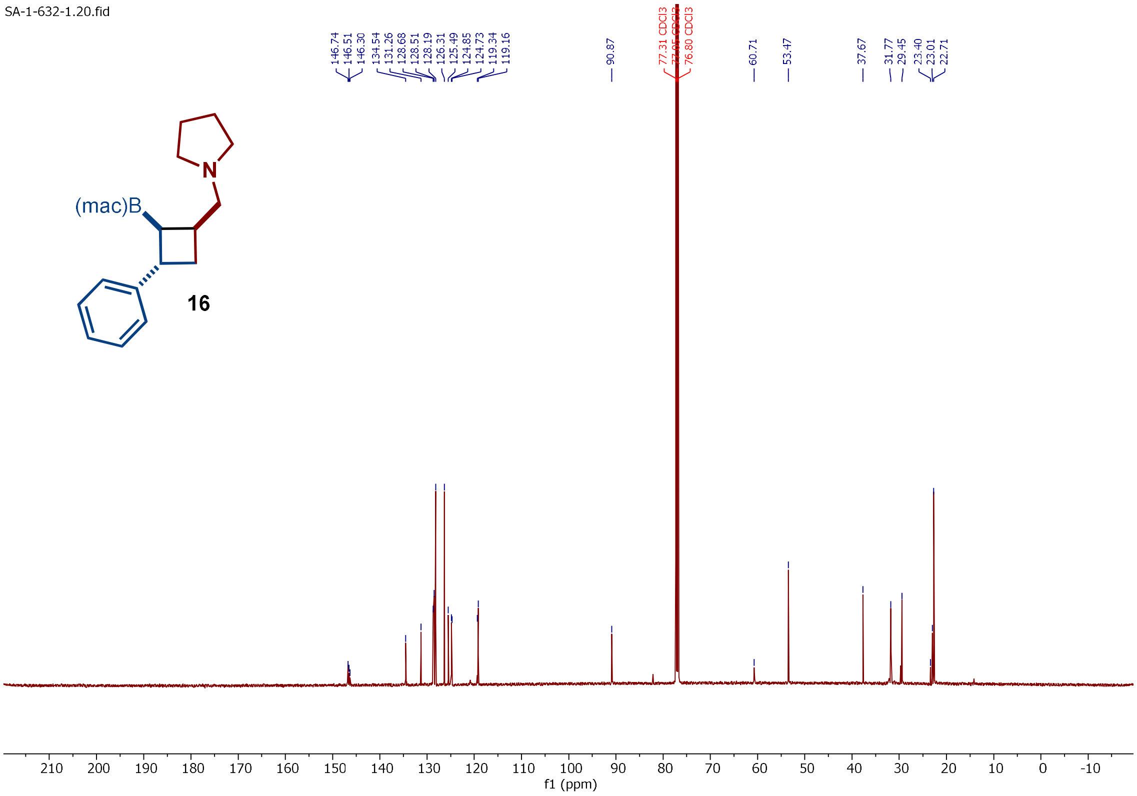

16

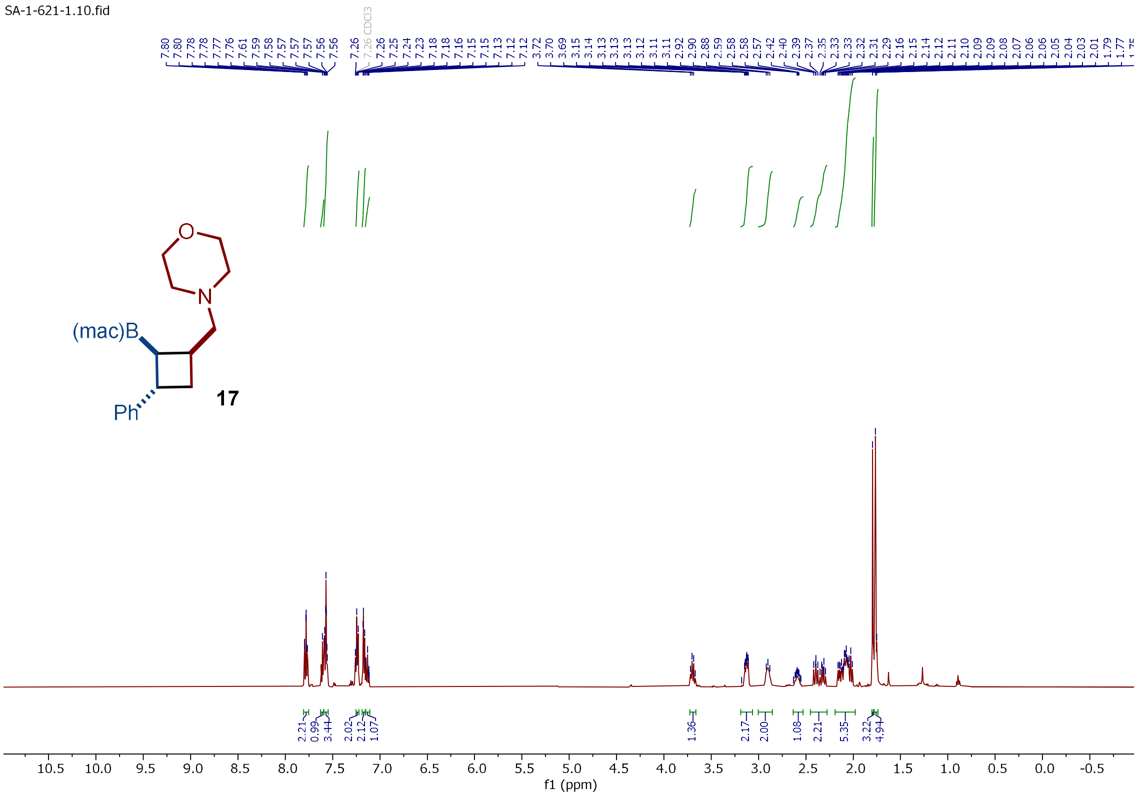
SA-1-621-1.10.fid
(mac)B
Ph
17
f1 (ppm)

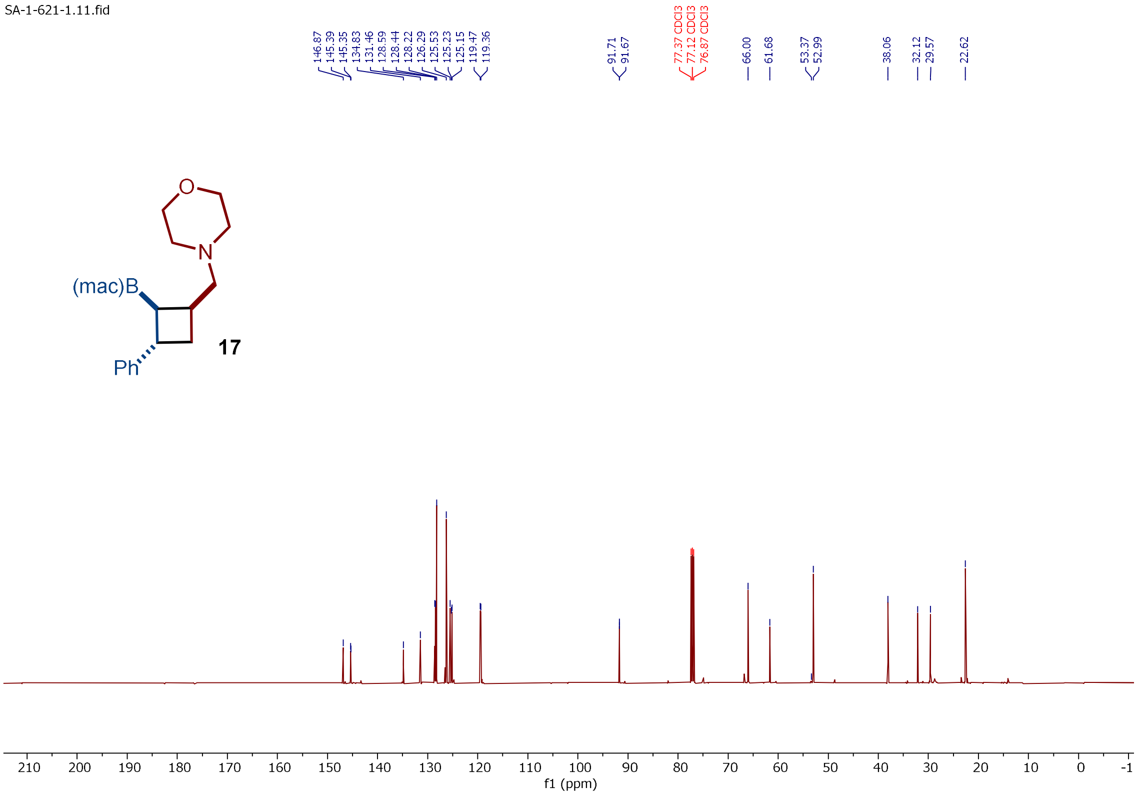
SA-1-621-1.11.fid
146.87
145.39
145.35
134.83
131.46
128.59
128.44
128.22
126.29
125.53
125.23
125.15
119.47
119.36
91.71
91.67
77.37 CDCl3
77.12 CDCl3
76.87 CDCl3
66.00
61.68
53.37
52.99
38.06
32.12
29.57
22.62
(mac)B
Ph
17
f1 (ppm)

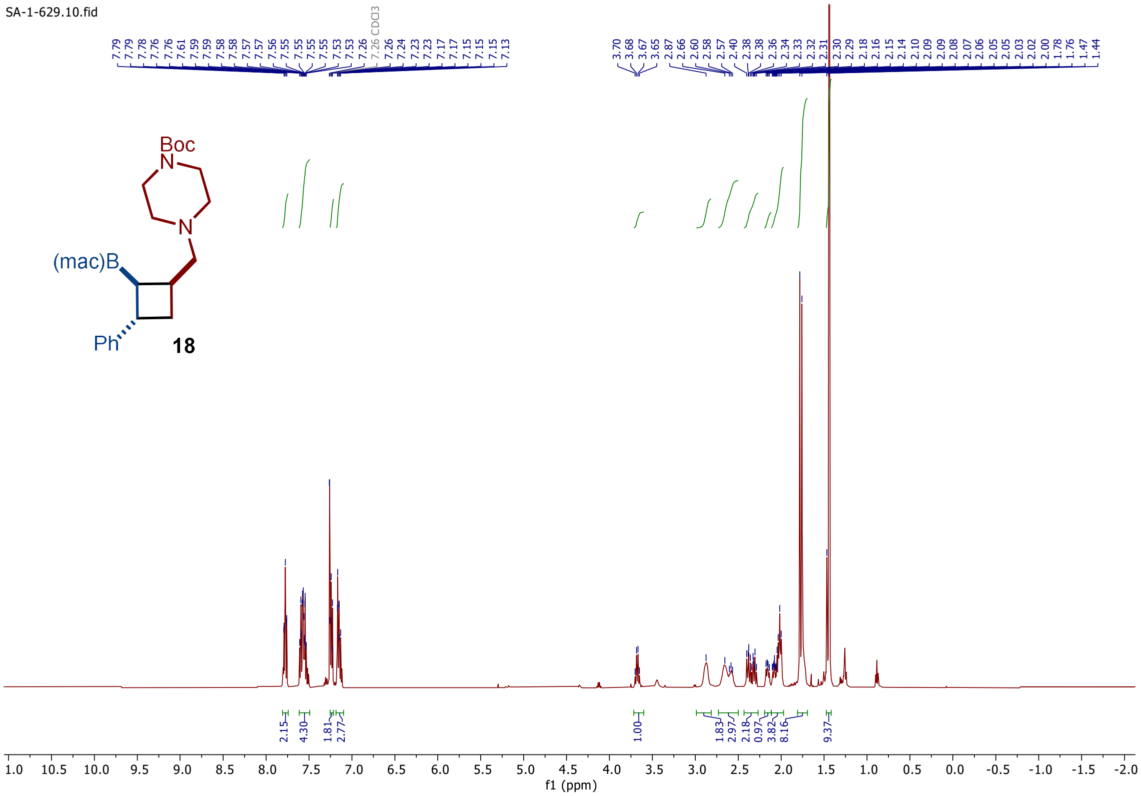
SA-1-629.10.fid
7.79 7.79 7.78 7.76 7.61 7.59 7.59 7.58 7.58 7.57 7.57 7.56 7.55 7.55 7.55 7.55 7.53 7.53 7.26 CDCl3 7.26 7.24 7.23 7.17 7.17 7.15 7.15 7.13
3.70 3.68 3.67 3.65 2.87 2.66 2.60 2.58 2.57 2.40 2.38 2.38 2.36 2.34 2.33 2.32 2.31 2.30 2.29 2.18 2.16 2.15 2.14 2.10 2.09 2.09 2.08 2.07 2.06 2.05 2.05 2.03 2.02 2.00 1.78 1.76 1.47 1.44
Boc
N
N
(mac)B
Ph
18
2.15
4.30
1.81
2.77
1.00
1.83
2.97
2.18
0.97
3.82
8.16
9.37
f1 (ppm)

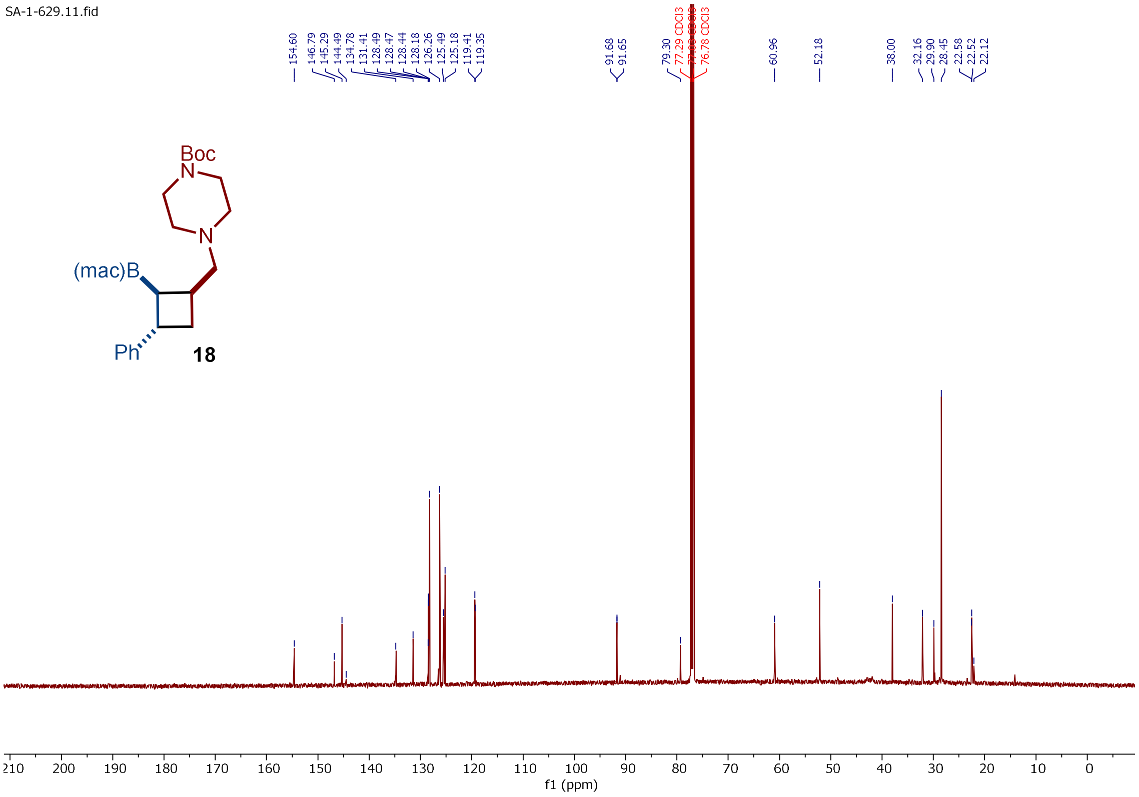
SA-1-629.11.fid
154.60 146.79 145.29 144.49 134.78 131.41 128.49 128.47 128.44 128.18 126.26 125.49 125.18 119.41 119.35
91.68 91.65
79.30 77.29 CDCl3 76.78 CDCl3
60.96
52.18
38.00 32.16 29.90 28.45 22.58 22.52 22.12
Boc
N
N
(mac)B
Ph
18
f1 (ppm)

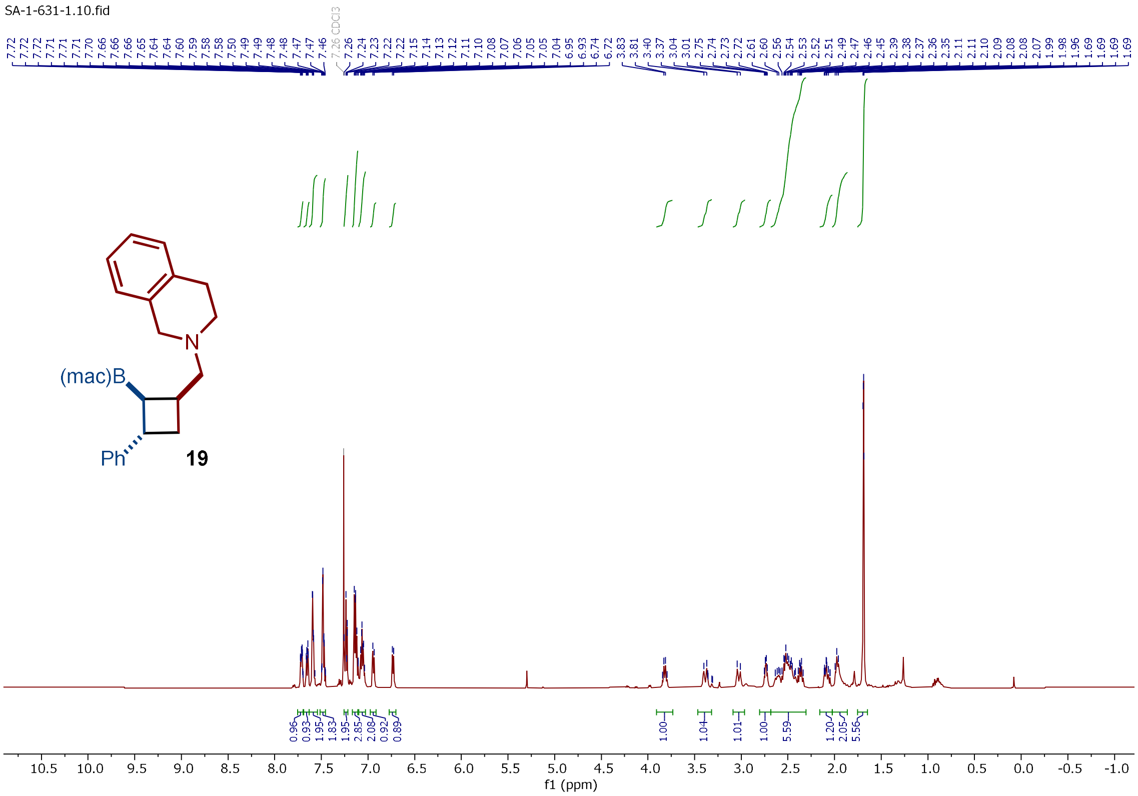
SA-1-631-1.10.fid
(mac)B
N
Ph
19
f1 (ppm)

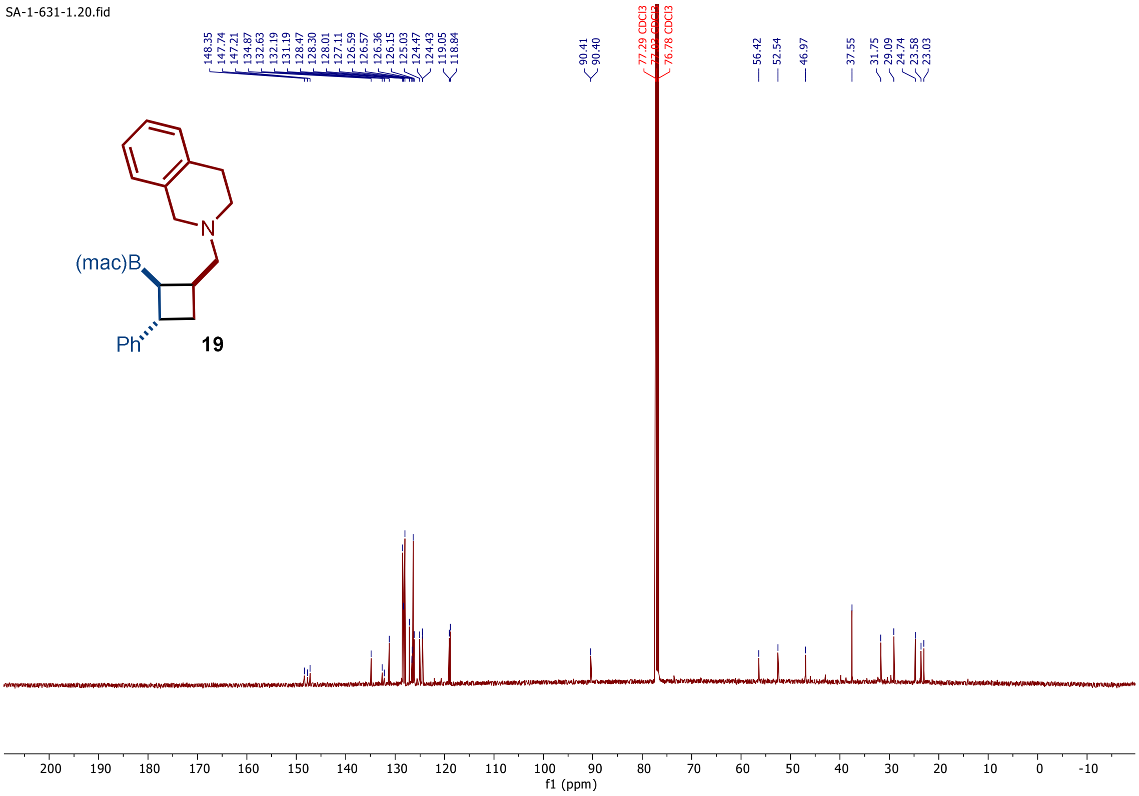
SA-1-631-1.20.fid
148.35
147.74
147.21
134.87
132.63
132.19
131.19
128.47
128.30
128.01
127.11
126.59
126.57
126.36
126.15
125.03
124.47
124.43
119.05
118.84
90.41
90.40
77.29 CDCl3
76.78 CDCl3
56.42
52.54
46.97
37.55
31.75
29.09
24.74
23.58
23.03
(mac)B
N
Ph
19
f1 (ppm)

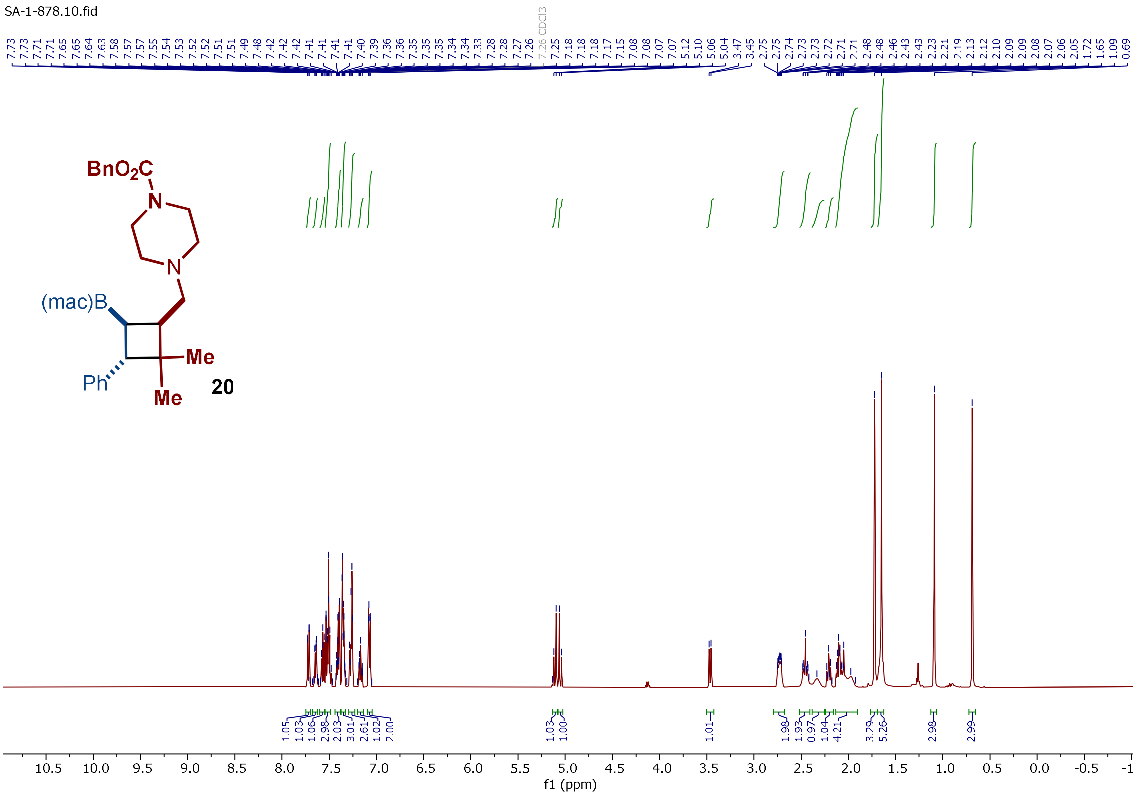

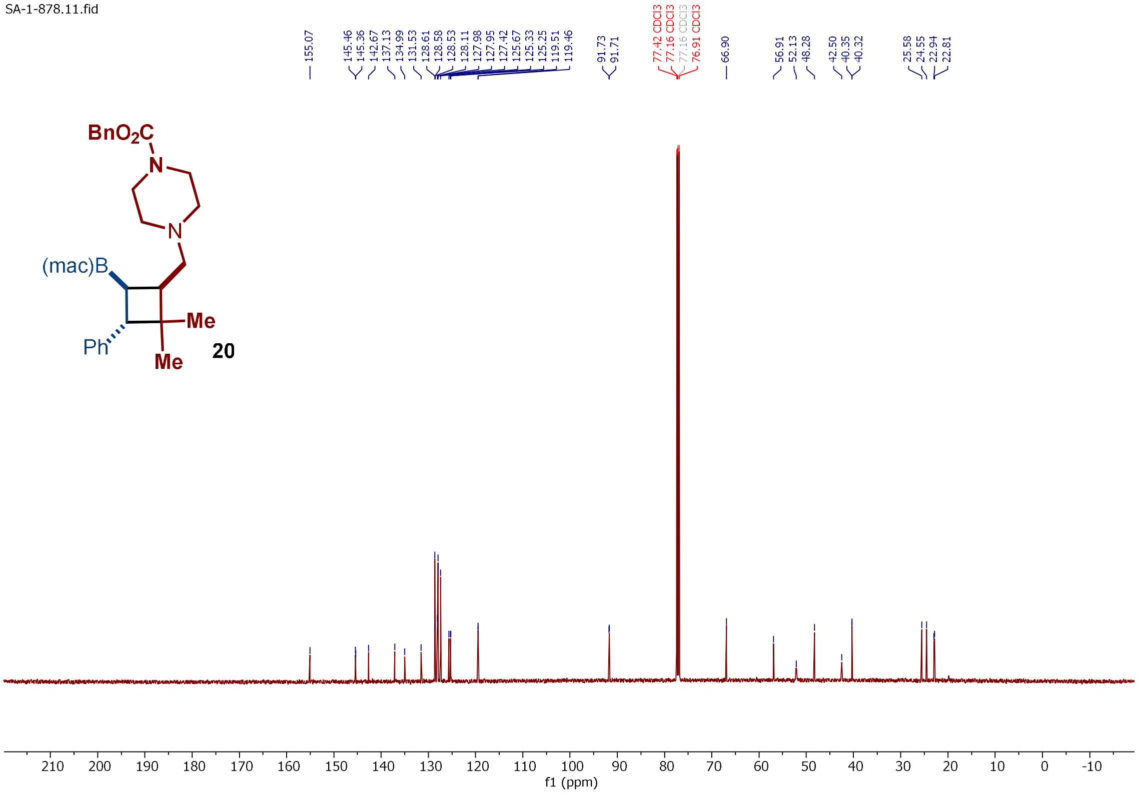

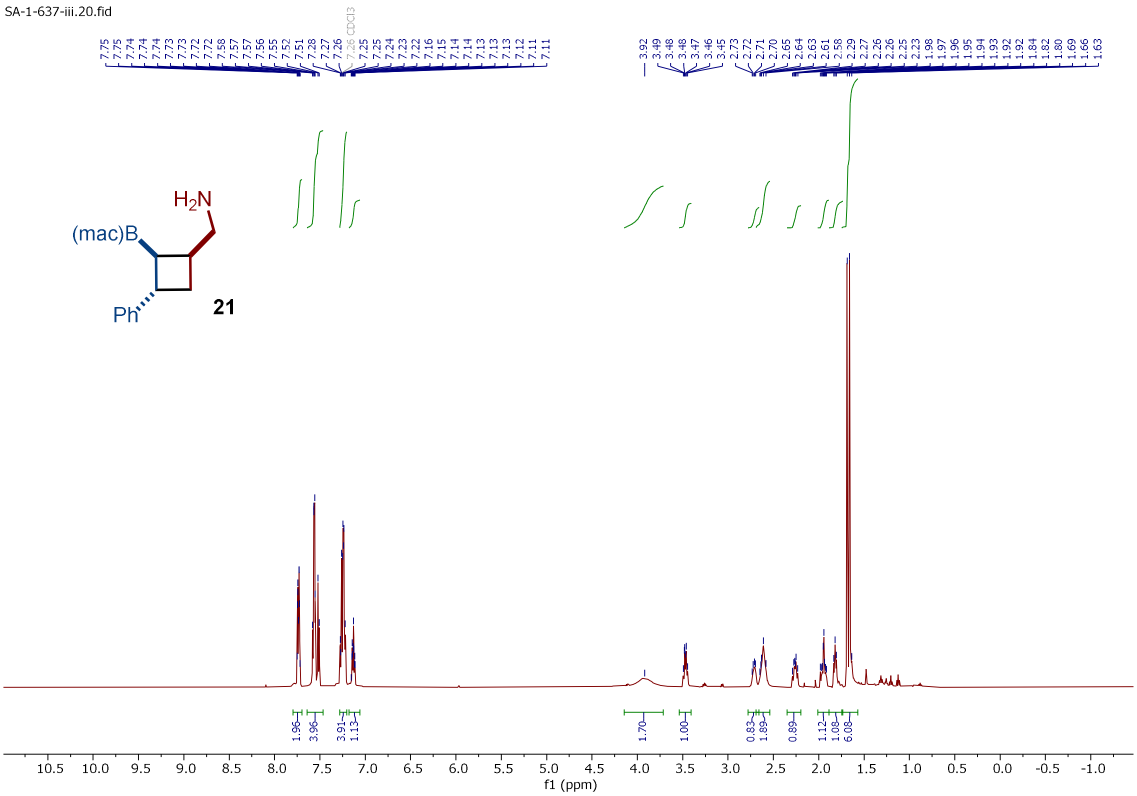
SA-1-637-iii.20.fid
7.75 7.75 7.74 7.74 7.73 7.73 7.72 7.72 7.58 7.57 7.57 7.56 7.55 7.52 7.51 7.28 7.27 7.26 7.26 CDCl3 7.25 7.25 7.24 7.23 7.22 7.16 7.15 7.14 7.14 7.13 7.13 7.12 7.11 7.11
3.92 3.49 3.48 3.48 3.47 3.46 3.45 2.73 2.72 2.71 2.70 2.65 2.64 2.63 2.61 2.58 2.29 2.27 2.26 2.26 2.25 2.23 1.98 1.97 1.96 1.95 1.94 1.93 1.92 1.92 1.84 1.82 1.80 1.69 1.66 1.63
H2N
(mac)B
Ph
21
1.96 3.96 3.91 1.13 1.70 1.00 0.83 1.89 0.89 1.12 1.08 6.08
10.5 10.0 9.5 9.0 8.5 8.0 7.5 7.0 6.5 6.0 5.5 5.0 4.5 4.0 3.5 3.0 2.5 2.0 1.5 1.0 0.5 0.0 -0.5 -1.0
f1 (ppm)

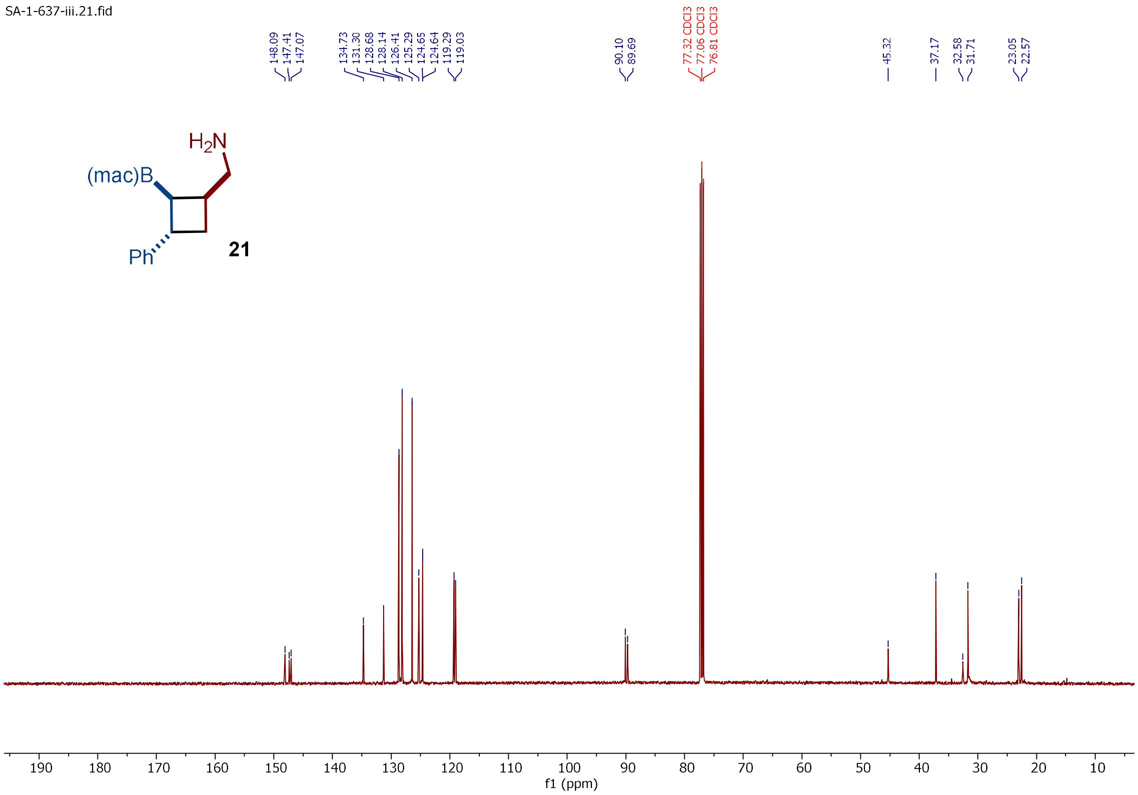
SA-1-637-iii.21.fid
148.09 147.41 147.07
134.73 131.30 128.68 128.14 126.41 125.29 124.65 124.64 119.29 119.03
90.10 89.69
77.32 CDCl3 77.06 CDCl3 76.81 CDCl3
45.32
37.17 32.58 31.71
23.05 22.57
H2N
(mac)B
Ph
21
190 180 170 160 150 140 130 120 110 100 90 80 70 60 50 40 30 20 10
f1 (ppm)

SA-1-641-ii.10.fid

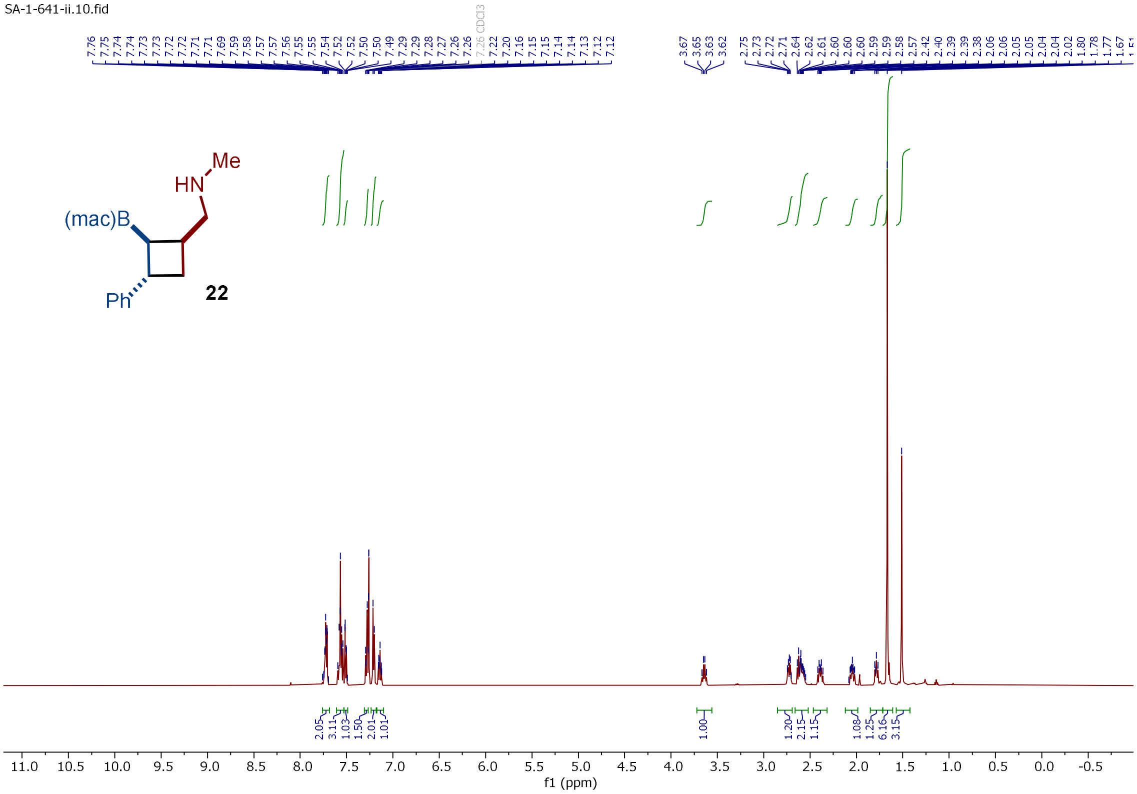

SA-1-641-ii.11.fid

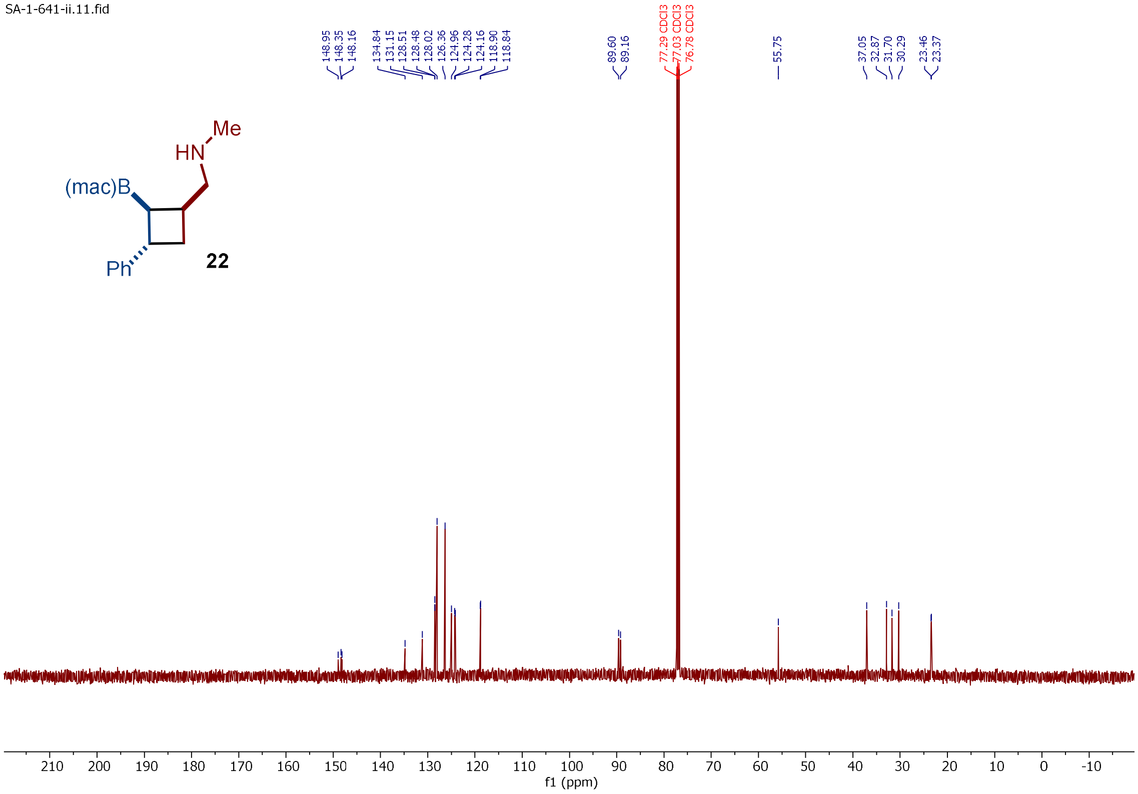

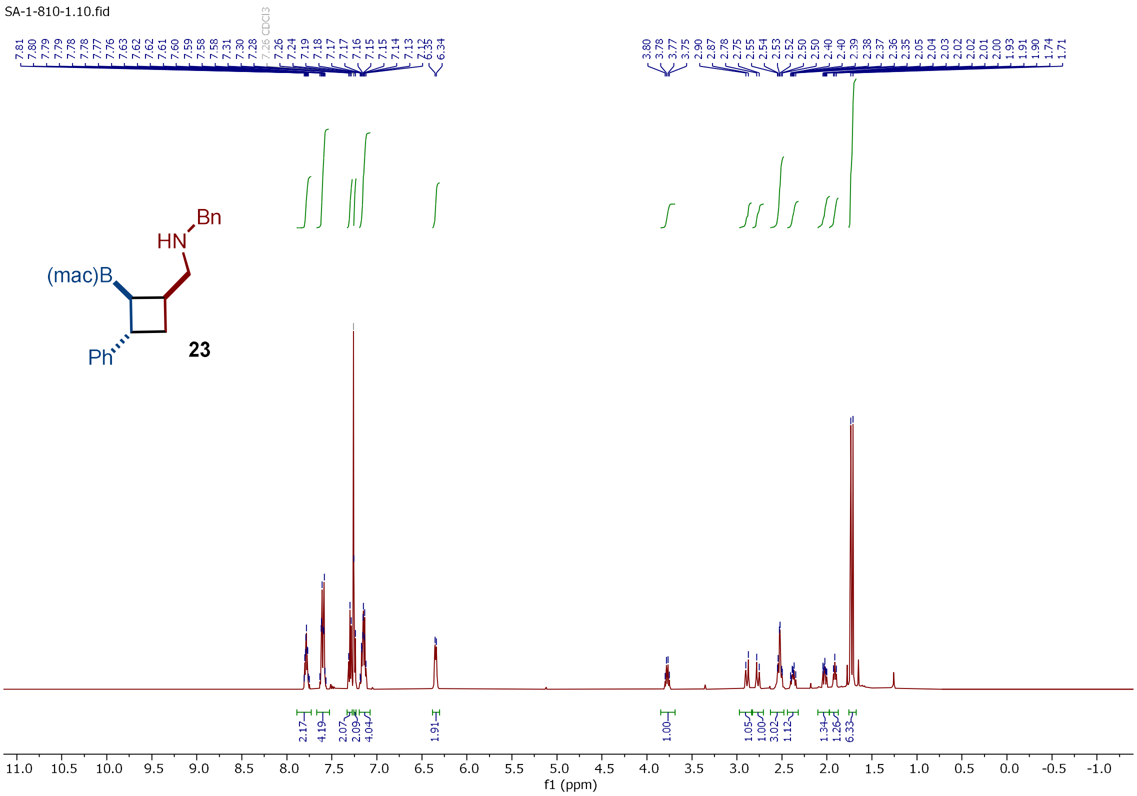
SA-1-810-1.10.fid
(mac)B
HN
Bn
Ph
23
f1 (ppm)

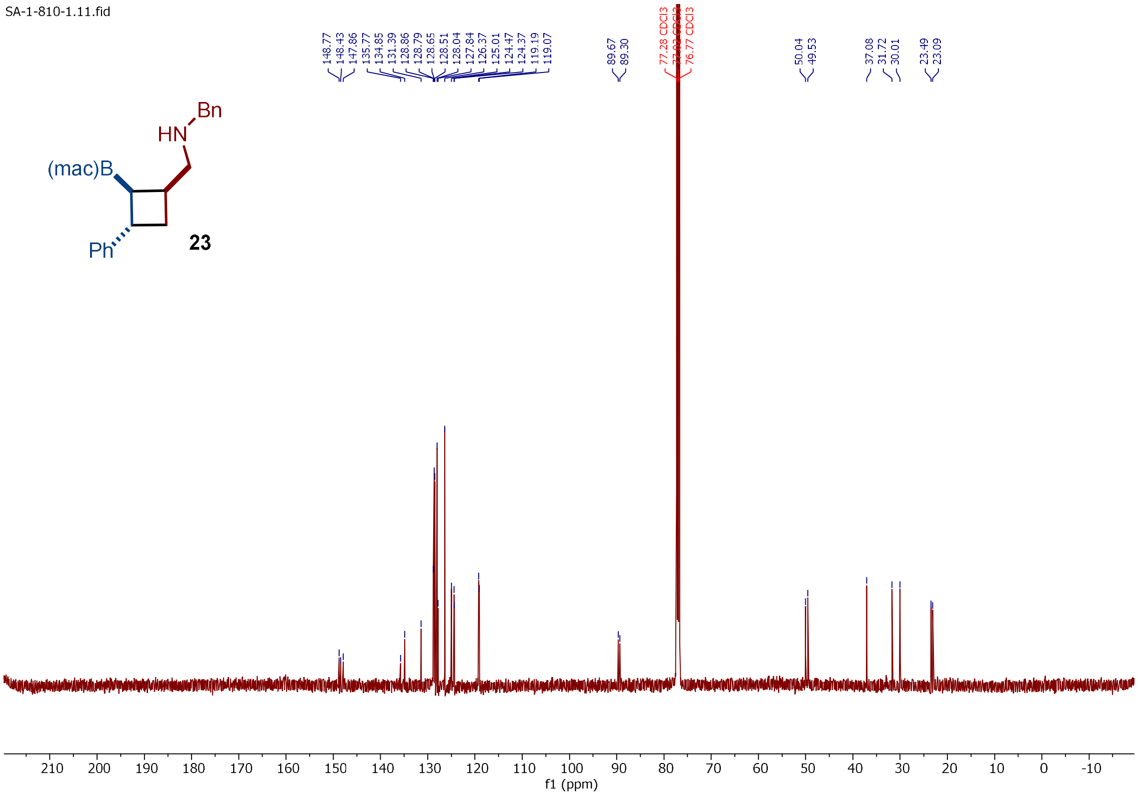
SA-1-810-1.11.fid
(mac)B
HN
Bn
Ph
23
f1 (ppm)

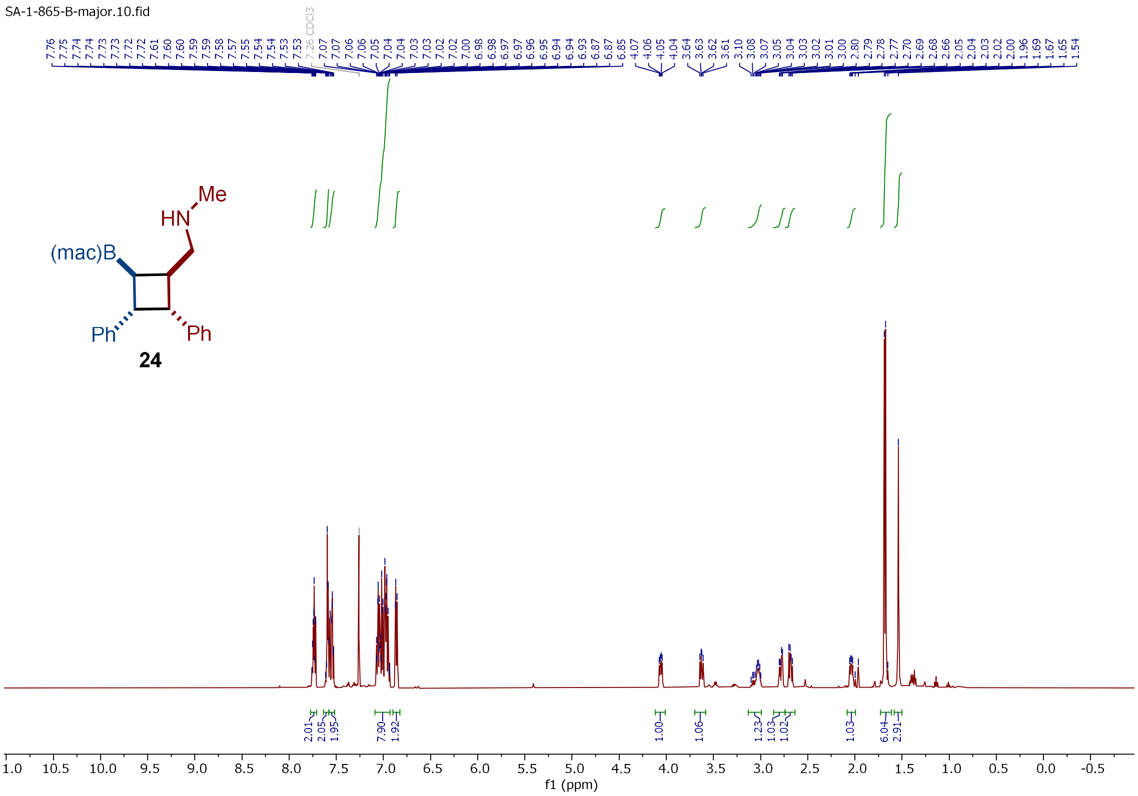
SA-1-865-B-major.10.fid
(mac)B
HN
Me
Ph
Ph
24
f1 (ppm)

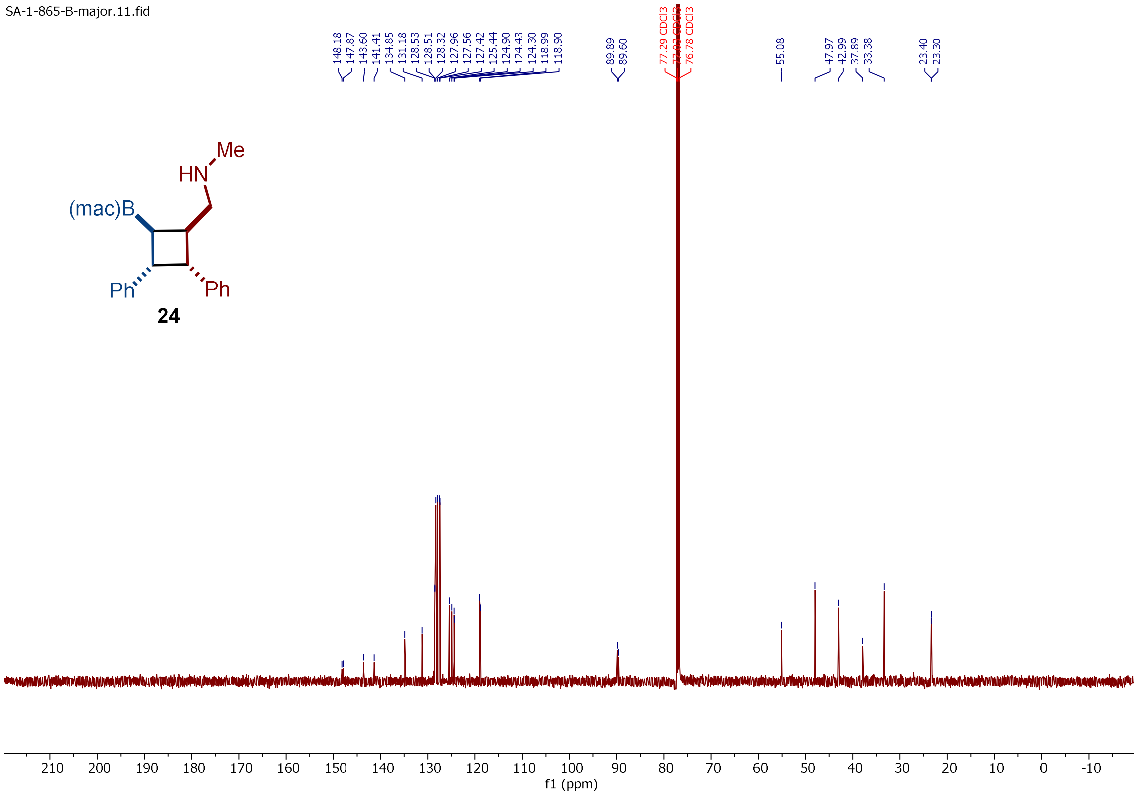
SA-1-865-B-major.11.fid
(mac)B
HN
Me
Ph
Ph
24
f1 (ppm)

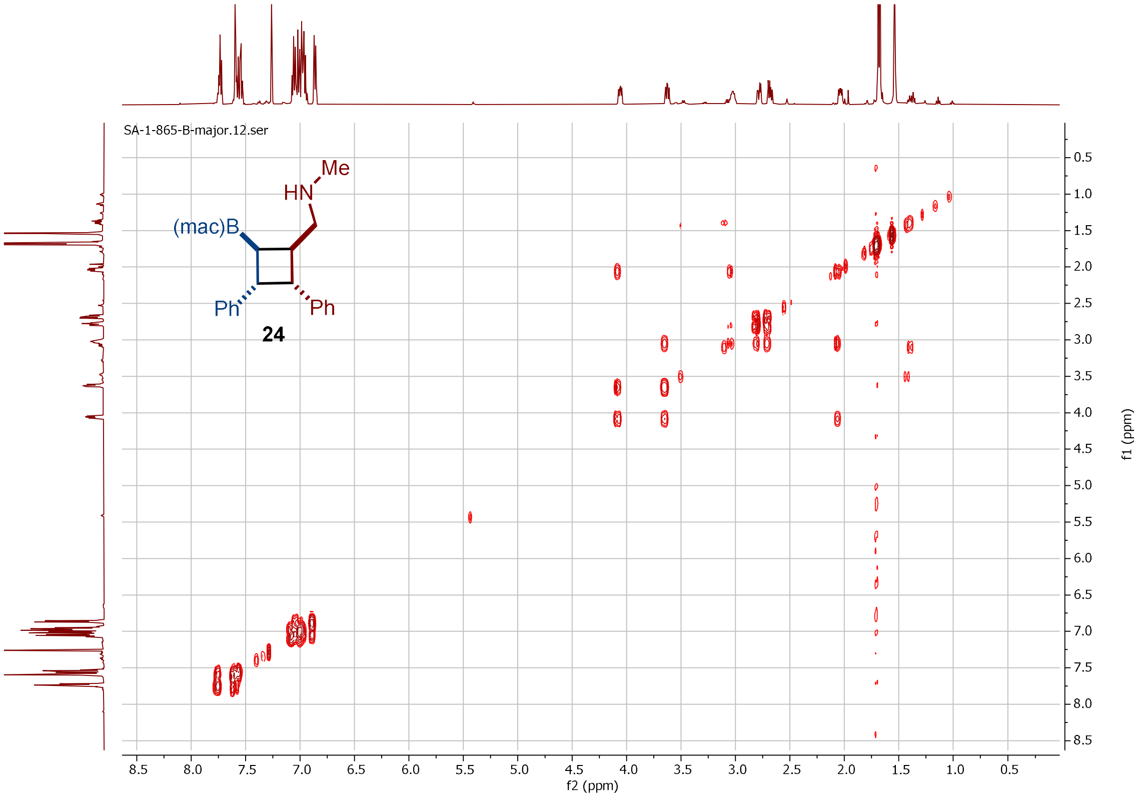
SA-1-865-B-major.12.ser
Me
HN
(mac)B
Ph
Ph
24
f2 (ppm)
f1 (ppm)

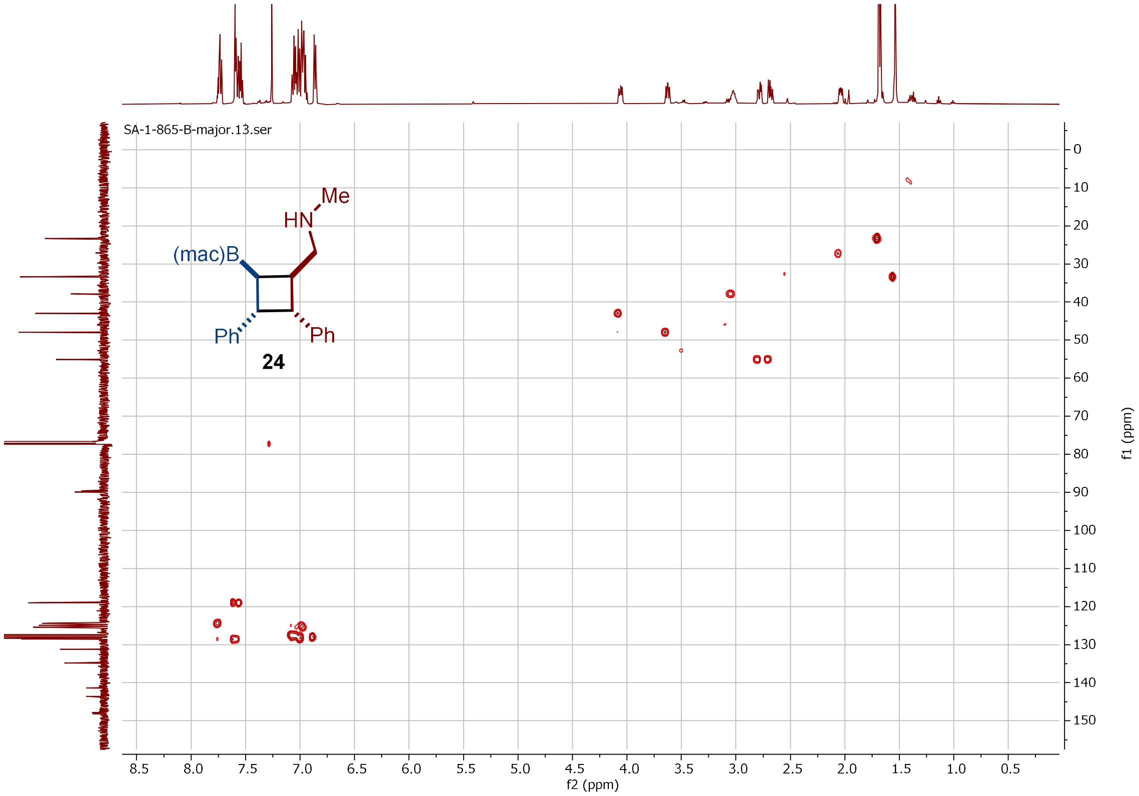
SA-1-865-B-major.13.ser
Me
HN
(mac)B
Ph
Ph
24
f2 (ppm)
f1 (ppm)

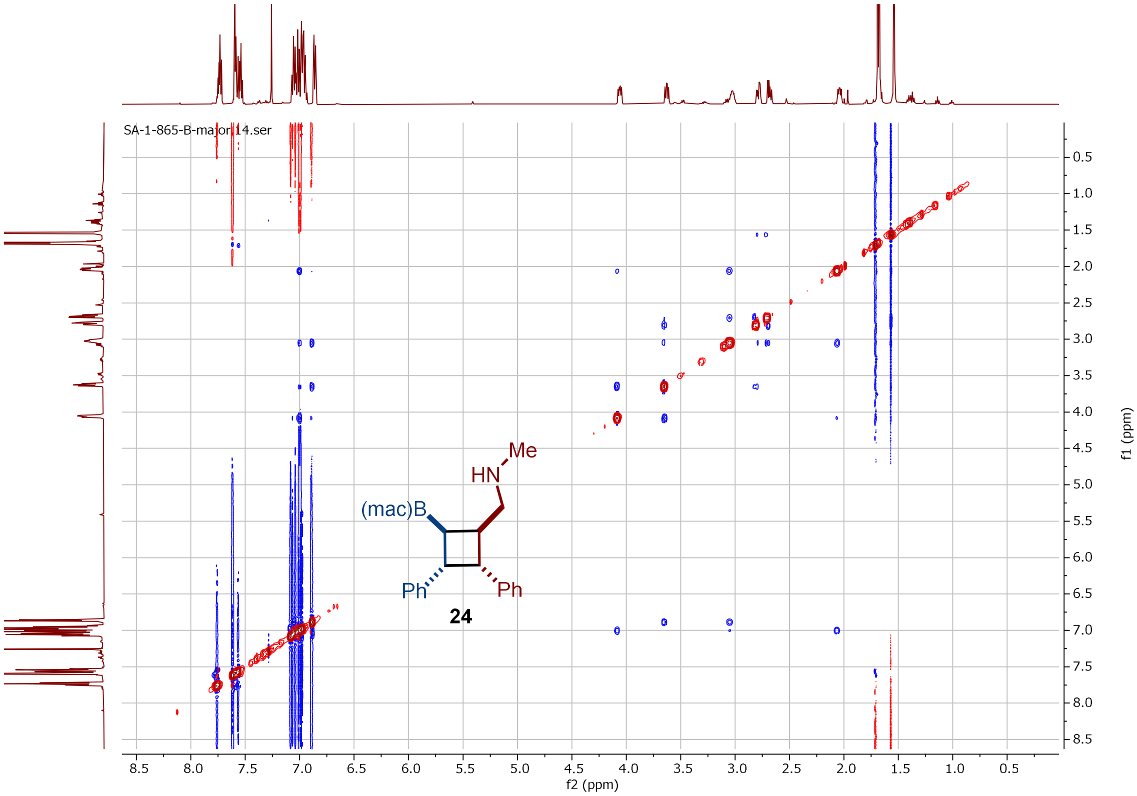

SA-1-865-B-major14.ser
(mac)B
HN
Me
Ph
Ph
24
f2 (ppm)
f1 (ppm)

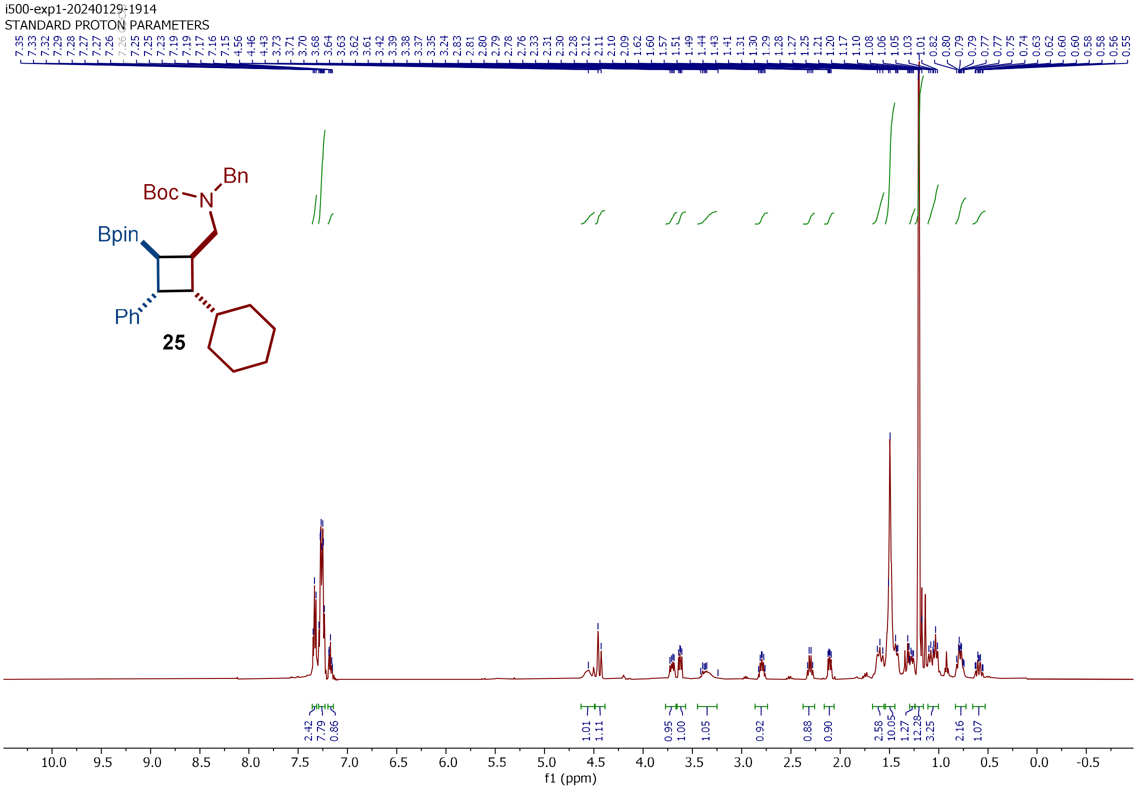

i500-exp1-20240129-1914
STANDARD PROTON PARAMETERS
Boc
N
Bn
Bpin
Ph
25
f1 (ppm)

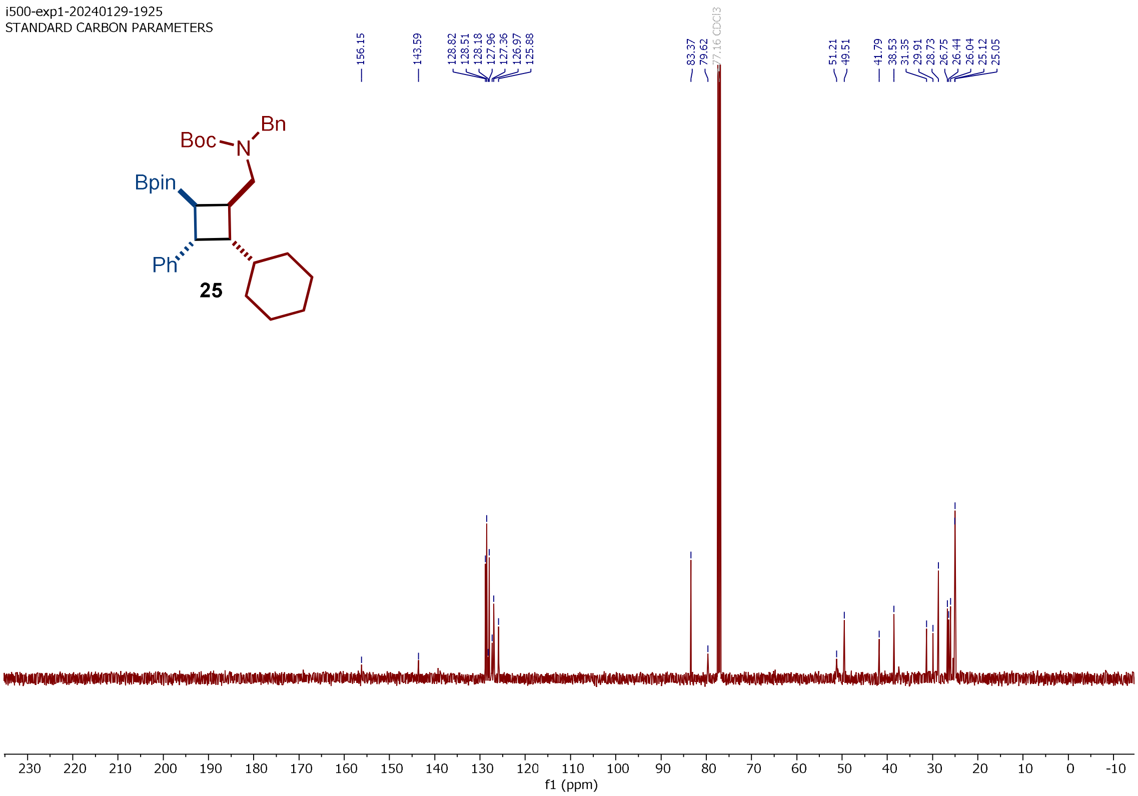
i500-exp1-20240129-1925
STANDARD CARBON PARAMETERS
156.15
143.59
128.82
128.51
128.18
127.96
127.36
126.97
125.88
83.37
79.62
77.16 CDCl3
51.21
49.51
41.79
38.53
31.35
29.91
28.73
26.75
26.44
26.04
25.12
25.05
Boc
N
Bn
Bpin
Ph
25
f1 (ppm)

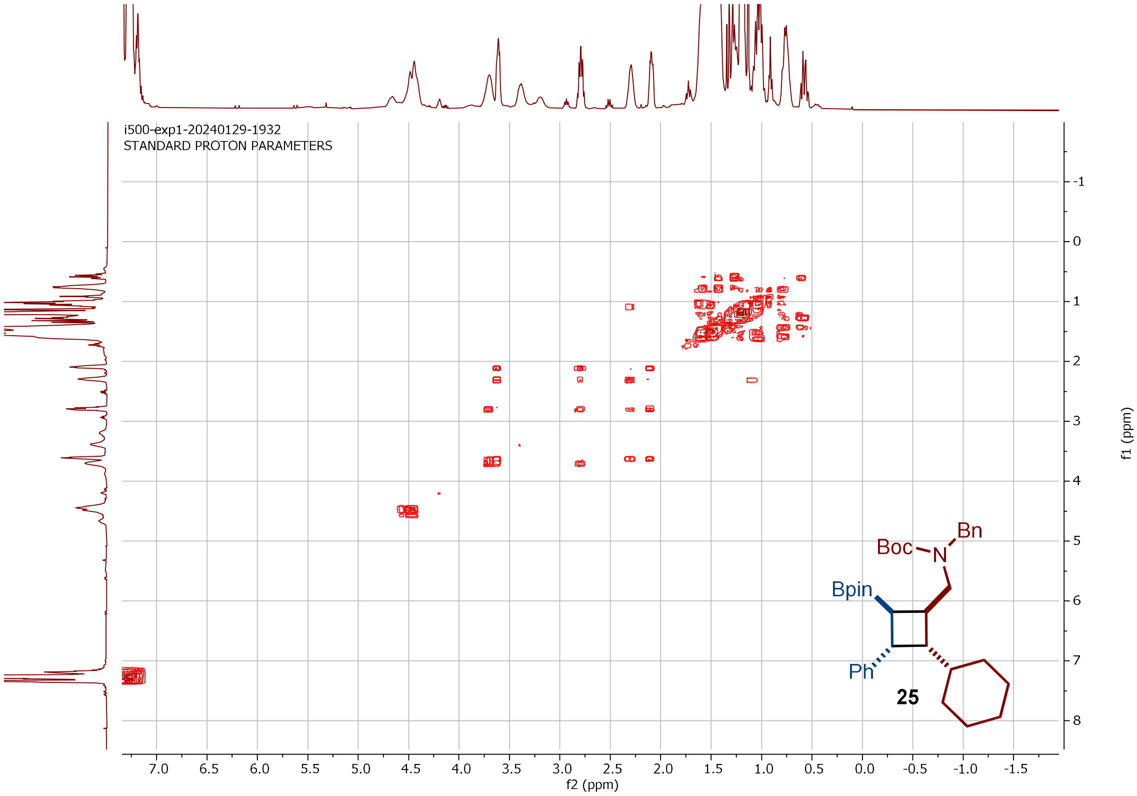
i500-exp1-20240129-1932
STANDARD PROTON PARAMETERS
Boc
N
Bn
Bpin
Ph
25
f2 (ppm)
f1 (ppm)

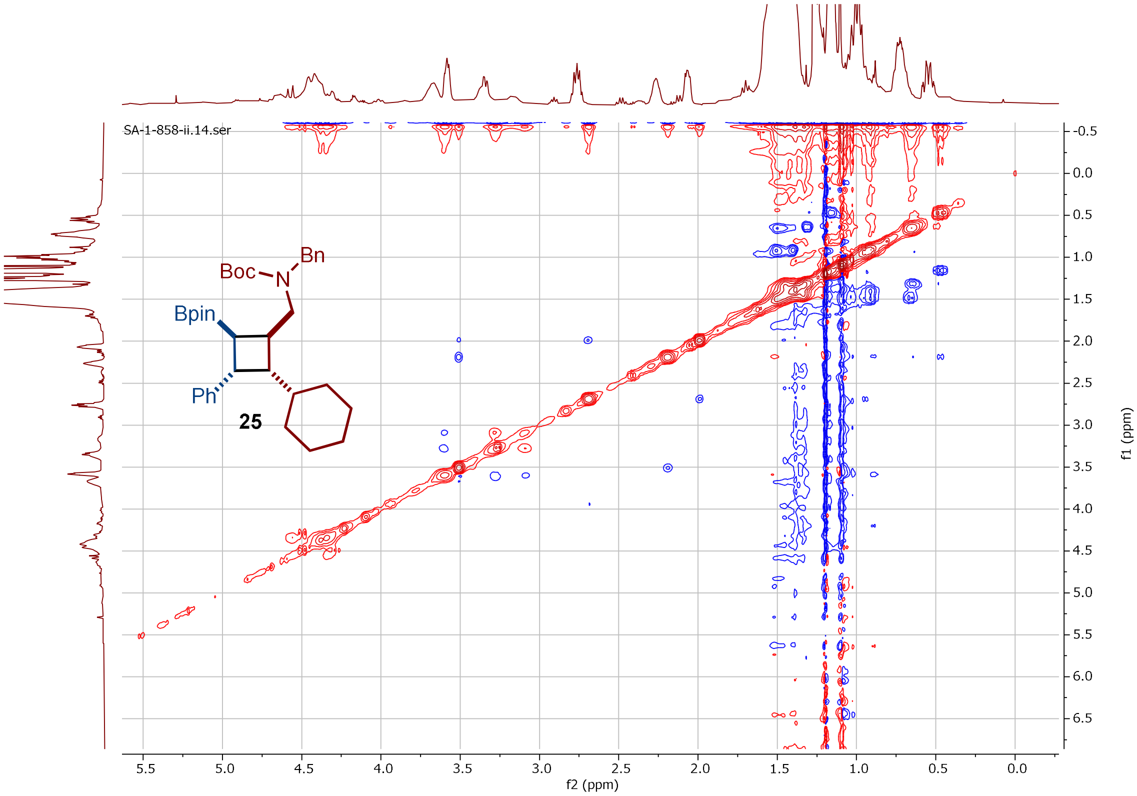
SA-1-858-ii.14.ser
Boc
N
Bn
Bpin
Ph
25
f2 (ppm)
f1 (ppm)

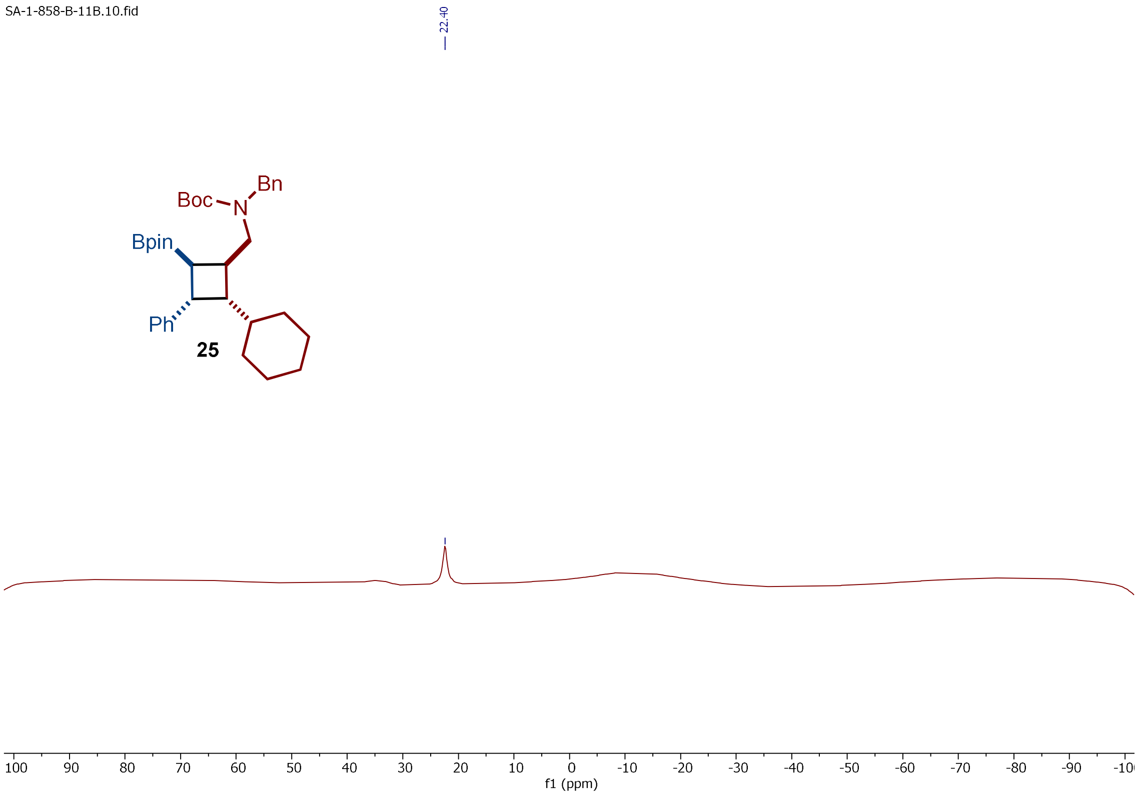
SA-1-858-B-11B.10.fid
22.40
Boc
N
Bn
Bpin
Ph
25
f1 (ppm)

SA-1-670-FINAL.10.fid

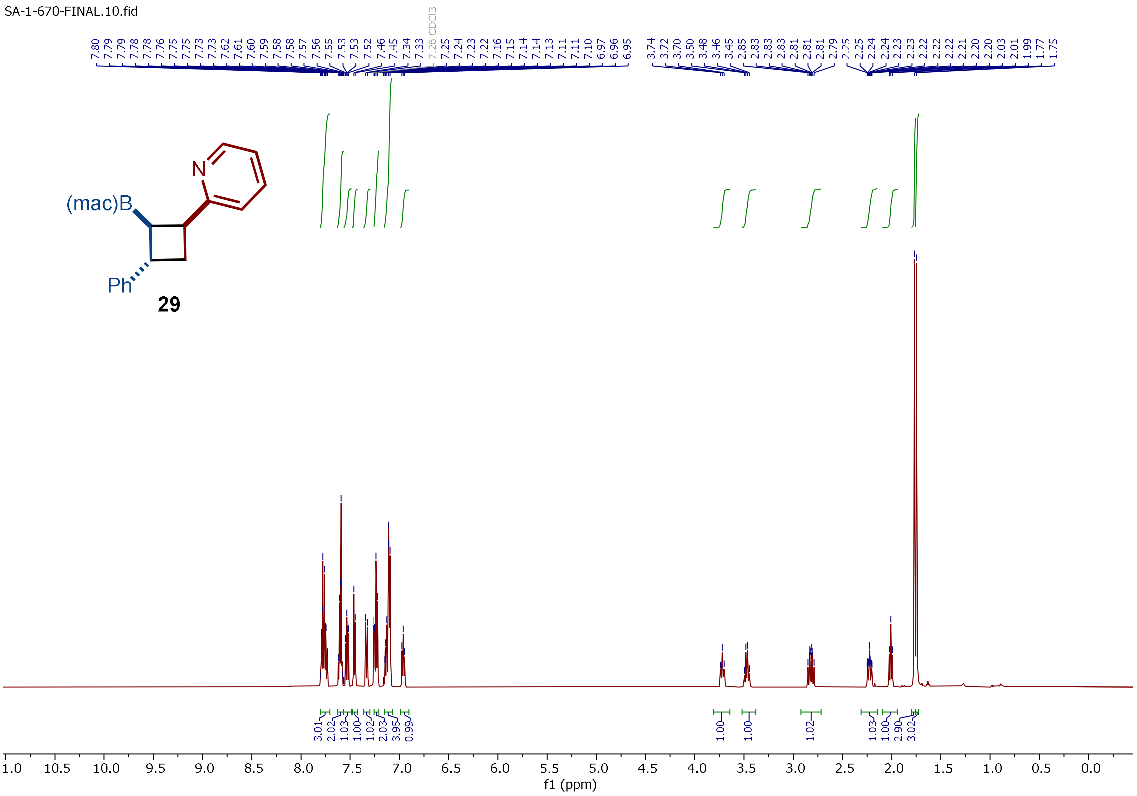

SA-1-670-FINAL.11.fid

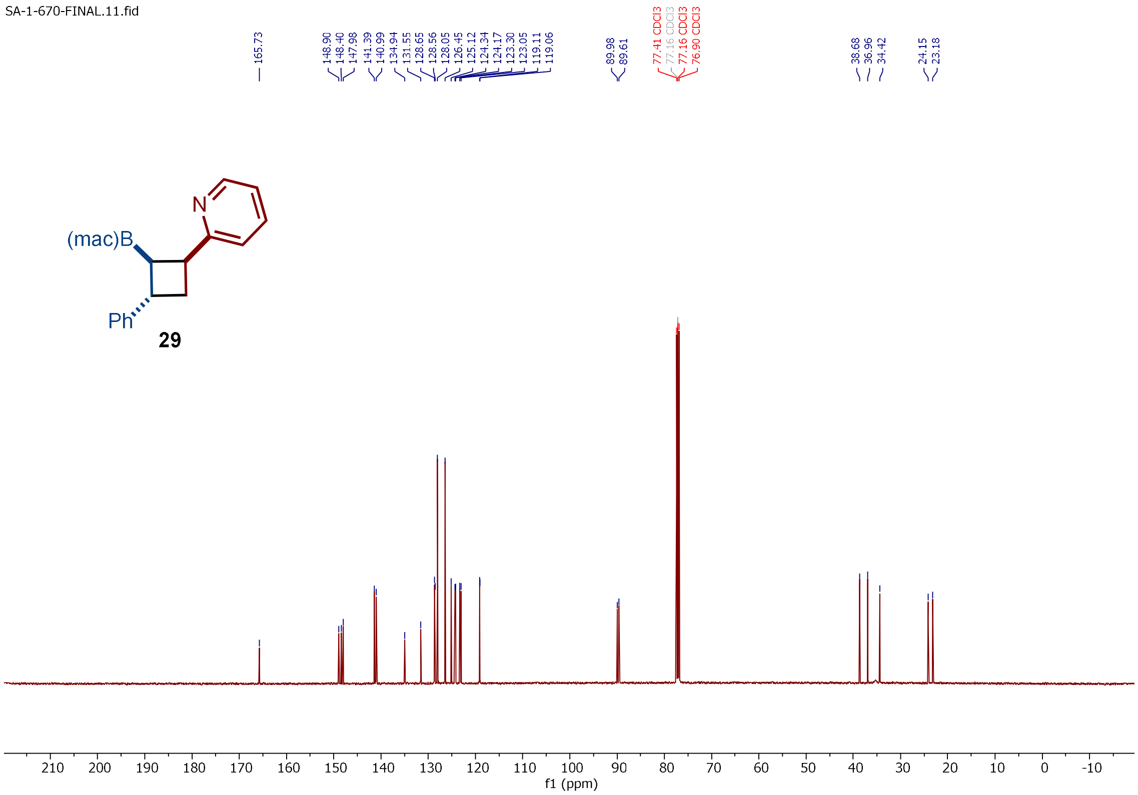

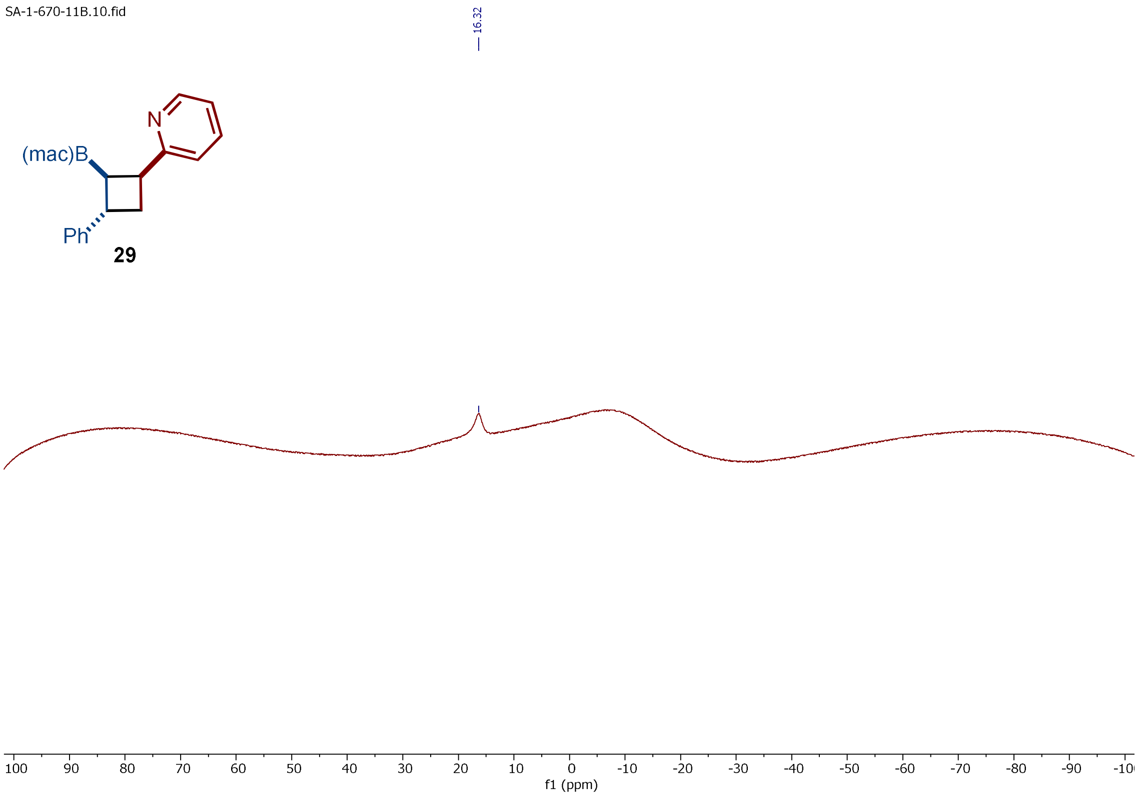
SA-1-670-11B.10.fid
16.32
(mac)B
N
Ph
29
f1 (ppm)

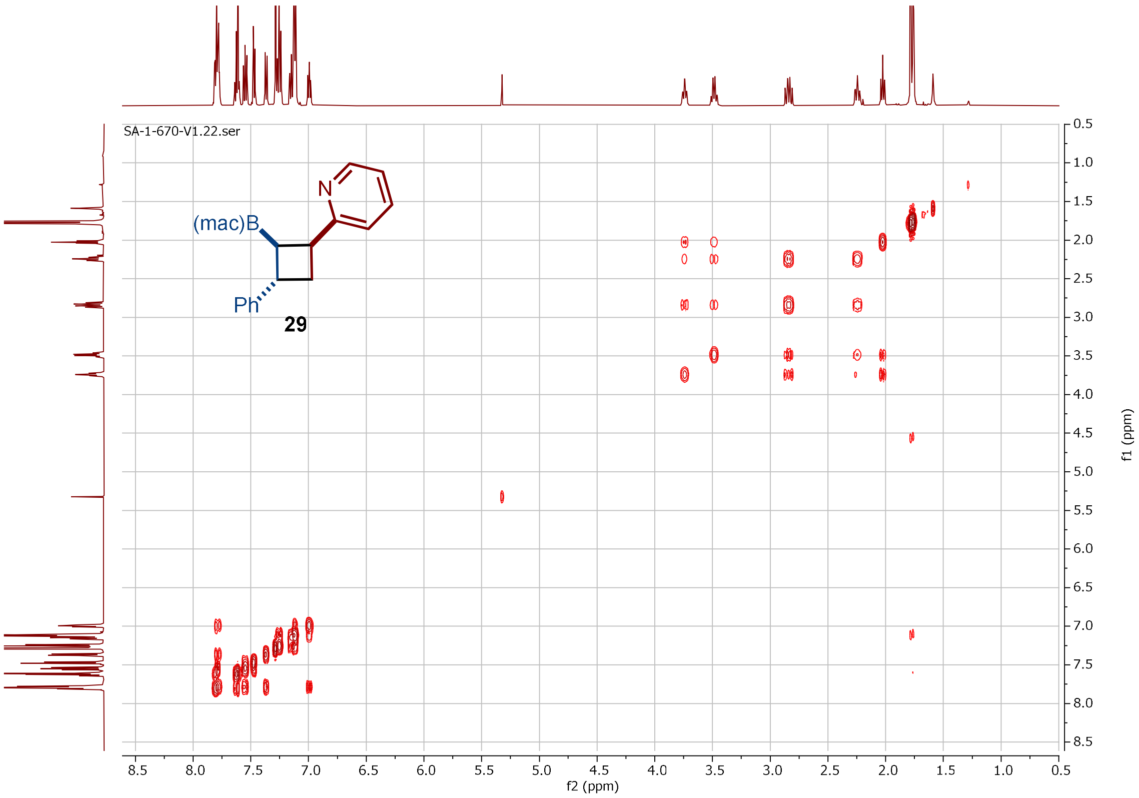
SA-1-670-V1.22.ser
(mac)B
N
Ph
29
f2 (ppm)
f1 (ppm)

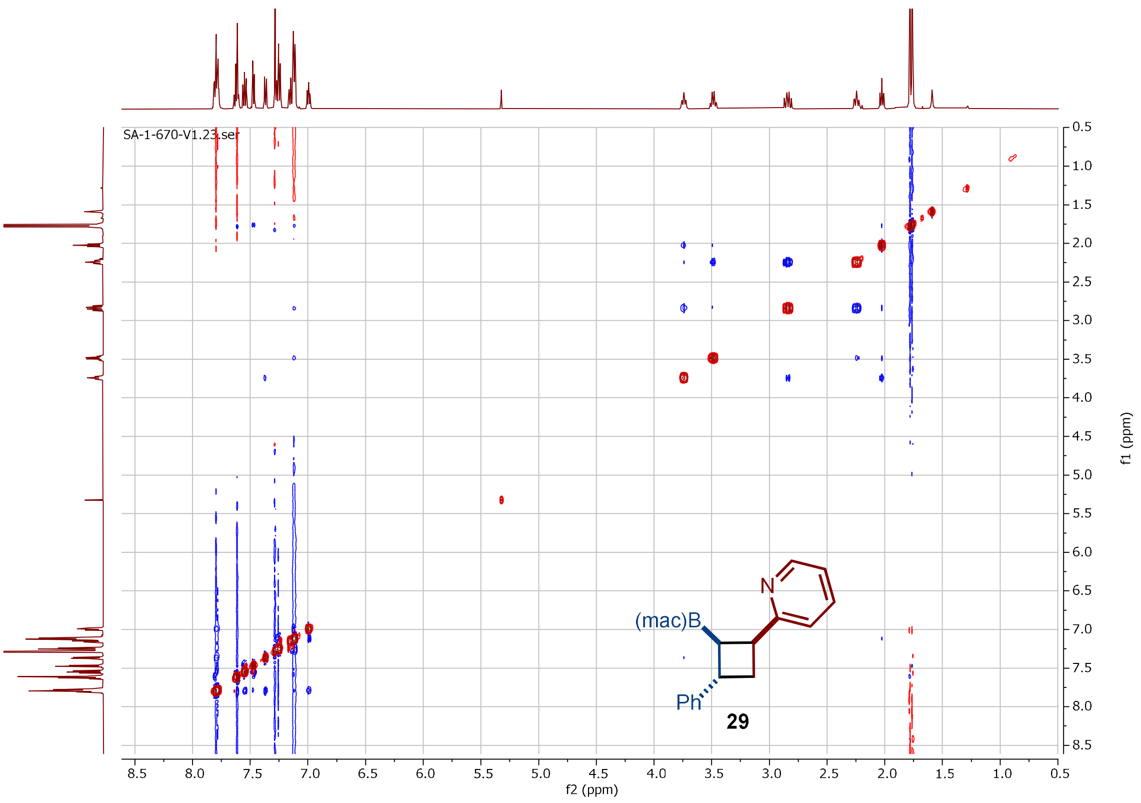
SA-1-670-V1.23.ser
(mac)B
N
Ph
29
f2 (ppm)
f1 (ppm)

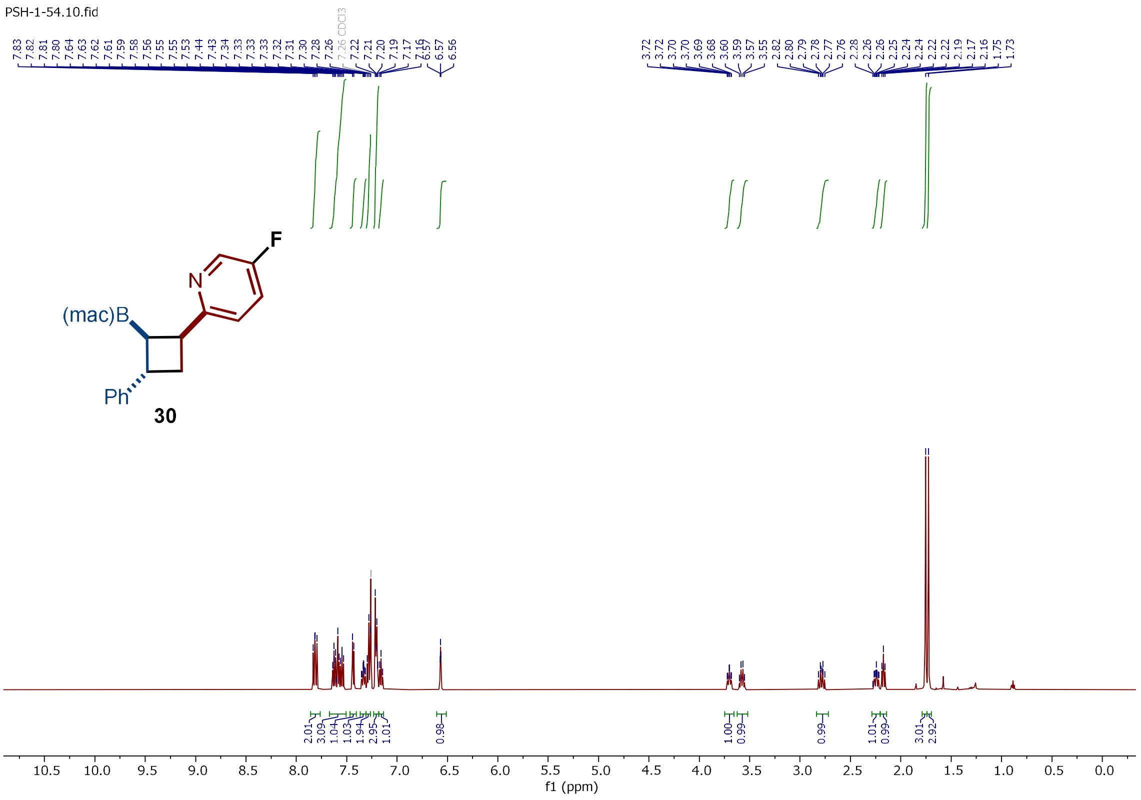
PSH-1-54.10.fid
(mac)B
N
F
Ph
30
f1 (ppm)

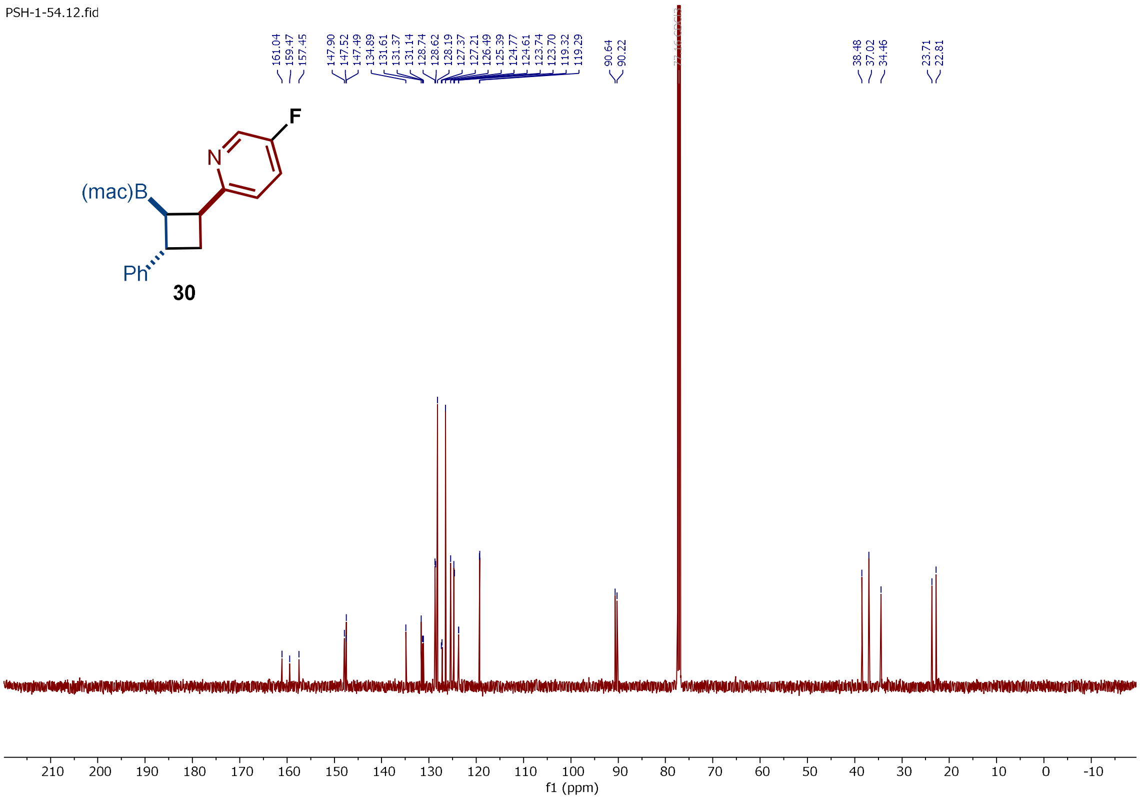

PSH-1-54.12.fid
161.04
159.47
157.45
147.90
147.52
147.49
134.89
131.61
131.37
131.14
128.74
128.62
128.19
127.37
127.21
126.49
125.39
124.77
124.61
123.74
123.70
119.32
119.29
90.64
90.22
38.48
37.02
34.46
23.71
22.81
F
N
(mac)B
Ph
30
210 200 190 180 170 160 150 140 130 120 110 100 90 80 70 60 50 40 30 20 10 0 -10
f1 (ppm)

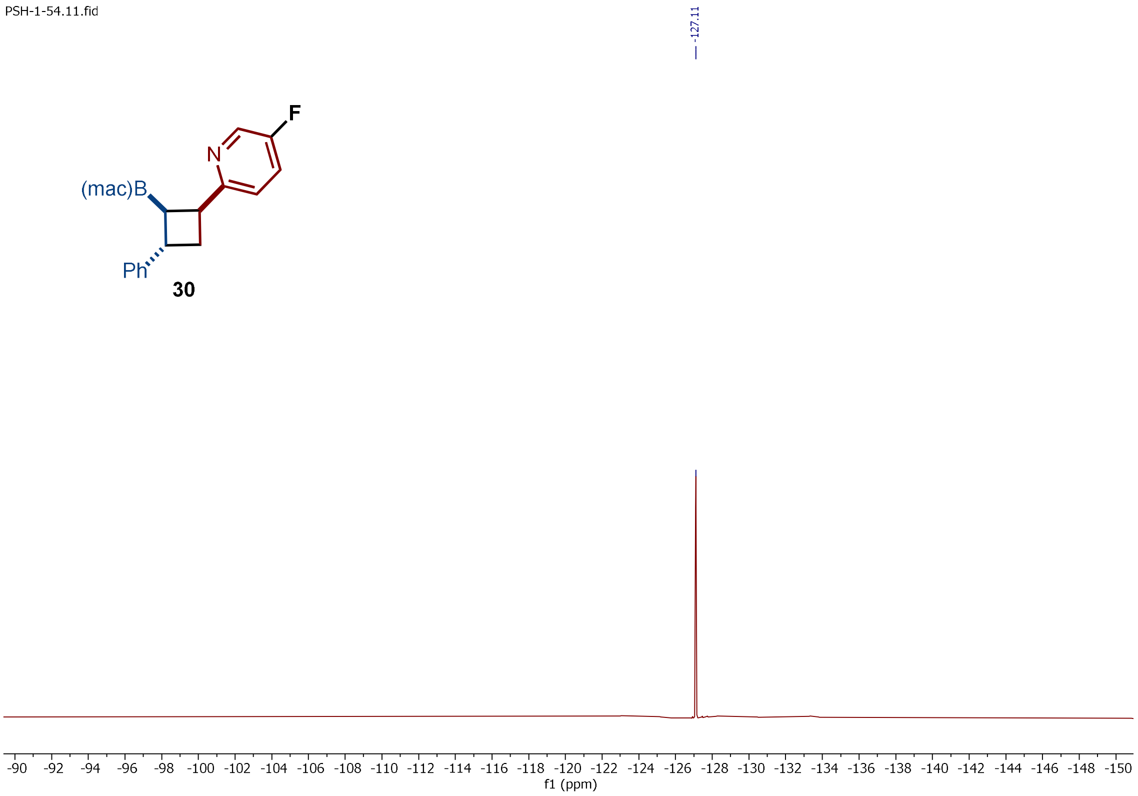

PSH-1-54.11.fid
-127.11
F
N
(mac)B
Ph
30
-90 -92 -94 -96 -98 -100 -102 -104 -106 -108 -110 -112 -114 -116 -118 -120 -122 -124 -126 -128 -130 -132 -134 -136 -138 -140 -142 -144 -146 -148 -150
f1 (ppm)

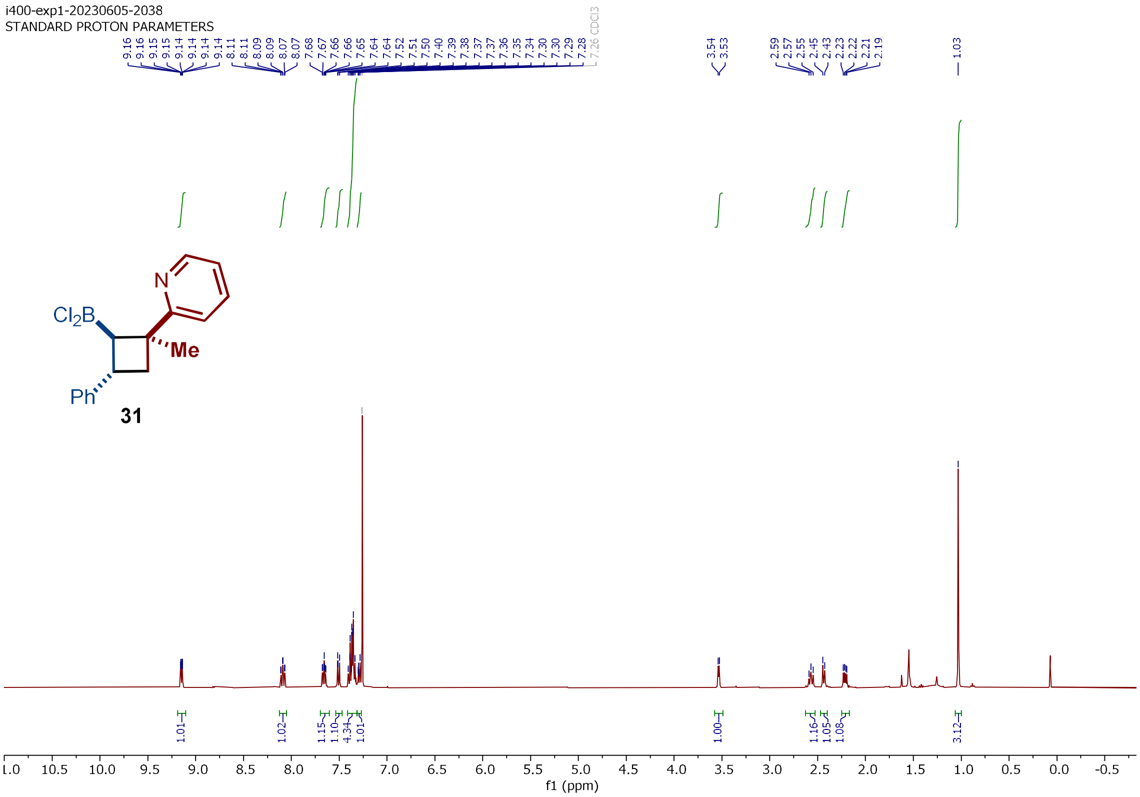
i400-exp1-20230605-2038
STANDARD PROTON PARAMETERS
9.16 9.16 9.15 9.15 9.14 9.14 9.14 9.14 9.14 8.11 8.11 8.09 8.09 8.07 8.07 7.68 7.67 7.66 7.65 7.65 7.64 7.52 7.51 7.50 7.40 7.39 7.38 7.37 7.37 7.36 7.35 7.34 7.30 7.30 7.29 7.28 7.26 CDCl3
3.54 3.53
2.59 2.57 2.55 2.45 2.43 2.23 2.22 2.21 2.19
1.03
N
Cl2B
Me
Ph
31
1.01 1.02 1.15 1.10 4.34 1.01 1.00 1.16 1.05 1.08 3.12
f1 (ppm)

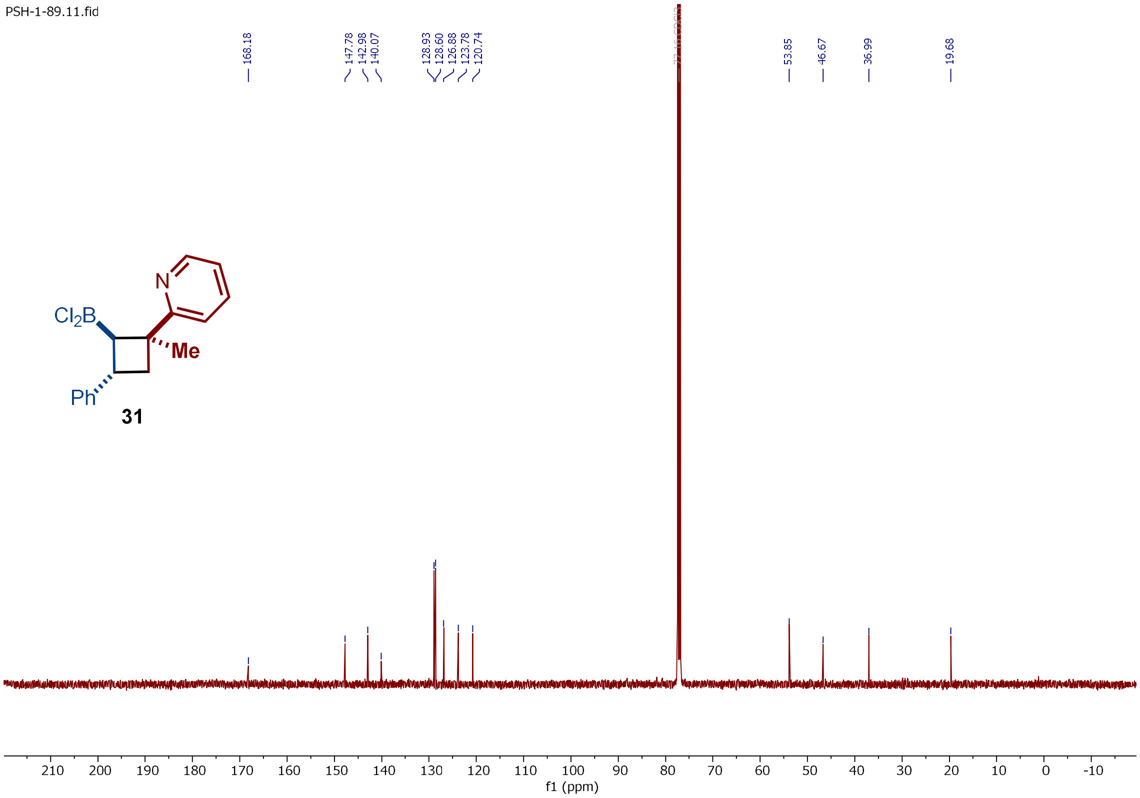
PSH-1-89.11.fid
168.18
147.78 142.98 140.07
128.93 128.60 126.88 123.78 120.74
53.85
46.67
36.99
19.68
N
Cl2B
Me
Ph
31
f1 (ppm)

i400-exp1-20230503-2017
STANDARD PROTON PARAMETERS

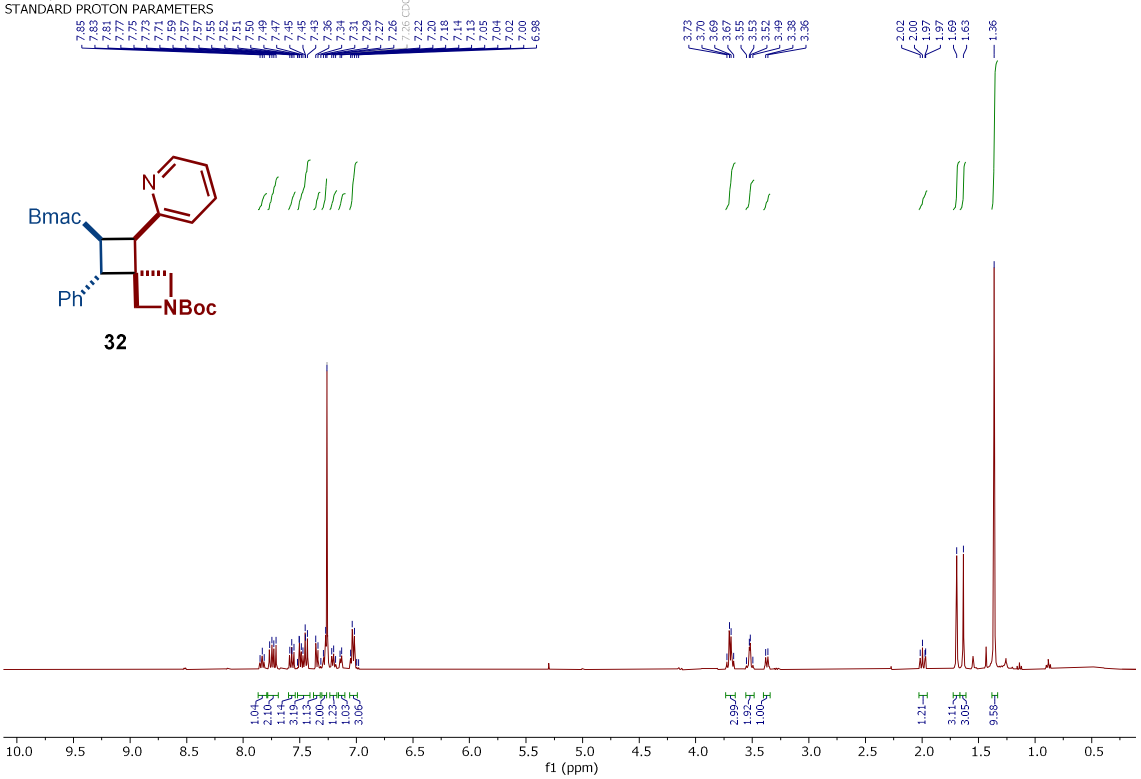

PSH-1-63.11.fid

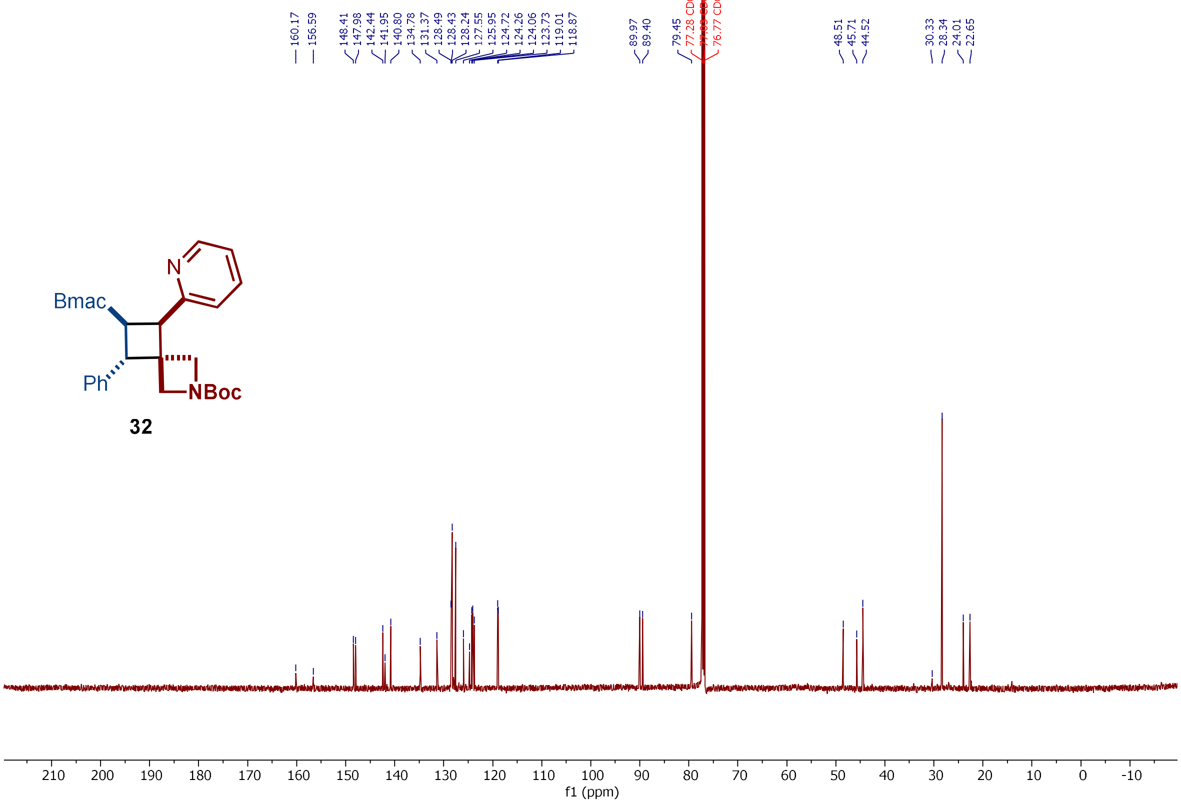

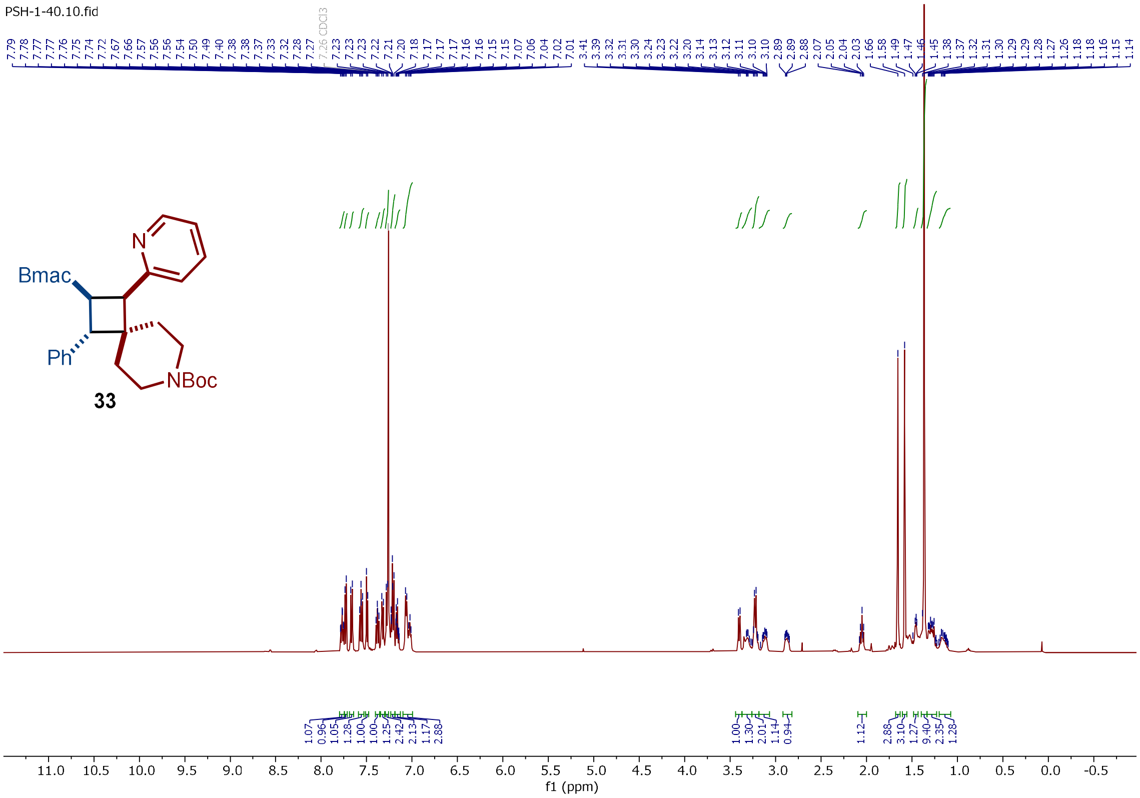
PSH-1-40.10.fid
7.79 7.78 7.77 7.76 7.75 7.74 7.72 7.67 7.66 7.57 7.56 7.56 7.54 7.50 7.49 7.40 7.38 7.38 7.37 7.33 7.32 7.28 7.27 7.26 CDCl3 7.23 7.23 7.23 7.22 7.21 7.20 7.18 7.17 7.17 7.16 7.15 7.15 7.07 7.06 7.04 7.02 7.01 3.41 3.39 3.32 3.31 3.30 3.24 3.23 3.22 3.20 3.14 3.13 3.12 3.11 3.10 3.10 2.89 2.89 2.88 2.07 2.05 2.04 2.03 1.66 1.58 1.49 1.47 1.46 1.45 1.38 1.37 1.32 1.31 1.30 1.29 1.29 1.28 1.27 1.26 1.18 1.18 1.16 1.15 1.14
Bmac
N
Ph
NBoc
33
1.07 0.96 1.05 1.28 1.00 1.00 1.25 2.42 2.13 1.17 2.88
1.00 1.30 2.01 1.14 0.94
1.12
2.88 3.10 1.27 9.40 2.35 1.28
11.0 10.5 10.0 9.5 9.0 8.5 8.0 7.5 7.0 6.5 6.0 5.5 5.0 4.5 4.0 3.5 3.0 2.5 2.0 1.5 1.0 0.5 0.0 -0.5
f1 (ppm)

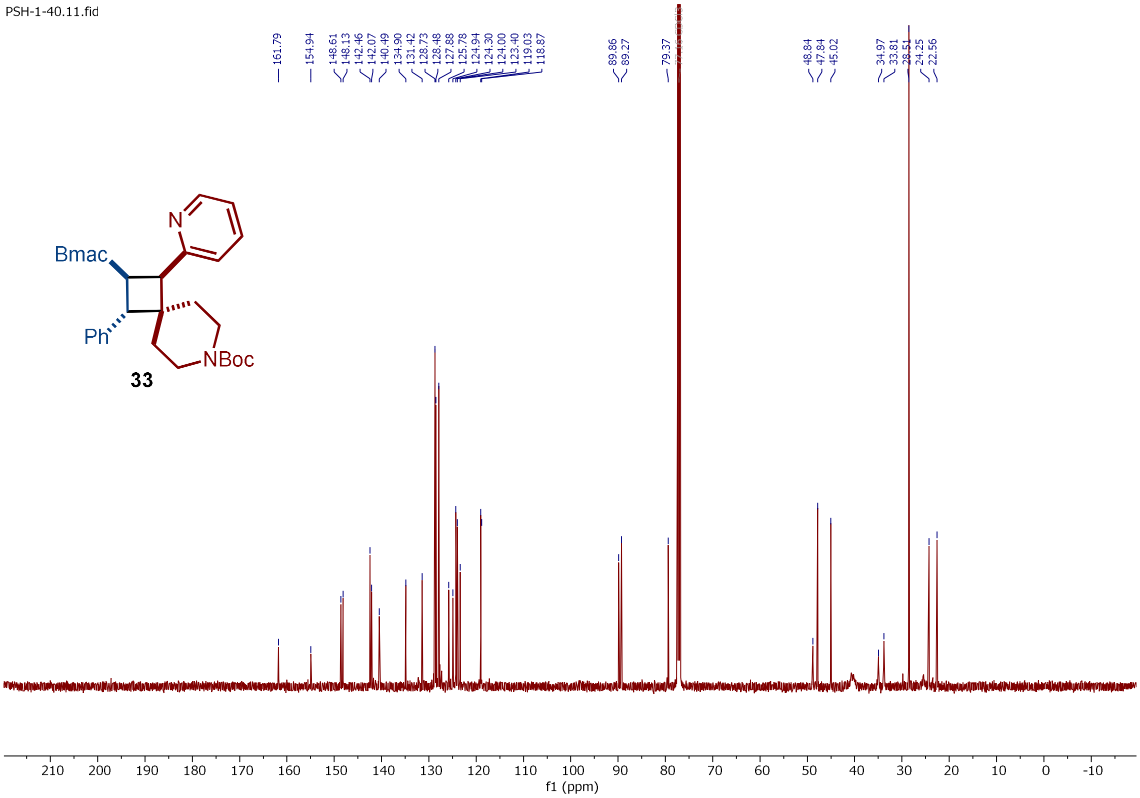
PSH-1-40.11.fid
161.79
154.94
148.61 148.13 142.46 142.07 140.49 134.90 131.42 128.73 128.48 127.88 125.78 124.94 124.30 124.00 123.40 119.03 118.87
89.86 89.27
79.37
48.84 47.84 45.02
34.97 33.81 28.51 24.25 22.56
Bmac
N
Ph
NBoc
33
210 200 190 180 170 160 150 140 130 120 110 100 90 80 70 60 50 40 30 20 10 0 -10
f1 (ppm)

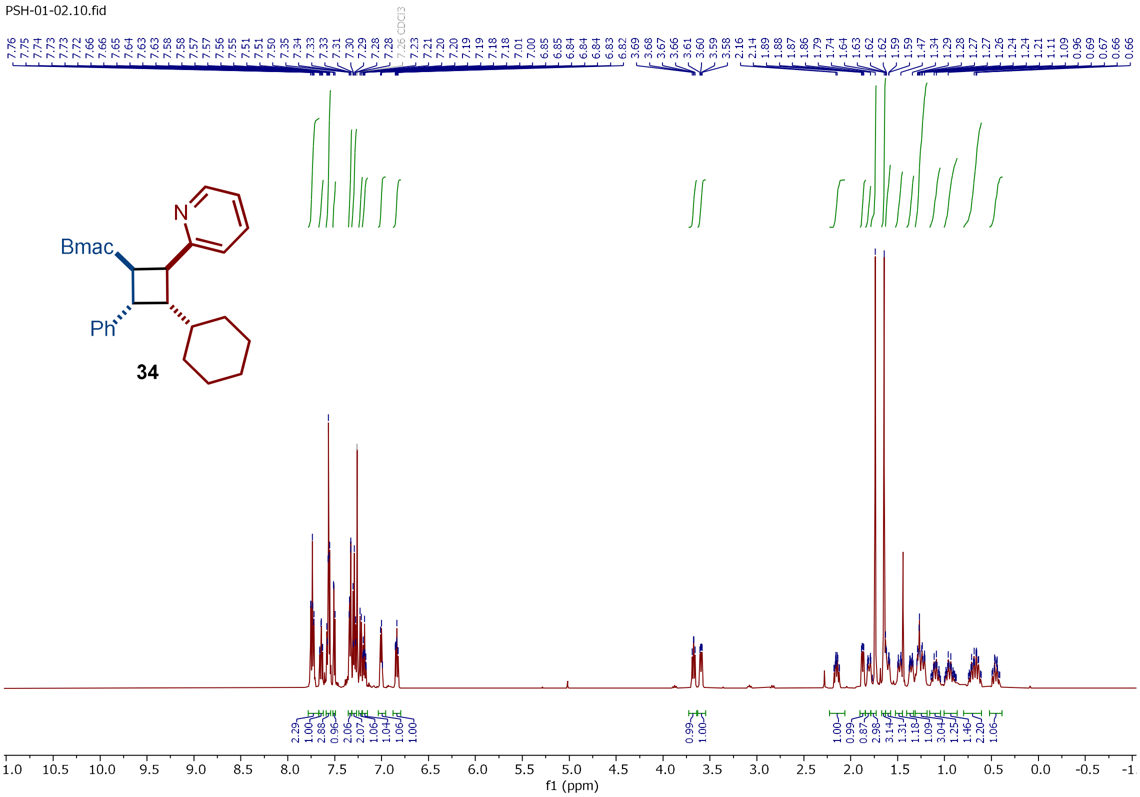
PSH-01-02.10.fid
7.76 7.75 7.74 7.73 7.72 7.66 7.65 7.64 7.63 7.63 7.58 7.58 7.57 7.57 7.56 7.55 7.51 7.51 7.50 7.35 7.34 7.33 7.33 7.31 7.30 7.29 7.28 7.28 7.26 CDCl3 7.23 7.21 7.20 7.20 7.19 7.19 7.18 7.18 7.01 7.00 6.85 6.85 6.84 6.84 6.84 6.83 6.82 3.69 3.68 3.67 3.66 3.61 3.60 3.59 3.58 2.16 2.14 1.89 1.88 1.87 1.86 1.79 1.74 1.64 1.63 1.62 1.62 1.59 1.59 1.47 1.34 1.29 1.28 1.27 1.27 1.26 1.24 1.24 1.21 1.11 1.09 0.96 0.69 0.67 0.66 0.66
N
Bmac
Ph
34
2.29 1.00 2.88 0.96 2.06 2.07 1.06 1.04 1.06 1.00 0.99 1.00 1.00 0.99 0.87 2.98 3.14 1.31 1.18 1.09 3.04 1.25 1.46 2.20 1.06
f1 (ppm)

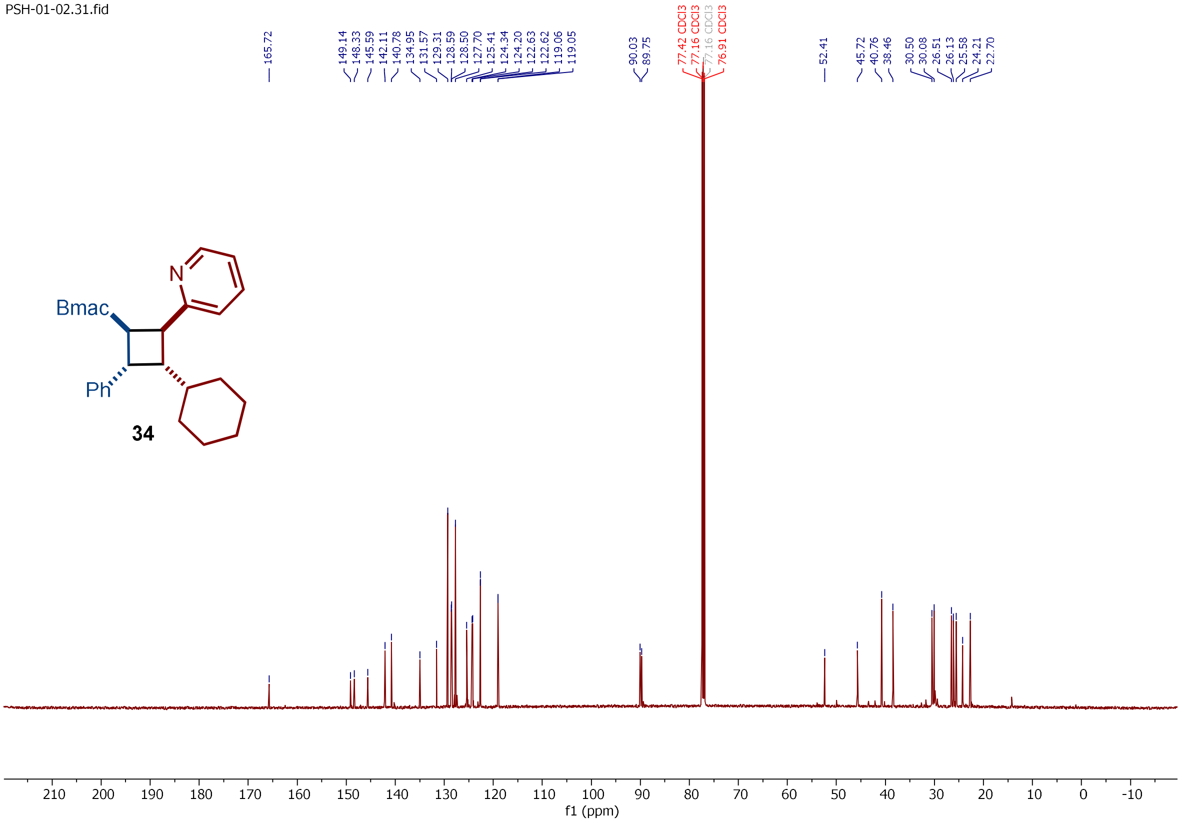
PSH-01-02.31.fid
165.72 149.14 148.33 145.59 142.11 140.78 134.95 131.57 129.31 128.59 128.50 127.70 125.41 124.34 124.20 122.63 122.62 119.06 119.05 90.03 89.75 77.42 CDCl3 77.16 CDCl3 77.16 CDCl3 76.91 CDCl3 52.41 45.72 40.76 38.46 30.50 30.08 26.51 26.13 25.58 24.21 22.70
N
Bmac
Ph
34
f1 (ppm)

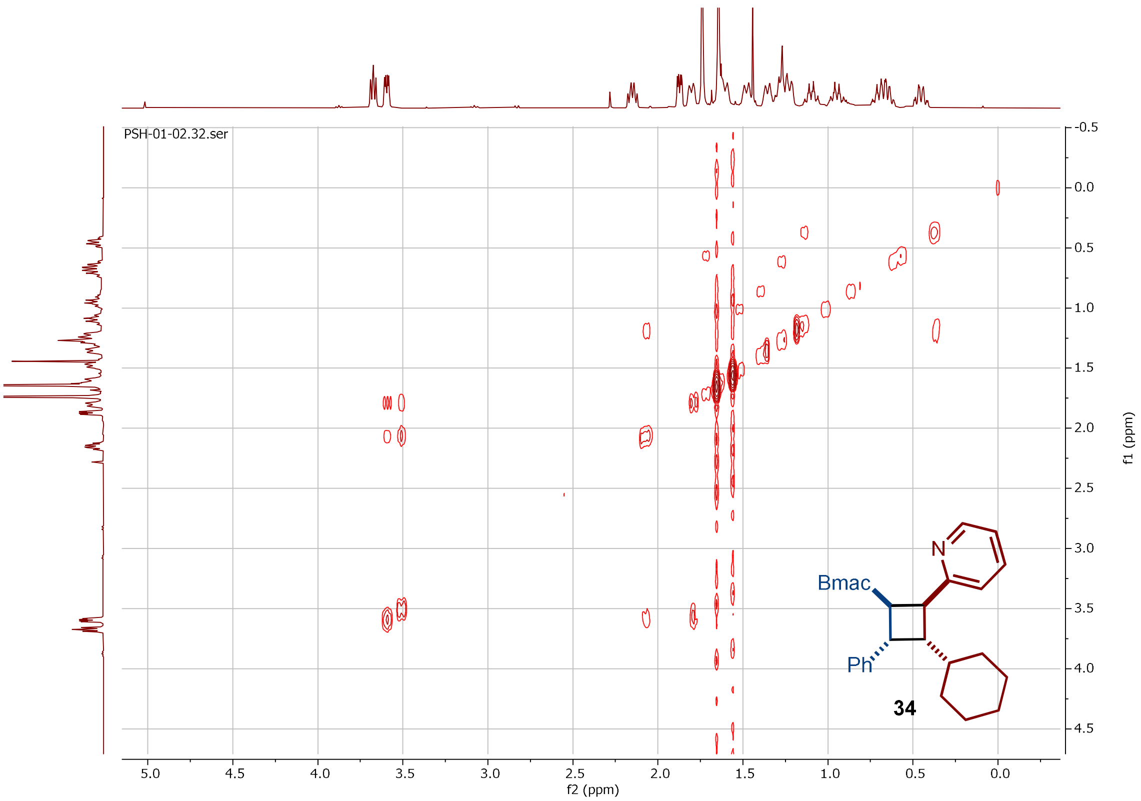
PSH-01-02.32.ser
Bmac
Ph
N
34
f2 (ppm)
f1 (ppm)

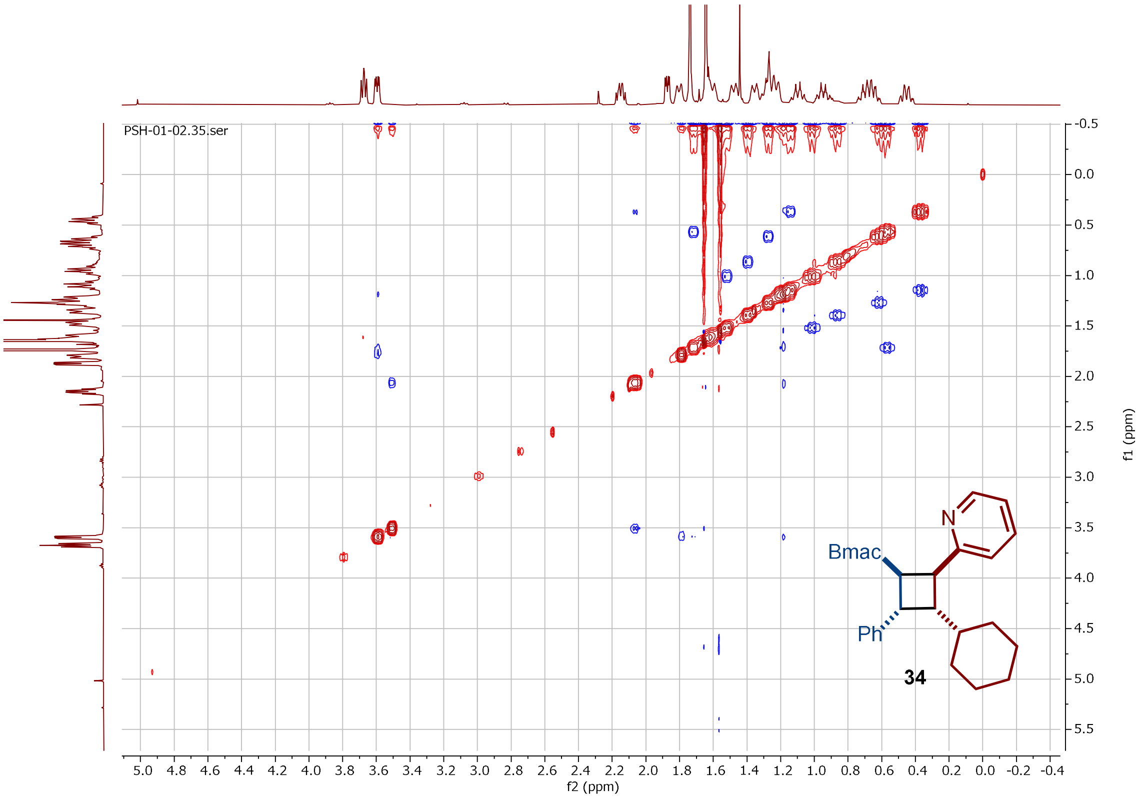
PSH-01-02.35.ser
Bmac
Ph
N
34
f2 (ppm)
f1 (ppm)

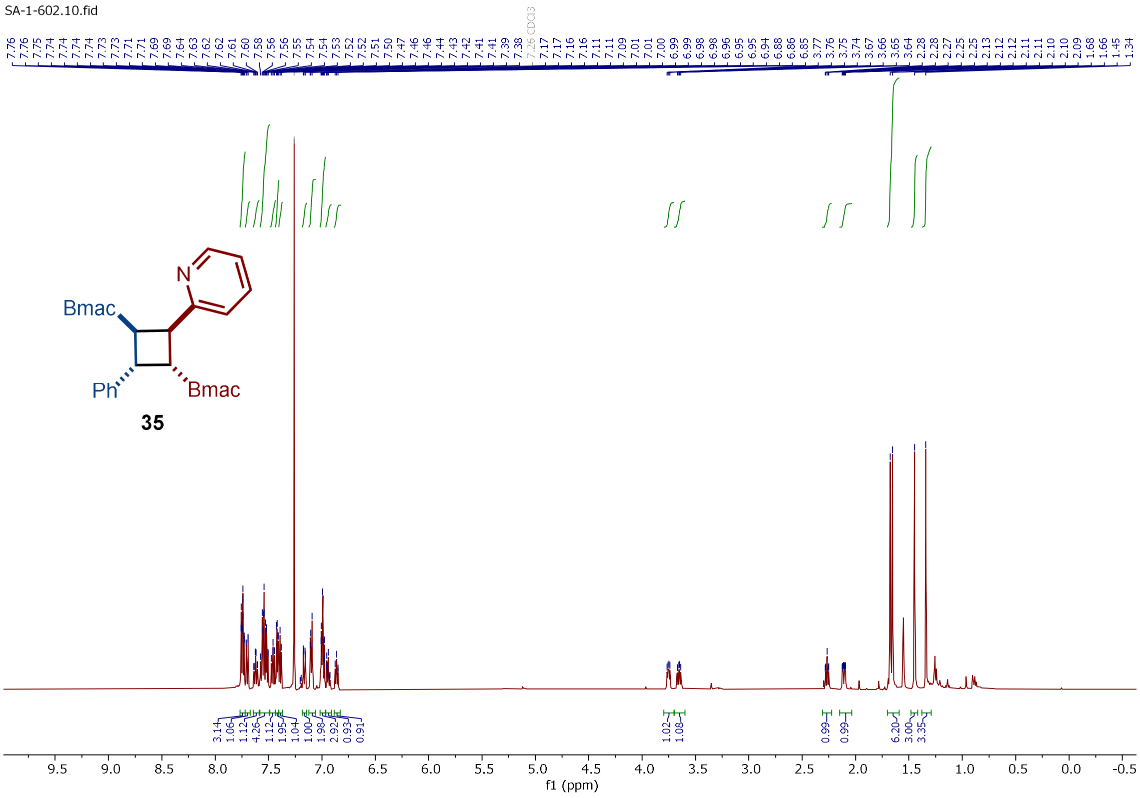
SA-1-602.10.fid
7.76 7.76 7.75 7.74 7.74 7.74 7.73 7.73 7.71 7.71 7.69 7.69 7.64 7.63 7.62 7.62 7.61 7.60 7.58 7.56 7.56 7.55 7.54 7.54 7.53 7.52 7.52 7.51 7.50 7.47 7.46 7.44 7.43 7.42 7.41 7.41 7.39 7.38
7.26 CDCl3
7.17 7.17 7.16 7.16 7.11 7.11 7.09 7.01 7.01 7.00 6.99 6.99 6.98 6.98 6.96 6.95 6.95 6.94 6.88 6.86 6.85 3.77 3.76 3.75 3.74 3.67 3.66 3.65 3.64 2.28 2.28 2.27 2.25 2.25 2.13 2.12 2.12 2.11 2.11 2.10 2.10 2.09 1.68 1.66 1.45 1.34
3.14 1.06 1.12 4.26 1.12 1.95 1.04 1.00 1.98 2.92 0.93 0.91
1.02 1.08
0.99 0.99
6.20 3.00 3.35
Bmac
N
Ph
Bmac
35
9.5 9.0 8.5 8.0 7.5 7.0 6.5 6.0 5.5 5.0 4.5 4.0 3.5 3.0 2.5 2.0 1.5 1.0 0.5 0.0 -0.5
f1 (ppm)

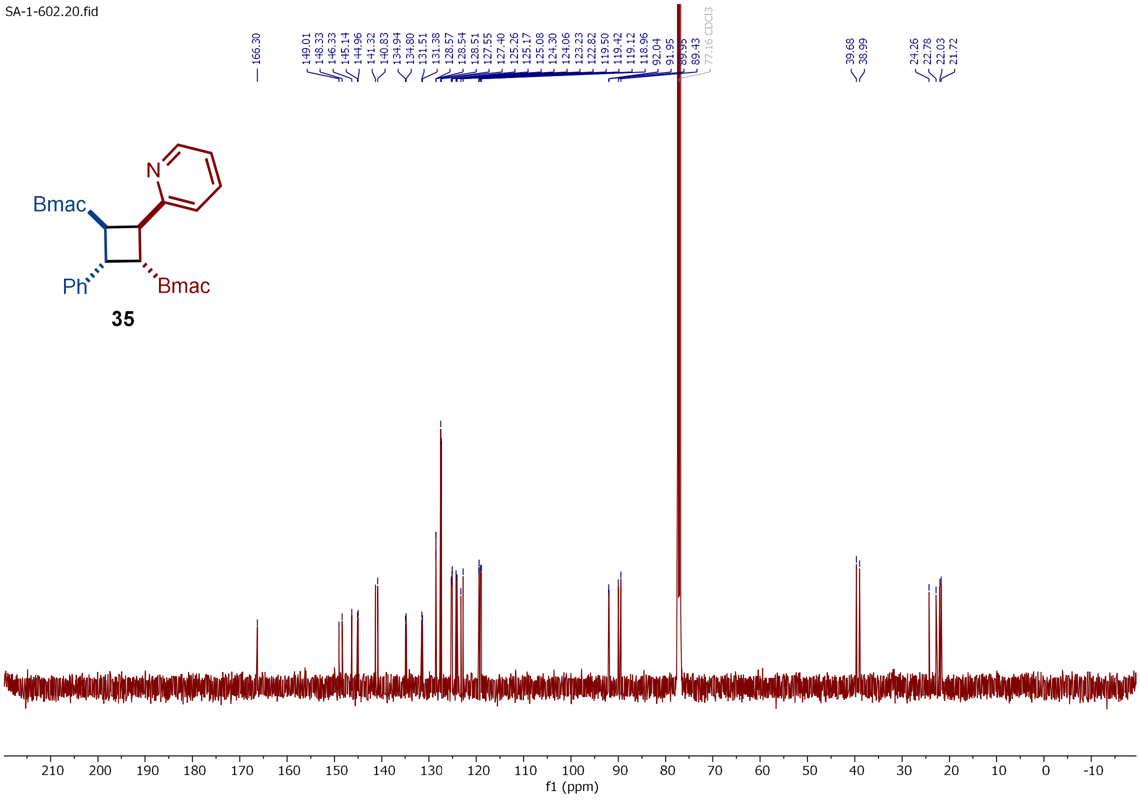
SA-1-602.20.fid
166.30
149.01 148.33 146.33 145.14 144.96 141.32 140.83 134.94 134.80 131.51 131.38 128.57 128.54 128.51 127.55 127.40 125.26 125.17 125.08 124.30 124.06 123.23 122.82 119.50 119.42 119.12 118.96 92.04 91.95 89.95 89.43
77.16 CDCl3
39.68 38.99
24.26 22.78 22.03 21.72
Bmac
N
Ph
Bmac
35
210 200 190 180 170 160 150 140 130 120 110 100 90 80 70 60 50 40 30 20 10 0 -10
f1 (ppm)

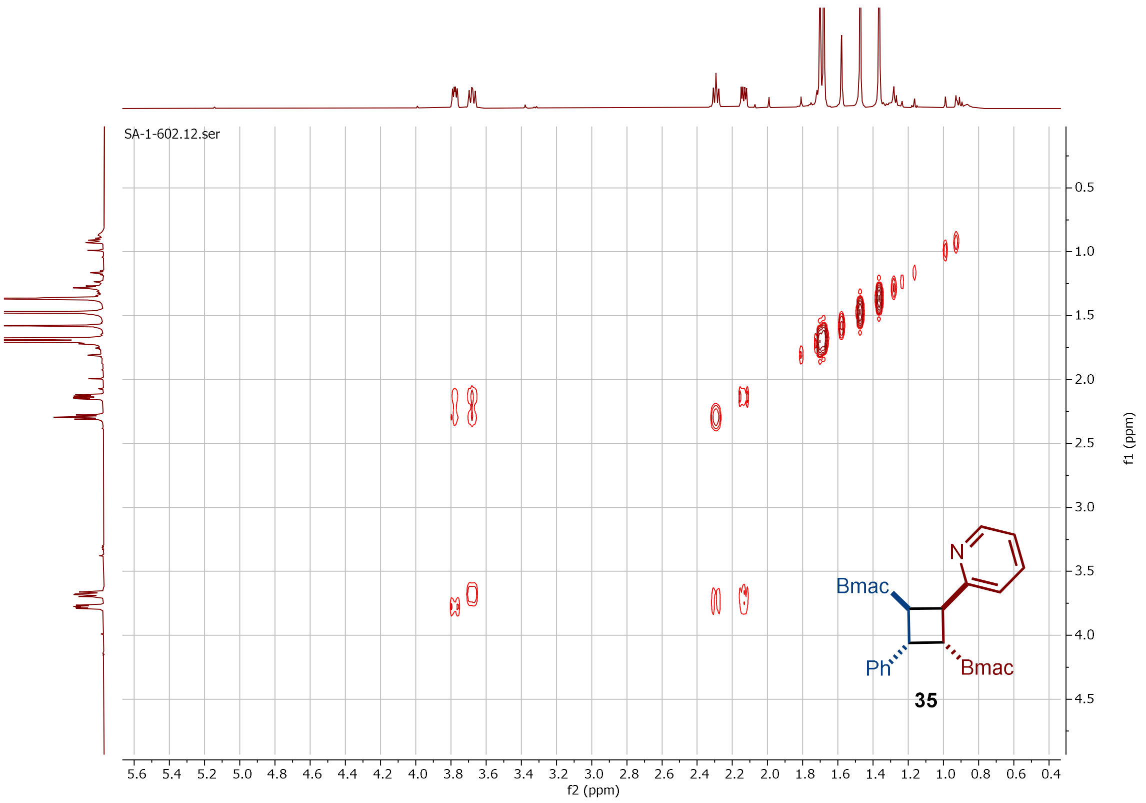
SA-1-602.12.ser
Bmac
N
Ph
Bmac
35
f2 (ppm)
f1 (ppm)

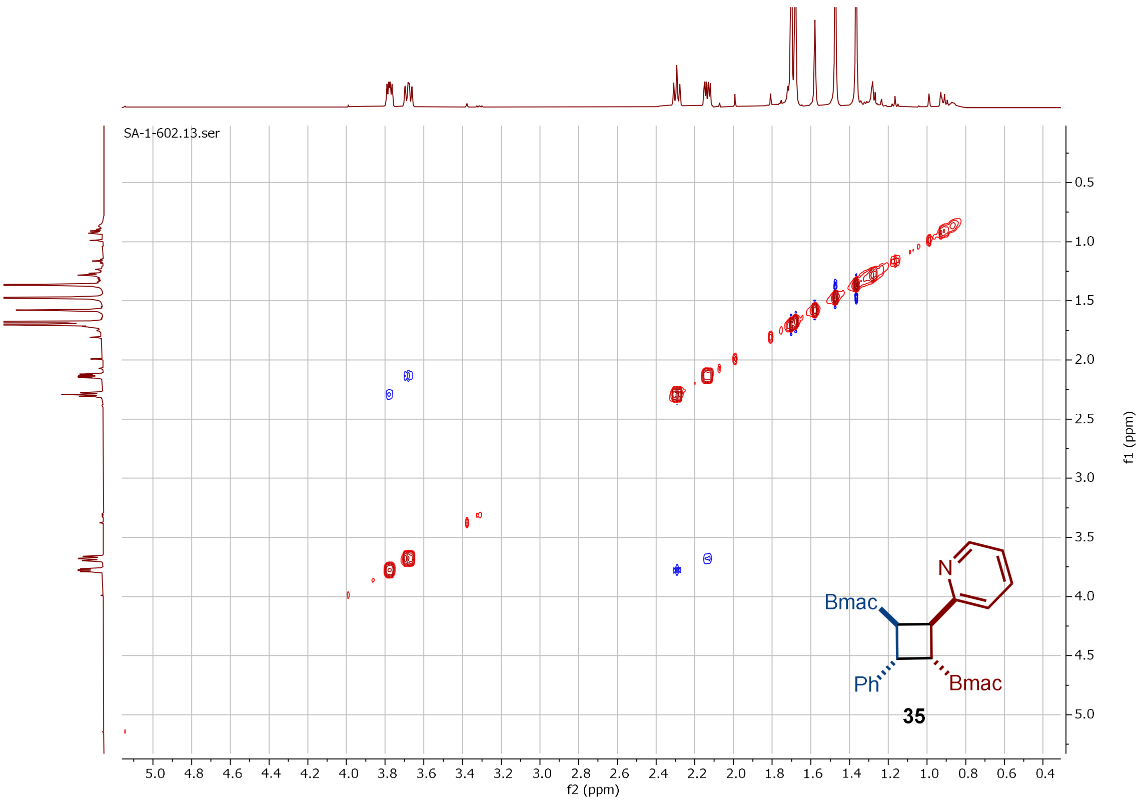
SA-1-602.13.ser
Bmac
N
Ph
Bmac
35
f2 (ppm)
f1 (ppm)

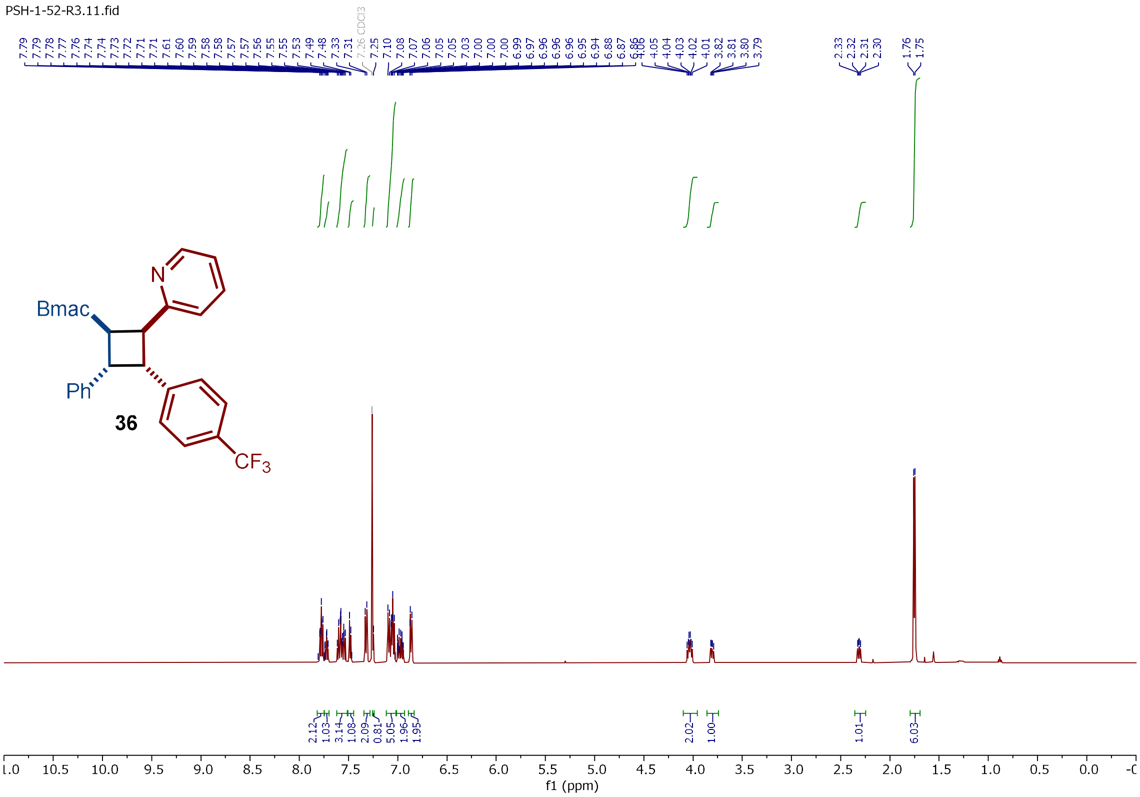
PSH-1-52-R3.11.fid
7.79 7.79 7.78 7.77 7.76 7.74 7.74 7.73 7.72 7.71 7.71 7.61 7.60 7.59 7.58 7.58 7.57 7.57 7.56 7.55 7.55 7.55 7.53 7.49 7.48 7.33 7.31 7.26 CDCl3 7.25 7.10 7.08 7.07 7.06 7.05 7.05 7.03 7.00 7.00 7.00 6.99 6.97 6.96 6.96 6.96 6.95 6.94 6.88 6.87 6.86 4.06 4.05 4.04 4.03 4.02 4.01 3.82 3.81 3.80 3.79 2.33 2.32 2.31 2.30 1.76 1.75
Bmac
N
Ph
36
CF3
2.12 1.03 3.14 1.08 2.09 0.81 5.05 1.96 1.95 2.02 1.00 1.01 6.03
f1 (ppm)

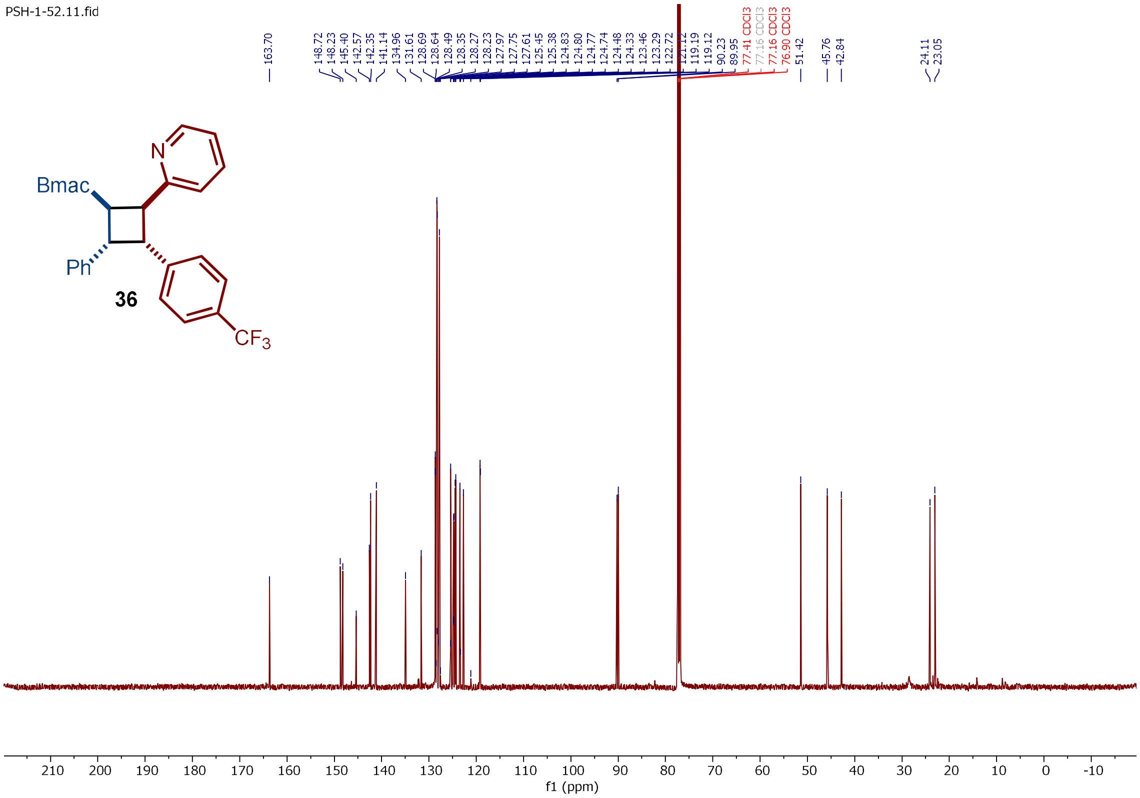
PSH-1-52.11.fid
163.70 148.72 148.23 145.40 142.57 142.35 141.14 134.96 131.61 128.69 128.64 128.49 128.35 128.27 128.23 127.97 127.75 127.61 125.45 125.38 124.83 124.80 124.77 124.74 124.48 124.33 123.46 123.29 122.72 121.12 119.19 119.12 90.23 89.95 77.41 CDCl3 77.16 CDCl3 76.90 CDCl3 51.42 45.76 42.84 24.11 23.05
Bmac
N
Ph
36
CF3
f1 (ppm)

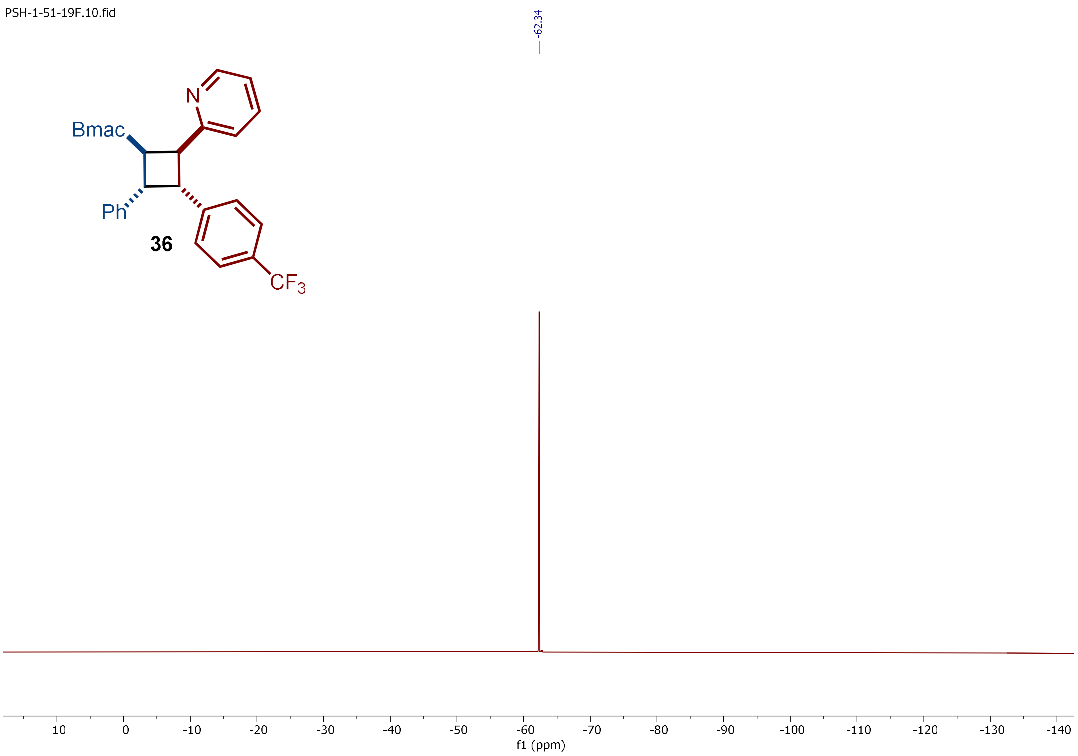
PSH-1-51-19F.10.fid
-62.34
N
Bmac
Ph
36
CF3
f1 (ppm)

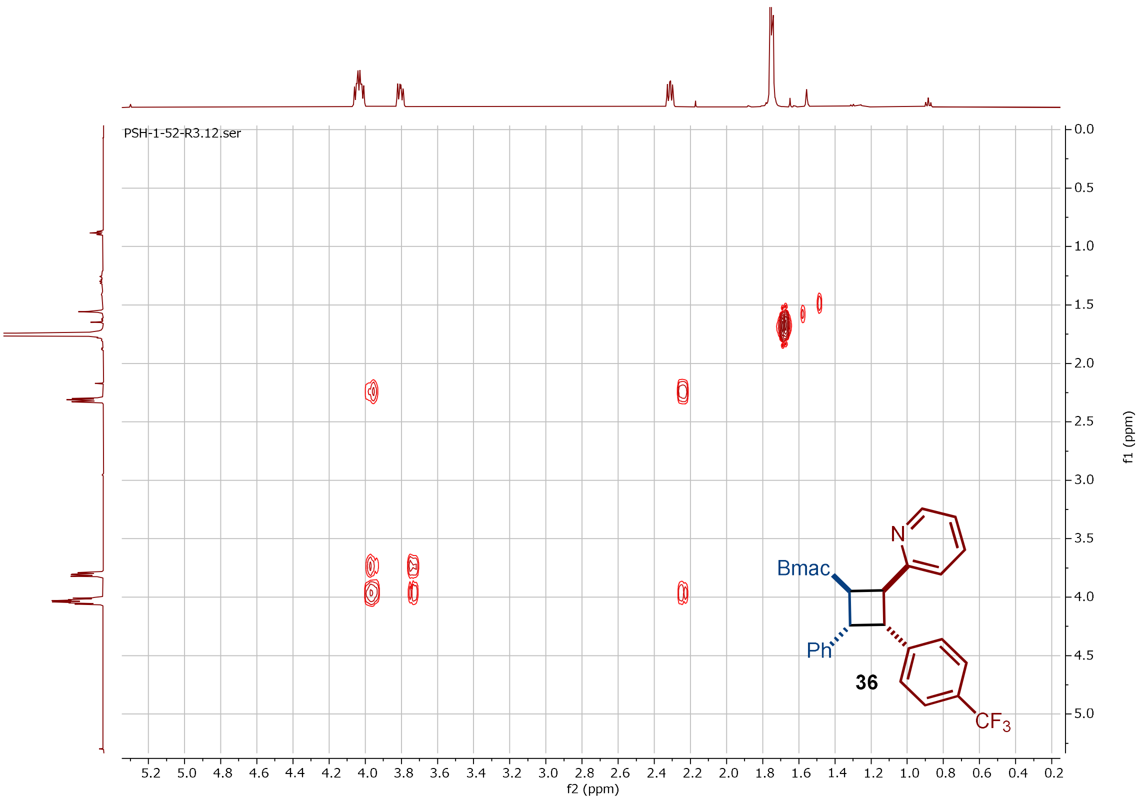
PSH-1-52-R3.12.ser
N
Bmac
Ph
36
CF3
f2 (ppm)
f1 (ppm)

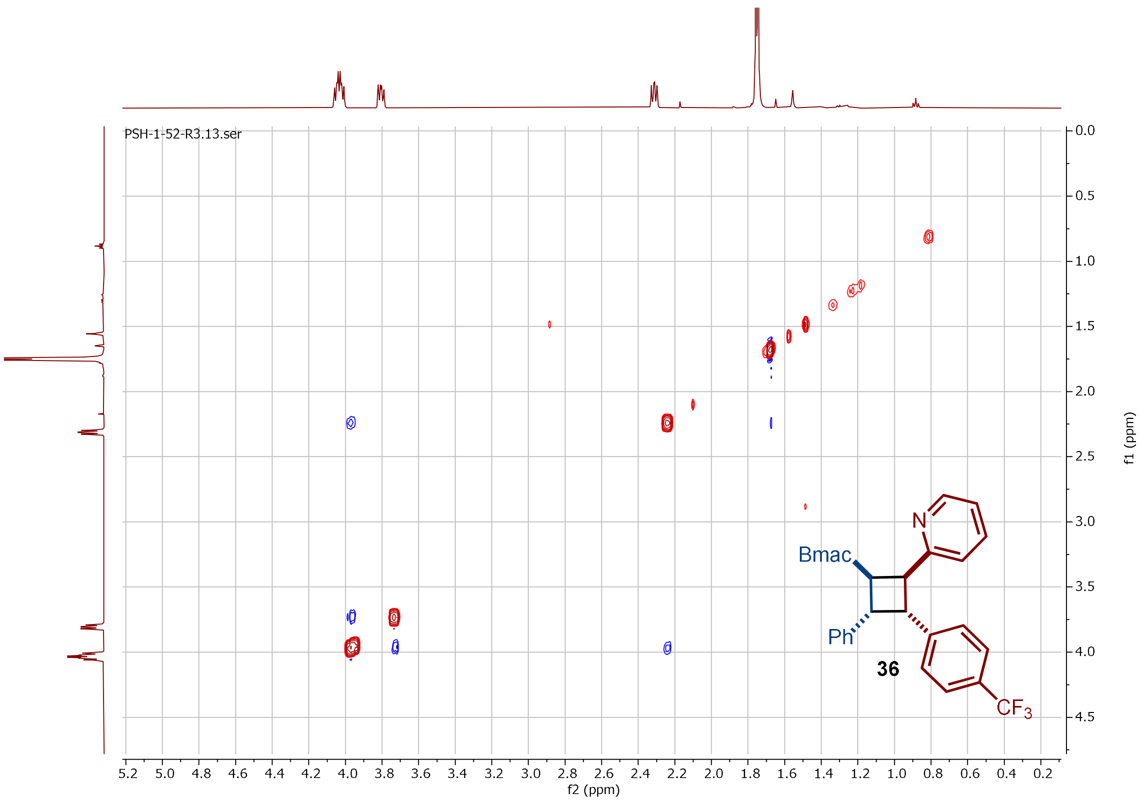
PSH-1-52-R3.13.ser
Bmac
N
Ph
36
CF_3
f2 (ppm)
f1 (ppm)

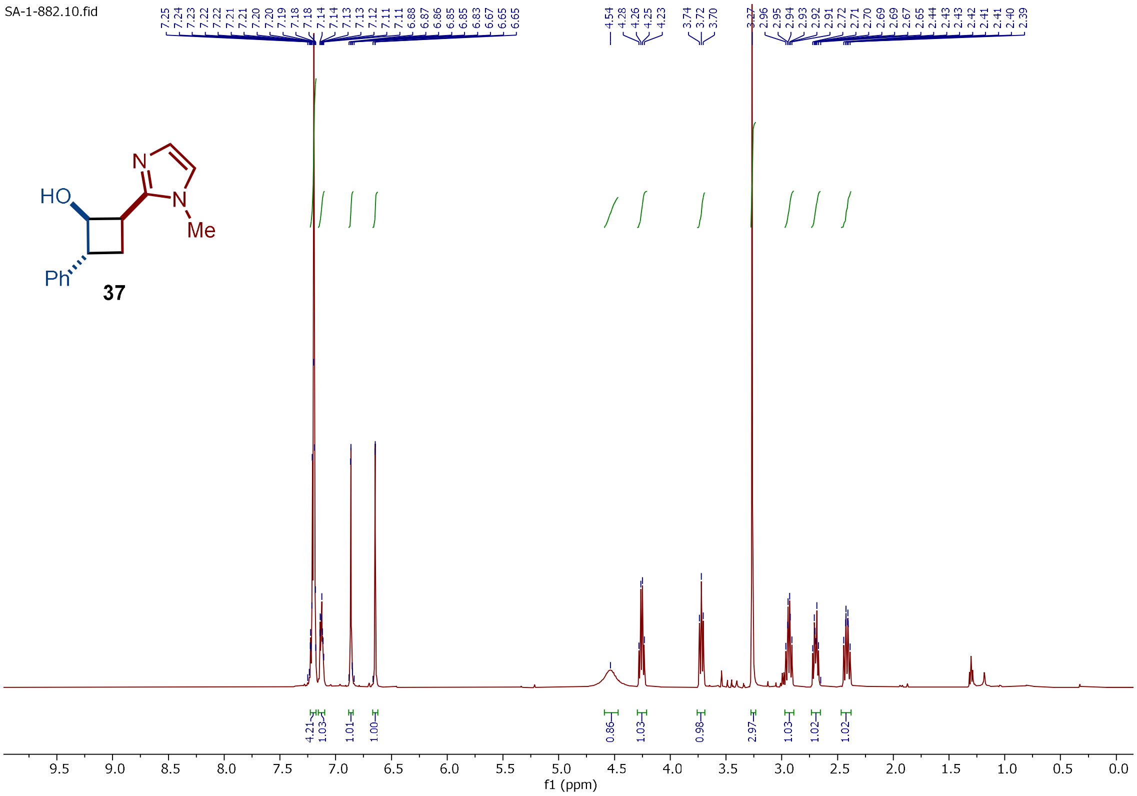
SA-1-882.10.fid
7.25 7.24 7.23 7.22 7.22 7.21 7.21 7.20 7.20 7.19 7.18 7.18 7.14 7.14 7.13 7.13 7.12 7.11 6.88 6.87 6.86 6.85 6.83 6.67 6.65 6.65
4.54 4.28 4.26 4.25 4.23
3.74 3.72 3.70
3.27 2.96 2.95 2.94 2.93 2.92 2.91 2.72 2.71 2.70 2.69 2.69 2.67 2.65 2.44 2.43 2.43 2.42 2.41 2.40 2.39
HO
N
N
Me
Ph
37
4.21 1.03 1.01 1.00 0.86 1.03 0.98 2.97 1.03 1.02 1.02
f1 (ppm)

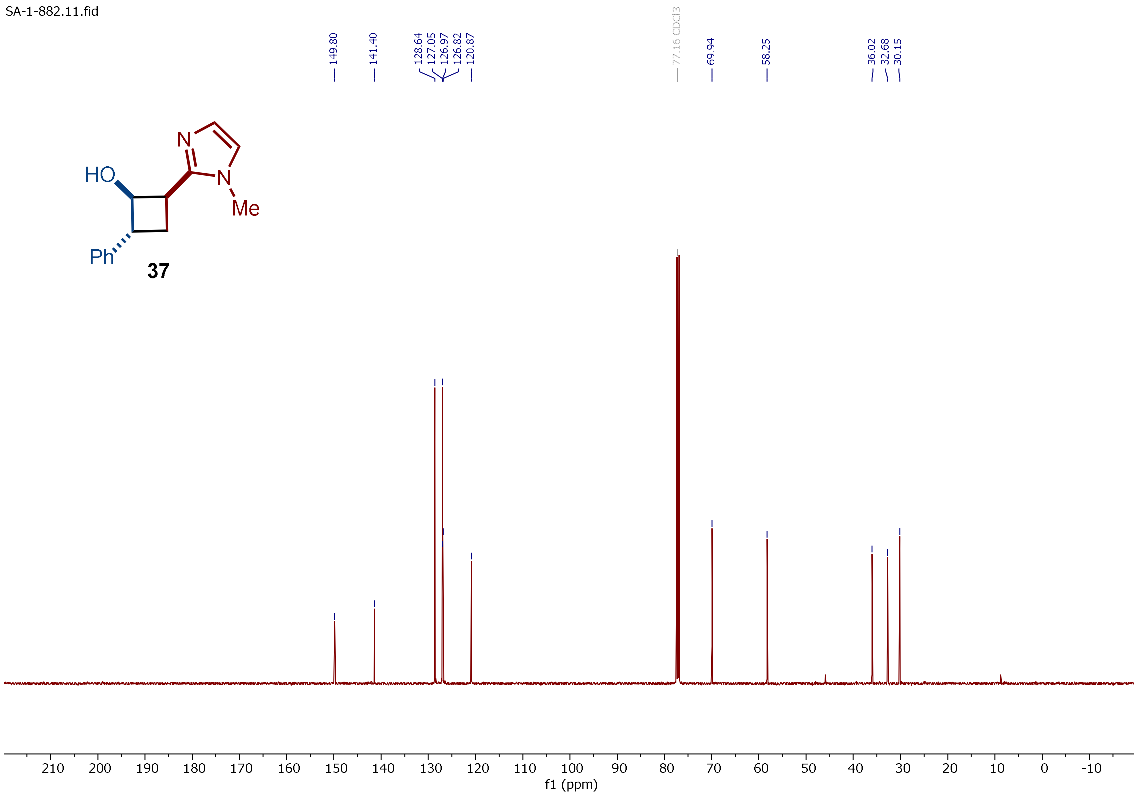
SA-1-882.11.fid
149.80
141.40
128.64
127.05
126.97
126.82
120.87
77.16 CDCl3
69.94
58.25
36.02
32.68
30.15
HO
N
N
Me
Ph
37
210 200 190 180 170 160 150 140 130 120 110 100 90 80 70 60 50 40 30 20 10 0 -10
f1 (ppm)

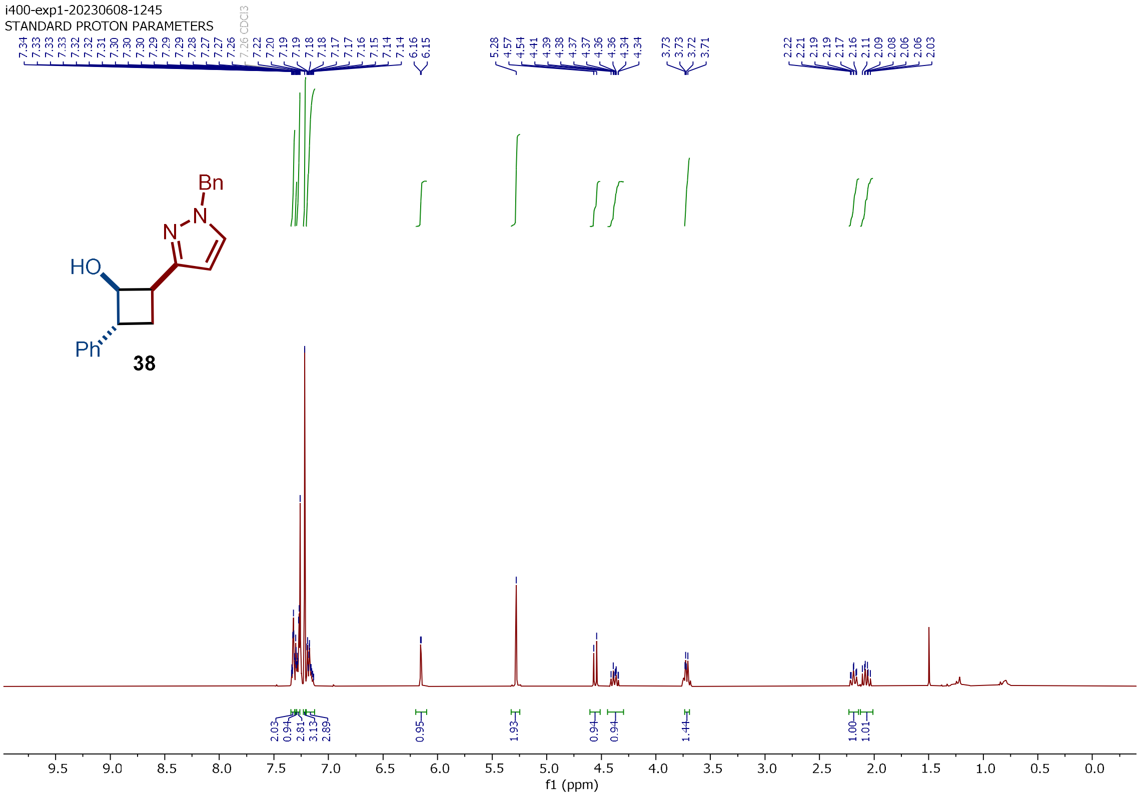
i400-exp1-20230608-1245
STANDARD PROTON PARAMETERS
7.34 7.33 7.33 7.32 7.32 7.31 7.30 7.30 7.30 7.29 7.29 7.29 7.28 7.27 7.27 7.26 7.26 CDCl3 7.22 7.20 7.19 7.19 7.18 7.18 7.17 7.16 7.15 7.14 7.14 6.16 6.15
5.28 4.57 4.54 4.41 4.39 4.38 4.37 4.36 4.36 4.34 4.34
3.73 3.73 3.72 3.71
2.22 2.21 2.19 2.19 2.17 2.16 2.11 2.09 2.08 2.06 2.06 2.03
Bn
N
N
HO
Ph
38
2.03 0.94 2.81 3.13 2.89 0.95 1.93 0.94 0.94 1.44 1.00 1.01
9.5 9.0 8.5 8.0 7.5 7.0 6.5 6.0 5.5 5.0 4.5 4.0 3.5 3.0 2.5 2.0 1.5 1.0 0.5 0.0
f1 (ppm)

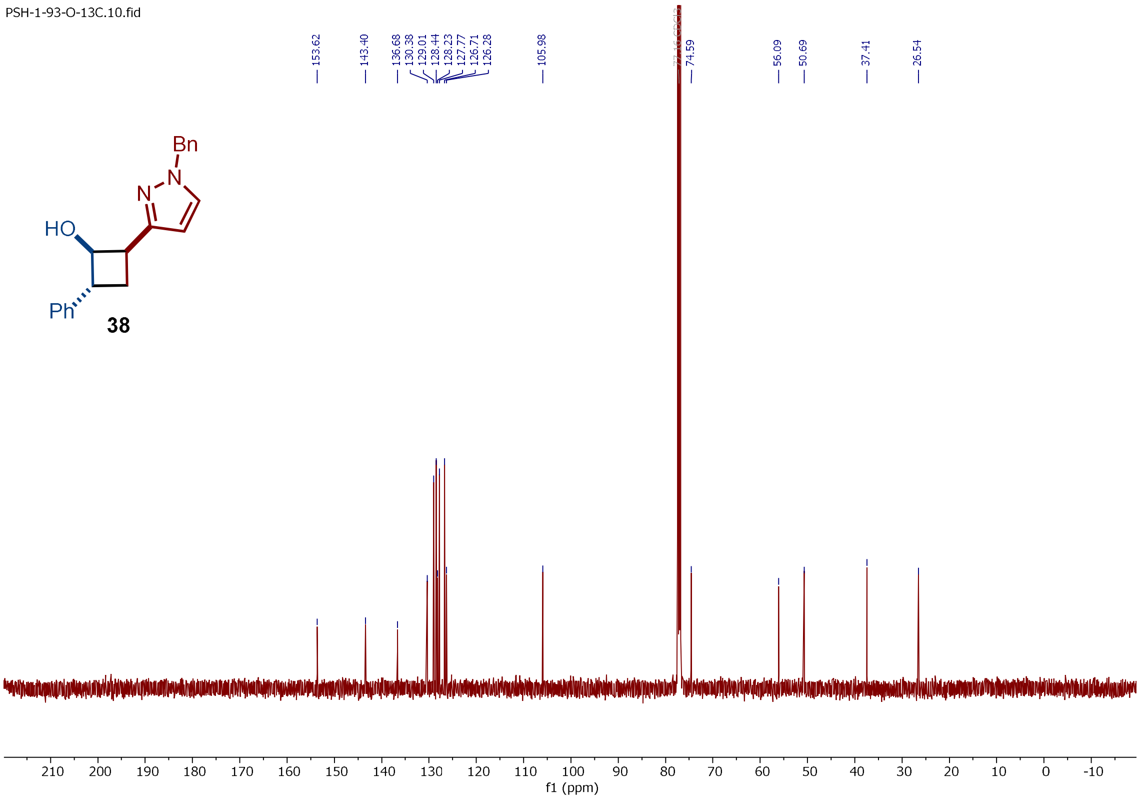
PSH-1-93-O-13C.10.fid
153.62
143.40
136.68
130.38
129.01
128.44
128.23
127.77
126.71
126.28
105.98
74.59
56.09
50.69
37.41
26.54
Bn
N
N
HO
Ph
38
210 200 190 180 170 160 150 140 130 120 110 100 90 80 70 60 50 40 30 20 10 0 -10
f1 (ppm)

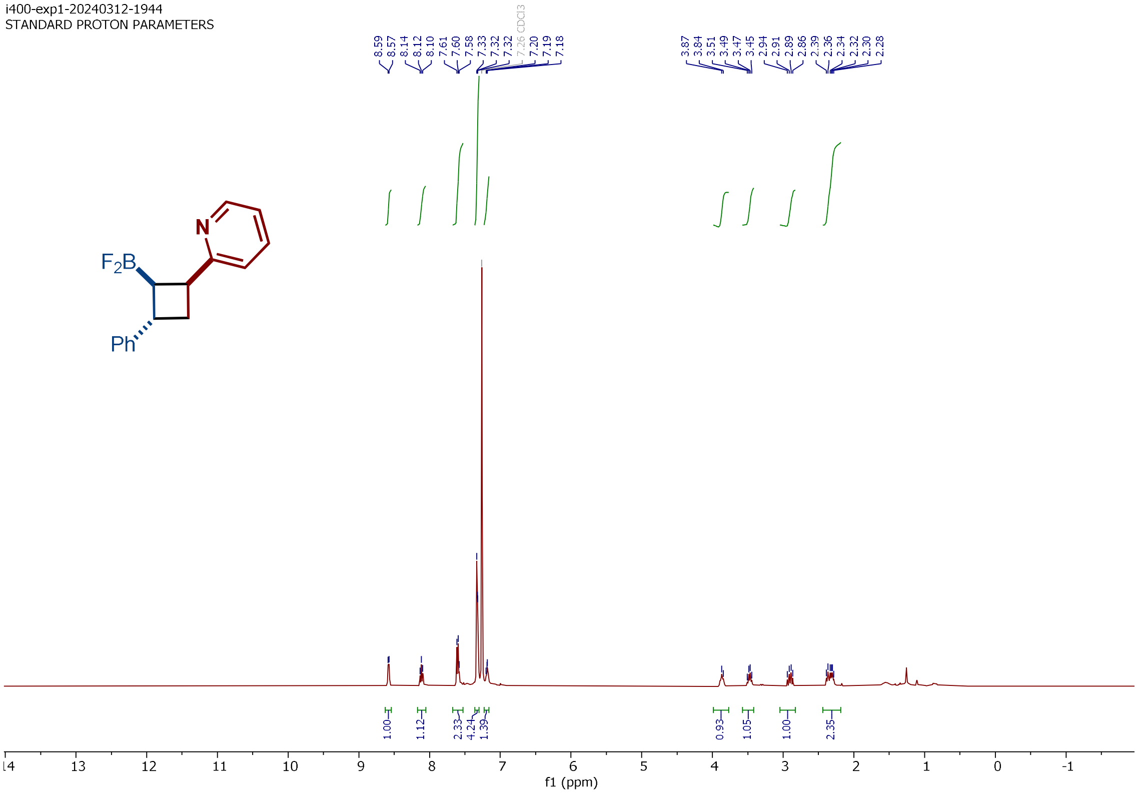
i400-exp1-20240312-1944
STANDARD PROTON PARAMETERS
8.59
8.57
8.14
8.12
8.10
7.61
7.60
7.58
7.33
7.32
7.32
7.26 CDCl3
7.20
7.19
7.18
3.87
3.84
3.51
3.49
3.47
3.45
2.94
2.91
2.89
2.86
2.39
2.36
2.34
2.32
2.30
2.28
N
F_2B
Ph
1.00
1.12
2.33
4.24
1.39
0.93
1.05
1.00
2.35
14 13 12 11 10 9 8 7 6 5 4 3 2 1 0 -1
f1 (ppm)

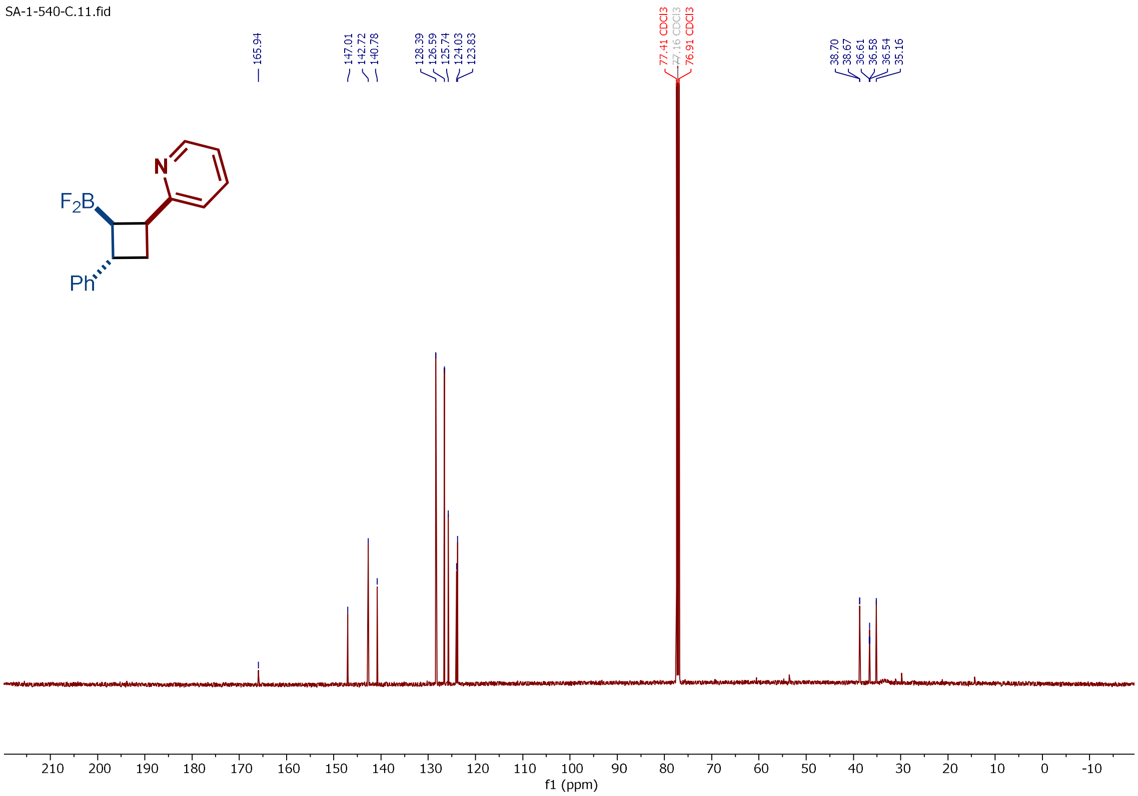
SA-1-540-C.11.fid
165.94
147.01
142.72
140.78
128.39
126.59
125.74
124.03
123.83
77.41 CDCl3
77.16 CDCl3
76.91 CDCl3
38.70
38.67
36.61
36.58
36.54
35.16
F2B
N
Ph
f1 (ppm)

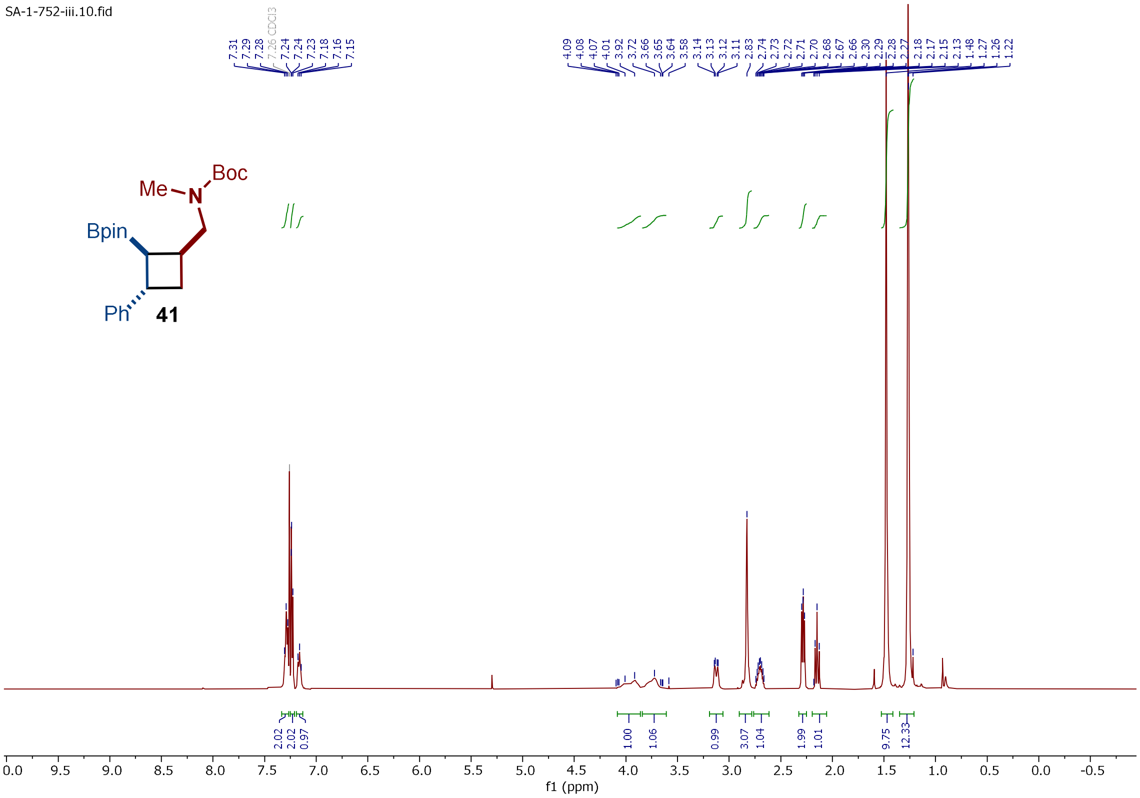
SA-1-752-iii.10.fid
7.31
7.29
7.28
7.26 CDCl3
7.24
7.24
7.24
7.23
7.18
7.16
7.15
4.09
4.08
4.07
4.01
3.92
3.72
3.66
3.65
3.64
3.58
3.14
3.13
3.12
3.11
2.83
2.74
2.73
2.72
2.71
2.70
2.68
2.67
2.66
2.30
2.29
2.28
2.27
2.18
2.17
2.15
2.13
1.48
1.27
1.26
1.22
Me
N
Boc
Bpin
Ph
41
2.02
2.02
0.97
1.00
1.06
0.99
3.07
1.04
1.99
1.01
9.75
12.33
f1 (ppm)

SA-1-752-ii.11.fid

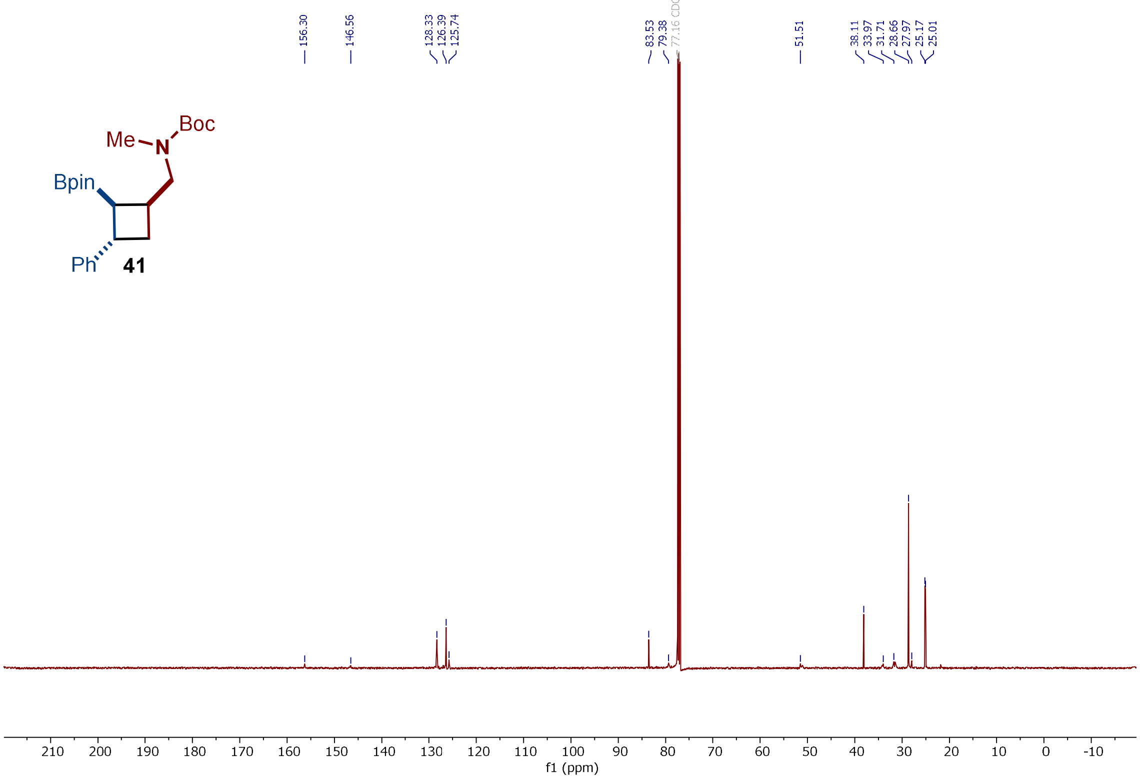

SA-1-758.10.fid

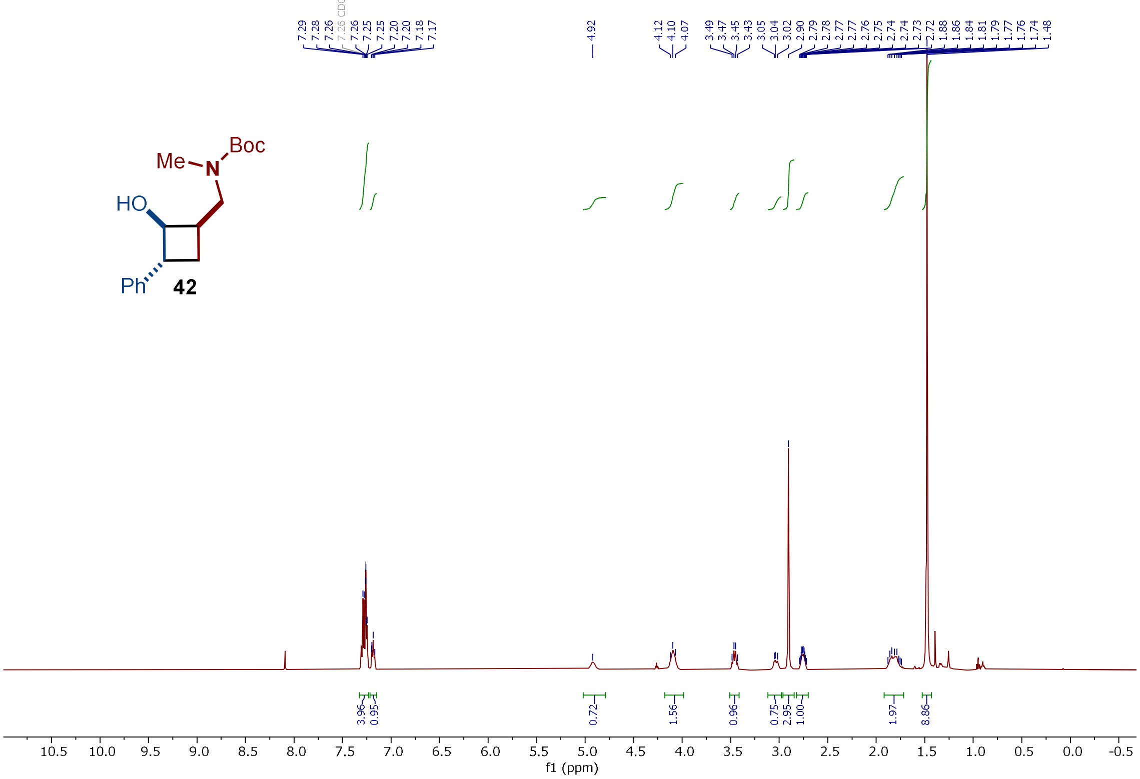

SA-1-758.11.fid

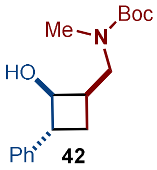

42

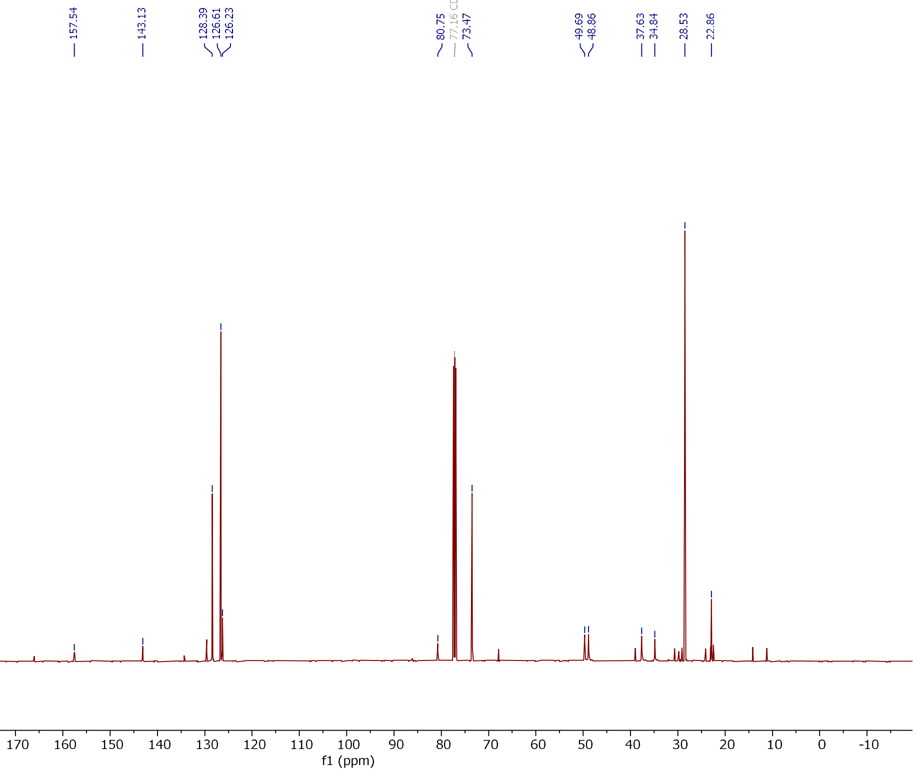

SA-1-757-ii.10.fid

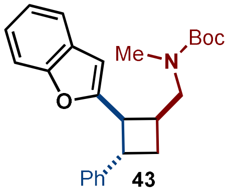

43

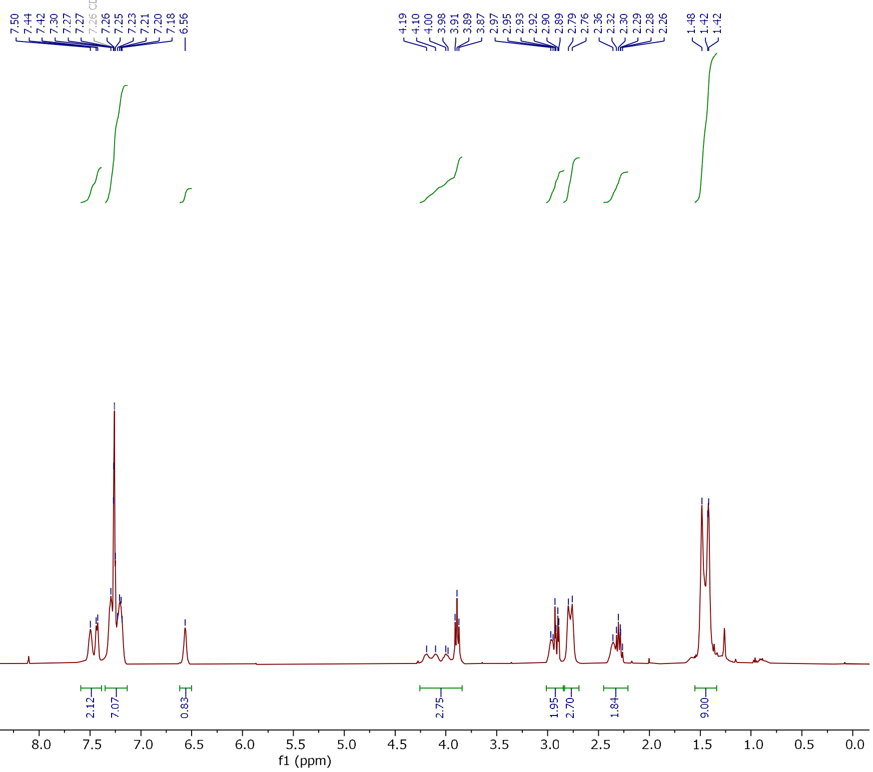

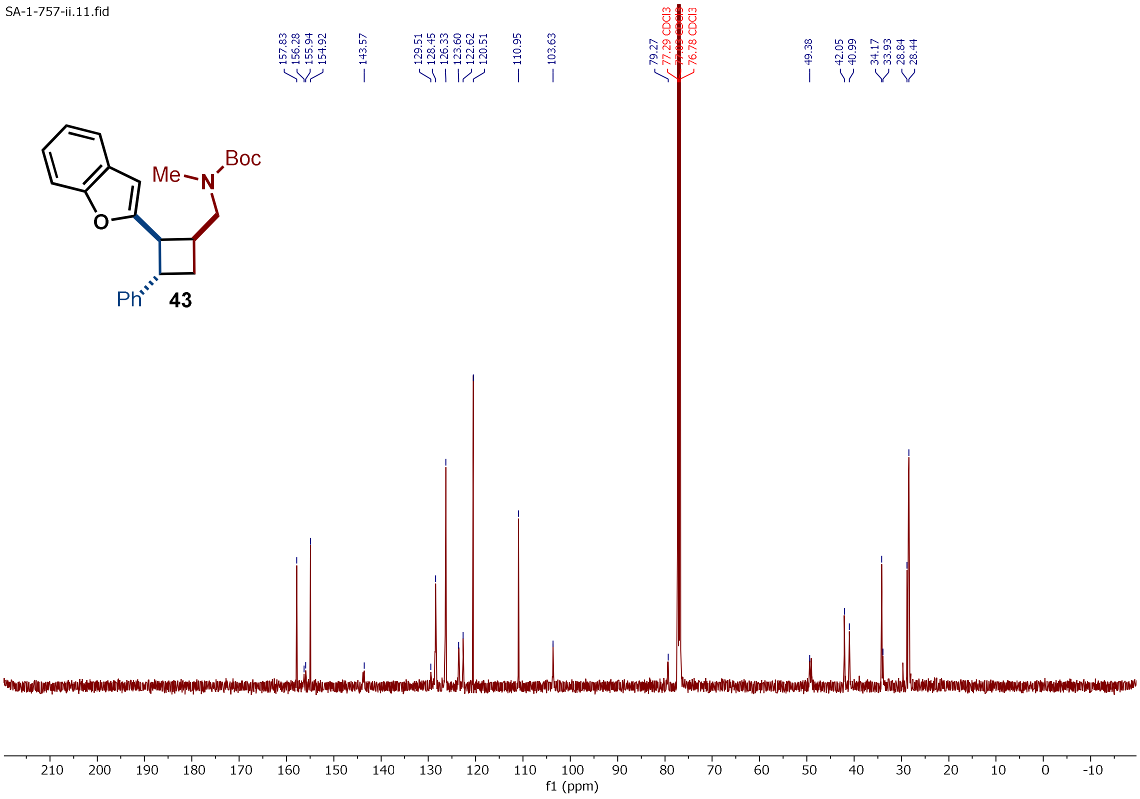
SA-1-757-ii.11.fid
157.83
156.28
155.94
154.92
143.57
129.51
128.45
126.33
123.60
122.62
120.51
110.95
103.63
79.27
77.29 CDCl3
76.78 CDCl3
49.38
42.05
40.99
34.17
33.93
28.84
28.44
Me
N
Boc
O
Ph
43
210 200 190 180 170 160 150 140 130 120 110 100 90 80 70 60 50 40 30 20 10 0 -10
f1 (ppm)

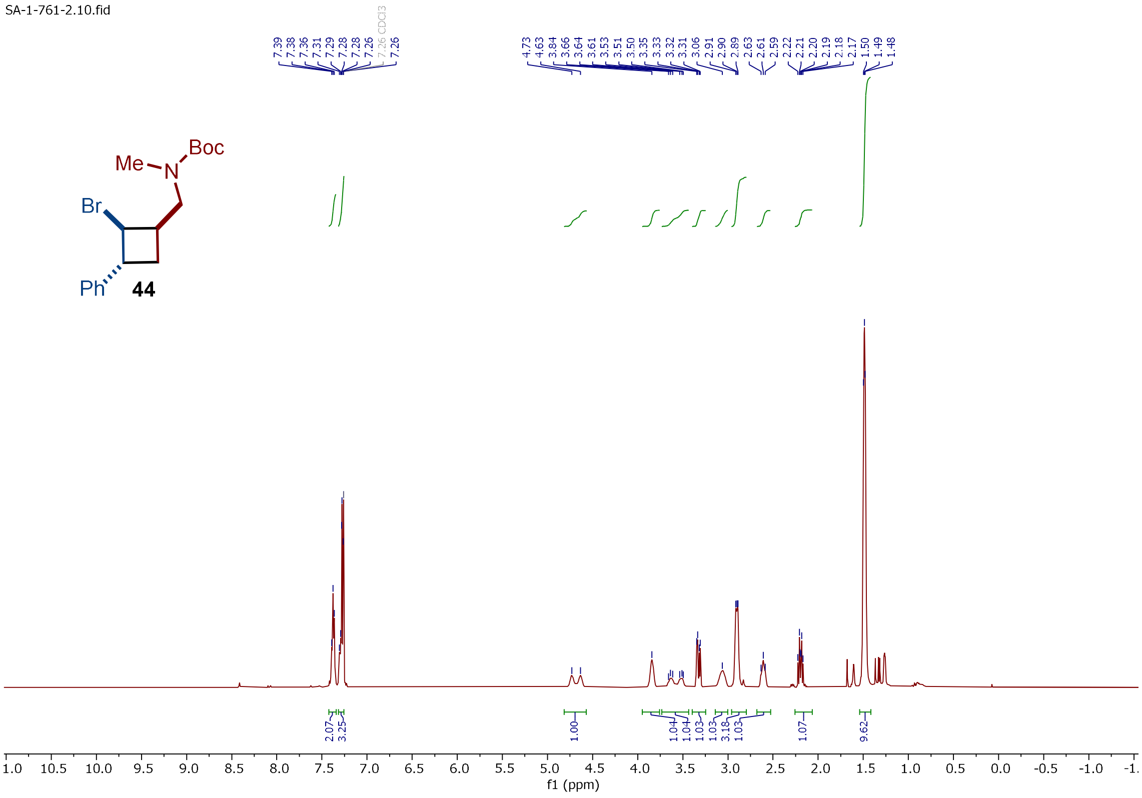
SA-1-761-2.10.fid
7.39
7.38
7.36
7.31
7.29
7.28
7.28
7.26
7.26 CDCl3
7.26
4.73
4.63
3.84
3.66
3.64
3.61
3.53
3.51
3.50
3.35
3.33
3.32
3.31
3.06
2.91
2.90
2.89
2.63
2.61
2.59
2.22
2.21
2.20
2.19
2.18
2.17
1.50
1.49
1.48
Me
N
Boc
Br
Ph
44
2.07
3.25
1.00
1.04
1.04
1.03
1.03
3.18
1.03
1.07
9.62
1.0 10.5 10.0 9.5 9.0 8.5 8.0 7.5 7.0 6.5 6.0 5.5 5.0 4.5 4.0 3.5 3.0 2.5 2.0 1.5 1.0 0.5 0.0 -0.5 -1.0 -1.
f1 (ppm)

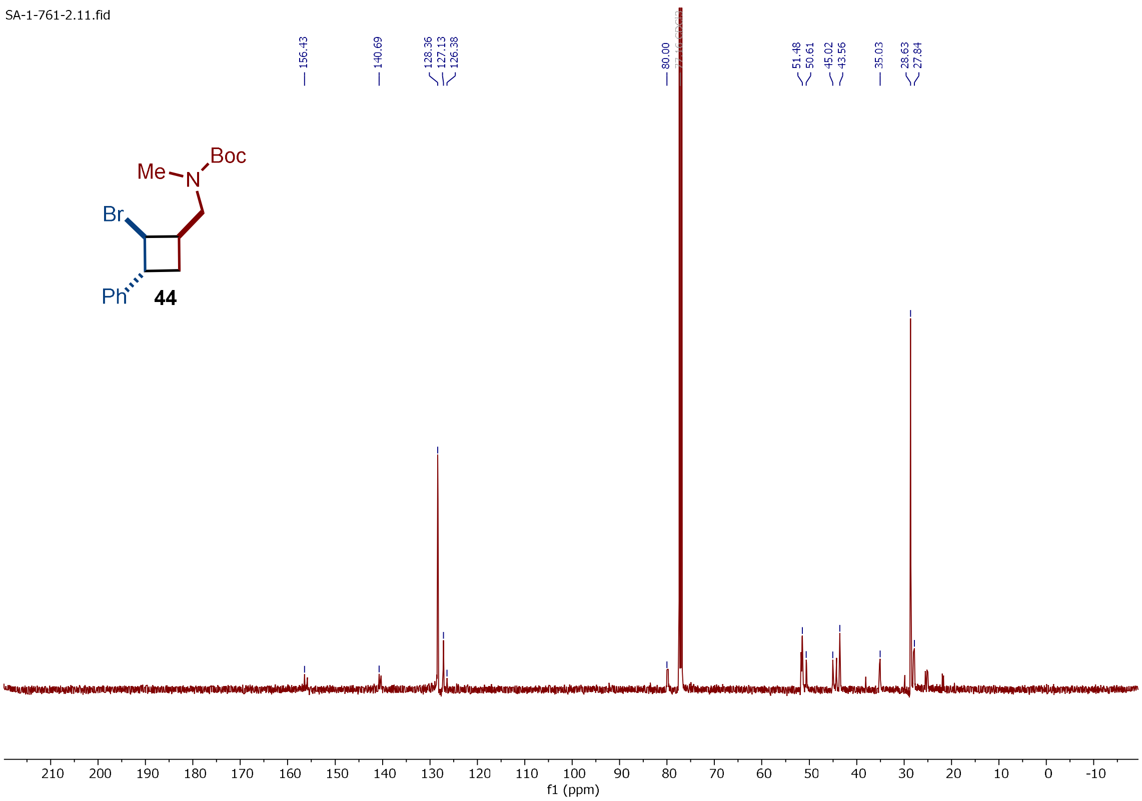
SA-1-761-2.11.fid
156.43
140.69
128.36
127.13
126.38
80.00
51.48
50.61
45.02
43.56
35.03
28.63
27.84
Me
N
Boc
Br
Ph
44
f1 (ppm)

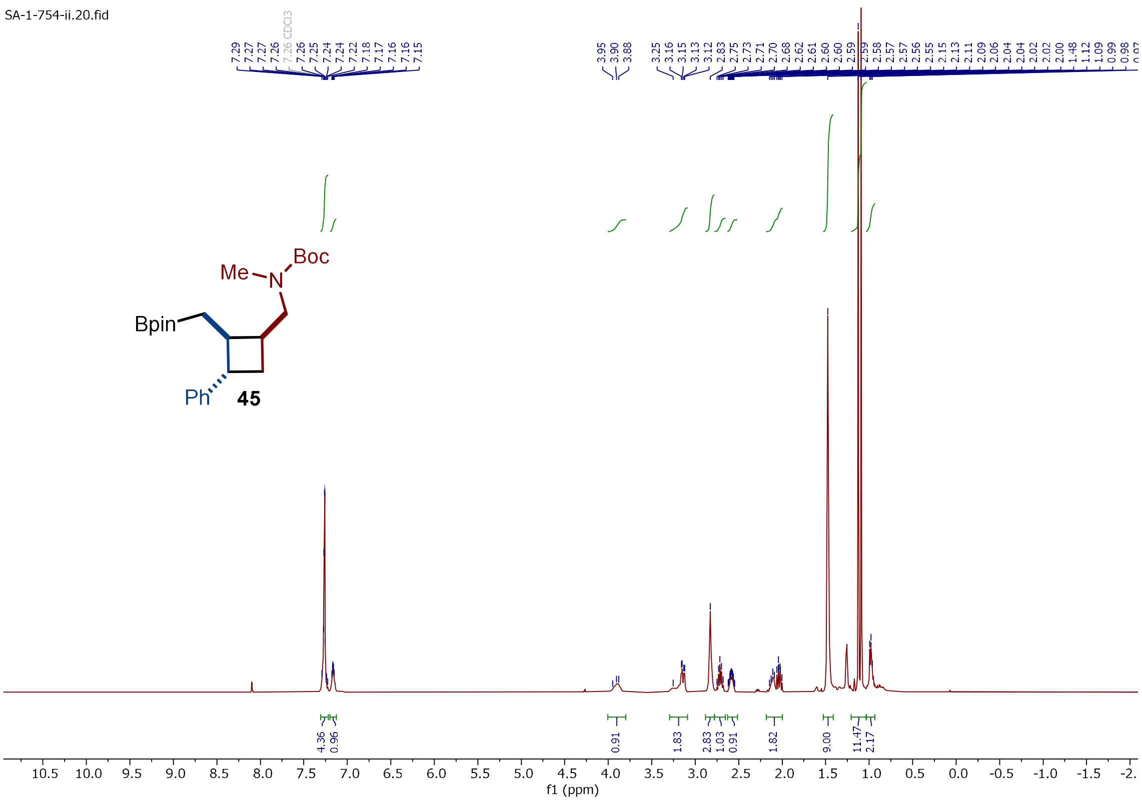
SA-1-754-ii.20.fid
7.29
7.27
7.26
7.26 CDCl3
7.26
7.25
7.24
7.24
7.22
7.18
7.17
7.16
7.15
3.95
3.90
3.88
3.25
3.16
3.15
3.13
3.12
2.83
2.75
2.73
2.71
2.70
2.68
2.62
2.61
2.60
2.60
2.59
2.59
2.58
2.57
2.57
2.56
2.55
2.15
2.13
2.11
2.09
2.06
2.04
2.04
2.02
2.02
2.00
1.48
1.12
1.09
0.99
0.98
Me
N
Boc
Bpin
Ph
45
4.36
0.96
0.91
1.83
2.83
1.03
0.91
1.82
9.00
11.47
2.17
f1 (ppm)

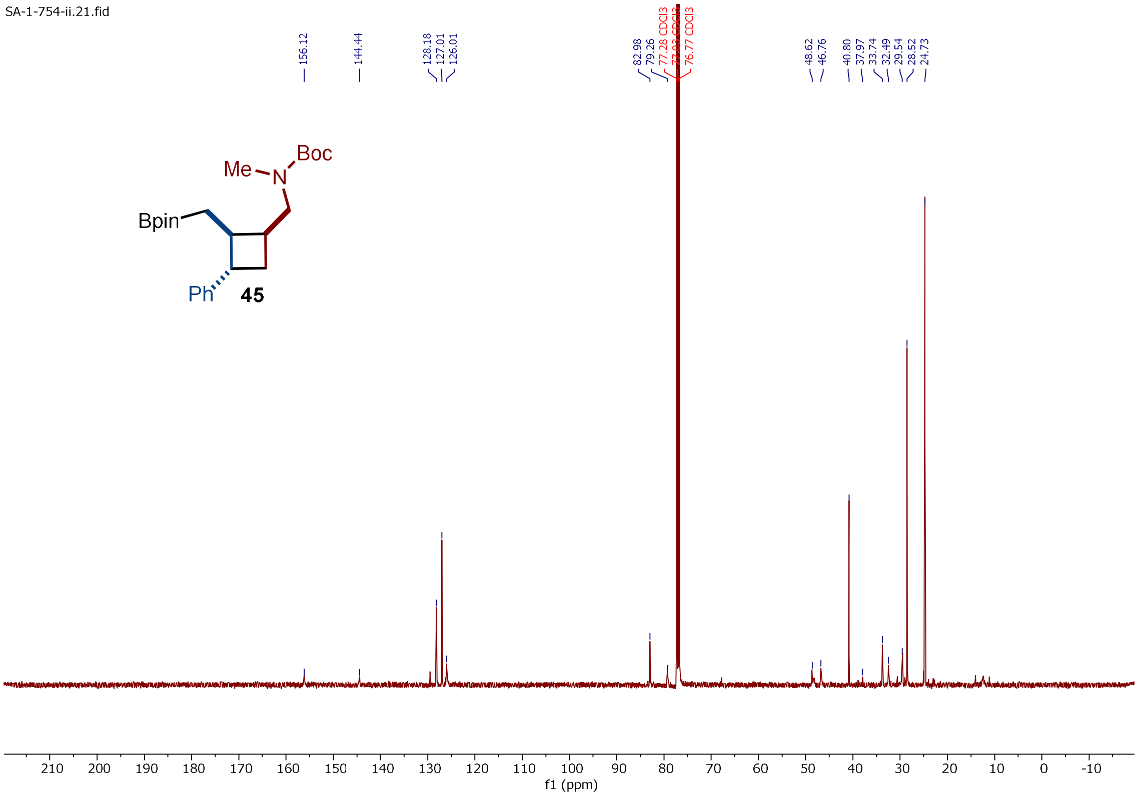
SA-1-754-ii.21.fid
156.12
144.44
128.18
127.01
126.01
82.98
79.26
77.28 CDCl3
76.77 CDCl3
48.62
46.76
40.80
37.97
33.74
32.49
29.54
28.52
24.73
Me
N
Boc
Bpin
Ph
45
f1 (ppm)

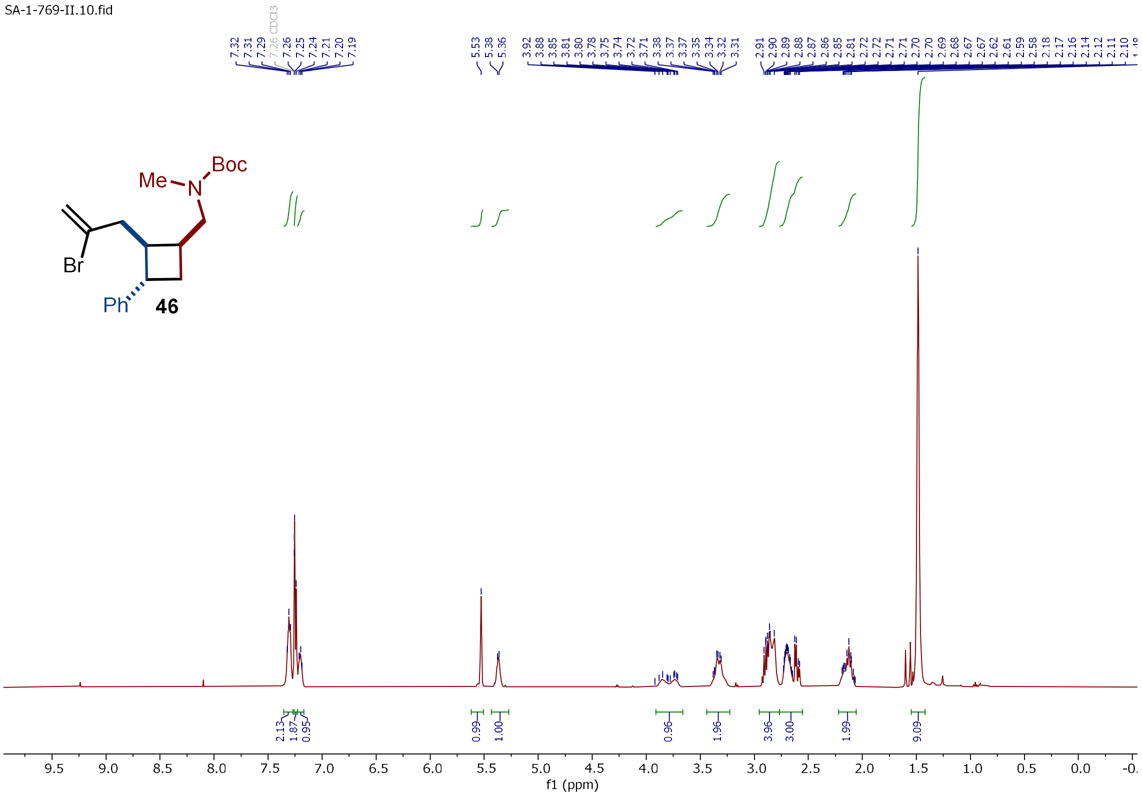
SA-1-769-II.10.fid
7.26 CDCl3
Me
N
Boc
Br
Ph
46
2.13
1.87
0.95
0.99
1.00
0.96
1.96
3.96
3.00
1.99
9.09
f1 (ppm)

SA-1-769-II.11.fid

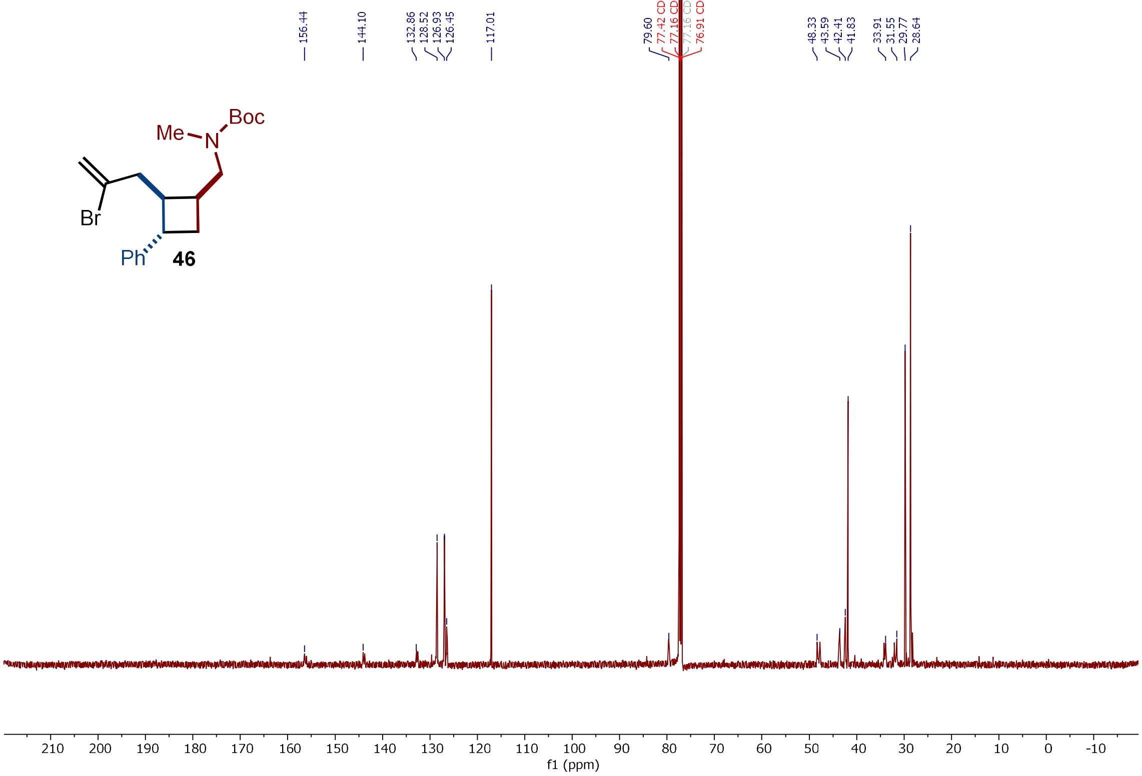

SA-1-759.10.fid

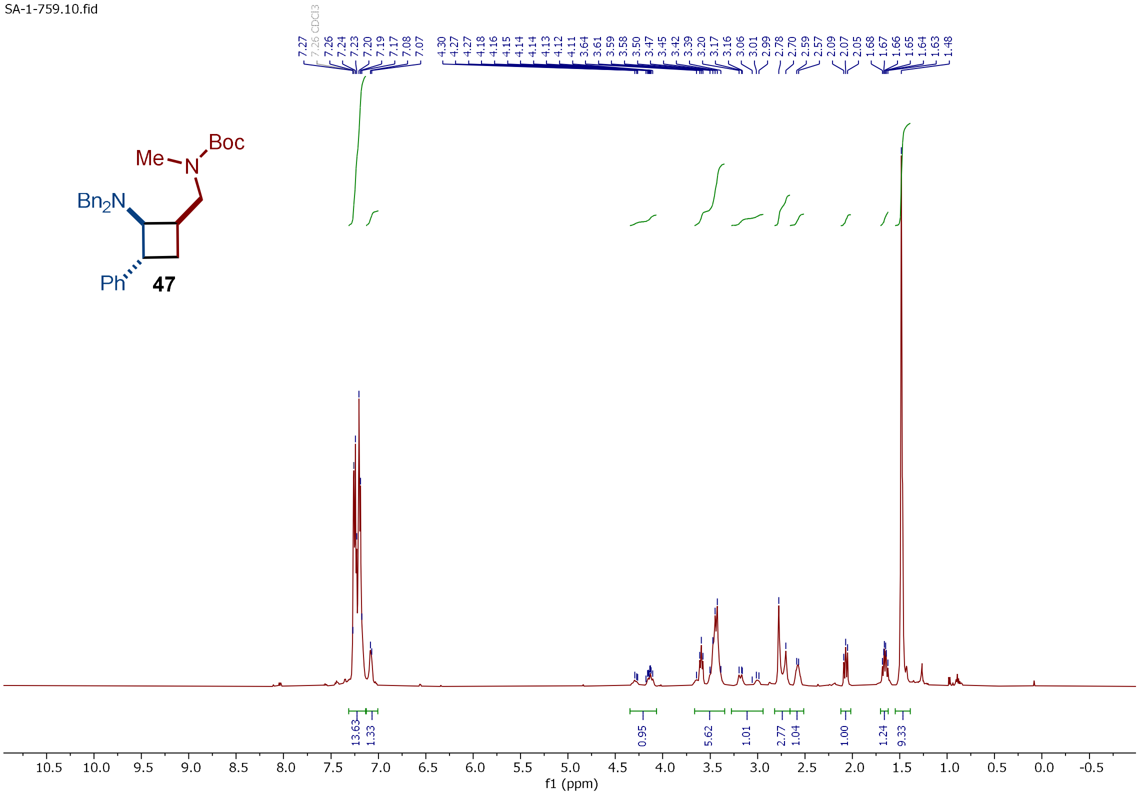

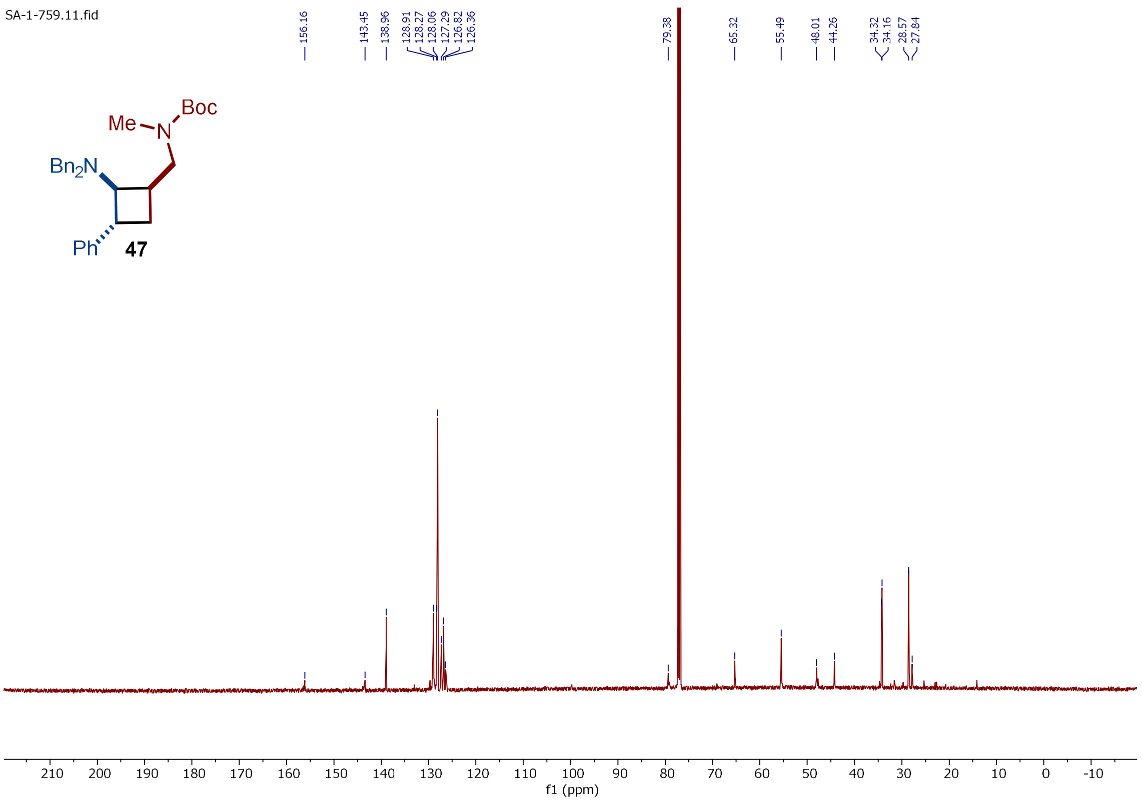
SA-1-759.11.fid
156.16
143.45
138.96
128.91
128.27
128.06
127.29
126.82
126.36
79.38
65.32
55.49
48.01
44.26
34.32
34.16
28.57
27.84
Me
N
Boc
Bn2N
Ph
47
210 200 190 180 170 160 150 140 130 120 110 100 90 80 70 60 50 40 30 20 10 0 -10
f1 (ppm)

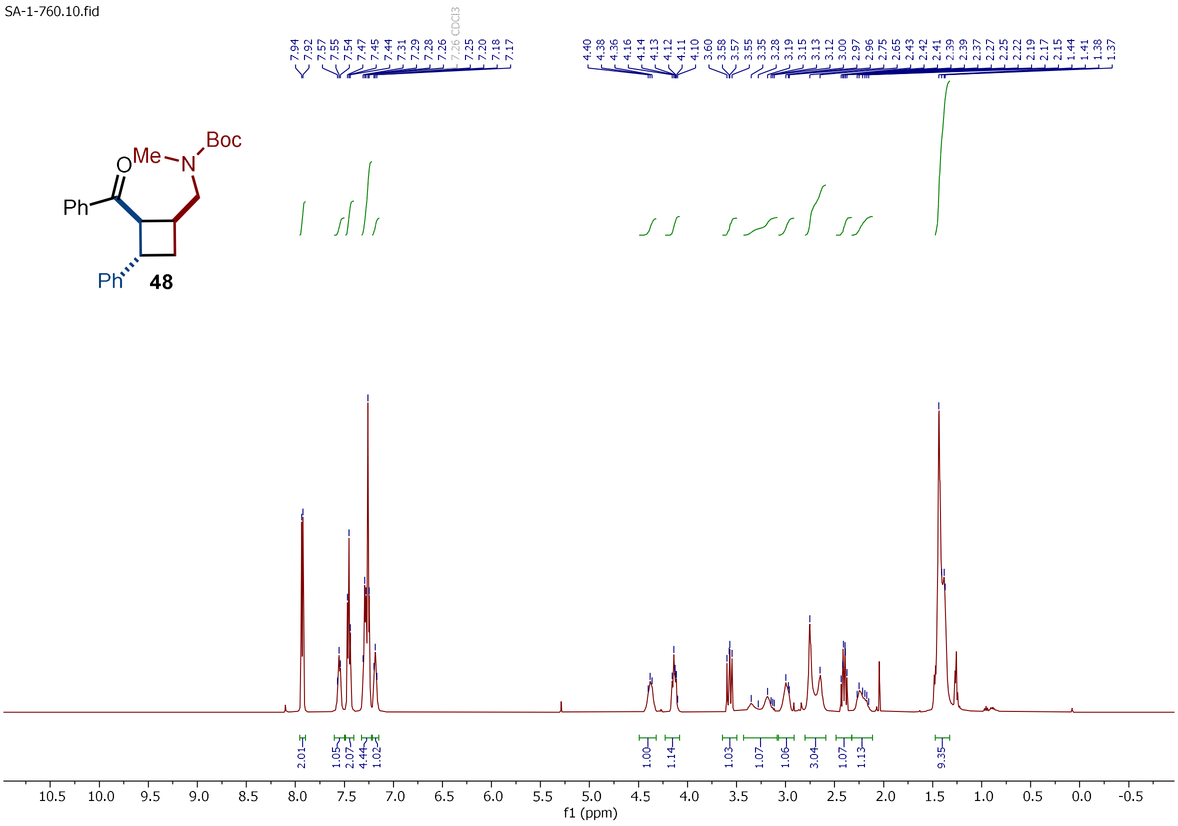
SA-1-760.10.fid
7.94
7.92
7.57
7.55
7.54
7.47
7.45
7.44
7.31
7.29
7.28
7.26 CDCl3
7.25
7.20
7.18
7.17
4.40
4.38
4.36
4.16
4.14
4.13
4.12
4.11
4.10
3.60
3.58
3.57
3.55
3.35
3.28
3.19
3.15
3.13
3.12
3.00
2.97
2.96
2.75
2.65
2.43
2.42
2.41
2.39
2.37
2.27
2.25
2.22
2.19
2.17
2.15
1.44
1.41
1.38
1.37
O
Me
N
Boc
Ph
Ph
48
2.01
1.05
2.07
4.44
1.02
1.00
1.14
1.03
1.07
1.06
3.04
1.07
1.13
9.35
10.5 10.0 9.5 9.0 8.5 8.0 7.5 7.0 6.5 6.0 5.5 5.0 4.5 4.0 3.5 3.0 2.5 2.0 1.5 1.0 0.5 0.0 -0.5
f1 (ppm)

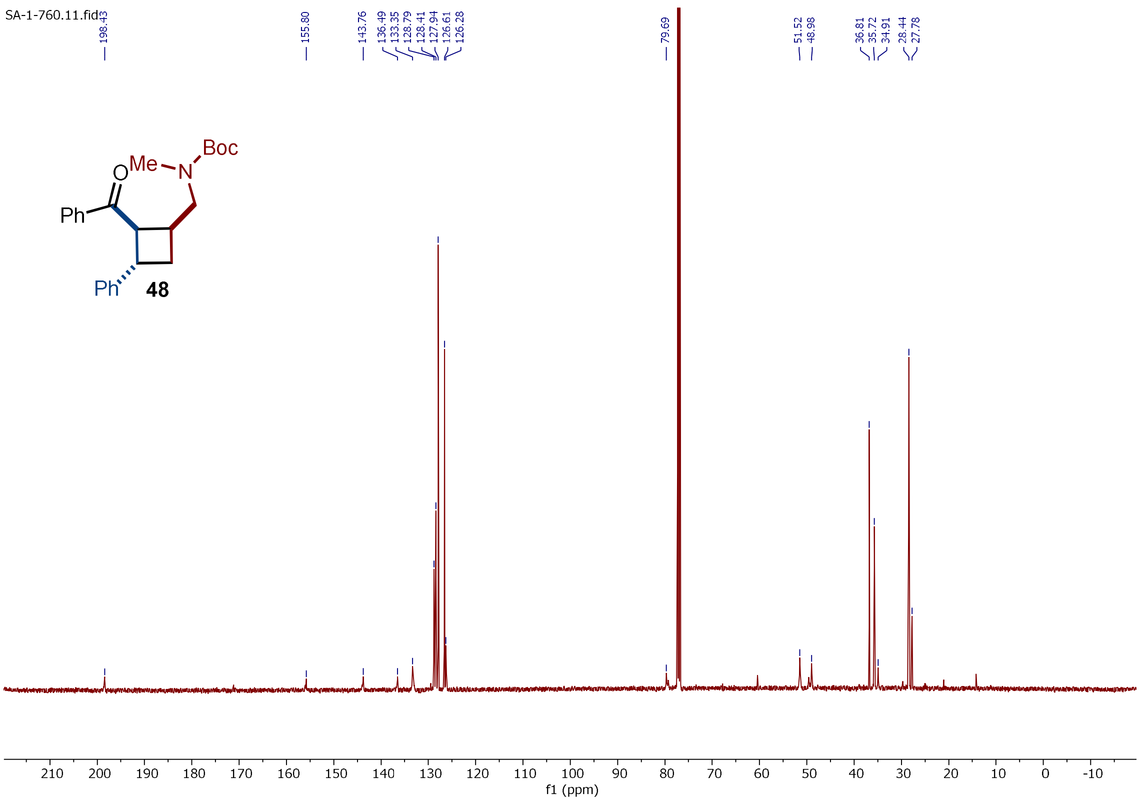
SA-1-760.11.fid
198.43
155.80
143.76
136.49
133.35
128.79
128.41
127.94
126.61
126.28
79.69
51.52
48.98
36.81
35.72
34.91
28.44
27.78
Me
N
Boc
O
Ph
Ph
48
f1 (ppm)

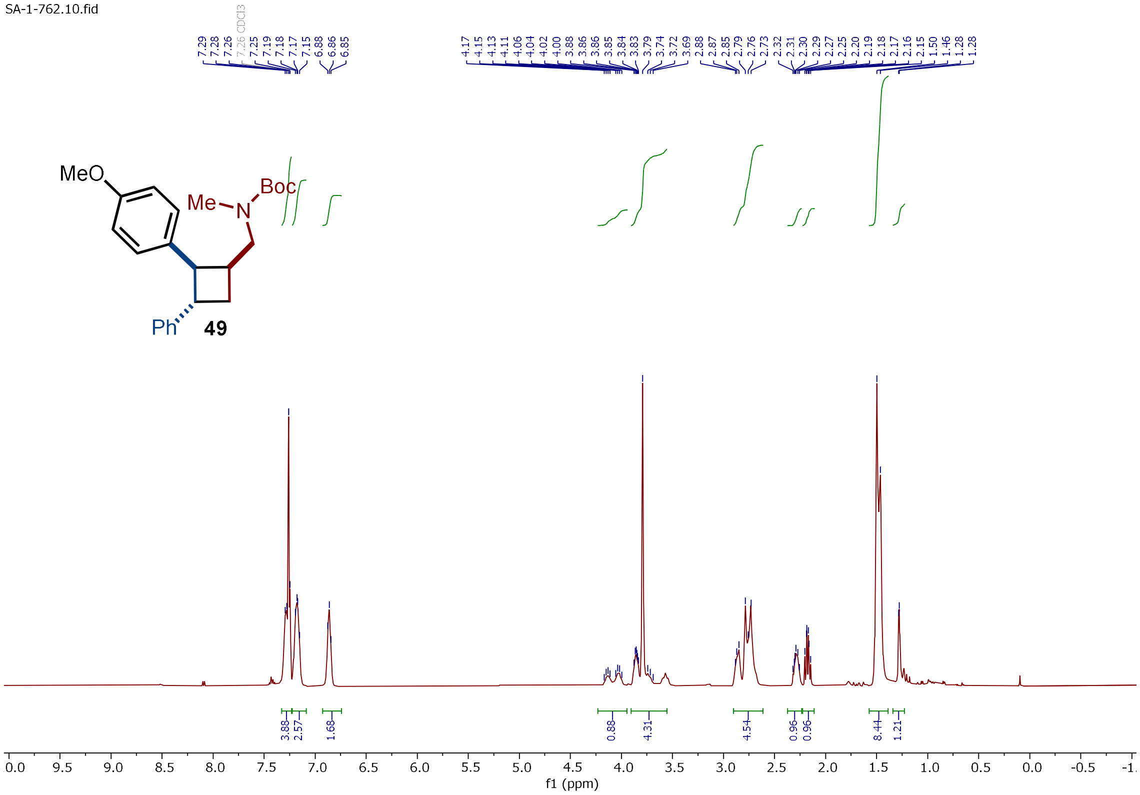
SA-1-762.10.fid
7.29
7.28
7.26 CDCl3
7.25
7.19
7.18
7.17
7.15
6.88
6.86
6.85
4.17
4.15
4.13
4.11
4.06
4.04
4.02
4.00
3.88
3.86
3.86
3.85
3.84
3.83
3.79
3.74
3.72
3.69
2.88
2.87
2.85
2.79
2.76
2.73
2.32
2.31
2.30
2.29
2.27
2.25
2.20
2.19
2.18
2.17
2.16
2.15
1.50
1.46
1.28
1.28
MeO
Me
N
Boc
Ph
49
3.88
2.57
1.68
0.88
4.31
4.54
0.96
0.96
8.44
1.21
f1 (ppm)

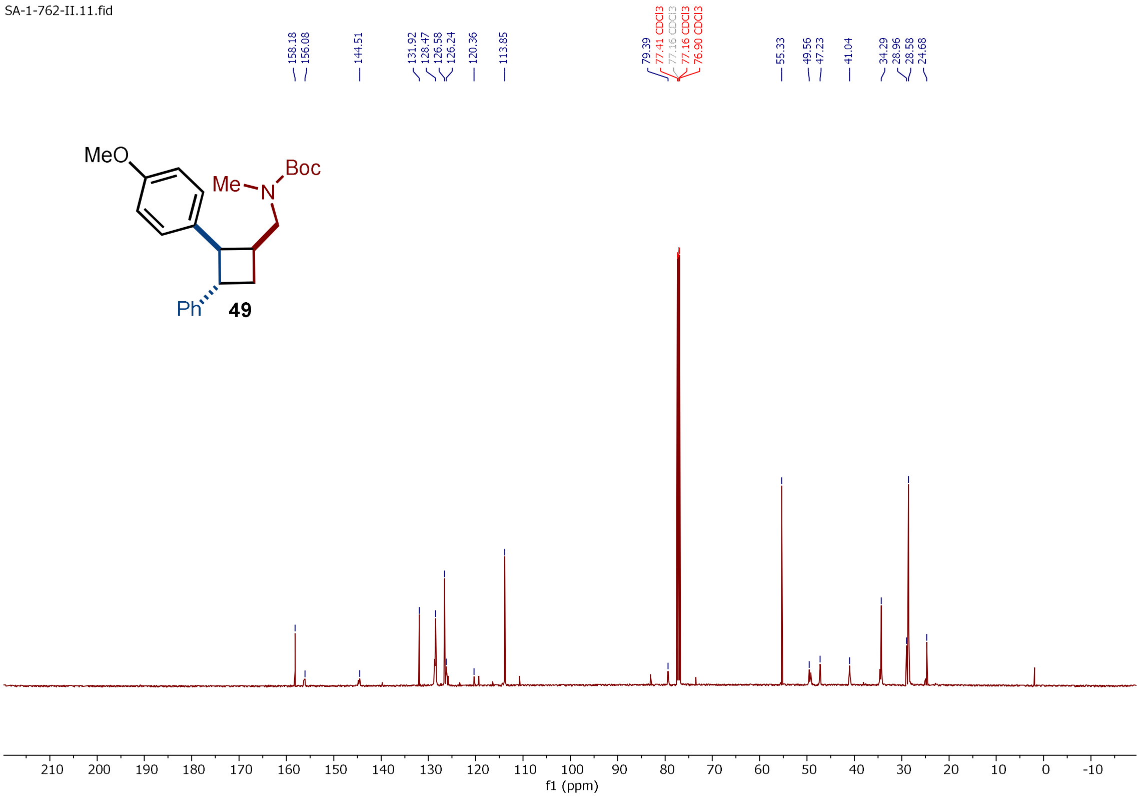
SA-1-762-II.11.fid
158.18
156.08
144.51
131.92
128.47
126.58
126.24
120.36
113.85
79.39
77.41 CDCl3
77.16 CDCl3
77.16 CDCl3
76.90 CDCl3
55.33
49.56
47.23
41.04
34.29
28.96
28.58
24.68
MeO
Me
N
Boc
Ph
49
210 200 190 180 170 160 150 140 130 120 110 100 90 80 70 60 50 40 30 20 10 0 -10
f1 (ppm)

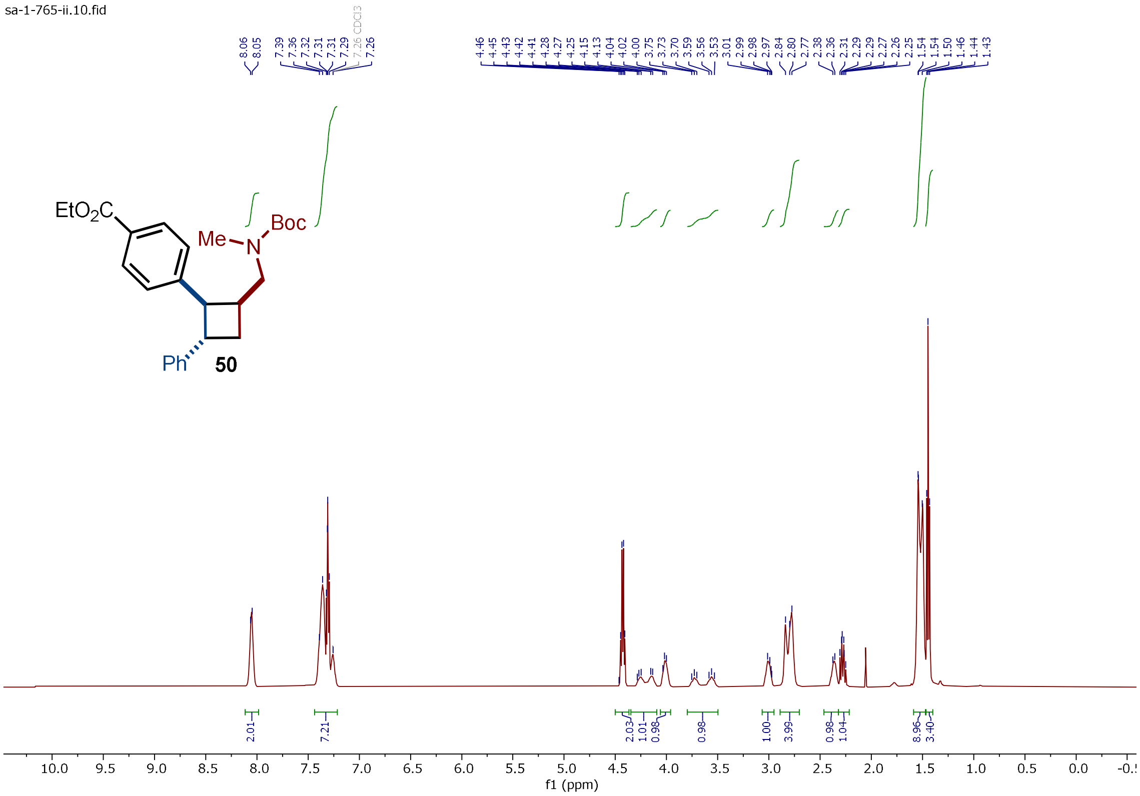
sa-1-765-ii.10.fid
8.06
8.05
7.39
7.36
7.32
7.31
7.31
7.29
7.26 CDCl3
7.26
4.46
4.45
4.43
4.42
4.41
4.28
4.27
4.25
4.15
4.13
4.04
4.02
4.00
3.75
3.73
3.70
3.59
3.56
3.53
3.01
2.99
2.98
2.97
2.84
2.80
2.77
2.38
2.36
2.31
2.29
2.29
2.27
2.26
2.25
1.54
1.54
1.50
1.46
1.44
1.43
EtO_2C
Me
N
Boc
Ph
50
2.01
7.21
2.03
1.01
0.98
0.98
1.00
3.99
0.98
1.04
8.96
3.40
10.0 9.5 9.0 8.5 8.0 7.5 7.0 6.5 6.0 5.5 5.0 4.5 4.0 3.5 3.0 2.5 2.0 1.5 1.0 0.5 0.0
f1 (ppm)

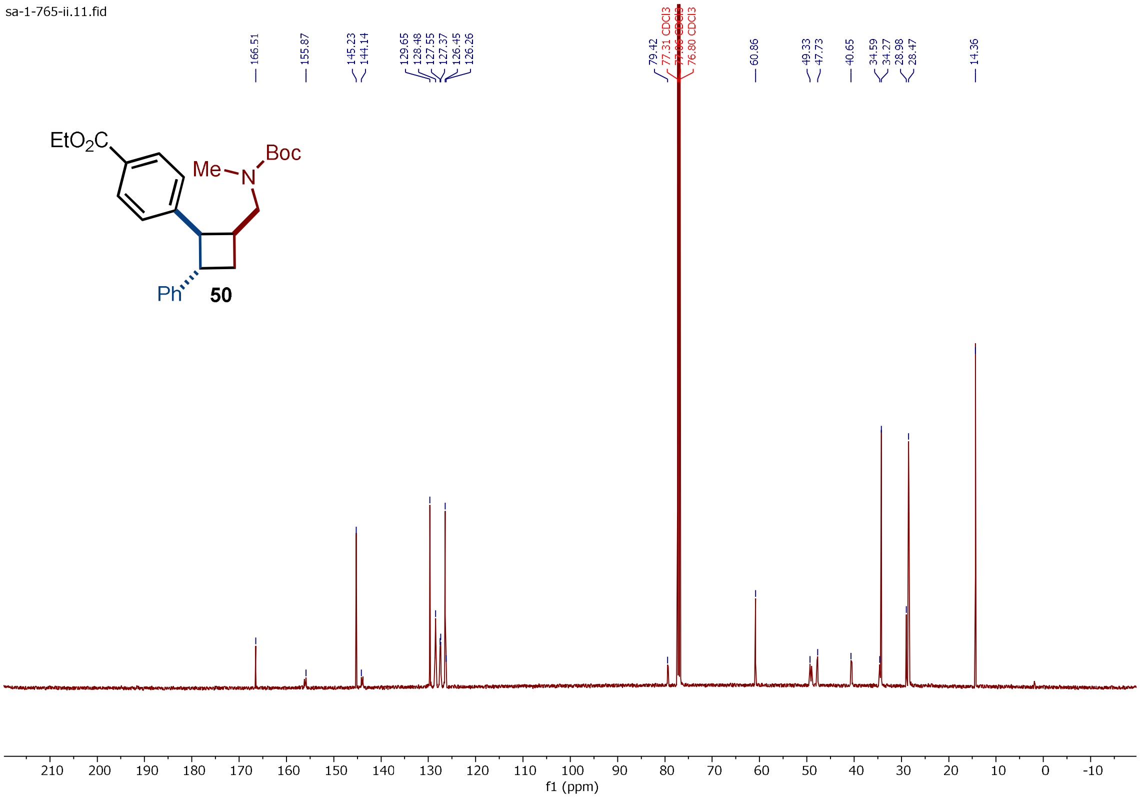
sa-1-765-ii.11.fid
166.51
155.87
145.23
144.14
129.65
128.48
127.55
127.37
126.45
126.26
79.42
77.31 CDCl3
77.06 CDCl3
76.80 CDCl3
60.86
49.33
47.73
40.65
34.59
34.27
28.98
28.47
14.36
EtO2C
Me
N
Boc
Ph
50
210 200 190 180 170 160 150 140 130 120 110 100 90 80 70 60 50 40 30 20 10 0 -10
f1 (ppm)

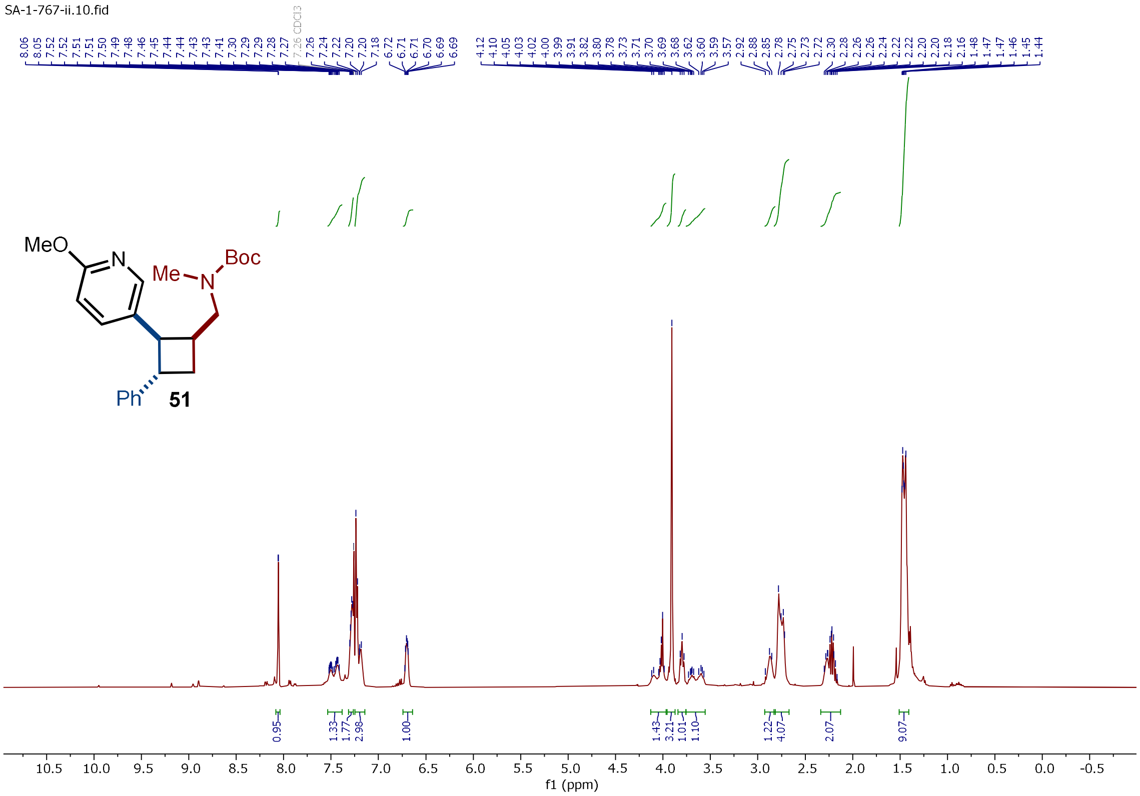
SA-1-767-ii.10.fid
8.06 8.05 7.52 7.52 7.51 7.51 7.50 7.49 7.48 7.46 7.45 7.44 7.44 7.43 7.43 7.41 7.30 7.29 7.29 7.28 7.27 7.26 CDCl3 7.26 7.24 7.22 7.22 7.20 7.20 7.18 6.72 6.71 6.71 6.70 6.69 6.69
4.12 4.10 4.05 4.03 4.02 4.00 3.99 3.91 3.82 3.80 3.78 3.73 3.71 3.70 3.69 3.68 3.62 3.60 3.59 3.57 2.92 2.88 2.85 2.78 2.75 2.73 2.72 2.30 2.28 2.26 2.24 2.22 2.22 2.20 2.20 2.18 2.16 1.48 1.47 1.46 1.45 1.44
MeO
N
Me
N
Boc
Ph
51
0.95 1.33 1.77 2.98 1.00 1.43 3.21 1.01 1.10 1.22 4.07 2.07 9.07
10.5 10.0 9.5 9.0 8.5 8.0 7.5 7.0 6.5 6.0 5.5 5.0 4.5 4.0 3.5 3.0 2.5 2.0 1.5 1.0 0.5 0.0 -0.5
f1 (ppm)

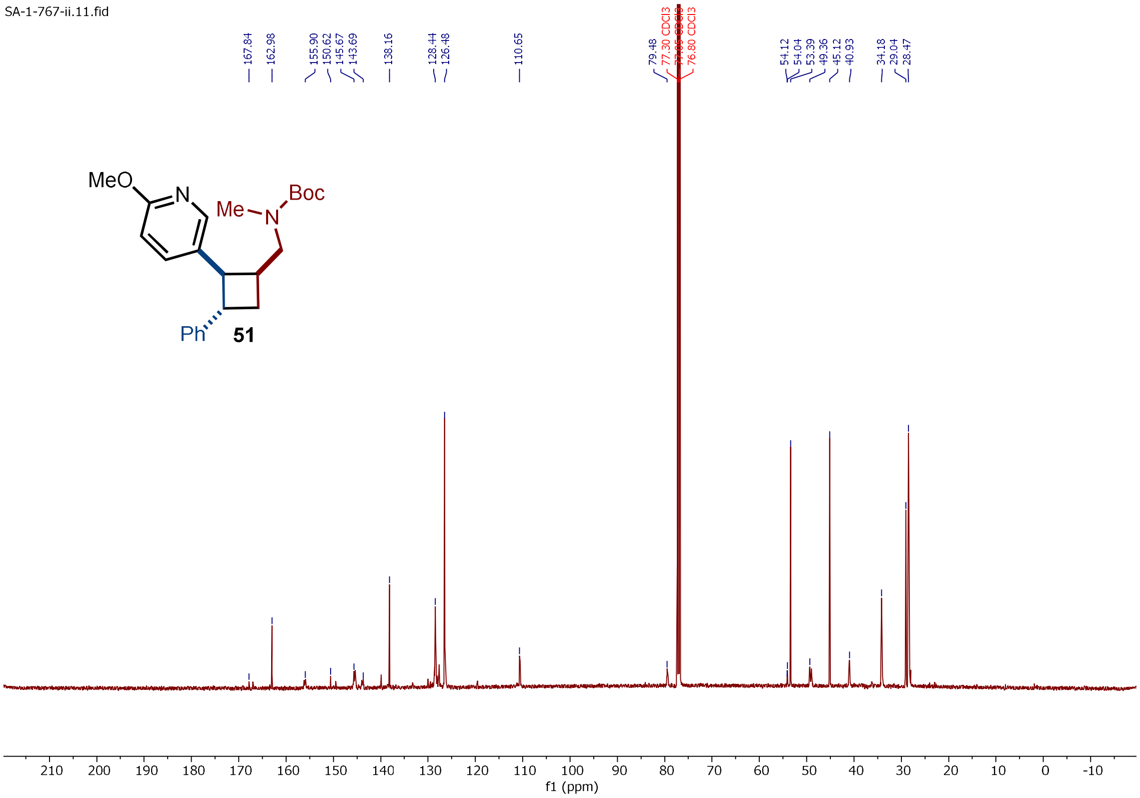
SA-1-767-ii.11.fid
167.84
162.98
155.90
150.62
145.67
143.69
138.16
128.44
126.48
110.65
79.48
77.30 CDCl3
76.80 CDCl3
54.12
54.04
53.39
49.36
45.12
40.93
34.18
29.04
28.47
MeO
N
Me
N
Boc
Ph
51
f1 (ppm)

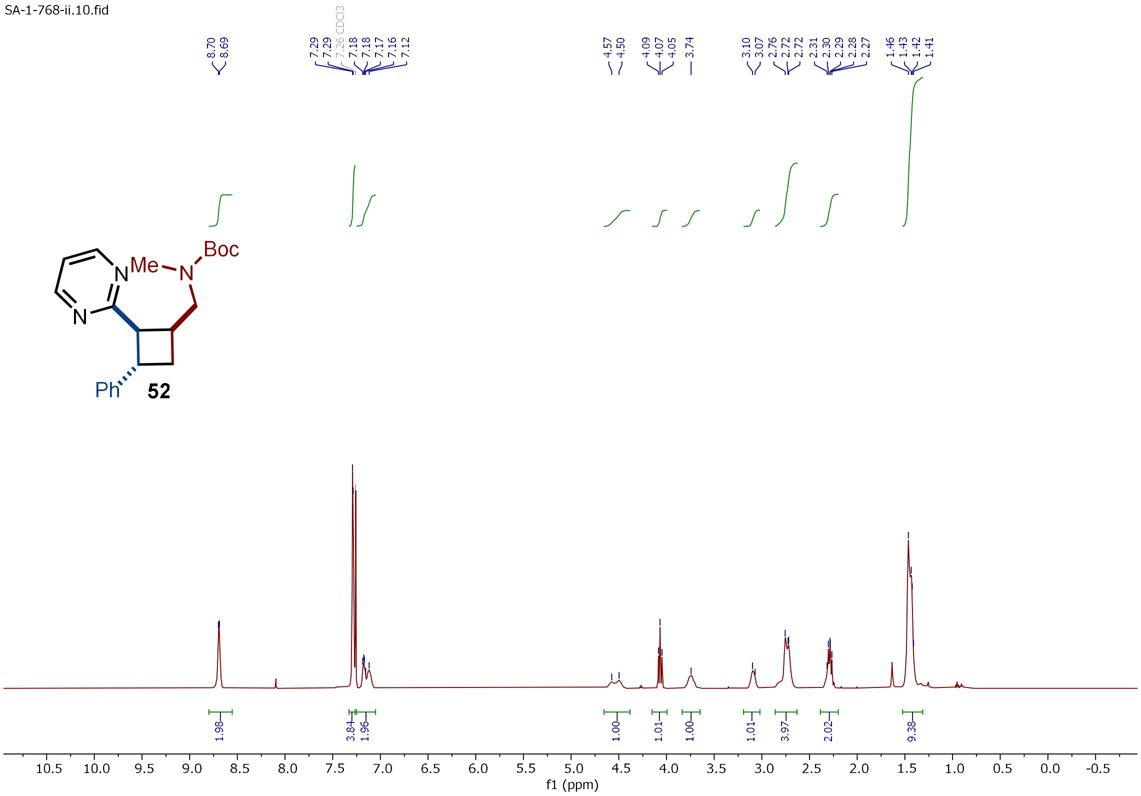
SA-1-768-ii.10.fid
8.70
8.69
7.29
7.29
7.26 CDCl3
7.18
7.18
7.17
7.16
7.12
4.57
4.50
4.09
4.07
4.05
3.74
3.10
3.07
2.76
2.72
2.72
2.31
2.30
2.29
2.28
2.27
1.46
1.43
1.42
1.41
N
N
Me
N
Boc
Ph
52
1.98
3.84
1.96
1.00
1.01
1.00
1.01
3.97
2.02
9.38
f1 (ppm)

SA-1-768-ii.20.fid

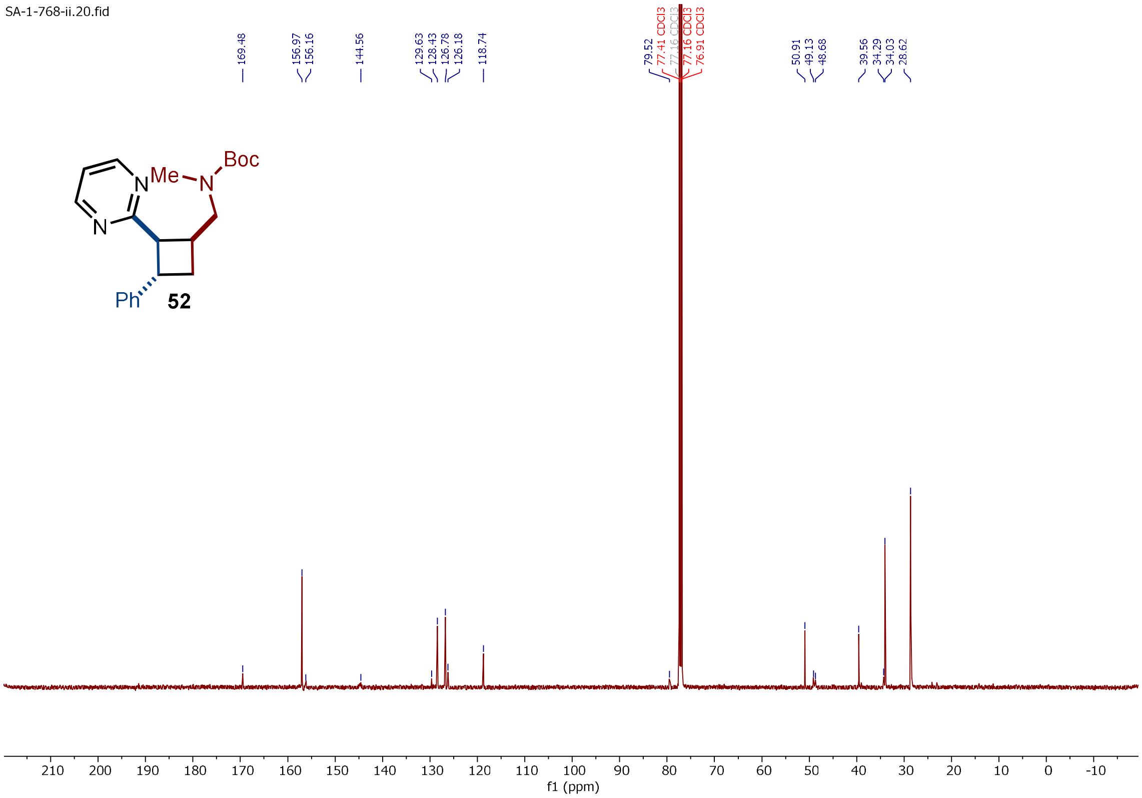

SA-1-828.10.fid

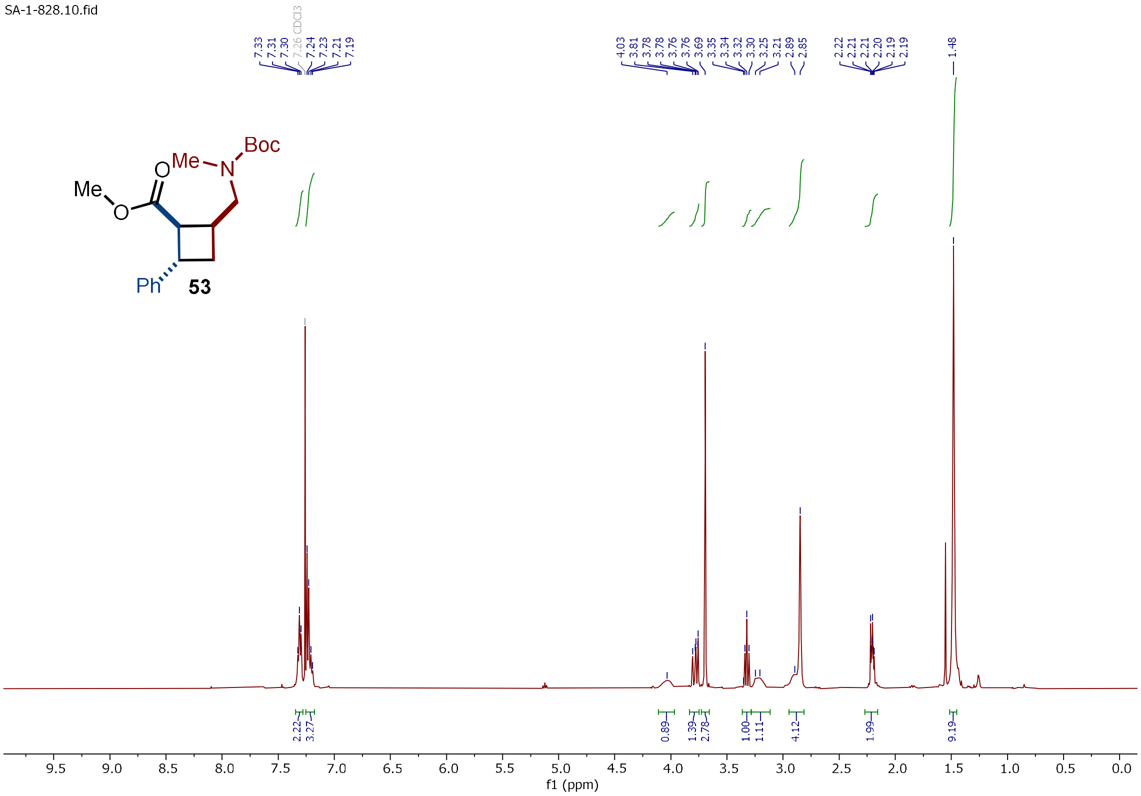

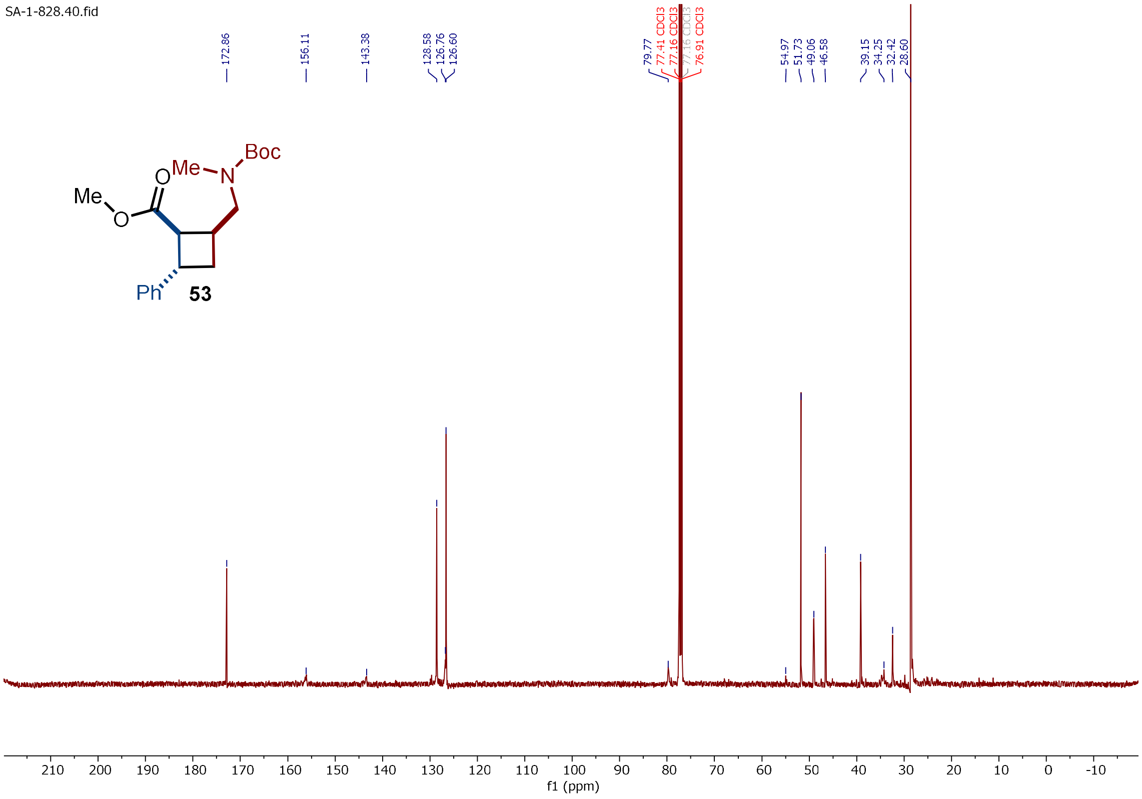
SA-1-828.40.fid
172.86
156.11
143.38
128.58
126.76
126.60
79.77
77.41 CDCl3
77.16 CDCl3
76.91 CDCl3
54.97
51.73
49.06
46.58
39.15
34.25
32.42
28.60
Me
O
O
Me
N
Boc
Ph
53
f1 (ppm)

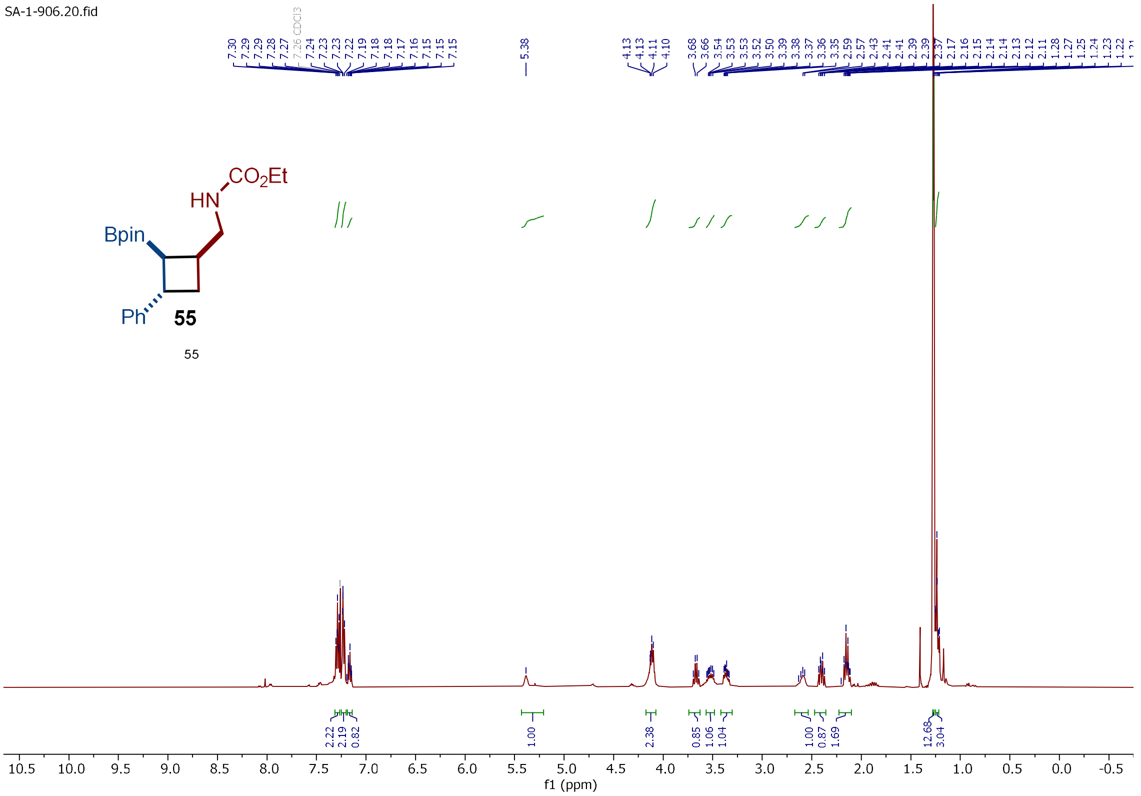
SA-1-906.20.fid
7.30
7.29
7.29
7.28
7.27
7.26 CDCl3
7.24
7.23
7.23
7.22
7.19
7.18
7.18
7.17
7.16
7.15
7.15
7.15
5.38
4.13
4.13
4.11
4.10
3.68
3.66
3.54
3.53
3.53
3.52
3.50
3.39
3.38
3.37
3.36
3.35
2.59
2.57
2.43
2.41
2.41
2.39
2.39
2.37
2.17
2.16
2.15
2.14
2.13
2.12
2.11
1.28
1.27
1.25
1.24
1.23
1.22
HN
CO2Et
Bpin
Ph
55
55
2.22
2.19
0.82
1.00
2.38
0.85
1.06
1.04
1.00
0.87
1.69
12.68
3.04
f1 (ppm)

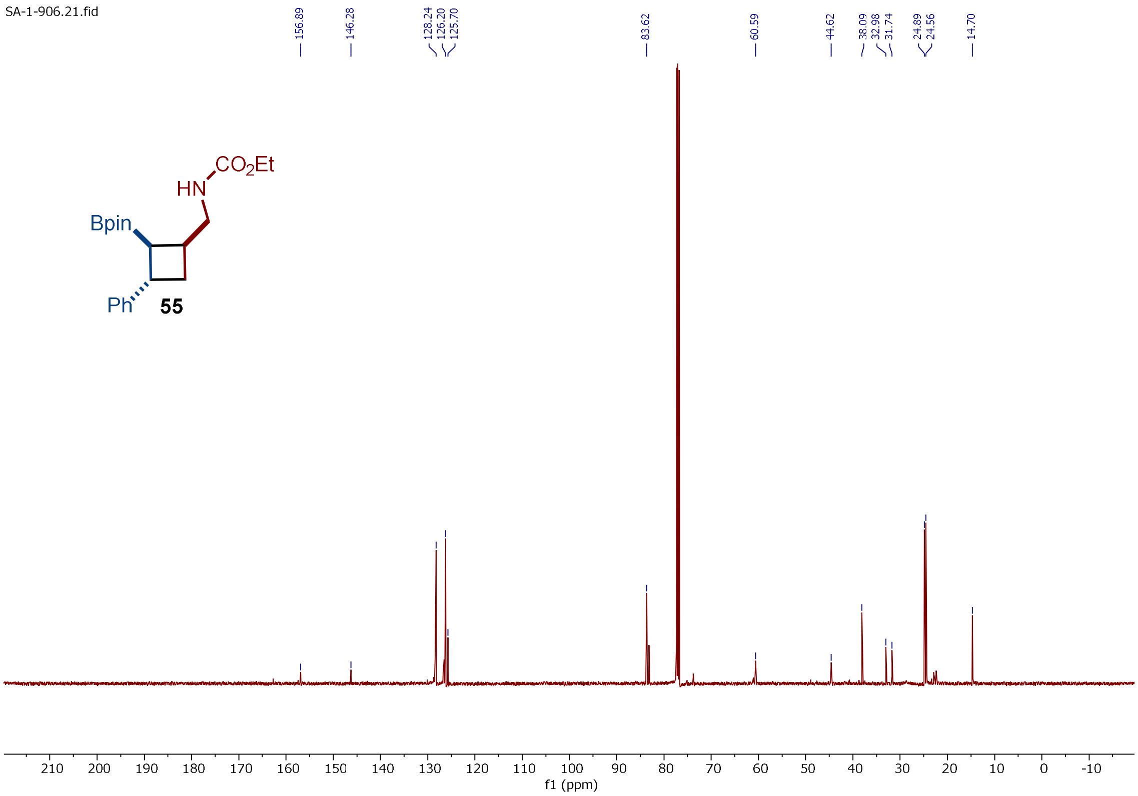
SA-1-906.21.fid
156.89
146.28
128.24
126.20
125.70
83.62
60.59
44.62
38.09
32.98
31.74
24.89
24.56
14.70
HN
CO_2Et
Bpin
Ph
55
210 200 190 180 170 160 150 140 130 120 110 100 90 80 70 60 50 40 30 20 10 0 -10
f1 (ppm)

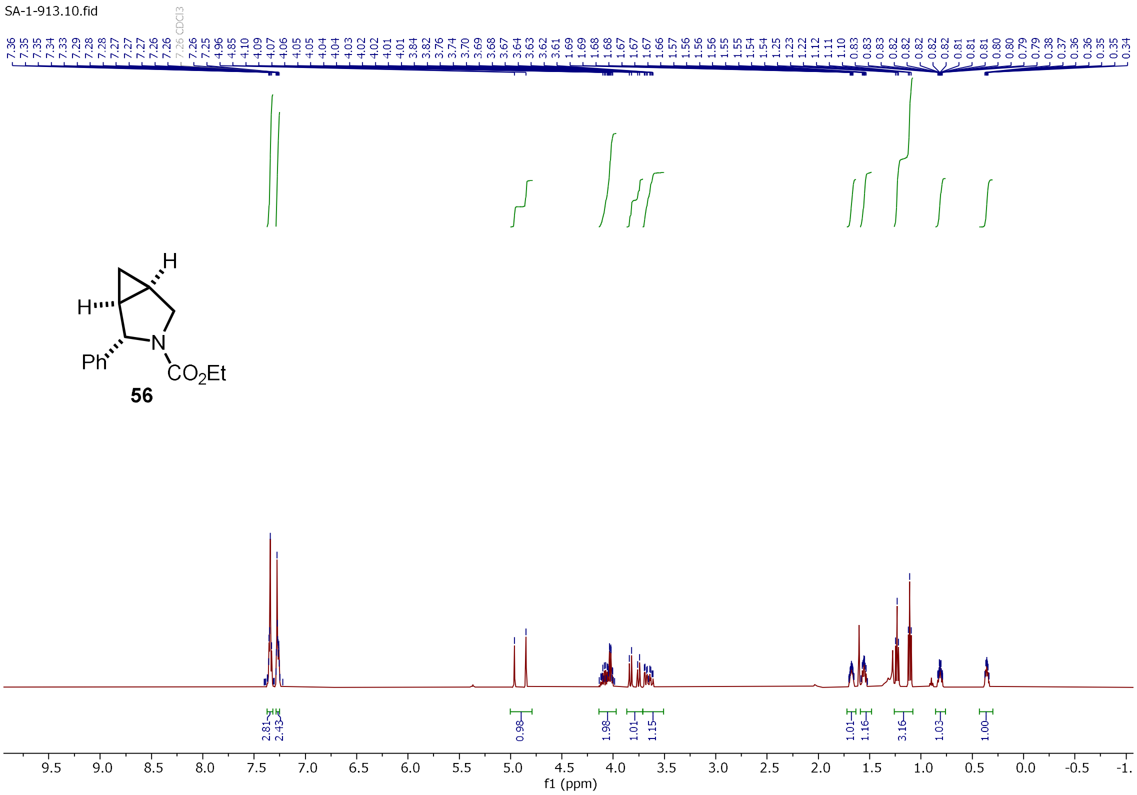
SA-1-913.10.fid
7.36 7.35 7.35 7.34 7.33 7.29 7.28 7.28 7.27 7.27 7.27 7.26 7.26 7.26 CDCl3 7.26 7.25 4.96 4.85 4.10 4.09 4.07 4.06 4.05 4.05 4.04 4.04 4.03 4.02 4.02 4.01 4.01 3.84 3.82 3.76 3.74 3.70 3.69 3.68 3.67 3.64 3.63 3.62 3.61 1.69 1.69 1.68 1.68 1.67 1.67 1.67 1.66 1.57 1.56 1.56 1.56 1.55 1.55 1.54 1.54 1.25 1.23 1.22 1.12 1.11 1.10 0.83 0.83 0.83 0.82 0.82 0.82 0.82 0.82 0.81 0.81 0.81 0.80 0.80 0.79 0.38 0.37 0.36 0.36 0.35 0.34
H
H
Ph
N
CO_2Et
56
2.81
2.43
0.98
1.98
1.01
1.15
1.01
1.16
3.16
1.03
1.00
9.5 9.0 8.5 8.0 7.5 7.0 6.5 6.0 5.5 5.0 4.5 4.0 3.5 3.0 2.5 2.0 1.5 1.0 0.5 0.0 -0.5 -1.
f1 (ppm)

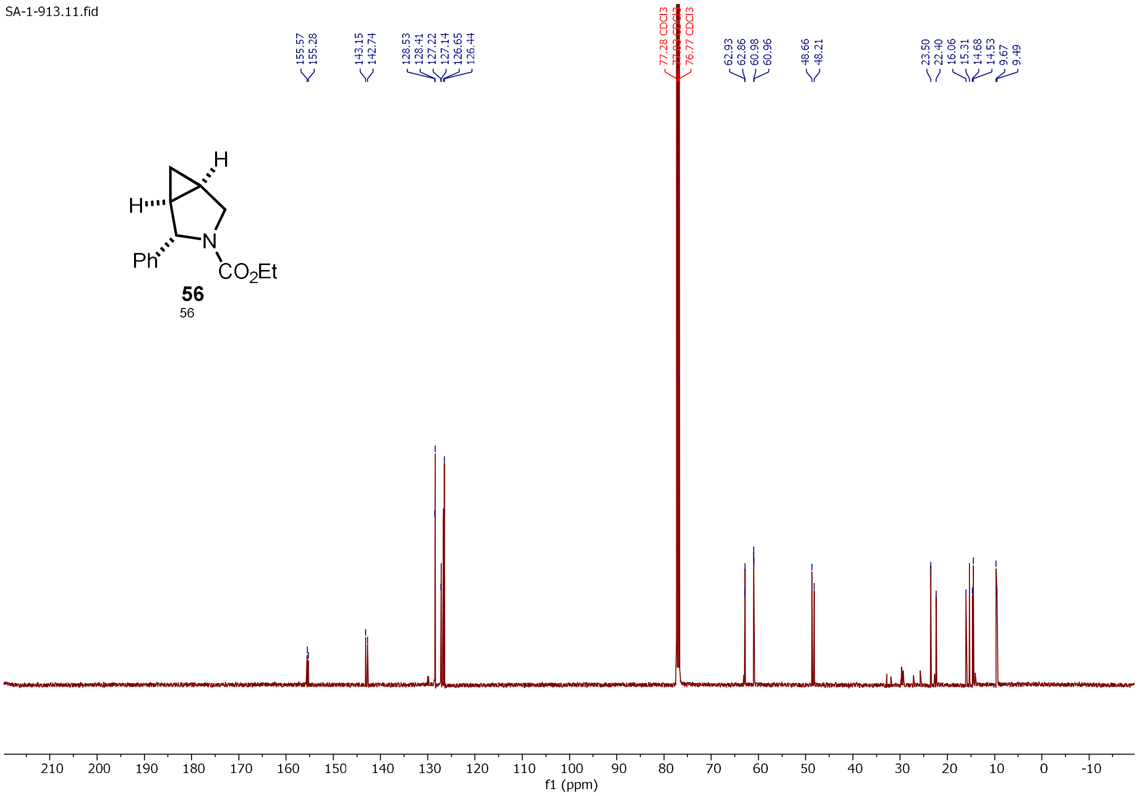
SA-1-913.11.fid
155.57
155.28
143.15
142.74
128.53
128.41
127.22
127.14
126.65
126.44
77.28 CDCl3
76.77 CDCl3
62.93
62.86
60.98
60.96
48.66
48.21
23.50
22.40
16.06
15.31
14.68
14.53
9.67
9.49
56
56
f1 (ppm)

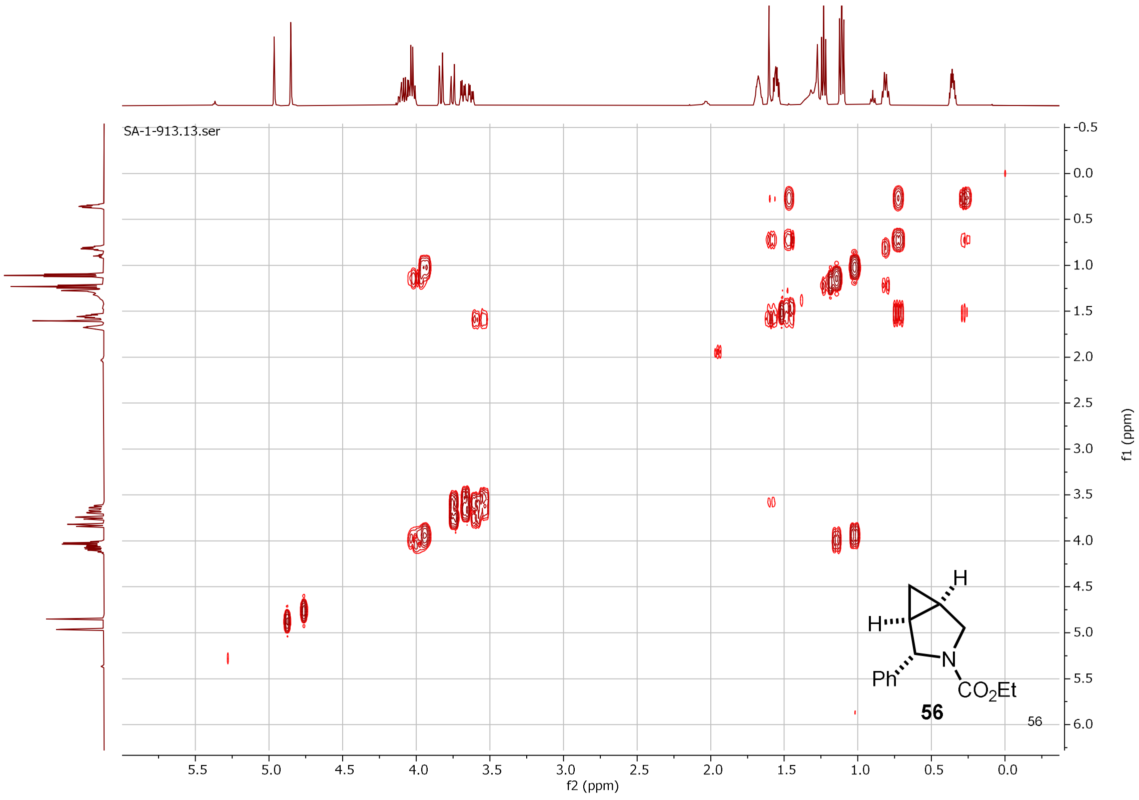
SA-1-913.13.ser
56
56
f2 (ppm)
f1 (ppm)

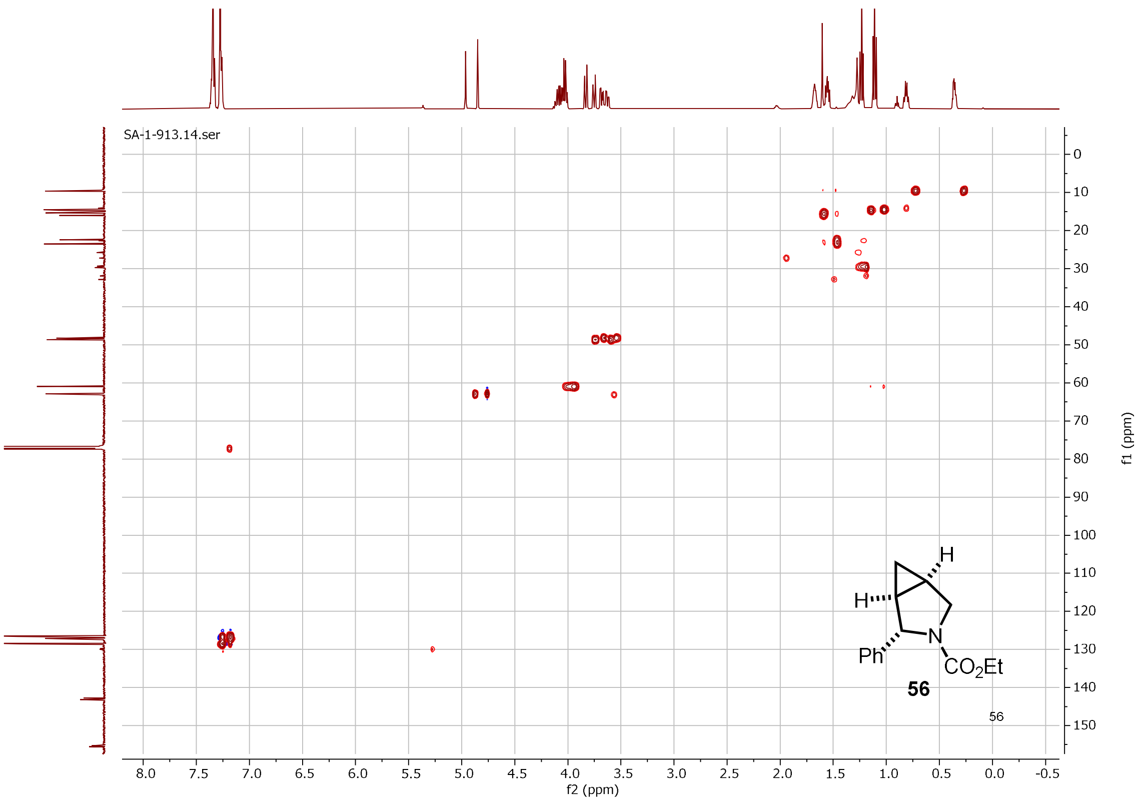
SA-1-913.14.ser
f2 (ppm)
f1 (ppm)
H
H
Ph
N
CO2Et
56
56

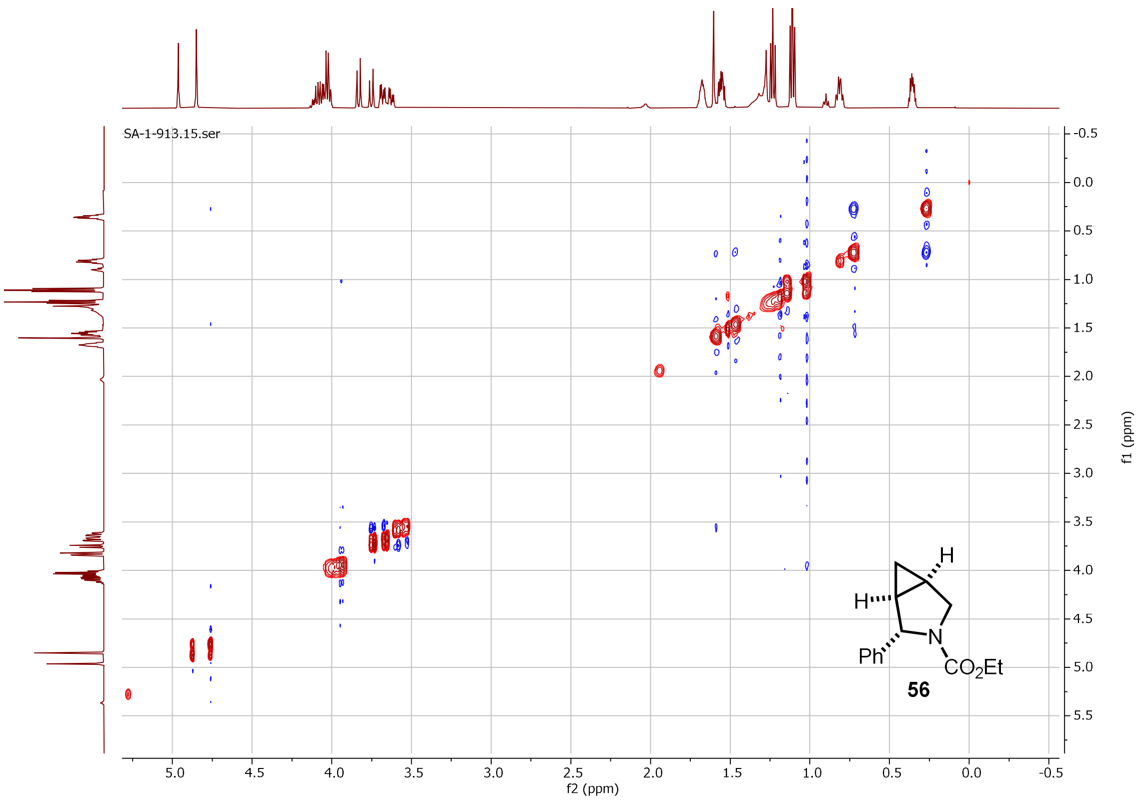
SA-1-913.15.ser
f2 (ppm)
f1 (ppm)
H
H
Ph
N
CO2Et
56

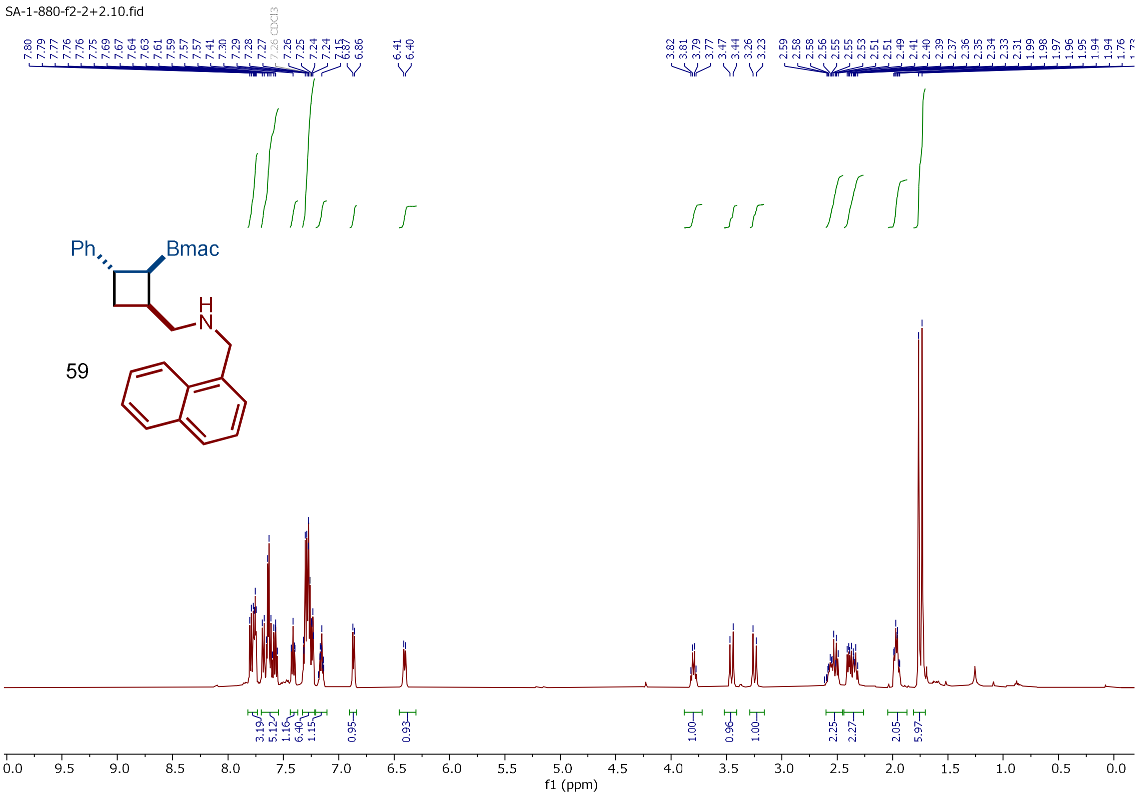
SA-1-880-f2-2+2.10.fid
7.80 7.79 7.77 7.76 7.76 7.75 7.69 7.67 7.64 7.63 7.61 7.59 7.57 7.57 7.41 7.30 7.29 7.28 7.27 7.26 CDCl3 7.26 7.25 7.24 7.24 7.15 6.87 6.86 6.41 6.40
3.82 3.81 3.79 3.77 3.47 3.44 3.26 3.23 2.59 2.58 2.58 2.56 2.55 2.55 2.53 2.51 2.51 2.49 2.41 2.40 2.39 2.37 2.36 2.35 2.34 2.33 2.31 1.99 1.98 1.97 1.96 1.95 1.94 1.94 1.76
Ph
Bmac
H
N
59
3.19 5.12 1.16 6.40 1.15 0.95 0.93 1.00 0.96 1.00 2.25 2.27 2.05 5.97
f1 (ppm)

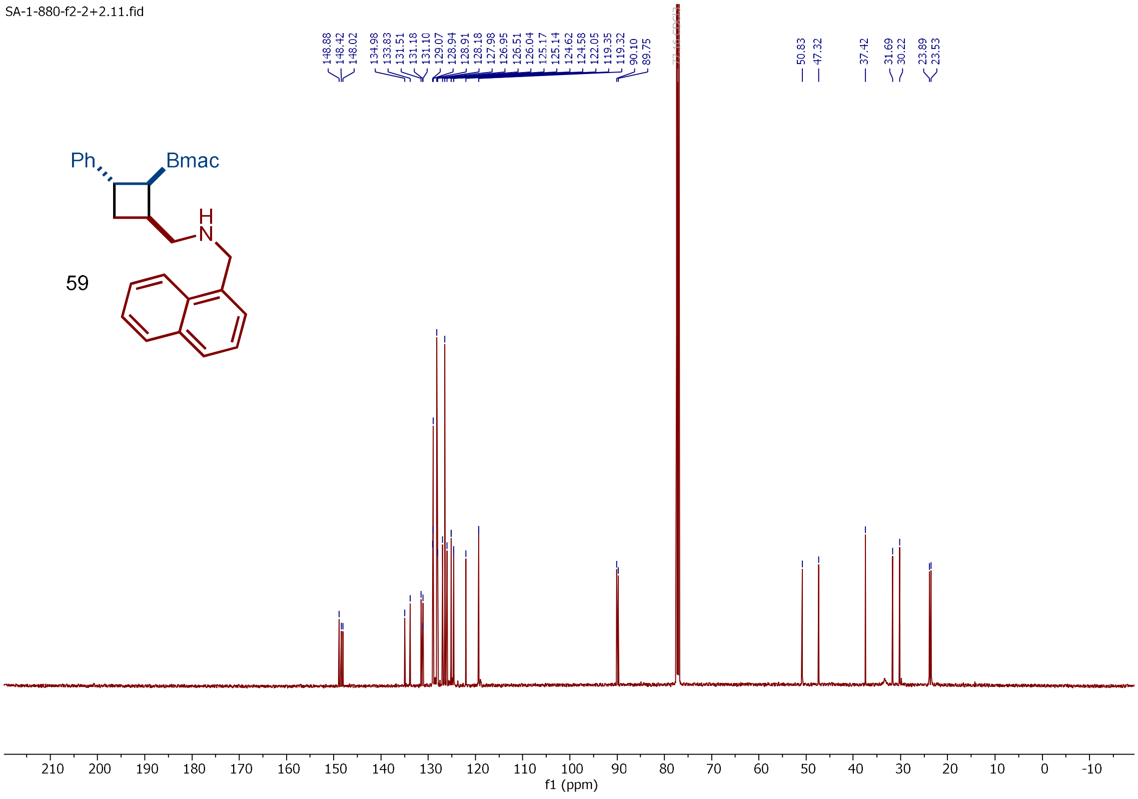
SA-1-880-f2-2+2.11.fid
148.88 148.42 148.02 134.98 133.83 131.51 131.18 131.10 129.07 128.94 128.91 128.18 127.98 126.95 126.51 126.04 125.17 125.14 124.62 124.58 122.05 119.35 119.32 90.10 89.75
50.83 47.32 37.42 31.69 30.22 23.89 23.53
Ph
Bmac
H
N
59
f1 (ppm)

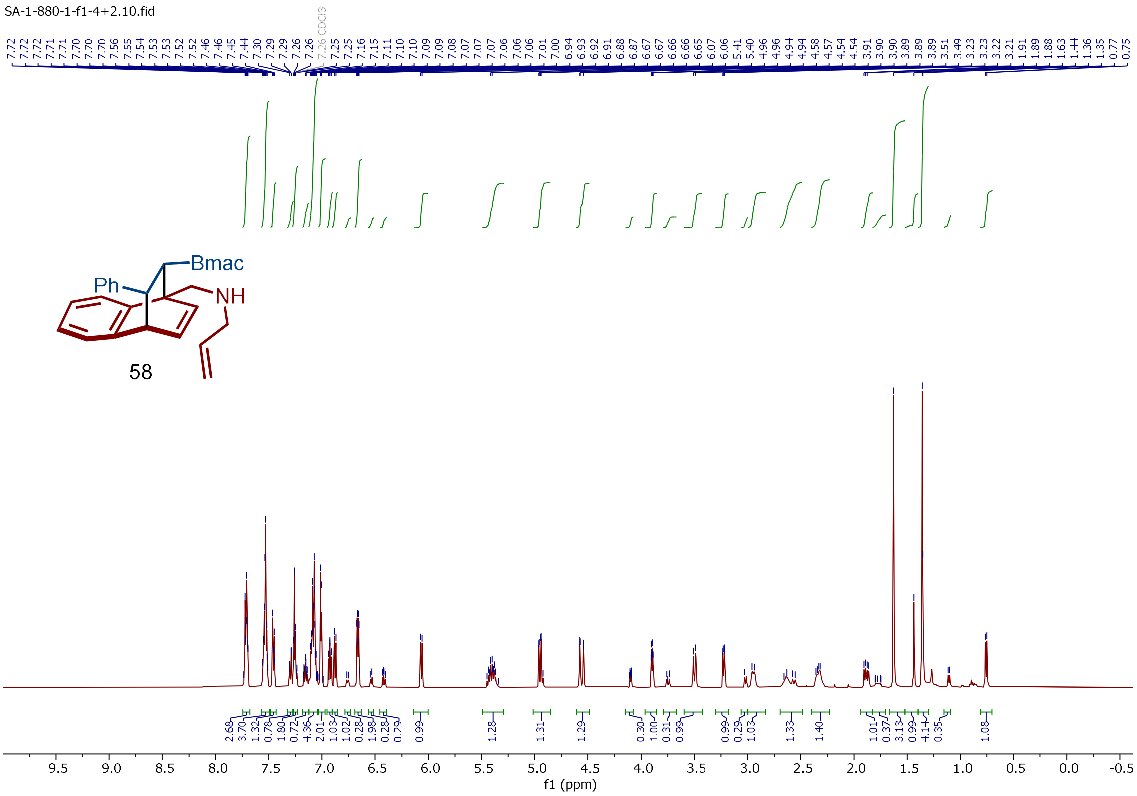
SA-1-880-1-f1-4+2.10.fid
7.72 7.72 7.72 7.71 7.71 7.70 7.70 7.56 7.55 7.54 7.53 7.53 7.52 7.52 7.46 7.45 7.44 7.30 7.29 7.29 7.26 7.26 CDCl3 7.25 7.25 7.16 7.15 7.11 7.10 7.10 7.09 7.09 7.08 7.07 7.07 7.07 7.06 7.06 7.01 7.00 6.94 6.93 6.92 6.91 6.88 6.87 6.67 6.67 6.66 6.66 6.65 6.07 6.06 5.41 5.40 4.96 4.96 4.94 4.94 4.58 4.57 4.54 4.54 3.91 3.90 3.90 3.89 3.89 3.51 3.49 3.23 3.22 3.21 1.91 1.89 1.88 1.63 1.44 1.36 1.35 0.77 0.75
Bmac
Ph
NH
58
2.68 3.70 1.32 0.78 1.80 0.72 4.36 2.01 1.03 1.02 1.98 0.28 0.28 0.29 0.99 1.28 1.31 1.29 0.30 1.00 0.31 0.99 0.99 0.29 1.03 1.33 1.40 1.01 0.37 3.13 0.99 4.14 0.35 1.08
9.5 9.0 8.5 8.0 7.5 7.0 6.5 6.0 5.5 5.0 4.5 4.0 3.5 3.0 2.5 2.0 1.5 1.0 0.5 0.0 -0.5
f1 (ppm)

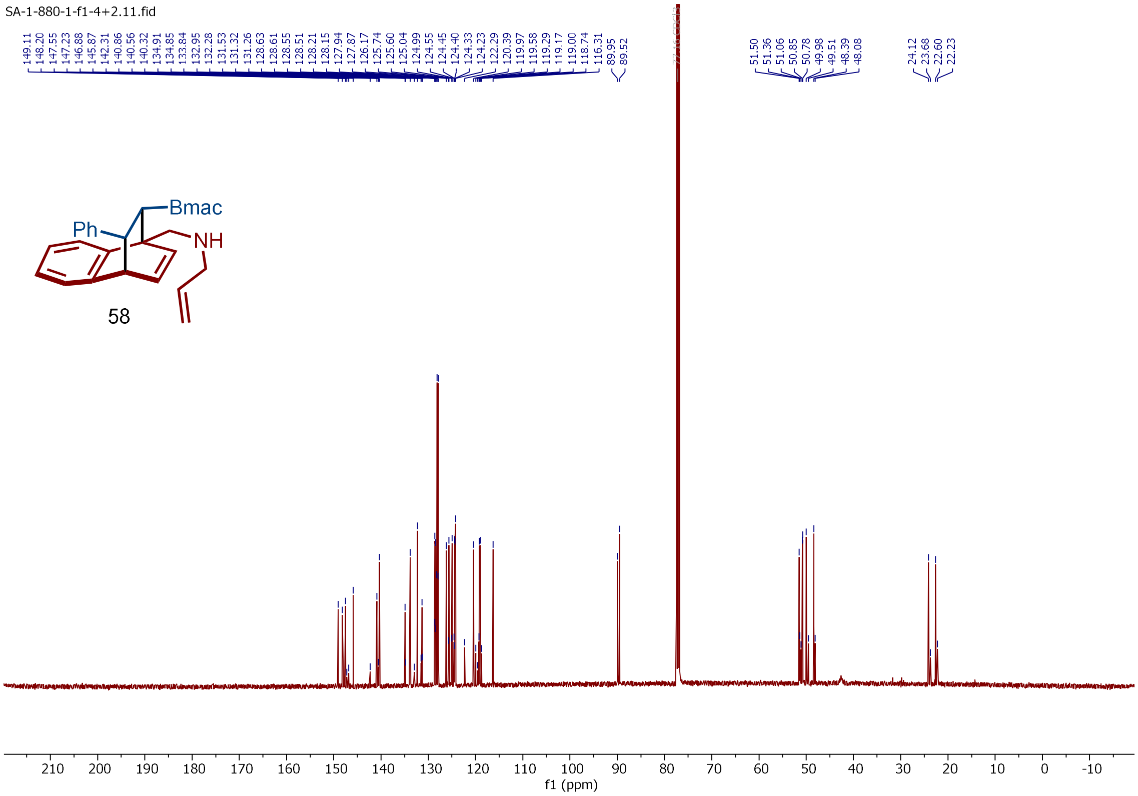
SA-1-880-1-f1-4+2.11.fid
149.11 148.20 147.55 147.23 146.88 145.87 142.31 140.86 140.56 140.32 134.91 134.85 133.84 132.95 132.28 131.53 131.32 131.26 128.63 128.61 128.55 128.51 128.21 128.15 127.94 127.87 126.17 125.74 125.60 125.04 124.99 124.55 124.45 124.40 124.33 124.23 122.29 120.39 119.97 119.58 119.29 119.17 119.00 118.74 116.31 89.95 89.52
51.50 51.36 51.06 50.85 50.78 49.98 49.51 48.39 48.08
24.12 23.68 22.60 22.23
Bmac
Ph
NH
58
210 200 190 180 170 160 150 140 130 120 110 100 90 80 70 60 50 40 30 20 10 0 -10
f1 (ppm)

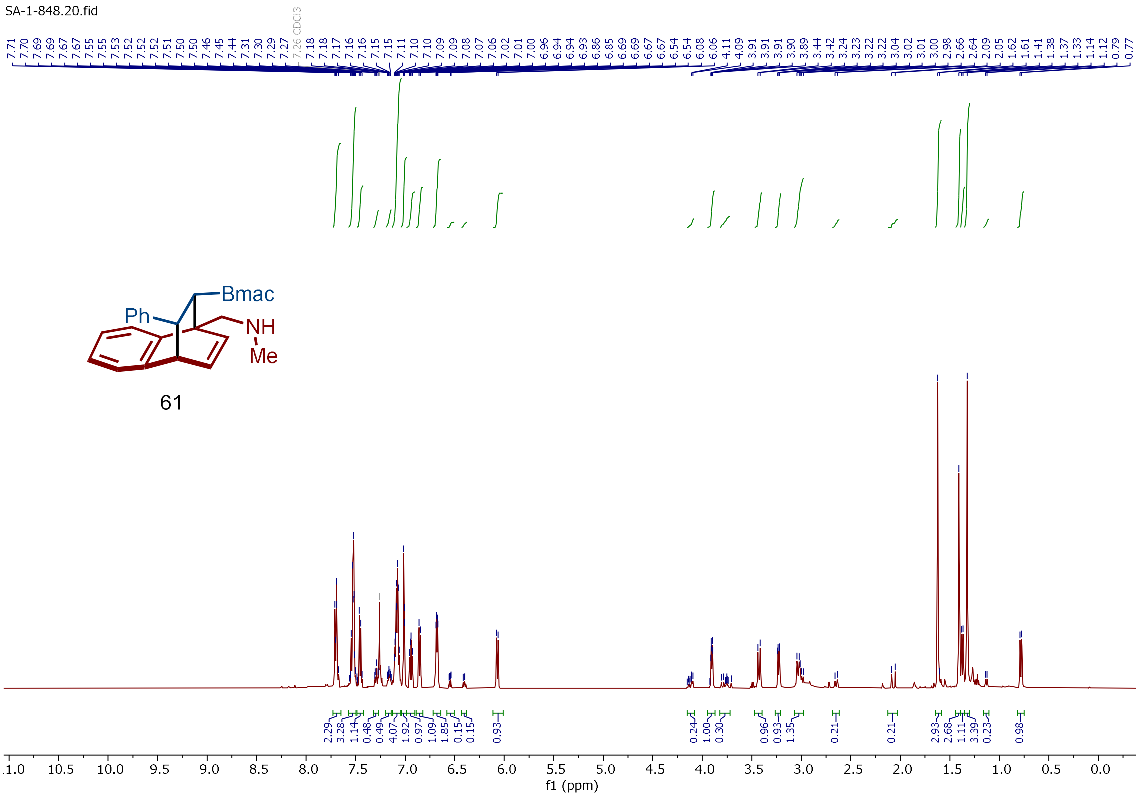
SA-1-848.20.fid
7.71 7.70 7.69 7.69 7.67 7.67 7.55 7.55 7.53 7.52 7.52 7.51 7.50 7.50 7.46 7.45 7.44 7.31 7.30 7.29 7.27 7.26 CDCl3 7.18 7.18 7.17 7.16 7.16 7.15 7.15 7.11 7.10 7.09 7.09 7.08 7.07 7.06 7.02 7.01 7.00 6.96 6.94 6.94 6.93 6.86 6.85 6.69 6.69 6.67 6.67 6.54 6.54 6.08 6.06 4.11 4.09 3.91 3.91 3.91 3.90 3.89 3.44 3.42 3.24 3.23 3.22 3.22 3.04 3.02 3.01 3.00 2.98 2.66 2.64 2.09 2.05 1.62 1.61 1.41 1.38 1.37 1.33 1.14 1.12 0.79 0.77
Bmac
Ph
NH
Me
61
2.29 3.28 1.14 0.48 0.49 4.07 1.92 0.97 1.09 1.85 0.15 0.15 0.93 0.24 1.00 0.30 0.96 0.93 1.35 0.21 0.21 2.93 2.68 1.11 3.39 0.23 0.98
f1 (ppm)

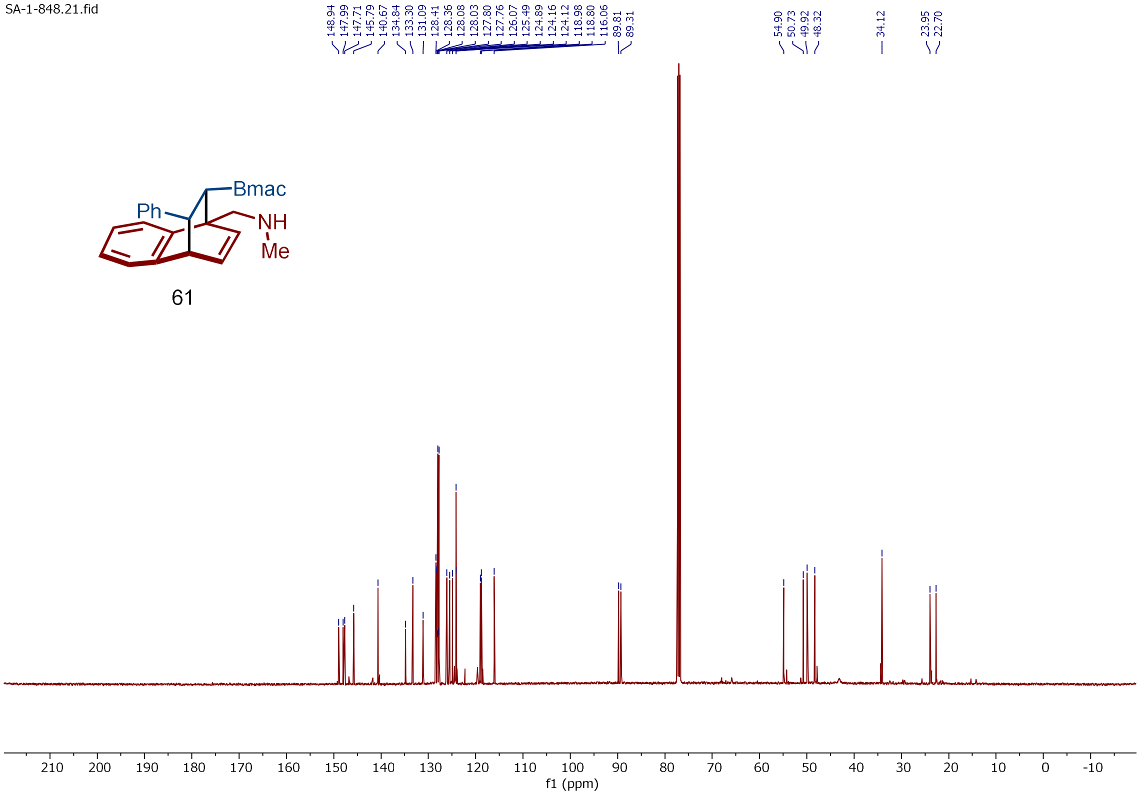
SA-1-848.21.fid
148.94 147.99 147.71 145.79 140.67 134.84 133.30 131.09 128.41 128.36 128.08 128.03 127.80 127.76 126.07 125.49 124.89 124.16 124.12 118.98 118.80 116.06 89.81 89.31
54.90 50.73 49.92 48.32
34.12
23.95 22.70
Bmac
Ph
NH
Me
61
f1 (ppm)

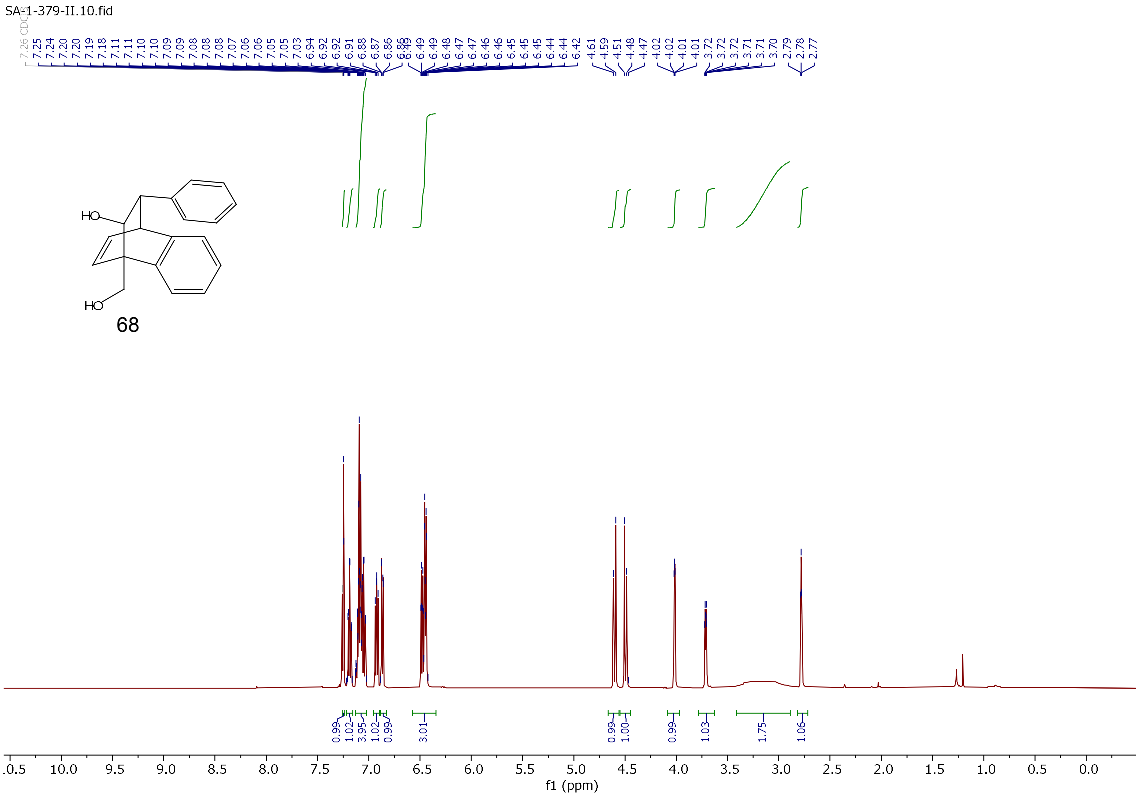
SA-1-379-II.10.fid
7.26 CDCl3
7.25 7.24 7.20 7.20 7.19 7.18 7.11 7.11 7.10 7.09 7.09 7.08 7.08 7.08 7.07 7.06 7.06 7.05 7.05 7.03 6.94 6.92 6.92 6.91 6.88 6.87 6.86 6.86 6.49 6.49 6.49 6.48 6.47 6.47 6.46 6.46 6.45 6.45 6.44 6.44 6.42 4.61 4.59 4.51 4.48 4.47 4.02 4.02 4.01 4.01 3.72 3.72 3.72 3.71 3.71 3.70 2.79 2.78 2.77
HO
HO
68
0.99 1.02 3.95 1.02 0.99 3.01 0.99 1.00 0.99 1.03 1.75 1.06
.0.5 10.0 9.5 9.0 8.5 8.0 7.5 7.0 6.5 6.0 5.5 5.0 4.5 4.0 3.5 3.0 2.5 2.0 1.5 1.0 0.5 0.0
f1 (ppm)

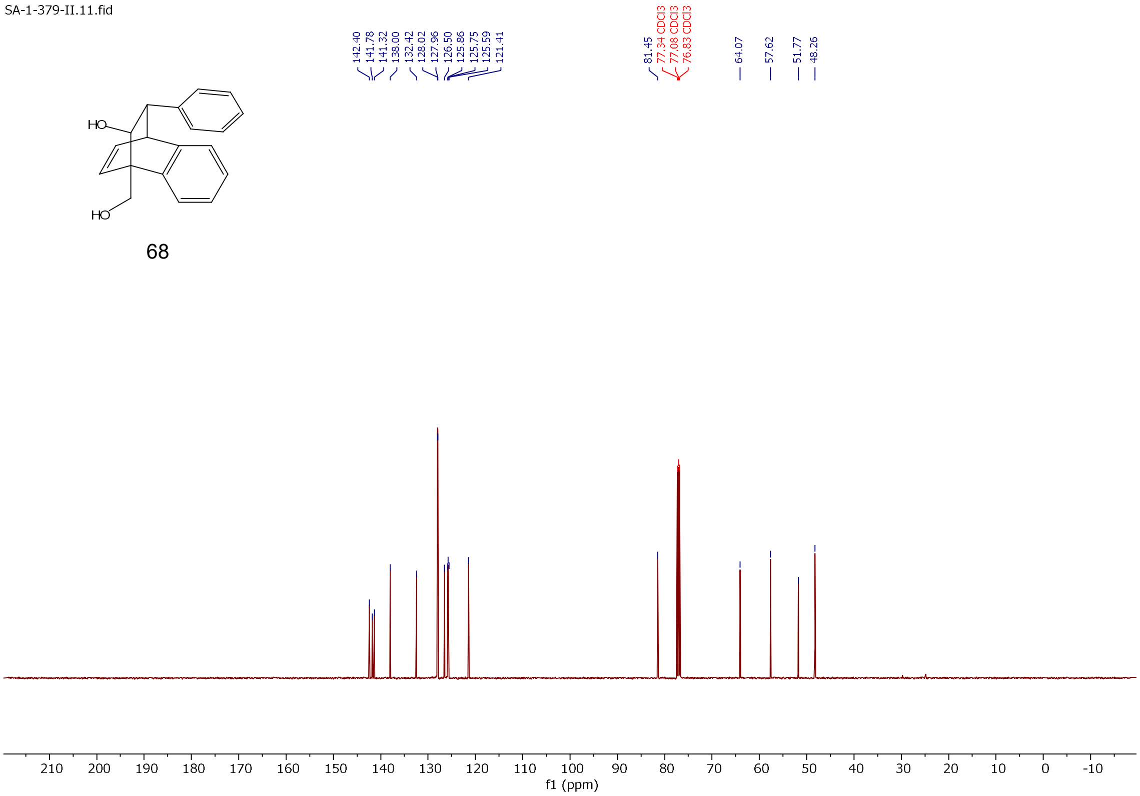
SA-1-379-II.11.fid
142.40 141.78 141.32 138.00 132.42 128.02 127.96 126.50 125.86 125.75 125.59 121.41
81.45
77.34 CDCl3
77.08 CDCl3
76.83 CDCl3
64.07 57.62 51.77 48.26
HO
HO
68
210 200 190 180 170 160 150 140 130 120 110 100 90 80 70 60 50 40 30 20 10 0 -10
f1 (ppm)

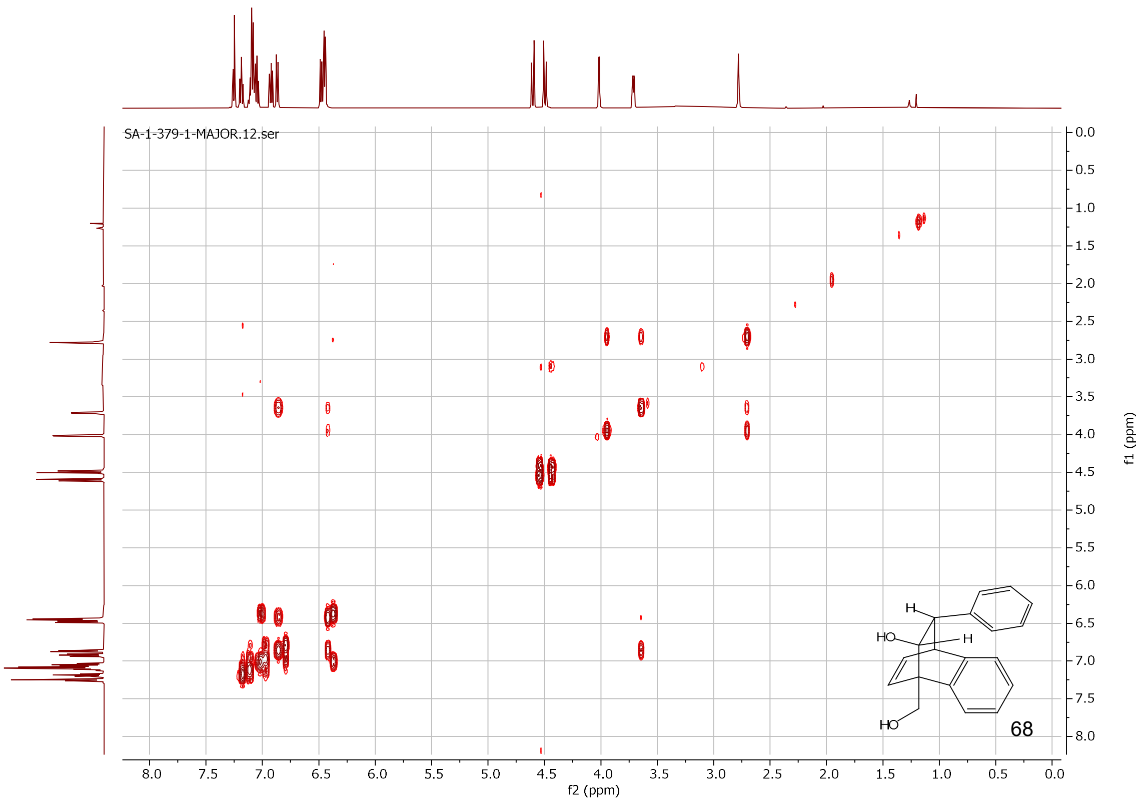
SA-1-379-1-MAJOR.12.ser
f2 (ppm)
f1 (ppm)
HO
H
H
HO
68

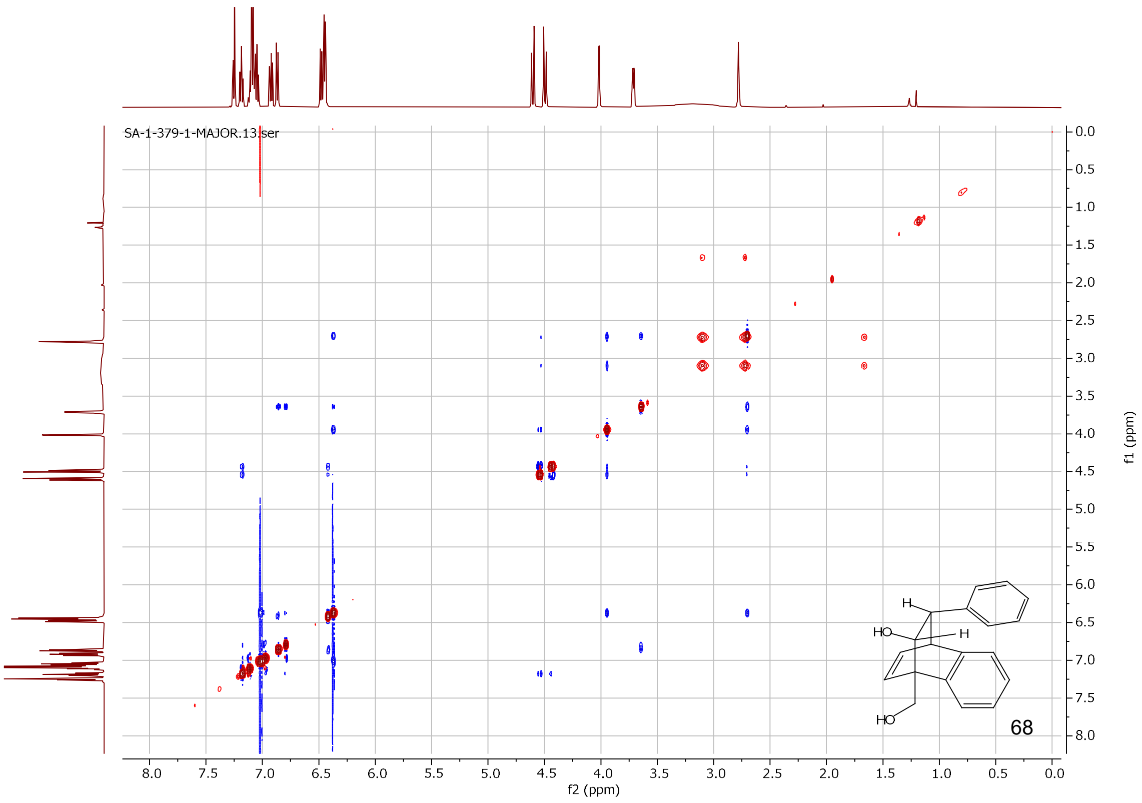
SA-1-379-1-MAJOR.13.ser
f2 (ppm)
f1 (ppm)
HO
H
H
HO
68

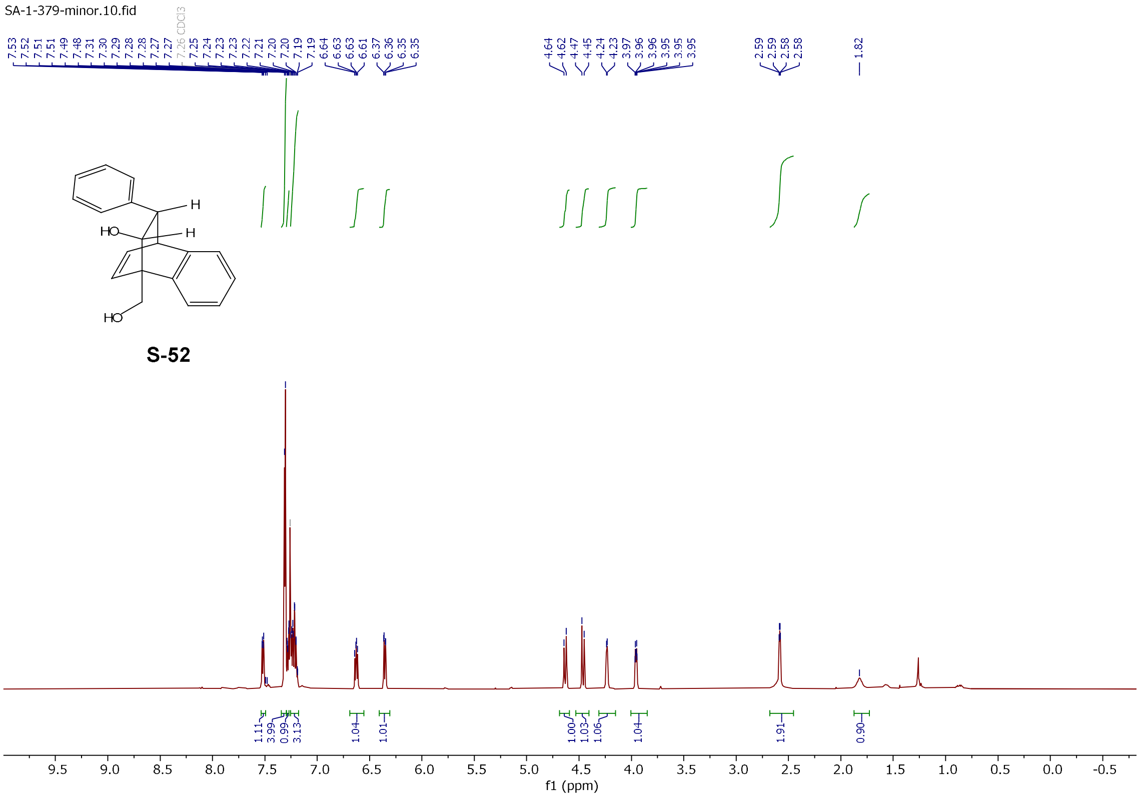
SA-1-379-minor.10.fid
7.53 7.52 7.51 7.51 7.49 7.48 7.31 7.30 7.29 7.28 7.28 7.27 7.27 7.26 CDCl3 7.25 7.24 7.23 7.23 7.22 7.22 7.21 7.20 7.20 7.19 7.19 6.64 6.63 6.63 6.61 6.37 6.36 6.35 6.35
4.64 4.62 4.47 4.45 4.24 4.23 3.97 3.96 3.96 3.95 3.95 3.95
2.59 2.59 2.58 2.58
1.82
H
HO
H
HO
S-52
1.11 3.99 0.99 3.13 1.04 1.01 1.00 1.03 1.06 1.04 1.91 0.90
9.5 9.0 8.5 8.0 7.5 7.0 6.5 6.0 5.5 5.0 4.5 4.0 3.5 3.0 2.5 2.0 1.5 1.0 0.5 0.0 -0.5
f1 (ppm)

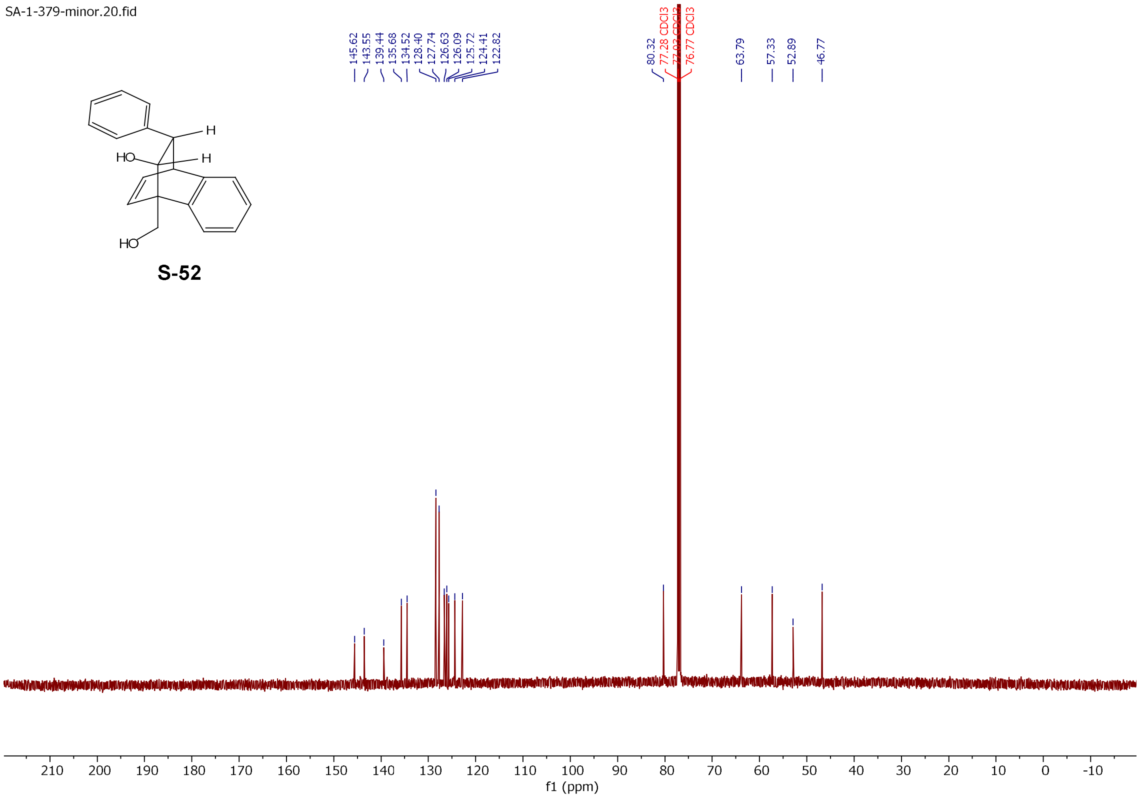
SA-1-379-minor.20.fid
145.62 143.55 139.44 135.68 134.52 128.40 127.74 126.63 126.09 125.72 124.41 122.82
80.32 77.28 CDCl3 77.02 CDCl3 76.77 CDCl3
63.79 57.33 52.89 46.77
H
HO
H
HO
S-52
210 200 190 180 170 160 150 140 130 120 110 100 90 80 70 60 50 40 30 20 10 0 -10
f1 (ppm)

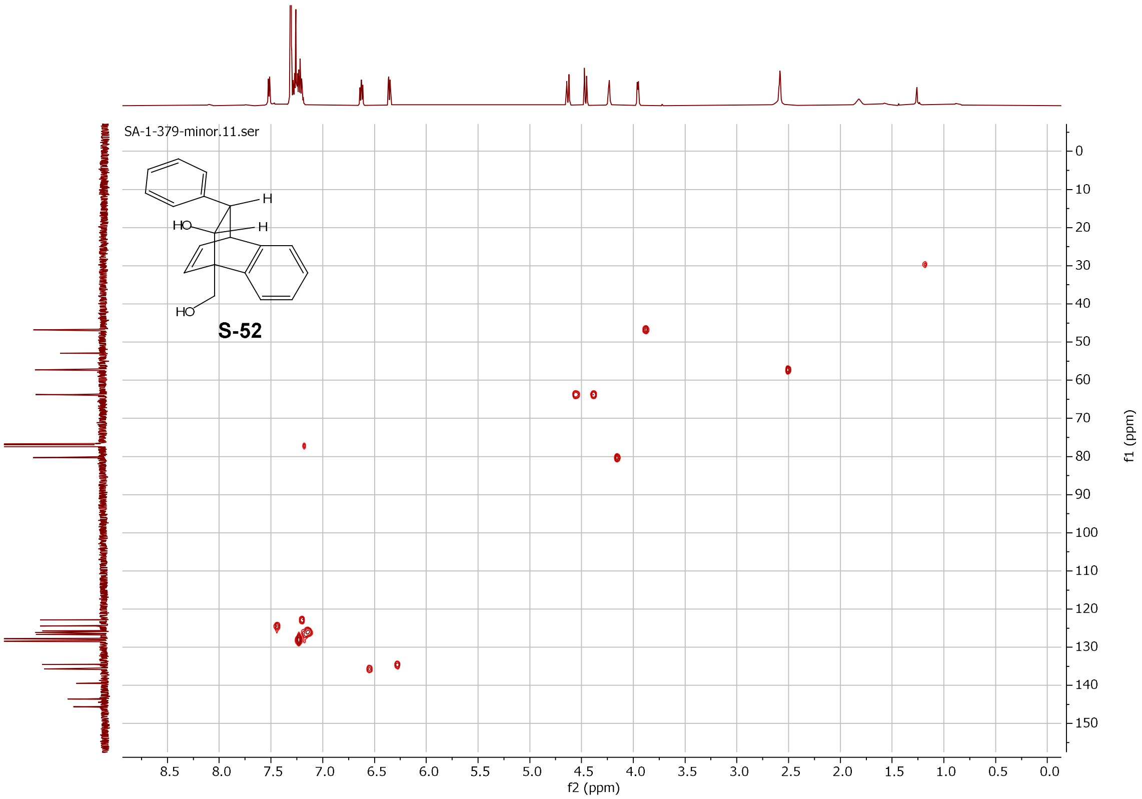
SA-1-379-minor.11.ser
S-52
f2 (ppm)
f1 (ppm)

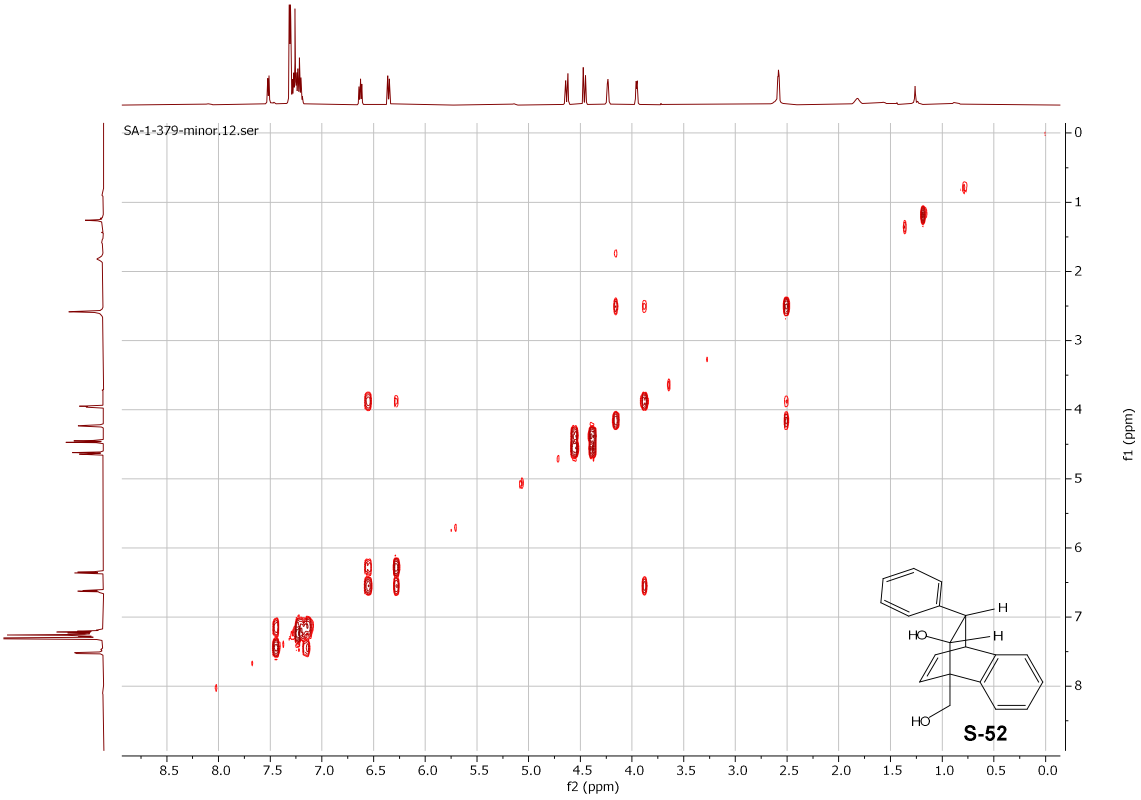
SA-1-379-minor.12.ser
S-52
f2 (ppm)
f1 (ppm)

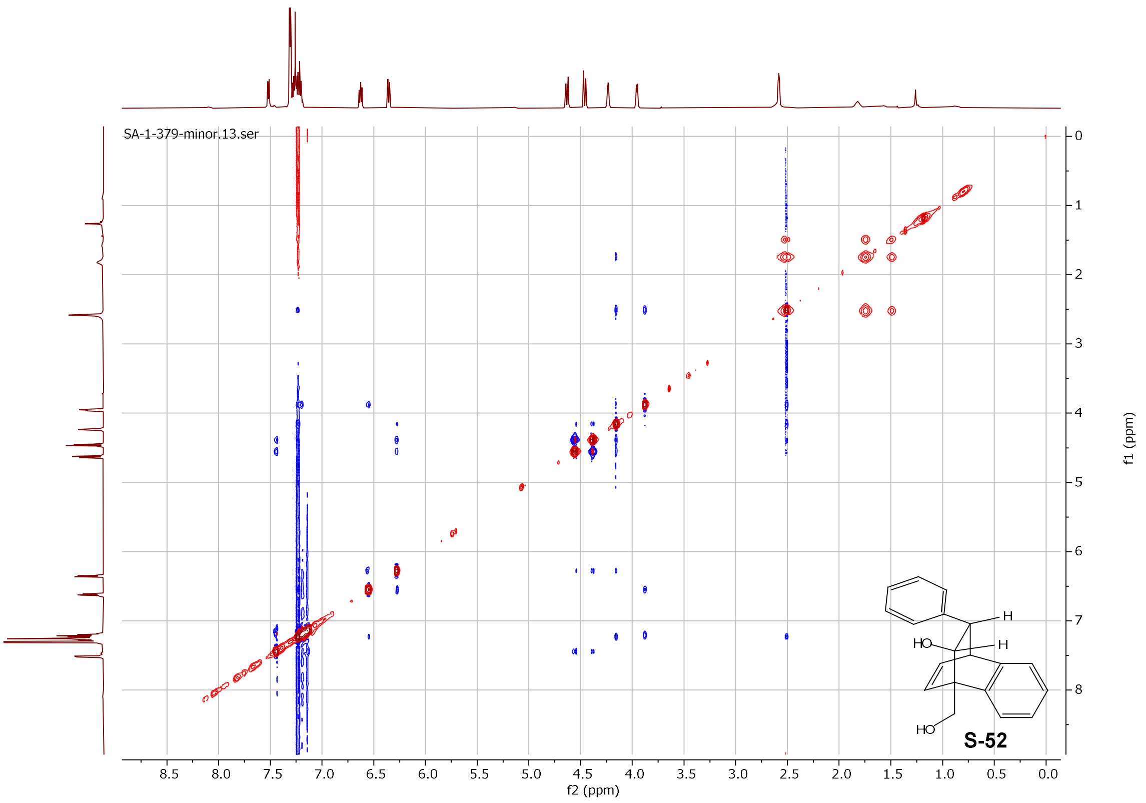
SA-1-379-minor.13.ser
f2 (ppm)
f1 (ppm)
HO
H
H
HO
S-52

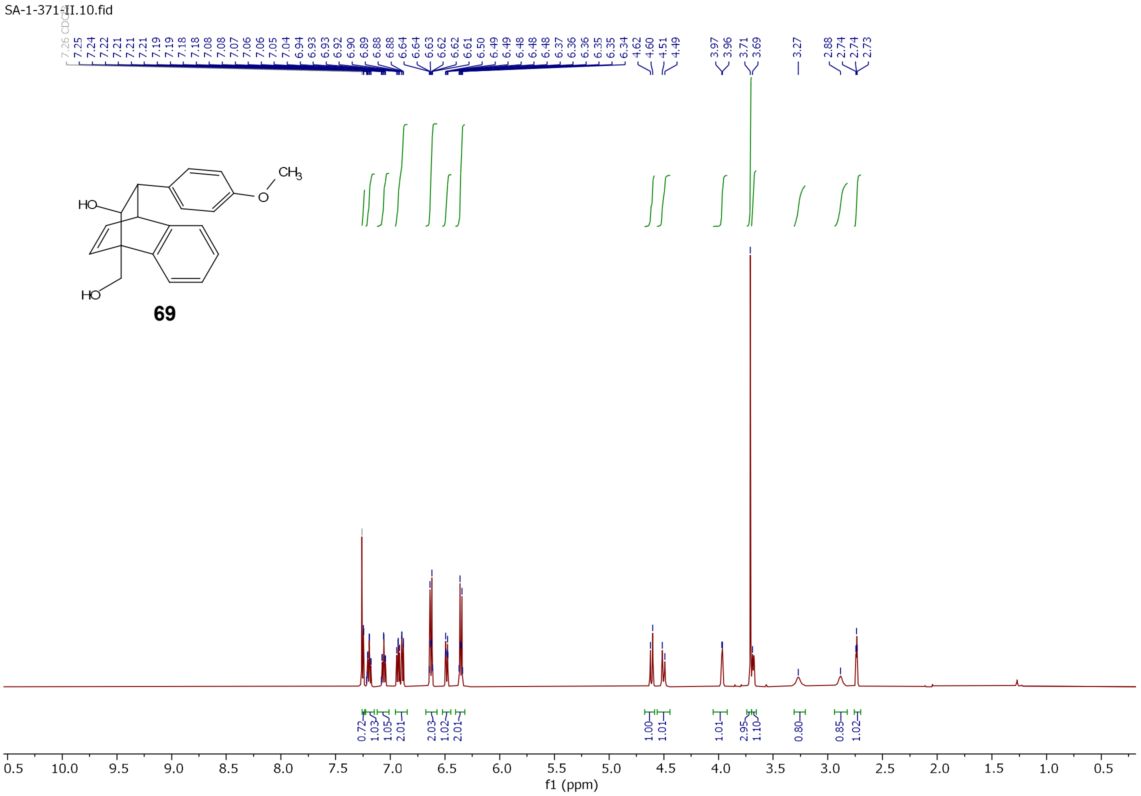
SA-1-371-II.10.fid
7.26 CDCl3
7.25 7.24 7.22 7.21 7.21 7.19 7.19 7.18 7.08 7.07 7.06 7.05 7.04 6.94 6.93 6.93 6.92 6.90 6.89 6.88 6.88 6.64 6.64 6.63 6.62 6.62 6.61 6.50 6.49 6.49 6.48 6.48 6.37 6.36 6.35 6.35 6.34 4.62 4.60 4.51 4.49 3.97 3.96 3.71 3.69 3.27 2.88 2.74 2.74 2.73
CH3
O
HO
HO
69
0.72 1.03 1.05 2.01 2.03 1.02 2.01 1.00 1.01 1.01 2.95 1.10 0.80 0.85 1.02
f1 (ppm)

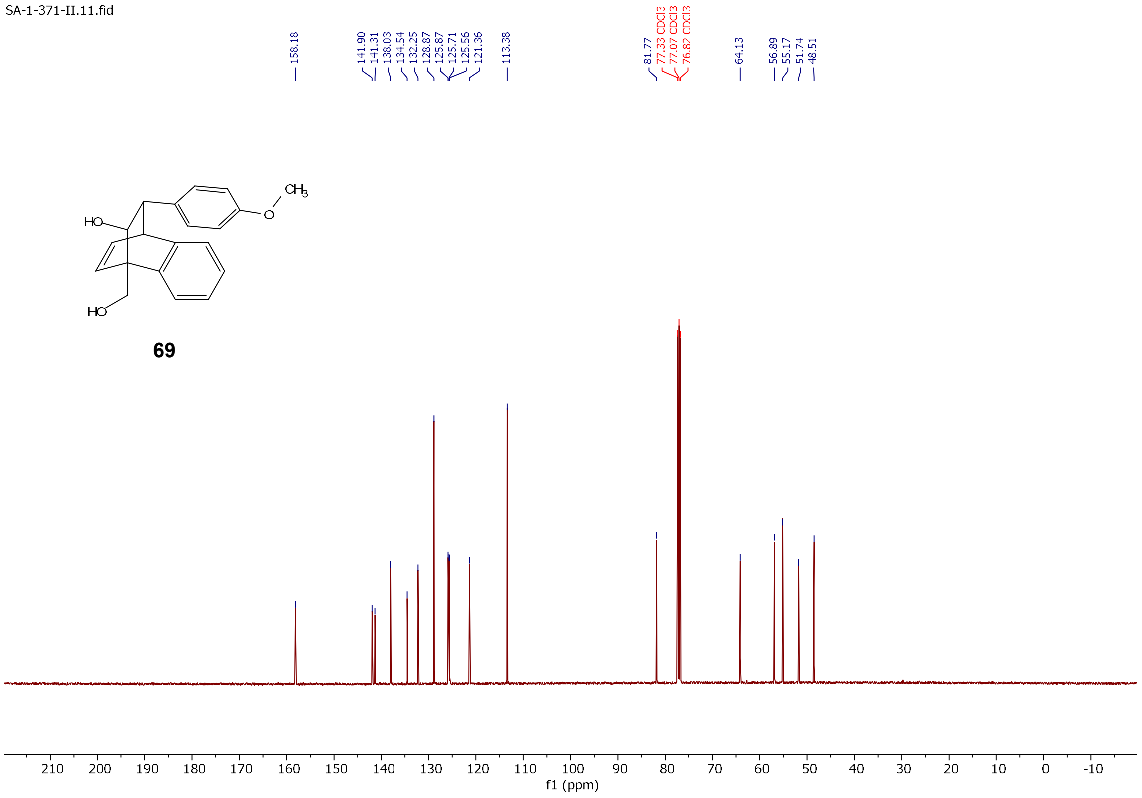
SA-1-371-II.11.fid
158.18
141.90
141.31
138.03
134.54
132.25
128.87
125.87
125.71
125.56
121.36
113.38
81.77
77.33 CDCl3
77.07 CDCl3
76.82 CDCl3
64.13
56.89
55.17
51.74
48.51
HO
CH3
O
HO
69
210 200 190 180 170 160 150 140 130 120 110 100 90 80 70 60 50 40 30 20 10 0 -10
f1 (ppm)

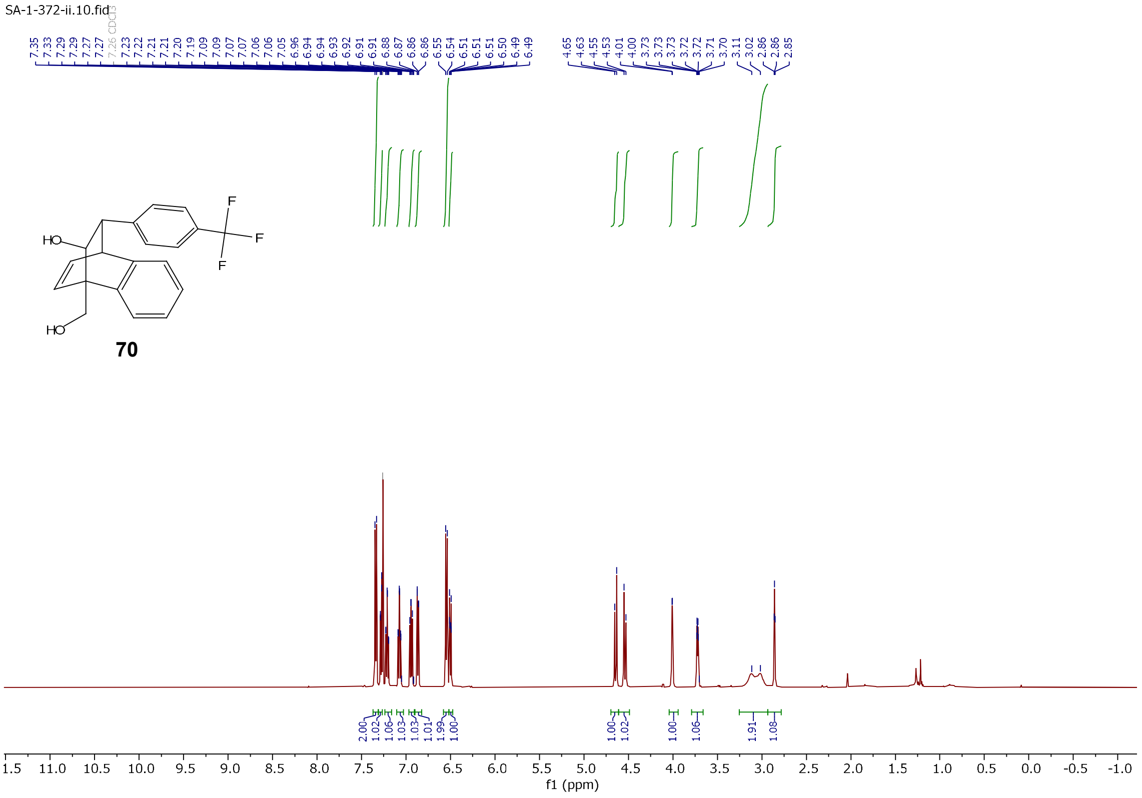
SA-1-372-ii.10.fid
7.35 7.33 7.29 7.29 7.27 7.27 7.26 CDCl3 7.23 7.22 7.21 7.21 7.20 7.19 7.09 7.09 7.07 7.07 7.06 7.06 7.05 6.96 6.94 6.94 6.93 6.92 6.91 6.91 6.88 6.87 6.86 6.86 6.55 6.54 6.51 6.51 6.51 6.50 6.49 6.49
4.65 4.63 4.55 4.53 4.01 4.00 3.73 3.73 3.72 3.72 3.71 3.70 3.11 3.02 2.86 2.85
HO
F
F
F
HO
70
2.00 1.02 1.06 1.03 1.03 1.01 1.99 1.00
1.00 1.02
1.00 1.06
1.91 1.08
11.5 11.0 10.5 10.0 9.5 9.0 8.5 8.0 7.5 7.0 6.5 6.0 5.5 5.0 4.5 4.0 3.5 3.0 2.5 2.0 1.5 1.0 0.5 0.0 -0.5 -1.0
f1 (ppm)

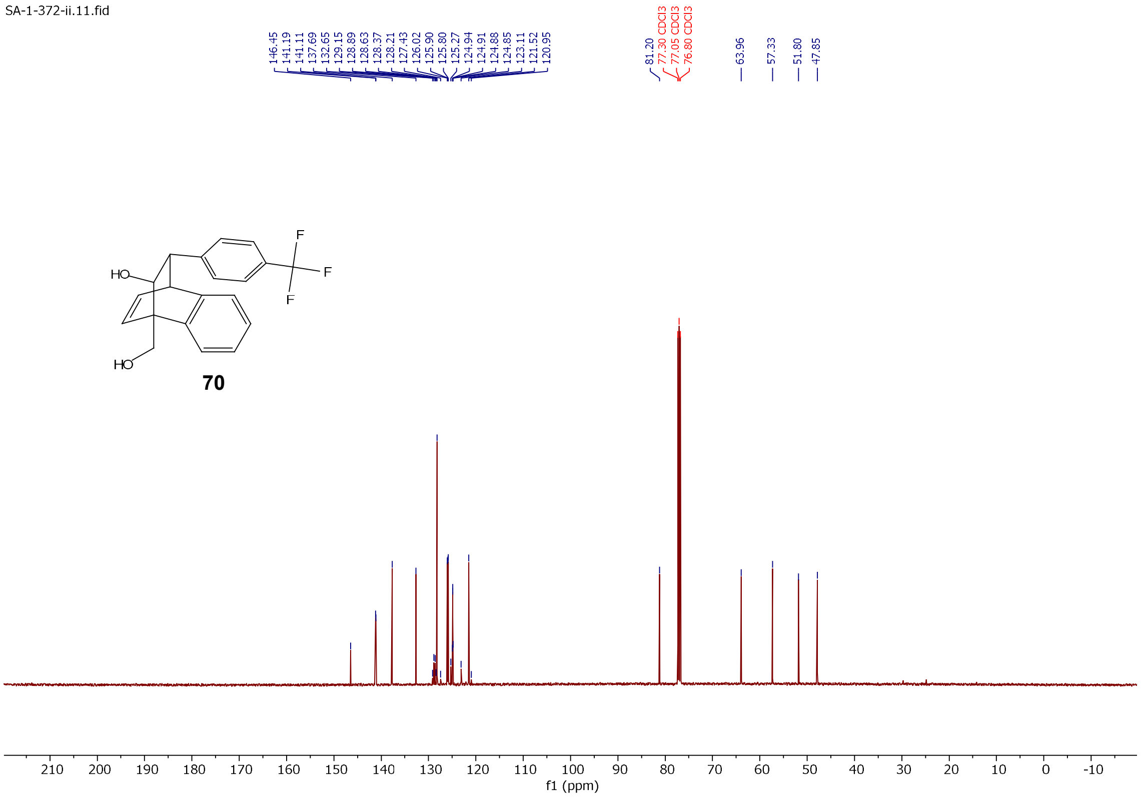
SA-1-372-ii.11.fid
146.45
141.19
141.11
137.69
132.65
129.15
128.89
128.63
128.37
128.21
127.43
126.02
125.90
125.80
125.27
124.94
124.91
124.88
124.85
123.11
121.52
120.95
81.20
77.30 CDCl3
77.05 CDCl3
76.80 CDCl3
63.96
57.33
51.80
47.85
HO
HO
F
F
F
70
210 200 190 180 170 160 150 140 130 120 110 100 90 80 70 60 50 40 30 20 10 0 -10
f1 (ppm)

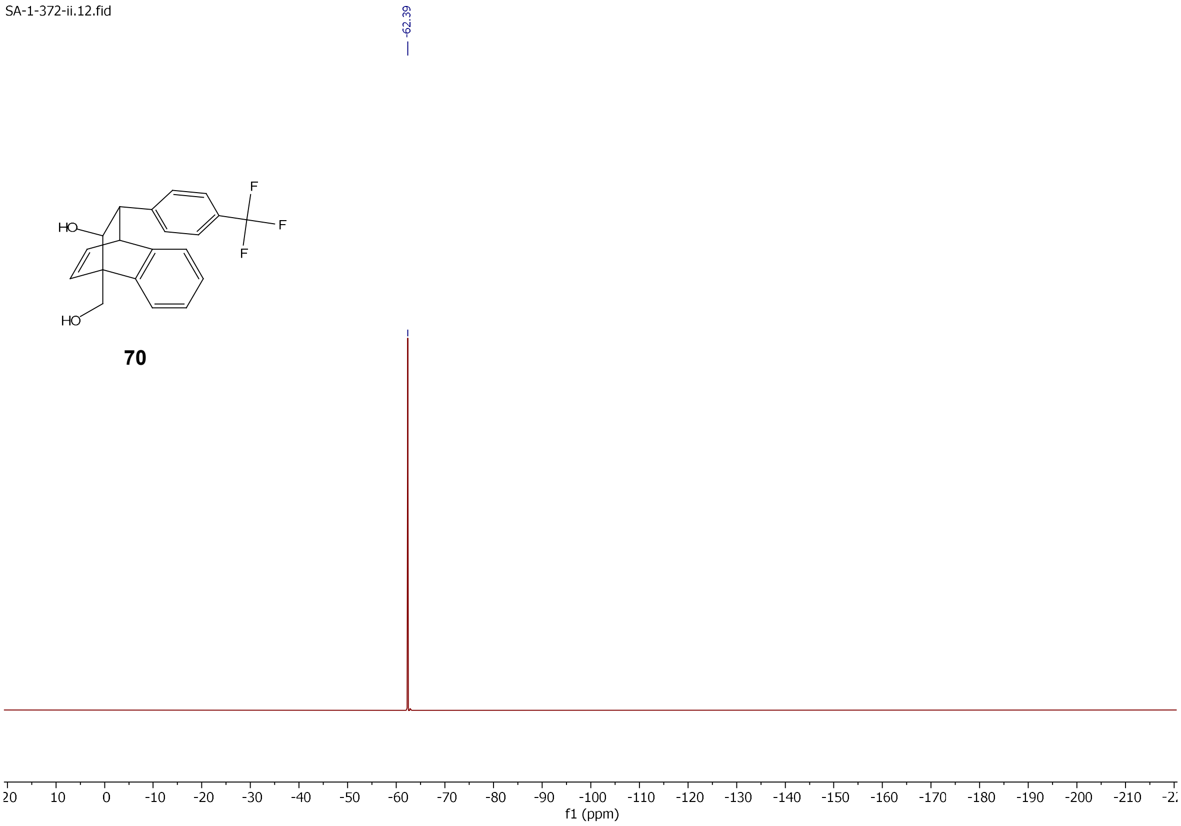
SA-1-372-ii.12.fid
-62.39
HO
HO
F
F
F
70
20 10 0 -10 -20 -30 -40 -50 -60 -70 -80 -90 -100 -110 -120 -130 -140 -150 -160 -170 -180 -190 -200 -210
f1 (ppm)

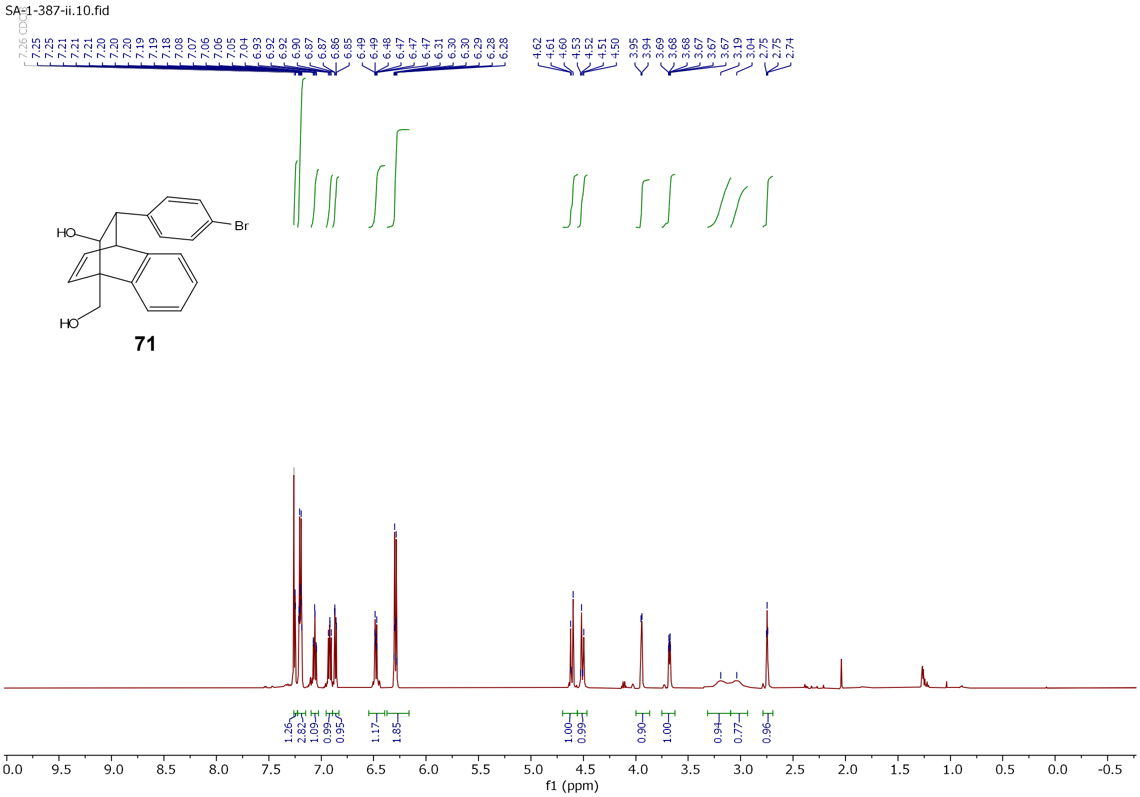
SA-1-387-ii.10.fid
7.26 CDCl3
7.25 7.25 7.21 7.21 7.21 7.20 7.20 7.20 7.19 7.19 7.18 7.08 7.07 7.06 7.06 7.05 7.04 6.93 6.92 6.92 6.92 6.90 6.87 6.87 6.86 6.85 6.49 6.49 6.48 6.47 6.47 6.47 6.31 6.30 6.30 6.29 6.28 6.28
4.62 4.61 4.60 4.53 4.52 4.51 4.50 3.95 3.94 3.69 3.68 3.68 3.67 3.67 3.67 3.19 3.04 2.75 2.75 2.74
HO
Br
HO
71
1.26 2.82 1.09 0.99 0.95 1.17 1.85 1.00 0.99 0.90 1.00 0.94 0.77 0.96
0.0 9.5 9.0 8.5 8.0 7.5 7.0 6.5 6.0 5.5 5.0 4.5 4.0 3.5 3.0 2.5 2.0 1.5 1.0 0.5 0.0 -0.5
f1 (ppm)

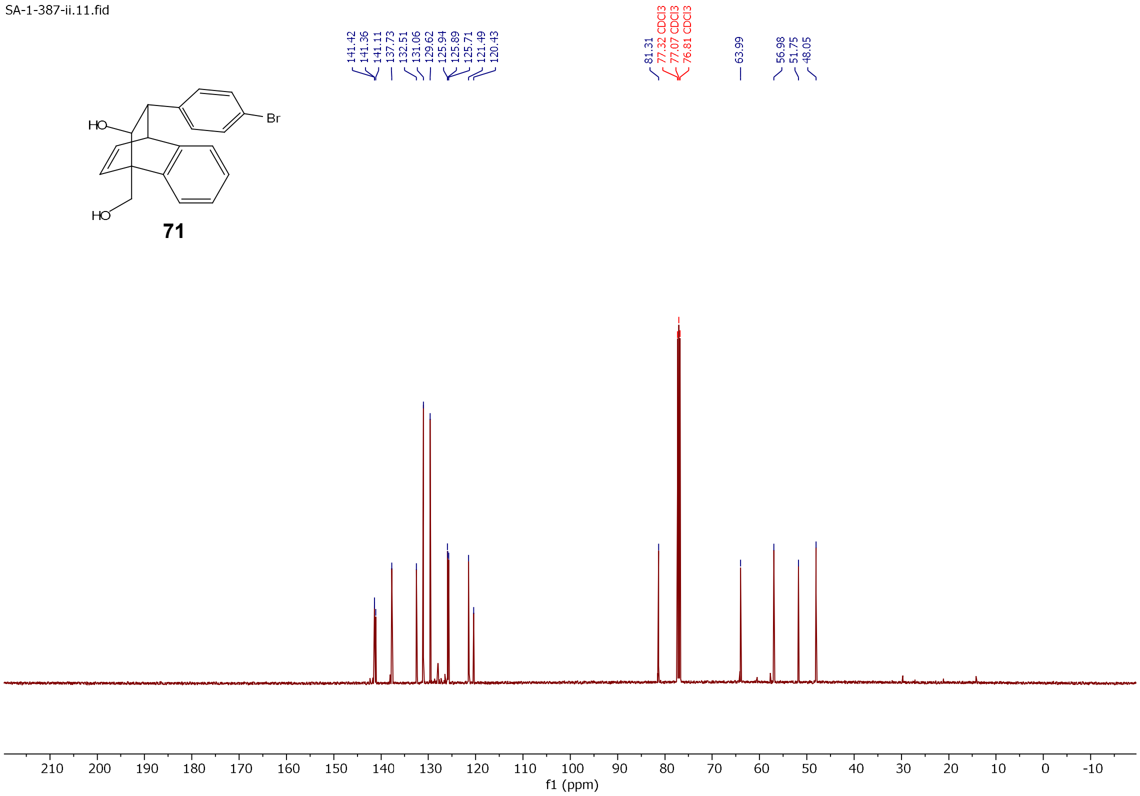
SA-1-387-ii.11.fid
141.42 141.36 141.11 137.73 132.51 131.06 129.62 125.94 125.89 125.71 121.49 120.43
81.31 77.32 CDCl3 77.07 CDCl3 76.81 CDCl3
63.99
56.98 51.75 48.05
HO
Br
HO
71
210 200 190 180 170 160 150 140 130 120 110 100 90 80 70 60 50 40 30 20 10 0 -10
f1 (ppm)

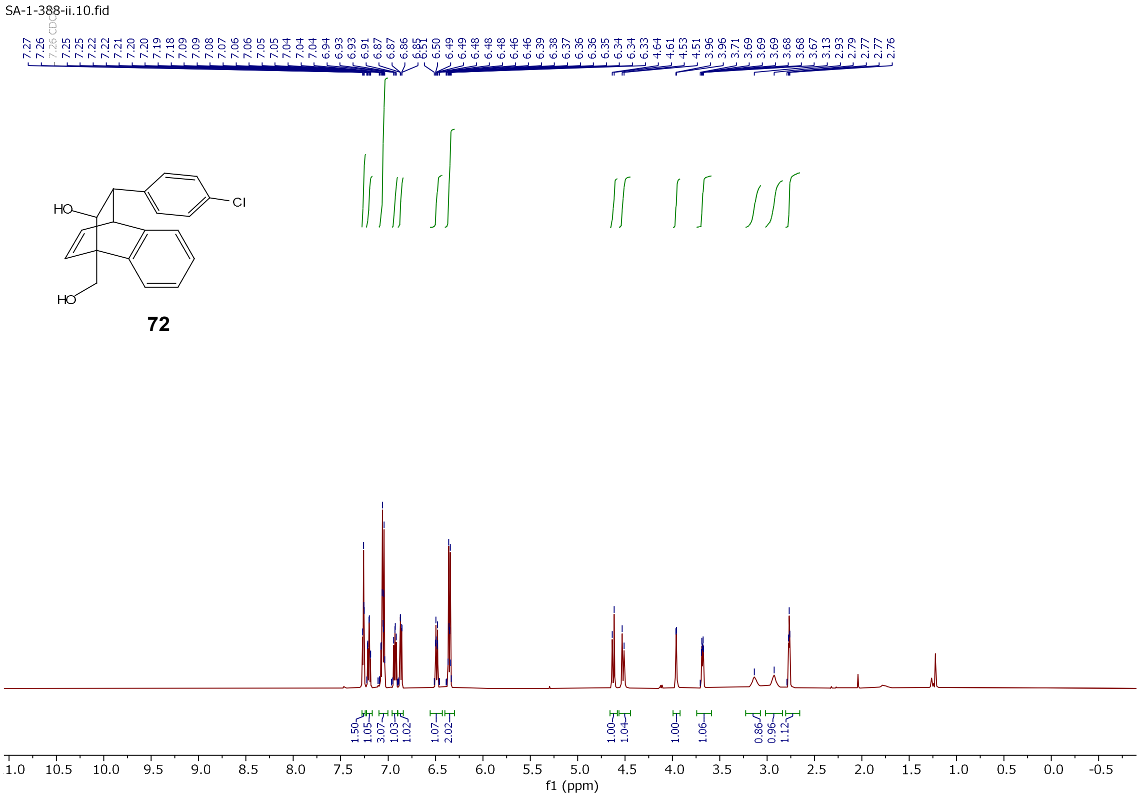
SA-1-388-ii.10.fid
7.27 7.26 7.26 CDCl3 7.25 7.25 7.22 7.22 7.21 7.20 7.20 7.19 7.18 7.09 7.09 7.08 7.07 7.06 7.06 7.05 7.05 7.04 7.04 7.04 7.04 6.94 6.93 6.93 6.91 6.87 6.87 6.86 6.86 6.51 6.50 6.49 6.49 6.48 6.48 6.48 6.46 6.39 6.38 6.37 6.36 6.36 6.35 6.34 6.34 6.33 4.64 4.61 4.53 4.51 3.96 3.96 3.71 3.69 3.69 3.69 3.68 3.67 3.13 2.93 2.79 2.77 2.77 2.76
HO
Cl
HO
72
1.50 1.05 3.07 1.03 1.02 1.07 2.02 1.00 1.04 1.00 1.06 0.86 0.96 1.12
1.0 10.5 10.0 9.5 9.0 8.5 8.0 7.5 7.0 6.5 6.0 5.5 5.0 4.5 4.0 3.5 3.0 2.5 2.0 1.5 1.0 0.5 0.0 -0.5
f1 (ppm)

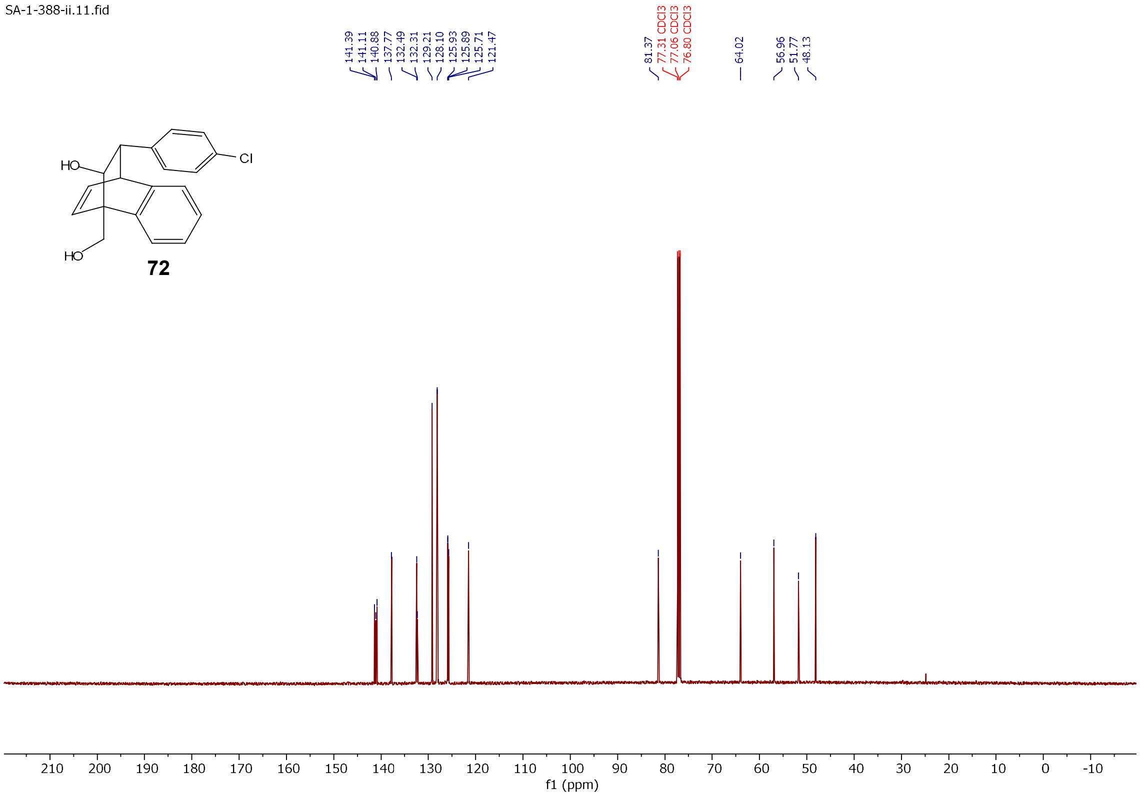
SA-1-388-ii.11.fid
141.39 141.11 140.88 137.77 132.49 132.31 129.21 128.10 125.93 125.89 125.71 121.47
81.37 77.31 CDCl3 77.06 CDCl3 76.80 CDCl3
64.02
56.96 51.77 48.13
HO
Cl
HO
72
210 200 190 180 170 160 150 140 130 120 110 100 90 80 70 60 50 40 30 20 10 0 -10
f1 (ppm)

SA-1-392-1.10.fid

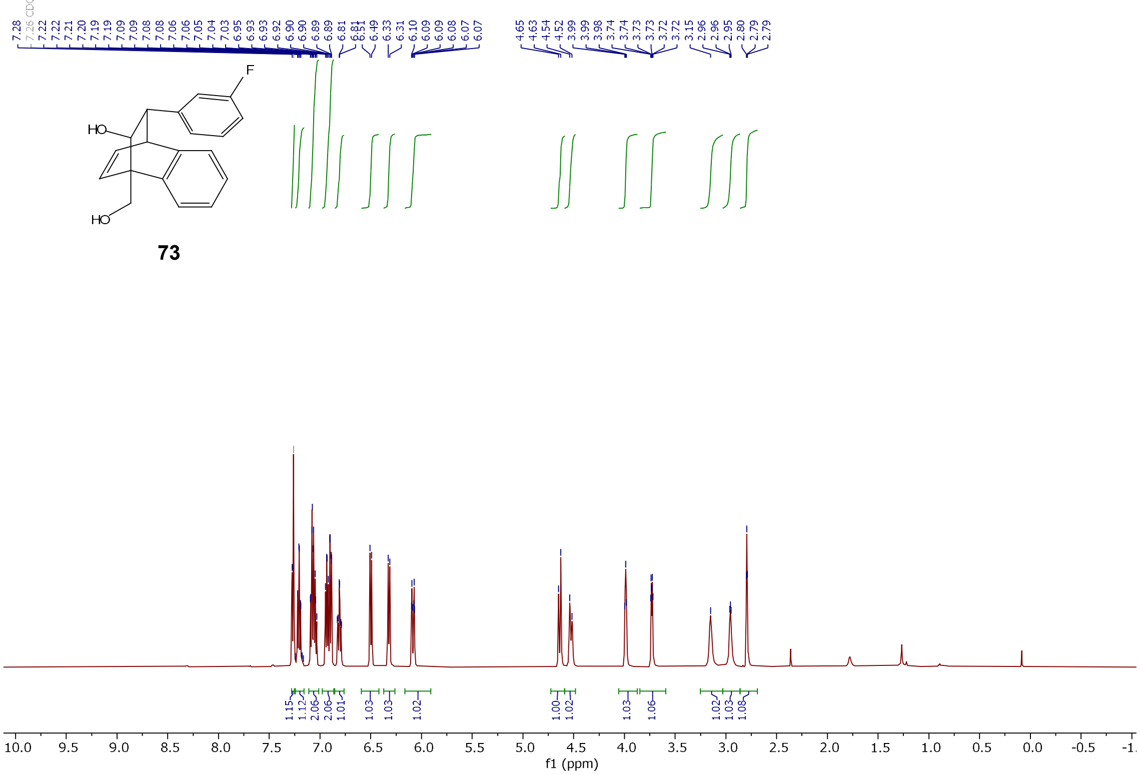

73

SA-1-392-1.11.fid

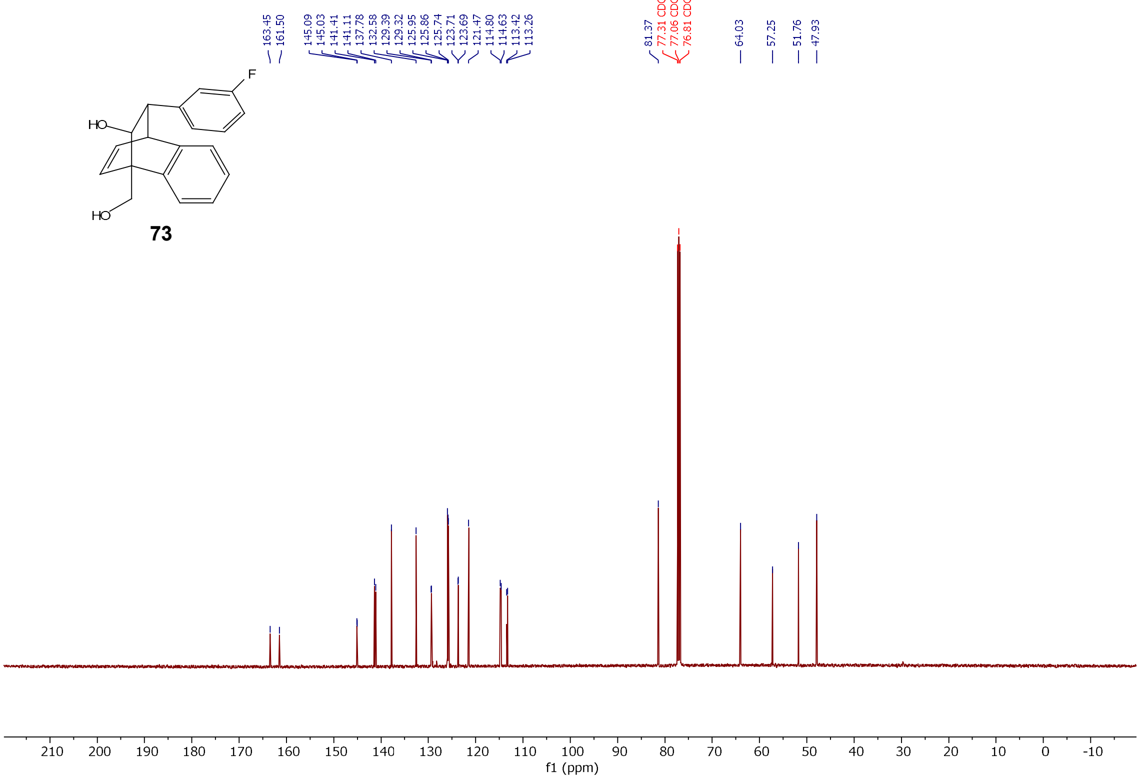

73

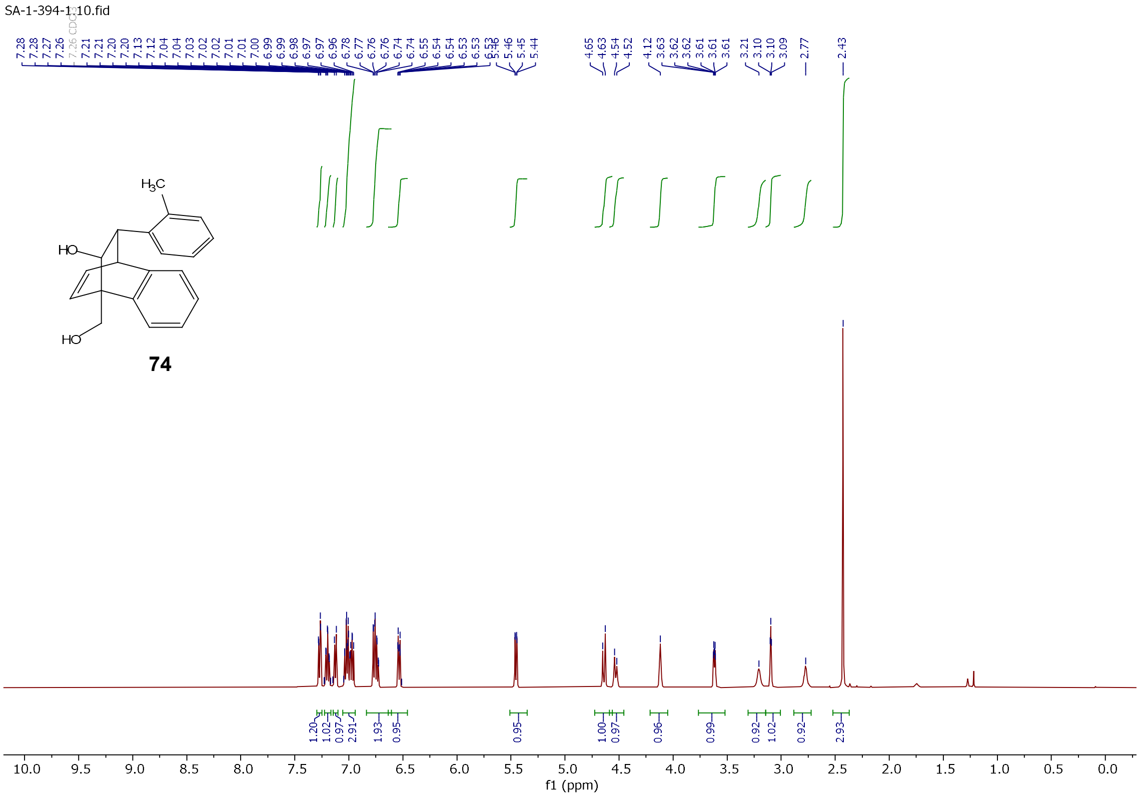
SA-1-394-1.10.fid
7.28 7.28 7.27 7.26 CDCl3 7.21 7.21 7.20 7.20 7.13 7.12 7.04 7.04 7.03 7.02 7.02 7.01 7.01 7.00 6.99 6.99 6.98 6.97 6.97 6.96 6.78 6.77 6.76 6.76 6.74 6.74 6.55 6.54 6.54 6.53 6.53 5.46 5.46 5.45 5.44 4.65 4.63 4.54 4.52 4.12 3.63 3.62 3.62 3.61 3.61 3.61 3.21 3.10 3.10 3.09 2.77 2.43
H3C
HO
HO
74
1.20 1.02 0.97 2.91 1.93 0.95 0.95 1.00 0.97 0.96 0.99 0.92 1.02 0.92 2.93
10.0 9.5 9.0 8.5 8.0 7.5 7.0 6.5 6.0 5.5 5.0 4.5 4.0 3.5 3.0 2.5 2.0 1.5 1.0 0.5 0.0
f1 (ppm)

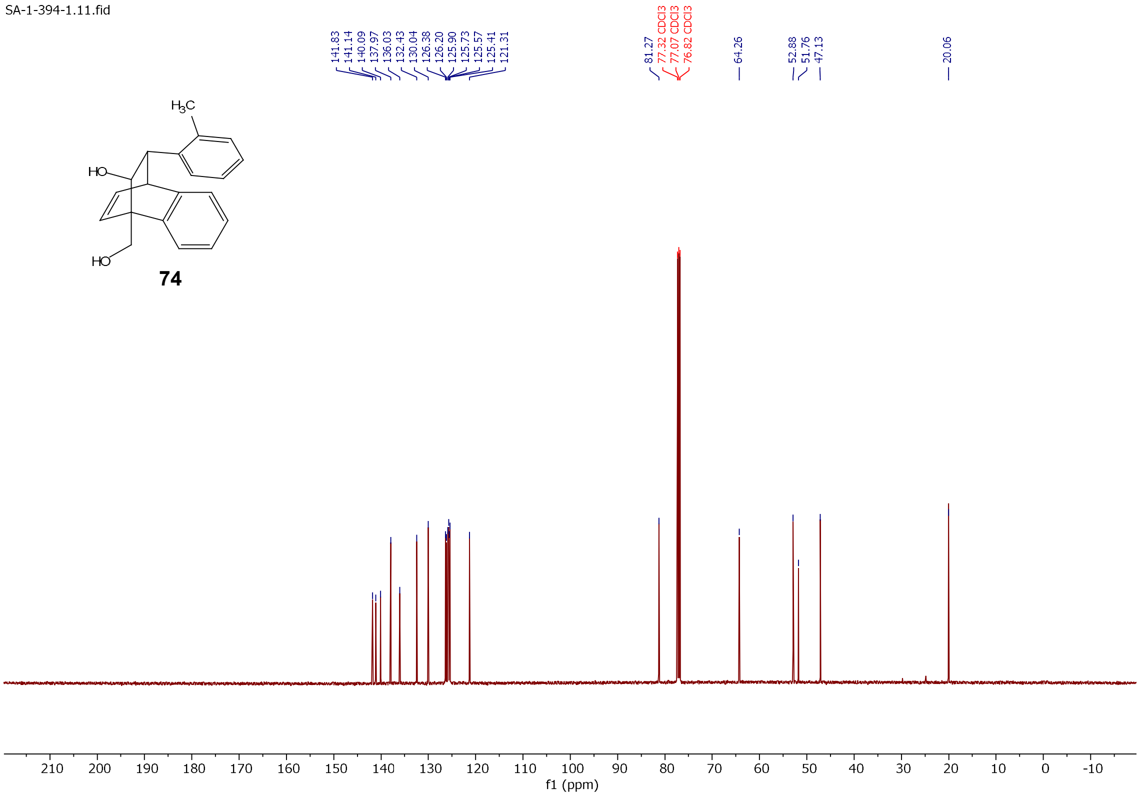
SA-1-394-1.11.fid
141.83 141.14 140.09 137.97 136.03 132.43 130.04 126.38 126.20 125.90 125.73 125.57 125.41 121.31
81.27 77.32 CDCl3 77.07 CDCl3 76.82 CDCl3
64.26 52.88 51.76 47.13 20.06
H3C
HO
HO
74
210 200 190 180 170 160 150 140 130 120 110 100 90 80 70 60 50 40 30 20 10 0 -10
f1 (ppm)

SA-1-397-1.40.fid

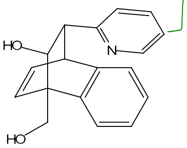

75

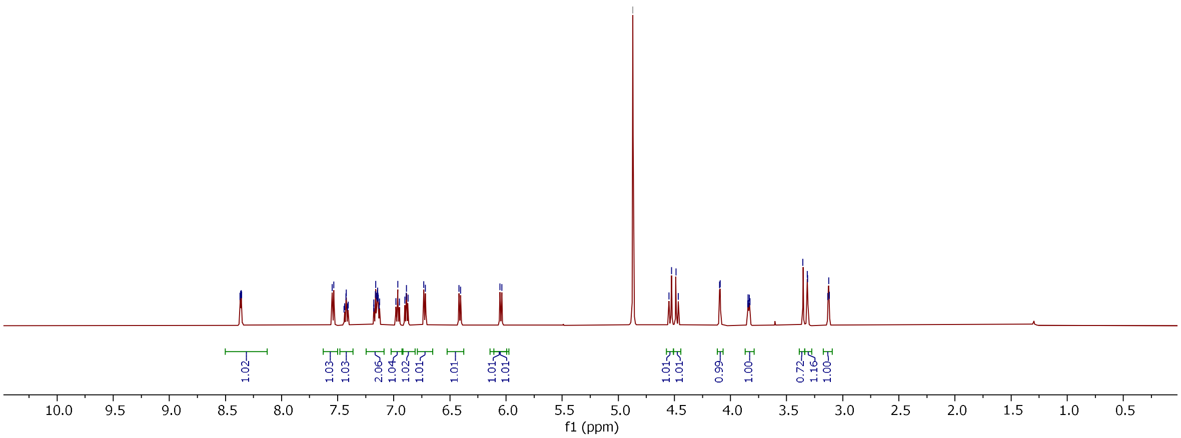

SA-1-397-1.41.fid

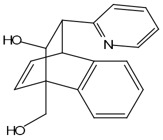

75

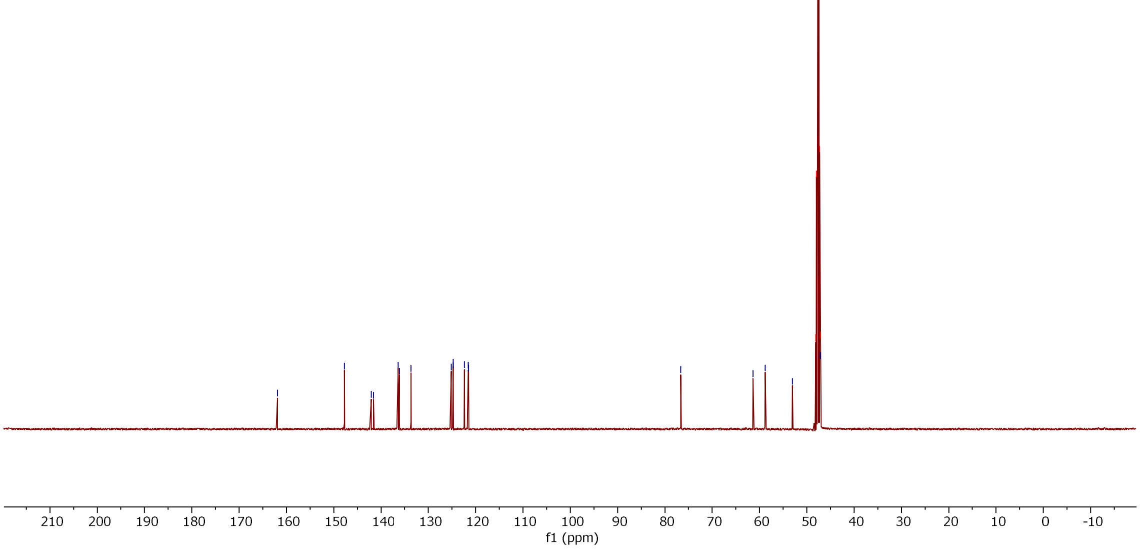

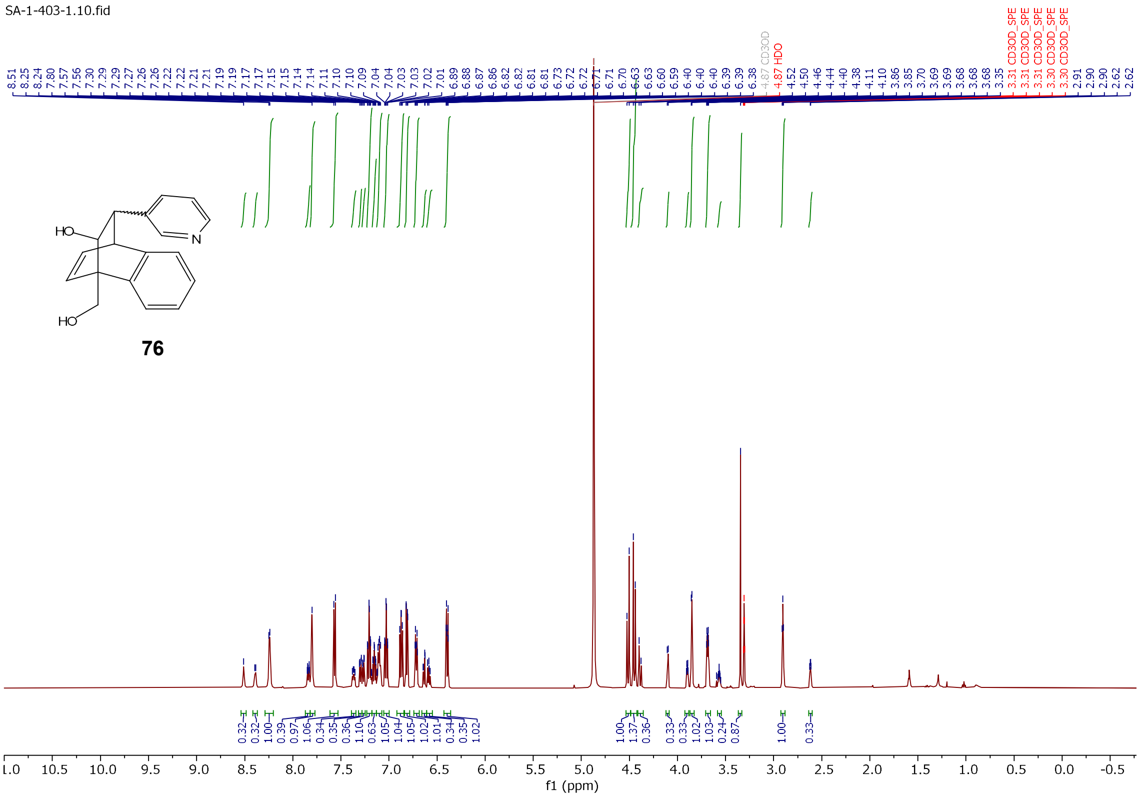

SA-1-403-1.10.fid
8.51 8.25 8.24 7.80 7.57 7.56 7.30 7.29 7.29 7.27 7.26 7.26 7.22 7.22 7.21 7.21 7.19 7.19 7.17 7.17 7.15 7.15 7.14 7.14 7.11 7.10 7.10 7.09 7.04 7.04 7.03 7.03 7.02 7.01 6.89 6.88 6.87 6.86 6.82 6.82 6.81 6.81 6.73 6.72 6.72 6.71 6.71 6.70 6.63 6.63 6.60 6.59 6.40 6.40 6.40 6.39 6.39 6.38
4.87 CD3OD
4.87 HDO
4.52 4.50 4.46 4.44 4.40 4.38 4.11 4.10 3.86 3.85 3.70 3.69 3.69 3.68 3.68 3.68 3.35
3.31 CD3OD_SPE
3.31 CD3OD_SPE
3.31 CD3OD_SPE
3.30 CD3OD_SPE
3.30 CD3OD_SPE
2.91 2.90 2.90 2.62 2.62
HO
N
HO
76
0.32 0.32 1.00 0.39 0.97 1.06 0.34 0.35 0.36 1.10 0.63 1.05 1.04 1.05 1.02 1.01 0.34 0.35 1.02
1.00 1.37 0.36 0.33 0.33 1.02 1.03 0.24 0.87 1.00 0.33
1.0 10.5 10.0 9.5 9.0 8.5 8.0 7.5 7.0 6.5 6.0 5.5 5.0 4.5 4.0 3.5 3.0 2.5 2.0 1.5 1.0 0.5 0.0 -0.5
f1 (ppm)

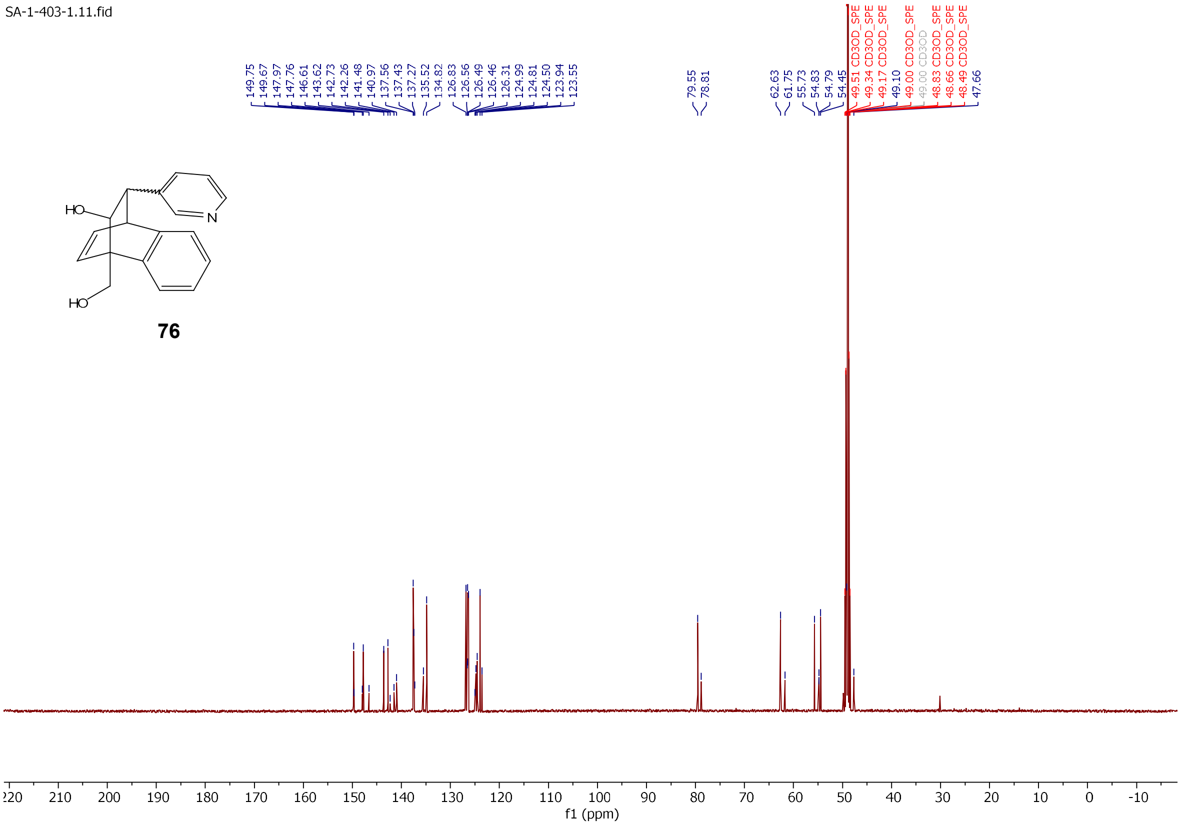

SA-1-403-1.11.fid
149.75 149.67 147.97 147.76 146.61 143.62 142.73 142.26 141.48 140.97 137.56 137.43 137.27 135.52 134.82 126.83 126.56 126.49 126.46 126.31 124.99 124.81 124.50 123.94 123.55
79.55 78.81
62.63 61.75 55.73 54.83 54.79 54.45
49.51 CD3OD_SPE
49.34 CD3OD_SPE
49.17 CD3OD_SPE
49.10
49.00 CD3OD_SPE
49.00 CD3OD
48.83 CD3OD_SPE
48.66 CD3OD_SPE
48.49 CD3OD_SPE
47.66
HO
N
HO
76
220 210 200 190 180 170 160 150 140 130 120 110 100 90 80 70 60 50 40 30 20 10 0 -10
f1 (ppm)

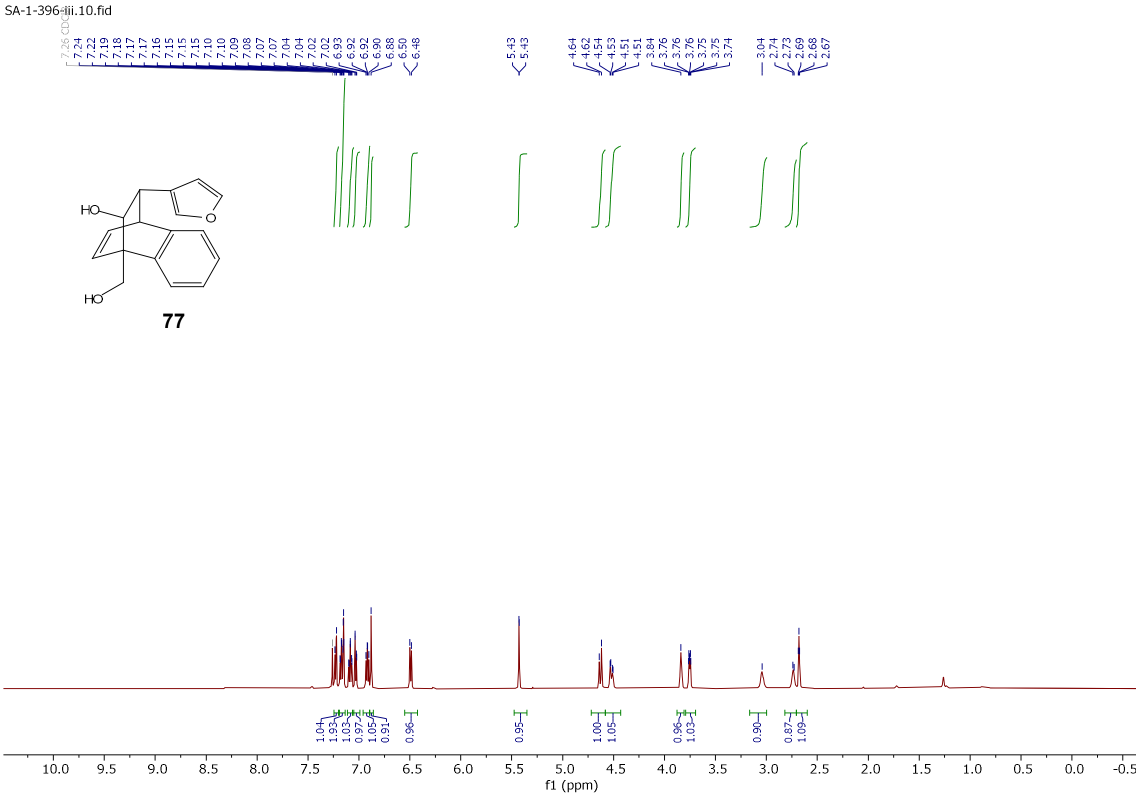
SA-1-396-iii.10.fid
7.26 CDCl3
7.24 7.22 7.19 7.18 7.17 7.17 7.16 7.15 7.15 7.15 7.10 7.10 7.09 7.08 7.07 7.07 7.04 7.04 7.02 7.02 6.93 6.93 6.92 6.92 6.90 6.88 6.50 6.48
5.43 5.43
4.64 4.62 4.54 4.53 4.51 4.51 3.84 3.76 3.76 3.75 3.75 3.74
3.04 2.74 2.73 2.69 2.68 2.67
HO
O
HO
77
1.04 1.93 1.03 0.97 1.05 0.91 0.96 0.95 1.00 1.05 0.96 1.03 0.90 0.87 1.09
10.0 9.5 9.0 8.5 8.0 7.5 7.0 6.5 6.0 5.5 5.0 4.5 4.0 3.5 3.0 2.5 2.0 1.5 1.0 0.5 0.0 -0.5
f1 (ppm)

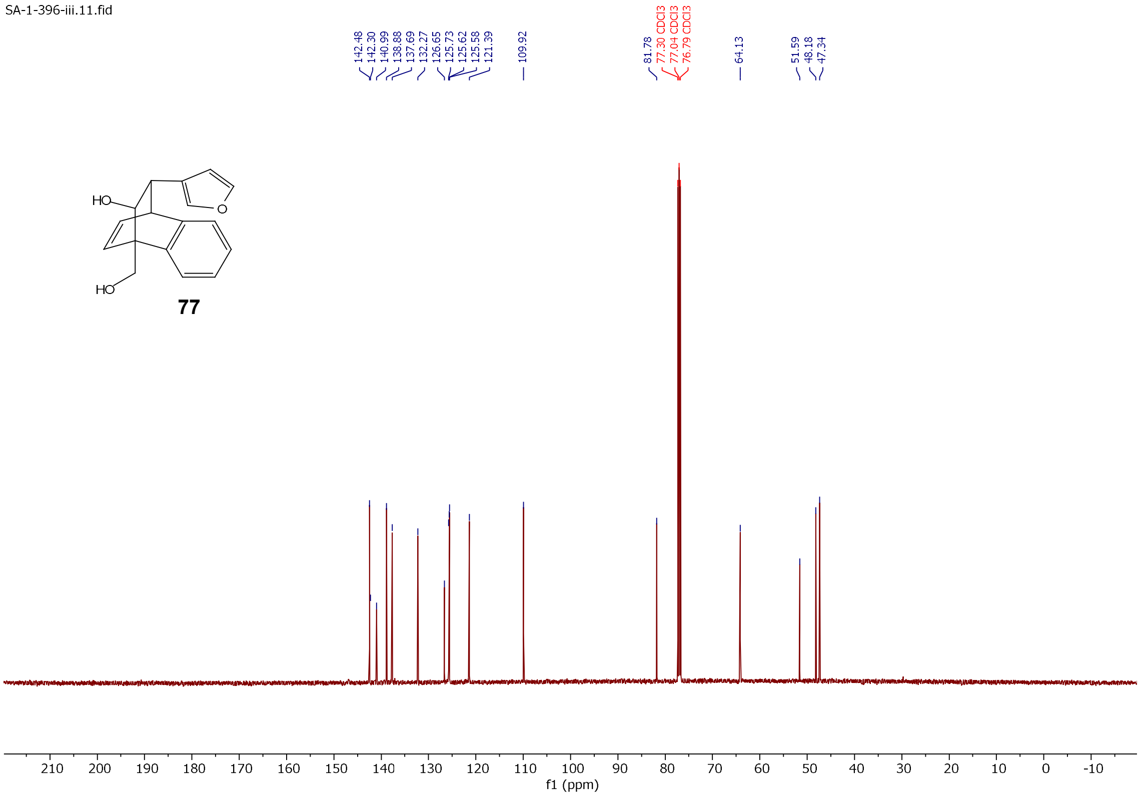
SA-1-396-iii.11.fid
142.48 142.30 140.99 138.88 137.69 132.27 126.65 125.73 125.62 125.58 121.39
109.92
81.78
77.30 CDCl3 77.04 CDCl3 76.79 CDCl3
64.13
51.59 48.18 47.34
HO
O
HO
77
210 200 190 180 170 160 150 140 130 120 110 100 90 80 70 60 50 40 30 20 10 0 -10
f1 (ppm)

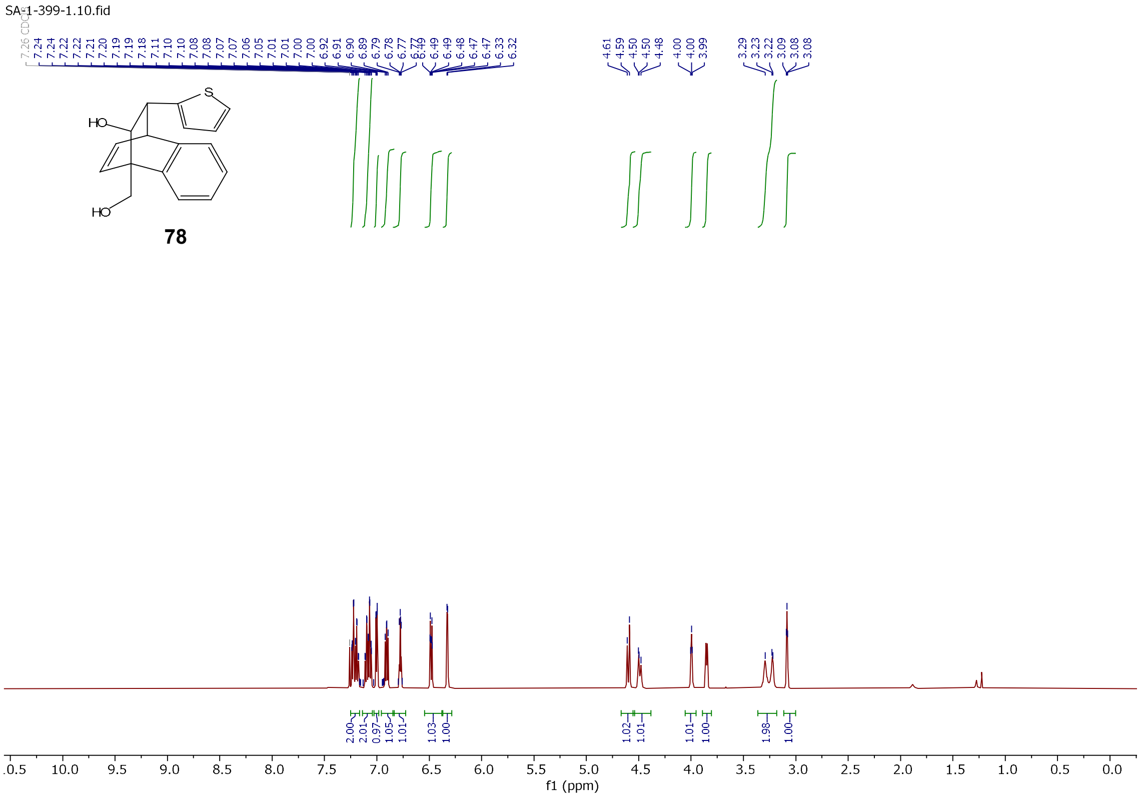
SA-1-399-1.10.fid
7.26 CDCl3
7.24 7.24 7.22 7.22 7.21 7.20 7.19 7.19 7.18 7.11 7.10 7.08 7.08 7.07 7.07 7.06 7.05 7.01 7.01 7.00 7.00 6.92 6.91 6.90 6.89 6.79 6.78 6.77 6.77 6.49 6.49 6.48 6.47 6.47 6.33 6.32
4.61 4.59 4.50 4.50 4.50 4.48 4.00 4.00 3.99 3.29 3.23 3.22 3.09 3.08 3.08
HO
S
HO
78
2.00 2.01 0.97 1.05 1.01 1.03 1.00 1.02 1.01 1.01 1.00 1.98 1.00
f1 (ppm)

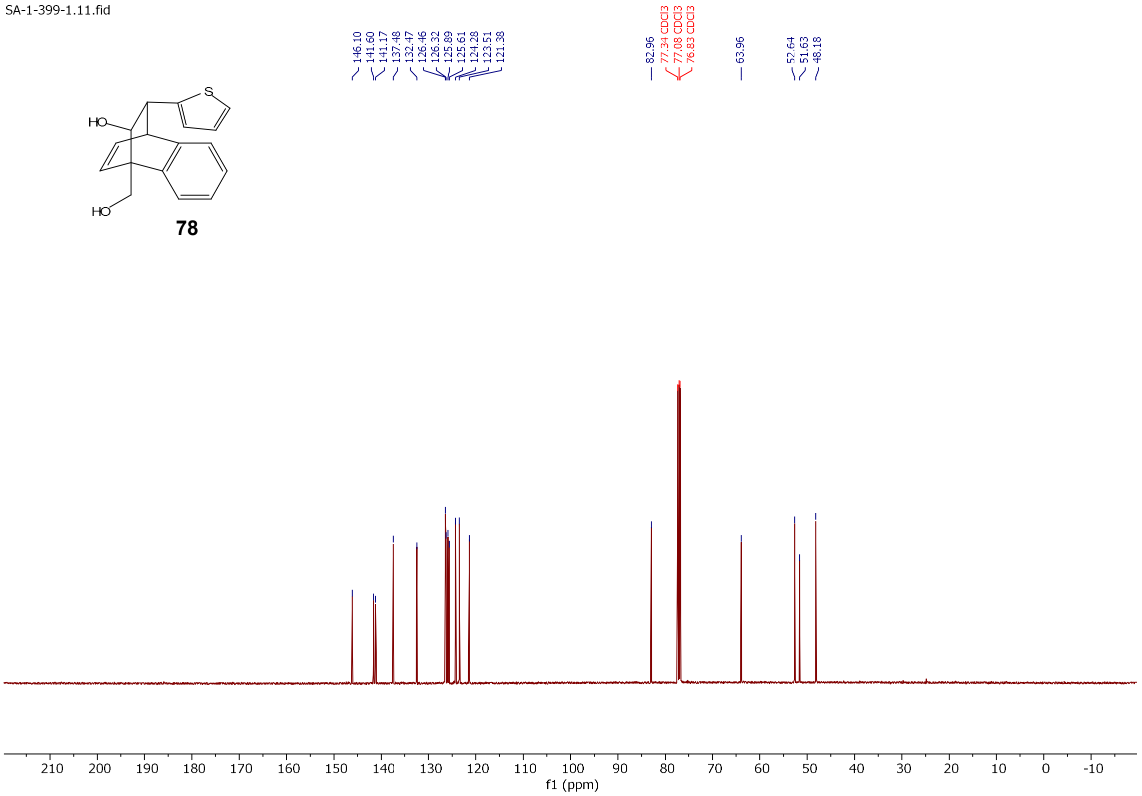
SA-1-399-1.11.fid
146.10 141.60 141.17 137.48 132.47 126.46 126.32 125.89 125.61 124.28 123.51 121.38
82.96
77.34 CDCl3
77.08 CDCl3
76.83 CDCl3
63.96
52.64 51.63 48.18
HO
S
HO
78
f1 (ppm)

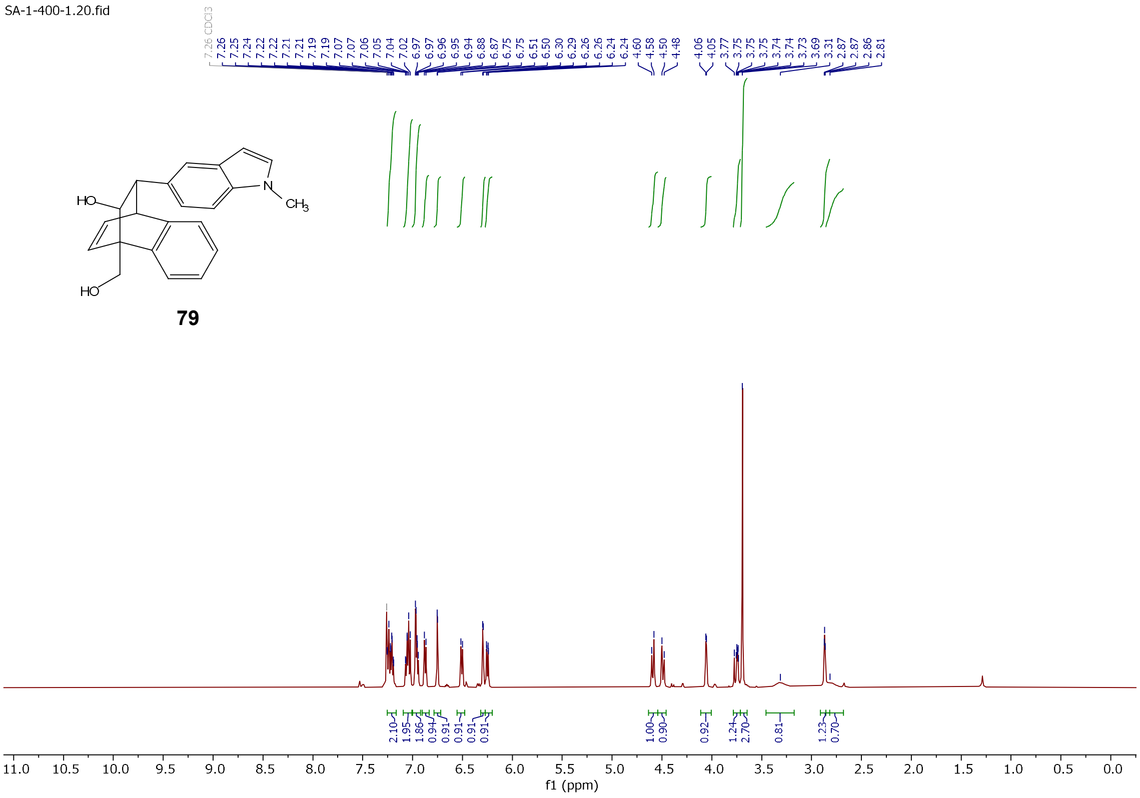

SA-1-400-1.20.fid
7.26 CDCl3
7.26 7.25 7.24 7.22 7.22 7.21 7.21 7.19 7.19 7.07 7.07 7.06 7.05 7.04 7.02 6.97 6.97 6.96 6.95 6.94 6.88 6.87 6.75 6.75 6.51 6.50 6.30 6.29 6.26 6.26 6.24 6.24 4.60 4.58 4.50 4.48 4.06 4.05 3.77 3.75 3.75 3.75 3.74 3.74 3.73 3.69 3.31 2.87 2.87 2.86 2.81
HO
N
CH3
HO
79
2.10 1.95 1.86 0.94 0.91 0.91 0.91 0.91 1.00 0.90 0.92 1.24 2.70 0.81 1.23 0.70
11.0 10.5 10.0 9.5 9.0 8.5 8.0 7.5 7.0 6.5 6.0 5.5 5.0 4.5 4.0 3.5 3.0 2.5 2.0 1.5 1.0 0.5 0.0
f1 (ppm)

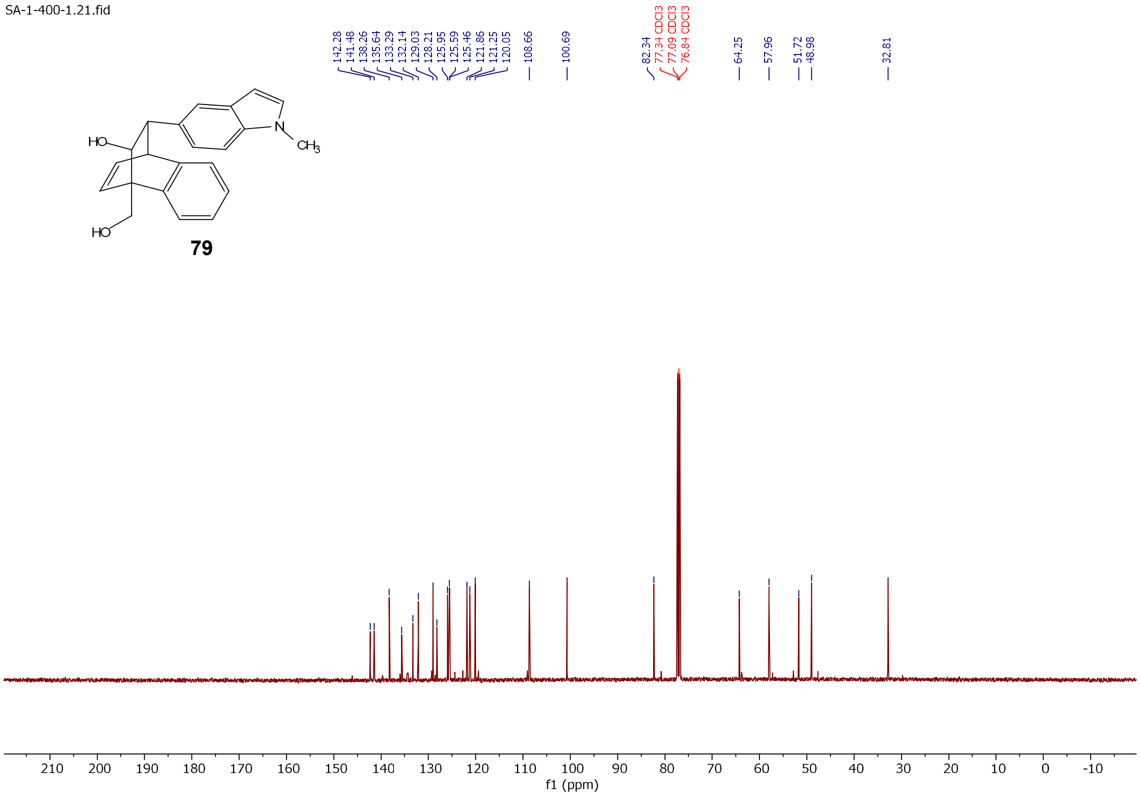

SA-1-400-1.21.fid
142.28 141.48 138.26 135.64 133.29 132.14 129.03 128.21 125.95 125.59 125.46 121.86 121.25 120.05 108.66 100.69
82.34 77.34 CDCl3 77.09 CDCl3 76.84 CDCl3
64.25 57.96 51.72 48.98 32.81
HO
N
CH3
HO
79
210 200 190 180 170 160 150 140 130 120 110 100 90 80 70 60 50 40 30 20 10 0 -10
f1 (ppm)

SA-1-404-1.10.fid

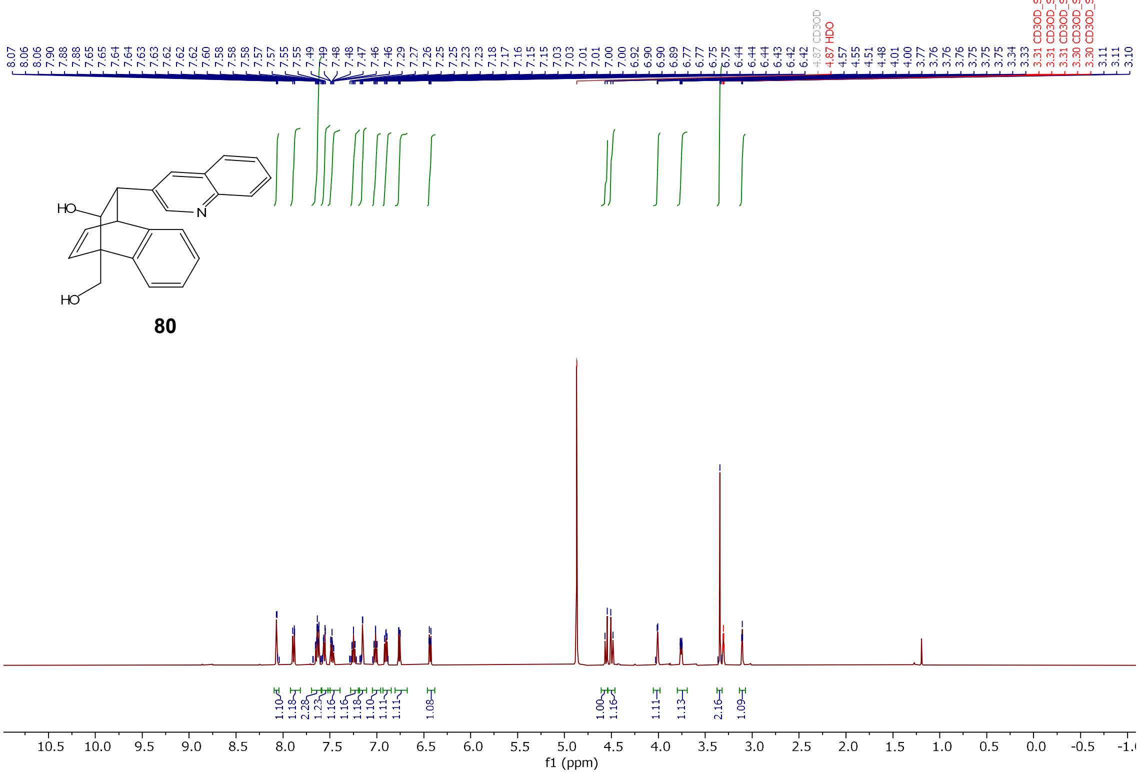

SA-1-404-1.11.fid

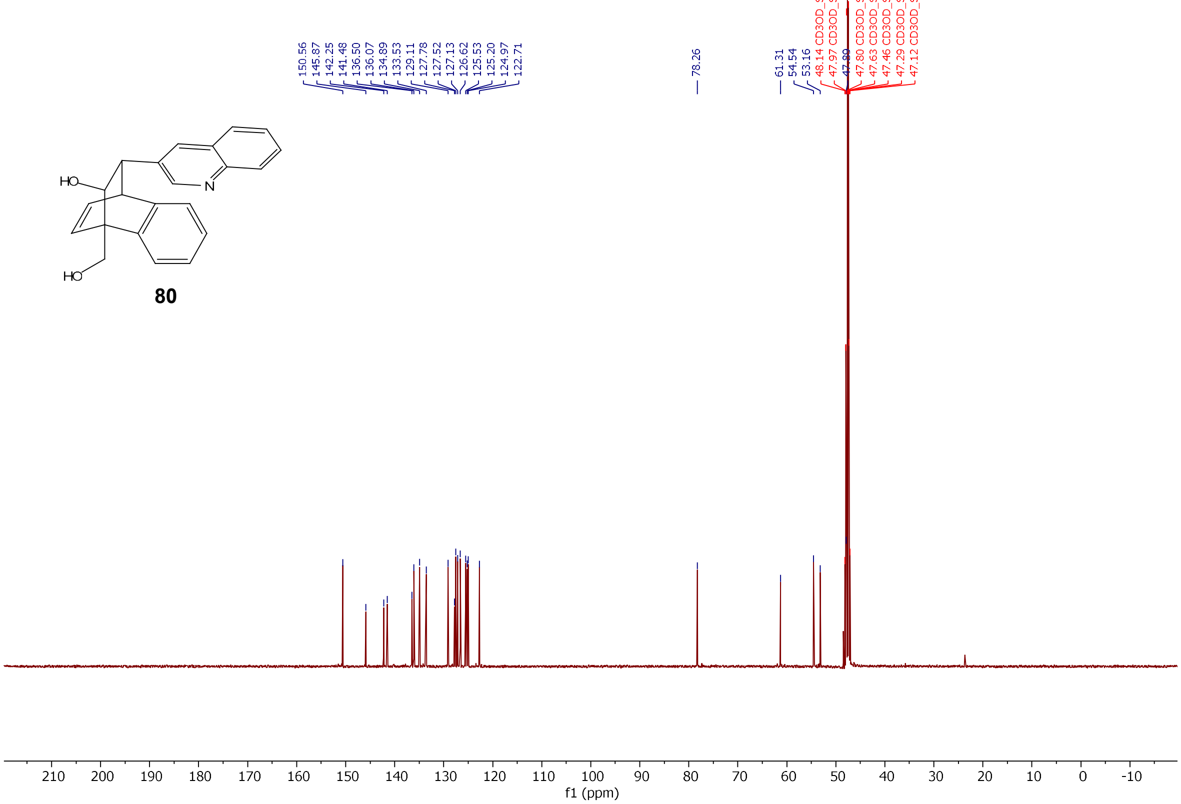

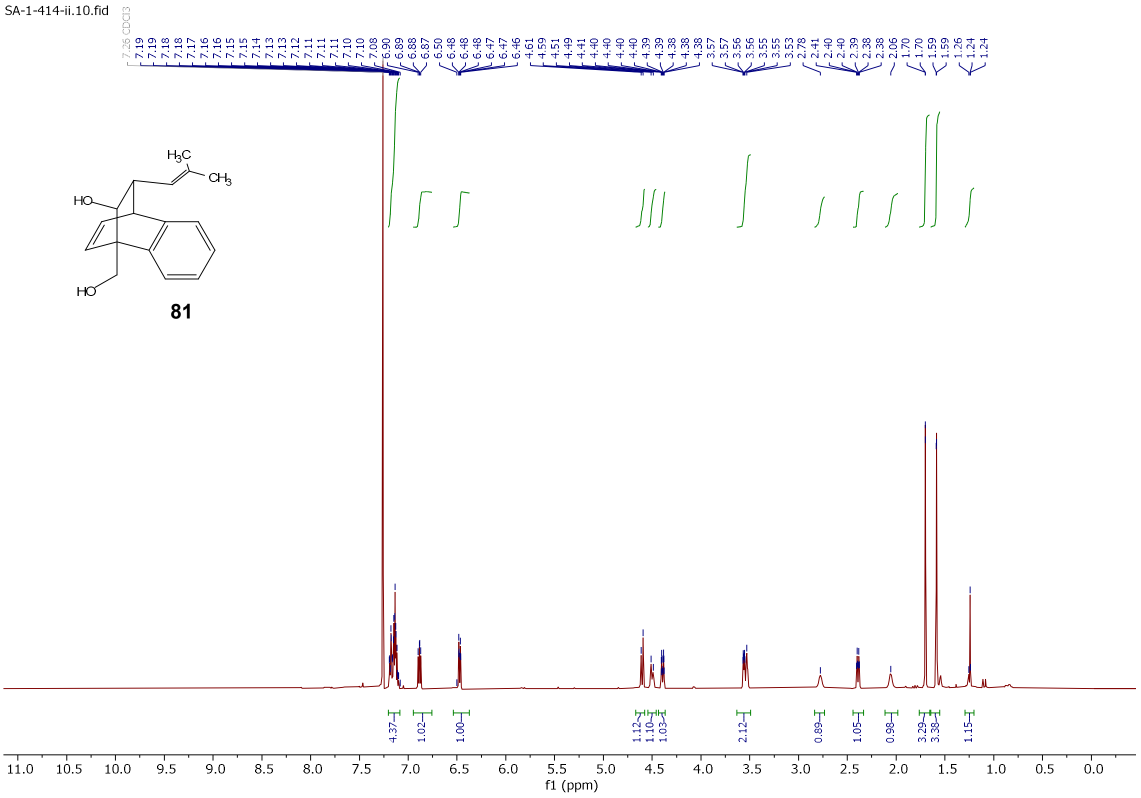
SA-1-414-ii.10.fid
81
f1 (ppm)

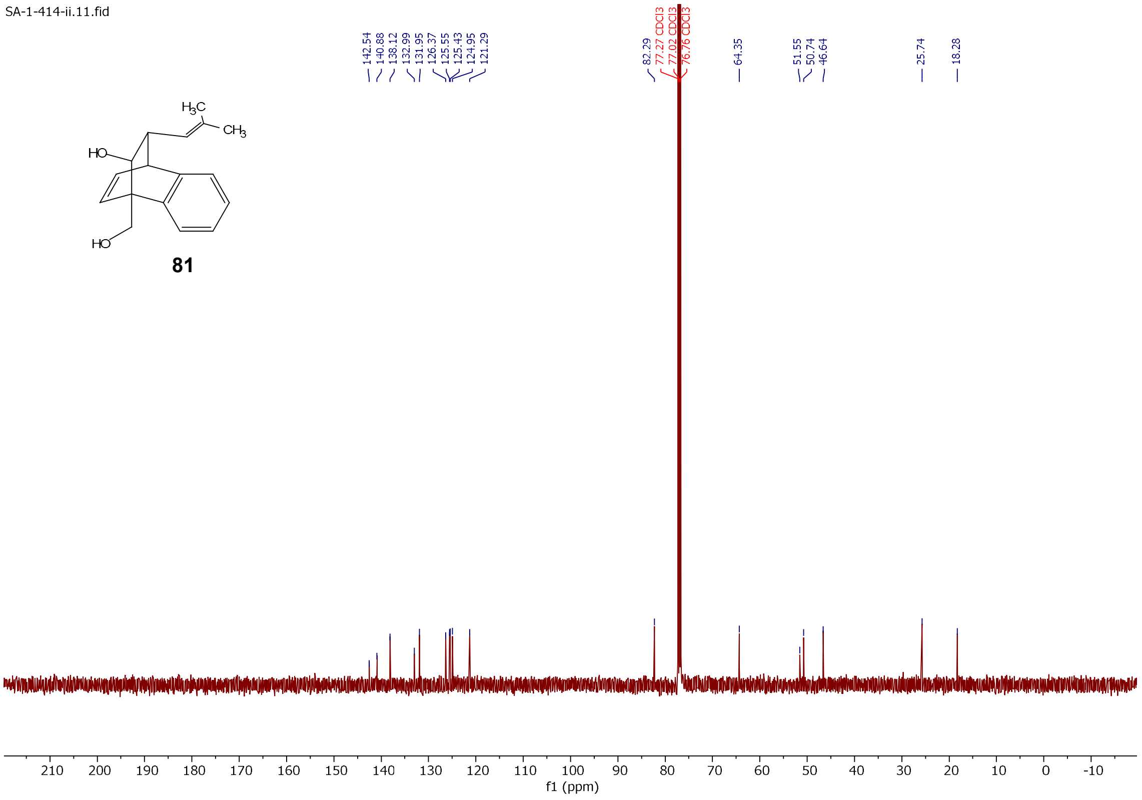
SA-1-414-ii.11.fid
81
f1 (ppm)

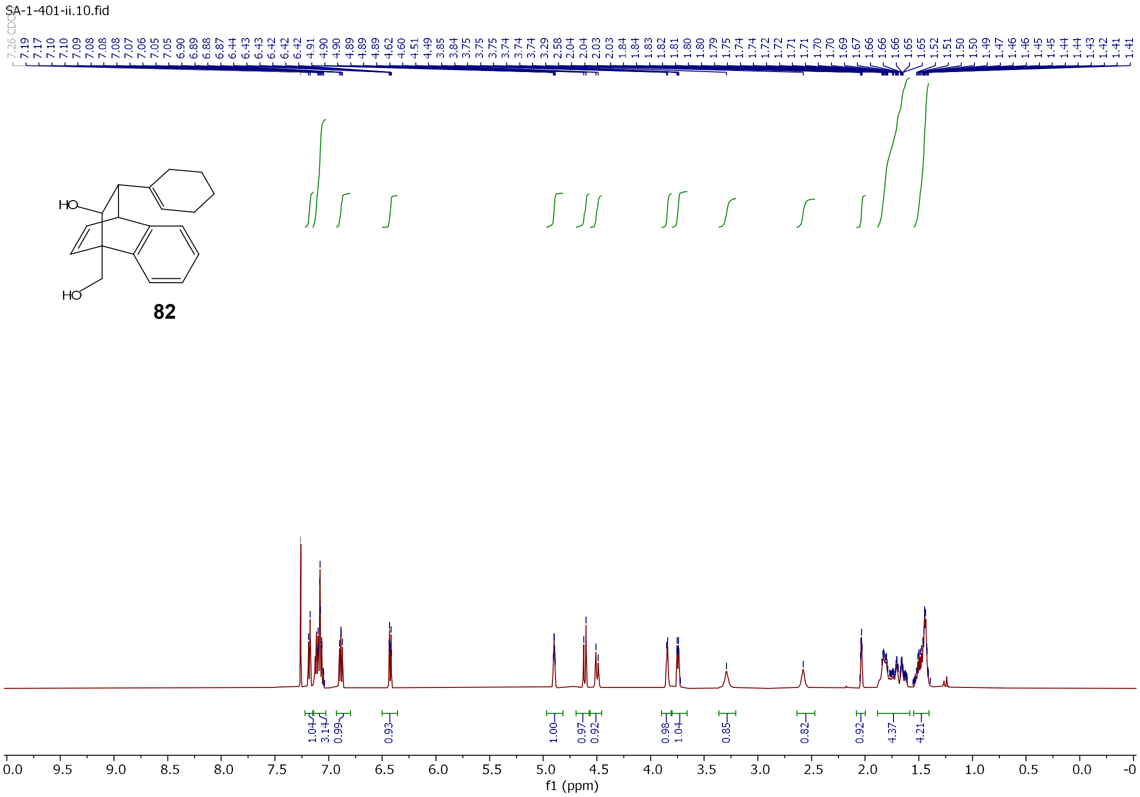
SA-1-401-ii.10.fid
7.26 CDCl3
7.19 7.17 7.10 7.10 7.09 7.08 7.08 7.08 7.07 7.06 7.05 7.05 6.90 6.89 6.88 6.87 6.44 6.43 6.43 6.42 6.42 4.91 4.90 4.90 4.89 4.89 4.89 4.62 4.60 4.51 4.49 3.85 3.84 3.75 3.75 3.75 3.74 3.74 3.74 3.29 2.58 2.04 2.04 2.03 2.03 1.84 1.84 1.83 1.82 1.81 1.80 1.80 1.79 1.75 1.74 1.74 1.72 1.72 1.71 1.71 1.70 1.70 1.69 1.67 1.66 1.66 1.66 1.65 1.65 1.52 1.51 1.50 1.50 1.49 1.47 1.46 1.45 1.45 1.44 1.44 1.43 1.42 1.41 1.41
HO
HO
82
1.04 3.14 0.99 0.93 1.00 0.97 0.92 0.98 1.04 0.85 0.82 0.92 4.37 4.21
f1 (ppm)

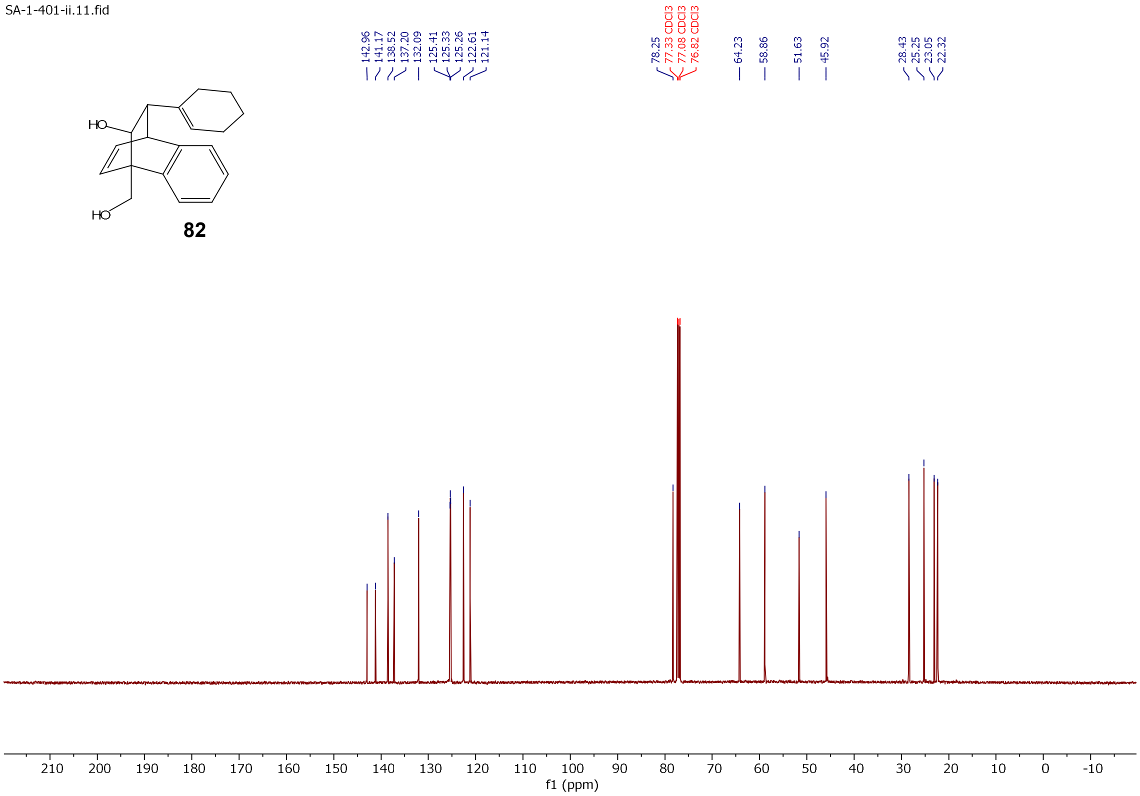
SA-1-401-ii.11.fid
142.96 141.17 138.52 137.20 132.09 125.41 125.33 125.26 122.61 121.14
78.25 77.33 CDCl3 77.08 CDCl3 76.82 CDCl3
64.23 58.86 51.63 45.92
28.43 25.25 23.05 22.32
HO
HO
82
f1 (ppm)

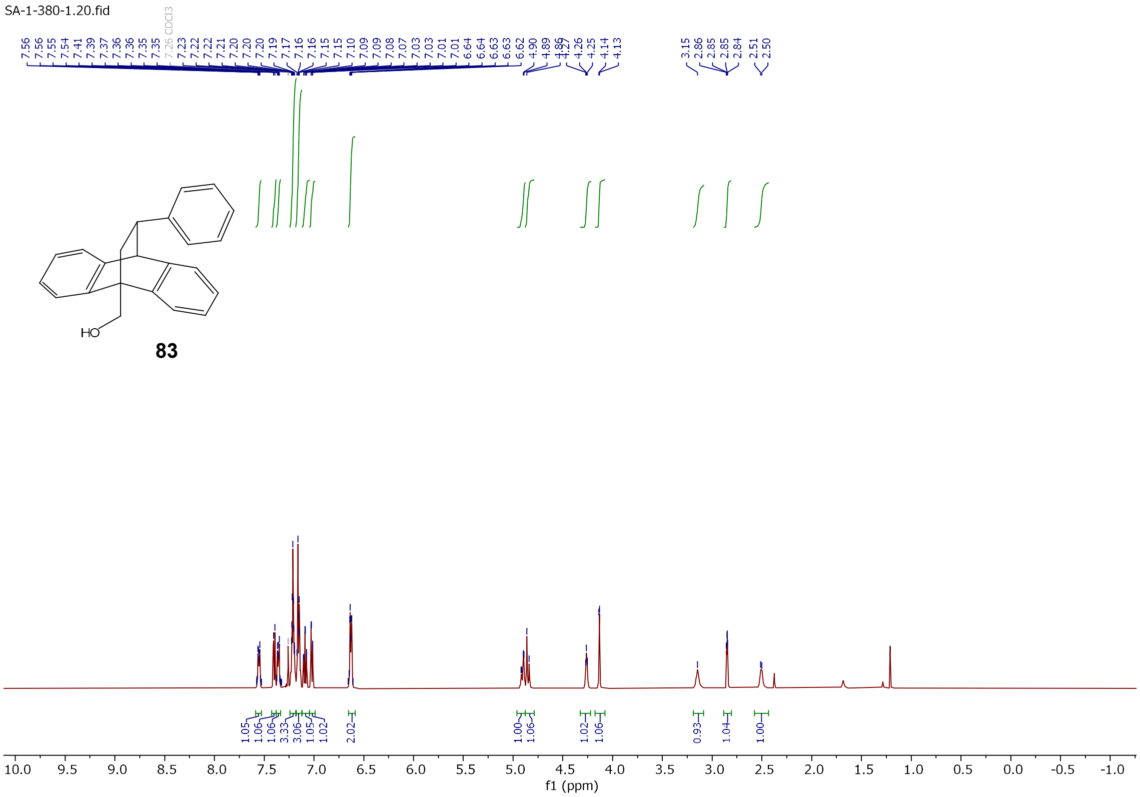
SA-1-380-1.20.fid
7.56
7.56
7.55
7.54
7.41
7.39
7.37
7.36
7.36
7.35
7.35
7.26 CDCl3
7.23
7.22
7.22
7.21
7.20
7.20
7.20
7.19
7.17
7.16
7.16
7.15
7.15
7.10
7.09
7.09
7.08
7.07
7.03
7.03
7.01
7.01
6.64
6.64
6.63
6.63
6.62
4.90
4.89
4.26
4.25
4.14
4.13
3.15
2.86
2.85
2.85
2.84
2.51
2.50
HO
1.05
1.06
1.06
3.33
3.06
1.05
1.02
2.02
1.00
1.06
1.02
1.06
0.93
1.04
1.00
10.0 9.5 9.0 8.5 8.0 7.5 7.0 6.5 6.0 5.5 5.0 4.5 4.0 3.5 3.0 2.5 2.0 1.5 1.0 0.5 0.0 -0.5 -1.0
f1 (ppm)

83

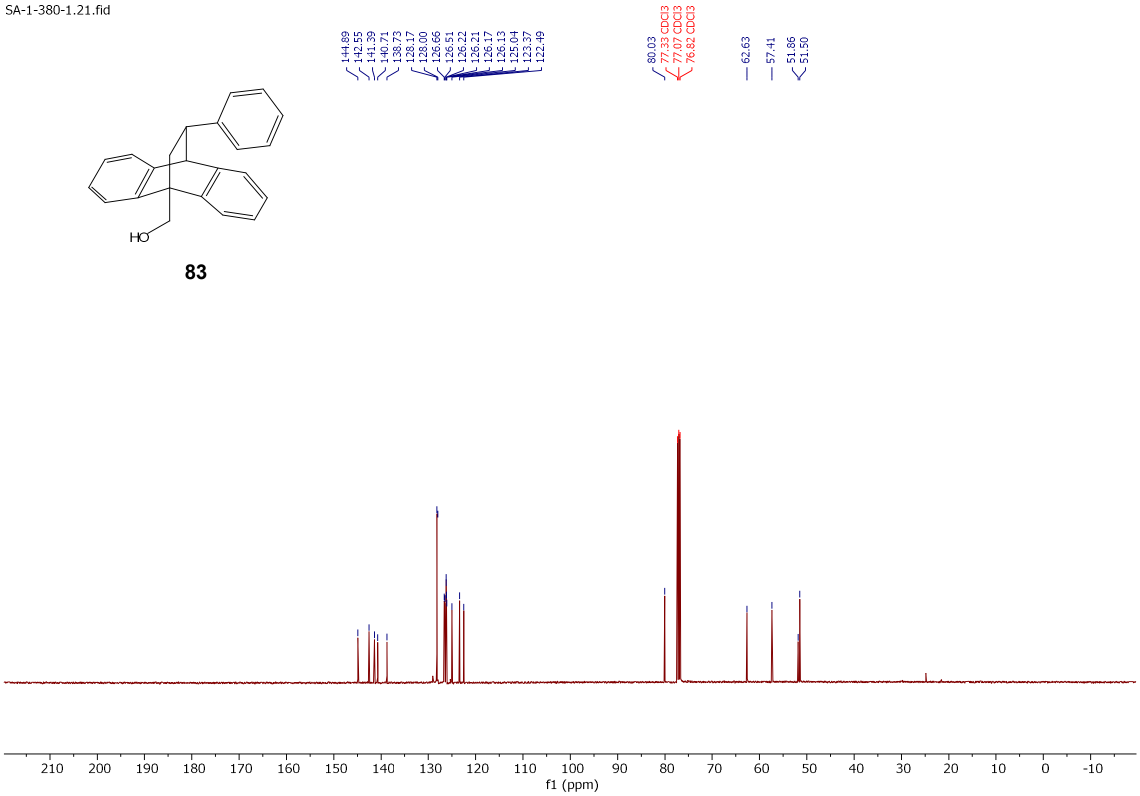
SA-1-380-1.21.fid
144.89
142.55
141.39
140.71
138.73
128.17
128.00
126.66
126.51
126.22
126.21
126.17
126.13
125.04
123.37
122.49
80.03
77.33 CDCl3
77.07 CDCl3
76.82 CDCl3
62.63
57.41
51.86
51.50
HO
210 200 190 180 170 160 150 140 130 120 110 100 90 80 70 60 50 40 30 20 10 0 -10
f1 (ppm)

83

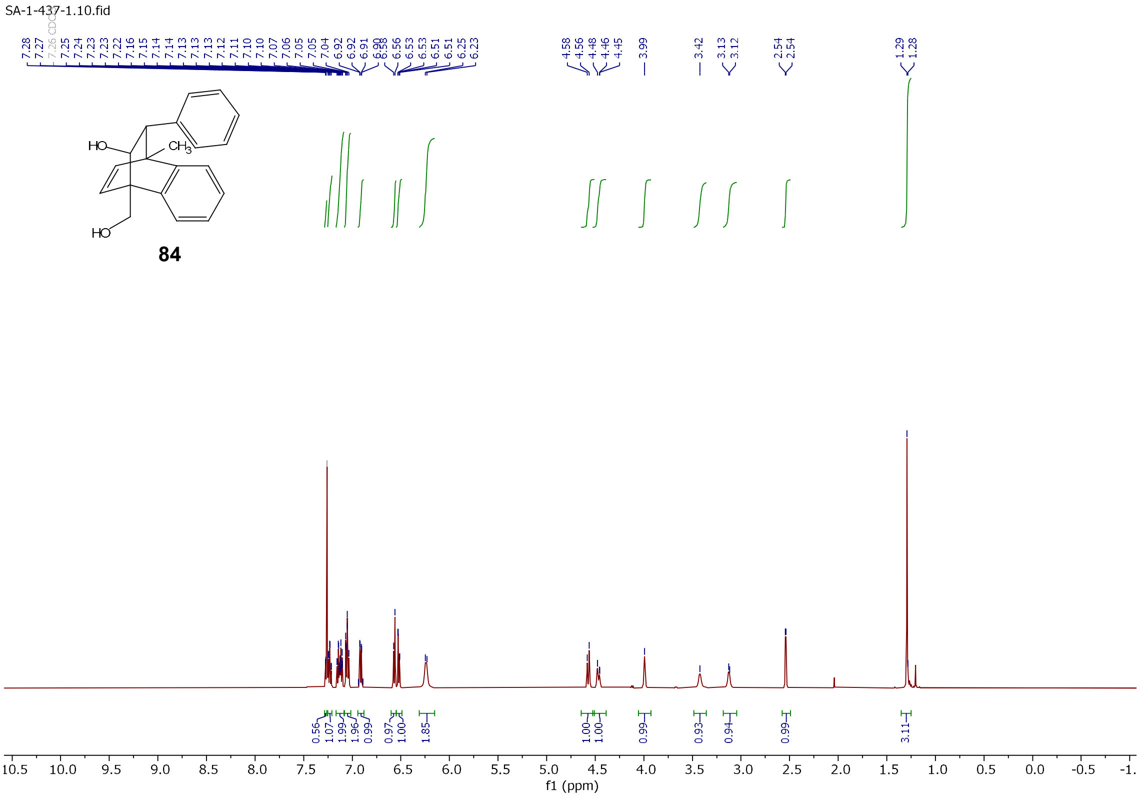
SA-1-437-1.10.fid
7.28 7.27 7.26 CDCl3 7.25 7.24 7.23 7.23 7.22 7.16 7.15 7.14 7.14 7.13 7.13 7.13 7.12 7.11 7.10 7.10 7.07 7.06 7.05 7.05 7.04 6.92 6.92 6.91 6.90 6.58 6.56 6.53 6.53 6.51 6.51 6.25 6.23
4.58 4.56 4.48 4.46 4.45
3.99
3.42
3.13 3.12
2.54 2.54
1.29 1.28
HO
CH3
HO
84
0.56 1.07 1.99 1.96 0.99 0.97 1.00 1.85
1.00 1.00
0.99
0.93
0.94
0.99
3.11
10.5 10.0 9.5 9.0 8.5 8.0 7.5 7.0 6.5 6.0 5.5 5.0 4.5 4.0 3.5 3.0 2.5 2.0 1.5 1.0 0.5 0.0 -0.5 -1.
f1 (ppm)

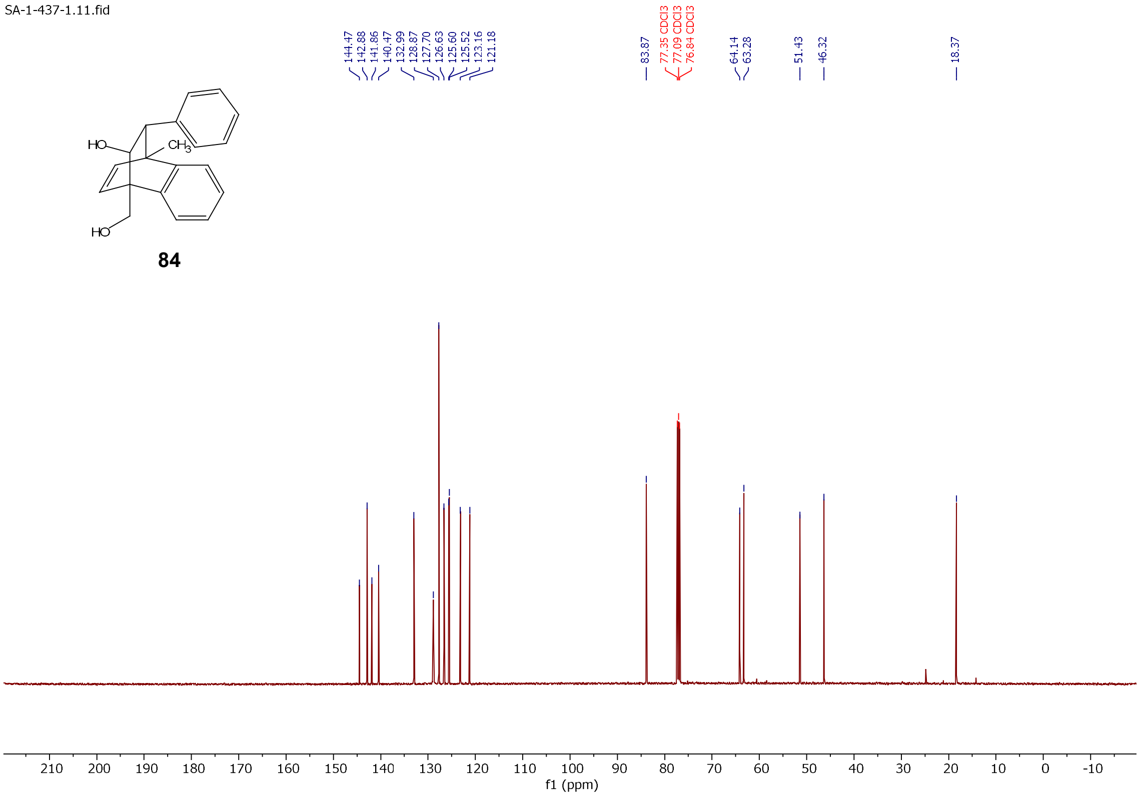
SA-1-437-1.11.fid
144.47 142.88 141.86 140.47 132.99 128.87 127.70 126.63 125.60 125.52 123.16 121.18
83.87
77.35 CDCl3 77.09 CDCl3 76.84 CDCl3
64.14 63.28
51.43
46.32
18.37
HO
CH3
HO
84
210 200 190 180 170 160 150 140 130 120 110 100 90 80 70 60 50 40 30 20 10 0 -10
f1 (ppm)

SA-1-441-1.10.fid

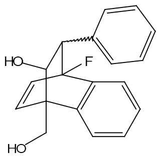

85

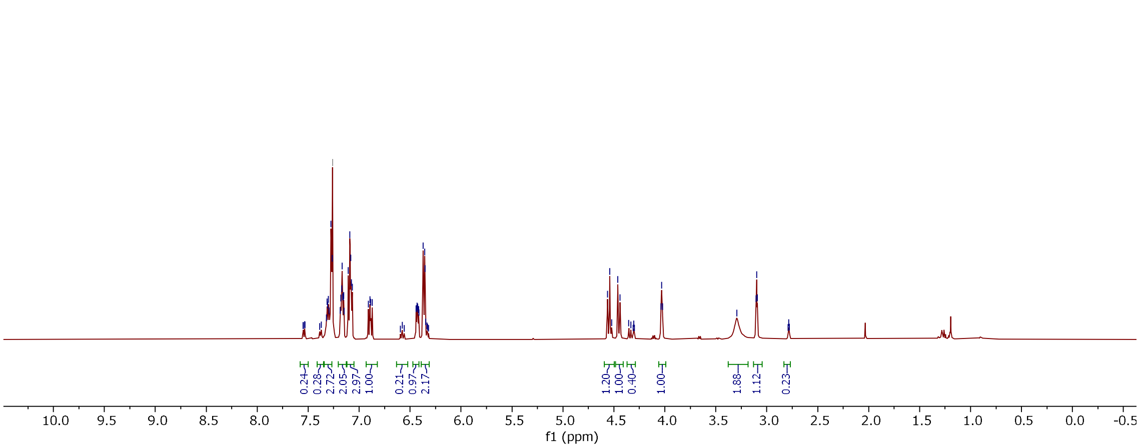

SA-1-441-1.11.fid

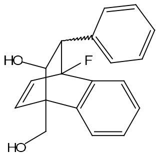

85

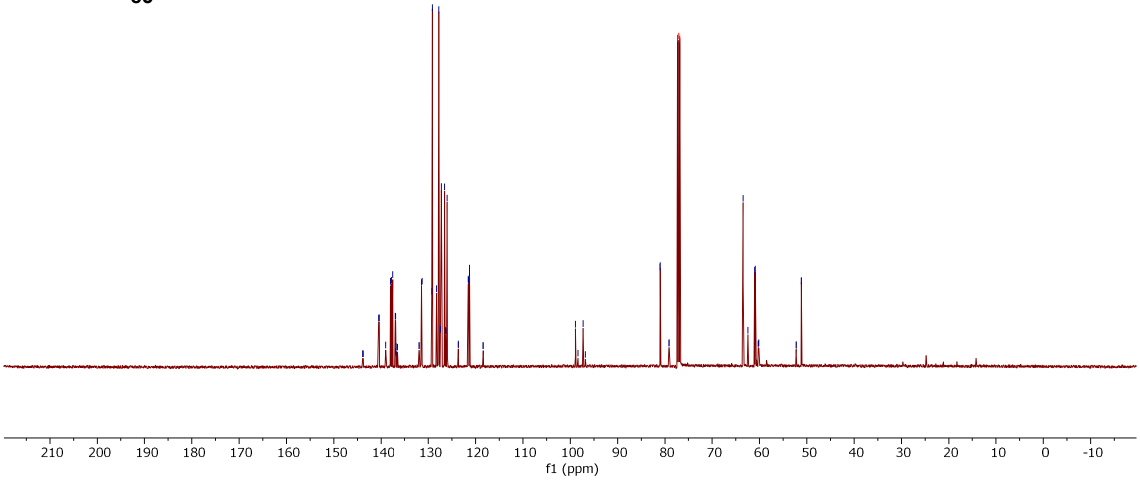

SA-1-441-1.12.fid

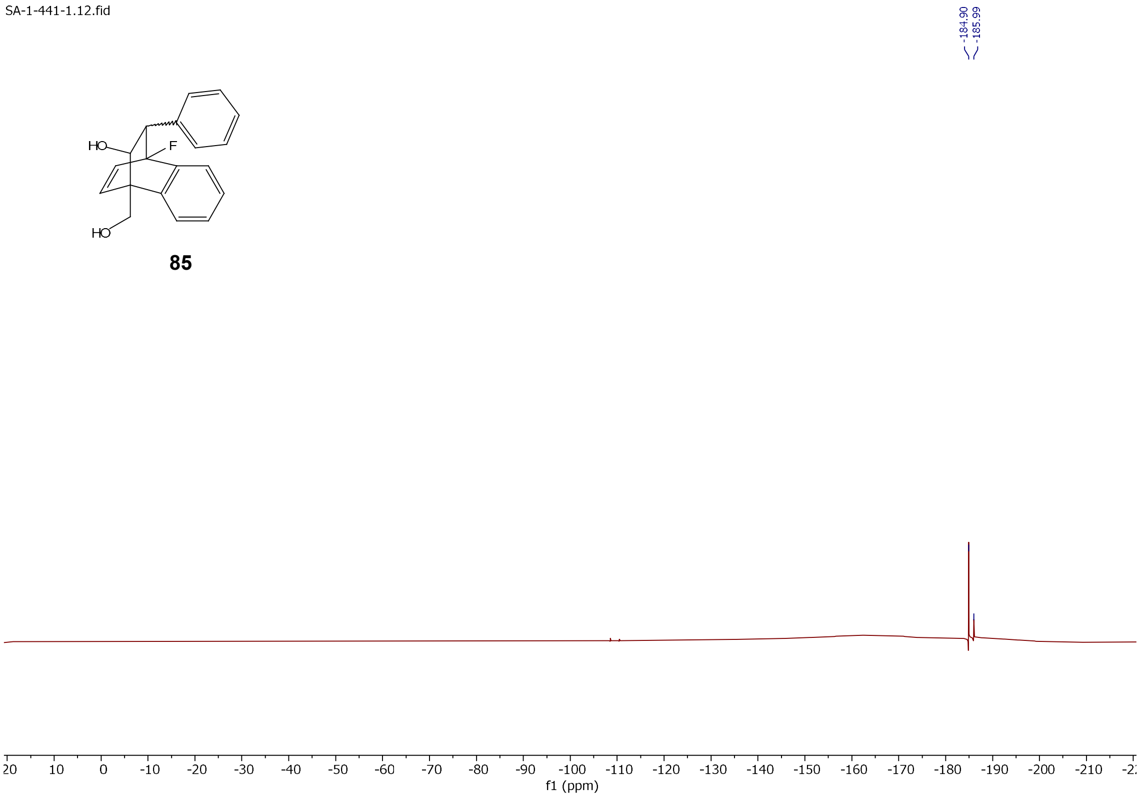

85

SA-1-501-1.10.fid

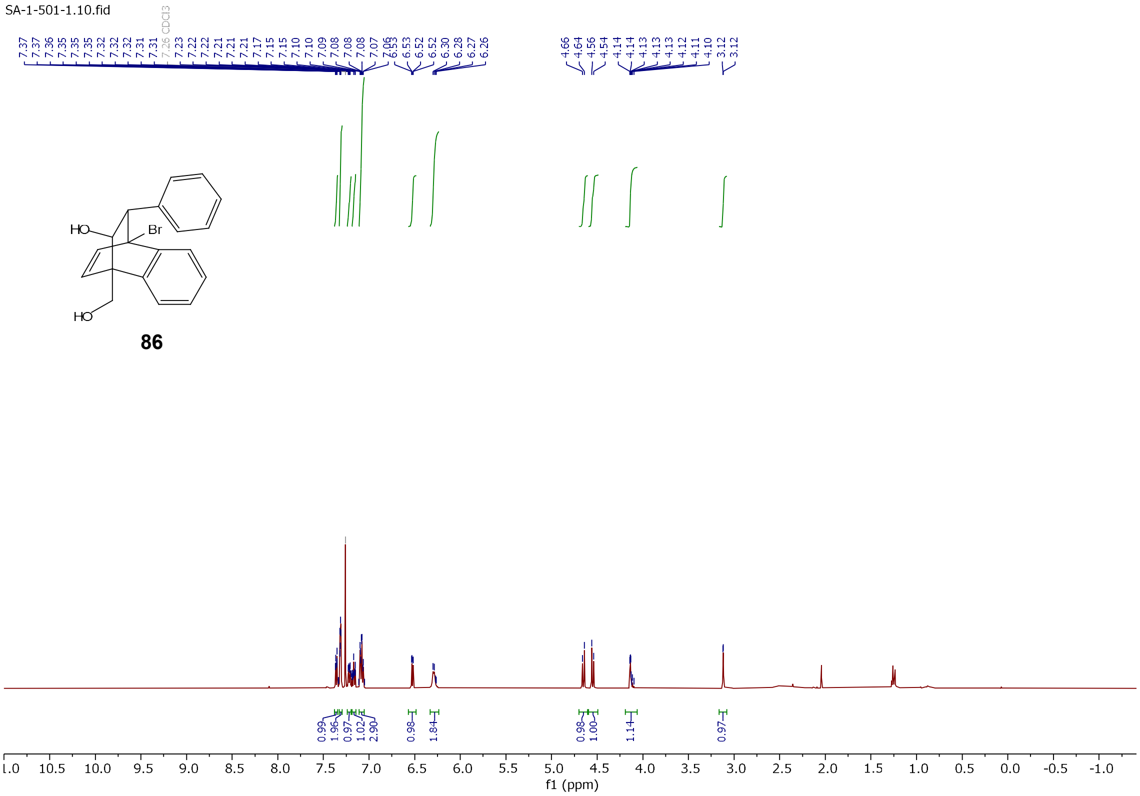

86

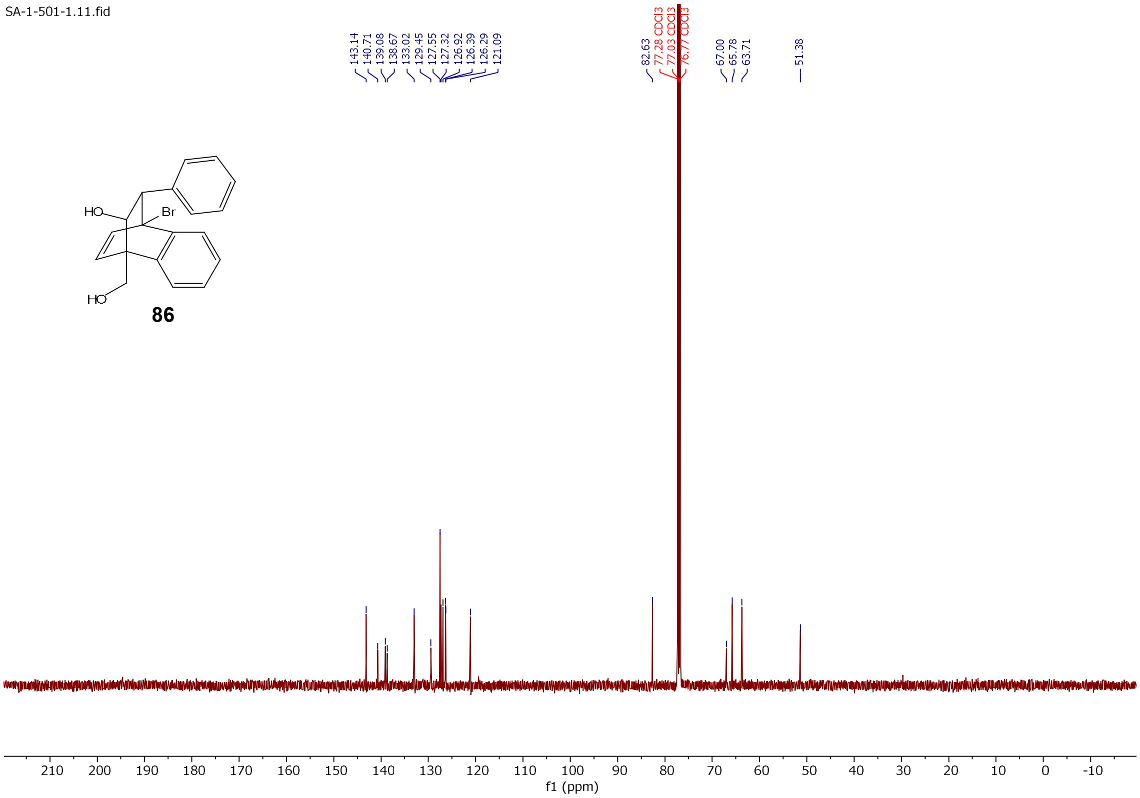
SA-1-501-1.11.fid
143.14
140.71
139.08
138.67
133.02
129.45
127.55
127.32
126.92
126.39
126.29
121.09
82.63
77.28 CDCl3
77.03 CDCl3
76.77 CDCl3
67.00
65.78
63.71
51.38
210 200 190 180 170 160 150 140 130 120 110 100 90 80 70 60 50 40 30 20 10 0 -10
f1 (ppm)
HO
Br
HO

86

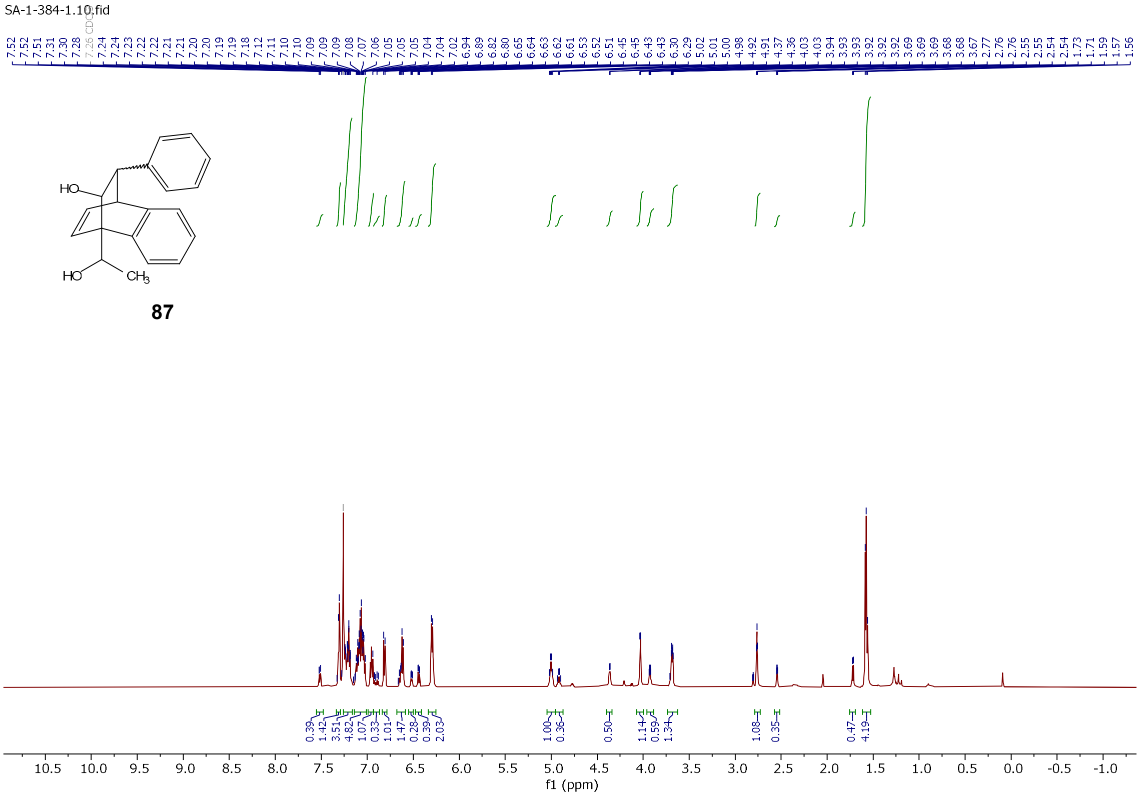
SA-1-384-1.10.fid
7.52 7.52 7.51 7.31 7.30 7.28 7.26 CDCl3 7.24 7.23 7.22 7.22 7.21 7.21 7.20 7.20 7.19 7.19 7.18 7.12 7.11 7.10 7.10 7.09 7.09 7.09 7.08 7.07 7.06 7.05 7.05 7.05 7.04 7.04 7.02 6.94 6.89 6.82 6.80 6.65 6.64 6.63 6.62 6.61 6.53 6.52 6.51 6.45 6.45 6.43 6.43 6.30 6.29 5.02 5.01 5.00 4.98 4.92 4.91 4.37 4.36 4.03 4.03 3.94 3.93 3.93 3.92 3.92 3.69 3.69 3.68 3.68 3.67 2.77 2.76 2.76 2.55 2.55 2.54 2.54 1.73 1.71 1.59 1.57 1.56
0.39 1.42 3.51 4.82 1.07 0.33 1.01 1.47 0.28 0.39 2.03 1.00 0.36 0.50 1.14 0.59 1.34 1.08 0.35 0.47 4.19
10.5 10.0 9.5 9.0 8.5 8.0 7.5 7.0 6.5 6.0 5.5 5.0 4.5 4.0 3.5 3.0 2.5 2.0 1.5 1.0 0.5 0.0 -0.5 -1.0
f1 (ppm)
HO
HO
CH3

87

SA-1-384-1.11.fid

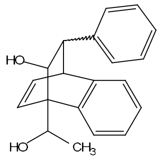

87

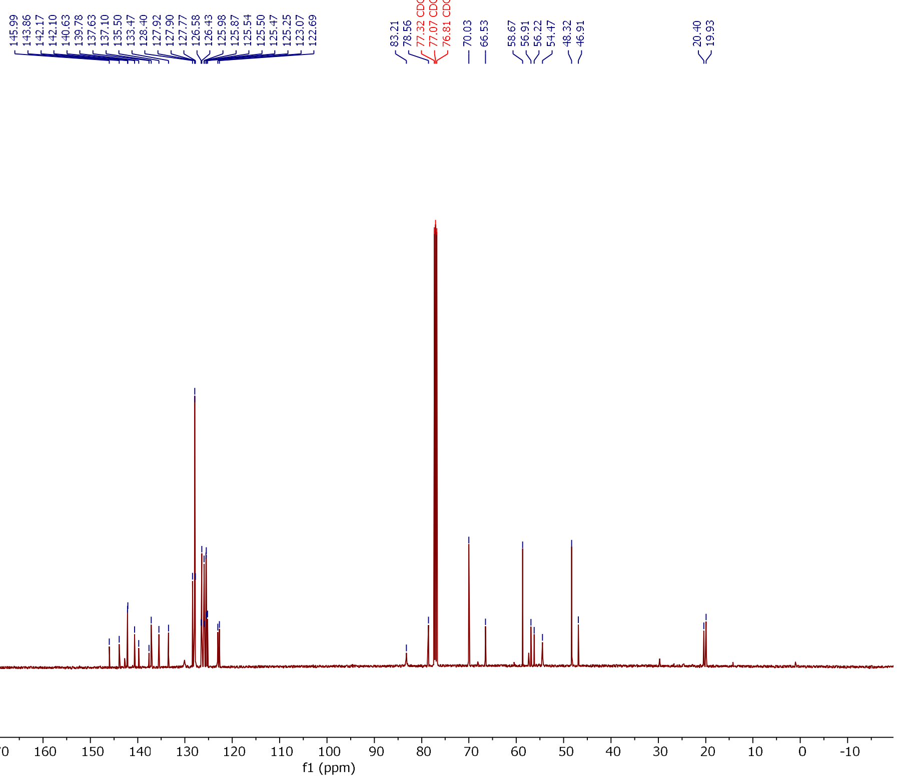

SA-1-853.10.fid

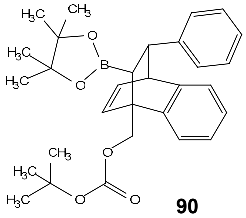

90

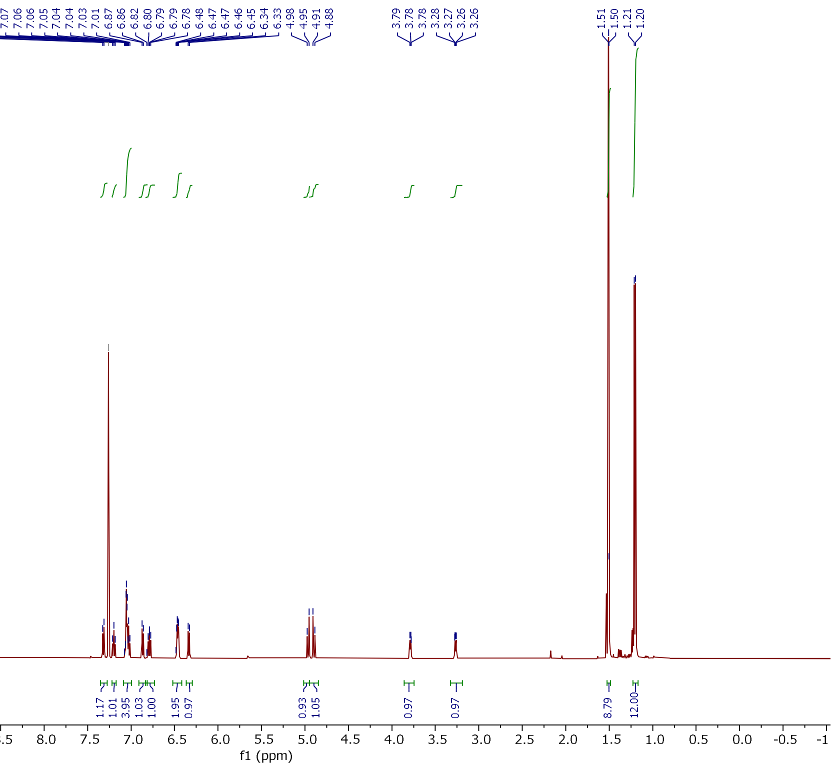

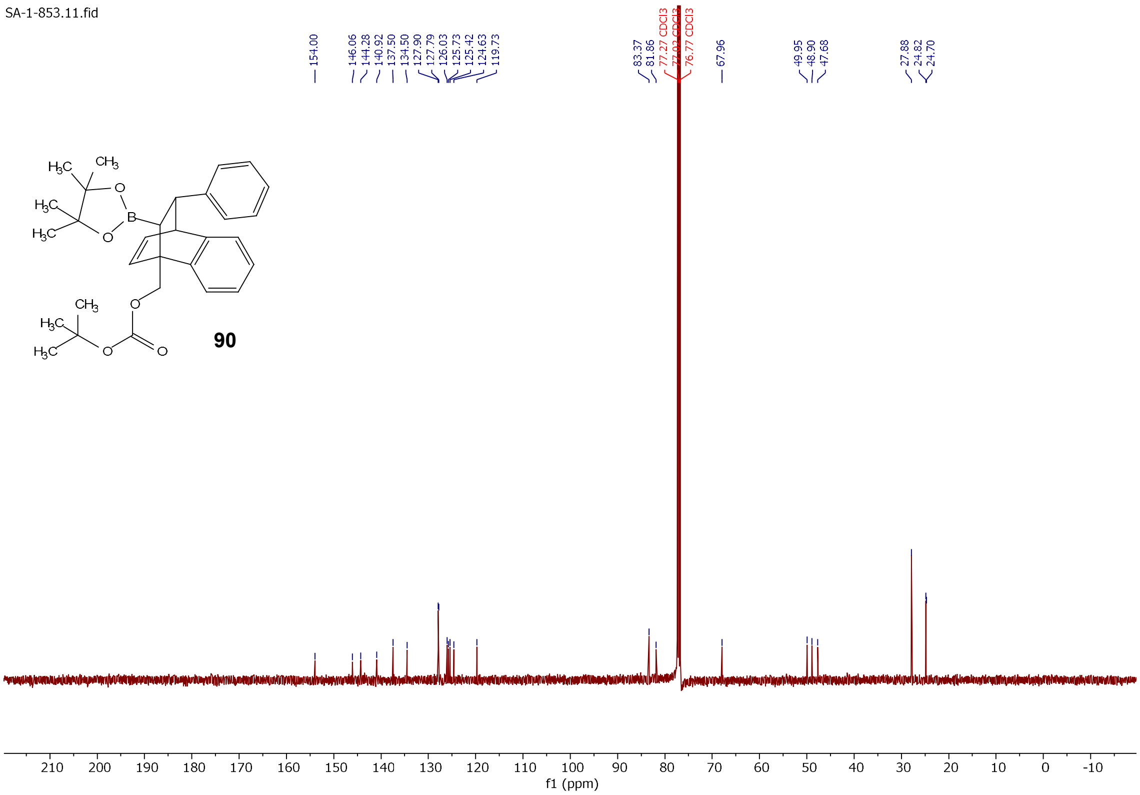
SA-1-853.11.fid
154.00
146.06
144.28
140.92
137.50
134.50
127.90
127.79
126.03
125.73
125.42
124.63
119.73
83.37
81.86
77.27 CDCl3
77.02 CDCl3
76.77 CDCl3
67.96
49.95
48.90
47.68
27.88
24.82
24.70
90
f1 (ppm)

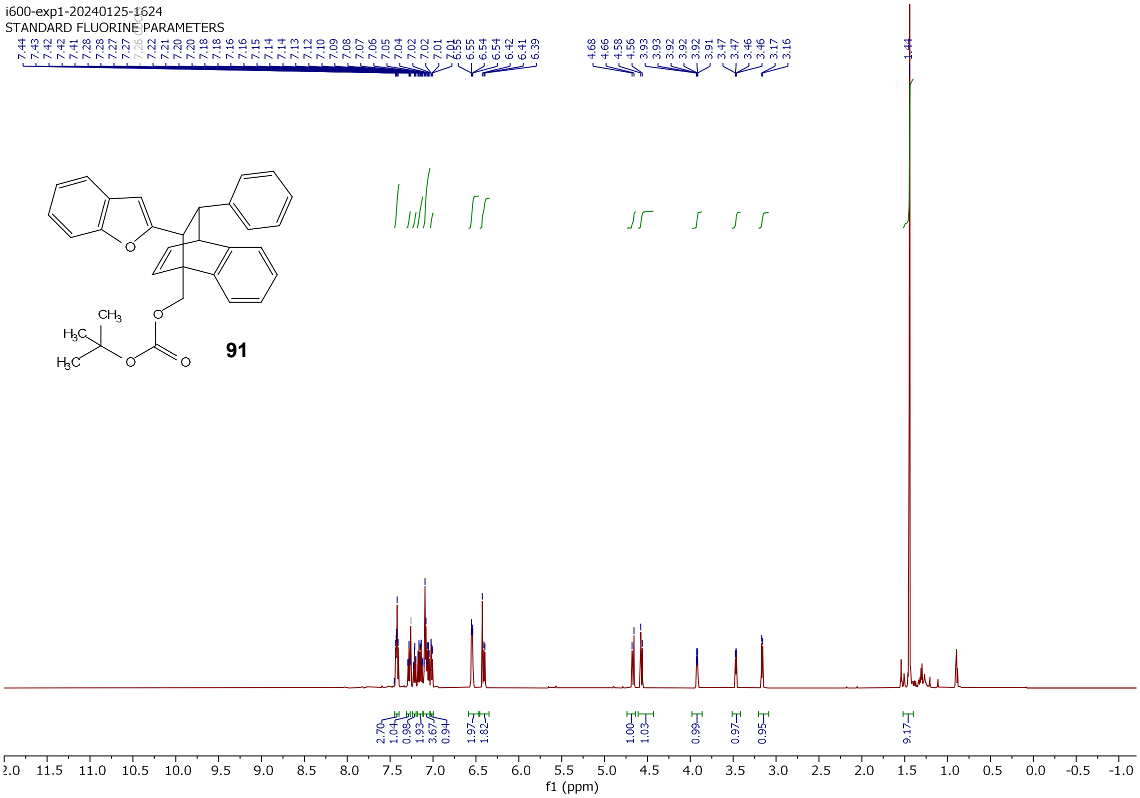
i600-exp1-20240125-1624
STANDARD FLUORINE PARAMETERS
7.44
7.43
7.42
7.41
7.28
7.27
7.27
7.26
7.22
7.21
7.20
7.18
7.18
7.16
7.16
7.15
7.14
7.14
7.13
7.12
7.10
7.09
7.08
7.07
7.06
7.05
7.04
7.02
7.02
7.01
7.01
6.55
6.55
6.54
6.54
6.42
6.41
6.39
4.68
4.66
4.58
4.56
3.93
3.93
3.92
3.92
3.91
3.47
3.47
3.46
3.46
3.17
3.16
1.44
2.70
1.04
0.98
1.93
3.67
0.94
1.97
1.82
1.00
1.03
0.99
0.97
0.95
9.17
91
f1 (ppm)

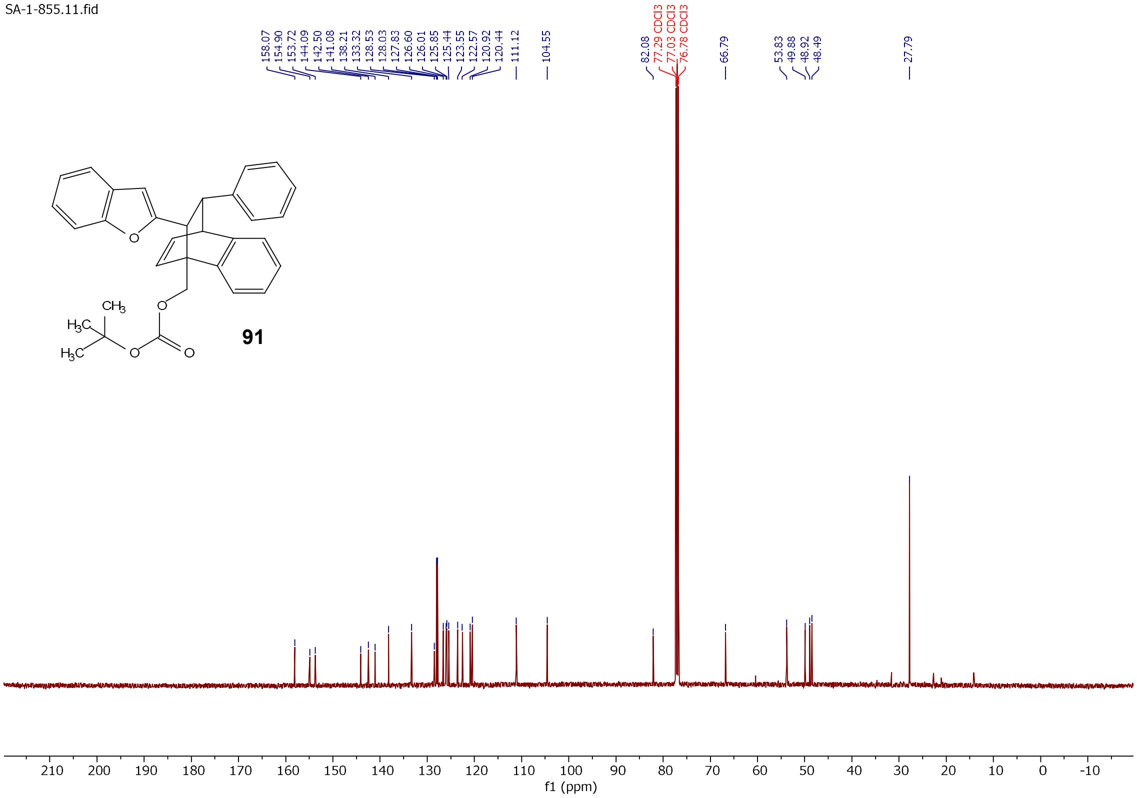
SA-1-855.11.fid
158.07
154.90
153.72
144.09
142.50
141.08
138.21
133.32
128.53
128.03
127.83
126.60
126.01
125.85
125.44
123.55
122.57
120.92
120.44
111.12
104.55
82.08
77.29 CDCl3
77.03 CDCl3
76.78 CDCl3
66.79
53.83
49.88
48.92
48.49
27.79
CH3
H3C
H3C
O
O
O
O
91
210 200 190 180 170 160 150 140 130 120 110 100 90 80 70 60 50 40 30 20 10 0 -10
f1 (ppm)

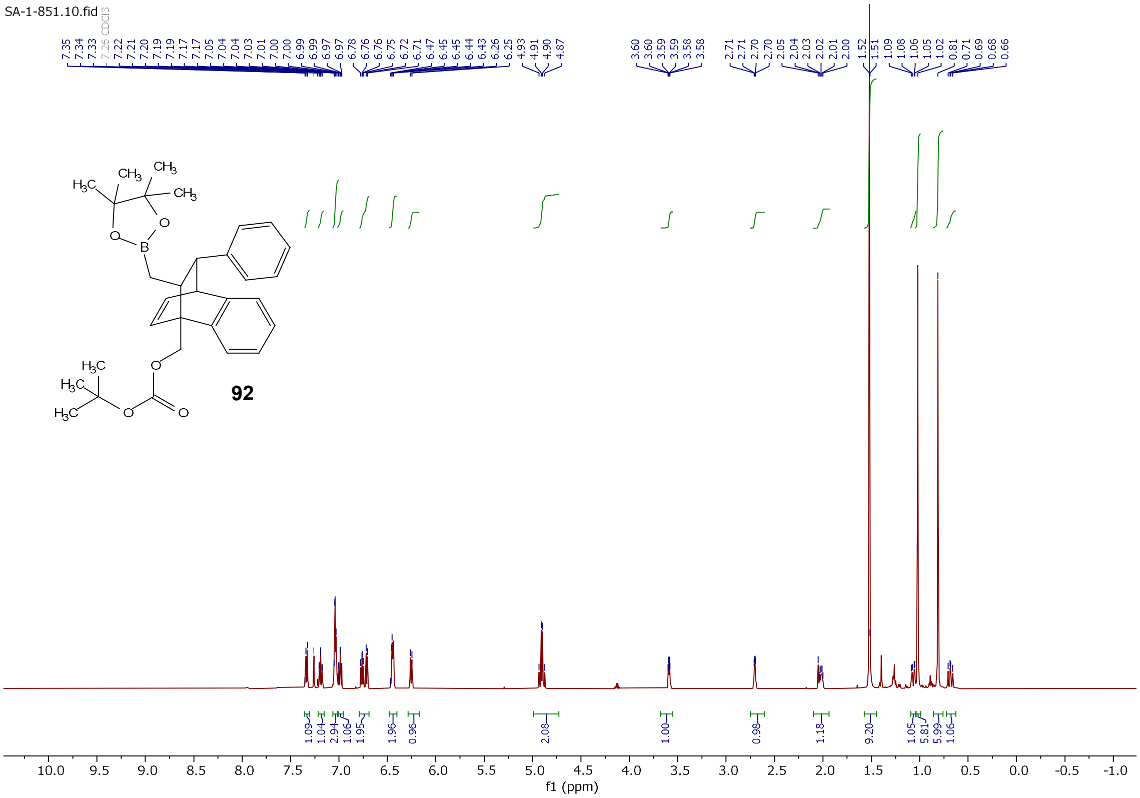
SA-1-851.10.fid
7.35
7.34
7.33
7.26 CDCl3
7.22
7.21
7.20
7.19
7.19
7.17
7.05
7.04
7.04
7.03
7.01
7.00
7.00
6.99
6.99
6.97
6.97
6.78
6.76
6.76
6.75
6.72
6.71
6.47
6.45
6.45
6.44
6.43
6.26
6.25
4.93
4.91
4.90
4.87
3.60
3.60
3.59
3.59
3.58
3.58
2.71
2.71
2.70
2.05
2.04
2.03
2.02
2.01
2.00
1.52
1.51
1.08
1.06
1.05
1.02
0.81
0.71
0.69
0.68
0.66
H3C
CH3
CH3
CH3
O
O
B
H3C
H3C
H3C
O
O
O
92
1.09
1.04
2.94
1.06
1.95
1.96
0.96
2.08
1.00
0.98
1.18
9.20
1.05
5.81
5.99
1.06
10.0 9.5 9.0 8.5 8.0 7.5 7.0 6.5 6.0 5.5 5.0 4.5 4.0 3.5 3.0 2.5 2.0 1.5 1.0 0.5 0.0 -0.5 -1.0
f1 (ppm)

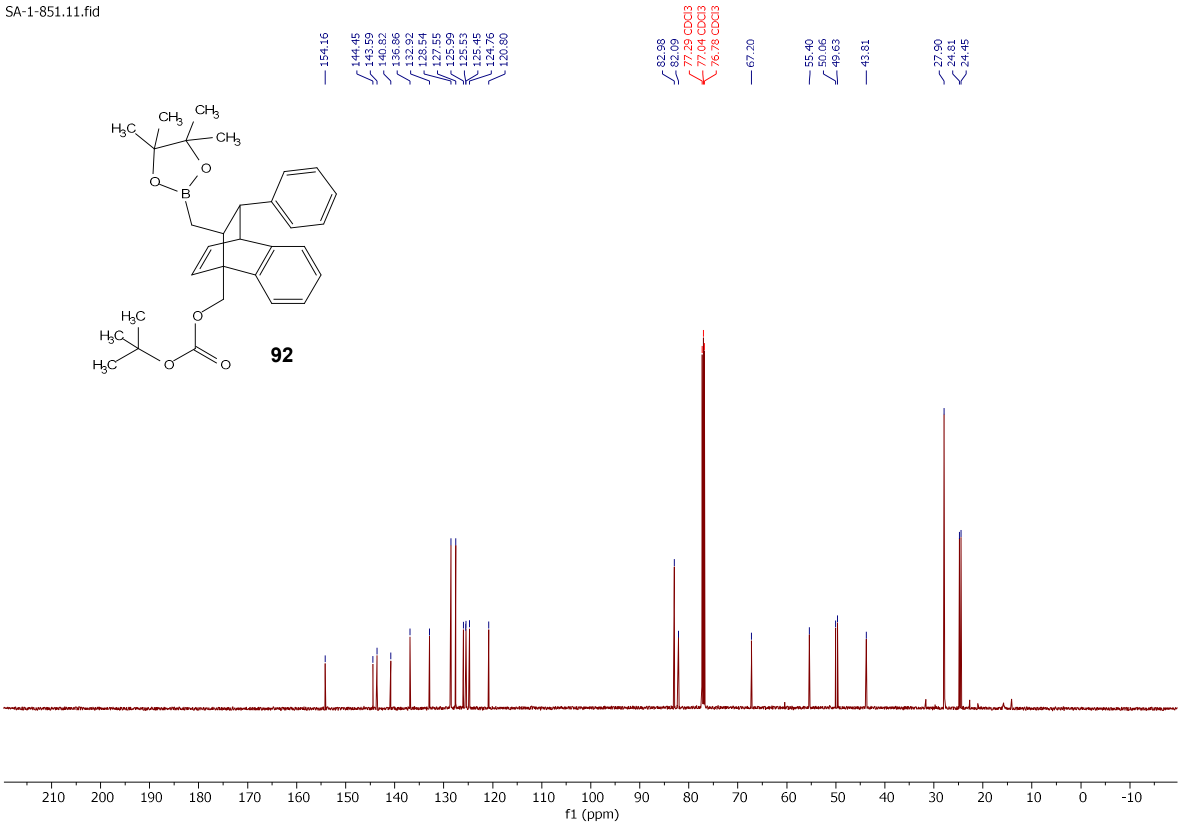
SA-1-851.11.fid
154.16
144.45
143.59
140.82
136.86
132.92
128.54
127.55
125.99
125.53
125.45
124.76
120.80
82.98
82.09
77.29 CDCl3
77.04 CDCl3
76.78 CDCl3
67.20
55.40
50.06
49.63
43.81
27.90
24.81
24.45
92
f1 (ppm)

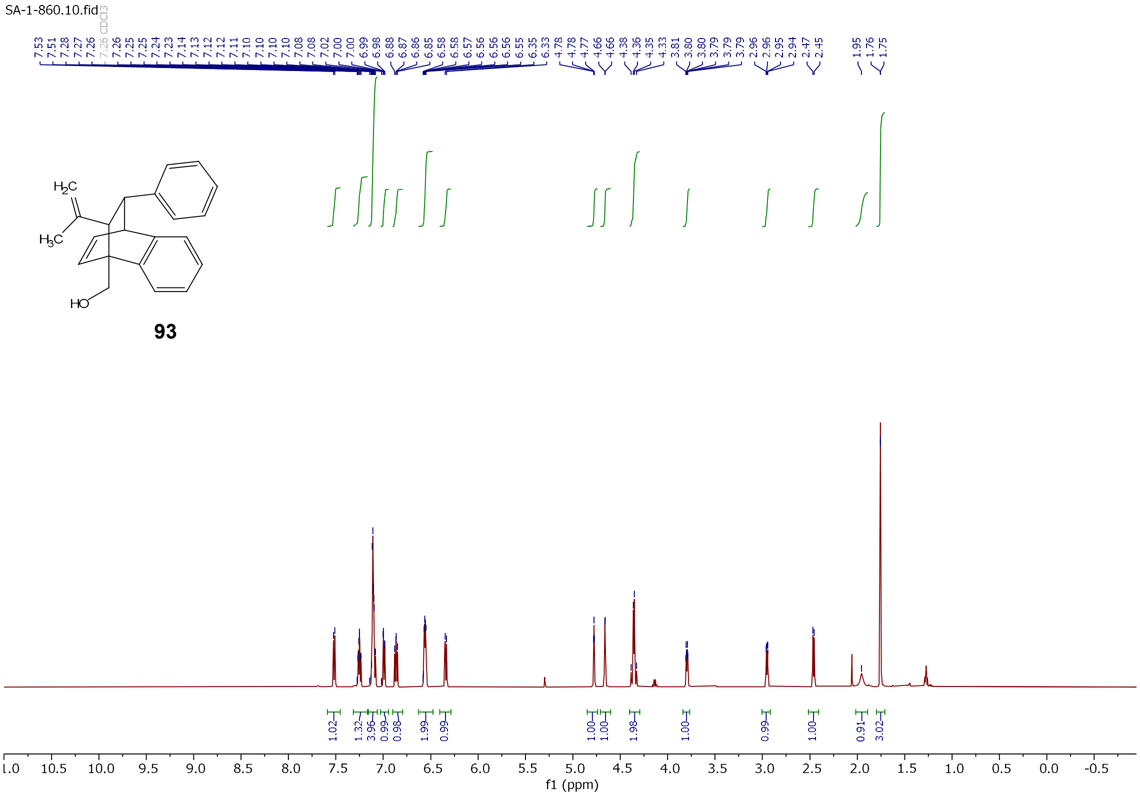
SA-1-860.10.fid
7.53
7.51
7.28
7.27
7.26
7.26 CDCl3
7.26
7.25
7.25
7.24
7.23
7.14
7.13
7.12
7.12
7.11
7.10
7.10
7.10
7.10
7.08
7.08
7.02
7.00
7.00
6.99
6.98
6.88
6.87
6.86
6.85
6.58
6.58
6.57
6.56
6.56
6.56
6.55
6.35
6.33
4.78
4.77
4.66
4.66
4.38
4.36
4.35
4.33
3.81
3.80
3.80
3.79
3.79
2.96
2.96
2.95
2.94
2.47
2.45
1.95
1.76
1.75
93
1.02
1.32
3.96
0.99
0.98
1.99
0.99
1.00
1.00
1.98
1.00
0.99
1.00
0.91
3.02
f1 (ppm)

SA-1-860.11.fid

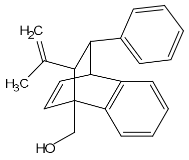

93

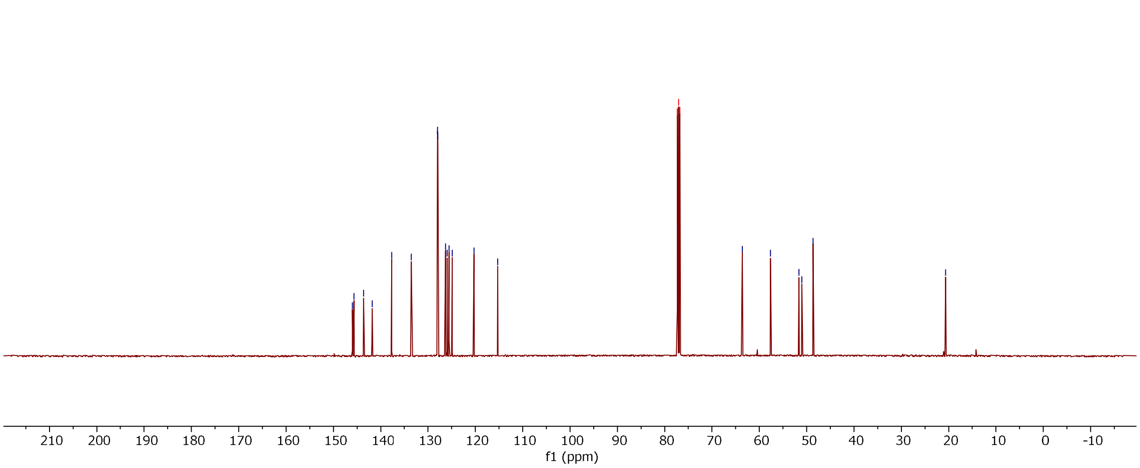

SA-1-854.10.fid

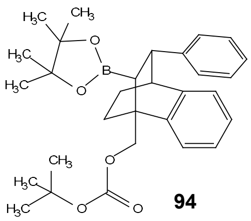

94

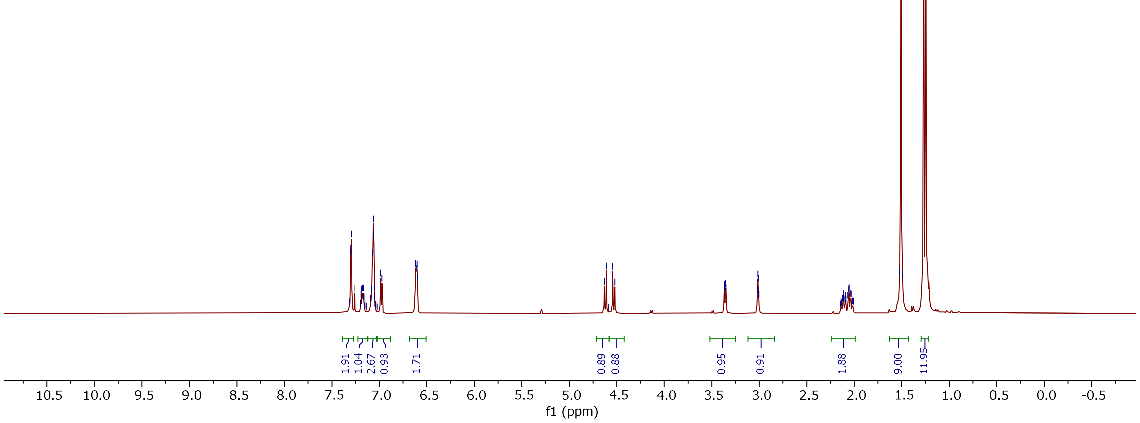

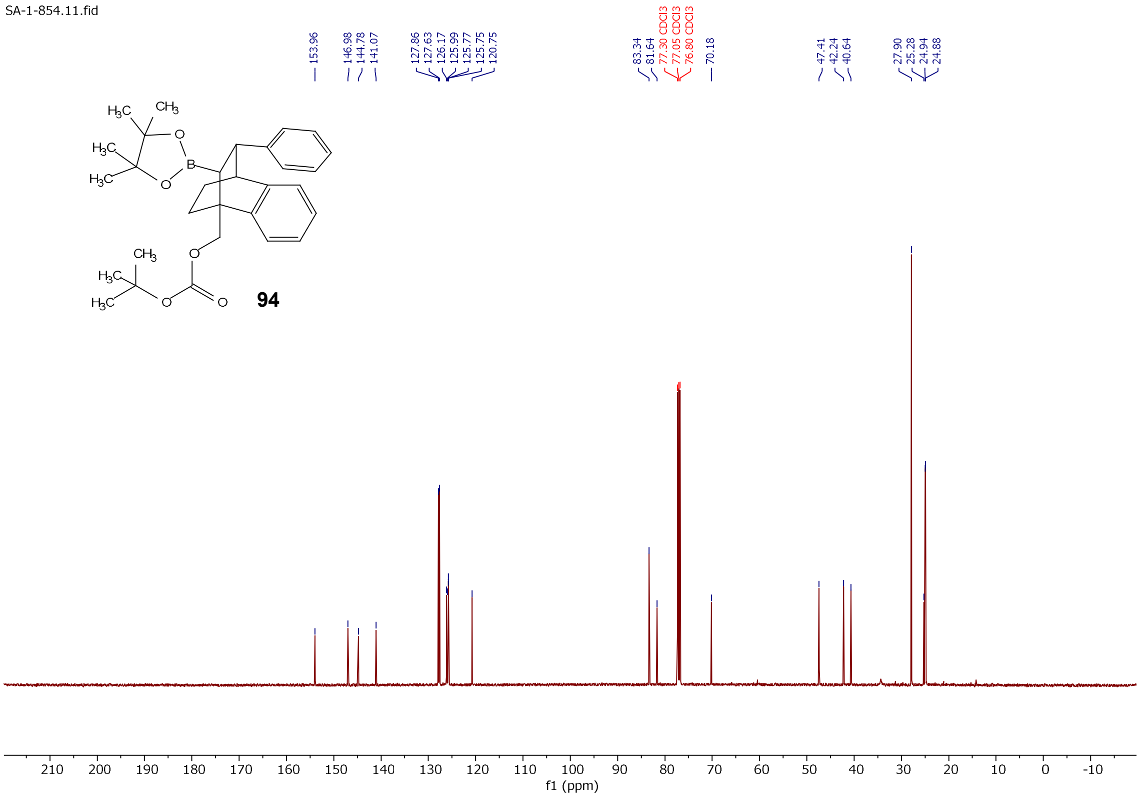
SA-1-854.11.fid
153.96
146.98
144.78
141.07
127.86
127.63
126.17
125.99
125.77
125.75
120.75
83.34
81.64
77.30 CDCl3
77.05 CDCl3
76.80 CDCl3
70.18
47.41
42.24
40.64
27.90
25.28
24.94
24.88
94
f1 (ppm)

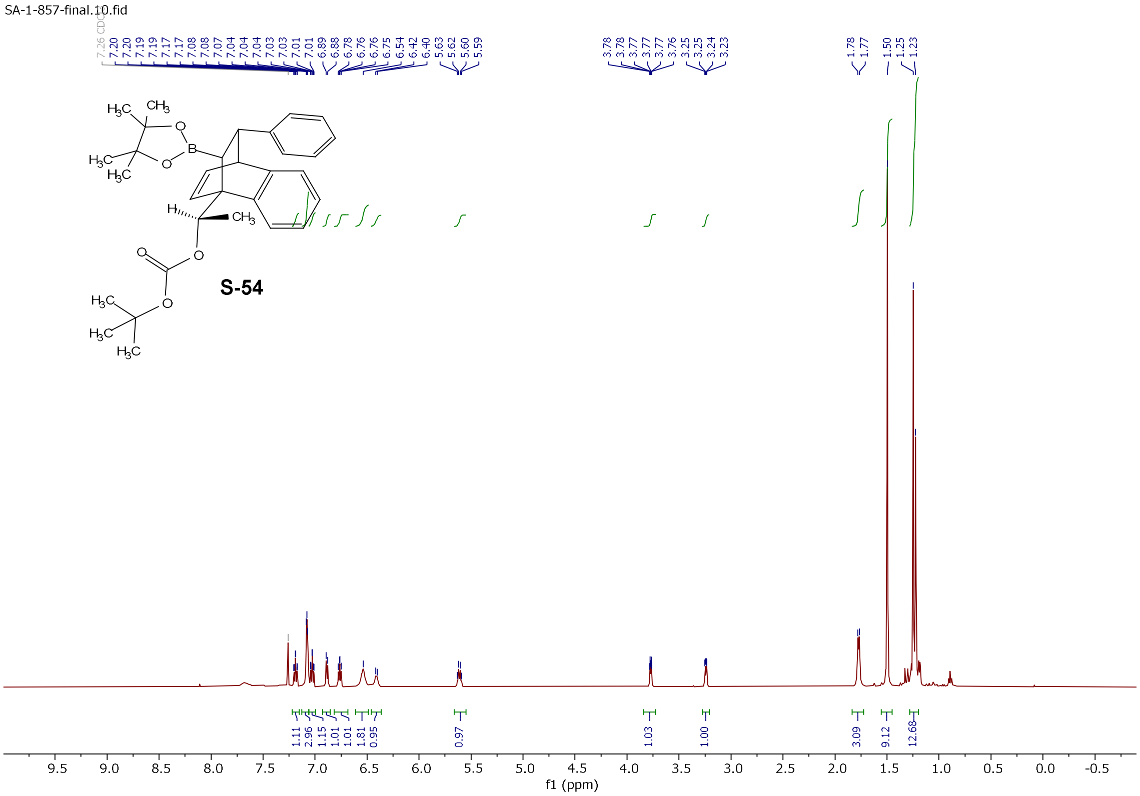
SA-1-857-final.10.fid
7.26 CDCl3
7.20
7.20
7.19
7.19
7.17
7.17
7.08
7.08
7.07
7.04
7.04
7.04
7.03
7.03
7.01
7.01
6.89
6.88
6.78
6.76
6.76
6.75
6.75
6.54
6.42
6.40
5.63
5.62
5.60
5.59
3.78
3.78
3.77
3.77
3.76
3.25
3.25
3.24
3.23
1.78
1.77
1.50
1.25
1.23
S-54
1.11
2.96
1.15
1.01
1.01
1.81
0.95
0.97
1.03
1.00
3.09
9.12
12.68
f1 (ppm)

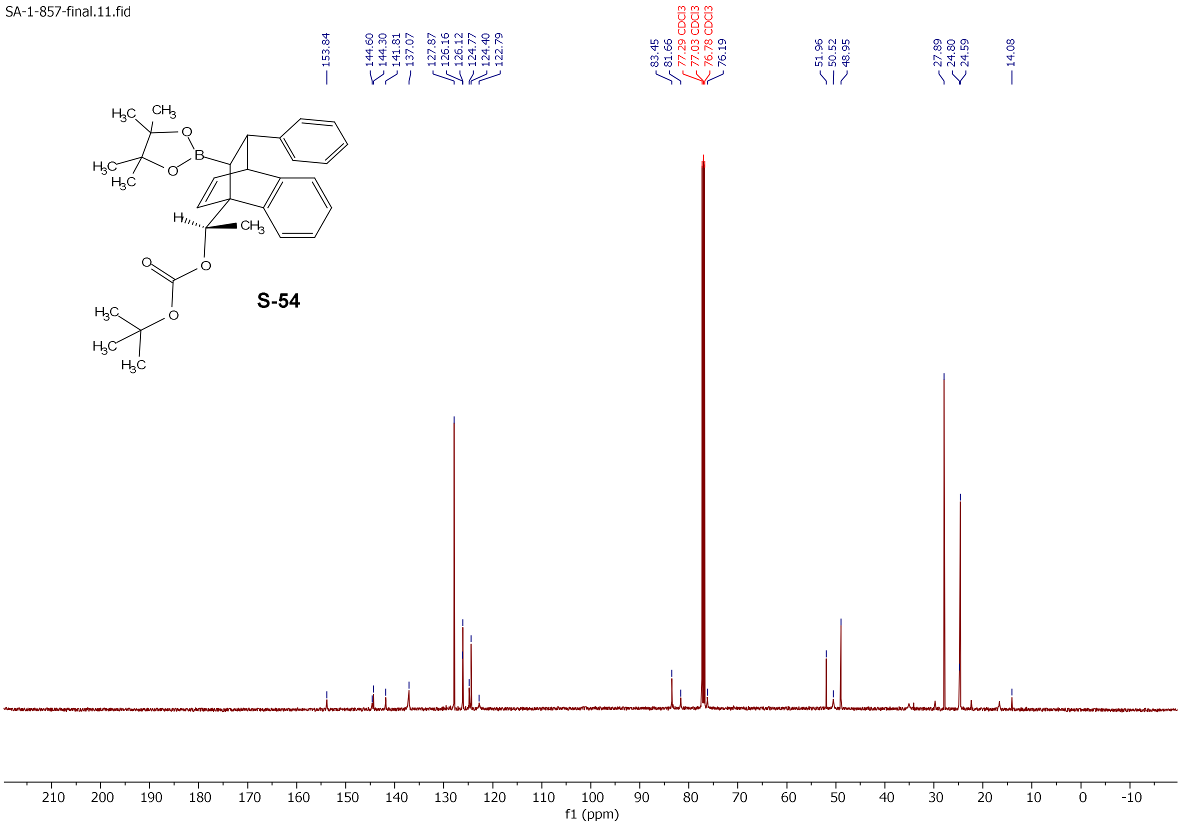

8. References:

[1] https://www.creativelightings.com/aboutus.asp.

[2] Y. Liu, D. Ni, M. K. Brown, *J. Am. Chem. Soc.* **2022**, *144*, 18790-18796.

[3] C. C. Le, M. K. Wismer, Z.-C. Shi, R. Zhang, D. V. Conway, G. Li, P. Vachal, I. W. Davies, D. W. C. MacMillan, *ACS Cent. Sci.* **2017**, *3*, 647-653.

[4] C. Zhang, W. Hu, G. J. Lovinger, J. Jin, J. Chen, J. P. Morken, *J. Am. Chem. Soc.* **2021**, *143*, 14189-14195.

[5] L. Du, Y. Gong, J. Han, X. Xin, H. Luo, Y. Tian, Y. Li, B. Li, *Org. Lett.* **2021**, *23*, 7966-7971.

[6] N. C. Bruno, M. T. Tudge, S. L. Buchwald, *Chem. Sci.* **2013**, *4*, 916-920.

[7] A. G. Amador, E. M. Sherbrook, T. P. Yoon, *Asian J. Org. Chem.* **2019**, *8*, 978-985.

[8] Y. Liu, D. Ni, B. G. Stevenson, V. Tripathy, S. E. Braley, K. Raghavachari, J. R. Swierk, M. K. Brown, *Angew. Chem. Int. Ed.* **2022**, *61*, e202200725.

[9]Same as ref: 2.

[10] S. R. Dubbaka, M. Salla, R. Bolisetti, S. Nizalapur, *RSC Advances* **2014**, *4*, 6496.

[11] J. Gui, H. Xie, H. Jiang, W. Zeng, *Org. Lett.* **2019**, *21*, 2804-2807.

[12] Z. Chen, M. Tan, C. Shan, X. Yuan, L. Chen, J. Shi, Y. Lan, Y. Li, *Angew. Chem. Int. Ed.* **2022**, *61*, e202212160.

[13] J. R. Harrison, S. Brand, V. Smith, D. A. Robinson, S. Thompson, A. Smith, K. Davies, N. Mok, L. S.

Torrie, I. Collie, I. Hallyburton, S. Norval, F. R. C. Simeons, L. Stojanovski, J. A. Frearson, R. Brenk, P. G. Wyatt, I. H. Gilbert, K. D. Read, *J. Med. Chem.* **2018**, *61*, 8374-8389.

[14] W. Xie, B. Gong, S. Ning, N. Liu, Z. Zhang, X. Che, L. Zheng, J. Xiang, *Synlett* **2019**, *30*, 2077-2080.

[15] E. Benedetti, M. Lomazzi, F. Tibiletti, J.-P. Goddard, L. Fensterbank, M. Malacria, G. Palmisano, A. Penoni, *Synthesis* **2012**, *44*, 3523-3533.

[16] F. Xu, S. A. Shuler, D. A. Watson, *Angew. Chem. Int. Ed.* **2018**, *57*, 12081-12085.

[17] X. Ren, A. L. Chandgude, R. Fasan, *ACS Catalysis* **2020**, *10*, 2308-2313.

[18] D. Kaiser, V. Tona, C. R. Gonçalves, S. Shaaban, A. Oppedisano, N. Maulide, *Angew. Chem. Int. Ed.* **2019**, *58*, 14639–14643.

[19] J. Li, J. Li, R. He, J. Liu, Y. Liu, L. Chen, Y. Huang, Y. Li, *Org. Lett.* **2022**, *24*, 1620-1625.

[20] B. Abarca-gonzalez, R. A. Jones, M. Medio-simon, J. Quilez-pardo, J. Sepulveda-arques, E. Zaballos-garcia, *Synthetic Communications* **1990**, *20*, 321-331.

[21] J. Liu, Q. Ren, X. Zhang, H. Gong, *Angew. Chem. Int. Ed.* **2016**, *55*, 15544–15548.

[22] Z. Liu, J. S. A. Ishibashi, C. Darrigan, A. Dargelos, A. Chrostowska, B. Li, M. Vasiliu, D. A. Dixon, S.-Y. Liu, *J. Am. Chem. Soc.* **2017**, *139*, 6082-6085.

[23] worldwide.espacenet.com/patent/search/family/045810668/publication/WO2012033069A1?q=WO2012033069A1

[24] Same as ref. 21.

[25] G. G. Levkovskaya, V. A. Kobelevskaya, E. V. Rudyakova, K. Q. Ha, D. O. Samultsev, I. B. Rozentsveig, *Tetrahedron* **2011**, *67*, 1844-1851.

[26] Z. C. Girvin, L. F. Cotter, H. Yoon, S. J. Chapman, J. M. Mayer, T. P. Yoon, S. J. Miller, *J. Am. Chem. Soc.* **2022**, *144*, 20109-20117.

[27] G. Yue, Q. Liu, J. Wei, Y. Pi, D. Qiu, F. Mo, *J. Org. Chem.* **2023**, *88*, 2735-2741.

[28] K. J. Schwarz, C. Yang, J. W. B. Fyfe, T. N. Snaddon, *Angew. Chem.* **2018**, *130*, 12278–12281.

[29] M. A. E. Pinto-Bazurco Mendieta, M. Negri, Q. Hu, U. E. Hille, C. Jagusch, K. Jahn-Hoffmann, U. Müller-Vieira, D. Schmidt, T. Lauterbach, R. W. Hartmann, *Arch. Pharm. Med. Chem.* **2008**, *341*, 597–609.

[30] G. L. Trammel, P. B. Kannangara, D. Vasko, O. Datsenko, P. Mykhailiuk, M. K. Brown, *Angew. Chem. Int. Ed.* **2022**, *61*, e202212117.

[31] A. K. Simlandy, M.-Y. Lyu, M. K. Brown, *ACS Catal.* **2021**, *11*, 12815-12820.

[32] S. O. Scholz, J. B. Kidd, L. Capaldo, N. E. Flikweert, R. M. Littlefield, T. P. Yoon, *Org. Lett.* **2021**, *23*, 3496-3501.

[33] Same ref.32.

[34] N. Xu, H. Liang, J. P. Morken, *J. Am. Chem. Soc.* **2022**, *144*, 11546-11552.

[35] Same ref. 34.

[36] H. Liang, J. P. Morken, *J. Am. Chem. Soc.* **2023**, *145*, 9976-9981.

[37] SAINT V8.40A (2020), Bruker AXS, Madison, WI.

[38] L. Krause, R. Herbst-Irmer, G. M. Sheldrick, D. Stalke: Comparison of silver and molybdenum microfocus X-ray sources for single-crystal structure determination. J. Appl. Cryst., 48, 3-10 (2015). Doi: 10.1107/S1600576714022985.

[39] G. M. Sheldrick: SHELXT--Integrated space-group and crystal-structure determination. *Acta Cryst*. A71, 3-8 (2015). Doi: 10.1107/S2053273314026370.

[40] G. M. Sheldrick: Crystal structure refinement with SHELXL. *Acta Cryst*. C71, 3-8 (2015). Doi: 10.1107/S2053229614024218.

[41] O. V. Dolomanov, L. J. Bourhis, R. J. Gildea, J. A. K. Howard and H. Puschmann. "OLEX2: a complete structure solution, refinement and analysis program". J. Appl. Cryst. 2009, 42, 339-341.
